# The CINSARC signature predicts the clinical outcome in patients with Luminal B breast cancer

**DOI:** 10.1038/s41523-021-00256-2

**Published:** 2021-05-05

**Authors:** Anthony Goncalves, Pascal Finetti, Daniel Birnbaum, François Bertucci

**Affiliations:** 1 Laboratoire d’Oncologie Prédictive, Centre de Recherche en Cancérologie de Marseille (CRCM), Institut Paoli-Calmettes, INSERM UMR1068, CNRS UMR725 Marseille, France; 2 Département d’Oncologie Médicale, Institut Paoli-Calmettes, Marseille, France; 3 Faculté de Médecine, Aix-Marseille Université, Marseille, France

**Keywords:** Prognostic markers, Breast cancer

## Abstract

CINSARC, a multigene expression signature originally developed in sarcomas, was shown to have prognostic impact in various cancers. We tested the prognostic value for disease-free survival (DFS) of CINSARC in a series of 6035 early-stage invasive primary breast cancers. CINSARC had independent prognostic value in the Luminal B subtype and not in the other subtypes. In Luminal B patients receiving adjuvant endocrine therapy but no chemotherapy, CINSARC identified patients with different 5-year DFS (90% [95%CI 86–95] in low-risk vs. 79% [95%CI 75–84] in high-risk, *p* = 1.04E−02). Luminal B CINSARC high-risk tumors were predicted to be less sensitive to endocrine therapy and CDK4/6 inhibitors, but more vulnerable to homologous recombination targeting and immunotherapy. We concluded that CINSARC adds prognostic information to that of clinicopathological features in Luminal B breast cancers, which might improve patients’ stratification and better orient adjuvant treatment. Moreover, it identifies potential therapeutic avenues in this aggressive molecular subtype.

## Introduction

During the last decades, significant progresses have been achieved in early breast cancer management, most notably through the routine use of post-operative systemic treatment including adjuvant cytotoxic chemotherapy and endocrine therapy^1,2^. Yet, the benefits conferred by these treatments are not uniformly distributed across the various molecular subtypes of disease described from gene expression profiling^3^. Thus, only endocrine receptor (ER)-positive breast cancers benefit from endocrine therapy, whereas cytotoxic chemotherapy, without and with anti-HER2 agents, has maximum efficacy in triple-negative and HER2-positive subtypes, respectively. In ER-positive/HER2-negative breast cancer, the so-called luminal-like breast cancer, only a minor subset of patients, with either a large tumor burden or a highly proliferative and aggressive biology, derive an actual benefit from chemotherapy. Accordingly, various prognostic signatures have been established and made commercially available to help identify these patients, and are now increasingly used in the clinic^4,5^. These signatures distinguish patients with low-, intermediate-, and high-risk of unfavorable outcome, the latter being recommended for adjuvant chemotherapy. Nevertheless, in those patients with molecularly-defined high-risk disease, the level of therapeutic discrepancy remains significant, because some patients receiving adjuvant chemotherapy will relapse and die, while a relatively high number of those high-risk patients could still achieve cure with endocrine therapy alone. Thus, alternative or additional molecular predictors are needed in this population.

The CINSARC (Complexity INdex in SARComas) signature was originally elaborated as a predictor of clinical outcome in soft tissue sarcomas with complex genetics and was subsequently demonstrated to have prognostic impact in different tumor types, including breast cancer^6,7^. CINSARC classifies the tumor samples into high-risk or low-risk of relapse. It includes genes implicated in mitosis and maintenance of chromosomes integrity, the deregulation of which may result in elevated genomic instability. Moreover, aberrant expression of CINSARC proteins was also suggested to favor higher migration and invasion abilities^8,9^. All of these features are associated with increased tumor aggressiveness and may explain the potential of this signature to prognosticate the recurrence of cancer across multiple malignancies.

Regarding the prognostic value of CINSARC in breast cancer, it is necessary to examine how it compares with the classical clinicopathological prognostic features in multivariate analysis. Thus, to further examine the potential prognostic value of CINSARC in breast cancer, we examined a set of 6035 early-stage, invasive primary breast cancers with publicly available gene expression and clinicopathological annotations including survival. We found that CINSARC had independent prognostic value in the Luminal B subtype but not in the other subtypes, notably in patients treated with adjuvant endocrine therapy without chemotherapy, thus identifying a subset of luminal B breast cancer in which therapeutic de-escalation might be possible. In addition, we identified in CINSARC high-risk patients an enrichment in gene signatures associated with response to PARP inhibitors and immunotherapy, thus providing potential clues to treat these poor-prognosis patients.

## Results

### The prognostic value of CINSARC in breast cancer is not independent

**Fig. 1:**
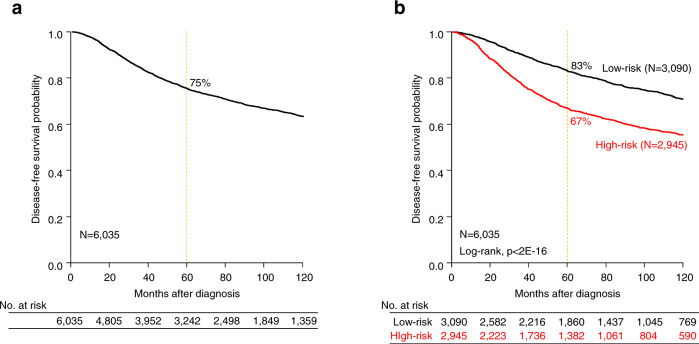
Disease-free survival in early breast cancer patients. **a** Kaplan–Meier disease-free survival (DFS) curve in 6035 informative early breast cancer patients. **b** Similar to **a**, but according to the two CINSARC classes (high-risk vs. low-risk).

We analyzed our database of 8930 patients with early breast cancer, including 6,035 treated with primary surgery and with available DFS data (Supplementary Table 1). With a median follow-up of 77 months (range 1–382), 1759 experienced a DFS event, while 4276 remained disease-free for a 5-year DFS of 75% (95%CI, 74–76) (Fig. 1a). Applying CINSARC to this population identified 2945 CINSARC-high risk (49%) and 3090 CINSARC-low risk (51%) breast cancer patients (Fig. 1b), with significantly different 5-year DFS (67% vs. 83%, respectively; *p* < 2E−16, log-rank test). In univariate analysis (Table 1), CINSARC-high risk patients had an 80% increase in risk of DFS event as compared to the CINSARC-low risk (Hazard Ratio HR = 1.80, 95%CI 1.64–1.98, *p* = 9.44E−34, Wald test). Other variables associated with shorter DFS included younger age, pathological lymph node involvement, type, grade and tumor size, PAM50 molecular subtypes, delivery of adjuvant chemotherapy, and absence of adjuvant endocrine therapy. However, in multivariate analysis, the pathological lymph node involvement, tumor size and type, molecular subtypes and lack of endocrine therapy remained independently associated with survival outcome, whereas CINSARC lost its significance (HR = 1.19, 95%CI 0.97–1.46, *p* = 0.095, Wald test).

**Table 1:** Univariate and multivariate Cox regression analyses for DFS in breast cancer and per molecular subtype.

DFS		Univariate			Multivariate		
		*N*	HR [95% CI]	*p*-value	*N*	HR [95% CI]	*p*-value
All breast cancers							
Patients’ age	>50 vs. ≤50	4707	0.81 [0.72–0.91]	3.20E−04	2265	0.92 [0.76–1.11]	0.384
Pathological tumor type	Lobular vs. ductal	3602	0.95 [0.75–1.21]	2.69E−04	2265	1.45 [1.08–1.95]	1.28E−02
	Other vs. ductal		0.61 [0.47–0.77]		2265	0.65 [0.48–0.88]	5.57E−03
Pathological grade	2 vs. 1	4018	1.68 [1.35–2.09]	2.46E−22	2265	1.12 [0.79–1.59]	0.512
	3 vs. 1		2.56 [2.06–3.17]		2265	1.06 [0.74–1.53]	0.738
Pathological axillary lymph node status	Positive vs. negative	5165	1.63 [1.47–1.82]	3.16E−19	2265	1.83 [1.49–2.23]	4.80E−09
Pathological tumor size	pT2 vs. pT1	4719	1.59 [1.40–1.80]	1.68E−22	2265	1.46 [1.23–1.73]	1.92E−05
	pT3 vs. pT1		2.58 [2.10–3.17]		2265	2.11 [1.56–2.87]	1.64E−06
Adjuvant chemotherapy	yes vs. no	4442	1.52 [1.33–1.73]	6.02E−10	2265	1.14 [0.90–1.43]	0.272
Adjuvant hormone therapy	yes vs. no	4382	0.77 [0.68–0.87]	2.95E−05	2265	0.71 [0.58–0.86]	4.89E−04
PAM50-derived molecular subtype	ERBB2 vs. Basal	6035	1.11 [0.96–1.27]	1.40E−45	2265	1.23 [0.97–1.55]	0.088
	Luminal A vs. Basal		0.45 [0.39–0.52]		2265	0.6 [0.44–0.82]	1.43E−03
	Luminal B vs. Basal		0.85 [0.75–0.97]		2265	1.08 [0.84–1.40]	0.536
	Normal vs. Basal		0.47 [0.39–0.57]		2265	0.67 [0.46–0.98]	4.03E−02
CINSARC classes	High vs. Low-risk	6035	1.80 [1.64–1.98]	9.44E−34	2265	1.19 [0.97–1.46]	0.095
Per molecular subtype							
CINSARC classes in Luminal A	High vs. Low-risk	1753	1.40 [1.03–1.91]	3.40E−02			
CINSARC classes in Luminal B	High vs. Low-risk	1438	1.43 [1.18–1.73]	2.18E−04			
CINSARC classes in Basal	High vs. Low-risk	1241	1.03 [0.76–1.40]	0.841			
CINSARC classes in ERBB2-enriched	High vs. Low-risk	911	1.01 [0.79–1.30]	0.925			
CINSARC classes in Normal-like	High vs. Low-risk	692	1.27 [0.83–1.92]	0.269			

Because prognostic signatures may have clinical interest restricted to specific molecular subtypes, we repeated the same univariate analysis in each PAM50 subtype (Table 1). Interestingly, CINSARC had a significant prognostic impact only in the Luminal subtypes (HR = 1.40 [95%CI 1.03–1.91], *p* = 3.40E−02, and 1.43 [95%CI 1.18–1.73], *p* = 2.18E−04 in Luminal A and Luminal B, respectively). Accordingly, subsequent analyses were focused on these subtypes.

### CINSARC has an independent prognostic value in the Luminal B subtype

Prognostic analyses were done in each Luminal subtype separately. In Luminal A breast cancer (*n* = 1592), the CINSARC high-risk class was associated with higher pathological grade (*p* = 7.60E-05) and tumor size (*p* = 4.00E−02), and with lower 5-year DFS (78%, 95%CI 72–86) as compared to the CINSARC low-risk class (88% [95%CI 86–89], *p* = 3.31E−02, log-rank test; Supplementary Table 2). In univariate analysis, CINSARC high-risk (HR = 1.40 [95%CI 1.03–1.91], *p* = 3.40E−02, Wald test) together with younger age, pathological lymph node involvement, higher grade and tumor size were associated with higher risk of DFS event (Supplementary Table 3). However, in multivariate analysis, CINSARC did not maintain its prognostic value (HR = 1.21 [95%CI 0.77–1.91], *p* = 0,398, Wald test). In addition, no prognostic value of CINSARC was found when focusing on the patients with Luminal A subtype treated with adjuvant endocrine treatment but without adjuvant chemotherapy (Supplementary Table 3).

**Fig. 2:**
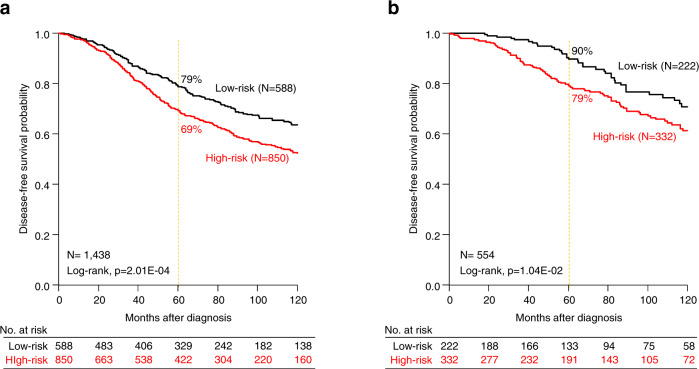
Disease-free survival in Luminal B early breast cancer patients according to CINSARC signature. **a** Kaplan–Meier disease-free survival (DFS) in Luminal B breast cancer patients according to the two CINSARC classes (high-risk versus low-risk) in the overall population. **b** Similar to **a**, but only in patients receiving adjuvant endocrine therapy but no adjuvant chemotherapy.

**Fig. 3:**
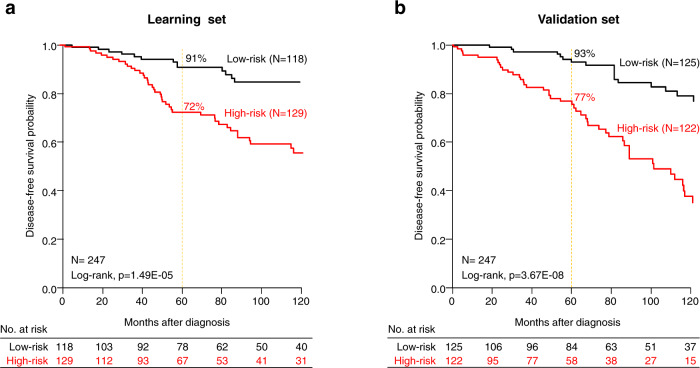
Disease-free survival in Luminal B early breast cancer patients only treated with adjuvant endocrine therapy without adjuvant chemotherapy according to a clinico-genomic model combining CINSARC, pathological tumor size and lymph node status. **a** Kaplan–Meier disease-free survival (DFS) in Luminal B breast cancer patients according to the two classes defined by the model (high-risk versus low-risk) in the learning set. **b** Similar to **a**, but in the validation set.

The results were different in the Luminal B subtype (*n* = 1438). The CINSARC high-risk class was associated with higher pathological grade (*p* = 2.18E−04) and lymph node involvement (*p* = 1.18E−02) (Table 2), and shorter DFS with 69% 5-year DFS (95%CI 66–73) as compared to the CINSARC low-risk class (79% [95%CI 75–83], *p* = 2.01E−04, log-rank test; Fig. 2a). Importantly and by contrast with what was observed in the Luminal A subtype, CINSARC demonstrated significant prognostic value in both univariate (HR = 1.43 [95%CI 1.18–1.73], *p* = 2.18E−04, Wald test) and multivariate analyses (HR = 1.46 [95%CI, 1.09–1.96], *p* = 1.20E−02, Wald test) (Table 3). Other clinicopathological features independently associated with shorter DFS included pathological lymph node involvement, tumor size, and type. Such prognostic complementarity between the clinicopathological variables and CINSARC was tested using the likelihood ratio (LR) test: CINSARC added prognostic information to that provided by the combination of clinicopathological variables (ΔLR-_X_^2^ = 6.53, *p* = 1.06E−02). Because a major aim of using a prognostic signature in Luminal breast cancer is therapeutic de-escalation, we assessed the prognostic value of CINSARC in the 554 Luminal B patients treated with adjuvant endocrine therapy only, without adjuvant chemotherapy. As shown in Fig. 2b, CINSARC identified 222 low-risk Luminal B patients with 90% 5-year DFS (95%CI 86–95), significantly better than the CINSARC high-risk patients (79% 5-year DFS [95%CI, 75–84]; *p* = 1.04E−02, log-rank test). Of note in this population also, CINSARC had independent prognostic value in multivariate analysis (HR = 1.62 [95%CI 1.11–2.37], *p* = 1.16E−02, Wald test), together with pathological lymph node involvement and tumor size (Table 3), and added independent prognostic information to these clinicopathological features (ΔLR-_X_^2^ = 6.71, *p* = 9.58E−03). We built a prognostic clinico-genomic model based on these three variables in a randomly defined learning set of 247 samples and tested its prognostic value in the validation set of 247 remaining samples: as shown in Fig. 3, the model was robust and identified an even lower risk subgroup with 5-year DFS of 93% (95%CI [89–97]).

**Table 2:** Correlations of CINSARC classes with clinicopathological characteristics in Luminal B breast cancer patients.

Characteristics		CINSARC classes			
		*N*	Low-risk	High-risk	*p*-value
Patients’ age					0.233
≤50		283	104 (23%)	179 (26%)	
>50		843	345 (77%)	498 (74%)	
Pathological tumor type					0.178
Ductal		723	289 (80%)	434 (81%)	
Lobular		76	26 (7%)	50 (9%)	
Other		96	46 (13%)	50 (9%)	
Pathological grade					3.13E−04
1		65	35 (9%)	30 (5%)	
2		427	198 (51%)	229 (42%)	
3		446	156 (40%)	290 (53%)	
Pathological axillary lymph node status					1.18E−02
Negative		676	295 (60%)	381 (52%)	
Positive		545	199 (40%)	346 (48%)	
Pathological tumor size					0.322
pT1		398	169 (37%)	229 (33%)	
pT2		664	252 (55%)	412 (59%)	
pT3		91	38 (8%)	53 (8%)	
Adjuvant chemotherapy					0.012
No		880	375 (83%)	505 (77%)	
Yes		230	77 (17%)	153 (23%)	
Adjuvant hormone therapy					0.709
No		457	188 (42%)	269 (41%)	
Yes		647	258 (58%)	389 (59%)	
Follow-up median, months (min–max)		1438	71 (0–243)	63 (0–294)	0.677
DFS event (%)		1438	164 (28%)	309 (36%)	9.19E−04
5-year DFS		1438	79% [75–83]	69% [66–73]	2.01E−04

**Table 3:** Univariate and multivariate Cox regression analyses for DFS in Luminal B breast cancer patients.

All patients		Univariate			Multivariate		
		*N*	HR [95% CI]	*p*-value	*N*	HR [95% CI]	*p*-value
Patients’ age	>50 vs. ≤50	1126	0.66 [0.52–0.84]	5.73E−04	759	0.69 [0.47–1.03]	0.070
Pathological tumor type	lobular vs. ductal	895	1.86 [1.21–2.84]	1.47E−02	759	2.17 [1.38–3.39]	7.18E-04
	other vs. ductal		0.94 [0.60–1.47]		759	1.19 [0.73–1.92]	0.485
Pathological grade	2 vs. 1	938	1.23 [0.75–2.00]	0.077			
	3 vs. 1		1.51 [0.93–2.46]				
Pathological axillary lymph node status	positive vs. negative	1221	1.69 [1.38–2.07]	4.75E−07	759	2.55 [1.79–3.63]	1.83E−07
Pathological tumor size	pT2 vs. pT1	1153	1.48 [1.17–1.88]	2.24E−07	759	1.52 [1.10–2.10]	1.14E−02
	pT3 vs. pT1		2.96 [1.99–4.38]		759	3.25 [1.89–5.58]	2.09E−05
Adjuvant chemotherapy	yes vs. no	1110	1.51 [1.13–2.01]	5.41E−03	759	1.08 [0.72–1.64]	0.702
Adjuvant hormone therapy	yes vs. no	1104	0.74 [0.60–0.92]	6.93E−03	759	0.69 [0.47–0.99]	4.48E−02
CINSARC classes	High vs. Low-risk	1438	1.43 [1.18–1.73]	2.18E−04	759	1.46 [1.09–1.96]	1.20E−02

Patients with adjuvant HT and without adjuvant CT		Univariate			Multivariate		
		*N*	HR [95% CI]	*p*-value	*N*	HR [95% CI]	*p*-value
Patients’ age	>50 vs. ≤50	517	0.99 [0.50–1.96]	0.985			
Pathological tumor type	lobular vs. ductal	482	1.71 [0.94–3.12]	0.195			
	other vs. ductal		0.92 [0.49–1.71]				
Pathological grade	2 vs. 1	407	0.84 [0.38–1.85]	0.871			
	3 vs. 1		0.81 [0.37–1.78]				
Pathological axillary lymph node status	positive vs. negative	535	2.88 [1.98–4.19]	3.13E−08	494	2.9 [1.91–4.40]	6.23E−07
Pathological tumor size	pT2 vs. pT1	513	1.87 [1.24–2.82]	1.76E−06	494	1.76 [1.16–2.66]	7.68E−03
	pT3 vs. pT1		5.21 [2.76–9.83]		494	4.71 [2.47–8.98]	2.41E−06
CINSARC classes	High vs. Low-risk	554	1.60 [1.11–2.30]	1.11E−02	494	1.62 [1.11–2.37]	1.16E−02

We had previously shown the prognostic complementarity and independence for DFS of commercial prognostic proliferation-based signatures (70_gene, Recurrence Score, ROR-P) and the ICR immune signature^10^. Thus we tested such independence between CINSARC and immune signatures including the Palmer’s metagenes (B-cells, T-cells, and CD8 T-cells)^11^, the Rooney’ cytolytic activity score^12^, and the three signatures predictive for response to immune therapy (ICR, TIS, and TLS). Multivariate analysis (Table 4) showed that in each case, CINSARC remained significant as well as each immune signature, suggesting independent prognostic value.

**Table 4:** Multivariate Cox regression analyses for DFS in Luminal B breast cancer including immune signatures.

DFS	Multivariate		
	*N*	HR [95%CI]	*p*-value
ICR score	1438	0.84 [0.73–0.97]	1.95E−02
CINSARC classes	1438	1.47 [1.22–1.78]	7.29E−05
TIS score	1438	0.89 [0.79–1.01]	0.074
CINSARC classes	1438	1.46 [1.20–1.76]	1.09E−04
TLS score	1438	0.83 [0.72–0.95]	8.46E−03
CINSARC classes	1438	1.47 [1.22–1.78]	6.79E−05
Palmer, B-cells module	1438	0.32 [0.23–0.45]	4.62E−11
CINSARC classes	1438	1.44 [1.19–1.74]	1.50E−04
Palmer, CD8 T-cells module	1438	0.60 [0.53–0.68]	3.58E−15
CINSARC classes	1438	1.43 [1.19–1.73]	1.90E−04
Palmer, T-cells module	1438	0.35 [0.25–0.48]	2.32E−10
CINSARC classes	1438	1.45 [1.20–1.75]	1.19E−04
Rooney, Cytolytic activity	1438	0.86 [0.77–0.95]	3.09E−03
CINSARC classes	1438	1.45 [1.20–1.75]	1.37E−04

### CINSARC classes and therapeutic vulnerability in the Luminal B subtype

Beyond prognostication, multigene signatures may also help identify therapeutic targets that might improve survival of patients with high risk of recurrence. Thus, in the whole population of 2028 Luminal B patients of our database, we wondered whether the two CINSARC classes displayed different probabilities of response to specific systemic therapies routinely used or under development in breast cancer (Table 5).

**Table 5:** Correlations of CINSARC classes with therapeutic response/vulnerability in Luminal B breast cancers.

Therapies	Characteristics		*N*	CINSARC classes		*p*-value
				Low-risk	High-risk	
Chemotherapy	107-gene signature	No pCR-like	1587	663 (83%)	924 (75%)	1.43E−04
		pCR-like	441	140 (17%)	301 (25%)	
	pCR	No pCR	124	49 (88%)	75 (80%)	0.270
		pCR	26	7 (12%)	19 (20%)	
Hormone therapy	E2F4-activation signature	Low	496	348 (43%)	148 (12%)	3.73E−57
		High	1532	455 (57%)	1077 (88%)	
CDK4/6 inhibitors	RBsig signature	Score	2028	0.01 (−0.9–1.6)	0.44 (−0.6–2.6)	1.57E−100
	E2F regulon signature	Score	2028	0.11 (−0.6–0.6)	0.3 (−0.5–0.73)	5.36E−56
PARP inhibitors	HRD signature	Low	248	94 (94%)	154 (81%)	2.59E−03
		High	42	6 (6%)	36 (19%)	
Immune checkpoint inhibitorss	ICR signature	Score	2028	−0.33 (−2.31–2.6)	−0.21 (−1.8–3.1)	8.07E−04
	TIS signature	Score	2028	−0.35 (−2.3–2.2)	−0.24 (−2.1–2.8)	2.68E−03
	TLS signature	Score	2028	−0.32 (−2.2–1.8)	−0.17 (−3.2–2.3)	1.69E−05
IA ESCAT alterations	*ERBB2* amplification^1^	No	764	305 (100%)	459 (98%)	0.156
		Yes	8	1 (0%)	7 (2%)	
	*PIK3CA* mutation	No	575	219 (72%)	356 (76%)	0.151
	(E542K, E545K/A, H1047R/L)	Yes	197	87 (28%)	110 (24%)	
IIA ESCAT alterations	*ESR1* mutation	No	772	306 (100%)	466 (100%)	—
	(E380Q, Y537S/C/N, D538G)	Yes	0	0 (0%)	0 (0%)	
	*PTEN* loss^2^	No	766	305 (100%)	461 (99%)	0.411
		Yes	6	1 (0%)	5 (1%)	
IIB ESCAT alterations	*AKT1* mutation	No	752	296 (97%)	456 (98%)	0.361
	(E17K)	Yes	20	10 (3%)	10 (2%)	
	*ERBB2* mutation^3^	No	761	302 (99%)	459 (98%)	1
		Yes	11	4 (1%)	7 (2%)	

pCR: pathological complete response; ^1^: >= 6 copies; ^2^homozigous deletion, truncated mutations and kown inactivating missense mutations (e.g., R130Q/G); ^3^hotspot activating missense mutations (e.g., S310F/Y, L755S, V777L), inframe insertion exon 2 O (e.g., Y772_A775dup).

Regarding chemotherapy, 94 CINSARC high-risk and 56 CINSARC low-risk cases were informative about achievement or not of a pathological complete response (pCR) after anthracycline/taxane-based neoadjuvant chemotherapy. High-risk patients had a numerical but non-statistically significant increase in pCR rate (20%) as compared to low-risk patients (12%, *p* = 0.270, Fisher exact test). Close percentages were observed when considering the probability of pCR as defined using an expression signature of pathological response to neoadjuvant chemotherapy in breast cancer^13^: 25% of high-risk patients were predicted with pCR versus 17% of low-risk patients, and the difference was significant (*p* = 1.43E−04). By contrast, CINSARC high-risk patients were associated with a lower probability of sensitivity to hormone therapy (88%) according to the E2F4-activation signature^14^, as compared to CINSARC low-risk patients (57%; *p* = 3.73E−57). Altogether, these results suggested that CINSARC high-risk patients might be more sensitive to chemotherapy and less sensitive to hormone therapy than low-risk patients.

We also examined the potential vulnerabilities of CINSARC classes to targeted therapies, using predictive gene signatures. We observed higher RBsig^15^ and E2F regulon^16^ scores in high-risk patients (*p* = 1.57E−100 and p = 3.36E−56, respectively), suggesting higher probability of RB1-pathway inactivation and of resistance to CDK4/6 inhibitors. Conversely, high-risk patients displayed a signature predictive of Homologous Recombination Deficiency^17^ more frequently than low-risk patients (19% versus 6%: *p* = 2.59E−03), which may indicate higher sensitivity to PARP inhibitors. We also compared the proportion of patients in each class displaying an actionable genomic alteration with high evidence level according to ESCAT scale^18^. There was no significant difference between CINSARC high-risk and low-risk patients regarding the proportion of ESCAT I level alterations (*ERBB2* amplification, and *PIK3CA* mutation), and ESCAT II level alterations (*ESR1* mutation, *PTEN* loss, *AKT1* mutations, *ERBB2* mutation).

Finally, high-risk patients displayed higher score for three signatures associated with response to immune checkpoint inhibitors: ICR^19^ (*p* = 8.07E−04), TIS^20^ (*p* = 2.68E−03), and TLS signature^21^ (*p* = 1.69E−05), suggesting a potential higher response to immunotherapy in Luminal B CINSARC high-risk patients than low-risk patients.

### Biological correlates of CINSARC classes in the Luminal B subtype

To further elucidate the biological differences between the two CINSARC classes and identify potential therapeutic targets, we compared their whole-exome mutational, whole-genome copy number and transcriptional, and proteomic (RPPA) profiles by using the TCGA dataset, which included 297 Luminal B cases. No genomic alteration was differentially mutated, deleted or amplified between the two classes (Supplementary Tables 4–6). A total of 510 genes were differentially expressed between the two classes (Supplementary Fig. 1, Supplementary Table 7). The robustness of this gene list was confirmed in the METABRIC independent validation set, and ontology analysis revealed a large preponderance of mitotic processes, including mitotic spindle assembly and chromosomal segregation, and DNA repair among the genes upregulated in the high-risk class (Supplementary Table 8). Proteomic analysis using RPPA results identified 16 proteins with differential expression between the two CINSARC classes (Table 6, Supplementary Table 9), including proteins involved in the cell cycle (cyclin B1, p27^kip1^, cyclin E2), cell proliferation (FOXM1 and its 14-3-3_zeta regulator^22^, ASNS^23^), DNA repair (KU80, RAD50, ERCC5, MSH6), AKT/mTOR pathway (4E-BP1, p70S6K), and epigenetic regulator (GCN5L2).

**Table 6:** List of 16 proteins/phosphoproteins differentially expressed between the two CINSARC classes in Luminal B TCGA breast cancers.

Gene#Protein	CINSARC, high- vs. low-risk				Expression status
	*N*	Odds ratio [95%CI]	*p*-value	*q*-value	
CCNB1#Cyclin_B1	240	1.66 [1.41–1.96]	6.53E−07	1.46E−04	up CINSARC high-risk
MSH6#MSH6	240	1.32 [1.19–1.47]	1.38E−05	1.03E−03	up CINSARC high-risk
FOXM1#FoxM1	240	1.31 [1.20–1.44]	2.31E−06	2.58E−04	up CINSARC high-risk
RPS6KB1#p70S6K	240	1.28 [1.11–1.47]	3.86E−03	0.086	up CINSARC high-risk
SYK#Syk	240	1.26 [1.10–1.44]	4.65E−03	0.093	up CINSARC high-risk
ENY2#ENY2	218	1.23 [1.10–1.37]	2.46E−03	0.063	up CINSARC high-risk
YWHAZ#14-3-3_zeta	240	1.20 [1.09–1.33]	2.21E−03	0.063	up CINSARC high-risk
ASNS#ASNS	240	1.20 [1.08–1.33]	5.48E−03	0.093	up CINSARC high-risk
KAT2A#GCN5L2	218	1.19 [1.07–1.32]	6.22E−03	0.093	up CINSARC high-risk
XRCC5#Ku80	240	1.15 [1.06–1.25]	6.65E−03	0.093	up CINSARC high-risk
CCNE2#Cyclin_E2	240	1.13 [1.06–1.20]	1.10E−03	0.061	up CINSARC high-risk
EIF4EBP1#4E-BP1_pT70	240	1.12 [1.05–1.20]	6.61E−03	0.093	up CINSARC high-risk
CDKN1B#p27_pT198	240	1.09 [1.03–1.14]	6.07E−03	0.093	up CINSARC high-risk
RAD50#Rad50	240	0.88 [0.83–0.94]	1.71E−03	0.063	down CINSARC high-risk
ERCC5#ERCC5	240	0.87 [0.81–0.94]	2.17E−03	0.063	down CINSARC high-risk
MAPK8#JNK_pT183_pY185	240	0.86 [0.79–0.93]	2.56E−03	0.063	down CINSARC high-risk

## Discussion

By examining the prognostic value of CINSARC signature in a large population of early breast cancers, we found that CINSARC was independently associated with survival outcome in the Luminal B subtype. In this subtype, CINSARC also identified potential vulnerabilities to specific therapeutics, including innovative classes of compounds that have been recently approved in HER2-negative breast cancer, as well as biological features that could be exploited as future therapeutic targets. These results may provide insights in the clinical development and use of prognostic signatures, and open perspectives for a further stratified management of breast cancer.

First, our study reinforces the need for integrating any new prognostic multigene signature together with other important clinical and biological features which are specifically related to a given tumor type. CINSARC signature was recently demonstrated to outperform histological grade in predicting metastatic outcome in soft tissue sarcomas^6,24^, and is currently prospectively tested to guide treatment in these tumors. CINSARC was also demonstrated to have prognostic value in various other tumor types and was proposed as a universal prognostic biomarker^7^. Based on a multivariate analysis involving several hundreds of clinically and biologically annotated breast cancers, our results demonstrate that CINSARC is not independently associated with survival in this disease and that its prognostic importance is dependent on the molecular subtypes. Thus, it is likely that in ERBB2-positive and basal-like breast cancers, other drivers than CINSARC genes are prominently leading the metastatic process, while in Luminal A breast cancers estrogen receptor signaling plays a major role. In Luminal B, the main biological processes that are captured by CINSARC, such as mitosis and chromosomal instability, may be of particular interest to predict clinical outcomes. And multivariate analyses showed that such prognostic value was also independent from that of immune signatures, clearly suggesting that mitosis and chromosomal instability and immune response provide complementary prognostic information. Of course because of a few limitations inherent to retrospective studies and associated biases), further validation in larger and prospective studies is warranted.

Second, while Luminal B breast cancer is thought to be an aggressive subtype and thus is almost always candidate to adjuvant chemotherapy, CINSARC also allowed identifying a population of patients with favorable outcome while only receiving adjuvant endocrine treatment without any adjuvant chemotherapy. In addition, combining CINSARC with clinical features, such as tumor size and lymph node status, identified a low-risk class of patients with a 93% probability of being disease-free at 5 years. All current prognostic signatures in breast cancer aim to separate low-risk patients, in which adjuvant chemotherapy may be safely spared and endocrine therapy alone may guarantee a high level of cure, from high-risk patients, in which endocrine treatment is not enough and adjuvant chemotherapy should be added. Yet, in the latter subgroup, 60–70% of patients would still be cured by endocrine treatment alone, which represents a high level of residual therapeutic inadequacy. Thus, CINSARC could be helpful in detecting those patients with “low-risk” Luminal B subtype in which the benefit of adjuvant chemotherapy remains questionable and might be replaced by alternative less toxic approaches.

Third, CINSARC also revealed potential therapeutic vulnerabilities in Luminal B breast cancers that may impact the future management of this hard-to-treat subtype. We found that CINSARC high-risk tumors were predicted to be more sensitive to chemotherapy but more resistant to endocrine therapy. Importantly, these high-risk tumors were associated with RB1 inactivation, indicating a higher probability of resistance to CDK4/6 inhibitors^25^, a therapeutic class improving survival in ER/PR-positive/HER2-negative advanced breast cancers and currently under investigation in the adjuvant setting^26–32^. Therefore, a low-risk CINSARC signature could identify Luminal B breast cancers with both relatively favorable outcome and relative resistance to chemotherapy, but with sensitivity to endocrine therapy and CDK4/6 inhibitors, making this combination an attractive alternative to evaluate in this population. Moreover, in accordance with its tight biological relationship with chromosomal instability and rearrangements, we found that CINSARC signature predicted higher sensitivity to both DNA repair- and immune-targeting therapeutics. Thus, high-risk CINSARC tumors were found to display more frequently a high HRD score (in nearly 20% of patients). Although PARP inhibitors were only approved in HER2-negative advanced breast cancer with germline *BRCA1/2* mutation (gBRCAm)^33,34^, including half of patients displaying ER-positive tumors, clinical trials are now underway to evaluate these compounds in other genetic contexts. Thus, CINSARC might contribute to better identify these tumors displaying gBRCA wild-type but HRD features that may also prove to be sensitive to PARP inhibitors and other DNA repair targeting therapeutics. High-risk patients were also predicted to be more sensitive to immunotherapy. Essentially developed in triple-negative breast cancer, with promising results in both advanced and early settings^35,36^, recent data indicate that immune checkpoint inhibitors might be also active in ER-positive breast cancer^37^, thus, CINSARC could be useful to identify those Luminal B patients who could be candidate to PD1/PD-L1 targeting agents.

Finally, our study also allowed describing biological features associated with CINSARC in Luminal B breast cancer and thus proposing new therapeutic avenues in the field. As expected, genes and proteins associated with high-risk signature were involved in mitotic processes, chromosomal segregation, cell cycle and proliferation, as well as DNA repair. Interestingly, cyclin E2 protein was found to be up-regulated in high-risk tumors. Both cyclin E2 and cyclin E1 are able to complex with CDK2 through G1-to-S-phases, allowing RB1 phosphorylation and thus cell cycle progression, and both were shown to promote resistance to endocrine treatment^38^ and CDK4/6 inhibitors^13,39^. Importantly, high cyclin E2 expression may predict activity of CDK2-targeted approaches that are in development, either as specific CDK2 inhibitors or pan-CDK inhibitors that include CDK2 in their spectrum of activity^40^. Other potentially actionable proteins upregulated in CINSARC high-risk tumors include 4E-BP1 and p70S6K, which are downstream effectors of mTOR and AKT pathways, respectively. While mTOR inhibitor everolimus has been registered in endocrine treatment-resistant advanced breast cancer and is under investigation in high-risk early breast cancer^41^, several AKT inhibitors are currently evaluated in advanced breast cancer, including endocrine treatment-resistant luminal disease^42^. Ultimately, CINSARC high-risk tumors may represent a favorable subpopulation to investigate those compounds in the early setting. Of note, histone acetyl transferase GCN5L2, which was shown to regulate TGFβ signaling pathway and induce expression of epithelial-mesenchymal transition^43^, was also upregulated in high-risk tumors and may indicate a potential for epigenetic treatment in this subtype.

In conclusion, we found that CINSARC, a multigene signature initially developed in sarcomas, has an independent prognostic value in breast cancer restricted to the Luminal B subtype. CINSARC may not only identify a subgroup of tumors with relatively favorable outcome, which may not require adjuvant chemotherapy, but also suggests clues to better select patients with a higher probability of benefit from therapeutics under investigation in early breast cancer, such as cell cycle inhibitors, DNA repair targeting agents, immune checkpoint inhibitors, AKT/mTOR inhibitors, and epigenetic regulating agents.

## Methods

### Breast cancer samples and molecular profiling

We analyzed our breast cancer gene expression database^10^ pooled from 36 public datasets (Supplementary Table 10), comprising 8982 invasive breast cancer samples. The details of Institutional Review Board and Ethical Committee approval and patients’ consent for all 36 studies are present in their corresponding publications listed in Supplementary Table 10. Our study is based upon public data from published studies in which ethics approval and informed consent to participate were already obtained by authors. This study was approved by our institutional review board (Comité d’Orientation Stratégique, COS). Gene expression profiles had been generated using DNA microarrays and RNA-Seq, and collected from the National Center for Biotechnology Information (NCBI)/Genbank GEO and ArrayExpress databases, and authors’ website. The final pooled data set contained 8930 non-redundant non-metastatic, non-inflammatory, primary, invasive breast cancers. Before analysis, data were processed as previously described^10^. Briefly, the pre-analytic processing first included normalization of each data set separately, and was done by Robust Multi-Array (RMA) with the oligo R package (version 1.46.0) for Affymetrix data and by quantile normalization with the limma R package (version 3.38.3) for other microarray platforms. When multiple probes mapped to the same GeneID, we retained the one with the highest variance in each data set. We log2-transformed the already normalized TCGA RNAseq data. We also collected DNA and proteomic processed data from TCGA (whole-exome sequencing (WES), array-CGH and HRD score, and RPPA) and METABRIC (targeted-NGS, array-CGH).

### Analysis of molecular profiles

To avoid biases related to trans-institutional immunohistochemical analyses and thanks to the bimodal distribution of respective mRNA expression levels, the ER, progesterone receptor (PR), and HER2 statutes (negative/positive) were defined on transcriptional data of *ESR1*, *PGR*, and *HER2* respectively, as previously described^44^. In addition to the CINSARC signature^6^, we applied to each dataset separately several multigene signatures: PAM50^5^ allowing to define the Luminal A, Luminal B, ERBB2-enriched, Basal, and Normal subtypes, immune signatures including the Palmer’s B-cell, T-cell, and CD8+ T-cell signatures^11^, and the Rooney’ cytolytic activity score^12^, 107-gene signature predictive for pathological response to anthracycline-based neoadjuvant chemotherapy in breast cancer^13^, E2F4-activation signature predictive for response to hormone therapy in breast cancer^14^, Rbsig^15^ and E2F regulon^16^ signatures predictive for resistance to CDK4/6 inhibitors on breast cancer pre-clinical models^14^ and clinical samples of PALOMA-3 trial^16^, and immune signatures predictive for response to immune checkpoint inhibitors: ICR (Immune Constant of Rejection)^19^ and TIS (T cell-inflamed signature)^20^ signatures and a TLS (tertiary lymphoid structures) signature^21^.

We also compared the molecular profiles of CINSARC high-risk versus low-risk Luminal B samples by applying supervised analyses to TCGA and METABRIC data sets at different levels: WES mutational, copy number alterations (CNA), and RPPA data using logistic regression with significance thresholds of *p* ≤ 0.05 and *q* ≤ 0.10, and transcriptional data using moderated t-test with significance thresholds of fold-change |FC | > 1.5, *p* ≤ 0.05 and *q* ≤ 0.10. This later used the TCGA set as learning set and the METABRIC set as independent validation set. Ontology analysis of the resulting gene list was based on the GO biological processes of the Database for Annotation, Visualization and Integrated Discovery (DAVID; david.abcc.ncifcrf.gov/).

### Statistical analysis

Correlations between tumor classes and clinicopathological variables were analyzed using the one-way analysis of variance (ANOVA) or the Fisher’s exact test when appropriate. Disease-free survival (DFS) was calculated from the date of diagnosis until the date of disease recurrence or death from any cause. Follow-up was measured from the date of diagnosis to the date of last news for event-free patients. Survivals were calculated using the Kaplan–Meier method and curves were compared with the log-rank test. Uni- and multivariate prognostic analyses were done using Cox regression analysis (Wald test). The variables submitted to univariate analyses included patients’ age at diagnosis (≤50 years vs > 50), pathological type (lobular *vs* ductal *vs* other), pathological axillary lymph node status (pN: negative *vs* positive), pathological tumor size (pT1 vs pT2 vs pT3), pathological grade (1 vs 2 vs 3), PAM50-derived molecular subtypes (Luminal A vs Luminal B vs Normal vs Basal vs ERBB2-enriched), delivery of adjuvant chemotherapy (CT), delivery of adjuvant hormone therapy (HT), and CINSARC-based classifications. The likelihood ratio (LR) tests were used to assess the prognostic information provided beyond that of a clinical model, assuming a _X_^2^ distribution. Changes in the LR values (LR-ΔX^2^) measured quantitatively the relative amount of information of one model compared with another. All statistical tests were two-sided at the 5% level of significance. In the case of multiple testing, the p-values were replaced by the corrected q-values. Statistical analysis was done using the survival package (version 2.30) in the R software (version 2.9.1; http://www.cran.r-project.org/). We followed the reporting REcommendations for tumor MARKer prognostic studies (REMARK criteria)^45^.

### Reporting summary

Further information on research design is available in the Nature Research Reporting Summary linked to this article.

## Supplementary information

Supplementary Information

Reporting Summary

## Data Availability

The data generated and analyzed during this study are described in the following data record: 10.6084/m9.figshare.14350871^46^. All data sets of primary breast cancer were downloaded from the Gene Expression Omnibus (GEO, https://www.ncbi.nlm.nih.gov/geo/), ArrayExpress (https://www.ebi.ac.uk/arrayexpress/), Genomic Data Commons (GDC, https://portal.gdc.cancer.gov/) and cBioPortal (https://www.cbioportal.org/) databases. All accession IDs are provided in Supplementary Table 10 (Table S10 revised.xlsx), which is included with the data record. The data underlying the figures and tables are contained in the files ‘Goncalves_supporting_data.xlsx’ and ‘Table S8.xlsx’, which are included with the data record. A detailed list of the data underlying each figure and table is also available in the file ‘Goncalves_2021_underlying_data_list.xlsx’, which is included with the data record.

## Code availability

Normalization of public data sets were done by Robust Multi-Array (RMA) with the oligo R package (version 1.46.0) for Affymetrix data and by quantile normalization with the limma R package (version 3.38.3) for other microarray platforms. Supervised analysis was done using a moderated t-test with empirical Bayes statistic included in the limma R package (version 3.38.3). For correction of the multiple-testing hypothesis, False Discovery Rate (FDR) was assessed using qvalue R package (version 2.14.1) (Storey et al., Annals of Statistics, 2003). Several multigene signatures were applied to each dataset separately: CINSARC^6^, PAM50^5^, and 107-gene predictive signatures^13^, who were based on nearest-centroid classification using genes, data and distance method described in each respective study. Also were applied Rbsig^15^, E2F regulon^16^, ICR^19^, TIS^20^, TLS^21^, Palmer’s immune modules (B-cells, T-cells, and CD8 T-cells)^11^, and the Rooney’ cytolytic activity score^12^ signatures who were based on a Z-score metagene using gene list described in each respective study. Statistics analysis was done with the stats R package (version 3.5.2) and the survival R package (version 3.1-12) for survival analysis.
