# Supplementary material for: The CINSARC signature predicts the clinical outcome in patients with Luminal B breast cancer
Source: NPJ Breast Cancer. 2021 May 5;7:48. doi: 10.1038/s41523-021-00256-2 (PMC8099860; doi:10.1038/s41523-021-00256-2)
Supplement: Supplementary file 1 — Supplementary Information [file 41523_2021_256_MOESM1_ESM.pdf]

Supplementary Table 1: Clinicopathological characteristics of 6,035 patients and tumor

Characteristics	All breast cancers N (%)	Luminal B subtype N (%)
Patients' age		
≤ 50	1584 (34%)	283 (25%)
> 50	3123 (66%)	843 (75%)
Pathological tumor type		
ductal	2770 (77%)	723 (81%)
lobular	397 (11%)	76 (8%)
other	435 (12%)	96 (11%)
Pathological grade		
1	548 (14%)	65 (7%)
2	1623 (40%)	427 (46%)
3	1847 (46%)	446 (47%)
Pathological axillary lymph node status		
negative	2984 (58%)	676 (55%)
positive	2181 (42%)	545 (45%)
Pathological tumor size		
pT1	1878 (40%)	398 (35%)
pT2	2443 (52%)	664 (58%)
pT3	398 (8%)	91 (7%)
Adjuvant chemotherapy		
no	3185 (72%)	880 (79%)
yes	1257 (28%)	230 (21%)
Adjuvant hormone therapy		
no	2488 (57%)	457 (41%)
yes	1894 (43%)	647 (59%)
Follow-up median, months (min-max)	77 (1-382)	68 (1-294)
DFS event (%)	1759 (29%)	473 (38%)
5-year DFS	75% [74-76]	73% [71-76]

's

Supplementary Table 2: Correlations of CINSARC classes with clinicopathological characteristics in Luminal A breast cancer patients

Characteristics		CINSARC classes		
	N	Low-risk	High-risk	p-value
Patients' age				0.211
≤ 50	401	360 (29%)	41 (35%)	
> 50	967	890 (71%)	77 (65%)	
Pathological tumor type				0.938
ductal	700	642 (69%)	58 (67%)	
lobular	164	149 (16%)	15 (17%)	
other	155	142 (15%)	13 (15%)	
Pathological grade				**7.60E-05**
1	335	317 (31%)	18 (17%)	
2	604	550 (53%)	54 (51%)	
3	206	172 (17%)	34 (32%)	
Pathological axillary lymph node status				0.305
negative	919	846 (62%)	73 (57%)	
positive	583	527 (38%)	56 (43%)	
Pathological tumor size				**4.01E-02**
pT1	682	636 (50%)	46 (38%)	
pT2	602	538 (43%)	64 (53%)	
pT3	97	87 (7%)	10 (8%)	
Adjuvant chemotherapy				0.665
no	1049	958 (82%)	91 (84%)	
yes	226	209 (18%)	17 (16%)	
Adjuvant hormone therapy				0.687
no	647	590 (51%)	57 (54%)	
yes	611	562 (49%)	49 (46%)	
Follow-up median, months (r	1753	83 (1-382)	87 (1-281)	**1.48E-03**
DFS event (%)	1753	305 (19%)	46 (29%)	**6.92E-03**
5-year DFS	1753	88% [86-89]	78% [72-86]	**3.31E-02**

Supplementary Table 3: Univariate and multivariate Cox regression analyses for DFS in Luminal A breast cancer

All patients		Univariate			Multivariate		
		N	HR [95%CI]	p-value	N	HR [95%CI]	p-value
Patients' age	> 50 vs. ≤ 50	1368	1.30 [0.96-1.77]	**9.34E-02**	934	1.13 [0.80-1.58]	0.490
Pathological tumor type	lobular vs. ductal	1019	1.32 [0.84-2.09]	0.158			
	other vs. ductal		0.73 [0.45-1.18]				
Pathological grade	2 vs. 1	1145	1.08 [0.77-1.50]	**1.61E-02**	934	1 [0.68-1.45]	0.979
	3 vs. 1		1.66 [1.13-2.43]		934	1.32 [0.85-2.04]	0.213
Pathological axillary lymph node status	positive vs. negative	1502	1.41 [1.09-1.82]	**9.18E-03**	934	1.09 [0.79-1.51]	0.587
Pathological tumor size	pT2 vs. pT1	1381	1.52 [1.16-2.01]	**1.19E-03**	934	1.54 [1.12-2.13]	**8.66E-03**
	pT3 vs. pT1		2.25 [1.28-3.96]		934	2.02 [0.96-4.25]	0.064
Adjuvant chemotherapy	yes vs. no	1275	0.82 [0.50-1.36]	0.443			
Adjuvant hormone therapy	yes vs. no	1258	1.00 [0.76-1.33]	0.978			
CINSARC classes	High-risk vs. Low-risk	1753	1.40 [1.03-1.91]	**3.40E-02**	934	1.21 [0.77-1.91]	0.398

Patients with adjuvant HT and without adjuvant CT		Univariate			Multivariate		
		N	HR [95%CI]	p-value	N	HR [95%CI]	p-value
Patients' age	> 50 vs. ≤ 50	493	1.13 [0.52-2.48]	0.752			
Pathological tumor type	lobular vs. ductal	458	1.23 [0.58-2.59]	0.479			
	other vs. ductal		0.66 [0.30-1.47]				
Pathological grade	2 vs. 1	353	1.35 [0.64-2.85]	0.117			
	3 vs. 1		2.10 [0.97-4.58]				
Pathological axillary lymph node status	positive vs. negative	515	1.41 [0.88-2.26]	0.155			
Pathological tumor size	pT2 vs. pT1	492	1.77 [1.07-2.91]	**1.47E-02**			
	pT3 vs. pT1		3.38 [1.29-8.83]				
CINSARC classes	High-risk vs. Low-risk	527	0.82 [0.36-1.90]	0.648			

Supplementary Table 4: Comparison of frequency of mutations among the 387 genes mutated in at least 5/270 tested samples (TCGA) between the two CINSARC classes in Luminal B breast cancers.

Symbol	CINSARC Low			CINSARC High			CINSARC High vs. Low		
	N tested	N altered	Fq alteration	N tested	N altered	Fq alteration	OR [95%CI]	p-value*	q-value
PIK3CA	93	34	36.56%	177	51	28.81%	0.70 [0.45-1.10]	0.194	0.895
TTN	93	14	15.05%	177	37	20.90%	1.49 [0.85-2.63]	0.245	0.895
TP53	93	11	11.83%	177	35	19.77%	1.84 [1.00-3.39]	0.102	0.895
GATA3	93	16	17.20%	177	32	18.08%	1.06 [0.61-1.85]	0.858	0.962
CDH1	93	6	6.45%	177	14	7.91%	1.25 [0.54-2.86]	0.664	0.910
CROCCP2	93	4	4.30%	177	14	7.91%	1.91 [0.73-4.98]	0.266	0.895
MUC16	93	4	4.30%	177	14	7.91%	1.91 [0.73-4.98]	0.266	0.895
MAP3K1	93	10	10.75%	177	12	6.78%	0.60 [0.29-1.26]	0.261	0.895
MLL3	93	7	7.53%	177	12	6.78%	0.89 [0.40-2.01]	0.82	0.939
MUC5B	93	5	5.38%	177	11	6.21%	1.17 [0.47-2.91]	0.782	0.910
HMCN1	93	4	4.30%	177	10	5.65%	1.33 [0.49-3.61]	0.636	0.910
FRG1B	93	2	2.15%	177	10	5.65%	2.72 [0.75-9.92]	0.202	0.895
DYNC2H1	93	2	2.15%	177	9	5.08%	2.44 [0.66-8.98]	0.261	0.895
HRNR	93	2	2.15%	177	9	5.08%	2.44 [0.66-8.98]	0.261	0.895
MUC12	93	5	5.38%	177	8	4.52%	0.83 [0.32-2.18]	0.755	0.910
NEB	93	5	5.38%	177	8	4.52%	0.83 [0.32-2.18]	0.755	0.910
SYNE2	93	5	5.38%	177	8	4.52%	0.83 [0.32-2.18]	0.755	0.910
MUC4	93	4	4.30%	177	8	4.52%	1.05 [0.38-2.95]	0.934	0.974
PTEN	93	4	4.30%	177	8	4.52%	1.05 [0.38-2.95]	0.934	0.974
ZFHX4	93	4	4.30%	177	8	4.52%	1.05 [0.38-2.95]	0.934	0.974
HUWE1	93	2	2.15%	177	8	4.52%	2.15 [0.58-8.05]	0.338	0.895
RELN	93	1	1.08%	177	8	4.52%	4.36 [0.75-25.25]	0.169	0.895
DMD	93	5	5.38%	177	7	3.95%	0.72 [0.27-1.94]	0.592	0.910
FLG	93	5	5.38%	177	7	3.95%	0.72 [0.27-1.94]	0.592	0.910
MAP2K4	93	5	5.38%	177	7	3.95%	0.72 [0.27-1.94]	0.592	0.910
RYR3	93	4	4.30%	177	7	3.95%	0.92 [0.32-2.63]	0.891	0.974
CDK12	93	1	1.08%	177	7	3.95%	3.79 [0.64-22.27]	0.216	0.895
MYH11	93	1	1.08%	177	7	3.95%	3.79 [0.64-22.27]	0.216	0.895
NCOR1	93	7	7.53%	177	6	3.39%	0.43 [0.17-1.10]	0.141	0.895
OBSCN	93	7	7.53%	177	6	3.39%	0.43 [0.17-1.10]	0.141	0.895
PCLO	93	6	6.45%	177	6	3.39%	0.51 [0.19-1.35]	0.254	0.895
SPTA1	93	5	5.38%	177	6	3.39%	0.62 [0.22-1.71]	0.437	0.895
FAM157B	93	4	4.30%	177	6	3.39%	0.78 [0.26-2.31]	0.707	0.910
FAT3	93	4	4.30%	177	6	3.39%	0.78 [0.26-2.31]	0.707	0.910
SPEN	93	4	4.30%	177	6	3.39%	0.78 [0.26-2.31]	0.707	0.910
USH2A	93	4	4.30%	177	6	3.39%	0.78 [0.26-2.31]	0.707	0.910
CHD6	93	3	3.23%	177	6	3.39%	1.05 [0.32-3.43]	0.943	0.974
PRUNE2	93	3	3.23%	177	6	3.39%	1.05 [0.32-3.43]	0.943	0.974
CADPS	93	2	2.15%	177	6	3.39%	1.60 [0.41-6.22]	0.571	0.910
DNAH7	93	2	2.15%	177	6	3.39%	1.60 [0.41-6.22]	0.571	0.910
FAT2	93	2	2.15%	177	6	3.39%	1.60 [0.41-6.22]	0.571	0.910
LRP2	93	2	2.15%	177	6	3.39%	1.60 [0.41-6.22]	0.571	0.910
RYR2	93	2	2.15%	177	6	3.39%	1.60 [0.41-6.22]	0.571	0.910
TBX3	93	2	2.15%	177	6	3.39%	1.60 [0.41-6.22]	0.571	0.910
DNHD1	93	1	1.08%	177	6	3.39%	3.23 [0.54-19.32]	0.281	0.895
DNM1P46	93	1	1.08%	177	6	3.39%	3.23 [0.54-19.32]	0.281	0.895
HIST1H3B	93	1	1.08%	177	6	3.39%	3.23 [0.54-19.32]	0.281	0.895
KALRN	93	1	1.08%	177	6	3.39%	3.23 [0.54-19.32]	0.281	0.895
NRXN2	93	1	1.08%	177	6	3.39%	3.23 [0.54-19.32]	0.281	0.895
SETDB1	93	1	1.08%	177	6	3.39%	3.23 [0.54-19.32]	0.281	0.895
XIST	93	1	1.08%	177	6	3.39%	3.23 [0.54-19.32]	0.281	0.895
FAM47C	93	0	0.00%	177	6	3.39%	3e+07 [0e+00-Inf]	0.993	0.993
APOB	93	6	6.45%	177	5	2.82%	0.42 [0.15-1.17]	0.163	0.895
DST	93	5	5.38%	177	5	2.82%	0.51 [0.18-1.48]	0.299	0.895
MACF1	93	5	5.38%	177	5	2.82%	0.51 [0.18-1.48]	0.299	0.895
PTPRD	93	5	5.38%	177	5	2.82%	0.51 [0.18-1.48]	0.299	0.895
FRMPD4	93	4	4.30%	177	5	2.82%	0.65 [0.21-1.99]	0.524	0.910
LRBA	93	4	4.30%	177	5	2.82%	0.65 [0.21-1.99]	0.524	0.910
MAP2	93	4	4.30%	177	5	2.82%	0.65 [0.21-1.99]	0.524	0.910
PCNXL2	93	4	4.30%	177	5	2.82%	0.65 [0.21-1.99]	0.524	0.910
ANK3	93	3	3.23%	177	5	2.82%	0.87 [0.26-2.95]	0.854	0.961
ERBB2	93	3	3.23%	177	5	2.82%	0.87 [0.26-2.95]	0.854	0.961
FBN3	93	3	3.23%	177	5	2.82%	0.87 [0.26-2.95]	0.854	0.961
LRP1	93	3	3.23%	177	5	2.82%	0.87 [0.26-2.95]	0.854	0.961
RAPGEF6	93	3	3.23%	177	5	2.82%	0.87 [0.26-2.95]	0.854	0.961
SYNE1	93	3	3.23%	177	5	2.82%	0.87 [0.26-2.95]	0.854	0.961
ADAMTSL1	93	2	2.15%	177	5	2.82%	1.32 [0.33-5.32]	0.741	0.910
CMYA5	93	2	2.15%	177	5	2.82%	1.32 [0.33-5.32]	0.741	0.910
CSMD2	93	2	2.15%	177	5	2.82%	1.32 [0.33-5.32]	0.741	0.910
CUBN	93	2	2.15%	177	5	2.82%	1.32 [0.33-5.32]	0.741	0.910
DNAH11	93	2	2.15%	177	5	2.82%	1.32 [0.33-5.32]	0.741	0.910
FREM3	93	2	2.15%	177	5	2.82%	1.32 [0.33-5.32]	0.741	0.910
HECTD4	93	2	2.15%	177	5	2.82%	1.32 [0.33-5.32]	0.741	0.910

Symbol	CINSARC Low			CINSARC High			CINSARC High vs. Low		
	N tested	N altered	Fq alteration	N tested	N altered	Fq alteration	OR [95%CI]	p-value*	q-value
KIAA1109	93	2	2.15%	177	5	2.82%	1.32 [0.33-5.32]	0.741	0.910
MST1P9	93	2	2.15%	177	5	2.82%	1.32 [0.33-5.32]	0.741	0.910
MTOR	93	2	2.15%	177	5	2.82%	1.32 [0.33-5.32]	0.741	0.910
NF1	93	2	2.15%	177	5	2.82%	1.32 [0.33-5.32]	0.741	0.910
SDHAP1	93	2	2.15%	177	5	2.82%	1.32 [0.33-5.32]	0.741	0.910
SEMA5A	93	2	2.15%	177	5	2.82%	1.32 [0.33-5.32]	0.741	0.910
TPR	93	2	2.15%	177	5	2.82%	1.32 [0.33-5.32]	0.741	0.910
ZAN	93	2	2.15%	177	5	2.82%	1.32 [0.33-5.32]	0.741	0.910
ASH1L	93	1	1.08%	177	5	2.82%	2.67 [0.44-16.41]	0.372	0.895
C5orf42	93	1	1.08%	177	5	2.82%	2.67 [0.44-16.41]	0.372	0.895
COL4A4	93	1	1.08%	177	5	2.82%	2.67 [0.44-16.41]	0.372	0.895
CRNKL1	93	1	1.08%	177	5	2.82%	2.67 [0.44-16.41]	0.372	0.895
ERCC6	93	1	1.08%	177	5	2.82%	2.67 [0.44-16.41]	0.372	0.895
HECW1	93	1	1.08%	177	5	2.82%	2.67 [0.44-16.41]	0.372	0.895
KIAA2022	93	1	1.08%	177	5	2.82%	2.67 [0.44-16.41]	0.372	0.895
LARP1	93	1	1.08%	177	5	2.82%	2.67 [0.44-16.41]	0.372	0.895
MYH8	93	1	1.08%	177	5	2.82%	2.67 [0.44-16.41]	0.372	0.895
MYO6	93	1	1.08%	177	5	2.82%	2.67 [0.44-16.41]	0.372	0.895
NES	93	1	1.08%	177	5	2.82%	2.67 [0.44-16.41]	0.372	0.895
PAPPA	93	1	1.08%	177	5	2.82%	2.67 [0.44-16.41]	0.372	0.895
PLCE1	93	1	1.08%	177	5	2.82%	2.67 [0.44-16.41]	0.372	0.895
SLX4	93	1	1.08%	177	5	2.82%	2.67 [0.44-16.41]	0.372	0.895
TAF1L	93	1	1.08%	177	5	2.82%	2.67 [0.44-16.41]	0.372	0.895
ZFP64	93	1	1.08%	177	5	2.82%	2.67 [0.44-16.41]	0.372	0.895
ABCC8	93	0	0.00%	177	5	2.82%	2.5e+07 [0e+00-Inf]	0.993	0.993
ACTN4	93	0	0.00%	177	5	2.82%	2.5e+07 [0e+00-Inf]	0.993	0.993
CNOT3	93	0	0.00%	177	5	2.82%	2.5e+07 [0e+00-Inf]	0.993	0.993
COL6A3	93	0	0.00%	177	5	2.82%	2.5e+07 [0e+00-Inf]	0.993	0.993
HSPG2	93	0	0.00%	177	5	2.82%	2.5e+07 [0e+00-Inf]	0.993	0.993
TBC1D8B	93	0	0.00%	177	5	2.82%	2.5e+07 [0e+00-Inf]	0.993	0.993
ZNF804B	93	0	0.00%	177	5	2.82%	2.5e+07 [0e+00-Inf]	0.993	0.993
CSMD1	93	5	5.38%	177	4	2.26%	0.41 [0.13-1.25]	0.188	0.895
FCGBP	93	5	5.38%	177	4	2.26%	0.41 [0.13-1.25]	0.188	0.895
GPR98	93	5	5.38%	177	4	2.26%	0.41 [0.13-1.25]	0.188	0.895
C9orf174	93	4	4.30%	177	4	2.26%	0.51 [0.16-1.68]	0.355	0.895
DYNC1H1	93	4	4.30%	177	4	2.26%	0.51 [0.16-1.68]	0.355	0.895
GNAS	93	4	4.30%	177	4	2.26%	0.51 [0.16-1.68]	0.355	0.895
MADD	93	4	4.30%	177	4	2.26%	0.51 [0.16-1.68]	0.355	0.895
MYH7	93	4	4.30%	177	4	2.26%	0.51 [0.16-1.68]	0.355	0.895
RP1	93	4	4.30%	177	4	2.26%	0.51 [0.16-1.68]	0.355	0.895
RYR1	93	4	4.30%	177	4	2.26%	0.51 [0.16-1.68]	0.355	0.895
VCAN	93	4	4.30%	177	4	2.26%	0.51 [0.16-1.68]	0.355	0.895
AKAP9	93	3	3.23%	177	4	2.26%	0.69 [0.19-2.48]	0.637	0.910
ATM	93	3	3.23%	177	4	2.26%	0.69 [0.19-2.48]	0.637	0.910
CIT	93	3	3.23%	177	4	2.26%	0.69 [0.19-2.48]	0.637	0.910
CTCF	93	3	3.23%	177	4	2.26%	0.69 [0.19-2.48]	0.637	0.910
DNAH10	93	3	3.23%	177	4	2.26%	0.69 [0.19-2.48]	0.637	0.910
DNAH9	93	3	3.23%	177	4	2.26%	0.69 [0.19-2.48]	0.637	0.910
FAT1	93	3	3.23%	177	4	2.26%	0.69 [0.19-2.48]	0.637	0.910
FLNB	93	3	3.23%	177	4	2.26%	0.69 [0.19-2.48]	0.637	0.910
GOLGA6L2	93	3	3.23%	177	4	2.26%	0.69 [0.19-2.48]	0.637	0.910
HERC2	93	3	3.23%	177	4	2.26%	0.69 [0.19-2.48]	0.637	0.910
NRXN1	93	3	3.23%	177	4	2.26%	0.69 [0.19-2.48]	0.637	0.910
PAPPA2	93	3	3.23%	177	4	2.26%	0.69 [0.19-2.48]	0.637	0.910
PLXNA4	93	3	3.23%	177	4	2.26%	0.69 [0.19-2.48]	0.637	0.910
RUNX1	93	3	3.23%	177	4	2.26%	0.69 [0.19-2.48]	0.637	0.910
SCN10A	93	3	3.23%	177	4	2.26%	0.69 [0.19-2.48]	0.637	0.910
TMEM132D	93	3	3.23%	177	4	2.26%	0.69 [0.19-2.48]	0.637	0.910
ABR	93	2	2.15%	177	4	2.26%	1.05 [0.25-4.44]	0.954	0.974
ANKRD30BL	93	2	2.15%	177	4	2.26%	1.05 [0.25-4.44]	0.954	0.974
APOBR	93	2	2.15%	177	4	2.26%	1.05 [0.25-4.44]	0.954	0.974
ATRX	93	2	2.15%	177	4	2.26%	1.05 [0.25-4.44]	0.954	0.974
C1orf173	93	2	2.15%	177	4	2.26%	1.05 [0.25-4.44]	0.954	0.974
CENPE	93	2	2.15%	177	4	2.26%	1.05 [0.25-4.44]	0.954	0.974
CHD8	93	2	2.15%	177	4	2.26%	1.05 [0.25-4.44]	0.954	0.974
COL12A1	93	2	2.15%	177	4	2.26%	1.05 [0.25-4.44]	0.954	0.974
CSMD3	93	2	2.15%	177	4	2.26%	1.05 [0.25-4.44]	0.954	0.974
DNAH17	93	2	2.15%	177	4	2.26%	1.05 [0.25-4.44]	0.954	0.974
EPG5	93	2	2.15%	177	4	2.26%	1.05 [0.25-4.44]	0.954	0.974
HEATR7B2	93	2	2.15%	177	4	2.26%	1.05 [0.25-4.44]	0.954	0.974
LRP4	93	2	2.15%	177	4	2.26%	1.05 [0.25-4.44]	0.954	0.974
MALAT1	93	2	2.15%	177	4	2.26%	1.05 [0.25-4.44]	0.954	0.974
MICAL3	93	2	2.15%	177	4	2.26%	1.05 [0.25-4.44]	0.954	0.974
MYO5B	93	2	2.15%	177	4	2.26%	1.05 [0.25-4.44]	0.954	0.974
NUP98	93	2	2.15%	177	4	2.26%	1.05 [0.25-4.44]	0.954	0.974
PARP4	93	2	2.15%	177	4	2.26%	1.05 [0.25-4.44]	0.954	0.974

Symbol	CINSARC Low			CINSARC High			CINSARC High vs. Low		
	N tested	N altered	Fq alteration	N tested	N altered	Fq alteration	OR [95%CI]	p-value*	q-value
PCDH19	93	2	2.15%	177	4	2.26%	1.05 [0.25-4.44]	0.954	0.974
PIK3R1	93	2	2.15%	177	4	2.26%	1.05 [0.25-4.44]	0.954	0.974
PIWIL1	93	2	2.15%	177	4	2.26%	1.05 [0.25-4.44]	0.954	0.974
PTPRB	93	2	2.15%	177	4	2.26%	1.05 [0.25-4.44]	0.954	0.974
SHROOM2	93	2	2.15%	177	4	2.26%	1.05 [0.25-4.44]	0.954	0.974
SRCAP	93	2	2.15%	177	4	2.26%	1.05 [0.25-4.44]	0.954	0.974
TG	93	2	2.15%	177	4	2.26%	1.05 [0.25-4.44]	0.954	0.974
VPS13B	93	2	2.15%	177	4	2.26%	1.05 [0.25-4.44]	0.954	0.974
ZNF814	93	2	2.15%	177	4	2.26%	1.05 [0.25-4.44]	0.954	0.974
ZNF831	93	2	2.15%	177	4	2.26%	1.05 [0.25-4.44]	0.954	0.974
ADAMTS20	93	1	1.08%	177	4	2.26%	2.13 [0.33-13.54]	0.502	0.895
ATP2A1	93	1	1.08%	177	4	2.26%	2.13 [0.33-13.54]	0.502	0.895
CACNA1A	93	1	1.08%	177	4	2.26%	2.13 [0.33-13.54]	0.502	0.895
CACNA1C	93	1	1.08%	177	4	2.26%	2.13 [0.33-13.54]	0.502	0.895
CDH12	93	1	1.08%	177	4	2.26%	2.13 [0.33-13.54]	0.502	0.895
CEP192	93	1	1.08%	177	4	2.26%	2.13 [0.33-13.54]	0.502	0.895
CNTN6	93	1	1.08%	177	4	2.26%	2.13 [0.33-13.54]	0.502	0.895
COL19A1	93	1	1.08%	177	4	2.26%	2.13 [0.33-13.54]	0.502	0.895
COL4A6	93	1	1.08%	177	4	2.26%	2.13 [0.33-13.54]	0.502	0.895
COL5A1	93	1	1.08%	177	4	2.26%	2.13 [0.33-13.54]	0.502	0.895
CR1	93	1	1.08%	177	4	2.26%	2.13 [0.33-13.54]	0.502	0.895
CRIPAK	93	1	1.08%	177	4	2.26%	2.13 [0.33-13.54]	0.502	0.895
DHX57	93	1	1.08%	177	4	2.26%	2.13 [0.33-13.54]	0.502	0.895
DNAH2	93	1	1.08%	177	4	2.26%	2.13 [0.33-13.54]	0.502	0.895
DNAH5	93	1	1.08%	177	4	2.26%	2.13 [0.33-13.54]	0.502	0.895
ERBB4	93	1	1.08%	177	4	2.26%	2.13 [0.33-13.54]	0.502	0.895
EYS	93	1	1.08%	177	4	2.26%	2.13 [0.33-13.54]	0.502	0.895
F5	93	1	1.08%	177	4	2.26%	2.13 [0.33-13.54]	0.502	0.895
FAM186A	93	1	1.08%	177	4	2.26%	2.13 [0.33-13.54]	0.502	0.895
FAT4	93	1	1.08%	177	4	2.26%	2.13 [0.33-13.54]	0.502	0.895
FGFR2	93	1	1.08%	177	4	2.26%	2.13 [0.33-13.54]	0.502	0.895
FNDC1	93	1	1.08%	177	4	2.26%	2.13 [0.33-13.54]	0.502	0.895
HECTD1	93	1	1.08%	177	4	2.26%	2.13 [0.33-13.54]	0.502	0.895
HS6ST1	93	1	1.08%	177	4	2.26%	2.13 [0.33-13.54]	0.502	0.895
KIAA0232	93	1	1.08%	177	4	2.26%	2.13 [0.33-13.54]	0.502	0.895
KIAA1210	93	1	1.08%	177	4	2.26%	2.13 [0.33-13.54]	0.502	0.895
KIF1B	93	1	1.08%	177	4	2.26%	2.13 [0.33-13.54]	0.502	0.895
LETM1	93	1	1.08%	177	4	2.26%	2.13 [0.33-13.54]	0.502	0.895
MAPRE3	93	1	1.08%	177	4	2.26%	2.13 [0.33-13.54]	0.502	0.895
MAST4	93	1	1.08%	177	4	2.26%	2.13 [0.33-13.54]	0.502	0.895
MED14	93	1	1.08%	177	4	2.26%	2.13 [0.33-13.54]	0.502	0.895
MYADML	93	1	1.08%	177	4	2.26%	2.13 [0.33-13.54]	0.502	0.895
OBSL1	93	1	1.08%	177	4	2.26%	2.13 [0.33-13.54]	0.502	0.895
PCDHA11	93	1	1.08%	177	4	2.26%	2.13 [0.33-13.54]	0.502	0.895
PRKCE	93	1	1.08%	177	4	2.26%	2.13 [0.33-13.54]	0.502	0.895
REV3L	93	1	1.08%	177	4	2.26%	2.13 [0.33-13.54]	0.502	0.895
RPS6KA1	93	1	1.08%	177	4	2.26%	2.13 [0.33-13.54]	0.502	0.895
SCN1A	93	1	1.08%	177	4	2.26%	2.13 [0.33-13.54]	0.502	0.895
SELP	93	1	1.08%	177	4	2.26%	2.13 [0.33-13.54]	0.502	0.895
SMARCC2	93	1	1.08%	177	4	2.26%	2.13 [0.33-13.54]	0.502	0.895
SOGA1	93	1	1.08%	177	4	2.26%	2.13 [0.33-13.54]	0.502	0.895
UBE4A	93	1	1.08%	177	4	2.26%	2.13 [0.33-13.54]	0.502	0.895
VCPIP1	93	1	1.08%	177	4	2.26%	2.13 [0.33-13.54]	0.502	0.895
VWDE	93	1	1.08%	177	4	2.26%	2.13 [0.33-13.54]	0.502	0.895
ZFYVE19	93	1	1.08%	177	4	2.26%	2.13 [0.33-13.54]	0.502	0.895
ZNF292	93	1	1.08%	177	4	2.26%	2.13 [0.33-13.54]	0.502	0.895
ATP10B	93	5	5.38%	177	3	1.69%	0.30 [0.09-1.03]	0.108	0.895
PKHD1L1	93	5	5.38%	177	3	1.69%	0.30 [0.09-1.03]	0.108	0.895
SACS	93	5	5.38%	177	3	1.69%	0.30 [0.09-1.03]	0.108	0.895
ANK2	93	4	4.30%	177	3	1.69%	0.38 [0.11-1.37]	0.216	0.895
ANKRD12	93	4	4.30%	177	3	1.69%	0.38 [0.11-1.37]	0.216	0.895
CCDC144A	93	4	4.30%	177	3	1.69%	0.38 [0.11-1.37]	0.216	0.895
ODZ2	93	4	4.30%	177	3	1.69%	0.38 [0.11-1.37]	0.216	0.895
RB1	93	4	4.30%	177	3	1.69%	0.38 [0.11-1.37]	0.216	0.895
TANC2	93	4	4.30%	177	3	1.69%	0.38 [0.11-1.37]	0.216	0.895
TNRC18	93	4	4.30%	177	3	1.69%	0.38 [0.11-1.37]	0.216	0.895
UBR4	93	4	4.30%	177	3	1.69%	0.38 [0.11-1.37]	0.216	0.895
AKD1	93	3	3.23%	177	3	1.69%	0.52 [0.13-2.01]	0.425	0.895
ARID1A	93	3	3.23%	177	3	1.69%	0.52 [0.13-2.01]	0.425	0.895
CDC42BPA	93	3	3.23%	177	3	1.69%	0.52 [0.13-2.01]	0.425	0.895
CELSR3	93	3	3.23%	177	3	1.69%	0.52 [0.13-2.01]	0.425	0.895
DNAH12	93	3	3.23%	177	3	1.69%	0.52 [0.13-2.01]	0.425	0.895
DOCK11	93	3	3.23%	177	3	1.69%	0.52 [0.13-2.01]	0.425	0.895
F8	93	3	3.23%	177	3	1.69%	0.52 [0.13-2.01]	0.425	0.895
FAM47B	93	3	3.23%	177	3	1.69%	0.52 [0.13-2.01]	0.425	0.895
FMN2	93	3	3.23%	177	3	1.69%	0.52 [0.13-2.01]	0.425	0.895

Symbol	CINSARC Low			CINSARC High			CINSARC High vs. Low		
	N tested	N altered	Fq alteration	N tested	N altered	Fq alteration	OR [95%CI]	p-value*	q-value
GON4L	93	3	3.23%	177	3	1.69%	0.52 [0.13-2.01]	0.425	0.895
HEPH	93	3	3.23%	177	3	1.69%	0.52 [0.13-2.01]	0.425	0.895
HERC1	93	3	3.23%	177	3	1.69%	0.52 [0.13-2.01]	0.425	0.895
ITPR1	93	3	3.23%	177	3	1.69%	0.52 [0.13-2.01]	0.425	0.895
MDN1	93	3	3.23%	177	3	1.69%	0.52 [0.13-2.01]	0.425	0.895
MED12	93	3	3.23%	177	3	1.69%	0.52 [0.13-2.01]	0.425	0.895
MLL	93	3	3.23%	177	3	1.69%	0.52 [0.13-2.01]	0.425	0.895
MYOF	93	3	3.23%	177	3	1.69%	0.52 [0.13-2.01]	0.425	0.895
ODZ4	93	3	3.23%	177	3	1.69%	0.52 [0.13-2.01]	0.425	0.895
PDZD2	93	3	3.23%	177	3	1.69%	0.52 [0.13-2.01]	0.425	0.895
PHF3	93	3	3.23%	177	3	1.69%	0.52 [0.13-2.01]	0.425	0.895
PKHD1	93	3	3.23%	177	3	1.69%	0.52 [0.13-2.01]	0.425	0.895
PLCH1	93	3	3.23%	177	3	1.69%	0.52 [0.13-2.01]	0.425	0.895
POM121	93	3	3.23%	177	3	1.69%	0.52 [0.13-2.01]	0.425	0.895
RPGR	93	3	3.23%	177	3	1.69%	0.52 [0.13-2.01]	0.425	0.895
SCN8A	93	3	3.23%	177	3	1.69%	0.52 [0.13-2.01]	0.425	0.895
SH3PXD2A	93	3	3.23%	177	3	1.69%	0.52 [0.13-2.01]	0.425	0.895
SPHKAP	93	3	3.23%	177	3	1.69%	0.52 [0.13-2.01]	0.425	0.895
TBC1D3P5	93	3	3.23%	177	3	1.69%	0.52 [0.13-2.01]	0.425	0.895
TLR7	93	3	3.23%	177	3	1.69%	0.52 [0.13-2.01]	0.425	0.895
TNS1	93	3	3.23%	177	3	1.69%	0.52 [0.13-2.01]	0.425	0.895
ZCCHC6	93	3	3.23%	177	3	1.69%	0.52 [0.13-2.01]	0.425	0.895
ABCA9	93	2	2.15%	177	3	1.69%	0.78 [0.17-3.57]	0.792	0.910
ABCB11	93	2	2.15%	177	3	1.69%	0.78 [0.17-3.57]	0.792	0.910
ADCY7	93	2	2.15%	177	3	1.69%	0.78 [0.17-3.57]	0.792	0.910
AHNAK2	93	2	2.15%	177	3	1.69%	0.78 [0.17-3.57]	0.792	0.910
BRWD3	93	2	2.15%	177	3	1.69%	0.78 [0.17-3.57]	0.792	0.910
CACNA1D	93	2	2.15%	177	3	1.69%	0.78 [0.17-3.57]	0.792	0.910
CCAR1	93	2	2.15%	177	3	1.69%	0.78 [0.17-3.57]	0.792	0.910
CDH23	93	2	2.15%	177	3	1.69%	0.78 [0.17-3.57]	0.792	0.910
CFH	93	2	2.15%	177	3	1.69%	0.78 [0.17-3.57]	0.792	0.910
CLEC16A	93	2	2.15%	177	3	1.69%	0.78 [0.17-3.57]	0.792	0.910
CNTLN	93	2	2.15%	177	3	1.69%	0.78 [0.17-3.57]	0.792	0.910
COL6A5	93	2	2.15%	177	3	1.69%	0.78 [0.17-3.57]	0.792	0.910
DCHS1	93	2	2.15%	177	3	1.69%	0.78 [0.17-3.57]	0.792	0.910
DENND1B	93	2	2.15%	177	3	1.69%	0.78 [0.17-3.57]	0.792	0.910
EFCAB5	93	2	2.15%	177	3	1.69%	0.78 [0.17-3.57]	0.792	0.910
FANCD2	93	2	2.15%	177	3	1.69%	0.78 [0.17-3.57]	0.792	0.910
FBXW7	93	2	2.15%	177	3	1.69%	0.78 [0.17-3.57]	0.792	0.910
FREM2	93	2	2.15%	177	3	1.69%	0.78 [0.17-3.57]	0.792	0.910
FRMD4A	93	2	2.15%	177	3	1.69%	0.78 [0.17-3.57]	0.792	0.910
FRMPD2	93	2	2.15%	177	3	1.69%	0.78 [0.17-3.57]	0.792	0.910
GLYR1	93	2	2.15%	177	3	1.69%	0.78 [0.17-3.57]	0.792	0.910
GRHL2	93	2	2.15%	177	3	1.69%	0.78 [0.17-3.57]	0.792	0.910
GUCY2F	93	2	2.15%	177	3	1.69%	0.78 [0.17-3.57]	0.792	0.910
INADL	93	2	2.15%	177	3	1.69%	0.78 [0.17-3.57]	0.792	0.910
INPPL1	93	2	2.15%	177	3	1.69%	0.78 [0.17-3.57]	0.792	0.910
KCNA1	93	2	2.15%	177	3	1.69%	0.78 [0.17-3.57]	0.792	0.910
KCNT2	93	2	2.15%	177	3	1.69%	0.78 [0.17-3.57]	0.792	0.910
KDM2A	93	2	2.15%	177	3	1.69%	0.78 [0.17-3.57]	0.792	0.910
KIF1A	93	2	2.15%	177	3	1.69%	0.78 [0.17-3.57]	0.792	0.910
KSR1	93	2	2.15%	177	3	1.69%	0.78 [0.17-3.57]	0.792	0.910
LAMB1	93	2	2.15%	177	3	1.69%	0.78 [0.17-3.57]	0.792	0.910
LRIG2	93	2	2.15%	177	3	1.69%	0.78 [0.17-3.57]	0.792	0.910
LRPPRC	93	2	2.15%	177	3	1.69%	0.78 [0.17-3.57]	0.792	0.910
MCM3AP	93	2	2.15%	177	3	1.69%	0.78 [0.17-3.57]	0.792	0.910
MLL2	93	2	2.15%	177	3	1.69%	0.78 [0.17-3.57]	0.792	0.910
MLLT10	93	2	2.15%	177	3	1.69%	0.78 [0.17-3.57]	0.792	0.910
MUC17	93	2	2.15%	177	3	1.69%	0.78 [0.17-3.57]	0.792	0.910
MYO3A	93	2	2.15%	177	3	1.69%	0.78 [0.17-3.57]	0.792	0.910
NAALADL2	93	2	2.15%	177	3	1.69%	0.78 [0.17-3.57]	0.792	0.910
NBEAL1	93	2	2.15%	177	3	1.69%	0.78 [0.17-3.57]	0.792	0.910
NIN	93	2	2.15%	177	3	1.69%	0.78 [0.17-3.57]	0.792	0.910
NLRP9	93	2	2.15%	177	3	1.69%	0.78 [0.17-3.57]	0.792	0.910
NPAS4	93	2	2.15%	177	3	1.69%	0.78 [0.17-3.57]	0.792	0.910
NYNRIN	93	2	2.15%	177	3	1.69%	0.78 [0.17-3.57]	0.792	0.910
OCRL	93	2	2.15%	177	3	1.69%	0.78 [0.17-3.57]	0.792	0.910
OTOF	93	2	2.15%	177	3	1.69%	0.78 [0.17-3.57]	0.792	0.910
PCDHA8	93	2	2.15%	177	3	1.69%	0.78 [0.17-3.57]	0.792	0.910
PLCL2	93	2	2.15%	177	3	1.69%	0.78 [0.17-3.57]	0.792	0.910
PLCZ1	93	2	2.15%	177	3	1.69%	0.78 [0.17-3.57]	0.792	0.910
PREX2	93	2	2.15%	177	3	1.69%	0.78 [0.17-3.57]	0.792	0.910
PRKCQ	93	2	2.15%	177	3	1.69%	0.78 [0.17-3.57]	0.792	0.910
PRPF8	93	2	2.15%	177	3	1.69%	0.78 [0.17-3.57]	0.792	0.910
PTPN22	93	2	2.15%	177	3	1.69%	0.78 [0.17-3.57]	0.792	0.910
SCN11A	93	2	2.15%	177	3	1.69%	0.78 [0.17-3.57]	0.792	0.910

Symbol	CINSARC Low			CINSARC High			CINSARC High vs. Low		
	N tested	N altered	Fq alteration	N tested	N altered	Fq alteration	OR [95%CI]	p-value*	q-value
SCN3A	93	2	2.15%	177	3	1.69%	0.78 [0.17-3.57]	0.792	0.910
SDK1	93	2	2.15%	177	3	1.69%	0.78 [0.17-3.57]	0.792	0.910
SHANK2	93	2	2.15%	177	3	1.69%	0.78 [0.17-3.57]	0.792	0.910
SIPA1L3	93	2	2.15%	177	3	1.69%	0.78 [0.17-3.57]	0.792	0.910
SLCO1B3	93	2	2.15%	177	3	1.69%	0.78 [0.17-3.57]	0.792	0.910
STARD8	93	2	2.15%	177	3	1.69%	0.78 [0.17-3.57]	0.792	0.910
SVEP1	93	2	2.15%	177	3	1.69%	0.78 [0.17-3.57]	0.792	0.910
TBC1D4	93	2	2.15%	177	3	1.69%	0.78 [0.17-3.57]	0.792	0.910
TNKS1BP1	93	2	2.15%	177	3	1.69%	0.78 [0.17-3.57]	0.792	0.910
TRPM3	93	2	2.15%	177	3	1.69%	0.78 [0.17-3.57]	0.792	0.910
UACA	93	2	2.15%	177	3	1.69%	0.78 [0.17-3.57]	0.792	0.910
UNC5D	93	2	2.15%	177	3	1.69%	0.78 [0.17-3.57]	0.792	0.910
ABCA13	93	5	5.38%	177	2	1.13%	0.20 [0.05-0.81]	0.0582	0.895
VPS13C	93	5	5.38%	177	2	1.13%	0.20 [0.05-0.81]	0.0582	0.895
AFF2	93	4	4.30%	177	2	1.13%	0.25 [0.06-1.07]	0.118	0.895
ALMS1	93	4	4.30%	177	2	1.13%	0.25 [0.06-1.07]	0.118	0.895
COL27A1	93	4	4.30%	177	2	1.13%	0.25 [0.06-1.07]	0.118	0.895
CPAMD8	93	4	4.30%	177	2	1.13%	0.25 [0.06-1.07]	0.118	0.895
DCHS2	93	4	4.30%	177	2	1.13%	0.25 [0.06-1.07]	0.118	0.895
DNAH1	93	4	4.30%	177	2	1.13%	0.25 [0.06-1.07]	0.118	0.895
KIAA0430	93	4	4.30%	177	2	1.13%	0.25 [0.06-1.07]	0.118	0.895
MYCBP2	93	4	4.30%	177	2	1.13%	0.25 [0.06-1.07]	0.118	0.895
PRRX1	93	4	4.30%	177	2	1.13%	0.25 [0.06-1.07]	0.118	0.895
PTPRT	93	4	4.30%	177	2	1.13%	0.25 [0.06-1.07]	0.118	0.895
TCHH	93	4	4.30%	177	2	1.13%	0.25 [0.06-1.07]	0.118	0.895
TP53BP1	93	4	4.30%	177	2	1.13%	0.25 [0.06-1.07]	0.118	0.895
VWA3B	93	4	4.30%	177	2	1.13%	0.25 [0.06-1.07]	0.118	0.895
WNK3	93	4	4.30%	177	2	1.13%	0.25 [0.06-1.07]	0.118	0.895
ADGB	93	3	3.23%	177	2	1.13%	0.34 [0.075-1.56]	0.246	0.895
AFF3	93	3	3.23%	177	2	1.13%	0.34 [0.075-1.56]	0.246	0.895
ARHGAP12	93	3	3.23%	177	2	1.13%	0.34 [0.075-1.56]	0.246	0.895
ATG9B	93	3	3.23%	177	2	1.13%	0.34 [0.075-1.56]	0.246	0.895
CBFB	93	3	3.23%	177	2	1.13%	0.34 [0.075-1.56]	0.246	0.895
CDH20	93	3	3.23%	177	2	1.13%	0.34 [0.075-1.56]	0.246	0.895
CDH9	93	3	3.23%	177	2	1.13%	0.34 [0.075-1.56]	0.246	0.895
CEP350	93	3	3.23%	177	2	1.13%	0.34 [0.075-1.56]	0.246	0.895
CHD1	93	3	3.23%	177	2	1.13%	0.34 [0.075-1.56]	0.246	0.895
DPP10	93	3	3.23%	177	2	1.13%	0.34 [0.075-1.56]	0.246	0.895
ELTD1	93	3	3.23%	177	2	1.13%	0.34 [0.075-1.56]	0.246	0.895
FREM1	93	3	3.23%	177	2	1.13%	0.34 [0.075-1.56]	0.246	0.895
FRYL	93	3	3.23%	177	2	1.13%	0.34 [0.075-1.56]	0.246	0.895
GRIK1	93	3	3.23%	177	2	1.13%	0.34 [0.075-1.56]	0.246	0.895
GTF3C1	93	3	3.23%	177	2	1.13%	0.34 [0.075-1.56]	0.246	0.895
IQSEC2	93	3	3.23%	177	2	1.13%	0.34 [0.075-1.56]	0.246	0.895
ITPR3	93	3	3.23%	177	2	1.13%	0.34 [0.075-1.56]	0.246	0.895
JAK1	93	3	3.23%	177	2	1.13%	0.34 [0.075-1.56]	0.246	0.895
KCNK18	93	3	3.23%	177	2	1.13%	0.34 [0.075-1.56]	0.246	0.895
KIAA1211	93	3	3.23%	177	2	1.13%	0.34 [0.075-1.56]	0.246	0.895
MYH10	93	3	3.23%	177	2	1.13%	0.34 [0.075-1.56]	0.246	0.895
MYO18B	93	3	3.23%	177	2	1.13%	0.34 [0.075-1.56]	0.246	0.895
NLRP2	93	3	3.23%	177	2	1.13%	0.34 [0.075-1.56]	0.246	0.895
ODZ1	93	3	3.23%	177	2	1.13%	0.34 [0.075-1.56]	0.246	0.895
PKD1	93	3	3.23%	177	2	1.13%	0.34 [0.075-1.56]	0.246	0.895
PLA2G4D	93	3	3.23%	177	2	1.13%	0.34 [0.075-1.56]	0.246	0.895
PLEKHG2	93	3	3.23%	177	2	1.13%	0.34 [0.075-1.56]	0.246	0.895
RBM12	93	3	3.23%	177	2	1.13%	0.34 [0.075-1.56]	0.246	0.895
RNF111	93	3	3.23%	177	2	1.13%	0.34 [0.075-1.56]	0.246	0.895
SPTBN2	93	3	3.23%	177	2	1.13%	0.34 [0.075-1.56]	0.246	0.895
SSH1	93	3	3.23%	177	2	1.13%	0.34 [0.075-1.56]	0.246	0.895
TEP1	93	3	3.23%	177	2	1.13%	0.34 [0.075-1.56]	0.246	0.895
TRAPPC8	93	3	3.23%	177	2	1.13%	0.34 [0.075-1.56]	0.246	0.895
USP25	93	3	3.23%	177	2	1.13%	0.34 [0.075-1.56]	0.246	0.895
WDR66	93	3	3.23%	177	2	1.13%	0.34 [0.075-1.56]	0.246	0.895
XDH	93	3	3.23%	177	2	1.13%	0.34 [0.075-1.56]	0.246	0.895
XIRP2	93	3	3.23%	177	2	1.13%	0.34 [0.075-1.56]	0.246	0.895
ZCCHC11	93	3	3.23%	177	2	1.13%	0.34 [0.075-1.56]	0.246	0.895
ZP4	93	3	3.23%	177	2	1.13%	0.34 [0.075-1.56]	0.246	0.895
MXRA5	93	6	6.45%	177	1	0.56%	0.082 [0.014-0.49]	2.18E-02	0.895
SCN2A	93	6	6.45%	177	1	0.56%	0.082 [0.014-0.49]	2.18E-02	0.895
SETX	93	6	6.45%	177	1	0.56%	0.082 [0.014-0.49]	2.18E-02	0.895
LRP1B	93	5	5.38%	177	1	0.56%	0.10 [0.016-0.61]	3.69E-02	0.895
PRKDC	93	5	5.38%	177	1	0.56%	0.10 [0.016-0.61]	3.69E-02	0.895
ASTN1	93	4	4.30%	177	1	0.56%	0.13 [0.02-0.81]	0.0661	0.895
DNAJC13	93	4	4.30%	177	1	0.56%	0.13 [0.02-0.81]	0.0661	0.895
DOCK8	93	4	4.30%	177	1	0.56%	0.13 [0.02-0.81]	0.0661	0.895
FN1	93	4	4.30%	177	1	0.56%	0.13 [0.02-0.81]	0.0661	0.895

Symbol	CINSARC Low			CINSARC High			CINSARC High vs. Low		
	N tested	N altered	Fq alteration	N tested	N altered	Fq alteration	OR [95%CI]	p-value*	q-value
GGT3P	93	4	4.30%	177	1	0.56%	0.13 [0.02-0.81]	0.0661	0.895
KIAA1731	93	4	4.30%	177	1	0.56%	0.13 [0.02-0.81]	0.0661	0.895
PCDH9	93	4	4.30%	177	1	0.56%	0.13 [0.02-0.81]	0.0661	0.895
PCNT	93	4	4.30%	177	1	0.56%	0.13 [0.02-0.81]	0.0661	0.895
SDK2	93	4	4.30%	177	1	0.56%	0.13 [0.02-0.81]	0.0661	0.895
SPATA13	93	4	4.30%	177	1	0.56%	0.13 [0.02-0.81]	0.0661	0.895
SSPO	93	4	4.30%	177	1	0.56%	0.13 [0.02-0.81]	0.0661	0.895
TEX15	93	4	4.30%	177	1	0.56%	0.13 [0.02-0.81]	0.0661	0.895
UBC	93	4	4.30%	177	1	0.56%	0.13 [0.02-0.81]	0.0661	0.895
UBE4B	93	4	4.30%	177	1	0.56%	0.13 [0.02-0.81]	0.0661	0.895

*, logistic regression

Supplementary Table 5: Comparison of frequency of amplifications among the 3,842 genes altered in at least 5/297 tested samples (TCGA) between the two CINSARC classes in Luminal B breast cancers.

Symbol	CINSARC Low			CINSARC High			CINSARC High vs. Low		
	N tested	N altered	Fq alteration	N tested	N altered	Fq alteration	OR [95%CI]	p-value*	q-value
RBFOX1	101	5	4.95%	196	5	2.55%	0.50 [0.17-1.45]	0.286	0.650
AANAT	101	3	2.97%	196	16	8.16%	2.90 [1.01-8.34]	0.097	0.507
AATF	101	2	1.98%	196	5	2.55%	1.30 [0.32-5.21]	0.759	0.996
AATK	101	2	1.98%	196	12	6.12%	3.23 [0.90-11.53]	0.130	0.535
ABAT	101	5	4.95%	196	5	2.55%	0.50 [0.17-1.45]	0.286	0.650
ABCA10	101	6	5.94%	196	20	10.20%	1.80 [0.81-3.98]	0.224	0.596
ABCA17P	101	6	5.94%	196	5	2.55%	0.41 [0.15-1.15]	0.154	0.535
ABCA3	101	6	5.94%	196	5	2.55%	0.41 [0.15-1.15]	0.154	0.535
ABCA5	101	5	4.95%	196	21	10.71%	2.30 [0.99-5.36]	0.104	0.507
ABCA6	101	6	5.94%	196	19	9.69%	1.70 [0.77-3.78]	0.274	0.643
ABCA8	101	7	6.93%	196	19	9.69%	1.44 [0.68-3.07]	0.427	0.791
ABCA9	101	6	5.94%	196	19	9.69%	1.70 [0.77-3.78]	0.274	0.643
ABCB10	101	6	5.94%	196	19	9.69%	1.70 [0.77-3.78]	0.274	0.643
ABCB5	101	0	0.00%	196	6	3.06%	2.7e+07 [0e+00-Inf]	0.992	1.000
ABCC12	101	1	0.99%	196	4	2.04%	2.08 [0.33-13.25]	0.514	0.805
ABCC1	101	7	6.93%	196	3	1.53%	0.21 [0.066-0.66]	2.55E-02	0.507
ABCC3	101	4	3.96%	196	19	9.69%	2.60 [1.03-6.59]	0.090	0.507
ABCC5	101	3	2.97%	196	2	1.02%	0.34 [0.074-1.53]	0.237	0.599
ABCC6P1	101	8	7.92%	196	5	2.55%	0.30 [0.12-0.80]	4.16E-02	0.507
ABCC6P2	101	11	10.89%	196	3	1.53%	0.13 [0.043-0.38]	1.89E-03	0.507
ABCC6	101	7	6.93%	196	3	1.53%	0.21 [0.066-0.66]	2.55E-02	0.507
ABCC8	101	1	0.99%	196	4	2.04%	2.08 [0.33-13.25]	0.514	0.805
ABCF3	101	3	2.97%	196	2	1.02%	0.34 [0.074-1.53]	0.237	0.599
ABHD15	101	3	2.97%	196	5	2.55%	0.86 [0.25-2.89]	0.833	1.000
ABHD2	101	1	0.99%	196	5	2.55%	2.62 [0.43-16.05]	0.383	0.750
ABI3	101	4	3.96%	196	17	8.67%	2.30 [0.90-5.88]	0.143	0.535
ABL2	101	5	4.95%	196	19	9.69%	2.06 [0.88-4.83]	0.163	0.535
ABRA	101	17	16.83%	196	52	26.53%	1.78 [1.07-2.98]	0.063	0.507
ACACA	101	2	1.98%	196	3	1.53%	0.77 [0.17-3.50]	0.776	0.996
ACAN	101	1	0.99%	196	6	3.06%	3.16 [0.53-18.88]	0.290	0.650
ACAP2	101	3	2.97%	196	3	1.53%	0.51 [0.13-1.98]	0.412	0.774
ACBD3	101	6	5.94%	196	19	9.69%	1.70 [0.77-3.78]	0.274	0.643
ACBD6	101	6	5.94%	196	19	9.69%	1.70 [0.77-3.78]	0.274	0.643
ACER3	101	7	6.93%	196	20	10.20%	1.53 [0.72-3.24]	0.355	0.722
ACE	101	8	7.92%	196	30	15.31%	2.10 [1.06-4.18]	0.076	0.507
ACIN1	101	1	0.99%	196	4	2.04%	2.08 [0.33-13.25]	0.514	0.805
ACOT1	101	12	11.88%	196	24	12.24%	1.03 [0.56-1.92]	0.928	1.000
ACOT8	101	5	4.95%	196	4	2.04%	0.40 [0.13-1.23]	0.179	0.535
ACOX1	101	4	3.96%	196	12	6.12%	1.58 [0.60-4.18]	0.438	0.798
ACP6	101	6	5.94%	196	12	6.12%	1.03 [0.44-2.41]	0.950	1.000
ACSF2	101	4	3.96%	196	19	9.69%	2.60 [1.03-6.59]	0.090	0.507
ACSM1	101	5	4.95%	196	2	1.02%	0.20 [0.049-0.80]	0.056	0.507
ACSM2A	101	4	3.96%	196	2	1.02%	0.25 [0.059-1.05]	0.113	0.511
ACSM2B	101	5	4.95%	196	2	1.02%	0.20 [0.049-0.80]	0.056	0.507
ACSM3	101	5	4.95%	196	2	1.02%	0.20 [0.049-0.80]	0.056	0.507
ACSM5	101	5	4.95%	196	3	1.53%	0.30 [0.088-1.01]	0.103	0.507
ACSS2	101	4	3.96%	196	7	3.57%	0.90 [0.31-2.57]	0.867	1.000
ACSS3	101	1	0.99%	196	4	2.04%	2.08 [0.33-13.25]	0.514	0.805
ACTA1	101	6	5.94%	196	19	9.69%	1.70 [0.77-3.78]	0.274	0.643
ACTG1	101	2	1.98%	196	12	6.12%	3.23 [0.90-11.53]	0.130	0.535
ACTL6A	101	4	3.96%	196	2	1.02%	0.25 [0.059-1.05]	0.113	0.511
ACTN2	101	7	6.93%	196	19	9.69%	1.44 [0.68-3.07]	0.427	0.791
ACTN3	101	5	4.95%	196	12	6.12%	1.25 [0.51-3.08]	0.681	0.943
ACTR5	101	2	1.98%	196	5	2.55%	1.30 [0.32-5.21]	0.759	0.996
ACY3	101	10	9.90%	196	19	9.69%	0.98 [0.50-1.92]	0.955	1.000
ADAM15	101	8	7.92%	196	15	7.65%	0.96 [0.46-2.04]	0.935	1.000
ADAM18	101	14	13.86%	196	37	18.88%	1.45 [0.83-2.53]	0.279	0.650
ADAM2	101	13	12.87%	196	32	16.33%	1.32 [0.74-2.37]	0.432	0.798
ADAM30	101	1	0.99%	196	4	2.04%	2.08 [0.33-13.25]	0.514	0.805
ADAM32	101	12	11.88%	196	36	18.37%	1.67 [0.93-3.01]	0.153	0.535
ADAM3A	101	25	24.75%	196	53	27.04%	1.13 [0.71-1.79]	0.671	0.943
ADAM5	101	17	16.83%	196	35	17.86%	1.07 [0.63-1.83]	0.826	1.000
ADAM9	101	12	11.88%	196	40	20.41%	1.90 [1.06-3.41]	0.070	0.507
ADAMTS4	101	6	5.94%	196	16	8.16%	1.41 [0.62-3.18]	0.490	0.805
ADAMTSL4	101	9	8.91%	196	17	8.67%	0.97 [0.48-1.97]	0.945	1.000
ADAP2	101	2	1.98%	196	3	1.53%	0.77 [0.17-3.50]	0.776	0.996
ADAR	101	7	6.93%	196	12	6.12%	0.88 [0.39-1.97]	0.788	0.996
ADA	101	4	3.96%	196	4	2.04%	0.51 [0.16-1.65]	0.342	0.716

Symbol	CINSARC Low			CINSARC High			CINSARC High vs. Low		
	N tested	N altered	Fq alteration	N tested	N altered	Fq alteration	OR [95%CI]	p-value*	q-value
ADCK2	101	1	0.99%	196	4	2.04%	2.08 [0.33-13.25]	0.514	0.805
ADCK5	101	18	17.82%	196	49	25.00%	1.54 [0.93-2.55]	0.163	0.535
ADCY10	101	4	3.96%	196	12	6.12%	1.58 [0.60-4.18]	0.438	0.798
ADCY8	101	15	14.85%	196	55	28.06%	2.24 [1.32-3.80]	1.24E-02	0.507
ADCY9	101	5	4.95%	196	6	3.06%	0.61 [0.22-1.68]	0.418	0.779
ADCYAP1R1	101	0	0.00%	196	7	3.57%	3.2e+07 [0e+00-Inf]	0.992	1.000
ADHFE1	101	15	14.85%	196	39	19.90%	1.42 [0.82-2.46]	0.287	0.650
ADIG	101	4	3.96%	196	5	2.55%	0.63 [0.21-1.95]	0.505	0.805
ADIPOR1	101	7	6.93%	196	28	14.29%	2.24 [1.08-4.63]	0.068	0.507
ADK	101	2	1.98%	196	5	2.55%	1.30 [0.32-5.21]	0.759	0.996
ADNP	101	6	5.94%	196	13	6.63%	1.12 [0.49-2.60]	0.817	1.000
ADORA1	101	7	6.93%	196	28	14.29%	2.24 [1.08-4.63]	0.068	0.507
ADRB3	101	10	9.90%	196	51	26.02%	3.20 [1.74-5.89]	1.70E-03	0.507
ADRM1	101	6	5.94%	196	10	5.10%	0.85 [0.36-2.04]	0.762	0.996
ADSS	101	5	4.95%	196	19	9.69%	2.06 [0.88-4.83]	0.163	0.535
AEN	101	0	0.00%	196	6	3.06%	2.7e+07 [0e+00-Inf]	0.992	1.000
AFMID	101	3	2.97%	196	17	8.67%	3.10 [1.09-8.87]	0.076	0.507
AFM	101	1	0.99%	196	6	3.06%	3.16 [0.53-18.88]	0.290	0.650
AFP	101	1	0.99%	196	6	3.06%	3.16 [0.53-18.88]	0.290	0.650
AGAP2	101	0	0.00%	196	5	2.55%	2.2e+07 [0e+00-Inf]	0.992	1.000
AGAP5	101	0	0.00%	196	5	2.55%	2.2e+07 [0e+00-Inf]	0.992	1.000
AGBL1	101	1	0.99%	196	5	2.55%	2.62 [0.43-16.05]	0.383	0.750
AGT	101	6	5.94%	196	19	9.69%	1.70 [0.77-3.78]	0.274	0.643
AHCTF1	101	5	4.95%	196	19	9.69%	2.06 [0.88-4.83]	0.163	0.535
AHSP	101	4	3.96%	196	4	2.04%	0.51 [0.16-1.65]	0.342	0.716
AIDA	101	6	5.94%	196	19	9.69%	1.70 [0.77-3.78]	0.274	0.643
AIM2	101	6	5.94%	196	8	4.08%	0.67 [0.27-1.68]	0.476	0.805
AIP	101	11	10.89%	196	20	10.20%	0.93 [0.48-1.79]	0.854	1.000
AKAP10	101	4	3.96%	196	2	1.02%	0.25 [0.059-1.05]	0.113	0.511
AKAP13	101	1	0.99%	196	6	3.06%	3.16 [0.53-18.88]	0.290	0.650
AKAP1	101	7	6.93%	196	25	12.76%	1.96 [0.94-4.09]	0.131	0.535
AKAP6	101	1	0.99%	196	7	3.57%	3.70 [0.63-21.75]	0.224	0.596
AKT3	101	5	4.95%	196	18	9.18%	1.94 [0.82-4.58]	0.203	0.570
ALB	101	1	0.99%	196	6	3.06%	3.16 [0.53-18.88]	0.290	0.650
ALDH3A1	101	4	3.96%	196	1	0.51%	0.12 [0.02-0.79]	0.064	0.507
ALDH3A2	101	4	3.96%	196	1	0.51%	0.12 [0.02-0.79]	0.064	0.507
ALDH3B1	101	9	8.91%	196	21	10.71%	1.23 [0.62-2.44]	0.626	0.907
ALDH3B2	101	9	8.91%	196	19	9.69%	1.10 [0.55-2.21]	0.827	1.000
ALDH9A1	101	4	3.96%	196	12	6.12%	1.58 [0.60-4.18]	0.438	0.798
ALDOA	101	5	4.95%	196	4	2.04%	0.40 [0.13-1.23]	0.179	0.535
ALDOC	101	3	2.97%	196	8	4.08%	1.39 [0.45-4.31]	0.632	0.909
ALG1	101	5	4.95%	196	5	2.55%	0.50 [0.17-1.45]	0.286	0.650
ALG3	101	3	2.97%	196	2	1.02%	0.34 [0.074-1.53]	0.237	0.599
ALG8	101	9	8.91%	196	18	9.18%	1.03 [0.51-2.09]	0.938	1.000
ALPK3	101	1	0.99%	196	6	3.06%	3.16 [0.53-18.88]	0.290	0.650
CCL18	101	2	1.98%	196	5	2.55%	1.30 [0.32-5.21]	0.759	0.996
AMDHD2	101	6	5.94%	196	5	2.55%	0.41 [0.15-1.15]	0.154	0.535
AMELY	101	0	0.00%	196	0	0.00%	1.00 [0e+00-Inf]	1.000	1.000
AMZ2P1	101	8	7.92%	196	23	11.73%	1.55 [0.76-3.14]	0.311	0.680
AMZ2	101	10	9.90%	196	27	13.78%	1.45 [0.76-2.77]	0.340	0.716
ANAPC11	101	2	1.98%	196	13	6.63%	3.52 [0.99-12.47]	0.102	0.507
ANGEL2	101	6	5.94%	196	21	10.71%	1.90 [0.86-4.19]	0.181	0.535
ANGPT1	101	15	14.85%	196	51	26.02%	2.02 [1.18-3.43]	3.03E-02	0.507
ANGPTL1	101	5	4.95%	196	18	9.18%	1.94 [0.82-4.58]	0.203	0.570
ANK1	101	15	14.85%	196	32	16.33%	1.12 [0.64-1.96]	0.742	0.996
ANK3	101	0	0.00%	196	5	2.55%	2.2e+07 [0e+00-Inf]	0.992	1.000
ANKFN1	101	9	8.91%	196	21	10.71%	1.23 [0.62-2.44]	0.626	0.907
ANKRD13B	101	3	2.97%	196	5	2.55%	0.86 [0.25-2.89]	0.833	1.000
ANKRD13D	101	10	9.90%	196	16	8.16%	0.81 [0.40-1.62]	0.616	0.898
ANKRD17	101	1	0.99%	196	7	3.57%	3.70 [0.63-21.75]	0.224	0.596
ANKRD34A	101	5	4.95%	196	10	5.10%	1.03 [0.41-2.60]	0.955	1.000
ANKRD35	101	5	4.95%	196	10	5.10%	1.03 [0.41-2.60]	0.955	1.000
ANKRD36BP1	101	4	3.96%	196	13	6.63%	1.72 [0.66-4.51]	0.353	0.722
ANKRD40	101	4	3.96%	196	21	10.71%	2.91 [1.16-7.31]	0.056	0.507
ANKRD45	101	5	4.95%	196	13	6.63%	1.36 [0.56-3.32]	0.566	0.852
ANKRD46	101	15	14.85%	196	55	28.06%	2.24 [1.32-3.80]	1.24E-02	0.507
ANKS3	101	5	4.95%	196	5	2.55%	0.50 [0.17-1.45]	0.286	0.650
ANKS4B	101	5	4.95%	196	2	1.02%	0.20 [0.049-0.80]	0.056	0.507
ANO1	101	21	20.79%	196	57	29.08%	1.56 [0.97-2.52]	0.126	0.535
ANO5	101	3	2.97%	196	2	1.02%	0.34 [0.074-1.53]	0.237	0.599
ANP32E	101	8	7.92%	196	15	7.65%	0.96 [0.46-2.04]	0.935	1.000

Symbol	CINSARC Low			CINSARC High			CINSARC High vs. Low		
	N tested	N altered	Fq alteration	N tested	N altered	Fq alteration	OR [95%CI]	p-value*	q-value
ANPEP	101	1	0.99%	196	8	4.08%	4.26 [0.73-24.65]	0.175	0.535
ANTXRL	101	1	0.99%	196	5	2.55%	2.62 [0.43-16.05]	0.383	0.750
ANXA13	101	14	13.86%	196	57	29.08%	2.55 [1.49-4.37]	4.36E-03	0.507
ANXA7	101	0	0.00%	196	5	2.55%	2.2e+07 [0e+00-Inf]	0.992	1.000
ANXA9	101	7	6.93%	196	15	7.65%	1.11 [0.51-2.43]	0.822	1.000
AP2B1	101	2	1.98%	196	4	2.04%	1.03 [0.24-4.35]	0.972	1.000
AP2M1	101	3	2.97%	196	2	1.02%	0.34 [0.074-1.53]	0.237	0.599
AP3B2	101	1	0.99%	196	4	2.04%	2.08 [0.33-13.25]	0.514	0.805
AP3M1	101	0	0.00%	196	5	2.55%	2.2e+07 [0e+00-Inf]	0.992	1.000
AP3M2	101	14	13.86%	196	33	16.84%	1.26 [0.71-2.22]	0.506	0.805
AP3S2	101	1	0.99%	196	9	4.59%	4.81 [0.84-27.58]	0.139	0.535
AP4S1	101	0	0.00%	196	7	3.57%	3.2e+07 [0e+00-Inf]	0.992	1.000
APCDD1L	101	8	7.92%	196	17	8.67%	1.10 [0.53-2.30]	0.825	1.000
APCS	101	6	5.94%	196	10	5.10%	0.85 [0.36-2.04]	0.762	0.996
APH1A	101	8	7.92%	196	15	7.65%	0.96 [0.46-2.04]	0.935	1.000
APOA2	101	6	5.94%	196	16	8.16%	1.41 [0.62-3.18]	0.490	0.805
APOBR	101	5	4.95%	196	3	1.53%	0.30 [0.088-1.01]	0.103	0.507
APOBEC4	101	5	4.95%	196	20	10.20%	2.18 [0.93-5.10]	0.130	0.535
APOD	101	2	1.98%	196	4	2.04%	1.03 [0.24-4.35]	0.972	1.000
APOH	101	9	8.91%	196	25	12.76%	1.49 [0.76-2.93]	0.327	0.712
APPBP2	101	8	7.92%	196	34	17.35%	2.44 [1.23-4.82]	3.12E-02	0.507
AQP10	101	7	6.93%	196	12	6.12%	0.88 [0.39-1.97]	0.788	0.996
AQP11	101	10	9.90%	196	22	11.22%	1.15 [0.59-2.23]	0.728	0.986
AQP1	101	0	0.00%	196	7	3.57%	3.2e+07 [0e+00-Inf]	0.992	1.000
AQP8	101	5	4.95%	196	4	2.04%	0.40 [0.13-1.23]	0.179	0.535
ARAP1	101	5	4.95%	196	10	5.10%	1.03 [0.41-2.60]	0.955	1.000
ARC	101	16	15.84%	196	49	25.00%	1.77 [1.05-2.99]	0.073	0.507
AREG	101	2	1.98%	196	3	1.53%	0.77 [0.17-3.50]	0.776	0.996
ARF1	101	6	5.94%	196	21	10.71%	1.90 [0.86-4.19]	0.181	0.535
ARFGAP1	101	7	6.93%	196	13	6.63%	0.95 [0.43-2.12]	0.923	1.000
ARFGEF1	101	15	14.85%	196	39	19.90%	1.42 [0.82-2.46]	0.287	0.650
ARFGEF2	101	6	5.94%	196	8	4.08%	0.67 [0.27-1.68]	0.476	0.805
ARFRP1	101	7	6.93%	196	15	7.65%	1.11 [0.51-2.43]	0.822	1.000
ARHGAP17	101	5	4.95%	196	3	1.53%	0.30 [0.088-1.01]	0.103	0.507
ARHGAP23	101	5	4.95%	196	7	3.57%	0.71 [0.27-1.90]	0.569	0.855
ARHGAP30	101	6	5.94%	196	17	8.67%	1.50 [0.67-3.38]	0.407	0.774
ARHGAP39	101	17	16.83%	196	49	25.00%	1.65 [0.98-2.76]	0.111	0.511
ARHGAP5	101	1	0.99%	196	7	3.57%	3.70 [0.63-21.75]	0.224	0.596
ARHGDIA	101	2	1.98%	196	13	6.63%	3.52 [0.99-12.47]	0.102	0.507
ARHGDIG	101	7	6.93%	196	4	2.04%	0.28 [0.098-0.80]	4.63E-02	0.507
ARHGEF11	101	6	5.94%	196	8	4.08%	0.67 [0.27-1.68]	0.476	0.805
ARHGEF17	101	7	6.93%	196	11	5.61%	0.80 [0.35-1.82]	0.652	0.928
ARHGEF2	101	7	6.93%	196	9	4.59%	0.65 [0.27-1.52]	0.401	0.774
ARID3C	101	1	0.99%	196	4	2.04%	2.08 [0.33-13.25]	0.514	0.805
ARID4B	101	7	6.93%	196	20	10.20%	1.53 [0.72-3.24]	0.355	0.722
ARL16	101	2	1.98%	196	12	6.12%	3.23 [0.90-11.53]	0.130	0.535
ARL17B	101	8	7.92%	196	10	5.10%	0.63 [0.28-1.40]	0.338	0.716
ARL5C	101	4	3.96%	196	12	6.12%	1.58 [0.60-4.18]	0.438	0.798
ARL6IP1	101	5	4.95%	196	4	2.04%	0.40 [0.13-1.23]	0.179	0.535
ARL8A	101	8	7.92%	196	24	12.24%	1.62 [0.80-3.28]	0.258	0.635
ARMC1	101	15	14.85%	196	35	17.86%	1.25 [0.72-2.17]	0.512	0.805
ARMC2	101	1	0.99%	196	4	2.04%	2.08 [0.33-13.25]	0.514	0.805
ARMC5	101	4	3.96%	196	4	2.04%	0.51 [0.16-1.65]	0.342	0.716
ARMC7	101	5	4.95%	196	12	6.12%	1.25 [0.51-3.08]	0.681	0.943
ARNT2	101	2	1.98%	196	4	2.04%	1.03 [0.24-4.35]	0.972	1.000
ARNT	101	8	7.92%	196	15	7.65%	0.96 [0.46-2.04]	0.935	1.000
ARPC5	101	5	4.95%	196	20	10.20%	2.18 [0.93-5.10]	0.130	0.535
ARRB1	101	5	4.95%	196	15	7.65%	1.59 [0.66-3.81]	0.382	0.750
ARRDC4	101	0	0.00%	196	9	4.59%	4.1e+07 [0e+00-Inf]	0.992	1.000
ARSG	101	9	8.91%	196	28	14.29%	1.70 [0.88-3.31]	0.188	0.553
ART3	101	1	0.99%	196	5	2.55%	2.62 [0.43-16.05]	0.383	0.750
ARV1	101	6	5.94%	196	19	9.69%	1.70 [0.77-3.78]	0.274	0.643
ASAP1-IT1	101	15	14.85%	196	60	30.61%	2.53 [1.49-4.28]	3.72E-03	0.507
ASAP1	101	15	14.85%	196	58	29.59%	2.41 [1.42-4.08]	6.08E-03	0.507
ASB7	101	1	0.99%	196	4	2.04%	2.08 [0.33-13.25]	0.514	0.805
ASH1L	101	6	5.94%	196	11	5.61%	0.94 [0.40-2.23]	0.908	1.000
ASH2L	101	11	10.89%	196	47	23.98%	2.58 [1.43-4.67]	8.55E-03	0.507
ASPHD1	101	5	4.95%	196	4	2.04%	0.40 [0.13-1.23]	0.179	0.535
ASPH	101	15	14.85%	196	32	16.33%	1.12 [0.64-1.96]	0.742	0.996
ASPM	101	5	4.95%	196	21	10.71%	2.30 [0.99-5.36]	0.104	0.507
ASPSCR1	101	2	1.98%	196	13	6.63%	3.52 [0.99-12.47]	0.102	0.507

Symbol	CINSARC Low			CINSARC High			CINSARC High vs. Low		
	N tested	N altered	Fq alteration	N tested	N altered	Fq alteration	OR [95%CI]	p-value*	q-value
ASTN1	101	6	5.94%	196	16	8.16%	1.41 [0.62-3.18]	0.490	0.805
ATAD2	101	14	13.86%	196	58	29.59%	2.61 [1.52-4.48]	3.40E-03	0.507
ATAD5	101	2	1.98%	196	3	1.53%	0.77 [0.17-3.50]	0.776	0.996
ATF3	101	6	5.94%	196	21	10.71%	1.90 [0.86-4.19]	0.181	0.535
ATF6	101	5	4.95%	196	17	8.67%	1.82 [0.77-4.32]	0.252	0.623
ATF7IP2	101	5	4.95%	196	4	2.04%	0.40 [0.13-1.23]	0.179	0.535
ATG16L2	101	5	4.95%	196	10	5.10%	1.03 [0.41-2.60]	0.955	1.000
ATG5	101	2	1.98%	196	7	3.57%	1.83 [0.48-6.96]	0.455	0.805
ATP11B	101	3	2.97%	196	2	1.02%	0.34 [0.074-1.53]	0.237	0.599
ATP13A3	101	3	2.97%	196	3	1.53%	0.51 [0.13-1.98]	0.412	0.774
ATP13A4	101	3	2.97%	196	3	1.53%	0.51 [0.13-1.98]	0.412	0.774
ATP13A5	101	3	2.97%	196	3	1.53%	0.51 [0.13-1.98]	0.412	0.774
ATP1A2	101	6	5.94%	196	12	6.12%	1.03 [0.44-2.41]	0.950	1.000
ATP1A4	101	6	5.94%	196	12	6.12%	1.03 [0.44-2.41]	0.950	1.000
ATP1B1	101	5	4.95%	196	12	6.12%	1.25 [0.51-3.08]	0.681	0.943
ATP2A1	101	6	5.94%	196	3	1.53%	0.25 [0.076-0.80]	0.051	0.507
ATP2B4	101	7	6.93%	196	28	14.29%	2.24 [1.08-4.63]	0.068	0.507
ATP6V0C	101	6	5.94%	196	5	2.55%	0.41 [0.15-1.15]	0.154	0.535
ATP6V0D2	101	18	17.82%	196	46	23.47%	1.41 [0.85-2.35]	0.263	0.643
ATP6V1C1	101	16	15.84%	196	56	28.57%	2.13 [1.27-3.57]	1.67E-02	0.507
ATP6V1G3	101	5	4.95%	196	22	11.22%	2.43 [1.05-5.63]	0.083	0.507
ATP6V1H	101	12	11.88%	196	31	15.82%	1.39 [0.77-2.54]	0.363	0.738
ATP8B2	101	7	6.93%	196	12	6.12%	0.88 [0.39-1.97]	0.788	0.996
ATP9A	101	6	5.94%	196	19	9.69%	1.70 [0.77-3.78]	0.274	0.643
ATXN2L	101	6	5.94%	196	3	1.53%	0.25 [0.076-0.80]	0.051	0.507
ATXN7L3B	101	1	0.99%	196	7	3.57%	3.70 [0.63-21.75]	0.224	0.596
AURKA	101	8	7.92%	196	13	6.63%	0.83 [0.38-1.78]	0.682	0.943
AVIL	101	0	0.00%	196	5	2.55%	2.2e+07 [0e+00-Inf]	0.992	1.000
AVL9	101	0	0.00%	196	7	3.57%	3.2e+07 [0e+00-Inf]	0.992	1.000
AVPR1A	101	1	0.99%	196	5	2.55%	2.62 [0.43-16.05]	0.383	0.750
AVPR1B	101	6	5.94%	196	24	12.24%	2.21 [1.01-4.82]	0.095	0.507
AXIN1	101	7	6.93%	196	4	2.04%	0.28 [0.098-0.80]	4.63E-02	0.507
AXIN2	101	9	8.91%	196	27	13.78%	1.63 [0.84-3.19]	0.227	0.599
AZIN1	101	16	15.84%	196	56	28.57%	2.13 [1.27-3.57]	1.67E-02	0.507
B3GALNT2	101	7	6.93%	196	20	10.20%	1.53 [0.72-3.24]	0.355	0.722
B3GALT2	101	5	4.95%	196	18	9.18%	1.94 [0.82-4.58]	0.203	0.570
B3GNT5	101	3	2.97%	196	2	1.02%	0.34 [0.074-1.53]	0.237	0.599
B3GNT6	101	7	6.93%	196	20	10.20%	1.53 [0.72-3.24]	0.355	0.722
B3GNTL1	101	2	1.98%	196	15	7.65%	4.10 [1.17-14.39]	0.064	0.507
B4GALNT2	101	3	2.97%	196	17	8.67%	3.10 [1.09-8.87]	0.076	0.507
B4GALT3	101	6	5.94%	196	16	8.16%	1.41 [0.62-3.18]	0.490	0.805
B4GALT5	101	7	6.93%	196	14	7.14%	1.03 [0.47-2.28]	0.946	1.000
B9D1	101	4	3.96%	196	2	1.02%	0.25 [0.059-1.05]	0.113	0.511
BAALC	101	16	15.84%	196	57	29.08%	2.18 [1.30-3.66]	1.33E-02	0.507
BAG4	101	11	10.89%	196	47	23.98%	2.58 [1.43-4.67]	8.55E-03	0.507
BAHCC1	101	2	1.98%	196	12	6.12%	3.23 [0.90-11.53]	0.130	0.535
BAIAP2	101	2	1.98%	196	12	6.12%	3.23 [0.90-11.53]	0.130	0.535
BAIAP3	101	6	5.94%	196	5	2.55%	0.41 [0.15-1.15]	0.154	0.535
PRRC2C	101	5	4.95%	196	13	6.63%	1.36 [0.56-3.32]	0.566	0.852
BATF3	101	6	5.94%	196	21	10.71%	1.90 [0.86-4.19]	0.181	0.535
BAZ1A	101	0	0.00%	196	8	4.08%	3.6e+07 [0e+00-Inf]	0.992	1.000
BBS10	101	1	0.99%	196	6	3.06%	3.16 [0.53-18.88]	0.290	0.650
BBS1	101	5	4.95%	196	11	5.61%	1.14 [0.46-2.84]	0.811	1.000
BBS9	101	0	0.00%	196	8	4.08%	3.6e+07 [0e+00-Inf]	0.992	1.000
BCAN	101	7	6.93%	196	8	4.08%	0.57 [0.24-1.37]	0.294	0.650
BCAR4	101	5	4.95%	196	3	1.53%	0.30 [0.088-1.01]	0.103	0.507
BCAS1	101	11	10.89%	196	22	11.22%	1.03 [0.54-1.97]	0.931	1.000
BCAS3	101	6	5.94%	196	33	16.84%	3.21 [1.50-6.86]	1.17E-02	0.507
BCAS4	101	5	4.95%	196	14	7.14%	1.48 [0.61-3.57]	0.467	0.805
BCKDK	101	4	3.96%	196	5	2.55%	0.63 [0.21-1.95]	0.505	0.805
BCL2	101	3	2.97%	196	2	1.02%	0.34 [0.074-1.53]	0.237	0.599
BCL7C	101	4	3.96%	196	5	2.55%	0.63 [0.21-1.95]	0.505	0.805
NBEAP1	101	1	0.99%	196	4	2.04%	2.08 [0.33-13.25]	0.514	0.805
BCL9	101	6	5.94%	196	12	6.12%	1.03 [0.44-2.41]	0.950	1.000
BCORP1	101	0	0.00%	196	0	0.00%	1.00 [0e+00-Inf]	1.000	1.000
BDH1	101	3	2.97%	196	4	2.04%	0.68 [0.19-2.43]	0.619	0.898
BEND3	101	1	0.99%	196	9	4.59%	4.81 [0.84-27.58]	0.139	0.535
BEST3	101	2	1.98%	196	15	7.65%	4.10 [1.17-14.39]	0.064	0.507
LINC00293	101	12	11.88%	196	26	13.27%	1.13 [0.61-2.09]	0.735	0.993
BFAR	101	7	6.93%	196	3	1.53%	0.21 [0.066-0.66]	2.55E-02	0.507
BGLAP	101	6	5.94%	196	9	4.59%	0.76 [0.31-1.86]	0.616	0.898

Symbol	CINSARC Low			CINSARC High			CINSARC High vs. Low		
	N tested	N altered	Fq alteration	N tested	N altered	Fq alteration	OR [95%CI]	p-value*	q-value
BHLHE22	101	14	13.86%	196	35	17.86%	1.35 [0.77-2.38]	0.381	0.750
BHLHE23	101	7	6.93%	196	13	6.63%	0.95 [0.43-2.12]	0.923	1.000
BIRC5	101	3	2.97%	196	17	8.67%	3.10 [1.09-8.87]	0.076	0.507
BIRC7	101	7	6.93%	196	13	6.63%	0.95 [0.43-2.12]	0.923	1.000
BLCAP	101	5	4.95%	196	3	1.53%	0.30 [0.088-1.01]	0.103	0.507
BLMH	101	2	1.98%	196	5	2.55%	1.30 [0.32-5.21]	0.759	0.996
BLM	101	1	0.99%	196	6	3.06%	3.16 [0.53-18.88]	0.290	0.650
BLZF1	101	4	3.96%	196	13	6.63%	1.72 [0.66-4.51]	0.353	0.722
BMP7	101	8	7.92%	196	17	8.67%	1.10 [0.53-2.30]	0.825	1.000
BMPER	101	0	0.00%	196	5	2.55%	2.2e+07 [0e+00-Inf]	0.992	1.000
BMS1P4	101	0	0.00%	196	5	2.55%	2.2e+07 [0e+00-Inf]	0.992	1.000
BNIPL	101	7	6.93%	196	14	7.14%	1.03 [0.47-2.28]	0.946	1.000
BOD1	101	2	1.98%	196	3	1.53%	0.77 [0.17-3.50]	0.776	0.996
BOLA1	101	6	5.94%	196	14	7.14%	1.22 [0.53-2.79]	0.696	0.951
BOLA2	101	4	3.96%	196	4	2.04%	0.51 [0.16-1.65]	0.342	0.716
BOP1	101	19	18.81%	196	50	25.51%	1.48 [0.90-2.43]	0.197	0.570
BPI	101	4	3.96%	196	4	2.04%	0.51 [0.16-1.65]	0.342	0.716
BPNT1	101	6	5.94%	196	20	10.20%	1.80 [0.81-3.98]	0.224	0.596
BPTF	101	10	9.90%	196	27	13.78%	1.45 [0.76-2.77]	0.340	0.716
BPY2	101	0	0.00%	196	0	0.00%	1.00 [0e+00-Inf]	1.000	1.000
BRAF	101	1	0.99%	196	4	2.04%	2.08 [0.33-13.25]	0.514	0.805
BREA2	101	17	16.83%	196	51	26.02%	1.74 [1.04-2.90]	0.076	0.507
BRF2	101	11	10.89%	196	51	26.02%	2.88 [1.60-5.19]	3.19E-03	0.507
BRIP1	101	9	8.91%	196	29	14.80%	1.78 [0.91-3.44]	0.155	0.535
BRMS1L	101	0	0.00%	196	9	4.59%	4.1e+07 [0e+00-Inf]	0.992	1.000
BRMS1	101	4	3.96%	196	7	3.57%	0.90 [0.31-2.57]	0.867	1.000
MPC2	101	4	3.96%	196	12	6.12%	1.58 [0.60-4.18]	0.438	0.798
BRSK1	101	1	0.99%	196	4	2.04%	2.08 [0.33-13.25]	0.514	0.805
SLX4	101	5	4.95%	196	4	2.04%	0.40 [0.13-1.23]	0.179	0.535
BTBD17	101	7	6.93%	196	14	7.14%	1.03 [0.47-2.28]	0.946	1.000
BTC	101	2	1.98%	196	3	1.53%	0.77 [0.17-3.50]	0.776	0.996
BTG1	101	0	0.00%	196	5	2.55%	2.2e+07 [0e+00-Inf]	0.992	1.000
BTG2	101	7	6.93%	196	28	14.29%	2.24 [1.08-4.63]	0.068	0.507
BTNL3	101	1	0.99%	196	4	2.04%	2.08 [0.33-13.25]	0.514	0.805
BVES	101	1	0.99%	196	6	3.06%	3.16 [0.53-18.88]	0.290	0.650
C10orf105	101	0	0.00%	196	5	2.55%	2.2e+07 [0e+00-Inf]	0.992	1.000
ZNF503-AS2	101	2	1.98%	196	5	2.55%	1.30 [0.32-5.21]	0.759	0.996
C10orf55	101	0	0.00%	196	5	2.55%	2.2e+07 [0e+00-Inf]	0.992	1.000
C11orf24	101	8	7.92%	196	18	9.18%	1.18 [0.57-2.44]	0.715	0.972
KIAA1549L	101	1	0.99%	196	4	2.04%	2.08 [0.33-13.25]	0.514	0.805
ANAPC15	101	4	3.96%	196	6	3.06%	0.77 [0.26-2.26]	0.685	0.943
C11orf58	101	1	0.99%	196	4	2.04%	2.08 [0.33-13.25]	0.514	0.805
LAMTOR1	101	4	3.96%	196	6	3.06%	0.77 [0.26-2.26]	0.685	0.943
PPP1R32	101	1	0.99%	196	5	2.55%	2.62 [0.43-16.05]	0.383	0.750
AAMDC	101	10	9.90%	196	22	11.22%	1.15 [0.59-2.23]	0.728	0.986
C11orf80	101	8	7.92%	196	13	6.63%	0.83 [0.38-1.78]	0.682	0.943
C11orf86	101	7	6.93%	196	12	6.12%	0.88 [0.39-1.97]	0.788	0.996
C12orf29	101	0	0.00%	196	5	2.55%	2.2e+07 [0e+00-Inf]	0.992	1.000
C12orf50	101	0	0.00%	196	5	2.55%	2.2e+07 [0e+00-Inf]	0.992	1.000
C12orf66	101	1	0.99%	196	5	2.55%	2.62 [0.43-16.05]	0.383	0.750
C12orf74	101	0	0.00%	196	5	2.55%	2.2e+07 [0e+00-Inf]	0.992	1.000
DTD2	101	1	0.99%	196	8	4.08%	4.26 [0.73-24.65]	0.175	0.535
ARHGAP5-AS1	101	1	0.99%	196	6	3.06%	3.16 [0.53-18.88]	0.290	0.650
SPTSSA	101	0	0.00%	196	8	4.08%	3.6e+07 [0e+00-Inf]	0.992	1.000
IGBP1P1	101	0	0.00%	196	9	4.59%	4.1e+07 [0e+00-Inf]	0.992	1.000
C15orf32	101	0	0.00%	196	7	3.57%	3.2e+07 [0e+00-Inf]	0.992	1.000
HEXA-AS1	101	1	0.99%	196	4	2.04%	2.08 [0.33-13.25]	0.514	0.805
TICRR	101	1	0.99%	196	7	3.57%	3.70 [0.63-21.75]	0.224	0.596
DNM1P46	101	1	0.99%	196	5	2.55%	2.62 [0.43-16.05]	0.383	0.750
GDPGP1	101	2	1.98%	196	7	3.57%	1.83 [0.48-6.96]	0.455	0.805
TSR3	101	6	5.94%	196	5	2.55%	0.41 [0.15-1.15]	0.154	0.535
PAGR1	101	5	4.95%	196	3	1.53%	0.30 [0.088-1.01]	0.103	0.507
C16orf54	101	5	4.95%	196	3	1.53%	0.30 [0.088-1.01]	0.103	0.507
C16orf58	101	4	3.96%	196	4	2.04%	0.51 [0.16-1.65]	0.342	0.716
CDIP1	101	5	4.95%	196	6	3.06%	0.61 [0.22-1.68]	0.418	0.779
FOPNL	101	8	7.92%	196	3	1.53%	0.18 [0.058-0.56]	1.30E-02	0.507
METTL22	101	5	4.95%	196	5	2.55%	0.50 [0.17-1.45]	0.286	0.650
C16orf71	101	5	4.95%	196	5	2.55%	0.50 [0.17-1.45]	0.286	0.650
C16orf72	101	6	5.94%	196	4	2.04%	0.33 [0.11-0.97]	0.092	0.507
MEIOB	101	7	6.93%	196	5	2.55%	0.35 [0.13-0.94]	0.081	0.507
RMI2	101	5	4.95%	196	4	2.04%	0.40 [0.13-1.23]	0.179	0.535

Symbol	CINSARC Low			CINSARC High			CINSARC High vs. Low		
	N tested	N altered	Fq alteration	N tested	N altered	Fq alteration	OR [95%CI]	p-value*	q-value
BRICD5	101	6	5.94%	196	5	2.55%	0.41 [0.15-1.15]	0.154	0.535
C16orf82	101	6	5.94%	196	3	1.53%	0.25 [0.076-0.80]	0.051	0.507
KNOP1	101	5	4.95%	196	4	2.04%	0.40 [0.13-1.23]	0.179	0.535
C16orf89	101	5	4.95%	196	5	2.55%	0.50 [0.17-1.45]	0.286	0.650
C16orf90	101	5	4.95%	196	4	2.04%	0.40 [0.13-1.23]	0.179	0.535
C16orf91	101	6	5.94%	196	5	2.55%	0.41 [0.15-1.15]	0.154	0.535
C16orf92	101	5	4.95%	196	4	2.04%	0.40 [0.13-1.23]	0.179	0.535
OGFOD3	101	2	1.98%	196	13	6.63%	3.52 [0.99-12.47]	0.102	0.507
TEN1	101	4	3.96%	196	12	6.12%	1.58 [0.60-4.18]	0.438	0.798
LYRM9	101	4	3.96%	196	6	3.06%	0.77 [0.26-2.26]	0.685	0.943
HID1	101	5	4.95%	196	13	6.63%	1.36 [0.56-3.32]	0.566	0.852
MIEN1	101	5	4.95%	196	10	5.10%	1.03 [0.41-2.60]	0.955	1.000
TEFM	101	2	1.98%	196	3	1.53%	0.77 [0.17-3.50]	0.776	0.996
sept-04	101	7	6.93%	196	28	14.29%	2.24 [1.08-4.63]	0.068	0.507
C17orf50	101	2	1.98%	196	4	2.04%	1.03 [0.24-4.35]	0.972	1.000
LINC00469	101	7	6.93%	196	14	7.14%	1.03 [0.47-2.28]	0.946	1.000
LINC00482	101	2	1.98%	196	12	6.12%	3.23 [0.90-11.53]	0.130	0.535
EFCAB13	101	1	0.99%	196	7	3.57%	3.70 [0.63-21.75]	0.224	0.596
C17orf58	101	10	9.90%	196	28	14.29%	1.52 [0.80-2.88]	0.286	0.650
MILR1	101	7	6.93%	196	27	13.78%	2.15 [1.03-4.45]	0.085	0.507
FAM222B	101	3	2.97%	196	9	4.59%	1.57 [0.52-4.80]	0.505	0.805
C17orf64	101	8	7.92%	196	34	17.35%	2.44 [1.23-4.82]	3.12E-02	0.507
C17orf67	101	8	7.92%	196	21	10.71%	1.39 [0.68-2.85]	0.444	0.805
SMG8	101	8	7.92%	196	29	14.80%	2.02 [1.01-4.03]	0.094	0.507
C17orf77	101	6	5.94%	196	13	6.63%	1.12 [0.49-2.60]	0.817	1.000
C17orf78	101	2	1.98%	196	4	2.04%	1.03 [0.24-4.35]	0.972	1.000
C17orf80	101	5	4.95%	196	17	8.67%	1.82 [0.77-4.32]	0.252	0.623
C17orf82	101	7	6.93%	196	32	16.33%	2.62 [1.28-5.38]	2.75E-02	0.507
OXLD1	101	2	1.98%	196	12	6.12%	3.23 [0.90-11.53]	0.130	0.535
PRAC2	101	2	1.98%	196	17	8.67%	4.70 [1.35-16.35]	4.11E-02	0.507
METTL23	101	3	2.97%	196	13	6.63%	2.32 [0.79-6.79]	0.197	0.570
C17orf98	101	4	3.96%	196	10	5.10%	1.30 [0.48-3.53]	0.661	0.935
C17orf99	101	3	2.97%	196	17	8.67%	3.10 [1.09-8.87]	0.076	0.507
LINC00305	101	2	1.98%	196	3	1.53%	0.77 [0.17-3.50]	0.776	0.996
DYNAP	101	2	1.98%	196	3	1.53%	0.77 [0.17-3.50]	0.776	0.996
C18orf54	101	2	1.98%	196	3	1.53%	0.77 [0.17-3.50]	0.776	0.996
C1QTNF1	101	3	2.97%	196	13	6.63%	2.32 [0.79-6.79]	0.197	0.570
C1QTNF8	101	6	5.94%	196	5	2.55%	0.41 [0.15-1.15]	0.154	0.535
C1orf100	101	5	4.95%	196	19	9.69%	2.06 [0.88-4.83]	0.163	0.535
RUSC1-AS1	101	7	6.93%	196	14	7.14%	1.03 [0.47-2.28]	0.946	1.000
C1orf105	101	5	4.95%	196	14	7.14%	1.48 [0.61-3.57]	0.467	0.805
C1orf112	101	4	3.96%	196	12	6.12%	1.58 [0.60-4.18]	0.438	0.798
CCDC181	101	5	4.95%	196	13	6.63%	1.36 [0.56-3.32]	0.566	0.852
C1orf115	101	6	5.94%	196	18	9.18%	1.60 [0.72-3.57]	0.335	0.716
C1orf116	101	6	5.94%	196	24	12.24%	2.21 [1.01-4.82]	0.095	0.507
SPRTN	101	6	5.94%	196	19	9.69%	1.70 [0.77-3.78]	0.274	0.643
AXDND1	101	5	4.95%	196	19	9.69%	2.06 [0.88-4.83]	0.163	0.535
MROH9	101	5	4.95%	196	13	6.63%	1.36 [0.56-3.32]	0.566	0.852
C1orf131	101	6	5.94%	196	19	9.69%	1.70 [0.77-3.78]	0.274	0.643
SERTAD4-AS1	101	6	5.94%	196	21	10.71%	1.90 [0.86-4.19]	0.181	0.535
SHCBP1L	101	5	4.95%	196	18	9.18%	1.94 [0.82-4.58]	0.203	0.570
GCSAML	101	5	4.95%	196	19	9.69%	2.06 [0.88-4.83]	0.163	0.535
PFN1P2	101	1	0.99%	196	7	3.57%	3.70 [0.63-21.75]	0.224	0.596
METTL18	101	4	3.96%	196	13	6.63%	1.72 [0.66-4.51]	0.353	0.722
LINC00303	101	7	6.93%	196	28	14.29%	2.24 [1.08-4.63]	0.068	0.507
TSACC	101	5	4.95%	196	9	4.59%	0.92 [0.36-2.37]	0.890	1.000
C1orf189	101	7	6.93%	196	12	6.12%	0.88 [0.39-1.97]	0.788	0.996
C1orf198	101	6	5.94%	196	19	9.69%	1.70 [0.77-3.78]	0.274	0.643
C1orf21	101	5	4.95%	196	19	9.69%	2.06 [0.88-4.83]	0.163	0.535
C1orf220	101	5	4.95%	196	17	8.67%	1.82 [0.77-4.32]	0.252	0.623
C1orf226	101	5	4.95%	196	18	9.18%	1.94 [0.82-4.58]	0.203	0.570
C1orf229	101	5	4.95%	196	19	9.69%	2.06 [0.88-4.83]	0.163	0.535
RIIAD1	101	6	5.94%	196	12	6.12%	1.03 [0.44-2.41]	0.950	1.000
TRMT1L	101	5	4.95%	196	18	9.18%	1.94 [0.82-4.58]	0.203	0.570
SWT1	101	5	4.95%	196	19	9.69%	2.06 [0.88-4.83]	0.163	0.535
COA6	101	7	6.93%	196	20	10.20%	1.53 [0.72-3.24]	0.355	0.722
C1orf35	101	6	5.94%	196	21	10.71%	1.90 [0.86-4.19]	0.181	0.535
C1orf43	101	7	6.93%	196	12	6.12%	0.88 [0.39-1.97]	0.788	0.996
TEX35	101	5	4.95%	196	17	8.67%	1.82 [0.77-4.32]	0.252	0.623
C1orf53	101	5	4.95%	196	23	11.73%	2.55 [1.10-5.90]	0.066	0.507
C1orf54	101	8	7.92%	196	15	7.65%	0.96 [0.46-2.04]	0.935	1.000

Symbol	CINSARC Low			CINSARC High			CINSARC High vs. Low		
	N tested	N altered	Fq alteration	N tested	N altered	Fq alteration	OR [95%CI]	p-value*	q-value
SDE2	101	6	5.94%	196	19	9.69%	1.70 [0.77-3.78]	0.274	0.643
C1orf56	101	7	6.93%	196	14	7.14%	1.03 [0.47-2.28]	0.946	1.000
NTPCR	101	6	5.94%	196	20	10.20%	1.80 [0.81-3.98]	0.224	0.596
BROX	101	6	5.94%	196	19	9.69%	1.70 [0.77-3.78]	0.274	0.643
C1orf61	101	5	4.95%	196	9	4.59%	0.92 [0.36-2.37]	0.890	1.000
RRNAD1	101	7	6.93%	196	8	4.08%	0.57 [0.24-1.37]	0.294	0.650
C1orf68	101	7	6.93%	196	13	6.63%	0.95 [0.43-2.12]	0.923	1.000
IBA57	101	6	5.94%	196	21	10.71%	1.90 [0.86-4.19]	0.181	0.535
C1orf74	101	6	5.94%	196	22	11.22%	2.00 [0.91-4.39]	0.146	0.535
CHTOP	101	7	6.93%	196	12	6.12%	0.88 [0.39-1.97]	0.788	0.996
LRRC71	101	6	5.94%	196	8	4.08%	0.67 [0.27-1.68]	0.476	0.805
CCSAP	101	6	5.94%	196	19	9.69%	1.70 [0.77-3.78]	0.274	0.643
LINC00467	101	7	6.93%	196	20	10.20%	1.53 [0.72-3.24]	0.355	0.722
SUCO	101	5	4.95%	196	15	7.65%	1.59 [0.66-3.81]	0.382	0.750
FAM209A	101	7	6.93%	196	14	7.14%	1.03 [0.47-2.28]	0.946	1.000
FAM209B	101	7	6.93%	196	14	7.14%	1.03 [0.47-2.28]	0.946	1.000
FAM210B	101	7	6.93%	196	11	5.61%	0.80 [0.35-1.82]	0.652	0.928
OSER1	101	3	2.97%	196	3	1.53%	0.51 [0.13-1.98]	0.412	0.774
SOGA1	101	4	3.96%	196	6	3.06%	0.77 [0.26-2.26]	0.685	0.943
TLDC2	101	3	2.97%	196	6	3.06%	1.03 [0.32-3.36]	0.965	1.000
GID8	101	7	6.93%	196	14	7.14%	1.03 [0.47-2.28]	0.946	1.000
OCSTAMP	101	4	3.96%	196	7	3.57%	0.90 [0.31-2.57]	0.867	1.000
MROH8	101	4	3.96%	196	3	1.53%	0.38 [0.11-1.35]	0.207	0.573
ABHD16B	101	6	5.94%	196	12	6.12%	1.03 [0.44-2.41]	0.950	1.000
RBBP8NL	101	6	5.94%	196	11	5.61%	0.94 [0.40-2.23]	0.908	1.000
CNBD2	101	4	3.96%	196	8	4.08%	1.03 [0.37-2.88]	0.960	1.000
SPATA25	101	5	4.95%	196	4	2.04%	0.40 [0.13-1.23]	0.179	0.535
C20orf173	101	4	3.96%	196	8	4.08%	1.03 [0.37-2.88]	0.960	1.000
FAM217B	101	10	9.90%	196	13	6.63%	0.65 [0.31-1.33]	0.321	0.700
C20orf197	101	11	10.89%	196	14	7.14%	0.63 [0.31-1.26]	0.274	0.643
ZFAS1	101	7	6.93%	196	7	3.57%	0.50 [0.20-1.23]	0.203	0.570
MRGBP	101	7	6.93%	196	14	7.14%	1.03 [0.47-2.28]	0.946	1.000
AAR2	101	4	3.96%	196	7	3.57%	0.90 [0.31-2.57]	0.867	1.000
C20orf85	101	8	7.92%	196	17	8.67%	1.10 [0.53-2.30]	0.825	1.000
C2CD3	101	7	6.93%	196	12	6.12%	0.88 [0.39-1.97]	0.788	0.996
C2CD4D	101	6	5.94%	196	12	6.12%	1.03 [0.44-2.41]	0.950	1.000
ERGIC3	101	4	3.96%	196	8	4.08%	1.03 [0.37-2.88]	0.960	1.000
XXYLT1	101	3	2.97%	196	3	1.53%	0.51 [0.13-1.98]	0.412	0.774
CEP19	101	3	2.97%	196	5	2.55%	0.86 [0.25-2.89]	0.833	1.000
MB21D2	101	3	2.97%	196	3	1.53%	0.51 [0.13-1.98]	0.412	0.774
C3orf70	101	3	2.97%	196	2	1.02%	0.34 [0.074-1.53]	0.237	0.599
C4BPA	101	6	5.94%	196	24	12.24%	2.21 [1.01-4.82]	0.095	0.507
C4BPB	101	6	5.94%	196	24	12.24%	2.21 [1.01-4.82]	0.095	0.507
GFOD1	101	1	0.99%	196	4	2.04%	2.08 [0.33-13.25]	0.514	0.805
C6orf203	101	1	0.99%	196	9	4.59%	4.81 [0.84-27.58]	0.139	0.535
CCDC170	101	1	0.99%	196	4	2.04%	2.08 [0.33-13.25]	0.514	0.805
PPP1R17	101	0	0.00%	196	8	4.08%	3.6e+07 [0e+00-Inf]	0.992	1.000
MALSU1	101	0	0.00%	196	6	3.06%	2.7e+07 [0e+00-Inf]	0.992	1.000
C7orf31	101	0	0.00%	196	7	3.57%	3.2e+07 [0e+00-Inf]	0.992	1.000
MTURN	101	0	0.00%	196	6	3.06%	2.7e+07 [0e+00-Inf]	0.992	1.000
FAM221A	101	0	0.00%	196	6	3.06%	2.7e+07 [0e+00-Inf]	0.992	1.000
C7orf71	101	0	0.00%	196	5	2.55%	2.2e+07 [0e+00-Inf]	0.992	1.000
TMEM249	101	18	17.82%	196	49	25.00%	1.54 [0.93-2.55]	0.163	0.535
C8orf31	101	16	15.84%	196	49	25.00%	1.77 [1.05-2.99]	0.073	0.507
C8orf33	101	16	15.84%	196	48	24.49%	1.72 [1.02-2.91]	0.088	0.507
C8orf34	101	14	13.86%	196	37	18.88%	1.45 [0.83-2.53]	0.279	0.650
C8orf37	101	16	15.84%	196	54	27.55%	2.02 [1.20-3.40]	2.60E-02	0.507
NDUFAF6	101	16	15.84%	196	54	27.55%	2.02 [1.20-3.40]	2.60E-02	0.507
RBM12B-AS1	101	17	16.83%	196	54	27.55%	1.88 [1.13-3.13]	4.21E-02	0.507
SMIM19	101	16	15.84%	196	30	15.31%	0.96 [0.55-1.67]	0.904	1.000
TTI2	101	4	3.96%	196	10	5.10%	1.30 [0.48-3.53]	0.661	0.935
C8orf44	101	15	14.85%	196	38	19.39%	1.38 [0.80-2.38]	0.335	0.716
MCMDC2	101	15	14.85%	196	39	19.90%	1.42 [0.82-2.46]	0.287	0.650
RHPN1-AS1	101	17	16.83%	196	51	26.02%	1.74 [1.04-2.90]	0.076	0.507
THEM6	101	16	15.84%	196	49	25.00%	1.77 [1.05-2.99]	0.073	0.507
LINC00588	101	14	13.86%	196	29	14.80%	1.08 [0.61-1.92]	0.828	1.000
MROH6	101	17	16.83%	196	51	26.02%	1.74 [1.04-2.90]	0.076	0.507
C8orf76	101	14	13.86%	196	57	29.08%	2.55 [1.49-4.37]	4.36E-03	0.507
ZNF252P-AS1	101	16	15.84%	196	47	23.98%	1.68 [0.99-2.84]	0.106	0.507
TRIQK	101	16	15.84%	196	54	27.55%	2.02 [1.20-3.40]	2.60E-02	0.507
SBSPON	101	16	15.84%	196	42	21.43%	1.45 [0.85-2.47]	0.252	0.623

Symbol	CINSARC Low			CINSARC High			CINSARC High vs. Low		
	N tested	N altered	Fq alteration	N tested	N altered	Fq alteration	OR [95%CI]	p-value*	q-value
AARD	101	16	15.84%	196	57	29.08%	2.18 [1.30-3.66]	1.33E-02	0.507
C8orf86	101	11	10.89%	196	43	21.94%	2.30 [1.27-4.18]	2.18E-02	0.507
C9orf131	101	1	0.99%	196	4	2.04%	2.08 [0.33-13.25]	0.514	0.805
FAM205A	101	1	0.99%	196	4	2.04%	2.08 [0.33-13.25]	0.514	0.805
RPP25L	101	1	0.99%	196	4	2.04%	2.08 [0.33-13.25]	0.514	0.805
CA10	101	4	3.96%	196	12	6.12%	1.58 [0.60-4.18]	0.438	0.798
CA13	101	17	16.83%	196	47	23.98%	1.56 [0.93-2.61]	0.158	0.535
CA14	101	8	7.92%	196	15	7.65%	0.96 [0.46-2.04]	0.935	1.000
CA1	101	17	16.83%	196	47	23.98%	1.56 [0.93-2.61]	0.158	0.535
CA2	101	17	16.83%	196	48	24.49%	1.60 [0.96-2.68]	0.133	0.535
CA3	101	17	16.83%	196	48	24.49%	1.60 [0.96-2.68]	0.133	0.535
CA4	101	7	6.93%	196	33	16.84%	2.72 [1.33-5.57]	2.17E-02	0.507
CA8	101	16	15.84%	196	33	16.84%	1.08 [0.62-1.86]	0.827	1.000
CABLES2	101	6	5.94%	196	11	5.61%	0.94 [0.40-2.23]	0.908	1.000
CABP2	101	11	10.89%	196	20	10.20%	0.93 [0.48-1.79]	0.854	1.000
CABP4	101	11	10.89%	196	20	10.20%	0.93 [0.48-1.79]	0.854	1.000
CACNA1E	101	5	4.95%	196	18	9.18%	1.94 [0.82-4.58]	0.203	0.570
CACNA1G	101	4	3.96%	196	19	9.69%	2.60 [1.03-6.59]	0.090	0.507
CACNA1H	101	6	5.94%	196	5	2.55%	0.41 [0.15-1.15]	0.154	0.535
CACNA1S	101	6	5.94%	196	24	12.24%	2.21 [1.01-4.82]	0.095	0.507
CACNB1	101	4	3.96%	196	12	6.12%	1.58 [0.60-4.18]	0.438	0.798
CACNG1	101	10	9.90%	196	29	14.80%	1.58 [0.83-3.00]	0.240	0.606
CACNG3	101	5	4.95%	196	3	1.53%	0.30 [0.088-1.01]	0.103	0.507
CACNG4	101	9	8.91%	196	29	14.80%	1.78 [0.91-3.44]	0.155	0.535
CACNG5	101	9	8.91%	196	30	15.31%	1.85 [0.95-3.58]	0.126	0.535
CACYBP	101	5	4.95%	196	16	8.16%	1.71 [0.72-4.07]	0.311	0.680
CADM3	101	6	5.94%	196	8	4.08%	0.67 [0.27-1.68]	0.476	0.805
CALB1	101	18	17.82%	196	48	24.49%	1.50 [0.90-2.48]	0.192	0.563
CALCA	101	1	0.99%	196	4	2.04%	2.08 [0.33-13.25]	0.514	0.805
CALCB	101	1	0.99%	196	4	2.04%	2.08 [0.33-13.25]	0.514	0.805
CALCOCO2	101	2	1.98%	196	18	9.18%	5.01 [1.44-17.35]	3.31E-02	0.507
CAMK1G	101	6	5.94%	196	23	11.73%	2.11 [0.96-4.60]	0.118	0.526
CAMK2B	101	0	0.00%	196	5	2.55%	2.2e+07 [0e+00-Inf]	0.992	1.000
CAMK2G	101	0	0.00%	196	5	2.55%	2.2e+07 [0e+00-Inf]	0.992	1.000
CAMK2N2	101	3	2.97%	196	2	1.02%	0.34 [0.074-1.53]	0.237	0.599
CAMSAP2	101	6	5.94%	196	24	12.24%	2.21 [1.01-4.82]	0.095	0.507
CAND1	101	4	3.96%	196	14	7.14%	1.87 [0.72-4.85]	0.283	0.650
CANT1	101	3	2.97%	196	13	6.63%	2.32 [0.79-6.79]	0.197	0.570
CAPN2	101	6	5.94%	196	18	9.18%	1.60 [0.72-3.57]	0.335	0.716
CAPN5	101	7	6.93%	196	19	9.69%	1.44 [0.68-3.07]	0.427	0.791
CAPN8	101	6	5.94%	196	18	9.18%	1.60 [0.72-3.57]	0.335	0.716
CAPN9	101	6	5.94%	196	19	9.69%	1.70 [0.77-3.78]	0.274	0.643
CAPS2	101	2	1.98%	196	7	3.57%	1.83 [0.48-6.96]	0.455	0.805
CARD14	101	4	3.96%	196	14	7.14%	1.87 [0.72-4.85]	0.283	0.650
CARHSP1	101	6	5.94%	196	4	2.04%	0.33 [0.11-0.97]	0.092	0.507
CARNS1	101	11	10.89%	196	20	10.20%	0.93 [0.48-1.79]	0.854	1.000
CASC3	101	5	4.95%	196	9	4.59%	0.92 [0.36-2.37]	0.890	1.000
CASKIN1	101	6	5.94%	196	5	2.55%	0.41 [0.15-1.15]	0.154	0.535
CASKIN2	101	5	4.95%	196	12	6.12%	1.25 [0.51-3.08]	0.681	0.943
CASQ1	101	6	5.94%	196	12	6.12%	1.03 [0.44-2.41]	0.950	1.000
CASS4	101	8	7.92%	196	14	7.14%	0.89 [0.42-1.91]	0.808	1.000
CATSPER1	101	0	0.00%	196	5	2.55%	2.2e+07 [0e+00-Inf]	0.992	1.000
CBLN4	101	6	5.94%	196	10	5.10%	0.85 [0.36-2.04]	0.762	0.996
CBX1	101	1	0.99%	196	13	6.63%	7.10 [1.27-39.64]	0.061	0.507
CBX2	101	4	3.96%	196	18	9.18%	2.45 [0.97-6.23]	0.114	0.512
CBX3	101	0	0.00%	196	5	2.55%	2.2e+07 [0e+00-Inf]	0.992	1.000
CBX4	101	3	2.97%	196	18	9.18%	3.30 [1.16-9.41]	0.060	0.507
CBX8	101	4	3.96%	196	20	10.20%	2.76 [1.09-6.95]	0.071	0.507
CCBE1	101	1	0.99%	196	4	2.04%	2.08 [0.33-13.25]	0.514	0.805
CCDC106	101	2	1.98%	196	4	2.04%	1.03 [0.24-4.35]	0.972	1.000
MCU	101	1	0.99%	196	4	2.04%	2.08 [0.33-13.25]	0.514	0.805
CCDC126	101	0	0.00%	196	6	3.06%	2.7e+07 [0e+00-Inf]	0.992	1.000
CCDC137	101	2	1.98%	196	12	6.12%	3.23 [0.90-11.53]	0.130	0.535
CCDC144B	101	3	2.97%	196	2	1.02%	0.34 [0.074-1.53]	0.237	0.599
CCDC154	101	6	5.94%	196	5	2.55%	0.41 [0.15-1.15]	0.154	0.535
CCDC158	101	2	1.98%	196	5	2.55%	1.30 [0.32-5.21]	0.759	0.996
CCDC39	101	4	3.96%	196	2	1.02%	0.25 [0.059-1.05]	0.113	0.511
CCDC40	101	3	2.97%	196	16	8.16%	2.90 [1.01-8.34]	0.097	0.507
CEP95	101	8	7.92%	196	27	13.78%	1.86 [0.93-3.72]	0.143	0.535
CEP112	101	9	8.91%	196	25	12.76%	1.49 [0.76-2.93]	0.327	0.712
CCDC47	101	8	7.92%	196	29	14.80%	2.02 [1.01-4.03]	0.094	0.507

Symbol	CINSARC Low			CINSARC High			CINSARC High vs. Low		
	N tested	N altered	Fq alteration	N tested	N altered	Fq alteration	OR [95%CI]	p-value*	q-value
CCDC50	101	3	2.97%	196	3	1.53%	0.51 [0.13-1.98]	0.412	0.774
NSRP1	101	2	1.98%	196	4	2.04%	1.03 [0.24-4.35]	0.972	1.000
CCDC57	101	2	1.98%	196	13	6.63%	3.52 [0.99-12.47]	0.102	0.507
CCDC68	101	2	1.98%	196	3	1.53%	0.77 [0.17-3.50]	0.776	0.996
CCDC78	101	6	5.94%	196	4	2.04%	0.33 [0.11-0.97]	0.092	0.507
CCDC81	101	1	0.99%	196	4	2.04%	2.08 [0.33-13.25]	0.514	0.805
CCDC83	101	2	1.98%	196	3	1.53%	0.77 [0.17-3.50]	0.776	0.996
CCDC87	101	5	4.95%	196	14	7.14%	1.48 [0.61-3.57]	0.467	0.805
CCDC89	101	2	1.98%	196	3	1.53%	0.77 [0.17-3.50]	0.776	0.996
CCL14	101	2	1.98%	196	4	2.04%	1.03 [0.24-4.35]	0.972	1.000
CCL15	101	2	1.98%	196	4	2.04%	1.03 [0.24-4.35]	0.972	1.000
CCL16	101	2	1.98%	196	4	2.04%	1.03 [0.24-4.35]	0.972	1.000
CCL19	101	1	0.99%	196	4	2.04%	2.08 [0.33-13.25]	0.514	0.805
CCL21	101	1	0.99%	196	4	2.04%	2.08 [0.33-13.25]	0.514	0.805
CCL23	101	2	1.98%	196	5	2.55%	1.30 [0.32-5.21]	0.759	0.996
CCL27	101	1	0.99%	196	4	2.04%	2.08 [0.33-13.25]	0.514	0.805
CCL3L1	101	3	2.97%	196	10	5.10%	1.76 [0.58-5.29]	0.401	0.774
CCL3L3	101	3	2.97%	196	10	5.10%	1.76 [0.58-5.29]	0.401	0.774
CCL3	101	2	1.98%	196	5	2.55%	1.30 [0.32-5.21]	0.759	0.996
CCL4L2	101	3	2.97%	196	11	5.61%	1.94 [0.65-5.78]	0.317	0.692
CCL4	101	2	1.98%	196	7	3.57%	1.83 [0.48-6.96]	0.455	0.805
CCL5	101	2	1.98%	196	5	2.55%	1.30 [0.32-5.21]	0.759	0.996
CCND1	101	25	24.75%	196	63	32.14%	1.44 [0.91-2.27]	0.188	0.553
CCNE2	101	16	15.84%	196	54	27.55%	2.02 [1.20-3.40]	2.60E-02	0.507
CCNF	101	6	5.94%	196	5	2.55%	0.41 [0.15-1.15]	0.154	0.535
CCR7	101	4	3.96%	196	5	2.55%	0.63 [0.21-1.95]	0.505	0.805
CCS	101	6	5.94%	196	14	7.14%	1.22 [0.53-2.79]	0.696	0.951
CCT2	101	2	1.98%	196	17	8.67%	4.70 [1.35-16.35]	4.11E-02	0.507
CCT3	101	5	4.95%	196	9	4.59%	0.92 [0.36-2.37]	0.890	1.000
CCT6B	101	1	0.99%	196	5	2.55%	2.62 [0.43-16.05]	0.383	0.750
CD160	101	5	4.95%	196	10	5.10%	1.03 [0.41-2.60]	0.955	1.000
CD19	101	6	5.94%	196	3	1.53%	0.25 [0.076-0.80]	0.051	0.507
CD1A	101	7	6.93%	196	10	5.10%	0.72 [0.31-1.67]	0.522	0.815
CD1B	101	7	6.93%	196	10	5.10%	0.72 [0.31-1.67]	0.522	0.815
CD1C	101	7	6.93%	196	9	4.59%	0.65 [0.27-1.52]	0.401	0.774
CD1D	101	7	6.93%	196	9	4.59%	0.65 [0.27-1.52]	0.401	0.774
CD1E	101	7	6.93%	196	10	5.10%	0.72 [0.31-1.67]	0.522	0.815
CD244	101	6	5.94%	196	16	8.16%	1.41 [0.62-3.18]	0.490	0.805
CD247	101	5	4.95%	196	12	6.12%	1.25 [0.51-3.08]	0.681	0.943
CD248	101	3	2.97%	196	6	3.06%	1.03 [0.32-3.36]	0.965	1.000
CD24	101	0	0.00%	196	0	0.00%	1.00 [0e+00-Inf]	1.000	1.000
CD2BP2	101	4	3.96%	196	5	2.55%	0.63 [0.21-1.95]	0.505	0.805
CD300A	101	7	6.93%	196	13	6.63%	0.95 [0.43-2.12]	0.923	1.000
CD300C	101	6	5.94%	196	13	6.63%	1.12 [0.49-2.60]	0.817	1.000
CD300E	101	6	5.94%	196	14	7.14%	1.22 [0.53-2.79]	0.696	0.951
CD300LB	101	6	5.94%	196	13	6.63%	1.12 [0.49-2.60]	0.817	1.000
CD300LD	101	6	5.94%	196	13	6.63%	1.12 [0.49-2.60]	0.817	1.000
CD300LF	101	5	4.95%	196	13	6.63%	1.36 [0.56-3.32]	0.566	0.852
CD34	101	7	6.93%	196	25	12.76%	1.96 [0.94-4.09]	0.131	0.535
CD40	101	5	4.95%	196	5	2.55%	0.50 [0.17-1.45]	0.286	0.650
CD44	101	1	0.99%	196	5	2.55%	2.62 [0.43-16.05]	0.383	0.750
CD46	101	7	6.93%	196	25	12.76%	1.96 [0.94-4.09]	0.131	0.535
CD48	101	6	5.94%	196	15	7.65%	1.31 [0.58-2.98]	0.586	0.869
CD55	101	6	5.94%	196	24	12.24%	2.21 [1.01-4.82]	0.095	0.507
CD59	101	1	0.99%	196	4	2.04%	2.08 [0.33-13.25]	0.514	0.805
CD5L	101	7	6.93%	196	9	4.59%	0.65 [0.27-1.52]	0.401	0.774
CD5	101	2	1.98%	196	3	1.53%	0.77 [0.17-3.50]	0.776	0.996
CD79B	101	8	7.92%	196	29	14.80%	2.02 [1.01-4.03]	0.094	0.507
CD7	101	2	1.98%	196	14	7.14%	3.81 [1.08-13.43]	0.081	0.507
CD84	101	5	4.95%	196	14	7.14%	1.48 [0.61-3.57]	0.467	0.805
CDC27	101	1	0.99%	196	5	2.55%	2.62 [0.43-16.05]	0.383	0.750
CDC42BPA	101	6	5.94%	196	20	10.20%	1.80 [0.81-3.98]	0.224	0.596
CDC42EP4	101	5	4.95%	196	17	8.67%	1.82 [0.77-4.32]	0.252	0.623
CDC42SE1	101	7	6.93%	196	14	7.14%	1.03 [0.47-2.28]	0.946	1.000
CDC6	101	4	3.96%	196	7	3.57%	0.90 [0.31-2.57]	0.867	1.000
CDC73	101	5	4.95%	196	18	9.18%	1.94 [0.82-4.58]	0.203	0.570
CDCA7L	101	0	0.00%	196	6	3.06%	2.7e+07 [0e+00-Inf]	0.992	1.000
CDH17	101	17	16.83%	196	52	26.53%	1.78 [1.07-2.98]	0.063	0.507
CDH20	101	2	1.98%	196	3	1.53%	0.77 [0.17-3.50]	0.776	0.996
CDH22	101	4	3.96%	196	5	2.55%	0.63 [0.21-1.95]	0.505	0.805
CDH24	101	1	0.99%	196	4	2.04%	2.08 [0.33-13.25]	0.514	0.805

Symbol	CINSARC Low			CINSARC High			CINSARC High vs. Low		
	N tested	N altered	Fq alteration	N tested	N altered	Fq alteration	OR [95%CI]	p-value*	q-value
CDH26	101	10	9.90%	196	13	6.63%	0.65 [0.31-1.33]	0.321	0.700
CDH4	101	7	6.93%	196	12	6.12%	0.88 [0.39-1.97]	0.788	0.996
CDIPT	101	5	4.95%	196	4	2.04%	0.40 [0.13-1.23]	0.179	0.535
CDK12	101	6	5.94%	196	13	6.63%	1.12 [0.49-2.60]	0.817	1.000
CDK18	101	7	6.93%	196	26	13.27%	2.05 [0.99-4.27]	0.106	0.507
CDK2AP2	101	11	10.89%	196	20	10.20%	0.93 [0.48-1.79]	0.854	1.000
CDK3	101	4	3.96%	196	12	6.12%	1.58 [0.60-4.18]	0.438	0.798
CDK4	101	0	0.00%	196	5	2.55%	2.2e+07 [0e+00-Inf]	0.992	1.000
CDK5R1	101	2	1.98%	196	5	2.55%	1.30 [0.32-5.21]	0.759	0.996
CDK5RAP3	101	1	0.99%	196	9	4.59%	4.81 [0.84-27.58]	0.139	0.535
CDKL2	101	1	0.99%	196	5	2.55%	2.62 [0.43-16.05]	0.383	0.750
CDR2L	101	5	4.95%	196	13	6.63%	1.36 [0.56-3.32]	0.566	0.852
CDR2	101	5	4.95%	196	3	1.53%	0.30 [0.088-1.01]	0.103	0.507
CDY1B	101	0	0.00%	196	0	0.00%	1.00 [0e+00-Inf]	1.000	1.000
CDY1	101	0	0.00%	196	0	0.00%	1.00 [0e+00-Inf]	1.000	1.000
CDY2B	101	0	0.00%	196	0	0.00%	1.00 [0e+00-Inf]	1.000	1.000
CEBPB	101	6	5.94%	196	14	7.14%	1.22 [0.53-2.79]	0.696	0.951
CEBPD	101	14	13.86%	196	28	14.29%	1.04 [0.58-1.85]	0.921	1.000
CELF3	101	6	5.94%	196	12	6.12%	1.03 [0.44-2.41]	0.950	1.000
CELF6	101	1	0.99%	196	4	2.04%	2.08 [0.33-13.25]	0.514	0.805
CEMP1	101	6	5.94%	196	5	2.55%	0.41 [0.15-1.15]	0.154	0.535
CENPF	101	6	5.94%	196	20	10.20%	1.80 [0.81-3.98]	0.224	0.596
CENPL	101	5	4.95%	196	13	6.63%	1.36 [0.56-3.32]	0.566	0.852
CEP170	101	5	4.95%	196	19	9.69%	2.06 [0.88-4.83]	0.163	0.535
CEP250	101	4	3.96%	196	8	4.08%	1.03 [0.37-2.88]	0.960	1.000
CEP290	101	0	0.00%	196	5	2.55%	2.2e+07 [0e+00-Inf]	0.992	1.000
CEP350	101	6	5.94%	196	19	9.69%	1.70 [0.77-3.78]	0.274	0.643
CFHR1	101	7	6.93%	196	24	12.24%	1.87 [0.90-3.92]	0.161	0.535
CFHR2	101	6	5.94%	196	20	10.20%	1.80 [0.81-3.98]	0.224	0.596
CFHR3	101	7	6.93%	196	27	13.78%	2.15 [1.03-4.45]	0.085	0.507
CFHR4	101	4	3.96%	196	19	9.69%	2.60 [1.03-6.59]	0.090	0.507
CFHR5	101	5	4.95%	196	21	10.71%	2.30 [0.99-5.36]	0.104	0.507
CFH	101	4	3.96%	196	21	10.71%	2.91 [1.16-7.31]	0.056	0.507
CFL2	101	0	0.00%	196	9	4.59%	4.1e+07 [0e+00-Inf]	0.992	1.000
CGN	101	6	5.94%	196	13	6.63%	1.12 [0.49-2.60]	0.817	1.000
CHAD	101	4	3.96%	196	20	10.20%	2.76 [1.09-6.95]	0.071	0.507
CHCHD1	101	0	0.00%	196	5	2.55%	2.2e+07 [0e+00-Inf]	0.992	1.000
CHCHD7	101	14	13.86%	196	31	15.82%	1.17 [0.66-2.07]	0.656	0.931
COA4	101	7	6.93%	196	12	6.12%	0.88 [0.39-1.97]	0.788	0.996
CHD1L	101	6	5.94%	196	12	6.12%	1.03 [0.44-2.41]	0.950	1.000
CHD2	101	0	0.00%	196	6	3.06%	2.7e+07 [0e+00-Inf]	0.992	1.000
CHD6	101	2	1.98%	196	4	2.04%	1.03 [0.24-4.35]	0.972	1.000
CHD7	101	16	15.84%	196	31	15.82%	1.00 [0.57-1.73]	0.995	1.000
CHI3L1	101	7	6.93%	196	28	14.29%	2.24 [1.08-4.63]	0.068	0.507
CHIT1	101	7	6.93%	196	28	14.29%	2.24 [1.08-4.63]	0.068	0.507
CHKA	101	8	7.92%	196	21	10.71%	1.39 [0.68-2.85]	0.444	0.805
CHML	101	4	3.96%	196	20	10.20%	2.76 [1.09-6.95]	0.071	0.507
CHMP4C	101	18	17.82%	196	46	23.47%	1.41 [0.85-2.35]	0.263	0.643
CHMP6	101	2	1.98%	196	12	6.12%	3.23 [0.90-11.53]	0.130	0.535
CHN2	101	0	0.00%	196	6	3.06%	2.7e+07 [0e+00-Inf]	0.992	1.000
CHORDC1	101	1	0.99%	196	4	2.04%	2.08 [0.33-13.25]	0.514	0.805
CHP2	101	5	4.95%	196	3	1.53%	0.30 [0.088-1.01]	0.103	0.507
CHRAC1	101	17	16.83%	196	50	25.51%	1.69 [1.01-2.83]	0.092	0.507
CHRDL2	101	5	4.95%	196	13	6.63%	1.36 [0.56-3.32]	0.566	0.852
CHRD	101	3	2.97%	196	2	1.02%	0.34 [0.074-1.53]	0.237	0.599
CHRM3	101	5	4.95%	196	20	10.20%	2.18 [0.93-5.10]	0.130	0.535
CHRNA4	101	7	6.93%	196	13	6.63%	0.95 [0.43-2.12]	0.923	1.000
CHRNA6	101	14	13.86%	196	28	14.29%	1.04 [0.58-1.85]	0.921	1.000
CHRNB2	101	7	6.93%	196	12	6.12%	0.88 [0.39-1.97]	0.788	0.996
CHRNB3	101	14	13.86%	196	28	14.29%	1.04 [0.58-1.85]	0.921	1.000
CHTF18	101	6	5.94%	196	4	2.04%	0.33 [0.11-0.97]	0.092	0.507
CIB1	101	2	1.98%	196	8	4.08%	2.11 [0.56-7.86]	0.352	0.722
CIB2	101	1	0.99%	196	4	2.04%	2.08 [0.33-13.25]	0.514	0.805
CIITA	101	5	4.95%	196	4	2.04%	0.40 [0.13-1.23]	0.179	0.535
CISD3	101	5	4.95%	196	9	4.59%	0.92 [0.36-2.37]	0.890	1.000
CKS1B	101	8	7.92%	196	15	7.65%	0.96 [0.46-2.04]	0.935	1.000
CLCF1	101	11	10.89%	196	17	8.67%	0.78 [0.40-1.52]	0.536	0.834
CLCN2	101	3	2.97%	196	2	1.02%	0.34 [0.074-1.53]	0.237	0.599
CLCN7	101	7	6.93%	196	5	2.55%	0.35 [0.13-0.94]	0.081	0.507
CLDN11	101	2	1.98%	196	3	1.53%	0.77 [0.17-3.50]	0.776	0.996
CLDN16	101	2	1.98%	196	3	1.53%	0.77 [0.17-3.50]	0.776	0.996

Symbol	CINSARC Low			CINSARC High			CINSARC High vs. Low		
	N tested	N altered	Fq alteration	N tested	N altered	Fq alteration	OR [95%CI]	p-value*	q-value
CLDN1	101	2	1.98%	196	3	1.53%	0.77 [0.17-3.50]	0.776	0.996
CLDN6	101	6	5.94%	196	5	2.55%	0.41 [0.15-1.15]	0.154	0.535
CLDN9	101	6	5.94%	196	5	2.55%	0.41 [0.15-1.15]	0.154	0.535
CLEC14A	101	0	0.00%	196	8	4.08%	3.6e+07 [0e+00-Inf]	0.992	1.000
CLEC16A	101	5	4.95%	196	4	2.04%	0.40 [0.13-1.23]	0.179	0.535
CLK2	101	7	6.93%	196	14	7.14%	1.03 [0.47-2.28]	0.946	1.000
CLLU1OS	101	0	0.00%	196	5	2.55%	2.2e+07 [0e+00-Inf]	0.992	1.000
CLLU1	101	0	0.00%	196	5	2.55%	2.2e+07 [0e+00-Inf]	0.992	1.000
CLN3	101	5	4.95%	196	3	1.53%	0.30 [0.088-1.01]	0.103	0.507
CLNS1A	101	10	9.90%	196	21	10.71%	1.09 [0.56-2.13]	0.828	1.000
CLPB	101	5	4.95%	196	9	4.59%	0.92 [0.36-2.37]	0.890	1.000
CLRN1-AS1	101	3	2.97%	196	2	1.02%	0.34 [0.074-1.53]	0.237	0.599
CLRN1	101	3	2.97%	196	2	1.02%	0.34 [0.074-1.53]	0.237	0.599
CLTC	101	9	8.91%	196	33	16.84%	2.07 [1.08-3.98]	0.068	0.507
CLUAP1	101	5	4.95%	196	4	2.04%	0.40 [0.13-1.23]	0.179	0.535
CLVS1	101	15	14.85%	196	30	15.31%	1.04 [0.59-1.82]	0.918	1.000
CNBD1	101	16	15.84%	196	47	23.98%	1.68 [0.99-2.84]	0.106	0.507
CNGB3	101	17	16.83%	196	48	24.49%	1.60 [0.96-2.68]	0.133	0.535
CNIH2	101	2	1.98%	196	5	2.55%	1.30 [0.32-5.21]	0.759	0.996
CNIH3	101	6	5.94%	196	17	8.67%	1.50 [0.67-3.38]	0.407	0.774
CNIH4	101	7	6.93%	196	17	8.67%	1.28 [0.59-2.75]	0.602	0.887
CNOT2	101	3	2.97%	196	16	8.16%	2.90 [1.01-8.34]	0.097	0.507
CNST	101	5	4.95%	196	18	9.18%	1.94 [0.82-4.58]	0.203	0.570
CNTFR	101	1	0.99%	196	4	2.04%	2.08 [0.33-13.25]	0.514	0.805
CNTN2	101	7	6.93%	196	26	13.27%	2.05 [0.99-4.27]	0.106	0.507
COCH	101	1	0.99%	196	8	4.08%	4.26 [0.73-24.65]	0.175	0.535
COG1	101	5	4.95%	196	18	9.18%	1.94 [0.82-4.58]	0.203	0.570
COG2	101	6	5.94%	196	19	9.69%	1.70 [0.77-3.78]	0.274	0.643
COG7	101	5	4.95%	196	2	1.02%	0.20 [0.049-0.80]	0.056	0.507
COIL	101	7	6.93%	196	23	11.73%	1.79 [0.85-3.74]	0.198	0.570
COL14A1	101	15	14.85%	196	54	27.55%	2.18 [1.28-3.70]	1.56E-02	0.507
COL1A1	101	5	4.95%	196	17	8.67%	1.82 [0.77-4.32]	0.252	0.623
COL20A1	101	7	6.93%	196	13	6.63%	0.95 [0.43-2.12]	0.923	1.000
COL22A1	101	15	14.85%	196	51	26.02%	2.02 [1.18-3.43]	3.03E-02	0.507
COL9A3	101	7	6.93%	196	14	7.14%	1.03 [0.47-2.28]	0.946	1.000
COLEC10	101	15	14.85%	196	59	30.10%	2.47 [1.46-4.18]	4.76E-03	0.507
COMMD5	101	17	16.83%	196	49	25.00%	1.65 [0.98-2.76]	0.111	0.511
COMTD1	101	2	1.98%	196	6	3.06%	1.56 [0.40-6.08]	0.589	0.869
COPA	101	6	5.94%	196	13	6.63%	1.12 [0.49-2.60]	0.817	1.000
COPS5	101	15	14.85%	196	39	19.90%	1.42 [0.82-2.46]	0.287	0.650
COPZ2	101	1	0.99%	196	12	6.12%	6.52 [1.16-36.57]	0.074	0.507
COQ7	101	5	4.95%	196	4	2.04%	0.40 [0.13-1.23]	0.179	0.535
CORO1A	101	4	3.96%	196	4	2.04%	0.51 [0.16-1.65]	0.342	0.716
CORO1B	101	11	10.89%	196	20	10.20%	0.93 [0.48-1.79]	0.854	1.000
CORO6	101	3	2.97%	196	5	2.55%	0.86 [0.25-2.89]	0.833	1.000
CORO7	101	5	4.95%	196	6	3.06%	0.61 [0.22-1.68]	0.418	0.779
COX11	101	7	6.93%	196	18	9.18%	1.36 [0.63-2.91]	0.509	0.805
COX18	101	1	0.99%	196	6	3.06%	3.16 [0.53-18.88]	0.290	0.650
COX6A2	101	4	3.96%	196	4	2.04%	0.51 [0.16-1.65]	0.342	0.716
COX6B2	101	1	0.99%	196	4	2.04%	2.08 [0.33-13.25]	0.514	0.805
COX6C	101	15	14.85%	196	56	28.57%	2.29 [1.35-3.89]	9.81E-03	0.507
CCP110	101	5	4.95%	196	4	2.04%	0.40 [0.13-1.23]	0.179	0.535
CPA6	101	15	14.85%	196	37	18.88%	1.33 [0.77-2.31]	0.388	0.757
CPD	101	2	1.98%	196	3	1.53%	0.77 [0.17-3.50]	0.776	0.996
CPEB1	101	1	0.99%	196	4	2.04%	2.08 [0.33-13.25]	0.514	0.805
CPLX4	101	1	0.99%	196	4	2.04%	2.08 [0.33-13.25]	0.514	0.805
CPM	101	2	1.98%	196	19	9.69%	5.31 [1.54-18.36]	2.67E-02	0.507
CPN2	101	3	2.97%	196	3	1.53%	0.51 [0.13-1.98]	0.412	0.774
CPNE1	101	4	3.96%	196	8	4.08%	1.03 [0.37-2.88]	0.960	1.000
CPNE3	101	18	17.82%	196	48	24.49%	1.50 [0.90-2.48]	0.192	0.563
CPPED1	101	5	4.95%	196	3	1.53%	0.30 [0.088-1.01]	0.103	0.507
CPSF1	101	18	17.82%	196	49	25.00%	1.54 [0.93-2.55]	0.163	0.535
CPSF4L	101	5	4.95%	196	17	8.67%	1.82 [0.77-4.32]	0.252	0.623
CPSF6	101	3	2.97%	196	19	9.69%	3.51 [1.24-9.95]	4.78E-02	0.507
CPSF7	101	1	0.99%	196	4	2.04%	2.08 [0.33-13.25]	0.514	0.805
CPT1A	101	12	11.88%	196	34	17.35%	1.56 [0.86-2.82]	0.220	0.596
CPVL	101	0	0.00%	196	7	3.57%	3.2e+07 [0e+00-Inf]	0.992	1.000
CP	101	3	2.97%	196	2	1.02%	0.34 [0.074-1.53]	0.237	0.599
CR1L	101	7	6.93%	196	24	12.24%	1.87 [0.90-3.92]	0.161	0.535
CR1	101	6	5.94%	196	24	12.24%	2.21 [1.01-4.82]	0.095	0.507
CR2	101	6	5.94%	196	24	12.24%	2.21 [1.01-4.82]	0.095	0.507

Symbol	CINSARC Low			CINSARC High			CINSARC High vs. Low		
	N tested	N altered	Fq alteration	N tested	N altered	Fq alteration	OR [95%CI]	p-value*	q-value
CRABP2	101	7	6.93%	196	8	4.08%	0.57 [0.24-1.37]	0.294	0.650
CRB1	101	5	4.95%	196	21	10.71%	2.30 [0.99-5.36]	0.104	0.507
CRCT1	101	7	6.93%	196	13	6.63%	0.95 [0.43-2.12]	0.923	1.000
CREB3L4	101	7	6.93%	196	13	6.63%	0.95 [0.43-2.12]	0.923	1.000
CREB5	101	0	0.00%	196	7	3.57%	3.2e+07 [0e+00-Inf]	0.992	1.000
CREBBP	101	5	4.95%	196	5	2.55%	0.50 [0.17-1.45]	0.286	0.650
CREBZF	101	2	1.98%	196	3	1.53%	0.77 [0.17-3.50]	0.776	0.996
CREG1	101	5	4.95%	196	12	6.12%	1.25 [0.51-3.08]	0.681	0.943
CRHR2	101	0	0.00%	196	7	3.57%	3.2e+07 [0e+00-Inf]	0.992	1.000
CRH	101	15	14.85%	196	37	18.88%	1.33 [0.77-2.31]	0.388	0.757
CRISPLD1	101	14	13.86%	196	41	20.92%	1.64 [0.94-2.86]	0.141	0.535
CRLF3	101	2	1.98%	196	4	2.04%	1.03 [0.24-4.35]	0.972	1.000
CRNN	101	7	6.93%	196	13	6.63%	0.95 [0.43-2.12]	0.923	1.000
CRP	101	6	5.94%	196	10	5.10%	0.85 [0.36-2.04]	0.762	0.996
CRTC2	101	7	6.93%	196	13	6.63%	0.95 [0.43-2.12]	0.923	1.000
CRTC3	101	1	0.99%	196	6	3.06%	3.16 [0.53-18.88]	0.290	0.650
CRYBA1	101	3	2.97%	196	5	2.55%	0.86 [0.25-2.89]	0.833	1.000
CRYM	101	5	4.95%	196	2	1.02%	0.20 [0.049-0.80]	0.056	0.507
YBX3P1	101	4	3.96%	196	4	2.04%	0.51 [0.16-1.65]	0.342	0.716
CSE1L	101	6	5.94%	196	7	3.57%	0.59 [0.23-1.50]	0.349	0.722
CSF3	101	5	4.95%	196	11	5.61%	1.14 [0.46-2.84]	0.811	1.000
CSH1	101	8	7.92%	196	29	14.80%	2.02 [1.01-4.03]	0.094	0.507
CSH2	101	8	7.92%	196	29	14.80%	2.02 [1.01-4.03]	0.094	0.507
CSHL1	101	8	7.92%	196	29	14.80%	2.02 [1.01-4.03]	0.094	0.507
CSMD1	101	1	0.99%	196	0	0.00%	1.6e-08 [0e+00-Inf]	0.996	1.000
CSMD3	101	16	15.84%	196	51	26.02%	1.87 [1.11-3.15]	4.89E-02	0.507
CSNK1D	101	2	1.98%	196	13	6.63%	3.52 [0.99-12.47]	0.102	0.507
CSPP1	101	15	14.85%	196	39	19.90%	1.42 [0.82-2.46]	0.287	0.650
CSRP1	101	7	6.93%	196	23	11.73%	1.79 [0.85-3.74]	0.198	0.570
CSRP2	101	0	0.00%	196	8	4.08%	3.6e+07 [0e+00-Inf]	0.992	1.000
CSRP3	101	1	0.99%	196	4	2.04%	2.08 [0.33-13.25]	0.514	0.805
CST6	101	0	0.00%	196	5	2.55%	2.2e+07 [0e+00-Inf]	0.992	1.000
CSTF1	101	8	7.92%	196	13	6.63%	0.83 [0.38-1.78]	0.682	0.943
CTAGE4	101	6	5.94%	196	10	5.10%	0.85 [0.36-2.04]	0.762	0.996
CTAGE6	101	4	3.96%	196	5	2.55%	0.63 [0.21-1.95]	0.505	0.805
CTCFL	101	9	8.91%	196	17	8.67%	0.97 [0.48-1.97]	0.945	1.000
CTDSP2	101	0	0.00%	196	5	2.55%	2.2e+07 [0e+00-Inf]	0.992	1.000
CTF1	101	4	3.96%	196	5	2.55%	0.63 [0.21-1.95]	0.505	0.805
CTHRC1	101	16	15.84%	196	55	28.06%	2.07 [1.23-3.48]	2.09E-02	0.507
CTNNBL1	101	5	4.95%	196	4	2.04%	0.40 [0.13-1.23]	0.179	0.535
CTSA	101	5	4.95%	196	4	2.04%	0.40 [0.13-1.23]	0.179	0.535
CTSC	101	1	0.99%	196	4	2.04%	2.08 [0.33-13.25]	0.514	0.805
CTSE	101	7	6.93%	196	24	12.24%	1.87 [0.90-3.92]	0.161	0.535
CTSF	101	5	4.95%	196	13	6.63%	1.36 [0.56-3.32]	0.566	0.852
CTSK	101	8	7.92%	196	15	7.65%	0.96 [0.46-2.04]	0.935	1.000
CTSS	101	8	7.92%	196	16	8.16%	1.03 [0.49-2.17]	0.942	1.000
CTSZ	101	9	8.91%	196	15	7.65%	0.85 [0.41-1.75]	0.707	0.962
CTTN	101	17	16.83%	196	51	26.02%	1.74 [1.04-2.90]	0.076	0.507
CUEDC1	101	6	5.94%	196	25	12.76%	2.31 [1.06-5.03]	0.076	0.507
CWC25	101	4	3.96%	196	10	5.10%	1.30 [0.48-3.53]	0.661	0.935
CXADRP2	101	1	0.99%	196	4	2.04%	2.08 [0.33-13.25]	0.514	0.805
CXCL10	101	1	0.99%	196	5	2.55%	2.62 [0.43-16.05]	0.383	0.750
CXCL11	101	1	0.99%	196	5	2.55%	2.62 [0.43-16.05]	0.383	0.750
CXCL1	101	1	0.99%	196	6	3.06%	3.16 [0.53-18.88]	0.290	0.650
CXCL2	101	2	1.98%	196	6	3.06%	1.56 [0.40-6.08]	0.589	0.869
CXCL3	101	2	1.98%	196	6	3.06%	1.56 [0.40-6.08]	0.589	0.869
CXCL5	101	2	1.98%	196	6	3.06%	1.56 [0.40-6.08]	0.589	0.869
CXCL6	101	1	0.99%	196	6	3.06%	3.16 [0.53-18.88]	0.290	0.650
CXCL9	101	1	0.99%	196	5	2.55%	2.62 [0.43-16.05]	0.383	0.750
CYB561	101	8	7.92%	196	31	15.82%	2.18 [1.10-4.34]	0.061	0.507
CYB5R1	101	7	6.93%	196	28	14.29%	2.24 [1.08-4.63]	0.068	0.507
CYB561A3	101	1	0.99%	196	4	2.04%	2.08 [0.33-13.25]	0.514	0.805
CYC1	101	17	16.83%	196	51	26.02%	1.74 [1.04-2.90]	0.076	0.507
CYCSP52	101	6	5.94%	196	8	4.08%	0.67 [0.27-1.68]	0.476	0.805
CYCS	101	0	0.00%	196	7	3.57%	3.2e+07 [0e+00-Inf]	0.992	1.000
CYGB	101	3	2.97%	196	15	7.65%	2.71 [0.94-7.82]	0.122	0.534
CYHR1	101	18	17.82%	196	49	25.00%	1.54 [0.93-2.55]	0.163	0.535
CYP11B1	101	16	15.84%	196	49	25.00%	1.77 [1.05-2.99]	0.073	0.507
CYP11B2	101	16	15.84%	196	49	25.00%	1.77 [1.05-2.99]	0.073	0.507
CYP24A1	101	12	11.88%	196	23	11.73%	0.99 [0.53-1.84]	0.970	1.000
CYP27B1	101	0	0.00%	196	5	2.55%	2.2e+07 [0e+00-Inf]	0.992	1.000

Symbol	CINSARC Low			CINSARC High			CINSARC High vs. Low		
	N tested	N altered	Fq alteration	N tested	N altered	Fq alteration	OR [95%CI]	p-value*	q-value
CYP7A1	101	14	13.86%	196	33	16.84%	1.26 [0.71-2.22]	0.506	0.805
CYP7B1	101	15	14.85%	196	33	16.84%	1.16 [0.66-2.03]	0.660	0.935
CYTH1	101	3	2.97%	196	14	7.14%	2.51 [0.86-7.30]	0.155	0.535
DAP3	101	7	6.93%	196	9	4.59%	0.65 [0.27-1.52]	0.401	0.774
DARS2	101	5	4.95%	196	13	6.63%	1.36 [0.56-3.32]	0.566	0.852
DAZ1	101	0	0.00%	196	0	0.00%	1.00 [0e+00-Inf]	1.000	1.000
DAZ2	101	0	0.00%	196	0	0.00%	1.00 [0e+00-Inf]	1.000	1.000
DAZ3	101	0	0.00%	196	0	0.00%	1.00 [0e+00-Inf]	1.000	1.000
DAZ4	101	0	0.00%	196	0	0.00%	1.00 [0e+00-Inf]	1.000	1.000
DBNDD2	101	5	4.95%	196	3	1.53%	0.30 [0.088-1.01]	0.103	0.507
DCAF13	101	16	15.84%	196	55	28.06%	2.07 [1.23-3.48]	2.09E-02	0.507
DCAF4L2	101	19	18.81%	196	49	25.00%	1.44 [0.87-2.37]	0.231	0.599
DCAF6	101	4	3.96%	196	12	6.12%	1.58 [0.60-4.18]	0.438	0.798
DCAF7	101	8	7.92%	196	30	15.31%	2.10 [1.06-4.18]	0.076	0.507
DCAF8	101	6	5.94%	196	12	6.12%	1.03 [0.44-2.41]	0.950	1.000
ECI1	101	6	5.94%	196	4	2.04%	0.33 [0.11-0.97]	0.092	0.507
DCN	101	0	0.00%	196	5	2.55%	2.2e+07 [0e+00-Inf]	0.992	1.000
DCST1	101	8	7.92%	196	15	7.65%	0.96 [0.46-2.04]	0.935	1.000
DCST2	101	8	7.92%	196	15	7.65%	0.96 [0.46-2.04]	0.935	1.000
DCTN3	101	1	0.99%	196	4	2.04%	2.08 [0.33-13.25]	0.514	0.805
DCTN5	101	5	4.95%	196	3	1.53%	0.30 [0.088-1.01]	0.103	0.507
DCTPP1	101	4	3.96%	196	5	2.55%	0.63 [0.21-1.95]	0.505	0.805
DCUN1D1	101	3	2.97%	196	2	1.02%	0.34 [0.074-1.53]	0.237	0.599
DCUN1D3	101	5	4.95%	196	2	1.02%	0.20 [0.049-0.80]	0.056	0.507
DCXR	101	2	1.98%	196	13	6.63%	3.52 [0.99-12.47]	0.102	0.507
DDB1	101	2	1.98%	196	4	2.04%	1.03 [0.24-4.35]	0.972	1.000
DDHD2	101	10	9.90%	196	43	21.94%	2.56 [1.38-4.74]	1.23E-02	0.507
DDR2	101	5	4.95%	196	14	7.14%	1.48 [0.61-3.57]	0.467	0.805
DDX27	101	8	7.92%	196	7	3.57%	0.43 [0.18-1.03]	0.114	0.512
DDX3Y	101	0	0.00%	196	0	0.00%	1.00 [0e+00-Inf]	1.000	1.000
DDX42	101	8	7.92%	196	29	14.80%	2.02 [1.01-4.03]	0.094	0.507
DDX52	101	3	2.97%	196	7	3.57%	1.21 [0.38-3.83]	0.786	0.996
DDX59	101	6	5.94%	196	24	12.24%	2.21 [1.01-4.82]	0.095	0.507
DDX5	101	8	7.92%	196	27	13.78%	1.86 [0.93-3.72]	0.143	0.535
DECR1	101	18	17.82%	196	48	24.49%	1.50 [0.90-2.48]	0.192	0.563
DECR2	101	7	6.93%	196	4	2.04%	0.28 [0.098-0.80]	4.63E-02	0.507
DEDD	101	6	5.94%	196	16	8.16%	1.41 [0.62-3.18]	0.490	0.805
DEFB108B	101	6	5.94%	196	8	4.08%	0.67 [0.27-1.68]	0.476	0.805
DEGS1	101	6	5.94%	196	17	8.67%	1.50 [0.67-3.38]	0.407	0.774
DENND1B	101	5	4.95%	196	21	10.71%	2.30 [0.99-5.36]	0.104	0.507
DENND3	101	18	17.82%	196	50	25.51%	1.58 [0.95-2.62]	0.137	0.535
DENND4B	101	7	6.93%	196	13	6.63%	0.95 [0.43-2.12]	0.923	1.000
DEPTOR	101	15	14.85%	196	54	27.55%	2.18 [1.28-3.70]	1.56E-02	0.507
DERL1	101	14	13.86%	196	56	28.57%	2.49 [1.45-4.27]	5.58E-03	0.507
DET1	101	0	0.00%	196	6	3.06%	2.7e+07 [0e+00-Inf]	0.992	1.000
DEXI	101	5	4.95%	196	4	2.04%	0.40 [0.13-1.23]	0.179	0.535
DGAT1	101	18	17.82%	196	50	25.51%	1.58 [0.95-2.62]	0.137	0.535
DGAT2	101	6	5.94%	196	20	10.20%	1.80 [0.81-3.98]	0.224	0.596
DGKE	101	8	7.92%	196	23	11.73%	1.55 [0.76-3.14]	0.311	0.680
DHCR7	101	12	11.88%	196	24	12.24%	1.03 [0.56-1.92]	0.928	1.000
DHRS11	101	3	2.97%	196	5	2.55%	0.86 [0.25-2.89]	0.833	1.000
DHRS13	101	3	2.97%	196	7	3.57%	1.21 [0.38-3.83]	0.786	0.996
DHX35	101	3	2.97%	196	5	2.55%	0.86 [0.25-2.89]	0.833	1.000
DHX40	101	9	8.91%	196	31	15.82%	1.92 [0.99-3.71]	0.103	0.507
DHX9	101	5	4.95%	196	18	9.18%	1.94 [0.82-4.58]	0.203	0.570
DIDO1	101	7	6.93%	196	13	6.63%	0.95 [0.43-2.12]	0.923	1.000
DISC1	101	6	5.94%	196	19	9.69%	1.70 [0.77-3.78]	0.274	0.643
DISC2	101	6	5.94%	196	19	9.69%	1.70 [0.77-3.78]	0.274	0.643
DISP1	101	6	5.94%	196	18	9.18%	1.60 [0.72-3.57]	0.335	0.716
DKFZP586I14	101	0	0.00%	196	7	3.57%	3.2e+07 [0e+00-Inf]	0.992	1.000
NCR3LG1	101	1	0.99%	196	4	2.04%	2.08 [0.33-13.25]	0.514	0.805
DKK4	101	14	13.86%	196	32	16.33%	1.21 [0.69-2.15]	0.578	0.867
DLG1	101	3	2.97%	196	5	2.55%	0.86 [0.25-2.89]	0.833	1.000
DLGAP4	101	4	3.96%	196	6	3.06%	0.77 [0.26-2.26]	0.685	0.943
DLX3	101	4	3.96%	196	17	8.67%	2.30 [0.90-5.88]	0.143	0.535
DLX4	101	4	3.96%	196	17	8.67%	2.30 [0.90-5.88]	0.143	0.535
DNAH11	101	0	0.00%	196	6	3.06%	2.7e+07 [0e+00-Inf]	0.992	1.000
DNAH14	101	6	5.94%	196	18	9.18%	1.60 [0.72-3.57]	0.335	0.716
DNAH17	101	3	2.97%	196	14	7.14%	2.51 [0.86-7.30]	0.155	0.535
DNAH3	101	5	4.95%	196	2	1.02%	0.20 [0.049-0.80]	0.056	0.507
DNAI2	101	6	5.94%	196	15	7.65%	1.31 [0.58-2.98]	0.586	0.869

Symbol	CINSARC Low			CINSARC High			CINSARC High vs. Low		
	N tested	N altered	Fq alteration	N tested	N altered	Fq alteration	OR [95%CI]	p-value*	q-value
DNAJA3	101	5	4.95%	196	6	3.06%	0.61 [0.22-1.68]	0.418	0.779
DNAJB13	101	7	6.93%	196	12	6.12%	0.88 [0.39-1.97]	0.788	0.996
DNAJB5	101	1	0.99%	196	4	2.04%	2.08 [0.33-13.25]	0.514	0.805
DNAJB6	101	1	0.99%	196	4	2.04%	2.08 [0.33-13.25]	0.514	0.805
DNAJC19	101	4	3.96%	196	2	1.02%	0.25 [0.059-1.05]	0.113	0.511
DNAJC5B	101	15	14.85%	196	37	18.88%	1.33 [0.77-2.31]	0.388	0.757
DNAJC5	101	6	5.94%	196	12	6.12%	1.03 [0.44-2.41]	0.950	1.000
DNAJC9	101	0	0.00%	196	5	2.55%	2.2e+07 [0e+00-Inf]	0.992	1.000
DNASE1L2	101	6	5.94%	196	4	2.04%	0.33 [0.11-0.97]	0.092	0.507
DNASE1	101	7	6.93%	196	4	2.04%	0.28 [0.098-0.80]	4.63E-02	0.507
DNM3	101	5	4.95%	196	13	6.63%	1.36 [0.56-3.32]	0.566	0.852
DNTTIP1	101	5	4.95%	196	4	2.04%	0.40 [0.13-1.23]	0.179	0.535
DOC2A	101	5	4.95%	196	4	2.04%	0.40 [0.13-1.23]	0.179	0.535
DOK5	101	10	9.90%	196	17	8.67%	0.86 [0.43-1.72]	0.728	0.986
DPH3P1	101	7	6.93%	196	14	7.14%	1.03 [0.47-2.28]	0.946	1.000
DPM1	101	6	5.94%	196	14	7.14%	1.22 [0.53-2.79]	0.696	0.951
DPM3	101	7	6.93%	196	13	6.63%	0.95 [0.43-2.12]	0.923	1.000
DPP3	101	5	4.95%	196	11	5.61%	1.14 [0.46-2.84]	0.811	1.000
DPRXP4	101	2	1.98%	196	3	1.53%	0.77 [0.17-3.50]	0.776	0.996
DPT	101	5	4.95%	196	13	6.63%	1.36 [0.56-3.32]	0.566	0.852
DPY19L1	101	0	0.00%	196	5	2.55%	2.2e+07 [0e+00-Inf]	0.992	1.000
DPY19L2P1	101	0	0.00%	196	5	2.55%	2.2e+07 [0e+00-Inf]	0.992	1.000
DPY19L2	101	2	1.98%	196	4	2.04%	1.03 [0.24-4.35]	0.972	1.000
DPY19L4	101	16	15.84%	196	54	27.55%	2.02 [1.20-3.40]	2.60E-02	0.507
DPYS	101	17	16.83%	196	50	25.51%	1.69 [1.01-2.83]	0.092	0.507
DSCC1	101	15	14.85%	196	54	27.55%	2.18 [1.28-3.70]	1.56E-02	0.507
DSG1	101	1	0.99%	196	4	2.04%	2.08 [0.33-13.25]	0.514	0.805
DSG4	101	1	0.99%	196	4	2.04%	2.08 [0.33-13.25]	0.514	0.805
DSN1	101	4	3.96%	196	6	3.06%	0.77 [0.26-2.26]	0.685	0.943
DSTYK	101	7	6.93%	196	26	13.27%	2.05 [0.99-4.27]	0.106	0.507
DTL	101	6	5.94%	196	20	10.20%	1.80 [0.81-3.98]	0.224	0.596
DUPD1	101	2	1.98%	196	7	3.57%	1.83 [0.48-6.96]	0.455	0.805
DUS1L	101	2	1.98%	196	14	7.14%	3.81 [1.08-13.43]	0.081	0.507
DUSP10	101	6	5.94%	196	20	10.20%	1.80 [0.81-3.98]	0.224	0.596
DUSP12	101	6	5.94%	196	15	7.65%	1.31 [0.58-2.98]	0.586	0.869
DUSP13	101	2	1.98%	196	6	3.06%	1.56 [0.40-6.08]	0.589	0.869
DUSP14	101	2	1.98%	196	7	3.57%	1.83 [0.48-6.96]	0.455	0.805
DUSP23	101	6	5.94%	196	10	5.10%	0.85 [0.36-2.04]	0.762	0.996
DUSP26	101	4	3.96%	196	12	6.12%	1.58 [0.60-4.18]	0.438	0.798
DUSP27	101	5	4.95%	196	13	6.63%	1.36 [0.56-3.32]	0.566	0.852
DUSP5P1	101	6	5.94%	196	21	10.71%	1.90 [0.86-4.19]	0.181	0.535
DUSP6	101	0	0.00%	196	5	2.55%	2.2e+07 [0e+00-Inf]	0.992	1.000
DVL3	101	3	2.97%	196	2	1.02%	0.34 [0.074-1.53]	0.237	0.599
DYNLL2	101	6	5.94%	196	24	12.24%	2.21 [1.01-4.82]	0.095	0.507
DYNLRB1	101	3	2.97%	196	4	2.04%	0.68 [0.19-2.43]	0.619	0.898
DYRK2	101	3	2.97%	196	15	7.65%	2.71 [0.94-7.82]	0.122	0.534
DYRK3	101	6	5.94%	196	25	12.76%	2.31 [1.06-5.03]	0.076	0.507
PPP1R27	101	2	1.98%	196	13	6.63%	3.52 [0.99-12.47]	0.102	0.507
E2F5	101	17	16.83%	196	47	23.98%	1.56 [0.93-2.61]	0.158	0.535
E2F7	101	0	0.00%	196	8	4.08%	3.6e+07 [0e+00-Inf]	0.992	1.000
E2F8	101	1	0.99%	196	5	2.55%	2.62 [0.43-16.05]	0.383	0.750
E4F1	101	6	5.94%	196	4	2.04%	0.33 [0.11-0.97]	0.092	0.507
EAPP	101	0	0.00%	196	7	3.57%	3.2e+07 [0e+00-Inf]	0.992	1.000
EARS2	101	5	4.95%	196	2	1.02%	0.20 [0.049-0.80]	0.056	0.507
EBAG9	101	15	14.85%	196	54	27.55%	2.18 [1.28-3.70]	1.56E-02	0.507
ECD	101	1	0.99%	196	5	2.55%	2.62 [0.43-16.05]	0.383	0.750
ECE2	101	3	2.97%	196	2	1.02%	0.34 [0.074-1.53]	0.237	0.599
ECM1	101	9	8.91%	196	16	8.16%	0.91 [0.44-1.86]	0.826	1.000
EDARADD	101	7	6.93%	196	19	9.69%	1.44 [0.68-3.07]	0.427	0.791
EDEM2	101	4	3.96%	196	8	4.08%	1.03 [0.37-2.88]	0.960	1.000
EDEM3	101	5	4.95%	196	19	9.69%	2.06 [0.88-4.83]	0.163	0.535
EDN3	101	10	9.90%	196	15	7.65%	0.75 [0.37-1.52]	0.510	0.805
EEA1	101	0	0.00%	196	5	2.55%	2.2e+07 [0e+00-Inf]	0.992	1.000
EED	101	1	0.99%	196	4	2.04%	2.08 [0.33-13.25]	0.514	0.805
EEF1A2	101	6	5.94%	196	14	7.14%	1.22 [0.53-2.79]	0.696	0.951
EEF1D	101	16	15.84%	196	51	26.02%	1.87 [1.11-3.15]	4.89E-02	0.507
EEF2K	101	5	4.95%	196	3	1.53%	0.30 [0.088-1.01]	0.103	0.507
EFCAB1	101	14	13.86%	196	25	12.76%	0.91 [0.50-1.64]	0.789	0.996
EFCAB2	101	5	4.95%	196	18	9.18%	1.94 [0.82-4.58]	0.203	0.570
EFCAB3	101	9	8.91%	196	34	17.35%	2.15 [1.12-4.12]	0.055	0.507
EFCAB5	101	2	1.98%	196	5	2.55%	1.30 [0.32-5.21]	0.759	0.996

Symbol	CINSARC Low			CINSARC High			CINSARC High vs. Low		
	N tested	N altered	Fq alteration	N tested	N altered	Fq alteration	OR [95%CI]	p-value*	q-value
EFNA1	101	7	6.93%	196	13	6.63%	0.95 [0.43-2.12]	0.923	1.000
EFNA3	101	7	6.93%	196	15	7.65%	1.11 [0.51-2.43]	0.822	1.000
EFNA4	101	8	7.92%	196	15	7.65%	0.96 [0.46-2.04]	0.935	1.000
EFR3A	101	15	14.85%	196	55	28.06%	2.24 [1.32-3.80]	1.24E-02	0.507
EGLN1	101	6	5.94%	196	19	9.69%	1.70 [0.77-3.78]	0.274	0.643
EGLN3	101	0	0.00%	196	9	4.59%	4.1e+07 [0e+00-Inf]	0.992	1.000
EHHADH	101	3	2.97%	196	3	1.53%	0.51 [0.13-1.98]	0.412	0.774
EIF1AD	101	0	0.00%	196	5	2.55%	2.2e+07 [0e+00-Inf]	0.992	1.000
EIF1AY	101	0	0.00%	196	0	0.00%	1.00 [0e+00-Inf]	1.000	1.000
EIF2B5	101	3	2.97%	196	2	1.02%	0.34 [0.074-1.53]	0.237	0.599
AGO2	101	18	17.82%	196	51	26.02%	1.62 [0.98-2.69]	0.115	0.516
EIF3CL	101	6	5.94%	196	3	1.53%	0.25 [0.076-0.80]	0.051	0.507
EIF3C	101	6	5.94%	196	3	1.53%	0.25 [0.076-0.80]	0.051	0.507
EIF3E	101	16	15.84%	196	50	25.51%	1.82 [1.08-3.07]	0.060	0.507
EIF3H	101	16	15.84%	196	59	30.10%	2.29 [1.37-3.83]	8.37E-03	0.507
EIF4A3	101	3	2.97%	196	15	7.65%	2.71 [0.94-7.82]	0.122	0.534
EIF4EBP1	101	10	9.90%	196	50	25.51%	3.12 [1.69-5.74]	2.20E-03	0.507
EIF4G1	101	3	2.97%	196	2	1.02%	0.34 [0.074-1.53]	0.237	0.599
EIF6	101	4	3.96%	196	6	3.06%	0.77 [0.26-2.26]	0.685	0.943
ELF3	101	8	7.92%	196	24	12.24%	1.62 [0.80-3.28]	0.258	0.635
ELK4	101	7	6.93%	196	26	13.27%	2.05 [0.99-4.27]	0.106	0.507
ELMO2	101	4	3.96%	196	7	3.57%	0.90 [0.31-2.57]	0.867	1.000
EME1	101	4	3.96%	196	19	9.69%	2.60 [1.03-6.59]	0.090	0.507
EME2	101	6	5.94%	196	5	2.55%	0.41 [0.15-1.15]	0.154	0.535
EMILIN3	101	2	1.98%	196	4	2.04%	1.03 [0.24-4.35]	0.972	1.000
EMP2	101	5	4.95%	196	4	2.04%	0.40 [0.13-1.23]	0.179	0.535
ENAH	101	6	5.94%	196	17	8.67%	1.50 [0.67-3.38]	0.407	0.774
ENGASE	101	3	2.97%	196	14	7.14%	2.51 [0.86-7.30]	0.155	0.535
ENHO	101	1	0.99%	196	4	2.04%	2.08 [0.33-13.25]	0.514	0.805
ENPP2	101	15	14.85%	196	56	28.57%	2.29 [1.35-3.89]	9.81E-03	0.507
ENPP7	101	4	3.96%	196	17	8.67%	2.30 [0.90-5.88]	0.143	0.535
ENSA	101	9	8.91%	196	17	8.67%	0.97 [0.48-1.97]	0.945	1.000
ENY2	101	15	14.85%	196	54	27.55%	2.18 [1.28-3.70]	1.56E-02	0.507
EPB41L1	101	4	3.96%	196	7	3.57%	0.90 [0.31-2.57]	0.867	1.000
EPGN	101	2	1.98%	196	4	2.04%	1.03 [0.24-4.35]	0.972	1.000
EPHB3	101	3	2.97%	196	3	1.53%	0.51 [0.13-1.98]	0.412	0.774
EPHB6	101	2	1.98%	196	4	2.04%	1.03 [0.24-4.35]	0.972	1.000
EPHX1	101	7	6.93%	196	20	10.20%	1.53 [0.72-3.24]	0.355	0.722
EPN1	101	2	1.98%	196	4	2.04%	1.03 [0.24-4.35]	0.972	1.000
EPN2	101	4	3.96%	196	1	0.51%	0.12 [0.02-0.79]	0.064	0.507
EPN3	101	4	3.96%	196	19	9.69%	2.60 [1.03-6.59]	0.090	0.507
EPPK1	101	17	16.83%	196	51	26.02%	1.74 [1.04-2.90]	0.076	0.507
EPRS	101	6	5.94%	196	20	10.20%	1.80 [0.81-3.98]	0.224	0.596
EPX	101	6	5.94%	196	26	13.27%	2.42 [1.12-5.25]	0.060	0.507
ERAL1	101	3	2.97%	196	8	4.08%	1.39 [0.45-4.31]	0.632	0.909
ERBB2	101	5	4.95%	196	13	6.63%	1.36 [0.56-3.32]	0.566	0.852
ERCC4	101	6	5.94%	196	3	1.53%	0.25 [0.076-0.80]	0.051	0.507
EREG	101	2	1.98%	196	4	2.04%	1.03 [0.24-4.35]	0.972	1.000
ERI2	101	5	4.95%	196	2	1.02%	0.20 [0.049-0.80]	0.056	0.507
ERLIN2	101	11	10.89%	196	53	27.04%	3.03 [1.68-5.46]	1.92E-03	0.507
ERN1	101	8	7.92%	196	27	13.78%	1.86 [0.93-3.72]	0.143	0.535
ERN2	101	5	4.95%	196	3	1.53%	0.30 [0.088-1.01]	0.103	0.507
ESRP1	101	17	16.83%	196	54	27.55%	1.88 [1.13-3.13]	4.21E-02	0.507
ESRRG	101	6	5.94%	196	21	10.71%	1.90 [0.86-4.19]	0.181	0.535
ETNK2	101	7	6.93%	196	28	14.29%	2.24 [1.08-4.63]	0.068	0.507
ETV3L	101	6	5.94%	196	8	4.08%	0.67 [0.27-1.68]	0.476	0.805
ETV3	101	6	5.94%	196	8	4.08%	0.67 [0.27-1.68]	0.476	0.805
EVPL	101	4	3.96%	196	12	6.12%	1.58 [0.60-4.18]	0.438	0.798
EVX1	101	0	0.00%	196	6	3.06%	2.7e+07 [0e+00-Inf]	0.992	1.000
EXO1	101	4	3.96%	196	19	9.69%	2.60 [1.03-6.59]	0.090	0.507
EXOC7	101	4	3.96%	196	13	6.63%	1.72 [0.66-4.51]	0.353	0.722
EXOC8	101	6	5.94%	196	19	9.69%	1.70 [0.77-3.78]	0.274	0.643
EXOSC4	101	16	15.84%	196	51	26.02%	1.87 [1.11-3.15]	4.89E-02	0.507
EXT1	101	15	14.85%	196	55	28.06%	2.24 [1.32-3.80]	1.24E-02	0.507
EYA1	101	12	11.88%	196	34	17.35%	1.56 [0.86-2.82]	0.220	0.596
EYA2	101	5	4.95%	196	7	3.57%	0.71 [0.27-1.90]	0.569	0.855
F11R	101	6	5.94%	196	17	8.67%	1.50 [0.67-3.38]	0.407	0.774
F13B	101	5	4.95%	196	21	10.71%	2.30 [0.99-5.36]	0.104	0.507
F5	101	4	3.96%	196	13	6.63%	1.72 [0.66-4.51]	0.353	0.722
FABP12	101	17	16.83%	196	47	23.98%	1.56 [0.93-2.61]	0.158	0.535
FABP4	101	17	16.83%	196	47	23.98%	1.56 [0.93-2.61]	0.158	0.535

Symbol	CINSARC Low			CINSARC High			CINSARC High vs. Low		
	N tested	N altered	Fq alteration	N tested	N altered	Fq alteration	OR [95%CI]	p-value*	q-value
FABP5	101	17	16.83%	196	47	23.98%	1.56 [0.93-2.61]	0.158	0.535
FABP9	101	17	16.83%	196	47	23.98%	1.56 [0.93-2.61]	0.158	0.535
FADD	101	20	19.80%	196	56	28.57%	1.62 [1.00-2.63]	0.103	0.507
FADS6	101	5	4.95%	196	13	6.63%	1.36 [0.56-3.32]	0.566	0.852
FAHD1	101	6	5.94%	196	4	2.04%	0.33 [0.11-0.97]	0.092	0.507
UBALD1	101	5	4.95%	196	6	3.06%	0.61 [0.22-1.68]	0.418	0.779
UBALD2	101	3	2.97%	196	14	7.14%	2.51 [0.86-7.30]	0.155	0.535
FAM104A	101	5	4.95%	196	17	8.67%	1.82 [0.77-4.32]	0.252	0.623
FAM106A	101	6	5.94%	196	6	3.06%	0.50 [0.19-1.32]	0.241	0.608
ABHD17C	101	2	1.98%	196	4	2.04%	1.03 [0.24-4.35]	0.972	1.000
FAM110B	101	14	13.86%	196	33	16.84%	1.26 [0.71-2.22]	0.506	0.805
FAM117A	101	4	3.96%	196	16	8.16%	2.16 [0.84-5.53]	0.180	0.535
FAM126A	101	0	0.00%	196	6	3.06%	2.7e+07 [0e+00-Inf]	0.992	1.000
FAM129A	101	5	4.95%	196	18	9.18%	1.94 [0.82-4.58]	0.203	0.570
FAM131A	101	3	2.97%	196	2	1.02%	0.34 [0.074-1.53]	0.237	0.599
FAM135B	101	15	14.85%	196	51	26.02%	2.02 [1.18-3.43]	3.03E-02	0.507
FAM149B1	101	0	0.00%	196	5	2.55%	2.2e+07 [0e+00-Inf]	0.992	1.000
FAM163A	101	6	5.94%	196	19	9.69%	1.70 [0.77-3.78]	0.274	0.643
ZC2HC1A	101	15	14.85%	196	47	23.98%	1.81 [1.06-3.09]	0.069	0.507
FAM168A	101	7	6.93%	196	13	6.63%	0.95 [0.43-2.12]	0.923	1.000
FAM169B	101	1	0.99%	196	8	4.08%	4.26 [0.73-24.65]	0.175	0.535
FAM173A	101	6	5.94%	196	4	2.04%	0.33 [0.11-0.97]	0.092	0.507
FAM174B	101	0	0.00%	196	7	3.57%	3.2e+07 [0e+00-Inf]	0.992	1.000
FAM177A1	101	0	0.00%	196	10	5.10%	4.6e+07 [0e+00-Inf]	0.992	1.000
FAM177B	101	6	5.94%	196	20	10.20%	1.80 [0.81-3.98]	0.224	0.596
FAM181B	101	2	1.98%	196	4	2.04%	1.03 [0.24-4.35]	0.972	1.000
FAM189B	101	7	6.93%	196	14	7.14%	1.03 [0.47-2.28]	0.946	1.000
TVP23A	101	5	4.95%	196	4	2.04%	0.40 [0.13-1.23]	0.179	0.535
FAM197Y2	101	0	0.00%	196	0	0.00%	1.00 [0e+00-Inf]	1.000	1.000
FAM20A	101	8	7.92%	196	24	12.24%	1.62 [0.80-3.28]	0.258	0.635
FAM20B	101	5	4.95%	196	18	9.18%	1.94 [0.82-4.58]	0.203	0.570
COX20	101	5	4.95%	196	18	9.18%	1.94 [0.82-4.58]	0.203	0.570
FAM41AY1	101	0	0.00%	196	0	0.00%	1.00 [0e+00-Inf]	1.000	1.000
FAM43A	101	3	2.97%	196	3	1.53%	0.51 [0.13-1.98]	0.412	0.774
FAM47E	101	1	0.99%	196	5	2.55%	2.62 [0.43-16.05]	0.383	0.750
FAM49B	101	16	15.84%	196	57	29.08%	2.18 [1.30-3.66]	1.33E-02	0.507
FAM57B	101	5	4.95%	196	4	2.04%	0.40 [0.13-1.23]	0.179	0.535
BRINP2	101	6	5.94%	196	17	8.67%	1.50 [0.67-3.38]	0.407	0.774
BRINP3	101	6	5.94%	196	17	8.67%	1.50 [0.67-3.38]	0.407	0.774
FAM66D	101	2	1.98%	196	4	2.04%	1.03 [0.24-4.35]	0.972	1.000
FAM71A	101	6	5.94%	196	21	10.71%	1.90 [0.86-4.19]	0.181	0.535
FAM71E2	101	1	0.99%	196	4	2.04%	2.08 [0.33-13.25]	0.514	0.805
FAM72A	101	6	5.94%	196	24	12.24%	2.21 [1.01-4.82]	0.095	0.507
SPATA31A6	101	0	0.00%	196	5	2.55%	2.2e+07 [0e+00-Inf]	0.992	1.000
FAM78B	101	4	3.96%	196	12	6.12%	1.58 [0.60-4.18]	0.438	0.798
RMDN1	101	18	17.82%	196	47	23.98%	1.45 [0.87-2.42]	0.226	0.598
FAM83A	101	14	13.86%	196	57	29.08%	2.55 [1.49-4.37]	4.36E-03	0.507
FAM83C	101	4	3.96%	196	6	3.06%	0.77 [0.26-2.26]	0.685	0.943
FAM83D	101	2	1.98%	196	6	3.06%	1.56 [0.40-6.08]	0.589	0.869
FAM83H	101	17	16.83%	196	50	25.51%	1.69 [1.01-2.83]	0.092	0.507
FAM86C1	101	5	4.95%	196	10	5.10%	1.03 [0.41-2.60]	0.955	1.000
FAM89A	101	6	5.94%	196	19	9.69%	1.70 [0.77-3.78]	0.274	0.643
FAM91A1	101	14	13.86%	196	57	29.08%	2.55 [1.49-4.37]	4.36E-03	0.507
FANCF	101	3	2.97%	196	2	1.02%	0.34 [0.074-1.53]	0.237	0.599
FANCI	101	1	0.99%	196	6	3.06%	3.16 [0.53-18.88]	0.290	0.650
FASLG	101	5	4.95%	196	15	7.65%	1.59 [0.66-3.81]	0.382	0.750
FASN	101	2	1.98%	196	13	6.63%	3.52 [0.99-12.47]	0.102	0.507
FBF1	101	4	3.96%	196	12	6.12%	1.58 [0.60-4.18]	0.438	0.798
FBRS	101	4	3.96%	196	5	2.55%	0.63 [0.21-1.95]	0.505	0.805
FBXL16	101	6	5.94%	196	4	2.04%	0.33 [0.11-0.97]	0.092	0.507
FBXL19	101	4	3.96%	196	6	3.06%	0.77 [0.26-2.26]	0.685	0.943
FBXL20	101	5	4.95%	196	12	6.12%	1.25 [0.51-3.08]	0.681	0.943
FBXL6	101	18	17.82%	196	49	25.00%	1.54 [0.93-2.55]	0.163	0.535
FBXO28	101	6	5.94%	196	17	8.67%	1.50 [0.67-3.38]	0.407	0.774
FBXO32	101	14	13.86%	196	57	29.08%	2.55 [1.49-4.37]	4.36E-03	0.507
FBXO3	101	1	0.99%	196	4	2.04%	2.08 [0.33-13.25]	0.514	0.805
FBXO43	101	15	14.85%	196	54	27.55%	2.18 [1.28-3.70]	1.56E-02	0.507
FBXO45	101	4	3.96%	196	4	2.04%	0.51 [0.16-1.65]	0.342	0.716
FBXO47	101	4	3.96%	196	12	6.12%	1.58 [0.60-4.18]	0.438	0.798
FCAMR	101	7	6.93%	196	25	12.76%	1.96 [0.94-4.09]	0.131	0.535
FCER1A	101	6	5.94%	196	9	4.59%	0.76 [0.31-1.86]	0.616	0.898

Symbol	CINSARC Low			CINSARC High			CINSARC High vs. Low		
	N tested	N altered	Fq alteration	N tested	N altered	Fq alteration	OR [95%CI]	p-value*	q-value
FCER1G	101	6	5.94%	196	16	8.16%	1.41 [0.62-3.18]	0.490	0.805
FCGR1A	101	7	6.93%	196	12	6.12%	0.88 [0.39-1.97]	0.788	0.996
FCGR2A	101	7	6.93%	196	18	9.18%	1.36 [0.63-2.91]	0.509	0.805
FCGR2B	101	10	9.90%	196	19	9.69%	0.98 [0.50-1.92]	0.955	1.000
FCGR2C	101	9	8.91%	196	22	11.22%	1.29 [0.65-2.56]	0.538	0.835
FCGR3A	101	9	8.91%	196	20	10.20%	1.16 [0.58-2.32]	0.722	0.980
FCGR3B	101	9	8.91%	196	22	11.22%	1.29 [0.65-2.56]	0.538	0.835
FCHO1	101	2	1.98%	196	3	1.53%	0.77 [0.17-3.50]	0.776	0.996
FCHSD2	101	6	5.94%	196	9	4.59%	0.76 [0.31-1.86]	0.616	0.898
FCRL1	101	7	6.93%	196	9	4.59%	0.65 [0.27-1.52]	0.401	0.774
FCRL2	101	7	6.93%	196	9	4.59%	0.65 [0.27-1.52]	0.401	0.774
FCRL3	101	6	5.94%	196	8	4.08%	0.67 [0.27-1.68]	0.476	0.805
FCRL4	101	6	5.94%	196	8	4.08%	0.67 [0.27-1.68]	0.476	0.805
FCRL5	101	6	5.94%	196	8	4.08%	0.67 [0.27-1.68]	0.476	0.805
FCRL6	101	6	5.94%	196	10	5.10%	0.85 [0.36-2.04]	0.762	0.996
FCRLA	101	6	5.94%	196	15	7.65%	1.31 [0.58-2.98]	0.586	0.869
FCRLB	101	6	5.94%	196	15	7.65%	1.31 [0.58-2.98]	0.586	0.869
FDPS	101	7	6.93%	196	14	7.14%	1.03 [0.47-2.28]	0.946	1.000
FDXR	101	5	4.95%	196	13	6.63%	1.36 [0.56-3.32]	0.566	0.852
FER1L6	101	14	13.86%	196	57	29.08%	2.55 [1.49-4.37]	4.36E-03	0.507
FES	101	1	0.99%	196	4	2.04%	2.08 [0.33-13.25]	0.514	0.805
FGD5	101	0	0.00%	196	5	2.55%	2.2e+07 [0e+00-Inf]	0.992	1.000
FGD6	101	0	0.00%	196	5	2.55%	2.2e+07 [0e+00-Inf]	0.992	1.000
FGF12	101	3	2.97%	196	4	2.04%	0.68 [0.19-2.43]	0.619	0.898
FGF19	101	23	22.77%	196	63	32.14%	1.61 [1.01-2.56]	0.093	0.507
FGF3	101	24	23.76%	196	59	30.10%	1.38 [0.87-2.19]	0.250	0.623
FGF4	101	24	23.76%	196	58	29.59%	1.35 [0.85-2.14]	0.288	0.650
FGFR1	101	11	10.89%	196	43	21.94%	2.30 [1.27-4.18]	2.18E-02	0.507
FH	101	4	3.96%	196	20	10.20%	2.76 [1.09-6.95]	0.071	0.507
FITM2	101	3	2.97%	196	3	1.53%	0.51 [0.13-1.98]	0.412	0.774
FIZ1	101	2	1.98%	196	4	2.04%	1.03 [0.24-4.35]	0.972	1.000
FKBP14	101	0	0.00%	196	6	3.06%	2.7e+07 [0e+00-Inf]	0.992	1.000
FKBP9	101	0	0.00%	196	8	4.08%	3.6e+07 [0e+00-Inf]	0.992	1.000
FLAD1	101	8	7.92%	196	15	7.65%	0.96 [0.46-2.04]	0.935	1.000
FLG2	101	7	6.93%	196	14	7.14%	1.03 [0.47-2.28]	0.946	1.000
FLG	101	7	6.93%	196	14	7.14%	1.03 [0.47-2.28]	0.946	1.000
FLJ16779	101	7	6.93%	196	13	6.63%	0.95 [0.43-2.12]	0.923	1.000
FLJ42393	101	2	1.98%	196	3	1.53%	0.77 [0.17-3.50]	0.776	0.996
FLJ42627	101	9	8.91%	196	5	2.55%	0.27 [0.10-0.69]	2.12E-02	0.507
FLOT2	101	3	2.97%	196	8	4.08%	1.39 [0.45-4.31]	0.632	0.909
FLT1	101	0	0.00%	196	5	2.55%	2.2e+07 [0e+00-Inf]	0.992	1.000
FLVCR1	101	6	5.94%	196	21	10.71%	1.90 [0.86-4.19]	0.181	0.535
FLYWCH1	101	6	5.94%	196	4	2.04%	0.33 [0.11-0.97]	0.092	0.507
FLYWCH2	101	6	5.94%	196	5	2.55%	0.41 [0.15-1.15]	0.154	0.535
FMN2	101	5	4.95%	196	15	7.65%	1.59 [0.66-3.81]	0.382	0.750
FMO1	101	5	4.95%	196	13	6.63%	1.36 [0.56-3.32]	0.566	0.852
FMO2	101	5	4.95%	196	13	6.63%	1.36 [0.56-3.32]	0.566	0.852
FMO3	101	5	4.95%	196	13	6.63%	1.36 [0.56-3.32]	0.566	0.852
FMO4	101	5	4.95%	196	13	6.63%	1.36 [0.56-3.32]	0.566	0.852
FMO5	101	6	5.94%	196	12	6.12%	1.03 [0.44-2.41]	0.950	1.000
FMO6P	101	5	4.95%	196	13	6.63%	1.36 [0.56-3.32]	0.566	0.852
FMO9P	101	4	3.96%	196	11	5.61%	1.44 [0.54-3.85]	0.540	0.837
FMOD	101	7	6.93%	196	28	14.29%	2.24 [1.08-4.63]	0.068	0.507
FN3KRP	101	2	1.98%	196	15	7.65%	4.10 [1.17-14.39]	0.064	0.507
FN3K	101	2	1.98%	196	15	7.65%	4.10 [1.17-14.39]	0.064	0.507
FNDC8	101	1	0.99%	196	7	3.57%	3.70 [0.63-21.75]	0.224	0.596
FNTA	101	13	12.87%	196	22	11.22%	0.86 [0.46-1.58]	0.677	0.943
FOLR1	101	4	3.96%	196	8	4.08%	1.03 [0.37-2.88]	0.960	1.000
FOLR2	101	5	4.95%	196	8	4.08%	0.82 [0.31-2.13]	0.729	0.986
FOLR3	101	4	3.96%	196	7	3.57%	0.90 [0.31-2.57]	0.867	1.000
FOXA1	101	0	0.00%	196	14	7.14%	6.6e+07 [0e+00-Inf]	0.992	1.000
FOXH1	101	18	17.82%	196	49	25.00%	1.54 [0.93-2.55]	0.163	0.535
FOXJ1	101	4	3.96%	196	13	6.63%	1.72 [0.66-4.51]	0.353	0.722
FOXK2	101	2	1.98%	196	15	7.65%	4.10 [1.17-14.39]	0.064	0.507
FOXN1	101	3	2.97%	196	8	4.08%	1.39 [0.45-4.31]	0.632	0.909
FPR1	101	1	0.99%	196	4	2.04%	2.08 [0.33-13.25]	0.514	0.805
FPR2	101	1	0.99%	196	4	2.04%	2.08 [0.33-13.25]	0.514	0.805
FPR3	101	1	0.99%	196	4	2.04%	2.08 [0.33-13.25]	0.514	0.805
FRS2	101	2	1.98%	196	17	8.67%	4.70 [1.35-16.35]	4.11E-02	0.507
FSCN2	101	2	1.98%	196	12	6.12%	3.23 [0.90-11.53]	0.130	0.535
FSD2	101	1	0.99%	196	4	2.04%	2.08 [0.33-13.25]	0.514	0.805

Symbol	CINSARC Low			CINSARC High			CINSARC High vs. Low		
	N tested	N altered	Fq alteration	N tested	N altered	Fq alteration	OR [95%CI]	p-value*	q-value
FTSJ3	101	8	7.92%	196	29	14.80%	2.02 [1.01-4.03]	0.094	0.507
FURIN	101	1	0.99%	196	4	2.04%	2.08 [0.33-13.25]	0.514	0.805
FUS	101	4	3.96%	196	5	2.55%	0.63 [0.21-1.95]	0.505	0.805
FUT10	101	4	3.96%	196	10	5.10%	1.30 [0.48-3.53]	0.661	0.935
FUT11	101	0	0.00%	196	5	2.55%	2.2e+07 [0e+00-Inf]	0.992	1.000
FUT3	101	3	2.97%	196	2	1.02%	0.34 [0.074-1.53]	0.237	0.599
FXR1	101	4	3.96%	196	2	1.02%	0.25 [0.059-1.05]	0.113	0.511
FYTTD1	101	3	2.97%	196	4	2.04%	0.68 [0.19-2.43]	0.619	0.898
FZD4	101	2	1.98%	196	5	2.55%	1.30 [0.32-5.21]	0.759	0.996
FZD6	101	16	15.84%	196	56	28.57%	2.13 [1.27-3.57]	1.67E-02	0.507
G0S2	101	6	5.94%	196	23	11.73%	2.11 [0.96-4.60]	0.118	0.526
G2E3	101	1	0.99%	196	7	3.57%	3.70 [0.63-21.75]	0.224	0.596
G3BP2	101	1	0.99%	196	5	2.55%	2.62 [0.43-16.05]	0.383	0.750
GAA	101	3	2.97%	196	16	8.16%	2.90 [1.01-8.34]	0.097	0.507
GAB2	101	8	7.92%	196	16	8.16%	1.03 [0.49-2.17]	0.942	1.000
GABARAPL3	101	2	1.98%	196	6	3.06%	1.56 [0.40-6.08]	0.589	0.869
GABPB2	101	7	6.93%	196	13	6.63%	0.95 [0.43-2.12]	0.923	1.000
GAL3ST3	101	0	0.00%	196	5	2.55%	2.2e+07 [0e+00-Inf]	0.992	1.000
GALK1	101	4	3.96%	196	12	6.12%	1.58 [0.60-4.18]	0.438	0.798
GALNT2	101	6	5.94%	196	19	9.69%	1.70 [0.77-3.78]	0.274	0.643
GALR2	101	4	3.96%	196	13	6.63%	1.72 [0.66-4.51]	0.353	0.722
GALT	101	1	0.99%	196	4	2.04%	2.08 [0.33-13.25]	0.514	0.805
GAL	101	10	9.90%	196	28	14.29%	1.52 [0.80-2.88]	0.286	0.650
GARS	101	0	0.00%	196	7	3.57%	3.2e+07 [0e+00-Inf]	0.992	1.000
GAS2L2	101	2	1.98%	196	4	2.04%	1.03 [0.24-4.35]	0.972	1.000
GAS2	101	3	2.97%	196	2	1.02%	0.34 [0.074-1.53]	0.237	0.599
GAS5	101	5	4.95%	196	13	6.63%	1.36 [0.56-3.32]	0.566	0.852
GATA5	101	6	5.94%	196	11	5.61%	0.94 [0.40-2.23]	0.908	1.000
GATAD2B	101	6	5.94%	196	11	5.61%	0.94 [0.40-2.23]	0.908	1.000
GBAP1	101	9	8.91%	196	16	8.16%	0.91 [0.44-1.86]	0.826	1.000
GBA	101	8	7.92%	196	13	6.63%	0.83 [0.38-1.78]	0.682	0.943
GCGR	101	2	1.98%	196	13	6.63%	3.52 [0.99-12.47]	0.102	0.507
GCK	101	0	0.00%	196	5	2.55%	2.2e+07 [0e+00-Inf]	0.992	1.000
GCNT7	101	7	6.93%	196	13	6.63%	0.95 [0.43-2.12]	0.923	1.000
GDAP1L1	101	3	2.97%	196	3	1.53%	0.51 [0.13-1.98]	0.412	0.774
GDAP1	101	17	16.83%	196	42	21.43%	1.35 [0.80-2.27]	0.348	0.722
GDE1	101	5	4.95%	196	4	2.04%	0.40 [0.13-1.23]	0.179	0.535
GDF5	101	4	3.96%	196	8	4.08%	1.03 [0.37-2.88]	0.960	1.000
GDF6	101	16	15.84%	196	53	27.04%	1.97 [1.17-3.31]	3.23E-02	0.507
GDPD1	101	8	7.92%	196	30	15.31%	2.10 [1.06-4.18]	0.076	0.507
GDPD3	101	5	4.95%	196	4	2.04%	0.40 [0.13-1.23]	0.179	0.535
GDPD4	101	8	7.92%	196	20	10.20%	1.32 [0.64-2.71]	0.525	0.818
GDPD5	101	4	3.96%	196	18	9.18%	2.45 [0.97-6.23]	0.114	0.512
GEM	101	17	16.83%	196	52	26.53%	1.78 [1.07-2.98]	0.063	0.507
GFER	101	6	5.94%	196	5	2.55%	0.41 [0.15-1.15]	0.154	0.535
GGA2	101	5	4.95%	196	2	1.02%	0.20 [0.049-0.80]	0.056	0.507
GGA3	101	5	4.95%	196	13	6.63%	1.36 [0.56-3.32]	0.566	0.852
GGCT	101	0	0.00%	196	7	3.57%	3.2e+07 [0e+00-Inf]	0.992	1.000
GGH	101	16	15.84%	196	33	16.84%	1.08 [0.62-1.86]	0.827	1.000
GGNBP2	101	2	1.98%	196	5	2.55%	1.30 [0.32-5.21]	0.759	0.996
GGPS1	101	7	6.93%	196	20	10.20%	1.53 [0.72-3.24]	0.355	0.722
GGT7	101	4	3.96%	196	6	3.06%	0.77 [0.26-2.26]	0.685	0.943
GH1	101	8	7.92%	196	29	14.80%	2.02 [1.01-4.03]	0.094	0.507
GH2	101	8	7.92%	196	29	14.80%	2.02 [1.01-4.03]	0.094	0.507
GHRHR	101	0	0.00%	196	7	3.57%	3.2e+07 [0e+00-Inf]	0.992	1.000
GHRH	101	5	4.95%	196	3	1.53%	0.30 [0.088-1.01]	0.103	0.507
GINS4	101	15	14.85%	196	31	15.82%	1.08 [0.61-1.89]	0.828	1.000
GIP	101	3	2.97%	196	17	8.67%	3.10 [1.09-8.87]	0.076	0.507
GIT1	101	3	2.97%	196	5	2.55%	0.86 [0.25-2.89]	0.833	1.000
SLX1B	101	4	3.96%	196	3	1.53%	0.38 [0.11-1.35]	0.207	0.573
GJA5	101	6	5.94%	196	12	6.12%	1.03 [0.44-2.41]	0.950	1.000
GJA8	101	6	5.94%	196	12	6.12%	1.03 [0.44-2.41]	0.950	1.000
GJC2	101	6	5.94%	196	21	10.71%	1.90 [0.86-4.19]	0.181	0.535
GJD3	101	4	3.96%	196	6	3.06%	0.77 [0.26-2.26]	0.685	0.943
GLI4	101	17	16.83%	196	51	26.02%	1.74 [1.04-2.90]	0.076	0.507
GLIPR1L1	101	2	1.98%	196	6	3.06%	1.56 [0.40-6.08]	0.589	0.869
GLIPR1L2	101	2	1.98%	196	5	2.55%	1.30 [0.32-5.21]	0.759	0.996
GLIPR1	101	2	1.98%	196	5	2.55%	1.30 [0.32-5.21]	0.759	0.996
GLIS2	101	5	4.95%	196	6	3.06%	0.61 [0.22-1.68]	0.418	0.779
GLRX2	101	5	4.95%	196	19	9.69%	2.06 [0.88-4.83]	0.163	0.535
COLGALT2	101	5	4.95%	196	20	10.20%	2.18 [0.93-5.10]	0.130	0.535

Symbol	CINSARC Low			CINSARC High			CINSARC High vs. Low		
	N tested	N altered	Fq alteration	N tested	N altered	Fq alteration	OR [95%CI]	p-value*	q-value
GLUL	101	5	4.95%	196	19	9.69%	2.06 [0.88-4.83]	0.163	0.535
GLYR1	101	5	4.95%	196	5	2.55%	0.50 [0.17-1.45]	0.286	0.650
GMEB2	101	6	5.94%	196	16	8.16%	1.41 [0.62-3.18]	0.490	0.805
GML	101	16	15.84%	196	49	25.00%	1.77 [1.05-2.99]	0.073	0.507
GNA13	101	8	7.92%	196	23	11.73%	1.55 [0.76-3.14]	0.311	0.680
GNAS-AS1	101	9	8.91%	196	14	7.14%	0.79 [0.38-1.64]	0.590	0.870
GNAS	101	9	8.91%	196	15	7.65%	0.85 [0.41-1.75]	0.707	0.962
GNB4	101	4	3.96%	196	2	1.02%	0.25 [0.059-1.05]	0.113	0.511
GNG13	101	6	5.94%	196	4	2.04%	0.33 [0.11-0.97]	0.092	0.507
GNG4	101	7	6.93%	196	20	10.20%	1.53 [0.72-3.24]	0.355	0.722
GNGT2	101	4	3.96%	196	17	8.67%	2.30 [0.90-5.88]	0.143	0.535
GNPAT	101	6	5.94%	196	19	9.69%	1.70 [0.77-3.78]	0.274	0.643
GNPTG	101	6	5.94%	196	5	2.55%	0.41 [0.15-1.15]	0.154	0.535
GNRHR2	101	5	4.95%	196	10	5.10%	1.03 [0.41-2.60]	0.955	1.000
GNS	101	3	2.97%	196	6	3.06%	1.03 [0.32-3.36]	0.965	1.000
GOLGA2P3Y	101	0	0.00%	196	0	0.00%	1.00 [0e+00-Inf]	1.000	1.000
GOLGA6L5P	101	2	1.98%	196	4	2.04%	1.03 [0.24-4.35]	0.972	1.000
GOLGA6L6	101	3	2.97%	196	4	2.04%	0.68 [0.19-2.43]	0.619	0.898
GOLGA7	101	13	12.87%	196	31	15.82%	1.27 [0.71-2.28]	0.499	0.805
GOLGA8CP	101	3	2.97%	196	4	2.04%	0.68 [0.19-2.43]	0.619	0.898
GOLPH3L	101	9	8.91%	196	17	8.67%	0.97 [0.48-1.97]	0.945	1.000
GOLT1A	101	7	6.93%	196	28	14.29%	2.24 [1.08-4.63]	0.068	0.507
GON4L	101	7	6.93%	196	9	4.59%	0.65 [0.27-1.52]	0.401	0.774
GORAB	101	4	3.96%	196	13	6.63%	1.72 [0.66-4.51]	0.353	0.722
GOSR1	101	2	1.98%	196	3	1.53%	0.77 [0.17-3.50]	0.776	0.996
GOSR2	101	1	0.99%	196	5	2.55%	2.62 [0.43-16.05]	0.383	0.750
GOT1L1	101	10	9.90%	196	51	26.02%	3.20 [1.74-5.89]	1.70E-03	0.507
GP2	101	5	4.95%	196	3	1.53%	0.30 [0.088-1.01]	0.103	0.507
GP5	101	3	2.97%	196	3	1.53%	0.51 [0.13-1.98]	0.412	0.774
GPA33	101	5	4.95%	196	13	6.63%	1.36 [0.56-3.32]	0.566	0.852
GPAA1	101	17	16.83%	196	51	26.02%	1.74 [1.04-2.90]	0.076	0.507
GPATCH2	101	6	5.94%	196	20	10.20%	1.80 [0.81-3.98]	0.224	0.596
GPATCH4	101	6	5.94%	196	8	4.08%	0.67 [0.27-1.68]	0.476	0.805
GPIHBP1	101	16	15.84%	196	48	24.49%	1.72 [1.02-2.91]	0.088	0.507
GPNMB	101	0	0.00%	196	6	3.06%	2.7e+07 [0e+00-Inf]	0.992	1.000
GPR137B	101	7	6.93%	196	20	10.20%	1.53 [0.72-3.24]	0.355	0.722
GPR139	101	5	4.95%	196	3	1.53%	0.30 [0.088-1.01]	0.103	0.507
GPR142	101	7	6.93%	196	14	7.14%	1.03 [0.47-2.28]	0.946	1.000
GPR152	101	11	10.89%	196	20	10.20%	0.93 [0.48-1.79]	0.854	1.000
GPR161	101	4	3.96%	196	12	6.12%	1.58 [0.60-4.18]	0.438	0.798
GPR171	101	3	2.97%	196	2	1.02%	0.34 [0.074-1.53]	0.237	0.599
SLC52A2	101	18	17.82%	196	49	25.00%	1.54 [0.93-2.55]	0.163	0.535
GPR179	101	5	4.95%	196	8	4.08%	0.82 [0.31-2.13]	0.729	0.986
GPR20	101	18	17.82%	196	50	25.51%	1.58 [0.95-2.62]	0.137	0.535
GPR25	101	7	6.93%	196	24	12.24%	1.87 [0.90-3.92]	0.161	0.535
GPR37L1	101	8	7.92%	196	24	12.24%	1.62 [0.80-3.28]	0.258	0.635
GPR52	101	5	4.95%	196	14	7.14%	1.48 [0.61-3.57]	0.467	0.805
GPR87	101	3	2.97%	196	2	1.02%	0.34 [0.074-1.53]	0.237	0.599
GPR89A	101	5	4.95%	196	10	5.10%	1.03 [0.41-2.60]	0.955	1.000
GPR89B	101	6	5.94%	196	12	6.12%	1.03 [0.44-2.41]	0.950	1.000
GPRC5B	101	5	4.95%	196	3	1.53%	0.30 [0.088-1.01]	0.103	0.507
GPRC5C	101	7	6.93%	196	13	6.63%	0.95 [0.43-2.12]	0.923	1.000
GPS1	101	2	1.98%	196	14	7.14%	3.81 [1.08-13.43]	0.081	0.507
GPT2	101	1	0.99%	196	4	2.04%	2.08 [0.33-13.25]	0.514	0.805
GPT	101	17	16.83%	196	49	25.00%	1.65 [0.98-2.76]	0.111	0.511
GRAPL	101	5	4.95%	196	1	0.51%	0.098 [0.016-0.60]	3.55E-02	0.507
GRB2	101	5	4.95%	196	11	5.61%	1.14 [0.46-2.84]	0.811	1.000
GRB7	101	6	5.94%	196	12	6.12%	1.03 [0.44-2.41]	0.950	1.000
GREM2	101	5	4.95%	196	19	9.69%	2.06 [0.88-4.83]	0.163	0.535
GRHL2	101	18	17.82%	196	55	28.06%	1.80 [1.09-2.97]	0.054	0.507
GRIN2A	101	5	4.95%	196	4	2.04%	0.40 [0.13-1.23]	0.179	0.535
GRIN2C	101	5	4.95%	196	13	6.63%	1.36 [0.56-3.32]	0.566	0.852
GRINA	101	17	16.83%	196	51	26.02%	1.74 [1.04-2.90]	0.076	0.507
GRIP1	101	2	1.98%	196	10	5.10%	2.66 [0.73-9.67]	0.212	0.586
GRM5	101	1	0.99%	196	4	2.04%	2.08 [0.33-13.25]	0.514	0.805
GRP	101	1	0.99%	196	4	2.04%	2.08 [0.33-13.25]	0.514	0.805
GSDMA	101	5	4.95%	196	10	5.10%	1.03 [0.41-2.60]	0.955	1.000
GSDMB	101	5	4.95%	196	12	6.12%	1.25 [0.51-3.08]	0.681	0.943
GSDMC	101	15	14.85%	196	57	29.08%	2.35 [1.39-3.99]	7.74E-03	0.507
GSDMD	101	17	16.83%	196	51	26.02%	1.74 [1.04-2.90]	0.076	0.507
GSG1L	101	5	4.95%	196	4	2.04%	0.40 [0.13-1.23]	0.179	0.535

Symbol	CINSARC Low			CINSARC High			CINSARC High vs. Low		
	N tested	N altered	Fq alteration	N tested	N altered	Fq alteration	OR [95%CI]	p-value*	q-value
GSPT1	101	5	4.95%	196	3	1.53%	0.30 [0.088-1.01]	0.103	0.507
GSR	101	2	1.98%	196	6	3.06%	1.56 [0.40-6.08]	0.589	0.869
GSS	101	4	3.96%	196	8	4.08%	1.03 [0.37-2.88]	0.960	1.000
GSTM1	101	20	19.80%	196	32	16.33%	0.79 [0.47-1.33]	0.456	0.805
GSTM2	101	5	4.95%	196	1	0.51%	0.098 [0.016-0.60]	3.55E-02	0.507
GSTP1	101	9	8.91%	196	20	10.20%	1.16 [0.58-2.32]	0.722	0.980
GSTT1	101	4	3.96%	196	8	4.08%	1.03 [0.37-2.88]	0.960	1.000
GTF2E2	101	2	1.98%	196	5	2.55%	1.30 [0.32-5.21]	0.759	0.996
GTF2H2	101	5	4.95%	196	6	3.06%	0.61 [0.22-1.68]	0.418	0.779
GTF3C1	101	5	4.95%	196	3	1.53%	0.30 [0.088-1.01]	0.103	0.507
MTG2	101	7	6.93%	196	12	6.12%	0.88 [0.39-1.97]	0.788	0.996
GTSF1L	101	2	1.98%	196	4	2.04%	1.03 [0.24-4.35]	0.972	1.000
GUCY2EP	101	6	5.94%	196	17	8.67%	1.50 [0.67-3.38]	0.407	0.774
GUK1	101	6	5.94%	196	21	10.71%	1.90 [0.86-4.19]	0.181	0.535
H3F3A	101	6	5.94%	196	19	9.69%	1.70 [0.77-3.78]	0.274	0.643
H3F3B	101	4	3.96%	196	12	6.12%	1.58 [0.60-4.18]	0.438	0.798
HACE1	101	0	0.00%	196	5	2.55%	2.2e+07 [0e+00-Inf]	0.992	1.000
HAGHL	101	6	5.94%	196	4	2.04%	0.33 [0.11-0.97]	0.092	0.507
HAGH	101	6	5.94%	196	4	2.04%	0.33 [0.11-0.97]	0.092	0.507
HAPLN2	101	6	5.94%	196	8	4.08%	0.67 [0.27-1.68]	0.476	0.805
HAPLN3	101	1	0.99%	196	5	2.55%	2.62 [0.43-16.05]	0.383	0.750
HAR1A	101	8	7.92%	196	13	6.63%	0.83 [0.38-1.78]	0.682	0.943
HAR1B	101	8	7.92%	196	13	6.63%	0.83 [0.38-1.78]	0.682	0.943
HAS1	101	1	0.99%	196	4	2.04%	2.08 [0.33-13.25]	0.514	0.805
HAS2-AS1	101	15	14.85%	196	53	27.04%	2.12 [1.25-3.61]	1.95E-02	0.507
HAS2	101	15	14.85%	196	54	27.55%	2.18 [1.28-3.70]	1.56E-02	0.507
HAX1	101	7	6.93%	196	12	6.12%	0.88 [0.39-1.97]	0.788	0.996
HBA1	101	7	6.93%	196	4	2.04%	0.28 [0.098-0.80]	4.63E-02	0.507
HBA2	101	7	6.93%	196	4	2.04%	0.28 [0.098-0.80]	4.63E-02	0.507
HBM	101	7	6.93%	196	4	2.04%	0.28 [0.098-0.80]	4.63E-02	0.507
HBQ1	101	7	6.93%	196	4	2.04%	0.28 [0.098-0.80]	4.63E-02	0.507
HBZ	101	7	6.93%	196	4	2.04%	0.28 [0.098-0.80]	4.63E-02	0.507
HCFC1R1	101	6	5.94%	196	4	2.04%	0.33 [0.11-0.97]	0.092	0.507
HCN3	101	7	6.93%	196	14	7.14%	1.03 [0.47-2.28]	0.946	1.000
HDDC3	101	1	0.99%	196	4	2.04%	2.08 [0.33-13.25]	0.514	0.805
HDGF	101	7	6.93%	196	8	4.08%	0.57 [0.24-1.37]	0.294	0.650
HEATR1	101	7	6.93%	196	19	9.69%	1.44 [0.68-3.07]	0.427	0.791
HEATR4	101	3	2.97%	196	7	3.57%	1.21 [0.38-3.83]	0.786	0.996
HEATR5A	101	1	0.99%	196	9	4.59%	4.81 [0.84-27.58]	0.139	0.535
HEATR6	101	7	6.93%	196	33	16.84%	2.72 [1.33-5.57]	2.17E-02	0.507
MROH1	101	18	17.82%	196	50	25.51%	1.58 [0.95-2.62]	0.137	0.535
HECTD1	101	0	0.00%	196	7	3.57%	3.2e+07 [0e+00-Inf]	0.992	1.000
HELB	101	2	1.98%	196	9	4.59%	2.38 [0.65-8.76]	0.273	0.643
HELZ	101	10	9.90%	196	29	14.80%	1.58 [0.83-3.00]	0.240	0.606
HERC2P4	101	4	3.96%	196	3	1.53%	0.38 [0.11-1.35]	0.207	0.573
HES1	101	3	2.97%	196	3	1.53%	0.51 [0.13-1.98]	0.412	0.774
HEXA	101	1	0.99%	196	4	2.04%	2.08 [0.33-13.25]	0.514	0.805
HEY1	101	17	16.83%	196	46	23.47%	1.52 [0.90-2.54]	0.187	0.551
HGSNAT	101	12	11.88%	196	24	12.24%	1.03 [0.56-1.92]	0.928	1.000
HGS	101	2	1.98%	196	12	6.12%	3.23 [0.90-11.53]	0.130	0.535
HHAT	101	6	5.94%	196	20	10.20%	1.80 [0.81-3.98]	0.224	0.596
HHIPL2	101	6	5.94%	196	18	9.18%	1.60 [0.72-3.57]	0.335	0.716
HHLA1	101	15	14.85%	196	55	28.06%	2.24 [1.32-3.80]	1.24E-02	0.507
HIBADH	101	0	0.00%	196	7	3.57%	3.2e+07 [0e+00-Inf]	0.992	1.000
HILS1	101	5	4.95%	196	17	8.67%	1.82 [0.77-4.32]	0.252	0.623
HIRIP3	101	5	4.95%	196	4	2.04%	0.40 [0.13-1.23]	0.179	0.535
HIST2H2AA3	101	8	7.92%	196	14	7.14%	0.89 [0.42-1.91]	0.808	1.000
HIST2H2AB	101	6	5.94%	196	14	7.14%	1.22 [0.53-2.79]	0.696	0.951
HIST2H2AC	101	6	5.94%	196	14	7.14%	1.22 [0.53-2.79]	0.696	0.951
HIST2H2BE	101	6	5.94%	196	14	7.14%	1.22 [0.53-2.79]	0.696	0.951
HIST2H2BF	101	7	6.93%	196	12	6.12%	0.88 [0.39-1.97]	0.788	0.996
HIST2H3C	101	6	5.94%	196	14	7.14%	1.22 [0.53-2.79]	0.696	0.951
HIST2H3D	101	7	6.93%	196	12	6.12%	0.88 [0.39-1.97]	0.788	0.996
HIST2H4A	101	8	7.92%	196	14	7.14%	0.89 [0.42-1.91]	0.808	1.000
HIST3H2A	101	7	6.93%	196	21	10.71%	1.61 [0.76-3.40]	0.294	0.650
HIST3H2BB	101	7	6.93%	196	21	10.71%	1.61 [0.76-3.40]	0.294	0.650
HIST3H3	101	7	6.93%	196	21	10.71%	1.61 [0.76-3.40]	0.294	0.650
HLA-DRB1	101	17	16.83%	196	35	17.86%	1.07 [0.63-1.83]	0.826	1.000
HLA-DRB5	101	24	23.76%	196	49	25.00%	1.07 [0.67-1.71]	0.814	1.000
HLA-DRB6	101	17	16.83%	196	33	16.84%	1.00 [0.58-1.71]	0.999	1.000
HLF	101	8	7.92%	196	21	10.71%	1.39 [0.68-2.85]	0.444	0.805

Symbol	CINSARC Low			CINSARC High			CINSARC High vs. Low		
	N tested	N altered	Fq alteration	N tested	N altered	Fq alteration	OR [95%CI]	p-value*	q-value
HLX	101	6	5.94%	196	18	9.18%	1.60 [0.72-3.57]	0.335	0.716
HMCN1	101	5	4.95%	196	17	8.67%	1.82 [0.77-4.32]	0.252	0.623
HMGA2	101	1	0.99%	196	7	3.57%	3.70 [0.63-21.75]	0.224	0.596
HMGB1	101	1	0.99%	196	5	2.55%	2.62 [0.43-16.05]	0.383	0.750
HMGB3P1	101	4	3.96%	196	6	3.06%	0.77 [0.26-2.26]	0.685	0.943
HMOX2	101	5	4.95%	196	6	3.06%	0.61 [0.22-1.68]	0.418	0.779
HMSD	101	3	2.97%	196	3	1.53%	0.51 [0.13-1.98]	0.412	0.774
HNF1B	101	3	2.97%	196	7	3.57%	1.21 [0.38-3.83]	0.786	0.996
HNF4A	101	2	1.98%	196	4	2.04%	1.03 [0.24-4.35]	0.972	1.000
HNF4G	101	14	13.86%	196	41	20.92%	1.64 [0.94-2.86]	0.141	0.535
HNRNPA2B1	101	0	0.00%	196	5	2.55%	2.2e+07 [0e+00-Inf]	0.992	1.000
HNRNPU	101	5	4.95%	196	18	9.18%	1.94 [0.82-4.58]	0.203	0.570
HOMER2	101	1	0.99%	196	4	2.04%	2.08 [0.33-13.25]	0.514	0.805
HOOK3	101	13	12.87%	196	26	13.27%	1.04 [0.57-1.88]	0.924	1.000
HORMAD1	101	8	7.92%	196	16	8.16%	1.03 [0.49-2.17]	0.942	1.000
HOXA10	101	0	0.00%	196	6	3.06%	2.7e+07 [0e+00-Inf]	0.992	1.000
HOXA11-AS	101	0	0.00%	196	6	3.06%	2.7e+07 [0e+00-Inf]	0.992	1.000
HOXA11	101	0	0.00%	196	6	3.06%	2.7e+07 [0e+00-Inf]	0.992	1.000
HOXA13	101	0	0.00%	196	6	3.06%	2.7e+07 [0e+00-Inf]	0.992	1.000
HOXA1	101	0	0.00%	196	6	3.06%	2.7e+07 [0e+00-Inf]	0.992	1.000
HOXA2	101	0	0.00%	196	6	3.06%	2.7e+07 [0e+00-Inf]	0.992	1.000
HOXA3	101	0	0.00%	196	6	3.06%	2.7e+07 [0e+00-Inf]	0.992	1.000
HOXA4	101	0	0.00%	196	6	3.06%	2.7e+07 [0e+00-Inf]	0.992	1.000
HOXA5	101	0	0.00%	196	6	3.06%	2.7e+07 [0e+00-Inf]	0.992	1.000
HOXA6	101	0	0.00%	196	6	3.06%	2.7e+07 [0e+00-Inf]	0.992	1.000
HOXA7	101	0	0.00%	196	6	3.06%	2.7e+07 [0e+00-Inf]	0.992	1.000
HOXA9	101	0	0.00%	196	6	3.06%	2.7e+07 [0e+00-Inf]	0.992	1.000
HOXB13	101	2	1.98%	196	17	8.67%	4.70 [1.35-16.35]	4.11E-02	0.507
HOXB1	101	2	1.98%	196	16	8.16%	4.40 [1.26-15.37]	0.051	0.507
HOXB2	101	2	1.98%	196	16	8.16%	4.40 [1.26-15.37]	0.051	0.507
HOXB3	101	2	1.98%	196	16	8.16%	4.40 [1.26-15.37]	0.051	0.507
HOXB4	101	2	1.98%	196	15	7.65%	4.10 [1.17-14.39]	0.064	0.507
HOXB5	101	2	1.98%	196	16	8.16%	4.40 [1.26-15.37]	0.051	0.507
HOXB6	101	2	1.98%	196	16	8.16%	4.40 [1.26-15.37]	0.051	0.507
HOXB7	101	2	1.98%	196	17	8.67%	4.70 [1.35-16.35]	4.11E-02	0.507
HOXB8	101	2	1.98%	196	17	8.67%	4.70 [1.35-16.35]	4.11E-02	0.507
HOXB9	101	2	1.98%	196	17	8.67%	4.70 [1.35-16.35]	4.11E-02	0.507
HPS3	101	3	2.97%	196	2	1.02%	0.34 [0.074-1.53]	0.237	0.599
HPYR1	101	15	14.85%	196	55	28.06%	2.24 [1.32-3.80]	1.24E-02	0.507
HRH3	101	7	6.93%	196	12	6.12%	0.88 [0.39-1.97]	0.788	0.996
RBFOX3	101	3	2.97%	196	15	7.65%	2.71 [0.94-7.82]	0.122	0.534
HRNR	101	7	6.93%	196	13	6.63%	0.95 [0.43-2.12]	0.923	1.000
HS3ST2	101	5	4.95%	196	2	1.02%	0.20 [0.049-0.80]	0.056	0.507
HS3ST4	101	5	4.95%	196	3	1.53%	0.30 [0.088-1.01]	0.103	0.507
HS3ST6	101	6	5.94%	196	5	2.55%	0.41 [0.15-1.15]	0.154	0.535
HSD11B1	101	6	5.94%	196	22	11.22%	2.00 [0.91-4.39]	0.146	0.535
HSD17B7	101	5	4.95%	196	14	7.14%	1.48 [0.61-3.57]	0.467	0.805
HSD3B7	101	4	3.96%	196	6	3.06%	0.77 [0.26-2.26]	0.685	0.943
HSF1	101	18	17.82%	196	50	25.51%	1.58 [0.95-2.62]	0.137	0.535
HSF5	101	7	6.93%	196	28	14.29%	2.24 [1.08-4.63]	0.068	0.507
HSFY2	101	0	0.00%	196	0	0.00%	1.00 [0e+00-Inf]	1.000	1.000
HSPA6	101	7	6.93%	196	19	9.69%	1.44 [0.68-3.07]	0.427	0.791
HSPA7	101	9	8.91%	196	22	11.22%	1.29 [0.65-2.56]	0.538	0.835
HTR3C	101	3	2.97%	196	2	1.02%	0.34 [0.074-1.53]	0.237	0.599
HTR3D	101	3	2.97%	196	2	1.02%	0.34 [0.074-1.53]	0.237	0.599
HTR3E	101	3	2.97%	196	2	1.02%	0.34 [0.074-1.53]	0.237	0.599
HTRA4	101	12	11.88%	196	39	19.90%	1.84 [1.03-3.31]	0.086	0.507
IARS2	101	6	5.94%	196	20	10.20%	1.80 [0.81-3.98]	0.224	0.596
ICAM2	101	8	7.92%	196	29	14.80%	2.02 [1.01-4.03]	0.094	0.507
IDH2	101	1	0.99%	196	8	4.08%	4.26 [0.73-24.65]	0.175	0.535
IDO1	101	13	12.87%	196	32	16.33%	1.32 [0.74-2.37]	0.432	0.798
IDO2	101	13	12.87%	196	32	16.33%	1.32 [0.74-2.37]	0.432	0.798
IER5	101	5	4.95%	196	19	9.69%	2.06 [0.88-4.83]	0.163	0.535
IFI16	101	6	5.94%	196	8	4.08%	0.67 [0.27-1.68]	0.476	0.805
IFNG	101	2	1.98%	196	15	7.65%	4.10 [1.17-14.39]	0.064	0.507
IFT140	101	6	5.94%	196	5	2.55%	0.41 [0.15-1.15]	0.154	0.535
IFT20	101	4	3.96%	196	7	3.57%	0.90 [0.31-2.57]	0.867	1.000
IFT52	101	2	1.98%	196	4	2.04%	1.03 [0.24-4.35]	0.972	1.000
IGF1R	101	1	0.99%	196	9	4.59%	4.81 [0.84-27.58]	0.139	0.535
IGF2BP1	101	3	2.97%	196	17	8.67%	3.10 [1.09-8.87]	0.076	0.507
IGF2BP2	101	3	2.97%	196	2	1.02%	0.34 [0.074-1.53]	0.237	0.599

Symbol	CINSARC Low			CINSARC High			CINSARC High vs. Low		
	N tested	N altered	Fq alteration	N tested	N altered	Fq alteration	OR [95%CI]	p-value*	q-value
IGF2BP3	101	0	0.00%	196	6	3.06%	2.7e+07 [0e+00-Inf]	0.992	1.000
IGFALS	101	6	5.94%	196	5	2.55%	0.41 [0.15-1.15]	0.154	0.535
IGFBP4	101	4	3.96%	196	5	2.55%	0.63 [0.21-1.95]	0.505	0.805
IGFN1	101	6	5.94%	196	24	12.24%	2.21 [1.01-4.82]	0.095	0.507
IGHMBP2	101	15	14.85%	196	37	18.88%	1.33 [0.77-2.31]	0.388	0.757
IGSF22	101	1	0.99%	196	4	2.04%	2.08 [0.33-13.25]	0.514	0.805
IGSF6	101	6	5.94%	196	2	1.02%	0.16 [0.042-0.64]	2.82E-02	0.507
IGSF8	101	6	5.94%	196	11	5.61%	0.94 [0.40-2.23]	0.908	1.000
IGSF9	101	6	5.94%	196	11	5.61%	0.94 [0.40-2.23]	0.908	1.000
IKBKB	101	14	13.86%	196	31	15.82%	1.17 [0.66-2.07]	0.656	0.931
IKBKE	101	6	5.94%	196	24	12.24%	2.21 [1.01-4.82]	0.095	0.507
IKZF3	101	6	5.94%	196	12	6.12%	1.03 [0.44-2.41]	0.950	1.000
IL10	101	6	5.94%	196	25	12.76%	2.31 [1.06-5.03]	0.076	0.507
IL11RA	101	1	0.99%	196	4	2.04%	2.08 [0.33-13.25]	0.514	0.805
IL11	101	1	0.99%	196	4	2.04%	2.08 [0.33-13.25]	0.514	0.805
IL18BP	101	4	3.96%	196	5	2.55%	0.63 [0.21-1.95]	0.505	0.805
IL19	101	6	5.94%	196	25	12.76%	2.31 [1.06-5.03]	0.076	0.507
IL1RAP	101	2	1.98%	196	3	1.53%	0.77 [0.17-3.50]	0.776	0.996
IL20	101	7	6.93%	196	25	12.76%	1.96 [0.94-4.09]	0.131	0.535
IL21R	101	5	4.95%	196	3	1.53%	0.30 [0.088-1.01]	0.103	0.507
IL22	101	2	1.98%	196	15	7.65%	4.10 [1.17-14.39]	0.064	0.507
IL24	101	7	6.93%	196	25	12.76%	1.96 [0.94-4.09]	0.131	0.535
IL26	101	2	1.98%	196	15	7.65%	4.10 [1.17-14.39]	0.064	0.507
IL27	101	5	4.95%	196	3	1.53%	0.30 [0.088-1.01]	0.103	0.507
IL32	101	7	6.93%	196	4	2.04%	0.28 [0.098-0.80]	4.63E-02	0.507
IL4R	101	5	4.95%	196	3	1.53%	0.30 [0.088-1.01]	0.103	0.507
IL6R	101	7	6.93%	196	11	5.61%	0.80 [0.35-1.82]	0.652	0.928
IL6	101	0	0.00%	196	6	3.06%	2.7e+07 [0e+00-Inf]	0.992	1.000
IL7	101	14	13.86%	196	47	23.98%	1.96 [1.13-3.39]	4.33E-02	0.507
ILDR2	101	5	4.95%	196	13	6.63%	1.36 [0.56-3.32]	0.566	0.852
ILF2	101	7	6.93%	196	12	6.12%	0.88 [0.39-1.97]	0.788	0.996
IMPA1	101	18	17.82%	196	46	23.47%	1.41 [0.85-2.35]	0.263	0.643
IMPAD1	101	14	13.86%	196	29	14.80%	1.08 [0.61-1.92]	0.828	1.000
INMT	101	0	0.00%	196	7	3.57%	3.2e+07 [0e+00-Inf]	0.992	1.000
INO80E	101	5	4.95%	196	4	2.04%	0.40 [0.13-1.23]	0.179	0.535
INPPL1	101	5	4.95%	196	8	4.08%	0.82 [0.31-2.13]	0.729	0.986
INSC	101	1	0.99%	196	4	2.04%	2.08 [0.33-13.25]	0.514	0.805
INSM2	101	0	0.00%	196	9	4.59%	4.1e+07 [0e+00-Inf]	0.992	1.000
INSRR	101	7	6.93%	196	8	4.08%	0.57 [0.24-1.37]	0.294	0.650
INTS2	101	9	8.91%	196	32	16.33%	1.99 [1.03-3.85]	0.084	0.507
INTS3	101	7	6.93%	196	12	6.12%	0.88 [0.39-1.97]	0.788	0.996
INTS4	101	10	9.90%	196	21	10.71%	1.09 [0.56-2.13]	0.828	1.000
INTS7	101	6	5.94%	196	20	10.20%	1.80 [0.81-3.98]	0.224	0.596
INTS8	101	16	15.84%	196	54	27.55%	2.02 [1.20-3.40]	2.60E-02	0.507
IPO9	101	7	6.93%	196	24	12.24%	1.87 [0.90-3.92]	0.161	0.535
IQCG	101	3	2.97%	196	4	2.04%	0.68 [0.19-2.43]	0.619	0.898
IQCK	101	5	4.95%	196	3	1.53%	0.30 [0.088-1.01]	0.103	0.507
IQGAP1	101	2	1.98%	196	5	2.55%	1.30 [0.32-5.21]	0.759	0.996
IQGAP3	101	6	5.94%	196	8	4.08%	0.67 [0.27-1.68]	0.476	0.805
IRAK3	101	2	1.98%	196	9	4.59%	2.38 [0.65-8.76]	0.273	0.643
IRF2BP2	101	7	6.93%	196	20	10.20%	1.53 [0.72-3.24]	0.355	0.722
IRF6	101	6	5.94%	196	22	11.22%	2.00 [0.91-4.39]	0.146	0.535
IRS2	101	0	0.00%	196	5	2.55%	2.2e+07 [0e+00-Inf]	0.992	1.000
ISG20L2	101	7	6.93%	196	8	4.08%	0.57 [0.24-1.37]	0.294	0.650
ISG20	101	0	0.00%	196	6	3.06%	2.7e+07 [0e+00-Inf]	0.992	1.000
ISOC2	101	2	1.98%	196	4	2.04%	1.03 [0.24-4.35]	0.972	1.000
ITCH	101	3	2.97%	196	3	1.53%	0.51 [0.13-1.98]	0.412	0.774
ITGA10	101	5	4.95%	196	10	5.10%	1.03 [0.41-2.60]	0.955	1.000
ITGA3	101	4	3.96%	196	17	8.67%	2.30 [0.90-5.88]	0.143	0.535
ITGAD	101	4	3.96%	196	4	2.04%	0.51 [0.16-1.65]	0.342	0.716
ITGAL	101	4	3.96%	196	5	2.55%	0.63 [0.21-1.95]	0.505	0.805
ITGAM	101	4	3.96%	196	5	2.55%	0.63 [0.21-1.95]	0.505	0.805
ITGAX	101	4	3.96%	196	4	2.04%	0.51 [0.16-1.65]	0.342	0.716
ITGB3	101	1	0.99%	196	7	3.57%	3.70 [0.63-21.75]	0.224	0.596
ITGB4	101	4	3.96%	196	12	6.12%	1.58 [0.60-4.18]	0.438	0.798
ITLN1	101	6	5.94%	196	16	8.16%	1.41 [0.62-3.18]	0.490	0.805
ITLN2	101	6	5.94%	196	17	8.67%	1.50 [0.67-3.38]	0.407	0.774
ITPKB	101	6	5.94%	196	19	9.69%	1.70 [0.77-3.78]	0.274	0.643
ITPRIPL2	101	5	4.95%	196	4	2.04%	0.40 [0.13-1.23]	0.179	0.535
IVL	101	7	6.93%	196	11	5.61%	0.80 [0.35-1.82]	0.652	0.928
IVNS1ABP	101	5	4.95%	196	19	9.69%	2.06 [0.88-4.83]	0.163	0.535

Symbol	CINSARC Low			CINSARC High			CINSARC High vs. Low		
	N tested	N altered	Fq alteration	N tested	N altered	Fq alteration	OR [95%CI]	p-value*	q-value
JAZF1	101	0	0.00%	196	7	3.57%	3.2e+07 [0e+00-Inf]	0.992	1.000
JMJD4	101	6	5.94%	196	21	10.71%	1.90 [0.86-4.19]	0.181	0.535
KDM8	101	5	4.95%	196	3	1.53%	0.30 [0.088-1.01]	0.103	0.507
JMJD6	101	3	2.97%	196	13	6.63%	2.32 [0.79-6.79]	0.197	0.570
JMJD8	101	6	5.94%	196	4	2.04%	0.33 [0.11-0.97]	0.092	0.507
JPH1	101	17	16.83%	196	42	21.43%	1.35 [0.80-2.27]	0.348	0.722
JPH2	101	3	2.97%	196	3	1.53%	0.51 [0.13-1.98]	0.412	0.774
JRK	101	16	15.84%	196	49	25.00%	1.77 [1.05-2.99]	0.073	0.507
JTB	101	7	6.93%	196	13	6.63%	0.95 [0.43-2.12]	0.923	1.000
KATNAL1	101	1	0.99%	196	4	2.04%	2.08 [0.33-13.25]	0.514	0.805
KBTBD2	101	0	0.00%	196	7	3.57%	3.2e+07 [0e+00-Inf]	0.992	1.000
KCNB1	101	7	6.93%	196	8	4.08%	0.57 [0.24-1.37]	0.294	0.650
KCNB2	101	15	14.85%	196	39	19.90%	1.42 [0.82-2.46]	0.287	0.650
KCNC1	101	1	0.99%	196	4	2.04%	2.08 [0.33-13.25]	0.514	0.805
KCNC2	101	2	1.98%	196	7	3.57%	1.83 [0.48-6.96]	0.455	0.805
KCNE3	101	6	5.94%	196	12	6.12%	1.03 [0.44-2.41]	0.950	1.000
KCNG1	101	6	5.94%	196	13	6.63%	1.12 [0.49-2.60]	0.817	1.000
KCNH1	101	7	6.93%	196	20	10.20%	1.53 [0.72-3.24]	0.355	0.722
KCNH6	101	8	7.92%	196	30	15.31%	2.10 [1.06-4.18]	0.076	0.507
KCNJ10	101	6	5.94%	196	11	5.61%	0.94 [0.40-2.23]	0.908	1.000
KCNJ11	101	1	0.99%	196	4	2.04%	2.08 [0.33-13.25]	0.514	0.805
KCNJ16	101	4	3.96%	196	21	10.71%	2.91 [1.16-7.31]	0.056	0.507
KCNJ2	101	4	3.96%	196	20	10.20%	2.76 [1.09-6.95]	0.071	0.507
KCNJ9	101	6	5.94%	196	11	5.61%	0.94 [0.40-2.23]	0.908	1.000
KCNK15	101	4	3.96%	196	4	2.04%	0.51 [0.16-1.65]	0.342	0.716
KCNK1	101	6	5.94%	196	20	10.20%	1.80 [0.81-3.98]	0.224	0.596
KCNK2	101	6	5.94%	196	20	10.20%	1.80 [0.81-3.98]	0.224	0.596
KCNK9	101	17	16.83%	196	50	25.51%	1.69 [1.01-2.83]	0.092	0.507
KCNMB2	101	3	2.97%	196	3	1.53%	0.51 [0.13-1.98]	0.412	0.774
KCNMB3	101	4	3.96%	196	3	1.53%	0.38 [0.11-1.35]	0.207	0.573
KCNMB4	101	3	2.97%	196	15	7.65%	2.71 [0.94-7.82]	0.122	0.534
KCNN3	101	8	7.92%	196	13	6.63%	0.83 [0.38-1.78]	0.682	0.943
KCNQ2	101	6	5.94%	196	14	7.14%	1.22 [0.53-2.79]	0.696	0.951
KCNQ3	101	14	13.86%	196	55	28.06%	2.42 [1.41-4.16]	7.11E-03	0.507
KCNS1	101	5	4.95%	196	2	1.02%	0.20 [0.049-0.80]	0.056	0.507
KCNS2	101	16	15.84%	196	53	27.04%	1.97 [1.17-3.31]	3.23E-02	0.507
KCNT2	101	5	4.95%	196	19	9.69%	2.06 [0.88-4.83]	0.163	0.535
KCNU1	101	9	8.91%	196	37	18.88%	2.38 [1.24-4.55]	2.79E-02	0.507
KCNV1	101	16	15.84%	196	54	27.55%	2.02 [1.20-3.40]	2.60E-02	0.507
KCTD13	101	5	4.95%	196	4	2.04%	0.40 [0.13-1.23]	0.179	0.535
KCTD14	101	9	8.91%	196	19	9.69%	1.10 [0.55-2.21]	0.827	1.000
KCTD21	101	9	8.91%	196	18	9.18%	1.03 [0.51-2.09]	0.938	1.000
KCTD2	101	5	4.95%	196	12	6.12%	1.25 [0.51-3.08]	0.681	0.943
KCTD3	101	6	5.94%	196	20	10.20%	1.80 [0.81-3.98]	0.224	0.596
KCTD5	101	6	5.94%	196	5	2.55%	0.41 [0.15-1.15]	0.154	0.535
KDM2A	101	11	10.89%	196	16	8.16%	0.73 [0.37-1.43]	0.440	0.800
KDM5B	101	7	6.93%	196	27	13.78%	2.15 [1.03-4.45]	0.085	0.507
KDM5D	101	0	0.00%	196	0	0.00%	1.00 [0e+00-Inf]	1.000	1.000
KDSR	101	3	2.97%	196	2	1.02%	0.34 [0.074-1.53]	0.237	0.599
KEL	101	2	1.98%	196	4	2.04%	1.03 [0.24-4.35]	0.972	1.000
KERA	101	0	0.00%	196	5	2.55%	2.2e+07 [0e+00-Inf]	0.992	1.000
KHDRBS3	101	16	15.84%	196	51	26.02%	1.87 [1.11-3.15]	4.89E-02	0.507
KIAA0040	101	5	4.95%	196	16	8.16%	1.71 [0.72-4.07]	0.311	0.680
KIAA0087	101	0	0.00%	196	5	2.55%	2.2e+07 [0e+00-Inf]	0.992	1.000
KIAA0100	101	3	2.97%	196	8	4.08%	1.39 [0.45-4.31]	0.632	0.909
SPIDR	101	13	12.87%	196	28	14.29%	1.13 [0.62-2.04]	0.738	0.996
KIAA0391	101	0	0.00%	196	9	4.59%	4.1e+07 [0e+00-Inf]	0.992	1.000
TTI1	101	5	4.95%	196	3	1.53%	0.30 [0.088-1.01]	0.103	0.507
KIAA0556	101	5	4.95%	196	3	1.53%	0.30 [0.088-1.01]	0.103	0.507
CLUHP3	101	4	3.96%	196	4	2.04%	0.51 [0.16-1.65]	0.342	0.716
ZSWIM8	101	0	0.00%	196	5	2.55%	2.2e+07 [0e+00-Inf]	0.992	1.000
MAP10	101	6	5.94%	196	20	10.20%	1.80 [0.81-3.98]	0.224	0.596
KIAA1614	101	5	4.95%	196	19	9.69%	2.06 [0.88-4.83]	0.163	0.535
KIAA1755	101	4	3.96%	196	4	2.04%	0.51 [0.16-1.65]	0.342	0.716
KIF14	101	6	5.94%	196	24	12.24%	2.21 [1.01-4.82]	0.095	0.507
KIF19	101	6	5.94%	196	14	7.14%	1.22 [0.53-2.79]	0.696	0.951
KIF21B	101	6	5.94%	196	24	12.24%	2.21 [1.01-4.82]	0.095	0.507
KIF22	101	5	4.95%	196	3	1.53%	0.30 [0.088-1.01]	0.103	0.507
KIF26B	101	4	3.96%	196	17	8.67%	2.30 [0.90-5.88]	0.143	0.535
KIF2B	101	7	6.93%	196	12	6.12%	0.88 [0.39-1.97]	0.788	0.996
KIF7	101	1	0.99%	196	7	3.57%	3.70 [0.63-21.75]	0.224	0.596

Symbol	CINSARC Low			CINSARC High			CINSARC High vs. Low		
	N tested	N altered	Fq alteration	N tested	N altered	Fq alteration	OR [95%CI]	p-value*	q-value
KIFAP3	101	5	4.95%	196	12	6.12%	1.25 [0.51-3.08]	0.681	0.943
KIFC2	101	18	17.82%	196	49	25.00%	1.54 [0.93-2.55]	0.163	0.535
KIR2DL1	101	3	2.97%	196	6	3.06%	1.03 [0.32-3.36]	0.965	1.000
KIR2DL3	101	1	0.99%	196	4	2.04%	2.08 [0.33-13.25]	0.514	0.805
KIR2DL4	101	3	2.97%	196	7	3.57%	1.21 [0.38-3.83]	0.786	0.996
KIR3DL1	101	1	0.99%	196	5	2.55%	2.62 [0.43-16.05]	0.383	0.750
KIR3DL3	101	1	0.99%	196	4	2.04%	2.08 [0.33-13.25]	0.514	0.805
KISS1	101	7	6.93%	196	28	14.29%	2.24 [1.08-4.63]	0.068	0.507
KITLG	101	0	0.00%	196	5	2.55%	2.2e+07 [0e+00-Inf]	0.992	1.000
KLC2	101	3	2.97%	196	5	2.55%	0.86 [0.25-2.89]	0.833	1.000
KLF10	101	15	14.85%	196	56	28.57%	2.29 [1.35-3.89]	9.81E-03	0.507
KLHDC8A	101	7	6.93%	196	27	13.78%	2.15 [1.03-4.45]	0.085	0.507
KLHDC9	101	6	5.94%	196	17	8.67%	1.50 [0.67-3.38]	0.407	0.774
KLHL12	101	7	6.93%	196	28	14.29%	2.24 [1.08-4.63]	0.068	0.507
KLHL20	101	5	4.95%	196	13	6.63%	1.36 [0.56-3.32]	0.566	0.852
KLHL24	101	3	2.97%	196	2	1.02%	0.34 [0.074-1.53]	0.237	0.599
KLHL25	101	1	0.99%	196	5	2.55%	2.62 [0.43-16.05]	0.383	0.750
KLHL35	101	4	3.96%	196	17	8.67%	2.30 [0.90-5.88]	0.143	0.535
KLHL38	101	14	13.86%	196	57	29.08%	2.55 [1.49-4.37]	4.36E-03	0.507
KLHL6	101	3	2.97%	196	2	1.02%	0.34 [0.074-1.53]	0.237	0.599
KLHL7	101	0	0.00%	196	6	3.06%	2.7e+07 [0e+00-Inf]	0.992	1.000
KMO	101	4	3.96%	196	20	10.20%	2.76 [1.09-6.95]	0.071	0.507
KPNA2	101	10	9.90%	196	27	13.78%	1.45 [0.76-2.77]	0.340	0.716
KPNB1	101	2	1.98%	196	8	4.08%	2.11 [0.56-7.86]	0.352	0.722
KPRP	101	7	6.93%	196	13	6.63%	0.95 [0.43-2.12]	0.923	1.000
KREMEN2	101	6	5.94%	196	4	2.04%	0.33 [0.11-0.97]	0.092	0.507
KRR1	101	2	1.98%	196	5	2.55%	1.30 [0.32-5.21]	0.759	0.996
KRT10	101	2	1.98%	196	5	2.55%	1.30 [0.32-5.21]	0.759	0.996
KRT12	101	1	0.99%	196	4	2.04%	2.08 [0.33-13.25]	0.514	0.805
KRT222	101	3	2.97%	196	5	2.55%	0.86 [0.25-2.89]	0.833	1.000
KRT23	101	1	0.99%	196	4	2.04%	2.08 [0.33-13.25]	0.514	0.805
KRT24	101	2	1.98%	196	5	2.55%	1.30 [0.32-5.21]	0.759	0.996
KRT25	101	2	1.98%	196	5	2.55%	1.30 [0.32-5.21]	0.759	0.996
KRT26	101	2	1.98%	196	5	2.55%	1.30 [0.32-5.21]	0.759	0.996
KRT27	101	2	1.98%	196	5	2.55%	1.30 [0.32-5.21]	0.759	0.996
KRT28	101	2	1.98%	196	5	2.55%	1.30 [0.32-5.21]	0.759	0.996
KRT34	101	2	1.98%	196	7	3.57%	1.83 [0.48-6.96]	0.455	0.805
KRTAP5-10	101	10	9.90%	196	18	9.18%	0.92 [0.47-1.82]	0.841	1.000
KRTAP5-11	101	10	9.90%	196	18	9.18%	0.92 [0.47-1.82]	0.841	1.000
KRTAP5-7	101	10	9.90%	196	20	10.20%	1.03 [0.53-2.02]	0.935	1.000
KRTAP5-8	101	10	9.90%	196	20	10.20%	1.03 [0.53-2.02]	0.935	1.000
KRTAP5-9	101	10	9.90%	196	18	9.18%	0.92 [0.47-1.82]	0.841	1.000
KRTCAP2	101	7	6.93%	196	13	6.63%	0.95 [0.43-2.12]	0.923	1.000
KSR1	101	4	3.96%	196	5	2.55%	0.63 [0.21-1.95]	0.505	0.805
L3MBTL1	101	2	1.98%	196	5	2.55%	1.30 [0.32-5.21]	0.759	0.996
LACTB2	101	16	15.84%	196	39	19.90%	1.32 [0.77-2.26]	0.395	0.770
LAD1	101	6	5.94%	196	24	12.24%	2.21 [1.01-4.82]	0.095	0.507
LAMA5	101	6	5.94%	196	10	5.10%	0.85 [0.36-2.04]	0.762	0.996
LAMB3	101	6	5.94%	196	23	11.73%	2.11 [0.96-4.60]	0.118	0.526
LAMC1	101	5	4.95%	196	18	9.18%	1.94 [0.82-4.58]	0.203	0.570
LAMC2	101	5	4.95%	196	19	9.69%	2.06 [0.88-4.83]	0.163	0.535
LAMP3	101	3	2.97%	196	2	1.02%	0.34 [0.074-1.53]	0.237	0.599
LAPTM4B	101	16	15.84%	196	53	27.04%	1.97 [1.17-3.31]	3.23E-02	0.507
LASP1	101	4	3.96%	196	11	5.61%	1.44 [0.54-3.85]	0.540	0.837
CERS2	101	7	6.93%	196	15	7.65%	1.11 [0.51-2.43]	0.822	1.000
LAT	101	6	5.94%	196	3	1.53%	0.25 [0.076-0.80]	0.051	0.507
LAX1	101	7	6.93%	196	28	14.29%	2.24 [1.08-4.63]	0.068	0.507
LBP	101	4	3.96%	196	4	2.04%	0.51 [0.16-1.65]	0.342	0.716
LBR	101	6	5.94%	196	17	8.67%	1.50 [0.67-3.38]	0.407	0.774
LCE1A	101	8	7.92%	196	12	6.12%	0.76 [0.35-1.65]	0.559	0.852
LCE1B	101	8	7.92%	196	12	6.12%	0.76 [0.35-1.65]	0.559	0.852
LCE1C	101	10	9.90%	196	14	7.14%	0.70 [0.34-1.43]	0.411	0.774
LCE1D	101	7	6.93%	196	14	7.14%	1.03 [0.47-2.28]	0.946	1.000
LCE1E	101	10	9.90%	196	12	6.12%	0.59 [0.28-1.24]	0.243	0.612
LCE1F	101	7	6.93%	196	13	6.63%	0.95 [0.43-2.12]	0.923	1.000
LCE2A	101	6	5.94%	196	13	6.63%	1.12 [0.49-2.60]	0.817	1.000
LCE2B	101	7	6.93%	196	13	6.63%	0.95 [0.43-2.12]	0.923	1.000
LCE2C	101	7	6.93%	196	13	6.63%	0.95 [0.43-2.12]	0.923	1.000
LCE2D	101	7	6.93%	196	13	6.63%	0.95 [0.43-2.12]	0.923	1.000
LCE3A	101	7	6.93%	196	13	6.63%	0.95 [0.43-2.12]	0.923	1.000
LCE3B	101	25	24.75%	196	46	23.47%	0.93 [0.58-1.49]	0.806	1.000

Symbol	CINSARC Low			CINSARC High			CINSARC High vs. Low		
	N tested	N altered	Fq alteration	N tested	N altered	Fq alteration	OR [95%CI]	p-value*	q-value
LCE3C	101	35	34.65%	196	65	33.16%	0.94 [0.61-1.43]	0.797	1.000
LCE3D	101	7	6.93%	196	13	6.63%	0.95 [0.43-2.12]	0.923	1.000
LCE3E	101	7	6.93%	196	13	6.63%	0.95 [0.43-2.12]	0.923	1.000
LCE4A	101	7	6.93%	196	13	6.63%	0.95 [0.43-2.12]	0.923	1.000
LCE5A	101	7	6.93%	196	13	6.63%	0.95 [0.43-2.12]	0.923	1.000
LCE6A	101	7	6.93%	196	11	5.61%	0.80 [0.35-1.82]	0.652	0.928
LCMT1	101	5	4.95%	196	3	1.53%	0.30 [0.088-1.01]	0.103	0.507
LDHAL6A	101	1	0.99%	196	4	2.04%	2.08 [0.33-13.25]	0.514	0.805
LDHA	101	1	0.99%	196	4	2.04%	2.08 [0.33-13.25]	0.514	0.805
LDHC	101	1	0.99%	196	4	2.04%	2.08 [0.33-13.25]	0.514	0.805
LEFTY1	101	6	5.94%	196	19	9.69%	1.70 [0.77-3.78]	0.274	0.643
LEFTY2	101	6	5.94%	196	19	9.69%	1.70 [0.77-3.78]	0.274	0.643
LELP1	101	7	6.93%	196	11	5.61%	0.80 [0.35-1.82]	0.652	0.928
LEMD1	101	7	6.93%	196	26	13.27%	2.05 [0.99-4.27]	0.106	0.507
LEMD3	101	2	1.98%	196	6	3.06%	1.56 [0.40-6.08]	0.589	0.869
LENEP	101	8	7.92%	196	14	7.14%	0.89 [0.42-1.91]	0.808	1.000
LETM2	101	12	11.88%	196	44	22.45%	2.15 [1.20-3.83]	2.99E-02	0.507
LGALS3BP	101	3	2.97%	196	13	6.63%	2.32 [0.79-6.79]	0.197	0.570
LGALS8	101	7	6.93%	196	19	9.69%	1.44 [0.68-3.07]	0.427	0.791
LGALS9C	101	6	5.94%	196	7	3.57%	0.59 [0.23-1.50]	0.349	0.722
LGALS9	101	4	3.96%	196	5	2.55%	0.63 [0.21-1.95]	0.505	0.805
LGR5	101	1	0.99%	196	13	6.63%	7.10 [1.27-39.64]	0.061	0.507
LGR6	101	8	7.92%	196	25	12.76%	1.70 [0.84-3.43]	0.213	0.588
LHX1	101	2	1.98%	196	5	2.55%	1.30 [0.32-5.21]	0.759	0.996
LHX4	101	6	5.94%	196	19	9.69%	1.70 [0.77-3.78]	0.274	0.643
LHX9	101	5	4.95%	196	23	11.73%	2.55 [1.10-5.90]	0.066	0.507
LIG3	101	1	0.99%	196	5	2.55%	2.62 [0.43-16.05]	0.383	0.750
LIMD2	101	8	7.92%	196	30	15.31%	2.10 [1.06-4.18]	0.076	0.507
LIME1	101	7	6.93%	196	15	7.65%	1.11 [0.51-2.43]	0.822	1.000
LIN28B	101	1	0.99%	196	6	3.06%	3.16 [0.53-18.88]	0.290	0.650
LIN7A	101	1	0.99%	196	4	2.04%	2.08 [0.33-13.25]	0.514	0.805
LIN9	101	6	5.94%	196	19	9.69%	1.70 [0.77-3.78]	0.274	0.643
LINGO4	101	6	5.94%	196	12	6.12%	1.03 [0.44-2.41]	0.950	1.000
LIPH	101	3	2.97%	196	2	1.02%	0.34 [0.074-1.53]	0.237	0.599
LIPT2	101	6	5.94%	196	12	6.12%	1.03 [0.44-2.41]	0.950	1.000
LITAF	101	5	4.95%	196	4	2.04%	0.40 [0.13-1.23]	0.179	0.535
LIX1L	101	5	4.95%	196	10	5.10%	1.03 [0.41-2.60]	0.955	1.000
LLGL2	101	5	4.95%	196	12	6.12%	1.25 [0.51-3.08]	0.681	0.943
LLPH	101	2	1.98%	196	8	4.08%	2.11 [0.56-7.86]	0.352	0.722
LMAN1	101	1	0.99%	196	4	2.04%	2.08 [0.33-13.25]	0.514	0.805
LMF1	101	6	5.94%	196	4	2.04%	0.33 [0.11-0.97]	0.092	0.507
LMLN	101	2	1.98%	196	4	2.04%	1.03 [0.24-4.35]	0.972	1.000
LMNA	101	6	5.94%	196	9	4.59%	0.76 [0.31-1.86]	0.616	0.898
LMOD1	101	7	6.93%	196	24	12.24%	1.87 [0.90-3.92]	0.161	0.535
LMX1A	101	4	3.96%	196	13	6.63%	1.72 [0.66-4.51]	0.353	0.722
LOC10012678	101	1	0.99%	196	4	2.04%	2.08 [0.33-13.25]	0.514	0.805
LOC10013098	101	10	9.90%	196	17	8.67%	0.86 [0.43-1.72]	0.728	0.986
LOC10013149	101	4	3.96%	196	8	4.08%	1.03 [0.37-2.88]	0.960	1.000
LOC10013436	101	7	6.93%	196	4	2.04%	0.28 [0.098-0.80]	4.63E-02	0.507
LOC10019098	101	6	5.94%	196	2	1.02%	0.16 [0.042-0.64]	2.82E-02	0.507
LOC148696	101	7	6.93%	196	25	12.76%	1.96 [0.94-4.09]	0.131	0.535
LOC154761	101	4	3.96%	196	5	2.55%	0.63 [0.21-1.95]	0.505	0.805
LOC220729	101	3	2.97%	196	4	2.04%	0.68 [0.19-2.43]	0.619	0.898
LOC388242	101	4	3.96%	196	3	1.53%	0.38 [0.11-1.35]	0.207	0.573
LOC391322	101	4	3.96%	196	8	4.08%	1.03 [0.37-2.88]	0.960	1.000
LOC401463	101	14	13.86%	196	35	17.86%	1.35 [0.77-2.38]	0.381	0.750
LOC441204	101	0	0.00%	196	6	3.06%	2.7e+07 [0e+00-Inf]	0.992	1.000
LOC606724	101	4	3.96%	196	3	1.53%	0.38 [0.11-1.35]	0.207	0.573
LOC646214	101	1	0.99%	196	4	2.04%	2.08 [0.33-13.25]	0.514	0.805
LOC646762	101	0	0.00%	196	6	3.06%	2.7e+07 [0e+00-Inf]	0.992	1.000
LOC652276	101	9	8.91%	196	5	2.55%	0.27 [0.10-0.69]	2.12E-02	0.507
LOC653653	101	7	6.93%	196	33	16.84%	2.72 [1.33-5.57]	2.17E-02	0.507
LOC728024	101	11	10.89%	196	53	27.04%	3.03 [1.68-5.46]	1.92E-03	0.507
LOC728989	101	6	5.94%	196	12	6.12%	1.03 [0.44-2.41]	0.950	1.000
LOR	101	7	6.93%	196	12	6.12%	0.88 [0.39-1.97]	0.788	0.996
LPGAT1	101	6	5.94%	196	20	10.20%	1.80 [0.81-3.98]	0.224	0.596
LPIN3	101	2	1.98%	196	4	2.04%	1.03 [0.24-4.35]	0.972	1.000
LPO	101	6	5.94%	196	26	13.27%	2.42 [1.12-5.25]	0.060	0.507
LPP	101	2	1.98%	196	3	1.53%	0.77 [0.17-3.50]	0.776	0.996
LRCH3	101	3	2.97%	196	4	2.04%	0.68 [0.19-2.43]	0.619	0.898
LRFN4	101	8	7.92%	196	14	7.14%	0.89 [0.42-1.91]	0.808	1.000

Symbol	CINSARC Low			CINSARC High			CINSARC High vs. Low		
	N tested	N altered	Fq alteration	N tested	N altered	Fq alteration	OR [95%CI]	p-value*	q-value
LRP12	101	17	16.83%	196	52	26.53%	1.78 [1.07-2.98]	0.063	0.507
LRP5	101	6	5.94%	196	19	9.69%	1.70 [0.77-3.78]	0.274	0.643
LRRC10B	101	1	0.99%	196	5	2.55%	2.62 [0.43-16.05]	0.383	0.750
LRRC10	101	2	1.98%	196	16	8.16%	4.40 [1.26-15.37]	0.051	0.507
LRRC14	101	18	17.82%	196	49	25.00%	1.54 [0.93-2.55]	0.163	0.535
LRRC15	101	3	2.97%	196	3	1.53%	0.51 [0.13-1.98]	0.412	0.774
LRRC24	101	18	17.82%	196	49	25.00%	1.54 [0.93-2.55]	0.163	0.535
LRRC28	101	1	0.99%	196	7	3.57%	3.70 [0.63-21.75]	0.224	0.596
LRRC32	101	6	5.94%	196	17	8.67%	1.50 [0.67-3.38]	0.407	0.774
NRROS	101	3	2.97%	196	4	2.04%	0.68 [0.19-2.43]	0.619	0.898
LRRC37A2	101	11	10.89%	196	16	8.16%	0.73 [0.37-1.43]	0.440	0.800
LRRC37A3	101	7	6.93%	196	21	10.71%	1.61 [0.76-3.40]	0.294	0.650
LRRC37A	101	8	7.92%	196	9	4.59%	0.56 [0.24-1.28]	0.248	0.623
LRRC45	101	2	1.98%	196	13	6.63%	3.52 [0.99-12.47]	0.102	0.507
LRRC46	101	1	0.99%	196	10	5.10%	5.38 [0.95-30.54]	0.111	0.511
LRRC52	101	4	3.96%	196	12	6.12%	1.58 [0.60-4.18]	0.438	0.798
LRRC59	101	4	3.96%	196	19	9.69%	2.60 [1.03-6.59]	0.090	0.507
PPP1R42	101	15	14.85%	196	40	20.41%	1.47 [0.85-2.53]	0.245	0.616
LRRC69	101	18	17.82%	196	46	23.47%	1.41 [0.85-2.35]	0.263	0.643
LRRC6	101	15	14.85%	196	56	28.57%	2.29 [1.35-3.89]	9.81E-03	0.507
LRRCC1	101	17	16.83%	196	47	23.98%	1.56 [0.93-2.61]	0.158	0.535
LRRN2	101	7	6.93%	196	26	13.27%	2.05 [0.99-4.27]	0.106	0.507
LRTOMT	101	5	4.95%	196	6	3.06%	0.61 [0.22-1.68]	0.418	0.779
LSG1	101	3	2.97%	196	3	1.53%	0.51 [0.13-1.98]	0.412	0.774
LSM14B	101	6	5.94%	196	12	6.12%	1.03 [0.44-2.41]	0.950	1.000
LSM1	101	11	10.89%	196	47	23.98%	2.58 [1.43-4.67]	8.55E-03	0.507
LSM5	101	0	0.00%	196	7	3.57%	3.2e+07 [0e+00-Inf]	0.992	1.000
LUC7L3	101	4	3.96%	196	20	10.20%	2.76 [1.09-6.95]	0.071	0.507
LUC7L	101	7	6.93%	196	4	2.04%	0.28 [0.098-0.80]	4.63E-02	0.507
LUM	101	0	0.00%	196	5	2.55%	2.2e+07 [0e+00-Inf]	0.992	1.000
LY6D	101	17	16.83%	196	49	25.00%	1.65 [0.98-2.76]	0.111	0.511
LY6E	101	16	15.84%	196	49	25.00%	1.77 [1.05-2.99]	0.073	0.507
LY6H	101	15	14.85%	196	50	25.51%	1.96 [1.15-3.35]	3.75E-02	0.507
LY6K	101	16	15.84%	196	49	25.00%	1.77 [1.05-2.99]	0.073	0.507
LY96	101	17	16.83%	196	41	20.92%	1.31 [0.77-2.21]	0.401	0.774
LY9	101	6	5.94%	196	16	8.16%	1.41 [0.62-3.18]	0.490	0.805
LYNX1	101	17	16.83%	196	49	25.00%	1.65 [0.98-2.76]	0.111	0.511
LYN	101	14	13.86%	196	31	15.82%	1.17 [0.66-2.07]	0.656	0.931
LYPD2	101	16	15.84%	196	49	25.00%	1.77 [1.05-2.99]	0.073	0.507
LYPLA1	101	12	11.88%	196	30	15.31%	1.34 [0.73-2.45]	0.423	0.787
LYPLAL1	101	6	5.94%	196	20	10.20%	1.80 [0.81-3.98]	0.224	0.596
LYRM1	101	5	4.95%	196	2	1.02%	0.20 [0.049-0.80]	0.056	0.507
LYSMD1	101	7	6.93%	196	13	6.63%	0.95 [0.43-2.12]	0.923	1.000
LYSMD4	101	1	0.99%	196	6	3.06%	3.16 [0.53-18.88]	0.290	0.650
LYST	101	7	6.93%	196	20	10.20%	1.53 [0.72-3.24]	0.355	0.722
LYZL6	101	2	1.98%	196	4	2.04%	1.03 [0.24-4.35]	0.972	1.000
LYZ	101	3	2.97%	196	18	9.18%	3.30 [1.16-9.41]	0.060	0.507
MAEL	101	5	4.95%	196	13	6.63%	1.36 [0.56-3.32]	0.566	0.852
MAF1	101	17	16.83%	196	51	26.02%	1.74 [1.04-2.90]	0.076	0.507
MAFA	101	17	16.83%	196	51	26.02%	1.74 [1.04-2.90]	0.076	0.507
MAFB	101	3	2.97%	196	6	3.06%	1.03 [0.32-3.36]	0.965	1.000
MAFG	101	2	1.98%	196	13	6.63%	3.52 [0.99-12.47]	0.102	0.507
MAGEF1	101	3	2.97%	196	3	1.53%	0.51 [0.13-1.98]	0.412	0.774
MAK16	101	4	3.96%	196	10	5.10%	1.30 [0.48-3.53]	0.661	0.935
MAL2	101	15	14.85%	196	59	30.10%	2.47 [1.46-4.18]	4.76E-03	0.507
MALT1	101	1	0.99%	196	4	2.04%	2.08 [0.33-13.25]	0.514	0.805
MAN2A2	101	1	0.99%	196	4	2.04%	2.08 [0.33-13.25]	0.514	0.805
MANBAL	101	5	4.95%	196	3	1.53%	0.30 [0.088-1.01]	0.103	0.507
MAP1LC3A	101	3	2.97%	196	4	2.04%	0.68 [0.19-2.43]	0.619	0.898
MAP1LC3C	101	5	4.95%	196	19	9.69%	2.06 [0.88-4.83]	0.163	0.535
MAP2K4	101	0	0.00%	196	1	0.51%	3.2e+07 [0e+00-Inf]	0.997	1.000
MAP2K6	101	5	4.95%	196	22	11.22%	2.43 [1.05-5.63]	0.083	0.507
MAP3K13	101	3	2.97%	196	3	1.53%	0.51 [0.13-1.98]	0.412	0.774
MAP3K3	101	8	7.92%	196	30	15.31%	2.10 [1.06-4.18]	0.076	0.507
MAP6D1	101	3	2.97%	196	2	1.02%	0.34 [0.074-1.53]	0.237	0.599
MAP6	101	5	4.95%	196	20	10.20%	2.18 [0.93-5.10]	0.130	0.535
MAPK15	101	17	16.83%	196	50	25.51%	1.69 [1.01-2.83]	0.092	0.507
MAPK3	101	5	4.95%	196	4	2.04%	0.40 [0.13-1.23]	0.179	0.535
MAPK7	101	4	3.96%	196	2	1.02%	0.25 [0.059-1.05]	0.113	0.511
MAPK8IP3	101	6	5.94%	196	5	2.55%	0.41 [0.15-1.15]	0.154	0.535
MAPKAPK2	101	6	5.94%	196	25	12.76%	2.31 [1.06-5.03]	0.076	0.507

Symbol	CINSARC Low			CINSARC High			CINSARC High vs. Low		
	N tested	N altered	Fq alteration	N tested	N altered	Fq alteration	OR [95%CI]	p-value*	q-value
MARCH10	101	8	7.92%	196	31	15.82%	2.18 [1.10-4.34]	0.061	0.507
MARCH9	101	0	0.00%	196	5	2.55%	2.2e+07 [0e+00-Inf]	0.992	1.000
MARK1	101	6	5.94%	196	18	9.18%	1.60 [0.72-3.57]	0.335	0.716
MATN2	101	16	15.84%	196	54	27.55%	2.02 [1.20-3.40]	2.60E-02	0.507
MATN4	101	5	4.95%	196	3	1.53%	0.30 [0.088-1.01]	0.103	0.507
MAZ	101	5	4.95%	196	3	1.53%	0.30 [0.088-1.01]	0.103	0.507
MBIP	101	0	0.00%	196	9	4.59%	4.1e+07 [0e+00-Inf]	0.992	1.000
MBTD1	101	4	3.96%	196	18	9.18%	2.45 [0.97-6.23]	0.114	0.512
MC3R	101	8	7.92%	196	12	6.12%	0.76 [0.35-1.65]	0.559	0.852
MCCC1	101	3	2.97%	196	2	1.02%	0.34 [0.074-1.53]	0.237	0.599
MCF2L2	101	3	2.97%	196	2	1.02%	0.34 [0.074-1.53]	0.237	0.599
MCL1	101	9	8.91%	196	16	8.16%	0.91 [0.44-1.86]	0.826	1.000
MCM4	101	14	13.86%	196	27	13.78%	0.99 [0.55-1.78]	0.984	1.000
MCTP2	101	0	0.00%	196	5	2.55%	2.2e+07 [0e+00-Inf]	0.992	1.000
MDM1	101	2	1.98%	196	15	7.65%	4.10 [1.17-14.39]	0.064	0.507
MDM2	101	2	1.98%	196	17	8.67%	4.70 [1.35-16.35]	4.11E-02	0.507
MDM4	101	7	6.93%	196	27	13.78%	2.15 [1.03-4.45]	0.085	0.507
MED13	101	9	8.91%	196	33	16.84%	2.07 [1.08-3.98]	0.068	0.507
MED1	101	5	4.95%	196	12	6.12%	1.25 [0.51-3.08]	0.681	0.943
MED24	101	5	4.95%	196	11	5.61%	1.14 [0.46-2.84]	0.811	1.000
MED30	101	15	14.85%	196	56	28.57%	2.29 [1.35-3.89]	9.81E-03	0.507
MEF2A	101	1	0.99%	196	6	3.06%	3.16 [0.53-18.88]	0.290	0.650
MEF2D	101	6	5.94%	196	9	4.59%	0.76 [0.31-1.86]	0.616	0.898
MEFV	101	5	4.95%	196	4	2.04%	0.40 [0.13-1.23]	0.179	0.535
MESP1	101	1	0.99%	196	8	4.08%	4.26 [0.73-24.65]	0.175	0.535
MESP2	101	1	0.99%	196	8	4.08%	4.26 [0.73-24.65]	0.175	0.535
METRNL	101	3	2.97%	196	15	7.65%	2.71 [0.94-7.82]	0.122	0.534
METRN	101	6	5.94%	196	4	2.04%	0.33 [0.11-0.97]	0.092	0.507
METTL11B	101	5	4.95%	196	13	6.63%	1.36 [0.56-3.32]	0.566	0.852
METTL1	101	0	0.00%	196	5	2.55%	2.2e+07 [0e+00-Inf]	0.992	1.000
METTL2A	101	9	8.91%	196	34	17.35%	2.15 [1.12-4.12]	0.055	0.507
METTL9	101	6	5.94%	196	2	1.02%	0.16 [0.042-0.64]	2.82E-02	0.507
MEX3A	101	6	5.94%	196	9	4.59%	0.76 [0.31-1.86]	0.616	0.898
MFAP4	101	4	3.96%	196	2	1.02%	0.25 [0.059-1.05]	0.113	0.511
MFGE8	101	1	0.99%	196	5	2.55%	2.62 [0.43-16.05]	0.383	0.750
MFN1	101	4	3.96%	196	2	1.02%	0.25 [0.059-1.05]	0.113	0.511
MFSD11	101	3	2.97%	196	13	6.63%	2.32 [0.79-6.79]	0.197	0.570
MFSD3	101	17	16.83%	196	49	25.00%	1.65 [0.98-2.76]	0.111	0.511
MGAT5B	101	3	2.97%	196	13	6.63%	2.32 [0.79-6.79]	0.197	0.570
MGC16275	101	6	5.94%	196	15	7.65%	1.31 [0.58-2.98]	0.586	0.869
MGC2889	101	3	2.97%	196	3	1.53%	0.51 [0.13-1.98]	0.412	0.774
MGRN1	101	5	4.95%	196	6	3.06%	0.61 [0.22-1.68]	0.418	0.779
MGST3	101	4	3.96%	196	12	6.12%	1.58 [0.60-4.18]	0.438	0.798
MIA3	101	6	5.94%	196	19	9.69%	1.70 [0.77-3.78]	0.274	0.643
MIF4GD	101	5	4.95%	196	12	6.12%	1.25 [0.51-3.08]	0.681	0.943
MIPOL1	101	0	0.00%	196	11	5.61%	5.1e+07 [0e+00-Inf]	0.992	1.000
MIXL1	101	6	5.94%	196	19	9.69%	1.70 [0.77-3.78]	0.274	0.643
MKRN1	101	1	0.99%	196	4	2.04%	2.08 [0.33-13.25]	0.514	0.805
MKS1	101	6	5.94%	196	26	13.27%	2.42 [1.12-5.25]	0.060	0.507
MLLT11	101	7	6.93%	196	14	7.14%	1.03 [0.47-2.28]	0.946	1.000
MLLT6	101	5	4.95%	196	9	4.59%	0.92 [0.36-2.37]	0.890	1.000
MLST8	101	6	5.94%	196	5	2.55%	0.41 [0.15-1.15]	0.154	0.535
MMD	101	8	7.92%	196	20	10.20%	1.32 [0.64-2.71]	0.525	0.818
MMP16	101	17	16.83%	196	50	25.51%	1.69 [1.01-2.83]	0.092	0.507
MMP24	101	4	3.96%	196	6	3.06%	0.77 [0.26-2.26]	0.685	0.943
MMP25	101	6	5.94%	196	4	2.04%	0.33 [0.11-0.97]	0.092	0.507
MMP28	101	2	1.98%	196	4	2.04%	1.03 [0.24-4.35]	0.972	1.000
MMP9	101	5	4.95%	196	4	2.04%	0.40 [0.13-1.23]	0.179	0.535
MNDA	101	6	5.94%	196	8	4.08%	0.67 [0.27-1.68]	0.476	0.805
MOCS3	101	6	5.94%	196	14	7.14%	1.22 [0.53-2.79]	0.696	0.951
MOGAT2	101	6	5.94%	196	20	10.20%	1.80 [0.81-3.98]	0.224	0.596
MON2	101	1	0.99%	196	6	3.06%	3.16 [0.53-18.88]	0.290	0.650
MARC1	101	6	5.94%	196	18	9.18%	1.60 [0.72-3.57]	0.335	0.716
MARC2	101	6	5.94%	196	18	9.18%	1.60 [0.72-3.57]	0.335	0.716
MOS	101	14	13.86%	196	31	15.82%	1.17 [0.66-2.07]	0.656	0.931
MPG	101	7	6.93%	196	4	2.04%	0.28 [0.098-0.80]	4.63E-02	0.507
MPO	101	6	5.94%	196	26	13.27%	2.42 [1.12-5.25]	0.060	0.507
MPP6	101	0	0.00%	196	6	3.06%	2.7e+07 [0e+00-Inf]	0.992	1.000
MPV17L	101	7	6.93%	196	3	1.53%	0.21 [0.066-0.66]	2.55E-02	0.507
MPZL1	101	5	4.95%	196	12	6.12%	1.25 [0.51-3.08]	0.681	0.943
MPZ	101	6	5.94%	196	16	8.16%	1.41 [0.62-3.18]	0.490	0.805

Symbol	CINSARC Low			CINSARC High			CINSARC High vs. Low		
	N tested	N altered	Fq alteration	N tested	N altered	Fq alteration	OR [95%CI]	p-value*	q-value
MR1	101	5	4.95%	196	19	9.69%	2.06 [0.88-4.83]	0.163	0.535
MRC2	101	8	7.92%	196	34	17.35%	2.44 [1.23-4.82]	3.12E-02	0.507
MRGPRD	101	16	15.84%	196	39	19.90%	1.32 [0.77-2.26]	0.395	0.770
MRGPRF	101	16	15.84%	196	43	21.94%	1.49 [0.88-2.54]	0.214	0.591
MRGPRX1	101	10	9.90%	196	7	3.57%	0.34 [0.15-0.78]	3.26E-02	0.507
MRM1	101	3	2.97%	196	5	2.55%	0.86 [0.25-2.89]	0.833	1.000
MRPL10	101	1	0.99%	196	10	5.10%	5.38 [0.95-30.54]	0.111	0.511
MRPL11	101	5	4.95%	196	10	5.10%	1.03 [0.41-2.60]	0.955	1.000
MRPL12	101	2	1.98%	196	12	6.12%	3.23 [0.90-11.53]	0.130	0.535
MRPL13	101	15	14.85%	196	53	27.04%	2.12 [1.25-3.61]	1.95E-02	0.507
MRPL15	101	12	11.88%	196	30	15.31%	1.34 [0.73-2.45]	0.423	0.787
MRPL21	101	15	14.85%	196	38	19.39%	1.38 [0.80-2.38]	0.335	0.716
MRPL24	101	7	6.93%	196	8	4.08%	0.57 [0.24-1.37]	0.294	0.650
MRPL27	101	4	3.96%	196	18	9.18%	2.45 [0.97-6.23]	0.114	0.512
MRPL28	101	7	6.93%	196	4	2.04%	0.28 [0.098-0.80]	4.63E-02	0.507
MRPL38	101	4	3.96%	196	12	6.12%	1.58 [0.60-4.18]	0.438	0.798
MRPL45	101	4	3.96%	196	8	4.08%	1.03 [0.37-2.88]	0.960	1.000
MRPL46	101	0	0.00%	196	6	3.06%	2.7e+07 [0e+00-Inf]	0.992	1.000
MRPL47	101	4	3.96%	196	2	1.02%	0.25 [0.059-1.05]	0.113	0.511
MRPL48	101	6	5.94%	196	11	5.61%	0.94 [0.40-2.23]	0.908	1.000
MRPL55	101	6	5.94%	196	21	10.71%	1.90 [0.86-4.19]	0.181	0.535
MRPL9	101	6	5.94%	196	12	6.12%	1.03 [0.44-2.41]	0.950	1.000
MRPS11	101	0	0.00%	196	6	3.06%	2.7e+07 [0e+00-Inf]	0.992	1.000
MRPS14	101	5	4.95%	196	16	8.16%	1.71 [0.72-4.07]	0.311	0.680
MRPS16	101	0	0.00%	196	5	2.55%	2.2e+07 [0e+00-Inf]	0.992	1.000
MRPS21	101	8	7.92%	196	15	7.65%	0.96 [0.46-2.04]	0.935	1.000
MRPS23	101	6	5.94%	196	26	13.27%	2.42 [1.12-5.25]	0.060	0.507
MRPS28	101	17	16.83%	196	48	24.49%	1.60 [0.96-2.68]	0.133	0.535
MRPS34	101	6	5.94%	196	5	2.55%	0.41 [0.15-1.15]	0.154	0.535
MRPS7	101	5	4.95%	196	12	6.12%	1.25 [0.51-3.08]	0.681	0.943
MRS2P2	101	2	1.98%	196	12	6.12%	3.23 [0.90-11.53]	0.130	0.535
MSC	101	15	14.85%	196	42	21.43%	1.56 [0.91-2.69]	0.175	0.535
MSI2	101	7	6.93%	196	28	14.29%	2.24 [1.08-4.63]	0.068	0.507
MSL1	101	5	4.95%	196	9	4.59%	0.92 [0.36-2.37]	0.890	1.000
MSLN	101	6	5.94%	196	4	2.04%	0.33 [0.11-0.97]	0.092	0.507
MSRB3	101	2	1.98%	196	7	3.57%	1.83 [0.48-6.96]	0.455	0.805
MSTO1	101	6	5.94%	196	10	5.10%	0.85 [0.36-2.04]	0.762	0.996
MSTO2P	101	6	5.94%	196	10	5.10%	0.85 [0.36-2.04]	0.762	0.996
MSX2P1	101	6	5.94%	196	24	12.24%	2.21 [1.01-4.82]	0.095	0.507
MTBP	101	15	14.85%	196	53	27.04%	2.12 [1.25-3.61]	1.95E-02	0.507
MTDH	101	16	15.84%	196	53	27.04%	1.97 [1.17-3.31]	3.23E-02	0.507
MTFR1	101	15	14.85%	196	37	18.88%	1.33 [0.77-2.31]	0.388	0.757
MTHFD2L	101	2	1.98%	196	3	1.53%	0.77 [0.17-3.50]	0.776	0.996
MTMR11	101	7	6.93%	196	14	7.14%	1.03 [0.47-2.28]	0.946	1.000
MTMR4	101	7	6.93%	196	28	14.29%	2.24 [1.08-4.63]	0.068	0.507
MTR	101	6	5.94%	196	19	9.69%	1.70 [0.77-3.78]	0.274	0.643
MTSS1	101	16	15.84%	196	59	30.10%	2.29 [1.37-3.83]	8.37E-03	0.507
MTVR2	101	7	6.93%	196	23	11.73%	1.79 [0.85-3.74]	0.198	0.570
MTX1	101	8	7.92%	196	14	7.14%	0.89 [0.42-1.91]	0.808	1.000
MUC1	101	7	6.93%	196	13	6.63%	0.95 [0.43-2.12]	0.923	1.000
MUC20	101	4	3.96%	196	6	3.06%	0.77 [0.26-2.26]	0.685	0.943
MUC4	101	3	2.97%	196	4	2.04%	0.68 [0.19-2.43]	0.619	0.898
MVP	101	5	4.95%	196	3	1.53%	0.30 [0.088-1.01]	0.103	0.507
MXRA7	101	3	2.97%	196	14	7.14%	2.51 [0.86-7.30]	0.155	0.535
MYADML2	101	2	1.98%	196	13	6.63%	3.52 [0.99-12.47]	0.102	0.507
MYBL1	101	15	14.85%	196	38	19.39%	1.38 [0.80-2.38]	0.335	0.716
MYBL2	101	2	1.98%	196	4	2.04%	1.03 [0.24-4.35]	0.972	1.000
MYBPH	101	7	6.93%	196	28	14.29%	2.24 [1.08-4.63]	0.068	0.507
MYCBPAP	101	4	3.96%	196	19	9.69%	2.60 [1.03-6.59]	0.090	0.507
MYC	101	14	13.86%	196	64	32.65%	3.01 [1.76-5.15]	7.10E-04	0.507
MYEOV	101	21	20.79%	196	54	27.55%	1.45 [0.90-2.34]	0.205	0.573
MYF5	101	1	0.99%	196	4	2.04%	2.08 [0.33-13.25]	0.514	0.805
MYF6	101	1	0.99%	196	4	2.04%	2.08 [0.33-13.25]	0.514	0.805
MYH11	101	7	6.93%	196	3	1.53%	0.21 [0.066-0.66]	2.55E-02	0.507
MYH7B	101	4	3.96%	196	8	4.08%	1.03 [0.37-2.88]	0.960	1.000
MYL4	101	1	0.99%	196	7	3.57%	3.70 [0.63-21.75]	0.224	0.596
MYL9	101	4	3.96%	196	6	3.06%	0.77 [0.26-2.26]	0.685	0.943
MYLPF	101	4	3.96%	196	5	2.55%	0.63 [0.21-1.95]	0.505	0.805
MYO15B	101	5	4.95%	196	12	6.12%	1.25 [0.51-3.08]	0.681	0.943
MYO16	101	0	0.00%	196	5	2.55%	2.2e+07 [0e+00-Inf]	0.992	1.000
MYO18A	101	3	2.97%	196	5	2.55%	0.86 [0.25-2.89]	0.833	1.000

Symbol	CINSARC Low			CINSARC High			CINSARC High vs. Low		
	N tested	N altered	Fq alteration	N tested	N altered	Fq alteration	OR [95%CI]	p-value*	q-value
MYO19	101	2	1.98%	196	5	2.55%	1.30 [0.32-5.21]	0.759	0.996
MYO1D	101	2	1.98%	196	5	2.55%	1.30 [0.32-5.21]	0.759	0.996
MYO7A	101	8	7.92%	196	20	10.20%	1.32 [0.64-2.71]	0.525	0.818
MYOC	101	5	4.95%	196	13	6.63%	1.36 [0.56-3.32]	0.566	0.852
MYOD1	101	1	0.99%	196	4	2.04%	2.08 [0.33-13.25]	0.514	0.805
MYOG	101	7	6.93%	196	27	13.78%	2.15 [1.03-4.45]	0.085	0.507
MYOZ1	101	0	0.00%	196	5	2.55%	2.2e+07 [0e+00-Inf]	0.992	1.000
KAT8	101	4	3.96%	196	5	2.55%	0.63 [0.21-1.95]	0.505	0.805
KAT7	101	4	3.96%	196	16	8.16%	2.16 [0.84-5.53]	0.180	0.535
KAT6A	101	16	15.84%	196	31	15.82%	1.00 [0.57-1.73]	0.995	1.000
KAT6B	101	2	1.98%	196	7	3.57%	1.83 [0.48-6.96]	0.455	0.805
MYT1	101	9	8.91%	196	11	5.61%	0.61 [0.28-1.31]	0.287	0.650
NAAA	101	2	1.98%	196	5	2.55%	1.30 [0.32-5.21]	0.759	0.996
NAALAD2	101	1	0.99%	196	4	2.04%	2.08 [0.33-13.25]	0.514	0.805
NACA2	101	8	7.92%	196	30	15.31%	2.10 [1.06-4.18]	0.076	0.507
NADSYN1	101	10	9.90%	196	24	12.24%	1.27 [0.66-2.44]	0.549	0.851
NAGPA	101	5	4.95%	196	5	2.55%	0.50 [0.17-1.45]	0.286	0.650
NAIP	101	4	3.96%	196	8	4.08%	1.03 [0.37-2.88]	0.960	1.000
NAP1L1	101	1	0.99%	196	4	2.04%	2.08 [0.33-13.25]	0.514	0.805
NARF	101	2	1.98%	196	15	7.65%	4.10 [1.17-14.39]	0.064	0.507
NARS2	101	7	6.93%	196	15	7.65%	1.11 [0.51-2.43]	0.822	1.000
NAT14	101	2	1.98%	196	4	2.04%	1.03 [0.24-4.35]	0.972	1.000
NAA60	101	5	4.95%	196	4	2.04%	0.40 [0.13-1.23]	0.179	0.535
NAT9	101	5	4.95%	196	14	7.14%	1.48 [0.61-3.57]	0.467	0.805
NAV1	101	7	6.93%	196	23	11.73%	1.79 [0.85-3.74]	0.198	0.570
NAV3	101	1	0.99%	196	4	2.04%	2.08 [0.33-13.25]	0.514	0.805
NBN	101	18	17.82%	196	50	25.51%	1.58 [0.95-2.62]	0.137	0.535
NBPF10	101	5	4.95%	196	9	4.59%	0.92 [0.36-2.37]	0.890	1.000
NBPF14	101	4	3.96%	196	8	4.08%	1.03 [0.37-2.88]	0.960	1.000
NBPF15	101	3	2.97%	196	5	2.55%	0.86 [0.25-2.89]	0.833	1.000
NBPF7	101	1	0.99%	196	4	2.04%	2.08 [0.33-13.25]	0.514	0.805
NBPF9	101	1	0.99%	196	7	3.57%	3.70 [0.63-21.75]	0.224	0.596
NCALD	101	17	16.83%	196	55	28.06%	1.93 [1.16-3.21]	3.42E-02	0.507
NCBP2	101	4	3.96%	196	4	2.04%	0.51 [0.16-1.65]	0.342	0.716
NCF2	101	5	4.95%	196	20	10.20%	2.18 [0.93-5.10]	0.130	0.535
NCOA2	101	16	15.84%	196	40	20.41%	1.36 [0.80-2.33]	0.342	0.716
NCOA3	101	5	4.95%	196	7	3.57%	0.71 [0.27-1.90]	0.569	0.855
NCOA5	101	5	4.95%	196	4	2.04%	0.40 [0.13-1.23]	0.179	0.535
NCOA6	101	4	3.96%	196	6	3.06%	0.77 [0.26-2.26]	0.685	0.943
LINC00029	101	7	6.93%	196	13	6.63%	0.95 [0.43-2.12]	0.923	1.000
LINC00051	101	17	16.83%	196	49	25.00%	1.65 [0.98-2.76]	0.111	0.511
LINC00052	101	1	0.99%	196	7	3.57%	3.70 [0.63-21.75]	0.224	0.596
FBXL19-AS1	101	4	3.96%	196	5	2.55%	0.63 [0.21-1.95]	0.505	0.805
LINC00685	101	2	1.98%	196	4	2.04%	1.03 [0.24-4.35]	0.972	1.000
CRYM-AS1	101	5	4.95%	196	3	1.53%	0.30 [0.088-1.01]	0.103	0.507
TTTY14	101	0	0.00%	196	0	0.00%	1.00 [0e+00-Inf]	1.000	1.000
FAM224B	101	0	0.00%	196	0	0.00%	1.00 [0e+00-Inf]	1.000	1.000
LINC00235	101	7	6.93%	196	4	2.04%	0.28 [0.098-0.80]	4.63E-02	0.507
NCSTN	101	6	5.94%	196	13	6.63%	1.12 [0.49-2.60]	0.817	1.000
NDE1	101	7	6.93%	196	3	1.53%	0.21 [0.066-0.66]	2.55E-02	0.507
NDRG1	101	16	15.84%	196	55	28.06%	2.07 [1.23-3.48]	2.09E-02	0.507
NDRG3	101	4	3.96%	196	6	3.06%	0.77 [0.26-2.26]	0.685	0.943
NDST2	101	0	0.00%	196	5	2.55%	2.2e+07 [0e+00-Inf]	0.992	1.000
NDUFAB1	101	5	4.95%	196	3	1.53%	0.30 [0.088-1.01]	0.103	0.507
NDUFB10	101	6	5.94%	196	5	2.55%	0.41 [0.15-1.15]	0.154	0.535
NDUFB2	101	1	0.99%	196	4	2.04%	2.08 [0.33-13.25]	0.514	0.805
NDUFB5	101	4	3.96%	196	2	1.02%	0.25 [0.059-1.05]	0.113	0.511
NDUFB9	101	16	15.84%	196	57	29.08%	2.18 [1.30-3.66]	1.33E-02	0.507
NDUFC2	101	9	8.91%	196	20	10.20%	1.16 [0.58-2.32]	0.722	0.980
NDUFS2	101	6	5.94%	196	16	8.16%	1.41 [0.62-3.18]	0.490	0.805
NDUFS8	101	9	8.91%	196	22	11.22%	1.29 [0.65-2.56]	0.538	0.835
NDUFV1	101	9	8.91%	196	20	10.20%	1.16 [0.58-2.32]	0.722	0.980
NECAB1	101	18	17.82%	196	47	23.98%	1.45 [0.87-2.42]	0.226	0.598
NEK2	101	7	6.93%	196	20	10.20%	1.53 [0.72-3.24]	0.355	0.722
NEK7	101	5	4.95%	196	22	11.22%	2.43 [1.05-5.63]	0.083	0.507
NEK8	101	3	2.97%	196	9	4.59%	1.57 [0.52-4.80]	0.505	0.805
NENF	101	7	6.93%	196	21	10.71%	1.61 [0.76-3.40]	0.294	0.650
NES	101	7	6.93%	196	8	4.08%	0.57 [0.24-1.37]	0.294	0.650
NEU3	101	6	5.94%	196	15	7.65%	1.31 [0.58-2.98]	0.586	0.869
NEURL2	101	5	4.95%	196	4	2.04%	0.40 [0.13-1.23]	0.179	0.535
NEUROD2	101	6	5.94%	196	12	6.12%	1.03 [0.44-2.41]	0.950	1.000

Symbol	CINSARC Low			CINSARC High			CINSARC High vs. Low		
	N tested	N altered	Fq alteration	N tested	N altered	Fq alteration	OR [95%CI]	p-value*	q-value
NEUROD6	101	0	0.00%	196	8	4.08%	3.6e+07 [0e+00-Inf]	0.992	1.000
NF1	101	2	1.98%	196	3	1.53%	0.77 [0.17-3.50]	0.776	0.996
NFASC	101	7	6.93%	196	26	13.27%	2.05 [0.99-4.27]	0.106	0.507
NFATC2IP	101	6	5.94%	196	3	1.53%	0.25 [0.076-0.80]	0.051	0.507
NFATC2	101	7	6.93%	196	15	7.65%	1.11 [0.51-2.43]	0.822	1.000
NFE2L1	101	1	0.99%	196	13	6.63%	7.10 [1.27-39.64]	0.061	0.507
NFE2L3	101	0	0.00%	196	5	2.55%	2.2e+07 [0e+00-Inf]	0.992	1.000
NFKBIA	101	0	0.00%	196	9	4.59%	4.1e+07 [0e+00-Inf]	0.992	1.000
TONSL	101	19	18.81%	196	49	25.00%	1.44 [0.87-2.37]	0.231	0.599
NFS1	101	4	3.96%	196	8	4.08%	1.03 [0.37-2.88]	0.960	1.000
NGFR	101	4	3.96%	196	17	8.67%	2.30 [0.90-5.88]	0.143	0.535
NGRN	101	2	1.98%	196	7	3.57%	1.83 [0.48-6.96]	0.455	0.805
NHLH1	101	5	4.95%	196	13	6.63%	1.36 [0.56-3.32]	0.566	0.852
NHLRC4	101	7	6.93%	196	4	2.04%	0.28 [0.098-0.80]	4.63E-02	0.507
NID1	101	7	6.93%	196	20	10.20%	1.53 [0.72-3.24]	0.355	0.722
NIPAL2	101	16	15.84%	196	54	27.55%	2.02 [1.20-3.40]	2.60E-02	0.507
NIT1	101	6	5.94%	196	16	8.16%	1.41 [0.62-3.18]	0.490	0.805
NKAIN3	101	14	13.86%	196	32	16.33%	1.21 [0.69-2.15]	0.578	0.867
NKAIN4	101	7	6.93%	196	13	6.63%	0.95 [0.43-2.12]	0.923	1.000
NKX2-1	101	0	0.00%	196	11	5.61%	5.1e+07 [0e+00-Inf]	0.992	1.000
NKX2-5	101	2	1.98%	196	4	2.04%	1.03 [0.24-4.35]	0.972	1.000
NKX2-8	101	0	0.00%	196	12	6.12%	5.6e+07 [0e+00-Inf]	0.992	1.000
NKX6-3	101	15	14.85%	196	32	16.33%	1.12 [0.64-1.96]	0.742	0.996
NLE1	101	1	0.99%	196	7	3.57%	3.70 [0.63-21.75]	0.224	0.596
NLGN1	101	2	1.98%	196	3	1.53%	0.77 [0.17-3.50]	0.776	0.996
NLGN4Y	101	0	0.00%	196	0	0.00%	1.00 [0e+00-Inf]	1.000	1.000
NLK	101	4	3.96%	196	4	2.04%	0.51 [0.16-1.65]	0.342	0.716
NLRC3	101	5	4.95%	196	4	2.04%	0.40 [0.13-1.23]	0.179	0.535
NLRP11	101	1	0.99%	196	4	2.04%	2.08 [0.33-13.25]	0.514	0.805
NLRP13	101	1	0.99%	196	4	2.04%	2.08 [0.33-13.25]	0.514	0.805
NLRP3	101	5	4.95%	196	19	9.69%	2.06 [0.88-4.83]	0.163	0.535
NLRP4	101	1	0.99%	196	4	2.04%	2.08 [0.33-13.25]	0.514	0.805
NLRP8	101	1	0.99%	196	4	2.04%	2.08 [0.33-13.25]	0.514	0.805
NLRP9	101	2	1.98%	196	4	2.04%	1.03 [0.24-4.35]	0.972	1.000
NMB	101	1	0.99%	196	5	2.55%	2.62 [0.43-16.05]	0.383	0.750
NME1-NME2	101	4	3.96%	196	19	9.69%	2.60 [1.03-6.59]	0.090	0.507
NME1	101	4	3.96%	196	19	9.69%	2.60 [1.03-6.59]	0.090	0.507
NME2	101	4	3.96%	196	19	9.69%	2.60 [1.03-6.59]	0.090	0.507
NME3	101	6	5.94%	196	5	2.55%	0.41 [0.15-1.15]	0.154	0.535
NME4	101	7	6.93%	196	4	2.04%	0.28 [0.098-0.80]	4.63E-02	0.507
NME7	101	4	3.96%	196	17	8.67%	2.30 [0.90-5.88]	0.143	0.535
NMNAT2	101	5	4.95%	196	19	9.69%	2.06 [0.88-4.83]	0.163	0.535
NMRAL1	101	5	4.95%	196	6	3.06%	0.61 [0.22-1.68]	0.418	0.779
NNAT	101	5	4.95%	196	3	1.53%	0.30 [0.088-1.01]	0.103	0.507
NOD1	101	0	0.00%	196	7	3.57%	3.2e+07 [0e+00-Inf]	0.992	1.000
NOG	101	9	8.91%	196	22	11.22%	1.29 [0.65-2.56]	0.538	0.835
NOL11	101	9	8.91%	196	29	14.80%	1.78 [0.91-3.44]	0.155	0.535
NOM1	101	2	1.98%	196	3	1.53%	0.77 [0.17-3.50]	0.776	0.996
NOMO1	101	9	8.91%	196	3	1.53%	0.16 [0.052-0.49]	6.71E-03	0.507
NOMO2	101	9	8.91%	196	6	3.06%	0.32 [0.13-0.79]	3.70E-02	0.507
NOMO3	101	7	6.93%	196	3	1.53%	0.21 [0.066-0.66]	2.55E-02	0.507
NOS1AP	101	5	4.95%	196	18	9.18%	1.94 [0.82-4.58]	0.203	0.570
NOS2	101	4	3.96%	196	5	2.55%	0.63 [0.21-1.95]	0.505	0.805
NOTUM	101	2	1.98%	196	13	6.63%	3.52 [0.99-12.47]	0.102	0.507
NOVA1	101	0	0.00%	196	5	2.55%	2.2e+07 [0e+00-Inf]	0.992	1.000
NOXO1	101	6	5.94%	196	5	2.55%	0.41 [0.15-1.15]	0.154	0.535
NPAS3	101	1	0.99%	196	7	3.57%	3.70 [0.63-21.75]	0.224	0.596
NPAS4	101	5	4.95%	196	8	4.08%	0.82 [0.31-2.13]	0.729	0.986
NPBWR1	101	12	11.88%	196	27	13.78%	1.18 [0.64-2.18]	0.647	0.928
NPBWR2	101	7	6.93%	196	11	5.61%	0.80 [0.35-1.82]	0.652	0.928
NPB	101	2	1.98%	196	13	6.63%	3.52 [0.99-12.47]	0.102	0.507
NPEPL1	101	8	7.92%	196	16	8.16%	1.03 [0.49-2.17]	0.942	1.000
NPEPPS	101	2	1.98%	196	6	3.06%	1.56 [0.40-6.08]	0.589	0.869
NPHS2	101	5	4.95%	196	19	9.69%	2.06 [0.88-4.83]	0.163	0.535
NPIPB3	101	6	5.94%	196	2	1.02%	0.16 [0.042-0.64]	2.82E-02	0.507
NPIPA1	101	7	6.93%	196	3	1.53%	0.21 [0.066-0.66]	2.55E-02	0.507
NPLOC4	101	2	1.98%	196	12	6.12%	3.23 [0.90-11.53]	0.130	0.535
NPL	101	5	4.95%	196	19	9.69%	2.06 [0.88-4.83]	0.163	0.535
NPR1	101	7	6.93%	196	12	6.12%	0.88 [0.39-1.97]	0.788	0.996
NPRL3	101	7	6.93%	196	4	2.04%	0.28 [0.098-0.80]	4.63E-02	0.507
NPSR1	101	0	0.00%	196	5	2.55%	2.2e+07 [0e+00-Inf]	0.992	1.000

Symbol	CINSARC Low			CINSARC High			CINSARC High vs. Low		
	N tested	N altered	Fq alteration	N tested	N altered	Fq alteration	OR [95%CI]	p-value*	q-value
NPTX1	101	2	1.98%	196	13	6.63%	3.52 [0.99-12.47]	0.102	0.507
NPVF	101	0	0.00%	196	6	3.06%	2.7e+07 [0e+00-Inf]	0.992	1.000
NPW	101	6	5.94%	196	5	2.55%	0.41 [0.15-1.15]	0.154	0.535
NPY	101	0	0.00%	196	6	3.06%	2.7e+07 [0e+00-Inf]	0.992	1.000
NR1D1	101	5	4.95%	196	9	4.59%	0.92 [0.36-2.37]	0.890	1.000
NR1I3	101	6	5.94%	196	16	8.16%	1.41 [0.62-3.18]	0.490	0.805
NR2E1	101	1	0.99%	196	5	2.55%	2.62 [0.43-16.05]	0.383	0.750
NR2F2	101	0	0.00%	196	5	2.55%	2.2e+07 [0e+00-Inf]	0.992	1.000
NR5A2	101	6	5.94%	196	23	11.73%	2.11 [0.96-4.60]	0.118	0.526
NRBP2	101	16	15.84%	196	51	26.02%	1.87 [1.11-3.15]	4.89E-02	0.507
NRG1	101	3	2.97%	196	8	4.08%	1.39 [0.45-4.31]	0.632	0.909
NSL1	101	6	5.94%	196	21	10.71%	1.90 [0.86-4.19]	0.181	0.535
NSMAF	101	14	13.86%	196	33	16.84%	1.26 [0.71-2.22]	0.506	0.805
NSMCE1	101	5	4.95%	196	3	1.53%	0.30 [0.088-1.01]	0.103	0.507
NSMCE2	101	16	15.84%	196	58	29.59%	2.23 [1.33-3.74]	1.06E-02	0.507
NT5C3A	101	0	0.00%	196	8	4.08%	3.6e+07 [0e+00-Inf]	0.992	1.000
NT5C	101	5	4.95%	196	12	6.12%	1.25 [0.51-3.08]	0.681	0.943
NTAN1	101	7	6.93%	196	3	1.53%	0.21 [0.066-0.66]	2.55E-02	0.507
NTHL1	101	6	5.94%	196	5	2.55%	0.41 [0.15-1.15]	0.154	0.535
NTN3	101	6	5.94%	196	5	2.55%	0.41 [0.15-1.15]	0.154	0.535
NTRK1	101	7	6.93%	196	8	4.08%	0.57 [0.24-1.37]	0.294	0.650
NTRK3	101	1	0.99%	196	5	2.55%	2.62 [0.43-16.05]	0.383	0.750
NTSR1	101	7	6.93%	196	13	6.63%	0.95 [0.43-2.12]	0.923	1.000
NUAK2	101	7	6.93%	196	27	13.78%	2.15 [1.03-4.45]	0.085	0.507
NUBP1	101	5	4.95%	196	3	1.53%	0.30 [0.088-1.01]	0.103	0.507
NUBP2	101	6	5.94%	196	5	2.55%	0.41 [0.15-1.15]	0.154	0.535
NUBPL	101	1	0.99%	196	8	4.08%	4.26 [0.73-24.65]	0.175	0.535
NUCB2	101	1	0.99%	196	4	2.04%	2.08 [0.33-13.25]	0.514	0.805
NUCKS1	101	7	6.93%	196	27	13.78%	2.15 [1.03-4.45]	0.085	0.507
NUDCD1	101	15	14.85%	196	54	27.55%	2.18 [1.28-3.70]	1.56E-02	0.507
NUDCD3	101	1	0.99%	196	4	2.04%	2.08 [0.33-13.25]	0.514	0.805
NUDT13	101	1	0.99%	196	5	2.55%	2.62 [0.43-16.05]	0.383	0.750
NUDT16L1	101	5	4.95%	196	6	3.06%	0.61 [0.22-1.68]	0.418	0.779
NUDT17	101	5	4.95%	196	10	5.10%	1.03 [0.41-2.60]	0.955	1.000
NUDT4	101	0	0.00%	196	5	2.55%	2.2e+07 [0e+00-Inf]	0.992	1.000
NUDT8	101	10	9.90%	196	19	9.69%	0.98 [0.50-1.92]	0.955	1.000
NUF2	101	5	4.95%	196	13	6.63%	1.36 [0.56-3.32]	0.566	0.852
NUFIP2	101	3	2.97%	196	5	2.55%	0.86 [0.25-2.89]	0.833	1.000
NUMA1	101	5	4.95%	196	5	2.55%	0.50 [0.17-1.45]	0.286	0.650
NUP107	101	2	1.98%	196	17	8.67%	4.70 [1.35-16.35]	4.11E-02	0.507
NUP133	101	6	5.94%	196	19	9.69%	1.70 [0.77-3.78]	0.274	0.643
NUP210L	101	7	6.93%	196	13	6.63%	0.95 [0.43-2.12]	0.923	1.000
NUP54	101	1	0.99%	196	5	2.55%	2.62 [0.43-16.05]	0.383	0.750
NUP85	101	5	4.95%	196	13	6.63%	1.36 [0.56-3.32]	0.566	0.852
NUPL2	101	0	0.00%	196	6	3.06%	2.7e+07 [0e+00-Inf]	0.992	1.000
NUPR1	101	5	4.95%	196	3	1.53%	0.30 [0.088-1.01]	0.103	0.507
NVL	101	7	6.93%	196	17	8.67%	1.28 [0.59-2.75]	0.602	0.887
NXPH3	101	5	4.95%	196	16	8.16%	1.71 [0.72-4.07]	0.311	0.680
OAZ3	101	6	5.94%	196	12	6.12%	1.03 [0.44-2.41]	0.950	1.000
OBSCN	101	6	5.94%	196	21	10.71%	1.90 [0.86-4.19]	0.181	0.535
OC90	101	15	14.85%	196	55	28.06%	2.24 [1.32-3.80]	1.24E-02	0.507
OCLM	101	5	4.95%	196	16	8.16%	1.71 [0.72-4.07]	0.311	0.680
ODF1	101	16	15.84%	196	57	29.08%	2.18 [1.30-3.66]	1.33E-02	0.507
TENM4	101	6	5.94%	196	13	6.63%	1.12 [0.49-2.60]	0.817	1.000
OGFR	101	7	6.93%	196	14	7.14%	1.03 [0.47-2.28]	0.946	1.000
OLFML2B	101	5	4.95%	196	16	8.16%	1.71 [0.72-4.07]	0.311	0.680
OMP	101	7	6.93%	196	21	10.71%	1.61 [0.76-3.40]	0.294	0.650
OPA1	101	3	2.97%	196	3	1.53%	0.51 [0.13-1.98]	0.412	0.774
OPLAH	101	17	16.83%	196	51	26.02%	1.74 [1.04-2.90]	0.076	0.507
OPN3	101	4	3.96%	196	20	10.20%	2.76 [1.09-6.95]	0.071	0.507
OPRK1	101	12	11.88%	196	29	14.80%	1.29 [0.70-2.36]	0.491	0.805
OPRL1	101	7	6.93%	196	11	5.61%	0.80 [0.35-1.82]	0.652	0.928
OPTC	101	7	6.93%	196	28	14.29%	2.24 [1.08-4.63]	0.068	0.507
OR10J1	101	6	5.94%	196	10	5.10%	0.85 [0.36-2.04]	0.762	0.996
OR10J3	101	6	5.94%	196	9	4.59%	0.76 [0.31-1.86]	0.616	0.898
OR10J5	101	6	5.94%	196	10	5.10%	0.85 [0.36-2.04]	0.762	0.996
OR10K1	101	6	5.94%	196	10	5.10%	0.85 [0.36-2.04]	0.762	0.996
OR10K2	101	6	5.94%	196	10	5.10%	0.85 [0.36-2.04]	0.762	0.996
OR10R2	101	6	5.94%	196	10	5.10%	0.85 [0.36-2.04]	0.762	0.996
OR10T2	101	7	6.93%	196	10	5.10%	0.72 [0.31-1.67]	0.522	0.815
OR10X1	101	6	5.94%	196	10	5.10%	0.85 [0.36-2.04]	0.762	0.996

Symbol	CINSARC Low			CINSARC High			CINSARC High vs. Low		
	N tested	N altered	Fq alteration	N tested	N altered	Fq alteration	OR [95%CI]	p-value*	q-value
OR10Z1	101	6	5.94%	196	9	4.59%	0.76 [0.31-1.86]	0.616	0.898
OR11L1	101	5	4.95%	196	21	10.71%	2.30 [0.99-5.36]	0.104	0.507
OR13G1	101	5	4.95%	196	21	10.71%	2.30 [0.99-5.36]	0.104	0.507
OR14A16	101	5	4.95%	196	21	10.71%	2.30 [0.99-5.36]	0.104	0.507
OR14C36	101	5	4.95%	196	20	10.20%	2.18 [0.93-5.10]	0.130	0.535
OR14I1	101	5	4.95%	196	19	9.69%	2.06 [0.88-4.83]	0.163	0.535
OR1C1	101	5	4.95%	196	21	10.71%	2.30 [0.99-5.36]	0.104	0.507
OR1F1	101	6	5.94%	196	4	2.04%	0.33 [0.11-0.97]	0.092	0.507
OR1F2P	101	6	5.94%	196	4	2.04%	0.33 [0.11-0.97]	0.092	0.507
OR2A12	101	1	0.99%	196	4	2.04%	2.08 [0.33-13.25]	0.514	0.805
OR2A14	101	1	0.99%	196	4	2.04%	2.08 [0.33-13.25]	0.514	0.805
OR2A1	101	7	6.93%	196	10	5.10%	0.72 [0.31-1.67]	0.522	0.815
OR2A25	101	1	0.99%	196	4	2.04%	2.08 [0.33-13.25]	0.514	0.805
OR2A2	101	1	0.99%	196	4	2.04%	2.08 [0.33-13.25]	0.514	0.805
OR2A5	101	1	0.99%	196	4	2.04%	2.08 [0.33-13.25]	0.514	0.805
OR2A7	101	6	5.94%	196	11	5.61%	0.94 [0.40-2.23]	0.908	1.000
OR2A9P	101	6	5.94%	196	11	5.61%	0.94 [0.40-2.23]	0.908	1.000
OR2AK2	101	5	4.95%	196	21	10.71%	2.30 [0.99-5.36]	0.104	0.507
OR2AT4	101	6	5.94%	196	15	7.65%	1.31 [0.58-2.98]	0.586	0.869
OR2B11	101	5	4.95%	196	19	9.69%	2.06 [0.88-4.83]	0.163	0.535
OR2C1	101	5	4.95%	196	4	2.04%	0.40 [0.13-1.23]	0.179	0.535
OR2C3	101	5	4.95%	196	19	9.69%	2.06 [0.88-4.83]	0.163	0.535
OR2F1	101	1	0.99%	196	4	2.04%	2.08 [0.33-13.25]	0.514	0.805
OR2F2	101	1	0.99%	196	4	2.04%	2.08 [0.33-13.25]	0.514	0.805
OR2G2	101	5	4.95%	196	19	9.69%	2.06 [0.88-4.83]	0.163	0.535
OR2G3	101	5	4.95%	196	19	9.69%	2.06 [0.88-4.83]	0.163	0.535
OR2G6	101	4	3.96%	196	19	9.69%	2.60 [1.03-6.59]	0.090	0.507
OR2L13	101	5	4.95%	196	20	10.20%	2.18 [0.93-5.10]	0.130	0.535
OR2L1P	101	5	4.95%	196	20	10.20%	2.18 [0.93-5.10]	0.130	0.535
OR2L2	101	5	4.95%	196	20	10.20%	2.18 [0.93-5.10]	0.130	0.535
OR2L3	101	5	4.95%	196	20	10.20%	2.18 [0.93-5.10]	0.130	0.535
OR2L8	101	5	4.95%	196	21	10.71%	2.30 [0.99-5.36]	0.104	0.507
OR2M1P	101	5	4.95%	196	20	10.20%	2.18 [0.93-5.10]	0.130	0.535
OR2M2	101	5	4.95%	196	20	10.20%	2.18 [0.93-5.10]	0.130	0.535
OR2M3	101	5	4.95%	196	20	10.20%	2.18 [0.93-5.10]	0.130	0.535
OR2M4	101	5	4.95%	196	21	10.71%	2.30 [0.99-5.36]	0.104	0.507
OR2M5	101	5	4.95%	196	20	10.20%	2.18 [0.93-5.10]	0.130	0.535
OR2M7	101	5	4.95%	196	20	10.20%	2.18 [0.93-5.10]	0.130	0.535
OR2T10	101	5	4.95%	196	17	8.67%	1.82 [0.77-4.32]	0.252	0.623
OR2T11	101	5	4.95%	196	17	8.67%	1.82 [0.77-4.32]	0.252	0.623
OR2T12	101	5	4.95%	196	21	10.71%	2.30 [0.99-5.36]	0.104	0.507
OR2T1	101	5	4.95%	196	20	10.20%	2.18 [0.93-5.10]	0.130	0.535
OR2T27	101	5	4.95%	196	18	9.18%	1.94 [0.82-4.58]	0.203	0.570
OR2T29	101	4	3.96%	196	19	9.69%	2.60 [1.03-6.59]	0.090	0.507
OR2T2	101	6	5.94%	196	22	11.22%	2.00 [0.91-4.39]	0.146	0.535
OR2T33	101	5	4.95%	196	21	10.71%	2.30 [0.99-5.36]	0.104	0.507
OR2T34	101	5	4.95%	196	18	9.18%	1.94 [0.82-4.58]	0.203	0.570
OR2T35	101	6	5.94%	196	16	8.16%	1.41 [0.62-3.18]	0.490	0.805
OR2T3	101	6	5.94%	196	21	10.71%	1.90 [0.86-4.19]	0.181	0.535
OR2T4	101	5	4.95%	196	20	10.20%	2.18 [0.93-5.10]	0.130	0.535
OR2T5	101	4	3.96%	196	19	9.69%	2.60 [1.03-6.59]	0.090	0.507
OR2T6	101	5	4.95%	196	20	10.20%	2.18 [0.93-5.10]	0.130	0.535
OR2T8	101	5	4.95%	196	21	10.71%	2.30 [0.99-5.36]	0.104	0.507
OR2W3	101	6	5.94%	196	21	10.71%	1.90 [0.86-4.19]	0.181	0.535
OR2W5	101	5	4.95%	196	19	9.69%	2.06 [0.88-4.83]	0.163	0.535
OR4C6	101	1	0.99%	196	5	2.55%	2.62 [0.43-16.05]	0.383	0.750
OR4D1	101	6	5.94%	196	24	12.24%	2.21 [1.01-4.82]	0.095	0.507
OR4D2	101	6	5.94%	196	24	12.24%	2.21 [1.01-4.82]	0.095	0.507
OR4F5	101	2	1.98%	196	9	4.59%	2.38 [0.65-8.76]	0.273	0.643
OR4F6	101	3	2.97%	196	2	1.02%	0.34 [0.074-1.53]	0.237	0.599
OR4K1	101	4	3.96%	196	16	8.16%	2.16 [0.84-5.53]	0.180	0.535
OR4K2	101	4	3.96%	196	16	8.16%	2.16 [0.84-5.53]	0.180	0.535
OR4K5	101	4	3.96%	196	16	8.16%	2.16 [0.84-5.53]	0.180	0.535
OR4M1	101	4	3.96%	196	16	8.16%	2.16 [0.84-5.53]	0.180	0.535
OR4M2	101	1	0.99%	196	5	2.55%	2.62 [0.43-16.05]	0.383	0.750
OR4N2	101	4	3.96%	196	16	8.16%	2.16 [0.84-5.53]	0.180	0.535
OR4N3P	101	1	0.99%	196	5	2.55%	2.62 [0.43-16.05]	0.383	0.750
OR4N4	101	1	0.99%	196	5	2.55%	2.62 [0.43-16.05]	0.383	0.750
OR4P4	101	1	0.99%	196	5	2.55%	2.62 [0.43-16.05]	0.383	0.750
OR4Q3	101	4	3.96%	196	16	8.16%	2.16 [0.84-5.53]	0.180	0.535
OR4S2	101	1	0.99%	196	5	2.55%	2.62 [0.43-16.05]	0.383	0.750

Symbol	CINSARC Low			CINSARC High			CINSARC High vs. Low		
	N tested	N altered	Fq alteration	N tested	N altered	Fq alteration	OR [95%CI]	p-value*	q-value
OR52N5	101	3	2.97%	196	2	1.02%	0.34 [0.074-1.53]	0.237	0.599
OR6B1	101	1	0.99%	196	4	2.04%	2.08 [0.33-13.25]	0.514	0.805
OR6F1	101	5	4.95%	196	21	10.71%	2.30 [0.99-5.36]	0.104	0.507
OR6K2	101	6	5.94%	196	9	4.59%	0.76 [0.31-1.86]	0.616	0.898
OR6K3	101	6	5.94%	196	8	4.08%	0.67 [0.27-1.68]	0.476	0.805
OR6K6	101	6	5.94%	196	8	4.08%	0.67 [0.27-1.68]	0.476	0.805
OR6N1	101	6	5.94%	196	8	4.08%	0.67 [0.27-1.68]	0.476	0.805
OR6N2	101	6	5.94%	196	8	4.08%	0.67 [0.27-1.68]	0.476	0.805
OR6P1	101	7	6.93%	196	10	5.10%	0.72 [0.31-1.67]	0.522	0.815
OR6V1	101	2	1.98%	196	4	2.04%	1.03 [0.24-4.35]	0.972	1.000
OR6W1P	101	2	1.98%	196	4	2.04%	1.03 [0.24-4.35]	0.972	1.000
OR6Y1	101	7	6.93%	196	10	5.10%	0.72 [0.31-1.67]	0.522	0.815
OR9A2	101	2	1.98%	196	4	2.04%	1.03 [0.24-4.35]	0.972	1.000
ORAI3	101	4	3.96%	196	6	3.06%	0.77 [0.26-2.26]	0.685	0.943
ORMDL3	101	5	4.95%	196	11	5.61%	1.14 [0.46-2.84]	0.811	1.000
OSBPL2	101	7	6.93%	196	12	6.12%	0.88 [0.39-1.97]	0.788	0.996
OSBPL3	101	0	0.00%	196	7	3.57%	3.2e+07 [0e+00-Inf]	0.992	1.000
OSBPL7	101	1	0.99%	196	10	5.10%	5.38 [0.95-30.54]	0.111	0.511
OSBPL8	101	1	0.99%	196	6	3.06%	3.16 [0.53-18.88]	0.290	0.650
OSGIN2	101	18	17.82%	196	50	25.51%	1.58 [0.95-2.62]	0.137	0.535
OSR2	101	15	14.85%	196	54	27.55%	2.18 [1.28-3.70]	1.56E-02	0.507
OSTM1	101	1	0.99%	196	4	2.04%	2.08 [0.33-13.25]	0.514	0.805
OSTN	101	2	1.98%	196	3	1.53%	0.77 [0.17-3.50]	0.776	0.996
SLC51A	101	4	3.96%	196	3	1.53%	0.38 [0.11-1.35]	0.207	0.573
OTOA	101	6	5.94%	196	2	1.02%	0.16 [0.042-0.64]	2.82E-02	0.507
OTOP2	101	5	4.95%	196	13	6.63%	1.36 [0.56-3.32]	0.566	0.852
OTOP3	101	5	4.95%	196	13	6.63%	1.36 [0.56-3.32]	0.566	0.852
OTUD6B	101	19	18.81%	196	49	25.00%	1.44 [0.87-2.37]	0.231	0.599
OTUD7B	101	8	7.92%	196	14	7.14%	0.89 [0.42-1.91]	0.808	1.000
OXR1	101	18	17.82%	196	52	26.53%	1.67 [1.01-2.76]	0.096	0.507
P2RY12	101	3	2.97%	196	2	1.02%	0.34 [0.074-1.53]	0.237	0.599
P2RY13	101	3	2.97%	196	2	1.02%	0.34 [0.074-1.53]	0.237	0.599
P2RY14	101	3	2.97%	196	2	1.02%	0.34 [0.074-1.53]	0.237	0.599
P2RY2	101	7	6.93%	196	11	5.61%	0.80 [0.35-1.82]	0.652	0.928
P2RY6	101	7	6.93%	196	10	5.10%	0.72 [0.31-1.67]	0.522	0.815
P4HA1	101	1	0.99%	196	4	2.04%	2.08 [0.33-13.25]	0.514	0.805
P4HA3	101	7	6.93%	196	14	7.14%	1.03 [0.47-2.28]	0.946	1.000
P4HB	101	2	1.98%	196	13	6.63%	3.52 [0.99-12.47]	0.102	0.507
POTEM	101	3	2.97%	196	2	1.02%	0.34 [0.074-1.53]	0.237	0.599
PAAF1	101	7	6.93%	196	11	5.61%	0.80 [0.35-1.82]	0.652	0.928
PABPC1L	101	4	3.96%	196	4	2.04%	0.51 [0.16-1.65]	0.342	0.716
PABPC1	101	15	14.85%	196	55	28.06%	2.24 [1.32-3.80]	1.24E-02	0.507
PACS1	101	1	0.99%	196	5	2.55%	2.62 [0.43-16.05]	0.383	0.750
PAG1	101	17	16.83%	196	48	24.49%	1.60 [0.96-2.68]	0.133	0.535
PAK1	101	9	8.91%	196	23	11.73%	1.36 [0.69-2.68]	0.458	0.805
PAK2	101	4	3.96%	196	4	2.04%	0.51 [0.16-1.65]	0.342	0.716
PALB2	101	5	4.95%	196	3	1.53%	0.30 [0.088-1.01]	0.103	0.507
PAPPA2	101	6	5.94%	196	16	8.16%	1.41 [0.62-3.18]	0.490	0.805
PAQR4	101	6	5.94%	196	4	2.04%	0.33 [0.11-0.97]	0.092	0.507
PAQR6	101	6	5.94%	196	9	4.59%	0.76 [0.31-1.86]	0.616	0.898
PAWR	101	1	0.99%	196	4	2.04%	2.08 [0.33-13.25]	0.514	0.805
PARD6B	101	5	4.95%	196	14	7.14%	1.48 [0.61-3.57]	0.467	0.805
PARL	101	3	2.97%	196	2	1.02%	0.34 [0.074-1.53]	0.237	0.599
PARM1	101	2	1.98%	196	4	2.04%	1.03 [0.24-4.35]	0.972	1.000
PARN	101	6	5.94%	196	3	1.53%	0.25 [0.076-0.80]	0.051	0.507
PARP10	101	17	16.83%	196	51	26.02%	1.74 [1.04-2.90]	0.076	0.507
PARP1	101	6	5.94%	196	19	9.69%	1.70 [0.77-3.78]	0.274	0.643
PARP6	101	1	0.99%	196	4	2.04%	2.08 [0.33-13.25]	0.514	0.805
PAX9	101	0	0.00%	196	12	6.12%	5.6e+07 [0e+00-Inf]	0.992	1.000
PBX1	101	6	5.94%	196	13	6.63%	1.12 [0.49-2.60]	0.817	1.000
PBXIP1	101	8	7.92%	196	15	7.65%	0.96 [0.46-2.04]	0.935	1.000
PCDH11Y	101	0	0.00%	196	0	0.00%	1.00 [0e+00-Inf]	1.000	1.000
PCF11	101	1	0.99%	196	4	2.04%	2.08 [0.33-13.25]	0.514	0.805
PCGF2	101	5	4.95%	196	9	4.59%	0.92 [0.36-2.37]	0.890	1.000
PCIF1	101	5	4.95%	196	4	2.04%	0.40 [0.13-1.23]	0.179	0.535
PCK1	101	9	8.91%	196	16	8.16%	0.91 [0.44-1.86]	0.826	1.000
PCMTD1	101	13	12.87%	196	27	13.78%	1.08 [0.60-1.96]	0.829	1.000
PCMTD2	101	8	7.92%	196	10	5.10%	0.63 [0.28-1.40]	0.338	0.716
PCP4L1	101	6	5.94%	196	16	8.16%	1.41 [0.62-3.18]	0.490	0.805
PCSK6	101	1	0.99%	196	4	2.04%	2.08 [0.33-13.25]	0.514	0.805
PCTP	101	8	7.92%	196	21	10.71%	1.39 [0.68-2.85]	0.444	0.805

Symbol	CINSARC Low			CINSARC High			CINSARC High vs. Low		
	N tested	N altered	Fq alteration	N tested	N altered	Fq alteration	OR [95%CI]	p-value*	q-value
PCYT1A	101	4	3.96%	196	3	1.53%	0.38 [0.11-1.35]	0.207	0.573
PCYT2	101	2	1.98%	196	13	6.63%	3.52 [0.99-12.47]	0.102	0.507
PC	101	7	6.93%	196	13	6.63%	0.95 [0.43-2.12]	0.923	1.000
PDC	101	5	4.95%	196	16	8.16%	1.71 [0.72-4.07]	0.311	0.680
PDE1C	101	0	0.00%	196	8	4.08%	3.6e+07 [0e+00-Inf]	0.992	1.000
PDE2A	101	5	4.95%	196	9	4.59%	0.92 [0.36-2.37]	0.890	1.000
PDE4DIP	101	2	1.98%	196	4	2.04%	1.03 [0.24-4.35]	0.972	1.000
PDE6G	101	2	1.98%	196	12	6.12%	3.23 [0.90-11.53]	0.130	0.535
PDE7A	101	15	14.85%	196	35	17.86%	1.25 [0.72-2.17]	0.512	0.805
PDE8A	101	1	0.99%	196	6	3.06%	3.16 [0.53-18.88]	0.290	0.650
PDIA2	101	7	6.93%	196	4	2.04%	0.28 [0.098-0.80]	4.63E-02	0.507
PDIA3P1	101	6	5.94%	196	12	6.12%	1.03 [0.44-2.41]	0.950	1.000
PDILT	101	5	4.95%	196	3	1.53%	0.30 [0.088-1.01]	0.103	0.507
PDK2	101	5	4.95%	196	17	8.67%	1.82 [0.77-4.32]	0.252	0.623
PDP1	101	17	16.83%	196	53	27.04%	1.83 [1.10-3.05]	0.052	0.507
PDPK1	101	6	5.94%	196	5	2.55%	0.41 [0.15-1.15]	0.154	0.535
PDSS2	101	1	0.99%	196	8	4.08%	4.26 [0.73-24.65]	0.175	0.535
PDXDC1	101	5	4.95%	196	3	1.53%	0.30 [0.088-1.01]	0.103	0.507
PDZD9	101	5	4.95%	196	3	1.53%	0.30 [0.088-1.01]	0.103	0.507
PDZK1P1	101	6	5.94%	196	11	5.61%	0.94 [0.40-2.23]	0.908	1.000
PDZK1	101	5	4.95%	196	10	5.10%	1.03 [0.41-2.60]	0.955	1.000
PEA15	101	6	5.94%	196	12	6.12%	1.03 [0.44-2.41]	0.950	1.000
PEAR1	101	6	5.94%	196	8	4.08%	0.67 [0.27-1.68]	0.476	0.805
PECAM1	101	7	6.93%	196	28	14.29%	2.24 [1.08-4.63]	0.068	0.507
PELI3	101	5	4.95%	196	10	5.10%	1.03 [0.41-2.60]	0.955	1.000
PENK	101	14	13.86%	196	31	15.82%	1.17 [0.66-2.07]	0.656	0.931
PEX11A	101	1	0.99%	196	7	3.57%	3.70 [0.63-21.75]	0.224	0.596
PEX11B	101	5	4.95%	196	10	5.10%	1.03 [0.41-2.60]	0.955	1.000
PEX19	101	6	5.94%	196	13	6.63%	1.12 [0.49-2.60]	0.817	1.000
PEX2	101	15	14.85%	196	48	24.49%	1.86 [1.09-3.18]	0.057	0.507
PEX5L	101	4	3.96%	196	2	1.02%	0.25 [0.059-1.05]	0.113	0.511
PF4V1	101	1	0.99%	196	6	3.06%	3.16 [0.53-18.88]	0.290	0.650
PF4	101	2	1.98%	196	6	3.06%	1.56 [0.40-6.08]	0.589	0.869
PFDN2	101	6	5.94%	196	17	8.67%	1.50 [0.67-3.38]	0.407	0.774
PFDN4	101	12	11.88%	196	22	11.22%	0.94 [0.50-1.76]	0.866	1.000
PFKFB2	101	6	5.94%	196	24	12.24%	2.21 [1.01-4.82]	0.095	0.507
PGA3	101	2	1.98%	196	4	2.04%	1.03 [0.24-4.35]	0.972	1.000
PGA4	101	2	1.98%	196	5	2.55%	1.30 [0.32-5.21]	0.759	0.996
PGA5	101	2	1.98%	196	5	2.55%	1.30 [0.32-5.21]	0.759	0.996
PGAP3	101	7	6.93%	196	12	6.12%	0.88 [0.39-1.97]	0.788	0.996
PGBD2	101	5	4.95%	196	19	9.69%	2.06 [0.88-4.83]	0.163	0.535
PGBD5	101	6	5.94%	196	19	9.69%	1.70 [0.77-3.78]	0.274	0.643
CPQ	101	14	13.86%	196	53	27.04%	2.30 [1.34-3.96]	1.14E-02	0.507
PGLYRP3	101	7	6.93%	196	12	6.12%	0.88 [0.39-1.97]	0.788	0.996
PGLYRP4	101	7	6.93%	196	12	6.12%	0.88 [0.39-1.97]	0.788	0.996
PGM2L1	101	7	6.93%	196	13	6.63%	0.95 [0.43-2.12]	0.923	1.000
PGPEP1L	101	1	0.99%	196	10	5.10%	5.38 [0.95-30.54]	0.111	0.511
PGP	101	6	5.94%	196	5	2.55%	0.41 [0.15-1.15]	0.154	0.535
PGS1	101	3	2.97%	196	15	7.65%	2.71 [0.94-7.82]	0.122	0.534
PHACTR1	101	1	0.99%	196	4	2.04%	2.08 [0.33-13.25]	0.514	0.805
PHACTR3	101	10	9.90%	196	14	7.14%	0.70 [0.34-1.43]	0.411	0.774
PHB	101	5	4.95%	196	18	9.18%	1.94 [0.82-4.58]	0.203	0.570
PHF12	101	3	2.97%	196	7	3.57%	1.21 [0.38-3.83]	0.786	0.996
PHF20L1	101	14	13.86%	196	59	30.10%	2.68 [1.56-4.59]	2.64E-03	0.507
PHF20	101	4	3.96%	196	8	4.08%	1.03 [0.37-2.88]	0.960	1.000
PHKB	101	1	0.99%	196	4	2.04%	2.08 [0.33-13.25]	0.514	0.805
PHKG2	101	4	3.96%	196	5	2.55%	0.63 [0.21-1.95]	0.505	0.805
PHLDA1	101	1	0.99%	196	4	2.04%	2.08 [0.33-13.25]	0.514	0.805
PHLDA3	101	7	6.93%	196	23	11.73%	1.79 [0.85-3.74]	0.198	0.570
PHOSPHO1	101	4	3.96%	196	17	8.67%	2.30 [0.90-5.88]	0.143	0.535
PHOX2A	101	5	4.95%	196	9	4.59%	0.92 [0.36-2.37]	0.890	1.000
PI15	101	14	13.86%	196	42	21.43%	1.69 [0.97-2.95]	0.117	0.524
PI3	101	5	4.95%	196	3	1.53%	0.30 [0.088-1.01]	0.103	0.507
PI4KB	101	6	5.94%	196	13	6.63%	1.12 [0.49-2.60]	0.817	1.000
PIAS3	101	5	4.95%	196	10	5.10%	1.03 [0.41-2.60]	0.955	1.000
PIGC	101	5	4.95%	196	14	7.14%	1.48 [0.61-3.57]	0.467	0.805
PIGM	101	6	5.94%	196	11	5.61%	0.94 [0.40-2.23]	0.908	1.000
PIGQ	101	7	6.93%	196	4	2.04%	0.28 [0.098-0.80]	4.63E-02	0.507
PIGR	101	7	6.93%	196	25	12.76%	1.96 [0.94-4.09]	0.131	0.535
PIGS	101	3	2.97%	196	8	4.08%	1.39 [0.45-4.31]	0.632	0.909
PIGT	101	5	4.95%	196	3	1.53%	0.30 [0.088-1.01]	0.103	0.507

Symbol	CINSARC Low			CINSARC High			CINSARC High vs. Low		
	N tested	N altered	Fq alteration	N tested	N altered	Fq alteration	OR [95%CI]	p-value*	q-value
PIGU	101	3	2.97%	196	5	2.55%	0.86 [0.25-2.89]	0.833	1.000
PIGW	101	2	1.98%	196	5	2.55%	1.30 [0.32-5.21]	0.759	0.996
PIGX	101	4	3.96%	196	5	2.55%	0.63 [0.21-1.95]	0.505	0.805
PIGZ	101	5	4.95%	196	4	2.04%	0.40 [0.13-1.23]	0.179	0.535
PIK3C2B	101	7	6.93%	196	27	13.78%	2.15 [1.03-4.45]	0.085	0.507
PIK3CA	101	4	3.96%	196	3	1.53%	0.38 [0.11-1.35]	0.207	0.573
PIP4K2B	101	4	3.96%	196	10	5.10%	1.30 [0.48-3.53]	0.661	0.935
PIP5K1A	101	6	5.94%	196	13	6.63%	1.12 [0.49-2.60]	0.817	1.000
PIPOX	101	3	2.97%	196	7	3.57%	1.21 [0.38-3.83]	0.786	0.996
PIP	101	2	1.98%	196	4	2.04%	1.03 [0.24-4.35]	0.972	1.000
PITPNC1	101	9	8.91%	196	29	14.80%	1.78 [0.91-3.44]	0.155	0.535
PITPNM1	101	11	10.89%	196	20	10.20%	0.93 [0.48-1.79]	0.854	1.000
PKD1	101	6	5.94%	196	5	2.55%	0.41 [0.15-1.15]	0.154	0.535
PKHD1L1	101	14	13.86%	196	54	27.55%	2.36 [1.37-4.06]	9.03E-03	0.507
PKIA	101	14	13.86%	196	46	23.47%	1.91 [1.10-3.30]	0.053	0.507
PKIG	101	5	4.95%	196	4	2.04%	0.40 [0.13-1.23]	0.179	0.535
PKLR	101	7	6.93%	196	14	7.14%	1.03 [0.47-2.28]	0.946	1.000
PKMYT1	101	6	5.94%	196	4	2.04%	0.33 [0.11-0.97]	0.092	0.507
PKP1	101	6	5.94%	196	24	12.24%	2.21 [1.01-4.82]	0.095	0.507
PLA2G10	101	9	8.91%	196	3	1.53%	0.16 [0.052-0.49]	6.71E-03	0.507
PLA2G4A	101	4	3.96%	196	15	7.65%	2.01 [0.78-5.19]	0.226	0.598
PLAG1	101	14	13.86%	196	31	15.82%	1.17 [0.66-2.07]	0.656	0.931
PLAT	101	14	13.86%	196	33	16.84%	1.26 [0.71-2.22]	0.506	0.805
PLAU	101	0	0.00%	196	5	2.55%	2.2e+07 [0e+00-Inf]	0.992	1.000
PLCG1	101	3	2.97%	196	7	3.57%	1.21 [0.38-3.83]	0.786	0.996
PLCXD1	101	2	1.98%	196	4	2.04%	1.03 [0.24-4.35]	0.972	1.000
PLD5	101	4	3.96%	196	19	9.69%	2.60 [1.03-6.59]	0.090	0.507
PLEC	101	17	16.83%	196	51	26.02%	1.74 [1.04-2.90]	0.076	0.507
PLEKHA2	101	12	11.88%	196	39	19.90%	1.84 [1.03-3.31]	0.086	0.507
PLEKHA6	101	7	6.93%	196	27	13.78%	2.15 [1.03-4.45]	0.085	0.507
PLEKHA7	101	1	0.99%	196	4	2.04%	2.08 [0.33-13.25]	0.514	0.805
PLEKHA8	101	0	0.00%	196	6	3.06%	2.7e+07 [0e+00-Inf]	0.992	1.000
PLEKHB1	101	7	6.93%	196	12	6.12%	0.88 [0.39-1.97]	0.788	0.996
PLEKHF2	101	16	15.84%	196	55	28.06%	2.07 [1.23-3.48]	2.09E-02	0.507
PLEKHG7	101	0	0.00%	196	5	2.55%	2.2e+07 [0e+00-Inf]	0.992	1.000
PLEKHO1	101	8	7.92%	196	14	7.14%	0.89 [0.42-1.91]	0.808	1.000
PLIN1	101	1	0.99%	196	7	3.57%	3.70 [0.63-21.75]	0.224	0.596
PLK1	101	5	4.95%	196	3	1.53%	0.30 [0.088-1.01]	0.103	0.507
PLTP	101	5	4.95%	196	4	2.04%	0.40 [0.13-1.23]	0.179	0.535
PLXDC1	101	4	3.96%	196	12	6.12%	1.58 [0.60-4.18]	0.438	0.798
PLXNA2	101	6	5.94%	196	25	12.76%	2.31 [1.06-5.03]	0.076	0.507
PM20D1	101	7	6.93%	196	24	12.24%	1.87 [0.90-3.92]	0.161	0.535
PMAIP1	101	1	0.99%	196	4	2.04%	2.08 [0.33-13.25]	0.514	0.805
PMEPA1	101	9	8.91%	196	16	8.16%	0.91 [0.44-1.86]	0.826	1.000
PMF1	101	6	5.94%	196	9	4.59%	0.76 [0.31-1.86]	0.616	0.898
PMM2	101	5	4.95%	196	4	2.04%	0.40 [0.13-1.23]	0.179	0.535
PMP2	101	17	16.83%	196	47	23.98%	1.56 [0.93-2.61]	0.158	0.535
PMVK	101	8	7.92%	196	15	7.65%	0.96 [0.46-2.04]	0.935	1.000
PNMT	101	6	5.94%	196	12	6.12%	1.03 [0.44-2.41]	0.950	1.000
PNPO	101	1	0.99%	196	9	4.59%	4.81 [0.84-27.58]	0.139	0.535
POC1B	101	0	0.00%	196	5	2.55%	2.2e+07 [0e+00-Inf]	0.992	1.000
POGK	101	5	4.95%	196	12	6.12%	1.25 [0.51-3.08]	0.681	0.943
POGZ	101	6	5.94%	196	13	6.63%	1.12 [0.49-2.60]	0.817	1.000
POLB	101	14	13.86%	196	32	16.33%	1.21 [0.69-2.15]	0.578	0.867
POLD3	101	5	4.95%	196	12	6.12%	1.25 [0.51-3.08]	0.681	0.943
POLD4	101	11	10.89%	196	17	8.67%	0.78 [0.40-1.52]	0.536	0.834
POLDIP2	101	3	2.97%	196	7	3.57%	1.21 [0.38-3.83]	0.786	0.996
POLG2	101	8	7.92%	196	26	13.27%	1.78 [0.88-3.57]	0.175	0.535
POLG	101	1	0.99%	196	6	3.06%	3.16 [0.53-18.88]	0.290	0.650
POLI	101	2	1.98%	196	3	1.53%	0.77 [0.17-3.50]	0.776	0.996
POLR2H	101	3	2.97%	196	2	1.02%	0.34 [0.074-1.53]	0.237	0.599
POLR2K	101	15	14.85%	196	54	27.55%	2.18 [1.28-3.70]	1.56E-02	0.507
POLR3C	101	5	4.95%	196	10	5.10%	1.03 [0.41-2.60]	0.955	1.000
POLR3E	101	5	4.95%	196	3	1.53%	0.30 [0.088-1.01]	0.103	0.507
POLR3GL	101	5	4.95%	196	10	5.10%	1.03 [0.41-2.60]	0.955	1.000
POLR3K	101	7	6.93%	196	4	2.04%	0.28 [0.098-0.80]	4.63E-02	0.507
POM121L8P	101	4	3.96%	196	2	1.02%	0.25 [0.059-1.05]	0.113	0.511
POMP	101	0	0.00%	196	5	2.55%	2.2e+07 [0e+00-Inf]	0.992	1.000
POP1	101	16	15.84%	196	54	27.55%	2.02 [1.20-3.40]	2.60E-02	0.507
POPDC3	101	2	1.98%	196	6	3.06%	1.56 [0.40-6.08]	0.589	0.869
POTEA	101	12	11.88%	196	24	12.24%	1.03 [0.56-1.92]	0.928	1.000

Symbol	CINSARC Low			CINSARC High			CINSARC High vs. Low		
	N tested	N altered	Fq alteration	N tested	N altered	Fq alteration	OR [95%CI]	p-value*	q-value
POTEB	101	1	0.99%	196	4	2.04%	2.08 [0.33-13.25]	0.514	0.805
POTEG	101	3	2.97%	196	6	3.06%	1.03 [0.32-3.36]	0.965	1.000
POU2F1	101	5	4.95%	196	12	6.12%	1.25 [0.51-3.08]	0.681	0.943
POU5F1B	101	14	13.86%	196	64	32.65%	3.01 [1.76-5.15]	7.10E-04	0.507
PPBPP2	101	2	1.98%	196	6	3.06%	1.56 [0.40-6.08]	0.589	0.869
PPBP	101	2	1.98%	196	6	3.06%	1.56 [0.40-6.08]	0.589	0.869
PPDPF	101	6	5.94%	196	14	7.14%	1.22 [0.53-2.79]	0.696	0.951
PPEF2	101	2	1.98%	196	5	2.55%	1.30 [0.32-5.21]	0.759	0.996
PPFIA1	101	19	18.81%	196	53	27.04%	1.60 [0.97-2.62]	0.119	0.530
PPFIA2	101	1	0.99%	196	4	2.04%	2.08 [0.33-13.25]	0.514	0.805
PPFIA4	101	7	6.93%	196	27	13.78%	2.15 [1.03-4.45]	0.085	0.507
PPIAL4D	101	4	3.96%	196	8	4.08%	1.03 [0.37-2.88]	0.960	1.000
PPIAL4E	101	4	3.96%	196	8	4.08%	1.03 [0.37-2.88]	0.960	1.000
PPL	101	5	4.95%	196	5	2.55%	0.50 [0.17-1.45]	0.286	0.650
PPM1D	101	8	7.92%	196	34	17.35%	2.44 [1.23-4.82]	3.12E-02	0.507
PPM1E	101	7	6.93%	196	29	14.80%	2.33 [1.13-4.81]	0.055	0.507
PPM1H	101	0	0.00%	196	6	3.06%	2.7e+07 [0e+00-Inf]	0.992	1.000
PPME1	101	7	6.93%	196	13	6.63%	0.95 [0.43-2.12]	0.923	1.000
PPOX	101	6	5.94%	196	16	8.16%	1.41 [0.62-3.18]	0.490	0.805
PPP1CA	101	11	10.89%	196	18	9.18%	0.83 [0.43-1.61]	0.639	0.918
PPP1R12A	101	1	0.99%	196	4	2.04%	2.08 [0.33-13.25]	0.514	0.805
PPP1R12B	101	7	6.93%	196	26	13.27%	2.05 [0.99-4.27]	0.106	0.507
PPP1R15B	101	7	6.93%	196	27	13.78%	2.15 [1.03-4.45]	0.085	0.507
PPP1R16A	101	17	16.83%	196	49	25.00%	1.65 [0.98-2.76]	0.111	0.511
PPP1R16B	101	2	1.98%	196	5	2.55%	1.30 [0.32-5.21]	0.759	0.996
PPP1R1B	101	6	5.94%	196	12	6.12%	1.03 [0.44-2.41]	0.950	1.000
PPP1R2	101	3	2.97%	196	3	1.53%	0.51 [0.13-1.98]	0.412	0.774
PPP1R3D	101	10	9.90%	196	13	6.63%	0.65 [0.31-1.33]	0.321	0.700
PPP1R9B	101	5	4.95%	196	17	8.67%	1.82 [0.77-4.32]	0.252	0.623
PPP2CB	101	2	1.98%	196	6	3.06%	1.56 [0.40-6.08]	0.589	0.869
PPP2R3B	101	2	1.98%	196	4	2.04%	1.03 [0.24-4.35]	0.972	1.000
PPP2R3C	101	0	0.00%	196	10	5.10%	4.6e+07 [0e+00-Inf]	0.992	1.000
PPP2R5A	101	7	6.93%	196	21	10.71%	1.61 [0.76-3.40]	0.294	0.650
PPP3CB	101	0	0.00%	196	5	2.55%	2.2e+07 [0e+00-Inf]	0.992	1.000
PPP4C	101	5	4.95%	196	4	2.04%	0.40 [0.13-1.23]	0.179	0.535
PPP4R1L	101	9	8.91%	196	17	8.67%	0.97 [0.48-1.97]	0.945	1.000
DESI2	101	5	4.95%	196	19	9.69%	2.06 [0.88-4.83]	0.163	0.535
PRAC1	101	2	1.98%	196	17	8.67%	4.70 [1.35-16.35]	4.11E-02	0.507
PRCC	101	7	6.93%	196	8	4.08%	0.57 [0.24-1.37]	0.294	0.650
PRCD	101	3	2.97%	196	15	7.65%	2.71 [0.94-7.82]	0.122	0.534
PRCP	101	2	1.98%	196	4	2.04%	1.03 [0.24-4.35]	0.972	1.000
PRDM14	101	16	15.84%	196	39	19.90%	1.32 [0.77-2.26]	0.395	0.770
PRDM1	101	2	1.98%	196	7	3.57%	1.83 [0.48-6.96]	0.455	0.805
PRDX6	101	5	4.95%	196	13	6.63%	1.36 [0.56-3.32]	0.566	0.852
PRELP	101	7	6.93%	196	28	14.29%	2.24 [1.08-4.63]	0.068	0.507
PREP	101	2	1.98%	196	7	3.57%	1.83 [0.48-6.96]	0.455	0.805
PREX1	101	6	5.94%	196	8	4.08%	0.67 [0.27-1.68]	0.476	0.805
PREX2	101	14	13.86%	196	35	17.86%	1.35 [0.77-2.38]	0.381	0.750
PRG4	101	5	4.95%	196	16	8.16%	1.71 [0.72-4.07]	0.311	0.680
HELZ2	101	6	5.94%	196	15	7.65%	1.31 [0.58-2.98]	0.586	0.869
PRKAB2	101	6	5.94%	196	12	6.12%	1.03 [0.44-2.41]	0.950	1.000
PRKAR1A	101	8	7.92%	196	25	12.76%	1.70 [0.84-3.43]	0.213	0.588
PRKCA	101	8	7.92%	196	29	14.80%	2.02 [1.01-4.03]	0.094	0.507
PRKCB	101	5	4.95%	196	3	1.53%	0.30 [0.088-1.01]	0.103	0.507
PRKCI	101	2	1.98%	196	3	1.53%	0.77 [0.17-3.50]	0.776	0.996
PRKD1	101	1	0.99%	196	6	3.06%	3.16 [0.53-18.88]	0.290	0.650
PRKDC	101	14	13.86%	196	27	13.78%	0.99 [0.55-1.78]	0.984	1.000
PRKY	101	0	0.00%	196	0	0.00%	1.00 [0e+00-Inf]	1.000	1.000
PRM1	101	5	4.95%	196	4	2.04%	0.40 [0.13-1.23]	0.179	0.535
PRM2	101	5	4.95%	196	4	2.04%	0.40 [0.13-1.23]	0.179	0.535
PRM3	101	5	4.95%	196	4	2.04%	0.40 [0.13-1.23]	0.179	0.535
PROCA1	101	3	2.97%	196	8	4.08%	1.39 [0.45-4.31]	0.632	0.909
PROCR	101	4	3.96%	196	6	3.06%	0.77 [0.26-2.26]	0.685	0.943
PROX1	101	6	5.94%	196	22	11.22%	2.00 [0.91-4.39]	0.146	0.535
PRPF3	101	8	7.92%	196	15	7.65%	0.96 [0.46-2.04]	0.935	1.000
PRPF6	101	7	6.93%	196	11	5.61%	0.80 [0.35-1.82]	0.652	0.928
PRPSAP1	101	3	2.97%	196	13	6.63%	2.32 [0.79-6.79]	0.197	0.570
PRR11	101	8	7.92%	196	29	14.80%	2.02 [1.01-4.03]	0.094	0.507
PRR14	101	4	3.96%	196	5	2.55%	0.63 [0.21-1.95]	0.505	0.805
PRR15L	101	1	0.99%	196	9	4.59%	4.81 [0.84-27.58]	0.139	0.535
PRR15	101	0	0.00%	196	6	3.06%	2.7e+07 [0e+00-Inf]	0.992	1.000

Symbol	CINSARC Low			CINSARC High			CINSARC High vs. Low		
	N tested	N altered	Fq alteration	N tested	N altered	Fq alteration	OR [95%CI]	p-value*	q-value
PRR25	101	6	5.94%	196	4	2.04%	0.33 [0.11-0.97]	0.092	0.507
PRRT2	101	5	4.95%	196	3	1.53%	0.30 [0.088-1.01]	0.103	0.507
PRRX1	101	4	3.96%	196	13	6.63%	1.72 [0.66-4.51]	0.353	0.722
PRSS1	101	2	1.98%	196	5	2.55%	1.30 [0.32-5.21]	0.759	0.996
PRSS21	101	6	5.94%	196	5	2.55%	0.41 [0.15-1.15]	0.154	0.535
PRSS22	101	6	5.94%	196	5	2.55%	0.41 [0.15-1.15]	0.154	0.535
PRSS23	101	1	0.99%	196	4	2.04%	2.08 [0.33-13.25]	0.514	0.805
PRSS27	101	6	5.94%	196	5	2.55%	0.41 [0.15-1.15]	0.154	0.535
PRSS30P	101	6	5.94%	196	5	2.55%	0.41 [0.15-1.15]	0.154	0.535
PRSS33	101	6	5.94%	196	5	2.55%	0.41 [0.15-1.15]	0.154	0.535
PRSS36	101	4	3.96%	196	5	2.55%	0.63 [0.21-1.95]	0.505	0.805
PRSS38	101	7	6.93%	196	21	10.71%	1.61 [0.76-3.40]	0.294	0.650
PRSS41	101	6	5.94%	196	5	2.55%	0.41 [0.15-1.15]	0.154	0.535
PRSS53	101	4	3.96%	196	6	3.06%	0.77 [0.26-2.26]	0.685	0.943
PRSS8	101	4	3.96%	196	5	2.55%	0.63 [0.21-1.95]	0.505	0.805
PRY2	101	0	0.00%	196	0	0.00%	1.00 [0e+00-Inf]	1.000	1.000
PSAP	101	0	0.00%	196	5	2.55%	2.2e+07 [0e+00-Inf]	0.992	1.000
PSCA	101	16	15.84%	196	49	25.00%	1.77 [1.05-2.99]	0.073	0.507
PSEN2	101	6	5.94%	196	19	9.69%	1.70 [0.77-3.78]	0.274	0.643
PSKH2	101	18	17.82%	196	47	23.98%	1.45 [0.87-2.42]	0.226	0.598
PSMA6	101	0	0.00%	196	9	4.59%	4.1e+07 [0e+00-Inf]	0.992	1.000
PSMA7	101	7	6.93%	196	12	6.12%	0.88 [0.39-1.97]	0.788	0.996
PSMB11	101	1	0.99%	196	4	2.04%	2.08 [0.33-13.25]	0.514	0.805
PSMB3	101	5	4.95%	196	9	4.59%	0.92 [0.36-2.37]	0.890	1.000
PSMB4	101	6	5.94%	196	13	6.63%	1.12 [0.49-2.60]	0.817	1.000
PSMB5	101	1	0.99%	196	4	2.04%	2.08 [0.33-13.25]	0.514	0.805
PSMC5	101	8	7.92%	196	29	14.80%	2.02 [1.01-4.03]	0.094	0.507
PSMD11	101	2	1.98%	196	5	2.55%	1.30 [0.32-5.21]	0.759	0.996
PSMD12	101	10	9.90%	196	27	13.78%	1.45 [0.76-2.77]	0.340	0.716
PSMD2	101	3	2.97%	196	2	1.02%	0.34 [0.074-1.53]	0.237	0.599
PSMD3	101	5	4.95%	196	10	5.10%	1.03 [0.41-2.60]	0.955	1.000
PSMD4	101	6	5.94%	196	13	6.63%	1.12 [0.49-2.60]	0.817	1.000
PTDSS1	101	16	15.84%	196	53	27.04%	1.97 [1.17-3.31]	3.23E-02	0.507
PTGIS	101	7	6.93%	196	10	5.10%	0.72 [0.31-1.67]	0.522	0.815
PTGS2	101	5	4.95%	196	15	7.65%	1.59 [0.66-3.81]	0.382	0.750
PTK2	101	18	17.82%	196	48	24.49%	1.50 [0.90-2.48]	0.192	0.563
PTK6	101	6	5.94%	196	14	7.14%	1.22 [0.53-2.79]	0.696	0.951
PTP4A3	101	18	17.82%	196	48	24.49%	1.50 [0.90-2.48]	0.192	0.563
PTPN14	101	6	5.94%	196	21	10.71%	1.90 [0.86-4.19]	0.181	0.535
PTPN1	101	6	5.94%	196	16	8.16%	1.41 [0.62-3.18]	0.490	0.805
PTPN5	101	1	0.99%	196	4	2.04%	2.08 [0.33-13.25]	0.514	0.805
PTPN7	101	8	7.92%	196	24	12.24%	1.62 [0.80-3.28]	0.258	0.635
PTPRB	101	3	2.97%	196	14	7.14%	2.51 [0.86-7.30]	0.155	0.535
PTPRCAP	101	11	10.89%	196	20	10.20%	0.93 [0.48-1.79]	0.854	1.000
PTPRC	101	5	4.95%	196	23	11.73%	2.55 [1.10-5.90]	0.066	0.507
PTPRR	101	3	2.97%	196	14	7.14%	2.51 [0.86-7.30]	0.155	0.535
PTPRT	101	2	1.98%	196	5	2.55%	1.30 [0.32-5.21]	0.759	0.996
PTPRVP	101	8	7.92%	196	24	12.24%	1.62 [0.80-3.28]	0.258	0.635
PTRH2	101	9	8.91%	196	33	16.84%	2.07 [1.08-3.98]	0.068	0.507
PTTG3P	101	15	14.85%	196	39	19.90%	1.42 [0.82-2.46]	0.287	0.650
PTX4	101	7	6.93%	196	5	2.55%	0.35 [0.13-0.94]	0.081	0.507
PUF60	101	16	15.84%	196	51	26.02%	1.87 [1.11-3.15]	4.89E-02	0.507
PURG	101	2	1.98%	196	6	3.06%	1.56 [0.40-6.08]	0.589	0.869
PVT1	101	14	13.86%	196	65	33.16%	3.08 [1.81-5.27]	5.41E-04	0.507
PXDNL	101	13	12.87%	196	27	13.78%	1.08 [0.60-1.96]	0.829	1.000
PYCARD	101	4	3.96%	196	5	2.55%	0.63 [0.21-1.95]	0.505	0.805
PYCR1	101	2	1.98%	196	13	6.63%	3.52 [0.99-12.47]	0.102	0.507
PYCR2	101	6	5.94%	196	19	9.69%	1.70 [0.77-3.78]	0.274	0.643
PYDC1	101	4	3.96%	196	5	2.55%	0.63 [0.21-1.95]	0.505	0.805
PYDC2	101	3	2.97%	196	3	1.53%	0.51 [0.13-1.98]	0.412	0.774
PYGO2	101	8	7.92%	196	15	7.65%	0.96 [0.46-2.04]	0.935	1.000
PYHIN1	101	6	5.94%	196	8	4.08%	0.67 [0.27-1.68]	0.476	0.805
PYY2	101	4	3.96%	196	6	3.06%	0.77 [0.26-2.26]	0.685	0.943
QPRT	101	5	4.95%	196	3	1.53%	0.30 [0.088-1.01]	0.103	0.507
QRICH2	101	3	2.97%	196	13	6.63%	2.32 [0.79-6.79]	0.197	0.570
QRSL1	101	1	0.99%	196	7	3.57%	3.70 [0.63-21.75]	0.224	0.596
QSER1	101	1	0.99%	196	4	2.04%	2.08 [0.33-13.25]	0.514	0.805
QSOX1	101	6	5.94%	196	19	9.69%	1.70 [0.77-3.78]	0.274	0.643
R3HDML	101	2	1.98%	196	4	2.04%	1.03 [0.24-4.35]	0.972	1.000
RAB11FIP1	101	10	9.90%	196	51	26.02%	3.20 [1.74-5.89]	1.70E-03	0.507
RAB11FIP3	101	7	6.93%	196	4	2.04%	0.28 [0.098-0.80]	4.63E-02	0.507

Symbol	CINSARC Low			CINSARC High			CINSARC High vs. Low		
	N tested	N altered	Fq alteration	N tested	N altered	Fq alteration	OR [95%CI]	p-value*	q-value
RAB13	101	7	6.93%	196	13	6.63%	0.95 [0.43-2.12]	0.923	1.000
RAB19	101	1	0.99%	196	4	2.04%	2.08 [0.33-13.25]	0.514	0.805
RAB1B	101	3	2.97%	196	5	2.55%	0.86 [0.25-2.89]	0.833	1.000
RAB21	101	1	0.99%	196	12	6.12%	6.52 [1.16-36.57]	0.074	0.507
RAB22A	101	9	8.91%	196	17	8.67%	0.97 [0.48-1.97]	0.945	1.000
RAB25	101	6	5.94%	196	9	4.59%	0.76 [0.31-1.86]	0.616	0.898
RAB26	101	6	5.94%	196	5	2.55%	0.41 [0.15-1.15]	0.154	0.535
RAB27B	101	2	1.98%	196	3	1.53%	0.77 [0.17-3.50]	0.776	0.996
RAB2A	101	16	15.84%	196	32	16.33%	1.04 [0.60-1.80]	0.914	1.000
RAB30	101	1	0.99%	196	4	2.04%	2.08 [0.33-13.25]	0.514	0.805
RAB34	101	3	2.97%	196	8	4.08%	1.39 [0.45-4.31]	0.632	0.909
RAB37	101	5	4.95%	196	14	7.14%	1.48 [0.61-3.57]	0.467	0.805
RAB38	101	1	0.99%	196	4	2.04%	2.08 [0.33-13.25]	0.514	0.805
RAB3GAP2	101	6	5.94%	196	19	9.69%	1.70 [0.77-3.78]	0.274	0.643
RAB3IP	101	2	1.98%	196	15	7.65%	4.10 [1.17-14.39]	0.064	0.507
RAB40B	101	2	1.98%	196	14	7.14%	3.81 [1.08-13.43]	0.081	0.507
RAB40C	101	6	5.94%	196	4	2.04%	0.33 [0.11-0.97]	0.092	0.507
RAB4A	101	6	5.94%	196	19	9.69%	1.70 [0.77-3.78]	0.274	0.643
RAB6A	101	8	7.92%	196	11	5.61%	0.69 [0.31-1.53]	0.443	0.805
RABEP2	101	6	5.94%	196	3	1.53%	0.25 [0.076-0.80]	0.051	0.507
RABGAP1L	101	4	3.96%	196	11	5.61%	1.44 [0.54-3.85]	0.540	0.837
RABIF	101	7	6.93%	196	28	14.29%	2.24 [1.08-4.63]	0.068	0.507
RAC3	101	2	1.98%	196	13	6.63%	3.52 [0.99-12.47]	0.102	0.507
RAD21	101	16	15.84%	196	58	29.59%	2.23 [1.33-3.74]	1.06E-02	0.507
RAD51C	101	7	6.93%	196	28	14.29%	2.24 [1.08-4.63]	0.068	0.507
RAD51D	101	1	0.99%	196	6	3.06%	3.16 [0.53-18.88]	0.290	0.650
RAD54B	101	17	16.83%	196	52	26.53%	1.78 [1.07-2.98]	0.063	0.507
RAD9A	101	11	10.89%	196	18	9.18%	0.83 [0.43-1.61]	0.639	0.918
RAE1	101	9	8.91%	196	17	8.67%	0.97 [0.48-1.97]	0.945	1.000
SLC50A1	101	7	6.93%	196	13	6.63%	0.95 [0.43-2.12]	0.923	1.000
RALA	101	1	0.99%	196	4	2.04%	2.08 [0.33-13.25]	0.514	0.805
RALGAPA1	101	0	0.00%	196	9	4.59%	4.1e+07 [0e+00-Inf]	0.992	1.000
RALGAPB	101	4	3.96%	196	4	2.04%	0.51 [0.16-1.65]	0.342	0.716
RALGPS2	101	5	4.95%	196	17	8.67%	1.82 [0.77-4.32]	0.252	0.623
RALYL	101	15	14.85%	196	43	21.94%	1.61 [0.94-2.77]	0.147	0.535
RAP1B	101	2	1.98%	196	17	8.67%	4.70 [1.35-16.35]	4.11E-02	0.507
RAPGEF5	101	0	0.00%	196	6	3.06%	2.7e+07 [0e+00-Inf]	0.992	1.000
RAPGEFL1	101	5	4.95%	196	9	4.59%	0.92 [0.36-2.37]	0.890	1.000
RARA	101	4	3.96%	196	7	3.57%	0.90 [0.31-2.57]	0.867	1.000
RASAL2	101	5	4.95%	196	17	8.67%	1.82 [0.77-4.32]	0.252	0.623
RASL10B	101	2	1.98%	196	4	2.04%	1.03 [0.24-4.35]	0.972	1.000
RASSF3	101	1	0.99%	196	5	2.55%	2.62 [0.43-16.05]	0.383	0.750
RASSF5	101	6	5.94%	196	25	12.76%	2.31 [1.06-5.03]	0.076	0.507
RASSF6	101	1	0.99%	196	6	3.06%	3.16 [0.53-18.88]	0.290	0.650
RAX	101	1	0.99%	196	4	2.04%	2.08 [0.33-13.25]	0.514	0.805
RB1CC1	101	12	11.88%	196	28	14.29%	1.24 [0.67-2.27]	0.566	0.852
RBBP5	101	7	6.93%	196	26	13.27%	2.05 [0.99-4.27]	0.106	0.507
RBBP6	101	5	4.95%	196	3	1.53%	0.30 [0.088-1.01]	0.103	0.507
RBL1	101	3	2.97%	196	2	1.02%	0.34 [0.074-1.53]	0.237	0.599
RBM12B	101	17	16.83%	196	54	27.55%	1.88 [1.13-3.13]	4.21E-02	0.507
RBM12	101	4	3.96%	196	8	4.08%	1.03 [0.37-2.88]	0.960	1.000
RBM14	101	6	5.94%	196	14	7.14%	1.22 [0.53-2.79]	0.696	0.951
RBM34	101	7	6.93%	196	20	10.20%	1.53 [0.72-3.24]	0.355	0.722
RBM38	101	9	8.91%	196	16	8.16%	0.91 [0.44-1.86]	0.826	1.000
RBM39	101	4	3.96%	196	8	4.08%	1.03 [0.37-2.88]	0.960	1.000
RBM4B	101	6	5.94%	196	13	6.63%	1.12 [0.49-2.60]	0.817	1.000
RBM4	101	6	5.94%	196	14	7.14%	1.22 [0.53-2.79]	0.696	0.951
RBM8A	101	5	4.95%	196	10	5.10%	1.03 [0.41-2.60]	0.955	1.000
RBMY1A1	101	0	0.00%	196	0	0.00%	1.00 [0e+00-Inf]	1.000	1.000
RBMY1A3P	101	0	0.00%	196	0	0.00%	1.00 [0e+00-Inf]	1.000	1.000
RBMY1B	101	0	0.00%	196	0	0.00%	1.00 [0e+00-Inf]	1.000	1.000
RBMY1E	101	0	0.00%	196	0	0.00%	1.00 [0e+00-Inf]	1.000	1.000
RBMY1F	101	0	0.00%	196	0	0.00%	1.00 [0e+00-Inf]	1.000	1.000
RBMY1J	101	0	0.00%	196	0	0.00%	1.00 [0e+00-Inf]	1.000	1.000
RBMY2EP	101	0	0.00%	196	0	0.00%	1.00 [0e+00-Inf]	1.000	1.000
RBMY2FP	101	0	0.00%	196	0	0.00%	1.00 [0e+00-Inf]	1.000	1.000
RBMY3AP	101	0	0.00%	196	0	0.00%	1.00 [0e+00-Inf]	1.000	1.000
RBPJL	101	5	4.95%	196	3	1.53%	0.30 [0.088-1.01]	0.103	0.507
RC3H1	101	5	4.95%	196	13	6.63%	1.36 [0.56-3.32]	0.566	0.852
RCE1	101	8	7.92%	196	13	6.63%	0.83 [0.38-1.78]	0.682	0.943
RCHY1	101	1	0.99%	196	5	2.55%	2.62 [0.43-16.05]	0.383	0.750

Symbol	CINSARC Low			CINSARC High			CINSARC High vs. Low		
	N tested	N altered	Fq alteration	N tested	N altered	Fq alteration	OR [95%CI]	p-value*	q-value
RCOR3	101	7	6.93%	196	20	10.20%	1.53 [0.72-3.24]	0.355	0.722
RCSD1	101	5	4.95%	196	12	6.12%	1.25 [0.51-3.08]	0.681	0.943
RD3	101	7	6.93%	196	20	10.20%	1.53 [0.72-3.24]	0.355	0.722
RDH10	101	16	15.84%	196	41	20.92%	1.41 [0.82-2.40]	0.294	0.650
RDM1	101	2	1.98%	196	4	2.04%	1.03 [0.24-4.35]	0.972	1.000
RECQL4	101	17	16.83%	196	49	25.00%	1.65 [0.98-2.76]	0.111	0.511
RECQL5	101	5	4.95%	196	12	6.12%	1.25 [0.51-3.08]	0.681	0.943
RELT	101	7	6.93%	196	12	6.12%	0.88 [0.39-1.97]	0.788	0.996
REN	101	7	6.93%	196	28	14.29%	2.24 [1.08-4.63]	0.068	0.507
RFFL	101	1	0.99%	196	5	2.55%	2.62 [0.43-16.05]	0.383	0.750
RFNG	101	2	1.98%	196	14	7.14%	3.81 [1.08-13.43]	0.081	0.507
RFPL4A	101	2	1.98%	196	5	2.55%	1.30 [0.32-5.21]	0.759	0.996
RFX5	101	6	5.94%	196	13	6.63%	1.12 [0.49-2.60]	0.817	1.000
RGL1	101	5	4.95%	196	20	10.20%	2.18 [0.93-5.10]	0.130	0.535
RGMA	101	0	0.00%	196	6	3.06%	2.7e+07 [0e+00-Inf]	0.992	1.000
RGS11	101	7	6.93%	196	4	2.04%	0.28 [0.098-0.80]	4.63E-02	0.507
RGS13	101	5	4.95%	196	19	9.69%	2.06 [0.88-4.83]	0.163	0.535
RGS16	101	5	4.95%	196	19	9.69%	2.06 [0.88-4.83]	0.163	0.535
RGS18	101	5	4.95%	196	19	9.69%	2.06 [0.88-4.83]	0.163	0.535
RGS19	101	7	6.93%	196	11	5.61%	0.80 [0.35-1.82]	0.652	0.928
RGS1	101	5	4.95%	196	19	9.69%	2.06 [0.88-4.83]	0.163	0.535
RGS20	101	12	11.88%	196	30	15.31%	1.34 [0.73-2.45]	0.423	0.787
RGS21	101	5	4.95%	196	19	9.69%	2.06 [0.88-4.83]	0.163	0.535
RGS22	101	15	14.85%	196	57	29.08%	2.35 [1.39-3.99]	7.74E-03	0.507
RGS2	101	5	4.95%	196	18	9.18%	1.94 [0.82-4.58]	0.203	0.570
RGS4	101	5	4.95%	196	13	6.63%	1.36 [0.56-3.32]	0.566	0.852
RGS5	101	5	4.95%	196	13	6.63%	1.36 [0.56-3.32]	0.566	0.852
RGS7	101	4	3.96%	196	19	9.69%	2.60 [1.03-6.59]	0.090	0.507
RGS8	101	5	4.95%	196	19	9.69%	2.06 [0.88-4.83]	0.163	0.535
RGS9	101	9	8.91%	196	24	12.24%	1.43 [0.72-2.81]	0.388	0.757
RGSL1	101	5	4.95%	196	19	9.69%	2.06 [0.88-4.83]	0.163	0.535
RHBDF1	101	7	6.93%	196	4	2.04%	0.28 [0.098-0.80]	4.63E-02	0.507
RHBDF2	101	3	2.97%	196	15	7.65%	2.71 [0.94-7.82]	0.122	0.534
RHBDL1	101	6	5.94%	196	4	2.04%	0.33 [0.11-0.97]	0.092	0.507
RHBG	101	5	4.95%	196	9	4.59%	0.92 [0.36-2.37]	0.890	1.000
RHCG	101	1	0.99%	196	6	3.06%	3.16 [0.53-18.88]	0.290	0.650
RHOD	101	8	7.92%	196	12	6.12%	0.76 [0.35-1.65]	0.559	0.852
RHOT2	101	6	5.94%	196	4	2.04%	0.33 [0.11-0.97]	0.092	0.507
RHOU	101	6	5.94%	196	19	9.69%	1.70 [0.77-3.78]	0.274	0.643
RHPN1	101	17	16.83%	196	51	26.02%	1.74 [1.04-2.90]	0.076	0.507
RIMS2	101	17	16.83%	196	53	27.04%	1.83 [1.10-3.05]	0.052	0.507
RIMS4	101	4	3.96%	196	4	2.04%	0.51 [0.16-1.65]	0.342	0.716
RIN1	101	4	3.96%	196	6	3.06%	0.77 [0.26-2.26]	0.685	0.943
RIPK2	101	18	17.82%	196	49	25.00%	1.54 [0.93-2.55]	0.163	0.535
RIT1	101	7	6.93%	196	9	4.59%	0.65 [0.27-1.52]	0.401	0.774
RLBP1	101	1	0.99%	196	6	3.06%	3.16 [0.53-18.88]	0.290	0.650
RNASEL	101	5	4.95%	196	19	9.69%	2.06 [0.88-4.83]	0.163	0.535
RNF112	101	4	3.96%	196	1	0.51%	0.12 [0.02-0.79]	0.064	0.507
RNF114	101	8	7.92%	196	14	7.14%	0.89 [0.42-1.91]	0.808	1.000
RNF115	101	5	4.95%	196	10	5.10%	1.03 [0.41-2.60]	0.955	1.000
RNF121	101	5	4.95%	196	6	3.06%	0.61 [0.22-1.68]	0.418	0.779
RNF122	101	4	3.96%	196	11	5.61%	1.44 [0.54-3.85]	0.540	0.837
RNF126P1	101	7	6.93%	196	23	11.73%	1.79 [0.85-3.74]	0.198	0.570
RNF135	101	2	1.98%	196	3	1.53%	0.77 [0.17-3.50]	0.776	0.996
RNF139	101	16	15.84%	196	57	29.08%	2.18 [1.30-3.66]	1.33E-02	0.507
RNF151	101	6	5.94%	196	5	2.55%	0.41 [0.15-1.15]	0.154	0.535
RNF157	101	2	1.98%	196	13	6.63%	3.52 [0.99-12.47]	0.102	0.507
RNF168	101	4	3.96%	196	4	2.04%	0.51 [0.16-1.65]	0.342	0.716
RNF169	101	5	4.95%	196	14	7.14%	1.48 [0.61-3.57]	0.467	0.805
RNF170	101	13	12.87%	196	26	13.27%	1.04 [0.57-1.88]	0.924	1.000
RNF187	101	7	6.93%	196	21	10.71%	1.61 [0.76-3.40]	0.294	0.650
RNF19A	101	15	14.85%	196	55	28.06%	2.24 [1.32-3.80]	1.24E-02	0.507
RNF213	101	5	4.95%	196	13	6.63%	1.36 [0.56-3.32]	0.566	0.852
RNF2	101	5	4.95%	196	18	9.18%	1.94 [0.82-4.58]	0.203	0.570
RNF40	101	4	3.96%	196	5	2.55%	0.63 [0.21-1.95]	0.505	0.805
RNF43	101	7	6.93%	196	29	14.80%	2.33 [1.13-4.81]	0.055	0.507
RNFT1	101	7	6.93%	196	34	17.35%	2.82 [1.38-5.76]	1.72E-02	0.507
RNPEP	101	8	7.92%	196	24	12.24%	1.62 [0.80-3.28]	0.258	0.635
RNPS1	101	6	5.94%	196	5	2.55%	0.41 [0.15-1.15]	0.154	0.535
LAMTOR2	101	6	5.94%	196	9	4.59%	0.76 [0.31-1.86]	0.616	0.898
ROGDI	101	5	4.95%	196	5	2.55%	0.50 [0.17-1.45]	0.286	0.650

Symbol	CINSARC Low			CINSARC High			CINSARC High vs. Low		
	N tested	N altered	Fq alteration	N tested	N altered	Fq alteration	OR [95%CI]	p-value*	q-value
ROMO1	101	4	3.96%	196	8	4.08%	1.03 [0.37-2.88]	0.960	1.000
RORC	101	6	5.94%	196	12	6.12%	1.03 [0.44-2.41]	0.950	1.000
RP1L1	101	2	1.98%	196	3	1.53%	0.77 [0.17-3.50]	0.776	0.996
RP1	101	13	12.87%	196	29	14.80%	1.18 [0.65-2.12]	0.652	0.928
RP9P	101	0	0.00%	196	8	4.08%	3.6e+07 [0e+00-Inf]	0.992	1.000
RP9	101	0	0.00%	196	8	4.08%	3.6e+07 [0e+00-Inf]	0.992	1.000
RPL19	101	4	3.96%	196	12	6.12%	1.58 [0.60-4.18]	0.438	0.798
RPL23A	101	3	2.97%	196	8	4.08%	1.39 [0.45-4.31]	0.632	0.909
RPL23P8	101	0	0.00%	196	6	3.06%	2.7e+07 [0e+00-Inf]	0.992	1.000
RPL23	101	4	3.96%	196	9	4.59%	1.17 [0.43-3.20]	0.801	1.000
RPL28	101	1	0.99%	196	4	2.04%	2.08 [0.33-13.25]	0.514	0.805
RPL30	101	16	15.84%	196	54	27.55%	2.02 [1.20-3.40]	2.60E-02	0.507
RPL31P11	101	6	5.94%	196	16	8.16%	1.41 [0.62-3.18]	0.490	0.805
RPL35A	101	3	2.97%	196	4	2.04%	0.68 [0.19-2.43]	0.619	0.898
RPL38	101	6	5.94%	196	15	7.65%	1.31 [0.58-2.98]	0.586	0.869
RPL3L	101	6	5.94%	196	5	2.55%	0.41 [0.15-1.15]	0.154	0.535
RPL7	101	16	15.84%	196	41	20.92%	1.41 [0.82-2.40]	0.294	0.650
RPL8	101	17	16.83%	196	49	25.00%	1.65 [0.98-2.76]	0.111	0.511
RPLP0P2	101	0	0.00%	196	5	2.55%	2.2e+07 [0e+00-Inf]	0.992	1.000
RPN2	101	5	4.95%	196	3	1.53%	0.30 [0.088-1.01]	0.103	0.507
RPRD1B	101	5	4.95%	196	3	1.53%	0.30 [0.088-1.01]	0.103	0.507
RPRD2	101	8	7.92%	196	15	7.65%	0.96 [0.46-2.04]	0.935	1.000
RPRML	101	1	0.99%	196	5	2.55%	2.62 [0.43-16.05]	0.383	0.750
RPS10P7	101	7	6.93%	196	23	11.73%	1.79 [0.85-3.74]	0.198	0.570
RPS15A	101	6	5.94%	196	4	2.04%	0.33 [0.11-0.97]	0.092	0.507
RPS17	101	1	0.99%	196	4	2.04%	2.08 [0.33-13.25]	0.514	0.805
RPS20	101	14	13.86%	196	31	15.82%	1.17 [0.66-2.07]	0.656	0.931
RPS21	101	6	5.94%	196	11	5.61%	0.94 [0.40-2.23]	0.908	1.000
RPS27	101	7	6.93%	196	13	6.63%	0.95 [0.43-2.12]	0.923	1.000
RPS2P32	101	0	0.00%	196	6	3.06%	2.7e+07 [0e+00-Inf]	0.992	1.000
RPS2	101	6	5.94%	196	5	2.55%	0.41 [0.15-1.15]	0.154	0.535
RPS3	101	5	4.95%	196	16	8.16%	1.71 [0.72-4.07]	0.311	0.680
RPS4Y1	101	0	0.00%	196	0	0.00%	1.00 [0e+00-Inf]	1.000	1.000
RPS4Y2	101	0	0.00%	196	0	0.00%	1.00 [0e+00-Inf]	1.000	1.000
RPS6KB1	101	7	6.93%	196	34	17.35%	2.82 [1.38-5.76]	1.72E-02	0.507
RPS6KB2	101	11	10.89%	196	20	10.20%	0.93 [0.48-1.79]	0.854	1.000
RPS6KC1	101	6	5.94%	196	22	11.22%	2.00 [0.91-4.39]	0.146	0.535
RPSAP52	101	1	0.99%	196	6	3.06%	3.16 [0.53-18.88]	0.290	0.650
RPTN	101	7	6.93%	196	13	6.63%	0.95 [0.43-2.12]	0.923	1.000
RPTOR	101	3	2.97%	196	12	6.12%	2.13 [0.72-6.28]	0.250	0.623
RPUSD1	101	6	5.94%	196	4	2.04%	0.33 [0.11-0.97]	0.092	0.507
RRM2B	101	16	15.84%	196	55	28.06%	2.07 [1.23-3.48]	2.09E-02	0.507
RRN3P1	101	6	5.94%	196	2	1.02%	0.16 [0.042-0.64]	2.82E-02	0.507
RRN3P2	101	5	4.95%	196	3	1.53%	0.30 [0.088-1.01]	0.103	0.507
RRN3P3	101	5	4.95%	196	3	1.53%	0.30 [0.088-1.01]	0.103	0.507
RRN3	101	7	6.93%	196	3	1.53%	0.21 [0.066-0.66]	2.55E-02	0.507
RRP15	101	6	5.94%	196	19	9.69%	1.70 [0.77-3.78]	0.274	0.643
RRS1	101	15	14.85%	196	38	19.39%	1.38 [0.80-2.38]	0.335	0.716
RSAD1	101	4	3.96%	196	20	10.20%	2.76 [1.09-6.95]	0.071	0.507
RSF1	101	10	9.90%	196	21	10.71%	1.09 [0.56-2.13]	0.828	1.000
RSL1D1	101	5	4.95%	196	3	1.53%	0.30 [0.088-1.01]	0.103	0.507
RSPO2	101	16	15.84%	196	50	25.51%	1.82 [1.08-3.07]	0.060	0.507
RTEL1	101	7	6.93%	196	16	8.16%	1.19 [0.55-2.59]	0.707	0.962
RTN4IP1	101	1	0.99%	196	7	3.57%	3.70 [0.63-21.75]	0.224	0.596
SNX29	101	5	4.95%	196	3	1.53%	0.30 [0.088-1.01]	0.103	0.507
SNX29P2	101	4	3.96%	196	4	2.04%	0.51 [0.16-1.65]	0.342	0.716
RUNX1T1	101	17	16.83%	196	48	24.49%	1.60 [0.96-2.68]	0.133	0.535
RUSC1	101	7	6.93%	196	14	7.14%	1.03 [0.47-2.28]	0.946	1.000
RXFP4	101	7	6.93%	196	9	4.59%	0.65 [0.27-1.52]	0.401	0.774
RXRG	101	4	3.96%	196	12	6.12%	1.58 [0.60-4.18]	0.438	0.798
RYR2	101	5	4.95%	196	19	9.69%	2.06 [0.88-4.83]	0.163	0.535
S100A10	101	6	5.94%	196	13	6.63%	1.12 [0.49-2.60]	0.817	1.000
S100A11	101	7	6.93%	196	13	6.63%	0.95 [0.43-2.12]	0.923	1.000
S100A12	101	8	7.92%	196	13	6.63%	0.83 [0.38-1.78]	0.682	0.943
S100A13	101	7	6.93%	196	12	6.12%	0.88 [0.39-1.97]	0.788	0.996
S100A14	101	7	6.93%	196	13	6.63%	0.95 [0.43-2.12]	0.923	1.000
S100A16	101	7	6.93%	196	13	6.63%	0.95 [0.43-2.12]	0.923	1.000
S100A1	101	7	6.93%	196	12	6.12%	0.88 [0.39-1.97]	0.788	0.996
S100A2	101	7	6.93%	196	12	6.12%	0.88 [0.39-1.97]	0.788	0.996
S100A3	101	8	7.92%	196	12	6.12%	0.76 [0.35-1.65]	0.559	0.852
S100A4	101	8	7.92%	196	12	6.12%	0.76 [0.35-1.65]	0.559	0.852

Symbol	CINSARC Low			CINSARC High			CINSARC High vs. Low		
	N tested	N altered	Fq alteration	N tested	N altered	Fq alteration	OR [95%CI]	p-value*	q-value
S100A5	101	8	7.92%	196	12	6.12%	0.76 [0.35-1.65]	0.559	0.852
S100A6	101	8	7.92%	196	12	6.12%	0.76 [0.35-1.65]	0.559	0.852
S100A7A	101	7	6.93%	196	12	6.12%	0.88 [0.39-1.97]	0.788	0.996
S100A7L2	101	7	6.93%	196	12	6.12%	0.88 [0.39-1.97]	0.788	0.996
S100A7	101	7	6.93%	196	12	6.12%	0.88 [0.39-1.97]	0.788	0.996
S100A8	101	7	6.93%	196	12	6.12%	0.88 [0.39-1.97]	0.788	0.996
S100A9	101	7	6.93%	196	12	6.12%	0.88 [0.39-1.97]	0.788	0.996
SALL4	101	6	5.94%	196	18	9.18%	1.60 [0.72-3.57]	0.335	0.716
SAMD10	101	6	5.94%	196	11	5.61%	0.94 [0.40-2.23]	0.908	1.000
SAMD12	101	15	14.85%	196	56	28.57%	2.29 [1.35-3.89]	9.81E-03	0.507
SAMD14	101	5	4.95%	196	17	8.67%	1.82 [0.77-4.32]	0.252	0.623
SAMD8	101	2	1.98%	196	6	3.06%	1.56 [0.40-6.08]	0.589	0.869
SAMHD1	101	3	2.97%	196	5	2.55%	0.86 [0.25-2.89]	0.833	1.000
SAP30BP	101	5	4.95%	196	12	6.12%	1.25 [0.51-3.08]	0.681	0.943
PPP6R3	101	7	6.93%	196	23	11.73%	1.79 [0.85-3.74]	0.198	0.570
SARM1	101	3	2.97%	196	7	3.57%	1.21 [0.38-3.83]	0.786	0.996
SBF1P1	101	14	13.86%	196	30	15.31%	1.12 [0.63-2.00]	0.740	0.996
SBK1	101	5	4.95%	196	3	1.53%	0.30 [0.088-1.01]	0.103	0.507
SBK2	101	2	1.98%	196	4	2.04%	1.03 [0.24-4.35]	0.972	1.000
SCAMP3	101	7	6.93%	196	14	7.14%	1.03 [0.47-2.28]	0.946	1.000
SCAND1	101	4	3.96%	196	8	4.08%	1.03 [0.37-2.88]	0.960	1.000
SCAND2P	101	1	0.99%	196	5	2.55%	2.62 [0.43-16.05]	0.383	0.750
SCARB2	101	1	0.99%	196	5	2.55%	2.62 [0.43-16.05]	0.383	0.750
SCARNA15	101	1	0.99%	196	4	2.04%	2.08 [0.33-13.25]	0.514	0.805
SCARNA16	101	2	1.98%	196	14	7.14%	3.81 [1.08-13.43]	0.081	0.507
SCARNA20	101	7	6.93%	196	33	16.84%	2.72 [1.33-5.57]	2.17E-02	0.507
SCARNA3	101	5	4.95%	196	14	7.14%	1.48 [0.61-3.57]	0.467	0.805
SCARNA4	101	7	6.93%	196	9	4.59%	0.65 [0.27-1.52]	0.401	0.774
SCCPDH	101	5	4.95%	196	18	9.18%	1.94 [0.82-4.58]	0.203	0.570
SCFD1	101	1	0.99%	196	8	4.08%	4.26 [0.73-24.65]	0.175	0.535
SCML4	101	1	0.99%	196	5	2.55%	2.62 [0.43-16.05]	0.383	0.750
SCN4A	101	8	7.92%	196	29	14.80%	2.02 [1.01-4.03]	0.094	0.507
SCNM1	101	7	6.93%	196	13	6.63%	0.95 [0.43-2.12]	0.923	1.000
SCNN1B	101	5	4.95%	196	2	1.02%	0.20 [0.049-0.80]	0.056	0.507
SCNN1G	101	5	4.95%	196	1	0.51%	0.098 [0.016-0.60]	3.55E-02	0.507
SCPEP1	101	7	6.93%	196	22	11.22%	1.70 [0.81-3.57]	0.242	0.610
SCRIB	101	16	15.84%	196	51	26.02%	1.87 [1.11-3.15]	4.89E-02	0.507
SCRN1	101	0	0.00%	196	6	3.06%	2.7e+07 [0e+00-Inf]	0.992	1.000
SCRN2	101	1	0.99%	196	10	5.10%	5.38 [0.95-30.54]	0.111	0.511
SCRT1	101	18	17.82%	196	50	25.51%	1.58 [0.95-2.62]	0.137	0.535
SCYL3	101	4	3.96%	196	12	6.12%	1.58 [0.60-4.18]	0.438	0.798
SDAD1	101	1	0.99%	196	5	2.55%	2.62 [0.43-16.05]	0.383	0.750
SDC2	101	15	14.85%	196	53	27.04%	2.12 [1.25-3.61]	1.95E-02	0.507
SDC4	101	5	4.95%	196	3	1.53%	0.30 [0.088-1.01]	0.103	0.507
SDCBP	101	14	13.86%	196	33	16.84%	1.26 [0.71-2.22]	0.506	0.805
SDCCAG8	101	5	4.95%	196	19	9.69%	2.06 [0.88-4.83]	0.163	0.535
SDF2	101	3	2.97%	196	8	4.08%	1.39 [0.45-4.31]	0.632	0.909
SDHAF2	101	1	0.99%	196	5	2.55%	2.62 [0.43-16.05]	0.383	0.750
SDHAP1	101	6	5.94%	196	3	1.53%	0.25 [0.076-0.80]	0.051	0.507
SDHAP2	101	5	4.95%	196	7	3.57%	0.71 [0.27-1.90]	0.569	0.855
SDHC	101	6	5.94%	196	16	8.16%	1.41 [0.62-3.18]	0.490	0.805
SDK2	101	5	4.95%	196	14	7.14%	1.48 [0.61-3.57]	0.467	0.805
SDR16C5	101	14	13.86%	196	30	15.31%	1.12 [0.63-2.00]	0.740	0.996
SEBOX	101	3	2.97%	196	7	3.57%	1.21 [0.38-3.83]	0.786	0.996
SEC11A	101	1	0.99%	196	5	2.55%	2.62 [0.43-16.05]	0.383	0.750
SEC11C	101	1	0.99%	196	4	2.04%	2.08 [0.33-13.25]	0.514	0.805
SEC14L1	101	2	1.98%	196	14	7.14%	3.81 [1.08-13.43]	0.081	0.507
SEC14L5	101	5	4.95%	196	5	2.55%	0.50 [0.17-1.45]	0.286	0.650
SEC16B	101	5	4.95%	196	16	8.16%	1.71 [0.72-4.07]	0.311	0.680
SEC22B	101	2	1.98%	196	4	2.04%	1.03 [0.24-4.35]	0.972	1.000
SEC24C	101	0	0.00%	196	5	2.55%	2.2e+07 [0e+00-Inf]	0.992	1.000
SEC63	101	1	0.99%	196	4	2.04%	2.08 [0.33-13.25]	0.514	0.805
SECTM1	101	2	1.98%	196	14	7.14%	3.81 [1.08-13.43]	0.081	0.507
SELENBP1	101	6	5.94%	196	13	6.63%	1.12 [0.49-2.60]	0.817	1.000
SELE	101	4	3.96%	196	13	6.63%	1.72 [0.66-4.51]	0.353	0.722
SELL	101	4	3.96%	196	13	6.63%	1.72 [0.66-4.51]	0.353	0.722
SELP	101	4	3.96%	196	13	6.63%	1.72 [0.66-4.51]	0.353	0.722
SEMA4A	101	6	5.94%	196	9	4.59%	0.76 [0.31-1.86]	0.616	0.898
SEMA4B	101	2	1.98%	196	8	4.08%	2.11 [0.56-7.86]	0.352	0.722
SEMA6C	101	7	6.93%	196	13	6.63%	0.95 [0.43-2.12]	0.923	1.000
SEMG1	101	5	4.95%	196	3	1.53%	0.30 [0.088-1.01]	0.103	0.507

Symbol	CINSARC Low			CINSARC High			CINSARC High vs. Low		
	N tested	N altered	Fq alteration	N tested	N altered	Fq alteration	OR [95%CI]	p-value*	q-value
SEMG2	101	5	4.95%	196	3	1.53%	0.30 [0.088-1.01]	0.103	0.507
SENP2	101	3	2.97%	196	2	1.02%	0.34 [0.074-1.53]	0.237	0.599
SENP5	101	4	3.96%	196	4	2.04%	0.51 [0.16-1.65]	0.342	0.716
SEPHS2	101	4	3.96%	196	5	2.55%	0.63 [0.21-1.95]	0.505	0.805
sept-12	101	5	4.95%	196	5	2.55%	0.50 [0.17-1.45]	0.286	0.650
sept-01	101	4	3.96%	196	5	2.55%	0.63 [0.21-1.95]	0.505	0.805
sept-09	101	3	2.97%	196	13	6.63%	2.32 [0.79-6.79]	0.197	0.570
MSRB1	101	6	5.94%	196	5	2.55%	0.41 [0.15-1.15]	0.154	0.535
SERINC3	101	3	2.97%	196	4	2.04%	0.68 [0.19-2.43]	0.619	0.898
SERPINB10	101	3	2.97%	196	3	1.53%	0.51 [0.13-1.98]	0.412	0.774
SERPINB11	101	3	2.97%	196	3	1.53%	0.51 [0.13-1.98]	0.412	0.774
SERPINB12	101	4	3.96%	196	2	1.02%	0.25 [0.059-1.05]	0.113	0.511
SERPINB13	101	4	3.96%	196	2	1.02%	0.25 [0.059-1.05]	0.113	0.511
SERPINB2	101	4	3.96%	196	3	1.53%	0.38 [0.11-1.35]	0.207	0.573
SERPINB3	101	3	2.97%	196	3	1.53%	0.51 [0.13-1.98]	0.412	0.774
SERPINB4	101	3	2.97%	196	2	1.02%	0.34 [0.074-1.53]	0.237	0.599
SERPINB5	101	3	2.97%	196	2	1.02%	0.34 [0.074-1.53]	0.237	0.599
SERPINB7	101	3	2.97%	196	3	1.53%	0.51 [0.13-1.98]	0.412	0.774
SERPINB8	101	3	2.97%	196	3	1.53%	0.51 [0.13-1.98]	0.412	0.774
SERPINC1	101	5	4.95%	196	13	6.63%	1.36 [0.56-3.32]	0.566	0.852
SERPINH1	101	4	3.96%	196	19	9.69%	2.60 [1.03-6.59]	0.090	0.507
SERTAD4	101	6	5.94%	196	21	10.71%	1.90 [0.86-4.19]	0.181	0.535
SESN1	101	1	0.99%	196	4	2.04%	2.08 [0.33-13.25]	0.514	0.805
SETD1A	101	4	3.96%	196	6	3.06%	0.77 [0.26-2.26]	0.685	0.943
SETDB1	101	8	7.92%	196	16	8.16%	1.03 [0.49-2.17]	0.942	1.000
SEZ6L2	101	5	4.95%	196	4	2.04%	0.40 [0.13-1.23]	0.179	0.535
SEZ6	101	3	2.97%	196	6	3.06%	1.03 [0.32-3.36]	0.965	1.000
SF3B2	101	0	0.00%	196	5	2.55%	2.2e+07 [0e+00-Inf]	0.992	1.000
SF3B4	101	7	6.93%	196	13	6.63%	0.95 [0.43-2.12]	0.923	1.000
SFRP1	101	13	12.87%	196	30	15.31%	1.22 [0.68-2.20]	0.573	0.860
SRSF1	101	6	5.94%	196	25	12.76%	2.31 [1.06-5.03]	0.076	0.507
SRSF2	101	3	2.97%	196	13	6.63%	2.32 [0.79-6.79]	0.197	0.570
SRSF6	101	3	2.97%	196	6	3.06%	1.03 [0.32-3.36]	0.965	1.000
SFT2D2	101	4	3.96%	196	13	6.63%	1.72 [0.66-4.51]	0.353	0.722
SFTA3	101	0	0.00%	196	10	5.10%	4.6e+07 [0e+00-Inf]	0.992	1.000
SFTPA1	101	0	0.00%	196	5	2.55%	2.2e+07 [0e+00-Inf]	0.992	1.000
SGCA	101	5	4.95%	196	17	8.67%	1.82 [0.77-4.32]	0.252	0.623
POMK	101	13	12.87%	196	23	11.73%	0.90 [0.49-1.66]	0.776	0.996
SGK2	101	2	1.98%	196	4	2.04%	1.03 [0.24-4.35]	0.972	1.000
SGK3	101	15	14.85%	196	39	19.90%	1.42 [0.82-2.46]	0.287	0.650
SGSH	101	4	3.96%	196	14	7.14%	1.87 [0.72-4.85]	0.283	0.650
SH2B1	101	6	5.94%	196	3	1.53%	0.25 [0.076-0.80]	0.051	0.507
SH2D1B	101	5	4.95%	196	16	8.16%	1.71 [0.72-4.07]	0.311	0.680
SH2D2A	101	7	6.93%	196	8	4.08%	0.57 [0.24-1.37]	0.294	0.650
SH2D7	101	1	0.99%	196	4	2.04%	2.08 [0.33-13.25]	0.514	0.805
SH3BP5L	101	5	4.95%	196	19	9.69%	2.06 [0.88-4.83]	0.163	0.535
SHANK2	101	15	14.85%	196	35	17.86%	1.25 [0.72-2.17]	0.512	0.805
SHARPIN	101	17	16.83%	196	51	26.02%	1.74 [1.04-2.90]	0.076	0.507
SHC1	101	8	7.92%	196	15	7.65%	0.96 [0.46-2.04]	0.935	1.000
SHE	101	7	6.93%	196	12	6.12%	0.88 [0.39-1.97]	0.788	0.996
SHISA4	101	7	6.93%	196	24	12.24%	1.87 [0.90-3.92]	0.161	0.535
SHISA7	101	2	1.98%	196	4	2.04%	1.03 [0.24-4.35]	0.972	1.000
SHISA9	101	5	4.95%	196	4	2.04%	0.40 [0.13-1.23]	0.179	0.535
SIAH2	101	3	2.97%	196	2	1.02%	0.34 [0.074-1.53]	0.237	0.599
SIGLEC14	101	7	6.93%	196	14	7.14%	1.03 [0.47-2.28]	0.946	1.000
SIGLEC9	101	1	0.99%	196	4	2.04%	2.08 [0.33-13.25]	0.514	0.805
SIGMAR1	101	1	0.99%	196	4	2.04%	2.08 [0.33-13.25]	0.514	0.805
SIPA1L2	101	7	6.93%	196	21	10.71%	1.61 [0.76-3.40]	0.294	0.650
SIRPB1	101	11	10.89%	196	21	10.71%	0.98 [0.51-1.88]	0.963	1.000
SIRT7	101	2	1.98%	196	13	6.63%	3.52 [0.99-12.47]	0.102	0.507
SKA2	101	8	7.92%	196	30	15.31%	2.10 [1.06-4.18]	0.076	0.507
SKAP1	101	2	1.98%	196	13	6.63%	3.52 [0.99-12.47]	0.102	0.507
SKAP2	101	0	0.00%	196	6	3.06%	2.7e+07 [0e+00-Inf]	0.992	1.000
SKIL	101	2	1.98%	196	3	1.53%	0.77 [0.17-3.50]	0.776	0.996
SLA2	101	4	3.96%	196	6	3.06%	0.77 [0.26-2.26]	0.685	0.943
SLAMF1	101	6	5.94%	196	14	7.14%	1.22 [0.53-2.79]	0.696	0.951
SLAMF6	101	5	4.95%	196	14	7.14%	1.48 [0.61-3.57]	0.467	0.805
SLAMF7	101	6	5.94%	196	15	7.65%	1.31 [0.58-2.98]	0.586	0.869
SLAMF8	101	6	5.94%	196	10	5.10%	0.85 [0.36-2.04]	0.762	0.996
SLAMF9	101	6	5.94%	196	11	5.61%	0.94 [0.40-2.23]	0.908	1.000
SLA	101	15	14.85%	196	57	29.08%	2.35 [1.39-3.99]	7.74E-03	0.507

Symbol	CINSARC Low			CINSARC High			CINSARC High vs. Low		
	N tested	N altered	Fq alteration	N tested	N altered	Fq alteration	OR [95%CI]	p-value*	q-value
SLC10A5	101	18	17.82%	196	46	23.47%	1.41 [0.85-2.35]	0.263	0.643
SLC12A5	101	5	4.95%	196	4	2.04%	0.40 [0.13-1.23]	0.179	0.535
SLC13A2	101	3	2.97%	196	8	4.08%	1.39 [0.45-4.31]	0.632	0.909
SLC13A3	101	4	3.96%	196	7	3.57%	0.90 [0.31-2.57]	0.867	1.000
SLC16A3	101	3	2.97%	196	13	6.63%	2.32 [0.79-6.79]	0.197	0.570
SLC16A5	101	5	4.95%	196	12	6.12%	1.25 [0.51-3.08]	0.681	0.943
SLC16A6	101	10	9.90%	196	28	14.29%	1.52 [0.80-2.88]	0.286	0.650
SLC17A6	101	3	2.97%	196	2	1.02%	0.34 [0.074-1.53]	0.237	0.599
SLC17A9	101	7	6.93%	196	14	7.14%	1.03 [0.47-2.28]	0.946	1.000
SLC19A2	101	4	3.96%	196	13	6.63%	1.72 [0.66-4.51]	0.353	0.722
SLC1A2	101	1	0.99%	196	4	2.04%	2.08 [0.33-13.25]	0.514	0.805
SLC20A2	101	15	14.85%	196	31	15.82%	1.08 [0.61-1.89]	0.828	1.000
SLC25A10	101	2	1.98%	196	12	6.12%	3.23 [0.90-11.53]	0.130	0.535
SLC25A19	101	5	4.95%	196	11	5.61%	1.14 [0.46-2.84]	0.811	1.000
SLC25A21	101	0	0.00%	196	11	5.61%	5.1e+07 [0e+00-Inf]	0.992	1.000
SLC25A32	101	16	15.84%	196	55	28.06%	2.07 [1.23-3.48]	2.09E-02	0.507
SLC25A44	101	6	5.94%	196	9	4.59%	0.76 [0.31-1.86]	0.616	0.898
SLC26A11	101	4	3.96%	196	14	7.14%	1.87 [0.72-4.85]	0.283	0.650
SLC26A7	101	17	16.83%	196	47	23.98%	1.56 [0.93-2.61]	0.158	0.535
SLC26A9	101	7	6.93%	196	24	12.24%	1.87 [0.90-3.92]	0.161	0.535
SLC27A3	101	7	6.93%	196	12	6.12%	0.88 [0.39-1.97]	0.788	0.996
SLC28A1	101	1	0.99%	196	6	3.06%	3.16 [0.53-18.88]	0.290	0.650
SLC29A2	101	5	4.95%	196	7	3.57%	0.71 [0.27-1.90]	0.569	0.855
SLC2A10	101	5	4.95%	196	7	3.57%	0.71 [0.27-1.90]	0.569	0.855
SLC2A4RG	101	7	6.93%	196	15	7.65%	1.11 [0.51-2.43]	0.822	1.000
SLC30A10	101	6	5.94%	196	20	10.20%	1.80 [0.81-3.98]	0.224	0.596
SLC30A1	101	7	6.93%	196	20	10.20%	1.53 [0.72-3.24]	0.355	0.722
SLC30A8	101	17	16.83%	196	57	29.08%	2.03 [1.22-3.37]	2.23E-02	0.507
SLC32A1	101	2	1.98%	196	5	2.55%	1.30 [0.32-5.21]	0.759	0.996
SLC35B1	101	4	3.96%	196	16	8.16%	2.16 [0.84-5.53]	0.180	0.535
SLC35C2	101	4	3.96%	196	6	3.06%	0.77 [0.26-2.26]	0.685	0.943
SLC35E3	101	2	1.98%	196	17	8.67%	4.70 [1.35-16.35]	4.11E-02	0.507
SLC35F3	101	7	6.93%	196	20	10.20%	1.53 [0.72-3.24]	0.355	0.722
SLC37A3	101	1	0.99%	196	4	2.04%	2.08 [0.33-13.25]	0.514	0.805
SLC38A10	101	2	1.98%	196	12	6.12%	3.23 [0.90-11.53]	0.130	0.535
SLC39A11	101	5	4.95%	196	15	7.65%	1.59 [0.66-3.81]	0.382	0.750
SLC39A1	101	7	6.93%	196	13	6.63%	0.95 [0.43-2.12]	0.923	1.000
SLC39A4	101	18	17.82%	196	49	25.00%	1.54 [0.93-2.55]	0.163	0.535
SLC41A1	101	7	6.93%	196	24	12.24%	1.87 [0.90-3.92]	0.161	0.535
SLC45A3	101	7	6.93%	196	27	13.78%	2.15 [1.03-4.45]	0.085	0.507
SLC45A4	101	18	17.82%	196	49	25.00%	1.54 [0.93-2.55]	0.163	0.535
SLC46A1	101	3	2.97%	196	7	3.57%	1.21 [0.38-3.83]	0.786	0.996
SLC46A3	101	0	0.00%	196	5	2.55%	2.2e+07 [0e+00-Inf]	0.992	1.000
SLC47A1	101	4	3.96%	196	1	0.51%	0.12 [0.02-0.79]	0.064	0.507
SLC47A2	101	4	3.96%	196	1	0.51%	0.12 [0.02-0.79]	0.064	0.507
SLC5A11	101	5	4.95%	196	3	1.53%	0.30 [0.088-1.01]	0.103	0.507
SLC5A2	101	4	3.96%	196	4	2.04%	0.51 [0.16-1.65]	0.342	0.716
SLC6A10P	101	5	4.95%	196	3	1.53%	0.30 [0.088-1.01]	0.103	0.507
SLC6A4	101	2	1.98%	196	5	2.55%	1.30 [0.32-5.21]	0.759	0.996
SLC7A13	101	18	17.82%	196	47	23.98%	1.45 [0.87-2.42]	0.226	0.598
SLC7A5P1	101	5	4.95%	196	3	1.53%	0.30 [0.088-1.01]	0.103	0.507
SLC7A5P2	101	6	5.94%	196	2	1.02%	0.16 [0.042-0.64]	2.82E-02	0.507
SLC9C2	101	5	4.95%	196	13	6.63%	1.36 [0.56-3.32]	0.566	0.852
SLC9A3R1	101	5	4.95%	196	14	7.14%	1.48 [0.61-3.57]	0.467	0.805
SLC9A3R2	101	6	5.94%	196	5	2.55%	0.41 [0.15-1.15]	0.154	0.535
SLC9A8	101	9	8.91%	196	15	7.65%	0.85 [0.41-1.75]	0.707	0.962
SLCO2B1	101	5	4.95%	196	16	8.16%	1.71 [0.72-4.07]	0.311	0.680
SLCO3A1	101	0	0.00%	196	6	3.06%	2.7e+07 [0e+00-Inf]	0.992	1.000
SLCO4A1	101	6	5.94%	196	13	6.63%	1.12 [0.49-2.60]	0.817	1.000
SLCO5A1	101	16	15.84%	196	40	20.41%	1.36 [0.80-2.33]	0.342	0.716
SLFN11	101	1	0.99%	196	4	2.04%	2.08 [0.33-13.25]	0.514	0.805
SLFN5	101	1	0.99%	196	4	2.04%	2.08 [0.33-13.25]	0.514	0.805
SLPI	101	5	4.95%	196	3	1.53%	0.30 [0.088-1.01]	0.103	0.507
SLURP1	101	16	15.84%	196	49	25.00%	1.77 [1.05-2.99]	0.073	0.507
SMARCD2	101	8	7.92%	196	29	14.80%	2.02 [1.01-4.03]	0.094	0.507
SMARCE1	101	4	3.96%	196	5	2.55%	0.63 [0.21-1.95]	0.505	0.805
SMCP	101	7	6.93%	196	11	5.61%	0.80 [0.35-1.82]	0.652	0.928
SMG1	101	5	4.95%	196	4	2.04%	0.40 [0.13-1.23]	0.179	0.535
SMG5	101	6	5.94%	196	9	4.59%	0.76 [0.31-1.86]	0.616	0.898
SMG7	101	5	4.95%	196	19	9.69%	2.06 [0.88-4.83]	0.163	0.535
SMURF2	101	8	7.92%	196	27	13.78%	1.86 [0.93-3.72]	0.143	0.535

Symbol	CINSARC Low			CINSARC High			CINSARC High vs. Low		
	N tested	N altered	Fq alteration	N tested	N altered	Fq alteration	OR [95%CI]	p-value*	q-value
SMYD2	101	6	5.94%	196	22	11.22%	2.00 [0.91-4.39]	0.146	0.535
SMYD3	101	5	4.95%	196	18	9.18%	1.94 [0.82-4.58]	0.203	0.570
SNAI1	101	8	7.92%	196	16	8.16%	1.03 [0.49-2.17]	0.942	1.000
SNAI2	101	13	12.87%	196	25	12.76%	0.99 [0.54-1.81]	0.977	1.000
SNAP47	101	6	5.94%	196	21	10.71%	1.90 [0.86-4.19]	0.181	0.535
SNAPIN	101	7	6.93%	196	12	6.12%	0.88 [0.39-1.97]	0.788	0.996
SNAR-I	101	2	1.98%	196	3	1.53%	0.77 [0.17-3.50]	0.776	0.996
SNF8	101	2	1.98%	196	16	8.16%	4.40 [1.26-15.37]	0.051	0.507
SNHG11	101	4	3.96%	196	4	2.04%	0.51 [0.16-1.65]	0.342	0.716
SNHG6	101	15	14.85%	196	40	20.41%	1.47 [0.85-2.53]	0.245	0.616
SNHG9	101	6	5.94%	196	5	2.55%	0.41 [0.15-1.15]	0.154	0.535
SNN	101	5	4.95%	196	3	1.53%	0.30 [0.088-1.01]	0.103	0.507
SNORA10	101	6	5.94%	196	5	2.55%	0.41 [0.15-1.15]	0.154	0.535
SNORA14B	101	7	6.93%	196	18	9.18%	1.36 [0.63-2.91]	0.509	0.805
SNORA16B	101	7	6.93%	196	21	10.71%	1.61 [0.76-3.40]	0.294	0.650
SNORA21	101	4	3.96%	196	9	4.59%	1.17 [0.43-3.20]	0.801	1.000
SNORA30	101	4	3.96%	196	5	2.55%	0.63 [0.21-1.95]	0.505	0.805
SNORA36B	101	6	5.94%	196	19	9.69%	1.70 [0.77-3.78]	0.274	0.643
SNORA37	101	2	1.98%	196	3	1.53%	0.77 [0.17-3.50]	0.776	0.996
SNORA38B	101	9	8.91%	196	29	14.80%	1.78 [0.91-3.44]	0.155	0.535
SNORA64	101	6	5.94%	196	5	2.55%	0.41 [0.15-1.15]	0.154	0.535
SNORA71A	101	4	3.96%	196	4	2.04%	0.51 [0.16-1.65]	0.342	0.716
SNORA71B	101	4	3.96%	196	4	2.04%	0.51 [0.16-1.65]	0.342	0.716
SNORA71C	101	4	3.96%	196	4	2.04%	0.51 [0.16-1.65]	0.342	0.716
SNORA71D	101	4	3.96%	196	4	2.04%	0.51 [0.16-1.65]	0.342	0.716
SNORA72	101	16	15.84%	196	54	27.55%	2.02 [1.20-3.40]	2.60E-02	0.507
SNORA77	101	7	6.93%	196	28	14.29%	2.24 [1.08-4.63]	0.068	0.507
SNORA78	101	6	5.94%	196	5	2.55%	0.41 [0.15-1.15]	0.154	0.535
SNORD104	101	7	6.93%	196	27	13.78%	2.15 [1.03-4.45]	0.085	0.507
SNORD124	101	5	4.95%	196	11	5.61%	1.14 [0.46-2.84]	0.811	1.000
SNORD12B	101	7	6.93%	196	7	3.57%	0.50 [0.20-1.23]	0.203	0.570
SNORD12C	101	7	6.93%	196	7	3.57%	0.50 [0.20-1.23]	0.203	0.570
SNORD12	101	7	6.93%	196	7	3.57%	0.50 [0.20-1.23]	0.203	0.570
SNORD15A	101	5	4.95%	196	16	8.16%	1.71 [0.72-4.07]	0.311	0.680
SNORD15B	101	4	3.96%	196	16	8.16%	2.16 [0.84-5.53]	0.180	0.535
SNORD1A	101	3	2.97%	196	15	7.65%	2.71 [0.94-7.82]	0.122	0.534
SNORD1B	101	3	2.97%	196	15	7.65%	2.71 [0.94-7.82]	0.122	0.534
SNORD1C	101	3	2.97%	196	15	7.65%	2.71 [0.94-7.82]	0.122	0.534
SNORD42A	101	3	2.97%	196	8	4.08%	1.39 [0.45-4.31]	0.632	0.909
SNORD42B	101	3	2.97%	196	8	4.08%	1.39 [0.45-4.31]	0.632	0.909
SNORD44	101	5	4.95%	196	13	6.63%	1.36 [0.56-3.32]	0.566	0.852
SNORD47	101	5	4.95%	196	13	6.63%	1.36 [0.56-3.32]	0.566	0.852
SNORD4A	101	3	2.97%	196	8	4.08%	1.39 [0.45-4.31]	0.632	0.909
SNORD4B	101	3	2.97%	196	8	4.08%	1.39 [0.45-4.31]	0.632	0.909
SNORD54	101	14	13.86%	196	31	15.82%	1.17 [0.66-2.07]	0.656	0.931
SNORD60	101	6	5.94%	196	5	2.55%	0.41 [0.15-1.15]	0.154	0.535
SNORD66	101	3	2.97%	196	2	1.02%	0.34 [0.074-1.53]	0.237	0.599
SNORD74	101	5	4.95%	196	13	6.63%	1.36 [0.56-3.32]	0.566	0.852
SNORD75	101	5	4.95%	196	13	6.63%	1.36 [0.56-3.32]	0.566	0.852
SNORD76	101	5	4.95%	196	13	6.63%	1.36 [0.56-3.32]	0.566	0.852
SNORD77	101	5	4.95%	196	13	6.63%	1.36 [0.56-3.32]	0.566	0.852
SNORD78	101	5	4.95%	196	13	6.63%	1.36 [0.56-3.32]	0.566	0.852
SNORD79	101	5	4.95%	196	13	6.63%	1.36 [0.56-3.32]	0.566	0.852
SNORD80	101	5	4.95%	196	13	6.63%	1.36 [0.56-3.32]	0.566	0.852
SNORD81	101	5	4.95%	196	13	6.63%	1.36 [0.56-3.32]	0.566	0.852
SNORD87	101	15	14.85%	196	40	20.41%	1.47 [0.85-2.53]	0.245	0.616
SNORD93	101	0	0.00%	196	6	3.06%	2.7e+07 [0e+00-Inf]	0.992	1.000
SNRNP25	101	7	6.93%	196	4	2.04%	0.28 [0.098-0.80]	4.63E-02	0.507
SNRPA1	101	1	0.99%	196	4	2.04%	2.08 [0.33-13.25]	0.514	0.805
SNRPE	101	7	6.93%	196	26	13.27%	2.05 [0.99-4.27]	0.106	0.507
SNTB1	101	14	13.86%	196	53	27.04%	2.30 [1.34-3.96]	1.14E-02	0.507
SNTG1	101	11	10.89%	196	24	12.24%	1.14 [0.60-2.16]	0.732	0.990
SNX10	101	0	0.00%	196	5	2.55%	2.2e+07 [0e+00-Inf]	0.992	1.000
SNX11	101	2	1.98%	196	12	6.12%	3.23 [0.90-11.53]	0.130	0.535
SNX16	101	18	17.82%	196	45	22.96%	1.37 [0.82-2.29]	0.306	0.676
SNX21	101	5	4.95%	196	4	2.04%	0.40 [0.13-1.23]	0.179	0.535
SNX27	101	6	5.94%	196	12	6.12%	1.03 [0.44-2.41]	0.950	1.000
SNX31	101	15	14.85%	196	55	28.06%	2.24 [1.32-3.80]	1.24E-02	0.507
SNX3	101	1	0.99%	196	5	2.55%	2.62 [0.43-16.05]	0.383	0.750
SNX6	101	0	0.00%	196	9	4.59%	4.1e+07 [0e+00-Inf]	0.992	1.000
SOAT1	101	5	4.95%	196	19	9.69%	2.06 [0.88-4.83]	0.163	0.535

Symbol	CINSARC Low			CINSARC High			CINSARC High vs. Low		
	N tested	N altered	Fq alteration	N tested	N altered	Fq alteration	OR [95%CI]	p-value*	q-value
SOBP	101	1	0.99%	196	6	3.06%	3.16 [0.53-18.88]	0.290	0.650
SOCS1	101	5	4.95%	196	4	2.04%	0.40 [0.13-1.23]	0.179	0.535
SOCS3	101	4	3.96%	196	14	7.14%	1.87 [0.72-4.85]	0.283	0.650
SOCS7	101	5	4.95%	196	8	4.08%	0.82 [0.31-2.13]	0.729	0.986
CAPN15	101	7	6.93%	196	4	2.04%	0.28 [0.098-0.80]	4.63E-02	0.507
SOX13	101	7	6.93%	196	28	14.29%	2.24 [1.08-4.63]	0.068	0.507
SOX17	101	13	12.87%	196	29	14.80%	1.18 [0.65-2.12]	0.652	0.928
SOX18	101	7	6.93%	196	11	5.61%	0.80 [0.35-1.82]	0.652	0.928
SOX2-OT	101	4	3.96%	196	2	1.02%	0.25 [0.059-1.05]	0.113	0.511
SOX2	101	4	3.96%	196	2	1.02%	0.25 [0.059-1.05]	0.113	0.511
SOX6	101	1	0.99%	196	4	2.04%	2.08 [0.33-13.25]	0.514	0.805
SOX8	101	6	5.94%	196	4	2.04%	0.33 [0.11-0.97]	0.092	0.507
SOX9	101	5	4.95%	196	16	8.16%	1.71 [0.72-4.07]	0.311	0.680
SP2	101	1	0.99%	196	10	5.10%	5.38 [0.95-30.54]	0.111	0.511
SP4	101	0	0.00%	196	6	3.06%	2.7e+07 [0e+00-Inf]	0.992	1.000
SP6	101	1	0.99%	196	10	5.10%	5.38 [0.95-30.54]	0.111	0.511
SP8	101	0	0.00%	196	6	3.06%	2.7e+07 [0e+00-Inf]	0.992	1.000
SPACA3	101	2	1.98%	196	5	2.55%	1.30 [0.32-5.21]	0.759	0.996
SPAG1	101	15	14.85%	196	54	27.55%	2.18 [1.28-3.70]	1.56E-02	0.507
SPAG4	101	4	3.96%	196	8	4.08%	1.03 [0.37-2.88]	0.960	1.000
SPAG5	101	3	2.97%	196	8	4.08%	1.39 [0.45-4.31]	0.632	0.909
SPAG9	101	4	3.96%	196	18	9.18%	2.45 [0.97-6.23]	0.114	0.512
SPATA17	101	6	5.94%	196	20	10.20%	1.80 [0.81-3.98]	0.224	0.596
SPATA20	101	4	3.96%	196	19	9.69%	2.60 [1.03-6.59]	0.090	0.507
SPATA2	101	9	8.91%	196	15	7.65%	0.85 [0.41-1.75]	0.707	0.962
SPATA8	101	0	0.00%	196	7	3.57%	3.2e+07 [0e+00-Inf]	0.992	1.000
SPATC1	101	17	16.83%	196	51	26.02%	1.74 [1.04-2.90]	0.076	0.507
SPCS2	101	6	5.94%	196	15	7.65%	1.31 [0.58-2.98]	0.586	0.869
SPHAR	101	6	5.94%	196	19	9.69%	1.70 [0.77-3.78]	0.274	0.643
SPHK1	101	3	2.97%	196	15	7.65%	2.71 [0.94-7.82]	0.122	0.534
EPPIN	101	5	4.95%	196	3	1.53%	0.30 [0.088-1.01]	0.103	0.507
SPINT3	101	5	4.95%	196	3	1.53%	0.30 [0.088-1.01]	0.103	0.507
SPINT4	101	5	4.95%	196	3	1.53%	0.30 [0.088-1.01]	0.103	0.507
SPNS1	101	6	5.94%	196	3	1.53%	0.25 [0.076-0.80]	0.051	0.507
SPN	101	5	4.95%	196	3	1.53%	0.30 [0.088-1.01]	0.103	0.507
SPO11	101	9	8.91%	196	17	8.67%	0.97 [0.48-1.97]	0.945	1.000
SPOP	101	5	4.95%	196	16	8.16%	1.71 [0.72-4.07]	0.311	0.680
SPRR1A	101	7	6.93%	196	11	5.61%	0.80 [0.35-1.82]	0.652	0.928
SPRR1B	101	7	6.93%	196	12	6.12%	0.88 [0.39-1.97]	0.788	0.996
SPRR2A	101	7	6.93%	196	12	6.12%	0.88 [0.39-1.97]	0.788	0.996
SPRR2B	101	7	6.93%	196	12	6.12%	0.88 [0.39-1.97]	0.788	0.996
SPRR2C	101	7	6.93%	196	11	5.61%	0.80 [0.35-1.82]	0.652	0.928
SPRR2D	101	7	6.93%	196	12	6.12%	0.88 [0.39-1.97]	0.788	0.996
SPRR2E	101	7	6.93%	196	12	6.12%	0.88 [0.39-1.97]	0.788	0.996
SPRR2F	101	7	6.93%	196	12	6.12%	0.88 [0.39-1.97]	0.788	0.996
SPRR2G	101	7	6.93%	196	11	5.61%	0.80 [0.35-1.82]	0.652	0.928
SPRR3	101	7	6.93%	196	12	6.12%	0.88 [0.39-1.97]	0.788	0.996
SPRR4	101	7	6.93%	196	11	5.61%	0.80 [0.35-1.82]	0.652	0.928
SPSB3	101	6	5.94%	196	5	2.55%	0.41 [0.15-1.15]	0.154	0.535
SPTA1	101	6	5.94%	196	9	4.59%	0.76 [0.31-1.86]	0.616	0.898
SPTBN2	101	7	6.93%	196	13	6.63%	0.95 [0.43-2.12]	0.923	1.000
SQLE	101	16	15.84%	196	57	29.08%	2.18 [1.30-3.66]	1.33E-02	0.507
SRCAP	101	4	3.96%	196	5	2.55%	0.63 [0.21-1.95]	0.505	0.805
SRCIN1	101	5	4.95%	196	8	4.08%	0.82 [0.31-2.13]	0.729	0.986
SRC	101	5	4.95%	196	3	1.53%	0.30 [0.088-1.01]	0.103	0.507
SRGAP1	101	2	1.98%	196	5	2.55%	1.30 [0.32-5.21]	0.759	0.996
SRGAP2	101	6	5.94%	196	24	12.24%	2.21 [1.01-4.82]	0.095	0.507
SRL	101	5	4.95%	196	6	3.06%	0.61 [0.22-1.68]	0.418	0.779
SRMS	101	6	5.94%	196	15	7.65%	1.31 [0.58-2.98]	0.586	0.869
SRP54	101	0	0.00%	196	9	4.59%	4.1e+07 [0e+00-Inf]	0.992	1.000
SRP68	101	4	3.96%	196	13	6.63%	1.72 [0.66-4.51]	0.353	0.722
SRP9	101	6	5.94%	196	18	9.18%	1.60 [0.72-3.57]	0.335	0.716
SRRM2	101	6	5.94%	196	5	2.55%	0.41 [0.15-1.15]	0.154	0.535
SRY	101	0	0.00%	196	0	0.00%	1.00 [0e+00-Inf]	1.000	1.000
SS18L1	101	7	6.93%	196	12	6.12%	0.88 [0.39-1.97]	0.788	0.996
SSC5D	101	2	1.98%	196	4	2.04%	1.03 [0.24-4.35]	0.972	1.000
SSH2	101	2	1.98%	196	5	2.55%	1.30 [0.32-5.21]	0.759	0.996
SSH3	101	10	9.90%	196	16	8.16%	0.81 [0.40-1.62]	0.616	0.898
SSR2	101	7	6.93%	196	10	5.10%	0.72 [0.31-1.67]	0.522	0.815
SSTR1	101	0	0.00%	196	9	4.59%	4.1e+07 [0e+00-Inf]	0.992	1.000
SSTR2	101	5	4.95%	196	18	9.18%	1.94 [0.82-4.58]	0.203	0.570

Symbol	CINSARC Low			CINSARC High			CINSARC High vs. Low		
	N tested	N altered	Fq alteration	N tested	N altered	Fq alteration	OR [95%CI]	p-value*	q-value
SSTR5	101	6	5.94%	196	5	2.55%	0.41 [0.15-1.15]	0.154	0.535
ST18	101	12	11.88%	196	27	13.78%	1.18 [0.64-2.18]	0.647	0.928
ST3GAL1	101	16	15.84%	196	54	27.55%	2.02 [1.20-3.40]	2.60E-02	0.507
ST6GALNAC1	101	3	2.97%	196	15	7.65%	2.71 [0.94-7.82]	0.122	0.534
ST6GALNAC2	101	3	2.97%	196	15	7.65%	2.71 [0.94-7.82]	0.122	0.534
ST8SIA2	101	0	0.00%	196	7	3.57%	3.2e+07 [0e+00-Inf]	0.992	1.000
STAC2	101	5	4.95%	196	12	6.12%	1.25 [0.51-3.08]	0.681	0.943
STARD10	101	5	4.95%	196	10	5.10%	1.03 [0.41-2.60]	0.955	1.000
STARD3	101	6	5.94%	196	12	6.12%	1.03 [0.44-2.41]	0.950	1.000
STARD6	101	2	1.98%	196	3	1.53%	0.77 [0.17-3.50]	0.776	0.996
STAR	101	11	10.89%	196	46	23.47%	2.51 [1.39-4.54]	1.09E-02	0.507
STAU1	101	6	5.94%	196	7	3.57%	0.59 [0.23-1.50]	0.349	0.722
STAU2	101	15	14.85%	196	40	20.41%	1.47 [0.85-2.53]	0.245	0.616
STBD1	101	1	0.99%	196	6	3.06%	3.16 [0.53-18.88]	0.290	0.650
STC2	101	2	1.98%	196	4	2.04%	1.03 [0.24-4.35]	0.972	1.000
STK31	101	0	0.00%	196	6	3.06%	2.7e+07 [0e+00-Inf]	0.992	1.000
STK3	101	16	15.84%	196	53	27.04%	1.97 [1.17-3.31]	3.23E-02	0.507
STK4	101	5	4.95%	196	3	1.53%	0.30 [0.088-1.01]	0.103	0.507
STMN2	101	17	16.83%	196	46	23.47%	1.52 [0.90-2.54]	0.187	0.551
STMN3	101	7	6.93%	196	16	8.16%	1.19 [0.55-2.59]	0.707	0.962
STRADA	101	8	7.92%	196	30	15.31%	2.10 [1.06-4.18]	0.076	0.507
STRN3	101	1	0.99%	196	8	4.08%	4.26 [0.73-24.65]	0.175	0.535
STUB1	101	6	5.94%	196	4	2.04%	0.33 [0.11-0.97]	0.092	0.507
STX16	101	8	7.92%	196	16	8.16%	1.03 [0.49-2.17]	0.942	1.000
STX1B	101	4	3.96%	196	6	3.06%	0.77 [0.26-2.26]	0.685	0.943
STX4	101	4	3.96%	196	6	3.06%	0.77 [0.26-2.26]	0.685	0.943
STX6	101	5	4.95%	196	19	9.69%	2.06 [0.88-4.83]	0.163	0.535
STXBP4	101	8	7.92%	196	18	9.18%	1.18 [0.57-2.44]	0.715	0.972
SULF1	101	16	15.84%	196	40	20.41%	1.36 [0.80-2.33]	0.342	0.716
SULF2	101	5	4.95%	196	8	4.08%	0.82 [0.31-2.13]	0.729	0.986
SULT1A1	101	5	4.95%	196	3	1.53%	0.30 [0.088-1.01]	0.103	0.507
SULT1A2	101	5	4.95%	196	3	1.53%	0.30 [0.088-1.01]	0.103	0.507
SULT1A3	101	4	3.96%	196	3	1.53%	0.38 [0.11-1.35]	0.207	0.573
SUMO1P1	101	12	11.88%	196	25	12.76%	1.08 [0.59-2.01]	0.829	1.000
SUMO1P3	101	6	5.94%	196	13	6.63%	1.12 [0.49-2.60]	0.817	1.000
SUMO2	101	5	4.95%	196	13	6.63%	1.36 [0.56-3.32]	0.566	0.852
SUPT4H1	101	6	5.94%	196	29	14.80%	2.75 [1.28-5.92]	3.02E-02	0.507
SUPT6H	101	3	2.97%	196	8	4.08%	1.39 [0.45-4.31]	0.632	0.909
SUSD4	101	6	5.94%	196	19	9.69%	1.70 [0.77-3.78]	0.274	0.643
SV2A	101	6	5.94%	196	14	7.14%	1.22 [0.53-2.79]	0.696	0.951
SV2B	101	0	0.00%	196	5	2.55%	2.2e+07 [0e+00-Inf]	0.992	1.000
SVIP	101	3	2.97%	196	2	1.02%	0.34 [0.074-1.53]	0.237	0.599
SYBU	101	16	15.84%	196	54	27.55%	2.02 [1.20-3.40]	2.60E-02	0.507
SYCP2	101	9	8.91%	196	13	6.63%	0.73 [0.35-1.53]	0.479	0.805
SYNGR2	101	3	2.97%	196	17	8.67%	3.10 [1.09-8.87]	0.076	0.507
SYNGR3	101	6	5.94%	196	5	2.55%	0.41 [0.15-1.15]	0.154	0.535
SYNM	101	1	0.99%	196	9	4.59%	4.81 [0.84-27.58]	0.139	0.535
SYNPO2L	101	0	0.00%	196	5	2.55%	2.2e+07 [0e+00-Inf]	0.992	1.000
SYNRG	101	2	1.98%	196	6	3.06%	1.56 [0.40-6.08]	0.589	0.869
SYS1-DBNDD2	101	5	4.95%	196	3	1.53%	0.30 [0.088-1.01]	0.103	0.507
SYS1	101	5	4.95%	196	3	1.53%	0.30 [0.088-1.01]	0.103	0.507
SYT11	101	7	6.93%	196	9	4.59%	0.65 [0.27-1.52]	0.401	0.774
SYT12	101	8	7.92%	196	12	6.12%	0.76 [0.35-1.65]	0.559	0.852
SYT14	101	6	5.94%	196	22	11.22%	2.00 [0.91-4.39]	0.146	0.535
SYT17	101	5	4.95%	196	4	2.04%	0.40 [0.13-1.23]	0.179	0.535
SYT2	101	7	6.93%	196	28	14.29%	2.24 [1.08-4.63]	0.068	0.507
SYT7	101	1	0.99%	196	5	2.55%	2.62 [0.43-16.05]	0.383	0.750
SYTL2	101	2	1.98%	196	3	1.53%	0.77 [0.17-3.50]	0.776	0.996
TAC4	101	4	3.96%	196	16	8.16%	2.16 [0.84-5.53]	0.180	0.535
TACC1	101	12	11.88%	196	42	21.43%	2.02 [1.13-3.62]	4.62E-02	0.507
TACO1	101	8	7.92%	196	30	15.31%	2.10 [1.06-4.18]	0.076	0.507
TADA1	101	5	4.95%	196	13	6.63%	1.36 [0.56-3.32]	0.566	0.852
TADA2A	101	2	1.98%	196	6	3.06%	1.56 [0.40-6.08]	0.589	0.869
TAF15	101	2	1.98%	196	4	2.04%	1.03 [0.24-4.35]	0.972	1.000
TAF1A	101	6	5.94%	196	18	9.18%	1.60 [0.72-3.57]	0.335	0.716
TAF2	101	15	14.85%	196	56	28.57%	2.29 [1.35-3.89]	9.81E-03	0.507
TAF4	101	6	5.94%	196	12	6.12%	1.03 [0.44-2.41]	0.950	1.000
TAF5L	101	6	5.94%	196	19	9.69%	1.70 [0.77-3.78]	0.274	0.643
TAGLN2	101	6	5.94%	196	11	5.61%	0.94 [0.40-2.23]	0.908	1.000
TANC2	101	8	7.92%	196	31	15.82%	2.18 [1.10-4.34]	0.061	0.507
TAOK1	101	3	2.97%	196	6	3.06%	1.03 [0.32-3.36]	0.965	1.000

Symbol	CINSARC Low			CINSARC High			CINSARC High vs. Low		
	N tested	N altered	Fq alteration	N tested	N altered	Fq alteration	OR [95%CI]	p-value*	q-value
TAOK2	101	5	4.95%	196	4	2.04%	0.40 [0.13-1.23]	0.179	0.535
TARBP1	101	7	6.93%	196	20	10.20%	1.53 [0.72-3.24]	0.355	0.722
TARP	101	4	3.96%	196	7	3.57%	0.90 [0.31-2.57]	0.867	1.000
TARS2	101	9	8.91%	196	16	8.16%	0.91 [0.44-1.86]	0.826	1.000
TAS2R39	101	2	1.98%	196	4	2.04%	1.03 [0.24-4.35]	0.972	1.000
TAS2R40	101	2	1.98%	196	3	1.53%	0.77 [0.17-3.50]	0.776	0.996
TAS2R43	101	4	3.96%	196	3	1.53%	0.38 [0.11-1.35]	0.207	0.573
TATDN1	101	16	15.84%	196	57	29.08%	2.18 [1.30-3.66]	1.33E-02	0.507
TATDN3	101	6	5.94%	196	21	10.71%	1.90 [0.86-4.19]	0.181	0.535
TAX1BP1	101	0	0.00%	196	7	3.57%	3.2e+07 [0e+00-Inf]	0.992	1.000
TBC1D10B	101	4	3.96%	196	5	2.55%	0.63 [0.21-1.95]	0.505	0.805
TBC1D10C	101	11	10.89%	196	18	9.18%	0.83 [0.43-1.61]	0.639	0.918
TBC1D15	101	2	1.98%	196	11	5.61%	2.94 [0.82-10.59]	0.166	0.535
TBC1D16	101	3	2.97%	196	17	8.67%	3.10 [1.09-8.87]	0.076	0.507
TBC1D24	101	6	5.94%	196	5	2.55%	0.41 [0.15-1.15]	0.154	0.535
TBC1D3B	101	3	2.97%	196	17	8.67%	3.10 [1.09-8.87]	0.076	0.507
TBC1D3C	101	4	3.96%	196	8	4.08%	1.03 [0.37-2.88]	0.960	1.000
TBC1D3G	101	2	1.98%	196	6	3.06%	1.56 [0.40-6.08]	0.589	0.869
TBC1D3H	101	2	1.98%	196	6	3.06%	1.56 [0.40-6.08]	0.589	0.869
TBC1D3P2	101	9	8.91%	196	34	17.35%	2.15 [1.12-4.12]	0.055	0.507
TBC1D3	101	4	3.96%	196	8	4.08%	1.03 [0.37-2.88]	0.960	1.000
TBC1D7	101	1	0.99%	196	4	2.04%	2.08 [0.33-13.25]	0.514	0.805
TBCD	101	2	1.98%	196	15	7.65%	4.10 [1.17-14.39]	0.064	0.507
TBCE	101	7	6.93%	196	20	10.20%	1.53 [0.72-3.24]	0.355	0.722
TBK1	101	1	0.99%	196	4	2.04%	2.08 [0.33-13.25]	0.514	0.805
TBKBP1	101	2	1.98%	196	9	4.59%	2.38 [0.65-8.76]	0.273	0.643
TBL1XR1	101	3	2.97%	196	3	1.53%	0.51 [0.13-1.98]	0.412	0.774
TBL1Y	101	0	0.00%	196	0	0.00%	1.00 [0e+00-Inf]	1.000	1.000
TBL3	101	6	5.94%	196	5	2.55%	0.41 [0.15-1.15]	0.154	0.535
TBX10	101	10	9.90%	196	19	9.69%	0.98 [0.50-1.92]	0.955	1.000
TBX19	101	4	3.96%	196	13	6.63%	1.72 [0.66-4.51]	0.353	0.722
TBX21	101	2	1.98%	196	9	4.59%	2.38 [0.65-8.76]	0.273	0.643
TBX2	101	7	6.93%	196	32	16.33%	2.62 [1.28-5.38]	2.75E-02	0.507
TBX4	101	7	6.93%	196	32	16.33%	2.62 [1.28-5.38]	2.75E-02	0.507
TBX6	101	5	4.95%	196	4	2.04%	0.40 [0.13-1.23]	0.179	0.535
TCAM1P	101	8	7.92%	196	29	14.80%	2.02 [1.01-4.03]	0.094	0.507
TCAP	101	6	5.94%	196	12	6.12%	1.03 [0.44-2.41]	0.950	1.000
TCEA1	101	12	11.88%	196	30	15.31%	1.34 [0.73-2.45]	0.423	0.787
TCEA2	101	7	6.93%	196	11	5.61%	0.80 [0.35-1.82]	0.652	0.928
TCFL5	101	7	6.93%	196	13	6.63%	0.95 [0.43-2.12]	0.923	1.000
TCHHL1	101	7	6.93%	196	13	6.63%	0.95 [0.43-2.12]	0.923	1.000
TCHH	101	7	6.93%	196	13	6.63%	0.95 [0.43-2.12]	0.923	1.000
TCIRG1	101	9	8.91%	196	22	11.22%	1.29 [0.65-2.56]	0.538	0.835
TCTEX1D2	101	4	3.96%	196	3	1.53%	0.38 [0.11-1.35]	0.207	0.573
TDRD10	101	7	6.93%	196	12	6.12%	0.88 [0.39-1.97]	0.788	0.996
TDRD5	101	6	5.94%	196	19	9.69%	1.70 [0.77-3.78]	0.274	0.643
TDRKH	101	6	5.94%	196	12	6.12%	1.03 [0.44-2.41]	0.950	1.000
TEDDM1	101	5	4.95%	196	19	9.69%	2.06 [0.88-4.83]	0.163	0.535
TEKT5	101	5	4.95%	196	3	1.53%	0.30 [0.088-1.01]	0.103	0.507
TELO2	101	7	6.93%	196	5	2.55%	0.35 [0.13-0.94]	0.081	0.507
TERF1	101	16	15.84%	196	42	21.43%	1.45 [0.85-2.47]	0.252	0.623
TEX14	101	8	7.92%	196	29	14.80%	2.02 [1.01-4.03]	0.094	0.507
TEX15	101	2	1.98%	196	7	3.57%	1.83 [0.48-6.96]	0.455	0.805
TEX19	101	2	1.98%	196	13	6.63%	3.52 [0.99-12.47]	0.102	0.507
TEX2	101	7	6.93%	196	28	14.29%	2.24 [1.08-4.63]	0.068	0.507
TFAP2C	101	7	6.93%	196	15	7.65%	1.11 [0.51-2.43]	0.822	1.000
TFAP4	101	5	4.95%	196	6	3.06%	0.61 [0.22-1.68]	0.418	0.779
TFB2M	101	5	4.95%	196	18	9.18%	1.94 [0.82-4.58]	0.203	0.570
TFRC	101	4	3.96%	196	3	1.53%	0.38 [0.11-1.35]	0.207	0.573
TGFB1I1	101	4	3.96%	196	4	2.04%	0.51 [0.16-1.65]	0.342	0.716
TGFB2	101	6	5.94%	196	19	9.69%	1.70 [0.77-3.78]	0.274	0.643
TGIF2LY	101	0	0.00%	196	0	0.00%	1.00 [0e+00-Inf]	1.000	1.000
TGIF2	101	4	3.96%	196	6	3.06%	0.77 [0.26-2.26]	0.685	0.943
TGM2	101	4	3.96%	196	4	2.04%	0.51 [0.16-1.65]	0.342	0.716
TGS1	101	14	13.86%	196	31	15.82%	1.17 [0.66-2.07]	0.656	0.931
TG	101	15	14.85%	196	58	29.59%	2.41 [1.42-4.08]	6.08E-03	0.507
NELFCD	101	9	8.91%	196	15	7.65%	0.85 [0.41-1.75]	0.707	0.962
THAP1	101	13	12.87%	196	27	13.78%	1.08 [0.60-1.96]	0.829	1.000
THAP2	101	1	0.99%	196	12	6.12%	6.52 [1.16-36.57]	0.074	0.507
THAP6	101	1	0.99%	196	5	2.55%	2.62 [0.43-16.05]	0.383	0.750
THBS3	101	7	6.93%	196	13	6.63%	0.95 [0.43-2.12]	0.923	1.000

Symbol	CINSARC Low			CINSARC High			CINSARC High vs. Low		
	N tested	N altered	Fq alteration	N tested	N altered	Fq alteration	OR [95%CI]	p-value*	q-value
THEM4	101	6	5.94%	196	13	6.63%	1.12 [0.49-2.60]	0.817	1.000
THEM5	101	6	5.94%	196	12	6.12%	1.03 [0.44-2.41]	0.950	1.000
THOC3	101	3	2.97%	196	8	4.08%	1.39 [0.45-4.31]	0.632	0.909
ALYREF	101	2	1.98%	196	13	6.63%	3.52 [0.99-12.47]	0.102	0.507
THOC6	101	6	5.94%	196	4	2.04%	0.33 [0.11-0.97]	0.092	0.507
THPO	101	3	2.97%	196	2	1.02%	0.34 [0.074-1.53]	0.237	0.599
THRA	101	5	4.95%	196	9	4.59%	0.92 [0.36-2.37]	0.890	1.000
THRSP	101	9	8.91%	196	20	10.20%	1.16 [0.58-2.32]	0.722	0.980
THUMPD1	101	5	4.95%	196	2	1.02%	0.20 [0.049-0.80]	0.056	0.507
TIAF1	101	3	2.97%	196	5	2.55%	0.86 [0.25-2.89]	0.833	1.000
TIGD5	101	16	15.84%	196	51	26.02%	1.87 [1.11-3.15]	4.89E-02	0.507
TIGD7	101	5	4.95%	196	4	2.04%	0.40 [0.13-1.23]	0.179	0.535
PAM16	101	5	4.95%	196	6	3.06%	0.61 [0.22-1.68]	0.418	0.779
TIMM17A	101	7	6.93%	196	24	12.24%	1.87 [0.90-3.92]	0.161	0.535
TIMP2	101	3	2.97%	196	13	6.63%	2.32 [0.79-6.79]	0.197	0.570
TIPRL	101	4	3.96%	196	13	6.63%	1.72 [0.66-4.51]	0.353	0.722
TK1	101	3	2.97%	196	17	8.67%	3.10 [1.09-8.87]	0.076	0.507
TLCD1	101	3	2.97%	196	8	4.08%	1.39 [0.45-4.31]	0.632	0.909
TLK2	101	8	7.92%	196	34	17.35%	2.44 [1.23-4.82]	3.12E-02	0.507
TLR5	101	6	5.94%	196	19	9.69%	1.70 [0.77-3.78]	0.274	0.643
TM2D2	101	12	11.88%	196	39	19.90%	1.84 [1.03-3.31]	0.086	0.507
TM4SF19	101	4	3.96%	196	3	1.53%	0.38 [0.11-1.35]	0.207	0.573
DCSTAMP	101	17	16.83%	196	50	25.51%	1.69 [1.01-2.83]	0.092	0.507
TMBIM4	101	2	1.98%	196	8	4.08%	2.11 [0.56-7.86]	0.352	0.722
TMC3	101	1	0.99%	196	4	2.04%	2.08 [0.33-13.25]	0.514	0.805
TMC5	101	5	4.95%	196	4	2.04%	0.40 [0.13-1.23]	0.179	0.535
TMC6	101	3	2.97%	196	16	8.16%	2.90 [1.01-8.34]	0.097	0.507
TMC7	101	5	4.95%	196	4	2.04%	0.40 [0.13-1.23]	0.179	0.535
TMC8	101	3	2.97%	196	16	8.16%	2.90 [1.01-8.34]	0.097	0.507
TMCC2	101	7	6.93%	196	26	13.27%	2.05 [0.99-4.27]	0.106	0.507
TMCO1	101	4	3.96%	196	12	6.12%	1.58 [0.60-4.18]	0.438	0.798
TMED10P1	101	16	15.84%	196	47	23.98%	1.68 [0.99-2.84]	0.106	0.507
TMEM100	101	8	7.92%	196	20	10.20%	1.32 [0.64-2.71]	0.525	0.818
TMEM104	101	5	4.95%	196	13	6.63%	1.36 [0.56-3.32]	0.566	0.852
TMEM105	101	2	1.98%	196	12	6.12%	3.23 [0.90-11.53]	0.130	0.535
TMEM114	101	5	4.95%	196	5	2.55%	0.50 [0.17-1.45]	0.286	0.650
TMEM126A	101	3	2.97%	196	3	1.53%	0.51 [0.13-1.98]	0.412	0.774
TMEM126B	101	3	2.97%	196	3	1.53%	0.51 [0.13-1.98]	0.412	0.774
TMEM134	101	11	10.89%	196	20	10.20%	0.93 [0.48-1.79]	0.854	1.000
TMEM135	101	2	1.98%	196	5	2.55%	1.30 [0.32-5.21]	0.759	0.996
TMEM138	101	1	0.99%	196	4	2.04%	2.08 [0.33-13.25]	0.514	0.805
TMEM150B	101	1	0.99%	196	4	2.04%	2.08 [0.33-13.25]	0.514	0.805
TMEM151A	101	2	1.98%	196	6	3.06%	1.56 [0.40-6.08]	0.589	0.869
TMEM159	101	5	4.95%	196	2	1.02%	0.20 [0.049-0.80]	0.056	0.507
TMEM183A	101	7	6.93%	196	27	13.78%	2.15 [1.03-4.45]	0.085	0.507
TMEM186	101	5	4.95%	196	4	2.04%	0.40 [0.13-1.23]	0.179	0.535
TMEM189-UE	101	6	5.94%	196	14	7.14%	1.22 [0.53-2.79]	0.696	0.951
TMEM189	101	6	5.94%	196	14	7.14%	1.22 [0.53-2.79]	0.696	0.951
TMEM190	101	1	0.99%	196	4	2.04%	2.08 [0.33-13.25]	0.514	0.805
TMEM199	101	3	2.97%	196	7	3.57%	1.21 [0.38-3.83]	0.786	0.996
TMEM19	101	1	0.99%	196	12	6.12%	6.52 [1.16-36.57]	0.074	0.507
TMEM202	101	1	0.99%	196	4	2.04%	2.08 [0.33-13.25]	0.514	0.805
TMEM204	101	6	5.94%	196	5	2.55%	0.41 [0.15-1.15]	0.154	0.535
TMEM206	101	7	6.93%	196	21	10.71%	1.61 [0.76-3.40]	0.294	0.650
TMEM207	101	2	1.98%	196	3	1.53%	0.77 [0.17-3.50]	0.776	0.996
TMEM216	101	1	0.99%	196	4	2.04%	2.08 [0.33-13.25]	0.514	0.805
TMEM219	101	5	4.95%	196	4	2.04%	0.40 [0.13-1.23]	0.179	0.535
TMEM41A	101	3	2.97%	196	2	1.02%	0.34 [0.074-1.53]	0.237	0.599
TMEM44	101	3	2.97%	196	3	1.53%	0.51 [0.13-1.98]	0.412	0.774
VMP1	101	9	8.91%	196	34	17.35%	2.15 [1.12-4.12]	0.055	0.507
TMEM63A	101	6	5.94%	196	20	10.20%	1.80 [0.81-3.98]	0.224	0.596
TMEM64	101	18	17.82%	196	47	23.98%	1.45 [0.87-2.42]	0.226	0.598
TMEM65	101	16	15.84%	196	57	29.08%	2.18 [1.30-3.66]	1.33E-02	0.507
TMEM67	101	17	16.83%	196	54	27.55%	1.88 [1.13-3.13]	4.21E-02	0.507
TMEM68	101	14	13.86%	196	30	15.31%	1.12 [0.63-2.00]	0.740	0.996
TMEM70	101	17	16.83%	196	41	20.92%	1.31 [0.77-2.21]	0.401	0.774
TMEM71	101	15	14.85%	196	58	29.59%	2.41 [1.42-4.08]	6.08E-03	0.507
TMEM74	101	15	14.85%	196	51	26.02%	2.02 [1.18-3.43]	3.03E-02	0.507
TMEM79	101	6	5.94%	196	9	4.59%	0.76 [0.31-1.86]	0.616	0.898
TMEM81	101	7	6.93%	196	26	13.27%	2.05 [0.99-4.27]	0.106	0.507
TMEM86A	101	1	0.99%	196	4	2.04%	2.08 [0.33-13.25]	0.514	0.805

Symbol	CINSARC Low			CINSARC High			CINSARC High vs. Low		
	N tested	N altered	Fq alteration	N tested	N altered	Fq alteration	OR [95%CI]	p-value*	q-value
TMEM8A	101	7	6.93%	196	4	2.04%	0.28 [0.098-0.80]	4.63E-02	0.507
TMEM92	101	4	3.96%	196	18	9.18%	2.45 [0.97-6.23]	0.114	0.512
TMEM97	101	4	3.96%	196	7	3.57%	0.90 [0.31-2.57]	0.867	1.000
TMEM98	101	2	1.98%	196	4	2.04%	1.03 [0.24-4.35]	0.972	1.000
TMEM99	101	2	1.98%	196	4	2.04%	1.03 [0.24-4.35]	0.972	1.000
TMEM9	101	6	5.94%	196	24	12.24%	2.21 [1.01-4.82]	0.095	0.507
TMIGD1	101	2	1.98%	196	5	2.55%	1.30 [0.32-5.21]	0.759	0.996
TMOD4	101	7	6.93%	196	13	6.63%	0.95 [0.43-2.12]	0.923	1.000
TMSB4Y	101	0	0.00%	196	0	0.00%	1.00 [0e+00-Inf]	1.000	1.000
TMTC3	101	0	0.00%	196	5	2.55%	2.2e+07 [0e+00-Inf]	0.992	1.000
TNFAIP1	101	3	2.97%	196	7	3.57%	1.21 [0.38-3.83]	0.786	0.996
TNFAIP8L2	101	7	6.93%	196	13	6.63%	0.95 [0.43-2.12]	0.923	1.000
TNFRSF11A	101	3	2.97%	196	2	1.02%	0.34 [0.074-1.53]	0.237	0.599
TNFRSF11B	101	15	14.85%	196	57	29.08%	2.35 [1.39-3.99]	7.74E-03	0.507
TNFRSF12A	101	6	5.94%	196	4	2.04%	0.33 [0.11-0.97]	0.092	0.507
TNFRSF17	101	5	4.95%	196	3	1.53%	0.30 [0.088-1.01]	0.103	0.507
TNFRSF6B	101	7	6.93%	196	15	7.65%	1.11 [0.51-2.43]	0.822	1.000
TNFSF18	101	5	4.95%	196	15	7.65%	1.59 [0.66-3.81]	0.382	0.750
TNFSF4	101	5	4.95%	196	13	6.63%	1.36 [0.56-3.32]	0.566	0.852
TNK2	101	4	3.96%	196	3	1.53%	0.38 [0.11-1.35]	0.207	0.573
TNNC2	101	5	4.95%	196	4	2.04%	0.40 [0.13-1.23]	0.179	0.535
TNNI1	101	7	6.93%	196	25	12.76%	1.96 [0.94-4.09]	0.131	0.535
TNNT2	101	6	5.94%	196	24	12.24%	2.21 [1.01-4.82]	0.095	0.507
TNN	101	5	4.95%	196	16	8.16%	1.71 [0.72-4.07]	0.311	0.680
TNP2	101	5	4.95%	196	4	2.04%	0.40 [0.13-1.23]	0.179	0.535
TNRC6A	101	5	4.95%	196	3	1.53%	0.30 [0.088-1.01]	0.103	0.507
TNRC6C	101	3	2.97%	196	16	8.16%	2.90 [1.01-8.34]	0.097	0.507
TNR	101	5	4.95%	196	17	8.67%	1.82 [0.77-4.32]	0.252	0.623
TNS4	101	4	3.96%	196	5	2.55%	0.63 [0.21-1.95]	0.505	0.805
TOB1	101	4	3.96%	196	20	10.20%	2.76 [1.09-6.95]	0.071	0.507
TOM1L1	101	7	6.93%	196	16	8.16%	1.19 [0.55-2.59]	0.707	0.962
TOMM20	101	7	6.93%	196	19	9.69%	1.44 [0.68-3.07]	0.427	0.791
TOMM34	101	4	3.96%	196	4	2.04%	0.51 [0.16-1.65]	0.342	0.716
TOMM40L	101	6	5.94%	196	16	8.16%	1.41 [0.62-3.18]	0.490	0.805
TOMM7	101	0	0.00%	196	6	3.06%	2.7e+07 [0e+00-Inf]	0.992	1.000
TOP1MT	101	17	16.83%	196	51	26.02%	1.74 [1.04-2.90]	0.076	0.507
TOP1P1	101	5	4.95%	196	13	6.63%	1.36 [0.56-3.32]	0.566	0.852
TOP1	101	3	2.97%	196	7	3.57%	1.21 [0.38-3.83]	0.786	0.996
TOP2A	101	4	3.96%	196	6	3.06%	0.77 [0.26-2.26]	0.685	0.943
TOR1AIP1	101	6	5.94%	196	19	9.69%	1.70 [0.77-3.78]	0.274	0.643
TOR1AIP2	101	6	5.94%	196	19	9.69%	1.70 [0.77-3.78]	0.274	0.643
TOR3A	101	5	4.95%	196	18	9.18%	1.94 [0.82-4.58]	0.203	0.570
TOX2	101	3	2.97%	196	4	2.04%	0.68 [0.19-2.43]	0.619	0.898
TOX	101	15	14.85%	196	33	16.84%	1.16 [0.66-2.03]	0.660	0.935
TP53BP2	101	6	5.94%	196	18	9.18%	1.60 [0.72-3.57]	0.335	0.716
TP53I13	101	3	2.97%	196	5	2.55%	0.86 [0.25-2.89]	0.833	1.000
TP53INP1	101	16	15.84%	196	53	27.04%	1.97 [1.17-3.31]	3.23E-02	0.507
TP53INP2	101	4	3.96%	196	6	3.06%	0.77 [0.26-2.26]	0.685	0.943
TP53RK	101	4	3.96%	196	7	3.57%	0.90 [0.31-2.57]	0.867	1.000
TP53TG3B	101	5	4.95%	196	4	2.04%	0.40 [0.13-1.23]	0.179	0.535
TP53TG5	101	5	4.95%	196	3	1.53%	0.30 [0.088-1.01]	0.103	0.507
TP63	101	2	1.98%	196	3	1.53%	0.77 [0.17-3.50]	0.776	0.996
TPCN2	101	16	15.84%	196	46	23.47%	1.63 [0.96-2.76]	0.128	0.535
TPD52L2	101	6	5.94%	196	12	6.12%	1.03 [0.44-2.41]	0.950	1.000
TPD52	101	17	16.83%	196	48	24.49%	1.60 [0.96-2.68]	0.133	0.535
TPH1	101	1	0.99%	196	4	2.04%	2.08 [0.33-13.25]	0.514	0.805
TPH2	101	2	1.98%	196	11	5.61%	2.94 [0.82-10.59]	0.166	0.535
TPM3	101	7	6.93%	196	12	6.12%	0.88 [0.39-1.97]	0.788	0.996
TPRG1	101	2	1.98%	196	3	1.53%	0.77 [0.17-3.50]	0.776	0.996
TPR	101	5	4.95%	196	16	8.16%	1.71 [0.72-4.07]	0.311	0.680
TPSAB1	101	6	5.94%	196	5	2.55%	0.41 [0.15-1.15]	0.154	0.535
TPSB2	101	6	5.94%	196	5	2.55%	0.41 [0.15-1.15]	0.154	0.535
TPSD1	101	6	5.94%	196	5	2.55%	0.41 [0.15-1.15]	0.154	0.535
TPSG1	101	6	5.94%	196	5	2.55%	0.41 [0.15-1.15]	0.154	0.535
TRA2A	101	0	0.00%	196	6	3.06%	2.7e+07 [0e+00-Inf]	0.992	1.000
TRAF3IP3	101	6	5.94%	196	22	11.22%	2.00 [0.91-4.39]	0.146	0.535
TRAF4	101	3	2.97%	196	9	4.59%	1.57 [0.52-4.80]	0.505	0.805
TRAF5	101	7	6.93%	196	20	10.20%	1.53 [0.72-3.24]	0.355	0.722
TRAF7	101	6	5.94%	196	5	2.55%	0.41 [0.15-1.15]	0.154	0.535
TRAM1	101	16	15.84%	196	40	20.41%	1.36 [0.80-2.33]	0.342	0.716
TRAP1	101	5	4.95%	196	4	2.04%	0.40 [0.13-1.23]	0.179	0.535

Symbol	CINSARC Low			CINSARC High			CINSARC High vs. Low		
	N tested	N altered	Fq alteration	N tested	N altered	Fq alteration	OR [95%CI]	p-value*	q-value
TRAPPC9	101	17	16.83%	196	50	25.51%	1.69 [1.01-2.83]	0.092	0.507
TRHDE	101	2	1.98%	196	11	5.61%	2.94 [0.82-10.59]	0.166	0.535
TRHR	101	15	14.85%	196	52	26.53%	2.07 [1.22-3.52]	2.44E-02	0.507
TRIB1	101	16	15.84%	196	58	29.59%	2.23 [1.33-3.74]	1.06E-02	0.507
TRIL	101	0	0.00%	196	8	4.08%	3.6e+07 [0e+00-Inf]	0.992	1.000
TRIM11	101	7	6.93%	196	21	10.71%	1.61 [0.76-3.40]	0.294	0.650
TRIM17	101	7	6.93%	196	21	10.71%	1.61 [0.76-3.40]	0.294	0.650
TRIM25	101	7	6.93%	196	22	11.22%	1.70 [0.81-3.57]	0.242	0.610
TRIM37	101	8	7.92%	196	29	14.80%	2.02 [1.01-4.03]	0.094	0.507
TRIM46	101	7	6.93%	196	13	6.63%	0.95 [0.43-2.12]	0.923	1.000
TRIM47	101	4	3.96%	196	12	6.12%	1.58 [0.60-4.18]	0.438	0.798
TRIM55	101	15	14.85%	196	37	18.88%	1.33 [0.77-2.31]	0.388	0.757
TRIM58	101	6	5.94%	196	21	10.71%	1.90 [0.86-4.19]	0.181	0.535
TRIM65	101	4	3.96%	196	12	6.12%	1.58 [0.60-4.18]	0.438	0.798
TRIM67	101	6	5.94%	196	19	9.69%	1.70 [0.77-3.78]	0.274	0.643
TRIM72	101	4	3.96%	196	5	2.55%	0.63 [0.21-1.95]	0.505	0.805
TRMT12	101	16	15.84%	196	57	29.08%	2.18 [1.30-3.66]	1.33E-02	0.507
TRPA1	101	15	14.85%	196	41	20.92%	1.52 [0.88-2.61]	0.207	0.573
TRPC4AP	101	4	3.96%	196	8	4.08%	1.03 [0.37-2.88]	0.960	1.000
TRPS1	101	16	15.84%	196	58	29.59%	2.23 [1.33-3.74]	1.06E-02	0.507
TRPV5	101	2	1.98%	196	4	2.04%	1.03 [0.24-4.35]	0.972	1.000
TRPV6	101	2	1.98%	196	4	2.04%	1.03 [0.24-4.35]	0.972	1.000
PRSS3P2	101	21	20.79%	196	37	18.88%	0.89 [0.54-1.47]	0.693	0.951
TSC2	101	6	5.94%	196	5	2.55%	0.41 [0.15-1.15]	0.154	0.535
TSEN15	101	5	4.95%	196	20	10.20%	2.18 [0.93-5.10]	0.130	0.535
TSEN54	101	5	4.95%	196	12	6.12%	1.25 [0.51-3.08]	0.681	0.943
TSFM	101	0	0.00%	196	5	2.55%	2.2e+07 [0e+00-Inf]	0.992	1.000
TSG101	101	1	0.99%	196	4	2.04%	2.08 [0.33-13.25]	0.514	0.805
TSHZ2	101	11	10.89%	196	23	11.73%	1.09 [0.57-2.06]	0.829	1.000
TSKU	101	6	5.94%	196	16	8.16%	1.41 [0.62-3.18]	0.490	0.805
TSNARE1	101	17	16.83%	196	49	25.00%	1.65 [0.98-2.76]	0.111	0.511
TSNAX-DISC1	101	6	5.94%	196	19	9.69%	1.70 [0.77-3.78]	0.274	0.643
TSNAX	101	6	5.94%	196	19	9.69%	1.70 [0.77-3.78]	0.274	0.643
TSPAN10	101	2	1.98%	196	12	6.12%	3.23 [0.90-11.53]	0.130	0.535
TSPAN31	101	0	0.00%	196	5	2.55%	2.2e+07 [0e+00-Inf]	0.992	1.000
TSPAN8	101	3	2.97%	196	13	6.63%	2.32 [0.79-6.79]	0.197	0.570
TSPY1	101	0	0.00%	196	1	0.51%	3.2e+07 [0e+00-Inf]	0.997	1.000
TSPY2	101	0	0.00%	196	0	0.00%	1.00 [0e+00-Inf]	1.000	1.000
TSPY3	101	0	0.00%	196	0	0.00%	1.00 [0e+00-Inf]	1.000	1.000
TSPY4	101	0	0.00%	196	0	0.00%	1.00 [0e+00-Inf]	1.000	1.000
TSPYL5	101	15	14.85%	196	53	27.04%	2.12 [1.25-3.61]	1.95E-02	0.507
TSTA3	101	16	15.84%	196	51	26.02%	1.87 [1.11-3.15]	4.89E-02	0.507
TSTD1	101	6	5.94%	196	17	8.67%	1.50 [0.67-3.38]	0.407	0.774
TTC13	101	6	5.94%	196	19	9.69%	1.70 [0.77-3.78]	0.274	0.643
TTC14	101	4	3.96%	196	2	1.02%	0.25 [0.059-1.05]	0.113	0.511
TTC23	101	1	0.99%	196	9	4.59%	4.81 [0.84-27.58]	0.139	0.535
TTC24	101	6	5.94%	196	8	4.08%	0.67 [0.27-1.68]	0.476	0.805
EMC2	101	15	14.85%	196	50	25.51%	1.96 [1.15-3.35]	3.75E-02	0.507
TTLL6	101	2	1.98%	196	19	9.69%	5.31 [1.54-18.36]	2.67E-02	0.507
TTPAL	101	3	2.97%	196	4	2.04%	0.68 [0.19-2.43]	0.619	0.898
TTPA	101	15	14.85%	196	33	16.84%	1.16 [0.66-2.03]	0.660	0.935
TTTY10	101	0	0.00%	196	0	0.00%	1.00 [0e+00-Inf]	1.000	1.000
TTTY11	101	0	0.00%	196	0	0.00%	1.00 [0e+00-Inf]	1.000	1.000
TTTY12	101	0	0.00%	196	0	0.00%	1.00 [0e+00-Inf]	1.000	1.000
TTTY13	101	0	0.00%	196	0	0.00%	1.00 [0e+00-Inf]	1.000	1.000
TTTY15	101	0	0.00%	196	0	0.00%	1.00 [0e+00-Inf]	1.000	1.000
TTTY16	101	0	0.00%	196	0	0.00%	1.00 [0e+00-Inf]	1.000	1.000
TTTY17A	101	0	0.00%	196	0	0.00%	1.00 [0e+00-Inf]	1.000	1.000
TTTY17B	101	0	0.00%	196	0	0.00%	1.00 [0e+00-Inf]	1.000	1.000
TTTY18	101	0	0.00%	196	0	0.00%	1.00 [0e+00-Inf]	1.000	1.000
TTTY19	101	0	0.00%	196	0	0.00%	1.00 [0e+00-Inf]	1.000	1.000
TTTY1B	101	0	0.00%	196	0	0.00%	1.00 [0e+00-Inf]	1.000	1.000
TTTY20	101	0	0.00%	196	0	0.00%	1.00 [0e+00-Inf]	1.000	1.000
TTTY21	101	0	0.00%	196	0	0.00%	1.00 [0e+00-Inf]	1.000	1.000
TTTY22	101	0	0.00%	196	0	0.00%	1.00 [0e+00-Inf]	1.000	1.000
TTTY23	101	0	0.00%	196	0	0.00%	1.00 [0e+00-Inf]	1.000	1.000
TTTY2	101	0	0.00%	196	0	0.00%	1.00 [0e+00-Inf]	1.000	1.000
TTTY3B	101	0	0.00%	196	0	0.00%	1.00 [0e+00-Inf]	1.000	1.000
TTTY4C	101	0	0.00%	196	0	0.00%	1.00 [0e+00-Inf]	1.000	1.000
TTTY5	101	0	0.00%	196	0	0.00%	1.00 [0e+00-Inf]	1.000	1.000
TTTY6B	101	0	0.00%	196	0	0.00%	1.00 [0e+00-Inf]	1.000	1.000

Symbol	CINSARC Low			CINSARC High			CINSARC High vs. Low		
	N tested	N altered	Fq alteration	N tested	N altered	Fq alteration	OR [95%CI]	p-value*	q-value
TTTY6	101	0	0.00%	196	0	0.00%	1.00 [0e+00-Inf]	1.000	1.000
TTTY7	101	0	0.00%	196	0	0.00%	1.00 [0e+00-Inf]	1.000	1.000
TTTY8	101	0	0.00%	196	0	0.00%	1.00 [0e+00-Inf]	1.000	1.000
TTTY9B	101	0	0.00%	196	0	0.00%	1.00 [0e+00-Inf]	1.000	1.000
TTYH2	101	6	5.94%	196	15	7.65%	1.31 [0.58-2.98]	0.586	0.869
TUBB1	101	9	8.91%	196	15	7.65%	0.85 [0.41-1.75]	0.707	0.962
TUBD1	101	8	7.92%	196	34	17.35%	2.44 [1.23-4.82]	3.12E-02	0.507
TUFM	101	6	5.94%	196	3	1.53%	0.25 [0.076-0.80]	0.051	0.507
TUFT1	101	6	5.94%	196	12	6.12%	1.03 [0.44-2.41]	0.950	1.000
TXNDC11	101	5	4.95%	196	3	1.53%	0.30 [0.088-1.01]	0.103	0.507
TXNIP	101	5	4.95%	196	10	5.10%	1.03 [0.41-2.60]	0.955	1.000
U2AF2	101	2	1.98%	196	4	2.04%	1.03 [0.24-4.35]	0.972	1.000
UAP1	101	5	4.95%	196	15	7.65%	1.59 [0.66-3.81]	0.382	0.750
UBAP2L	101	7	6.93%	196	12	6.12%	0.88 [0.39-1.97]	0.788	0.996
UBE2C	101	5	4.95%	196	4	2.04%	0.40 [0.13-1.23]	0.179	0.535
UBE2I	101	6	5.94%	196	5	2.55%	0.41 [0.15-1.15]	0.154	0.535
UBE2MP1	101	2	1.98%	196	4	2.04%	1.03 [0.24-4.35]	0.972	1.000
UBE2N	101	0	0.00%	196	5	2.55%	2.2e+07 [0e+00-Inf]	0.992	1.000
UBE2O	101	3	2.97%	196	16	8.16%	2.90 [1.01-8.34]	0.097	0.507
UBE2Q1	101	7	6.93%	196	12	6.12%	0.88 [0.39-1.97]	0.788	0.996
UBE2Q2P1	101	1	0.99%	196	4	2.04%	2.08 [0.33-13.25]	0.514	0.805
UBE2S	101	1	0.99%	196	4	2.04%	2.08 [0.33-13.25]	0.514	0.805
UBE2T	101	7	6.93%	196	25	12.76%	1.96 [0.94-4.09]	0.131	0.535
UBE2V1	101	6	5.94%	196	14	7.14%	1.22 [0.53-2.79]	0.696	0.951
UBE2V2	101	14	13.86%	196	27	13.78%	0.99 [0.55-1.78]	0.984	1.000
UBE2W	101	17	16.83%	196	40	20.41%	1.27 [0.75-2.14]	0.459	0.805
UBE2Z	101	2	1.98%	196	18	9.18%	5.01 [1.44-17.35]	3.31E-02	0.507
UBFD1	101	5	4.95%	196	3	1.53%	0.30 [0.088-1.01]	0.103	0.507
UBL3	101	1	0.99%	196	4	2.04%	2.08 [0.33-13.25]	0.514	0.805
UBN1	101	5	4.95%	196	5	2.55%	0.50 [0.17-1.45]	0.286	0.650
UBQLN4	101	6	5.94%	196	9	4.59%	0.76 [0.31-1.86]	0.616	0.898
UBR5	101	16	15.84%	196	55	28.06%	2.07 [1.23-3.48]	2.09E-02	0.507
UBTFL1	101	1	0.99%	196	4	2.04%	2.08 [0.33-13.25]	0.514	0.805
UBXN2B	101	14	13.86%	196	33	16.84%	1.26 [0.71-2.22]	0.506	0.805
UBXN7	101	4	3.96%	196	4	2.04%	0.51 [0.16-1.65]	0.342	0.716
UBXN8	101	2	1.98%	196	6	3.06%	1.56 [0.40-6.08]	0.589	0.869
UCHL5	101	5	4.95%	196	17	8.67%	1.82 [0.77-4.32]	0.252	0.623
UCK2	101	4	3.96%	196	13	6.63%	1.72 [0.66-4.51]	0.353	0.722
UCKL1-AS1	101	6	5.94%	196	11	5.61%	0.94 [0.40-2.23]	0.908	1.000
UCKL1	101	6	5.94%	196	11	5.61%	0.94 [0.40-2.23]	0.908	1.000
UCP2	101	7	6.93%	196	12	6.12%	0.88 [0.39-1.97]	0.788	0.996
UCP3	101	7	6.93%	196	12	6.12%	0.88 [0.39-1.97]	0.788	0.996
UFC1	101	6	5.94%	196	16	8.16%	1.41 [0.62-3.18]	0.490	0.805
UHMK1	101	5	4.95%	196	15	7.65%	1.59 [0.66-3.81]	0.382	0.750
ULK2	101	4	3.96%	196	1	0.51%	0.12 [0.02-0.79]	0.064	0.507
UMOD	101	5	4.95%	196	3	1.53%	0.30 [0.088-1.01]	0.103	0.507
UNC119	101	3	2.97%	196	8	4.08%	1.39 [0.45-4.31]	0.632	0.909
UNC13D	101	4	3.96%	196	12	6.12%	1.58 [0.60-4.18]	0.438	0.798
UNC45A	101	1	0.99%	196	4	2.04%	2.08 [0.33-13.25]	0.514	0.805
UNC45B	101	1	0.99%	196	5	2.55%	2.62 [0.43-16.05]	0.383	0.750
UNC5D	101	6	5.94%	196	16	8.16%	1.41 [0.62-3.18]	0.490	0.805
UNC93B1	101	9	8.91%	196	21	10.71%	1.23 [0.62-2.44]	0.626	0.907
UNKL	101	6	5.94%	196	5	2.55%	0.41 [0.15-1.15]	0.154	0.535
UNK	101	4	3.96%	196	12	6.12%	1.58 [0.60-4.18]	0.438	0.798
UQCC1	101	4	3.96%	196	6	3.06%	0.77 [0.26-2.26]	0.685	0.943
UQCRB	101	16	15.84%	196	53	27.04%	1.97 [1.17-3.31]	3.23E-02	0.507
UQCRC2	101	5	4.95%	196	3	1.53%	0.30 [0.088-1.01]	0.103	0.507
URB2	101	6	5.94%	196	19	9.69%	1.70 [0.77-3.78]	0.274	0.643
USF1	101	6	5.94%	196	17	8.67%	1.50 [0.67-3.38]	0.407	0.774
USH1C	101	1	0.99%	196	4	2.04%	2.08 [0.33-13.25]	0.514	0.805
USH1G	101	5	4.95%	196	13	6.63%	1.36 [0.56-3.32]	0.566	0.852
USH2A	101	6	5.94%	196	19	9.69%	1.70 [0.77-3.78]	0.274	0.643
USO1	101	2	1.98%	196	5	2.55%	1.30 [0.32-5.21]	0.759	0.996
USP13	101	4	3.96%	196	2	1.02%	0.25 [0.059-1.05]	0.113	0.511
USP17L2	101	2	1.98%	196	4	2.04%	1.03 [0.24-4.35]	0.972	1.000
USP21	101	6	5.94%	196	16	8.16%	1.41 [0.62-3.18]	0.490	0.805
USP31	101	5	4.95%	196	2	1.02%	0.20 [0.049-0.80]	0.056	0.507
USP32	101	7	6.93%	196	33	16.84%	2.72 [1.33-5.57]	2.17E-02	0.507
USP35	101	8	7.92%	196	17	8.67%	1.10 [0.53-2.30]	0.825	1.000
USP36	101	3	2.97%	196	13	6.63%	2.32 [0.79-6.79]	0.197	0.570
USP54	101	0	0.00%	196	5	2.55%	2.2e+07 [0e+00-Inf]	0.992	1.000

Symbol	CINSARC Low			CINSARC High			CINSARC High vs. Low		
	N tested	N altered	Fq alteration	N tested	N altered	Fq alteration	OR [95%CI]	p-value*	q-value
USP7	101	6	5.94%	196	5	2.55%	0.41 [0.15-1.15]	0.154	0.535
USP9Y	101	0	0.00%	196	0	0.00%	1.00 [0e+00-Inf]	1.000	1.000
USPL1	101	1	0.99%	196	4	2.04%	2.08 [0.33-13.25]	0.514	0.805
UTP18	101	4	3.96%	196	15	7.65%	2.01 [0.78-5.19]	0.226	0.598
UTP23	101	16	15.84%	196	58	29.59%	2.23 [1.33-3.74]	1.06E-02	0.507
UTS2B	101	2	1.98%	196	3	1.53%	0.77 [0.17-3.50]	0.776	0.996
UTS2R	101	2	1.98%	196	13	6.63%	3.52 [0.99-12.47]	0.102	0.507
UTY	101	0	0.00%	196	0	0.00%	1.00 [0e+00-Inf]	1.000	1.000
UVRAG	101	5	4.95%	196	19	9.69%	2.06 [0.88-4.83]	0.163	0.535
VAMP4	101	5	4.95%	196	13	6.63%	1.36 [0.56-3.32]	0.566	0.852
VANGL2	101	5	4.95%	196	14	7.14%	1.48 [0.61-3.57]	0.467	0.805
VAPB	101	9	8.91%	196	17	8.67%	0.97 [0.48-1.97]	0.945	1.000
VASH2	101	6	5.94%	196	21	10.71%	1.90 [0.86-4.19]	0.181	0.535
VASN	101	5	4.95%	196	6	3.06%	0.61 [0.22-1.68]	0.418	0.779
VCL	101	0	0.00%	196	5	2.55%	2.2e+07 [0e+00-Inf]	0.992	1.000
VCPIP1	101	15	14.85%	196	38	19.39%	1.38 [0.80-2.38]	0.335	0.716
VCP	101	1	0.99%	196	4	2.04%	2.08 [0.33-13.25]	0.514	0.805
VCY	101	0	0.00%	196	0	0.00%	1.00 [0e+00-Inf]	1.000	1.000
VDAC2	101	2	1.98%	196	6	3.06%	1.56 [0.40-6.08]	0.589	0.869
VDAC3	101	14	13.86%	196	31	15.82%	1.17 [0.66-2.07]	0.656	0.931
VEZF1	101	6	5.94%	196	25	12.76%	2.31 [1.06-5.03]	0.076	0.507
VHLL	101	6	5.94%	196	9	4.59%	0.76 [0.31-1.86]	0.616	0.898
VKORC1	101	4	3.96%	196	6	3.06%	0.77 [0.26-2.26]	0.685	0.943
VN1R5	101	5	4.95%	196	19	9.69%	2.06 [0.88-4.83]	0.163	0.535
VPS13B	101	15	14.85%	196	55	28.06%	2.24 [1.32-3.80]	1.24E-02	0.507
VPS28	101	18	17.82%	196	49	25.00%	1.54 [0.93-2.55]	0.163	0.535
VPS37C	101	2	1.98%	196	3	1.53%	0.77 [0.17-3.50]	0.776	0.996
VPS41	101	0	0.00%	196	5	2.55%	2.2e+07 [0e+00-Inf]	0.992	1.000
VPS45	101	8	7.92%	196	14	7.14%	0.89 [0.42-1.91]	0.808	1.000
VPS4B	101	3	2.97%	196	2	1.02%	0.34 [0.074-1.53]	0.237	0.599
VPS72	101	7	6.93%	196	13	6.63%	0.95 [0.43-2.12]	0.923	1.000
VPS8	101	3	2.97%	196	2	1.02%	0.34 [0.074-1.53]	0.237	0.599
VSIG8	101	6	5.94%	196	10	5.10%	0.85 [0.36-2.04]	0.762	0.996
VSTM2L	101	5	4.95%	196	3	1.53%	0.30 [0.088-1.01]	0.103	0.507
VTN	101	3	2.97%	196	7	3.57%	1.21 [0.38-3.83]	0.786	0.996
VWA3A	101	6	5.94%	196	3	1.53%	0.25 [0.076-0.80]	0.051	0.507
VWA5B2	101	3	2.97%	196	2	1.02%	0.34 [0.074-1.53]	0.237	0.599
VWCE	101	2	1.98%	196	4	2.04%	1.03 [0.24-4.35]	0.972	1.000
WBP2	101	4	3.96%	196	13	6.63%	1.72 [0.66-4.51]	0.353	0.722
WDR24	101	6	5.94%	196	4	2.04%	0.33 [0.11-0.97]	0.092	0.507
WDR26	101	7	6.93%	196	17	8.67%	1.28 [0.59-2.75]	0.602	0.887
WDR45B	101	2	1.98%	196	15	7.65%	4.10 [1.17-14.39]	0.064	0.507
WDR53	101	4	3.96%	196	4	2.04%	0.51 [0.16-1.65]	0.342	0.716
WDR64	101	4	3.96%	196	20	10.20%	2.76 [1.09-6.95]	0.071	0.507
TBC1D31	101	14	13.86%	196	56	28.57%	2.49 [1.45-4.27]	5.58E-03	0.507
WDR73	101	1	0.99%	196	5	2.55%	2.62 [0.43-16.05]	0.383	0.750
WDR90	101	6	5.94%	196	4	2.04%	0.33 [0.11-0.97]	0.092	0.507
WDR93	101	1	0.99%	196	7	3.57%	3.70 [0.63-21.75]	0.224	0.596
WDYHV1	101	14	13.86%	196	58	29.59%	2.61 [1.52-4.48]	3.40E-03	0.507
WFDC10A	101	5	4.95%	196	3	1.53%	0.30 [0.088-1.01]	0.103	0.507
WFDC10B	101	5	4.95%	196	3	1.53%	0.30 [0.088-1.01]	0.103	0.507
WFDC11	101	5	4.95%	196	3	1.53%	0.30 [0.088-1.01]	0.103	0.507
WFDC12	101	5	4.95%	196	2	1.02%	0.20 [0.049-0.80]	0.056	0.507
WFDC13	101	5	4.95%	196	3	1.53%	0.30 [0.088-1.01]	0.103	0.507
WFDC2	101	5	4.95%	196	3	1.53%	0.30 [0.088-1.01]	0.103	0.507
WFDC3	101	5	4.95%	196	3	1.53%	0.30 [0.088-1.01]	0.103	0.507
WFDC5	101	5	4.95%	196	2	1.02%	0.20 [0.049-0.80]	0.056	0.507
WFDC6	101	5	4.95%	196	3	1.53%	0.30 [0.088-1.01]	0.103	0.507
WFDC8	101	5	4.95%	196	3	1.53%	0.30 [0.088-1.01]	0.103	0.507
WFDC9	101	5	4.95%	196	3	1.53%	0.30 [0.088-1.01]	0.103	0.507
WFIKKN1	101	6	5.94%	196	4	2.04%	0.33 [0.11-0.97]	0.092	0.507
WFIKKN2	101	4	3.96%	196	21	10.71%	2.91 [1.16-7.31]	0.056	0.507
WHAMM	101	1	0.99%	196	4	2.04%	2.08 [0.33-13.25]	0.514	0.805
WIF1	101	2	1.98%	196	5	2.55%	1.30 [0.32-5.21]	0.759	0.996
WIPF2	101	4	3.96%	196	8	4.08%	1.03 [0.37-2.88]	0.960	1.000
WIPF3	101	0	0.00%	196	6	3.06%	2.7e+07 [0e+00-Inf]	0.992	1.000
WIPI1	101	8	7.92%	196	27	13.78%	1.86 [0.93-3.72]	0.143	0.535
WNT11	101	6	5.94%	196	17	8.67%	1.50 [0.67-3.38]	0.407	0.774
WNT3A	101	6	5.94%	196	21	10.71%	1.90 [0.86-4.19]	0.181	0.535
WNT3	101	1	0.99%	196	5	2.55%	2.62 [0.43-16.05]	0.383	0.750
WNT9A	101	7	6.93%	196	21	10.71%	1.61 [0.76-3.40]	0.294	0.650

Symbol	CINSARC Low			CINSARC High			CINSARC High vs. Low		
	N tested	N altered	Fq alteration	N tested	N altered	Fq alteration	OR [95%CI]	p-value*	q-value
WNT9B	101	1	0.99%	196	5	2.55%	2.62 [0.43-16.05]	0.383	0.750
WRN	101	2	1.98%	196	6	3.06%	1.56 [0.40-6.08]	0.589	0.869
WSB1	101	3	2.97%	196	4	2.04%	0.68 [0.19-2.43]	0.619	0.898
WWP1	101	18	17.82%	196	47	23.98%	1.45 [0.87-2.42]	0.226	0.598
XCL1	101	5	4.95%	196	15	7.65%	1.59 [0.66-3.81]	0.382	0.750
XCL2	101	5	4.95%	196	13	6.63%	1.36 [0.56-3.32]	0.566	0.852
XKR4	101	13	12.87%	196	30	15.31%	1.22 [0.68-2.20]	0.573	0.860
XKR9	101	16	15.84%	196	39	19.90%	1.32 [0.77-2.26]	0.395	0.770
XKRY2	101	0	0.00%	196	0	0.00%	1.00 [0e+00-Inf]	1.000	1.000
XKRY	101	0	0.00%	196	0	0.00%	1.00 [0e+00-Inf]	1.000	1.000
XPO6	101	5	4.95%	196	3	1.53%	0.30 [0.088-1.01]	0.103	0.507
XPOT	101	1	0.99%	196	4	2.04%	2.08 [0.33-13.25]	0.514	0.805
XPR1	101	6	5.94%	196	18	9.18%	1.60 [0.72-3.57]	0.335	0.716
XRRA1	101	5	4.95%	196	15	7.65%	1.59 [0.66-3.81]	0.382	0.750
XYLT1	101	6	5.94%	196	4	2.04%	0.33 [0.11-0.97]	0.092	0.507
XYLT2	101	4	3.96%	196	19	9.69%	2.60 [1.03-6.59]	0.090	0.507
YEATS2	101	3	2.97%	196	2	1.02%	0.34 [0.074-1.53]	0.237	0.599
YEATS4	101	2	1.98%	196	18	9.18%	5.01 [1.44-17.35]	3.31E-02	0.507
YIF1A	101	2	1.98%	196	6	3.06%	1.56 [0.40-6.08]	0.589	0.869
YKT6	101	0	0.00%	196	5	2.55%	2.2e+07 [0e+00-Inf]	0.992	1.000
YOD1	101	6	5.94%	196	24	12.24%	2.21 [1.01-4.82]	0.095	0.507
YPEL2	101	8	7.92%	196	30	15.31%	2.10 [1.06-4.18]	0.076	0.507
YPEL3	101	5	4.95%	196	4	2.04%	0.40 [0.13-1.23]	0.179	0.535
YTHDF1	101	7	6.93%	196	12	6.12%	0.88 [0.39-1.97]	0.788	0.996
YTHDF3	101	15	14.85%	196	32	16.33%	1.12 [0.64-1.96]	0.742	0.996
YWHAB	101	4	3.96%	196	4	2.04%	0.51 [0.16-1.65]	0.342	0.716
YWHAZ	101	15	14.85%	196	57	29.08%	2.35 [1.39-3.99]	7.74E-03	0.507
YY1AP1	101	7	6.93%	196	9	4.59%	0.65 [0.27-1.52]	0.401	0.774
ZACN	101	4	3.96%	196	13	6.63%	1.72 [0.66-4.51]	0.353	0.722
ZBP1	101	9	8.91%	196	16	8.16%	0.91 [0.44-1.86]	0.826	1.000
ZBTB10	101	17	16.83%	196	50	25.51%	1.69 [1.01-2.83]	0.092	0.507
ZBTB37	101	5	4.95%	196	13	6.63%	1.36 [0.56-3.32]	0.566	0.852
ZBTB41	101	5	4.95%	196	21	10.71%	2.30 [0.99-5.36]	0.104	0.507
ZBTB46	101	7	6.93%	196	13	6.63%	0.95 [0.43-2.12]	0.923	1.000
ZBTB7B	101	8	7.92%	196	15	7.65%	0.96 [0.46-2.04]	0.935	1.000
ZC3H11A	101	7	6.93%	196	27	13.78%	2.15 [1.03-4.45]	0.085	0.507
ZC3H3	101	17	16.83%	196	51	26.02%	1.74 [1.04-2.90]	0.076	0.507
ZC3H7A	101	5	4.95%	196	3	1.53%	0.30 [0.088-1.01]	0.103	0.507
ZCCHC2	101	4	3.96%	196	2	1.02%	0.25 [0.059-1.05]	0.113	0.511
ZDHHC17	101	0	0.00%	196	7	3.57%	3.2e+07 [0e+00-Inf]	0.992	1.000
ZDHHC19	101	4	3.96%	196	3	1.53%	0.38 [0.11-1.35]	0.207	0.573
ZDHHC24	101	5	4.95%	196	11	5.61%	1.14 [0.46-2.84]	0.811	1.000
ZFAND1	101	18	17.82%	196	46	23.47%	1.41 [0.85-2.35]	0.263	0.643
ZFAT-AS1	101	16	15.84%	196	54	27.55%	2.02 [1.20-3.40]	2.60E-02	0.507
ZFAT	101	16	15.84%	196	54	27.55%	2.02 [1.20-3.40]	2.60E-02	0.507
ZFC3H1	101	1	0.99%	196	13	6.63%	7.10 [1.27-39.64]	0.061	0.507
ZFHX4	101	15	14.85%	196	46	23.47%	1.76 [1.03-3.01]	0.084	0.507
ZFP41	101	16	15.84%	196	51	26.02%	1.87 [1.11-3.15]	4.89E-02	0.507
ZFP64	101	9	8.91%	196	15	7.65%	0.85 [0.41-1.75]	0.707	0.962
ZFPM2	101	16	15.84%	196	52	26.53%	1.92 [1.14-3.23]	3.98E-02	0.507
ZFY	101	0	0.00%	196	0	0.00%	1.00 [0e+00-Inf]	1.000	1.000
ZG16B	101	6	5.94%	196	5	2.55%	0.41 [0.15-1.15]	0.154	0.535
ZG16	101	5	4.95%	196	3	1.53%	0.30 [0.088-1.01]	0.103	0.507
ZGPAT	101	7	6.93%	196	15	7.65%	1.11 [0.51-2.43]	0.822	1.000
ZHX1	101	14	13.86%	196	57	29.08%	2.55 [1.49-4.37]	4.36E-03	0.507
ZHX2	101	14	13.86%	196	56	28.57%	2.49 [1.45-4.27]	5.58E-03	0.507
ZHX3	101	3	2.97%	196	7	3.57%	1.21 [0.38-3.83]	0.786	0.996
ZKSCAN2	101	5	4.95%	196	4	2.04%	0.40 [0.13-1.23]	0.179	0.535
ZMAT3	101	4	3.96%	196	3	1.53%	0.38 [0.11-1.35]	0.207	0.573
ZMAT4	101	13	12.87%	196	32	16.33%	1.32 [0.74-2.37]	0.432	0.798
MSS51	101	0	0.00%	196	5	2.55%	2.2e+07 [0e+00-Inf]	0.992	1.000
ZMYND8	101	6	5.94%	196	8	4.08%	0.67 [0.27-1.68]	0.476	0.805
ZNF124	101	5	4.95%	196	19	9.69%	2.06 [0.88-4.83]	0.163	0.535
ZNF160	101	1	0.99%	196	4	2.04%	2.08 [0.33-13.25]	0.514	0.805
ZNF16	101	16	15.84%	196	48	24.49%	1.72 [1.02-2.91]	0.088	0.507
ZNF174	101	5	4.95%	196	4	2.04%	0.40 [0.13-1.23]	0.179	0.535
ZNF200	101	5	4.95%	196	4	2.04%	0.40 [0.13-1.23]	0.179	0.535
ZNF205	101	6	5.94%	196	4	2.04%	0.33 [0.11-0.97]	0.092	0.507
ZNF213	101	6	5.94%	196	4	2.04%	0.33 [0.11-0.97]	0.092	0.507
ZNF217	101	12	11.88%	196	26	13.27%	1.13 [0.61-2.09]	0.735	0.993
ZBTB18	101	5	4.95%	196	19	9.69%	2.06 [0.88-4.83]	0.163	0.535

Symbol	CINSARC Low			CINSARC High			CINSARC High vs. Low		
	N tested	N altered	Fq alteration	N tested	N altered	Fq alteration	OR [95%CI]	p-value*	q-value
ZNF250	101	16	15.84%	196	48	24.49%	1.72 [1.02-2.91]	0.088	0.507
ZNF251	101	17	16.83%	196	49	25.00%	1.65 [0.98-2.76]	0.111	0.511
ZNF252P	101	16	15.84%	196	47	23.98%	1.68 [0.99-2.84]	0.106	0.507
ZNF263	101	5	4.95%	196	4	2.04%	0.40 [0.13-1.23]	0.179	0.535
ZNF267	101	4	3.96%	196	4	2.04%	0.51 [0.16-1.65]	0.342	0.716
ZNF281	101	6	5.94%	196	24	12.24%	2.21 [1.01-4.82]	0.095	0.507
ZNF311	101	3	2.97%	196	5	2.55%	0.86 [0.25-2.89]	0.833	1.000
ZNF334	101	4	3.96%	196	6	3.06%	0.77 [0.26-2.26]	0.685	0.943
ZNF335	101	5	4.95%	196	4	2.04%	0.40 [0.13-1.23]	0.179	0.535
ZNF347	101	1	0.99%	196	4	2.04%	2.08 [0.33-13.25]	0.514	0.805
ZNF34	101	17	16.83%	196	49	25.00%	1.65 [0.98-2.76]	0.111	0.511
ZNF350	101	1	0.99%	196	4	2.04%	2.08 [0.33-13.25]	0.514	0.805
ZNF415	101	1	0.99%	196	4	2.04%	2.08 [0.33-13.25]	0.514	0.805
ZSCAN32	101	5	4.95%	196	4	2.04%	0.40 [0.13-1.23]	0.179	0.535
ZNF468	101	1	0.99%	196	4	2.04%	2.08 [0.33-13.25]	0.514	0.805
ZNF48	101	4	3.96%	196	5	2.55%	0.63 [0.21-1.95]	0.505	0.805
ZNF496	101	5	4.95%	196	19	9.69%	2.06 [0.88-4.83]	0.163	0.535
ZNF500	101	5	4.95%	196	5	2.55%	0.50 [0.17-1.45]	0.286	0.650
ZNF503	101	2	1.98%	196	5	2.55%	1.30 [0.32-5.21]	0.759	0.996
ZNF512B	101	6	5.94%	196	11	5.61%	0.94 [0.40-2.23]	0.908	1.000
ZNF517	101	16	15.84%	196	49	25.00%	1.77 [1.05-2.99]	0.073	0.507
ZNF524	101	2	1.98%	196	4	2.04%	1.03 [0.24-4.35]	0.972	1.000
ZNF532	101	1	0.99%	196	4	2.04%	2.08 [0.33-13.25]	0.514	0.805
ZNF572	101	16	15.84%	196	57	29.08%	2.18 [1.30-3.66]	1.33E-02	0.507
ZNF579	101	2	1.98%	196	4	2.04%	1.03 [0.24-4.35]	0.972	1.000
ZNF580	101	2	1.98%	196	4	2.04%	1.03 [0.24-4.35]	0.972	1.000
ZNF581	101	2	1.98%	196	4	2.04%	1.03 [0.24-4.35]	0.972	1.000
ZNF592	101	1	0.99%	196	6	3.06%	3.16 [0.53-18.88]	0.290	0.650
ZNF597	101	5	4.95%	196	4	2.04%	0.40 [0.13-1.23]	0.179	0.535
ZNF598	101	6	5.94%	196	5	2.55%	0.41 [0.15-1.15]	0.154	0.535
ZNF613	101	1	0.99%	196	4	2.04%	2.08 [0.33-13.25]	0.514	0.805
ZNF614	101	1	0.99%	196	4	2.04%	2.08 [0.33-13.25]	0.514	0.805
ZNF615	101	1	0.99%	196	4	2.04%	2.08 [0.33-13.25]	0.514	0.805
ZNF623	101	16	15.84%	196	51	26.02%	1.87 [1.11-3.15]	4.89E-02	0.507
ZNF628	101	2	1.98%	196	4	2.04%	1.03 [0.24-4.35]	0.972	1.000
ZNF629	101	4	3.96%	196	5	2.55%	0.63 [0.21-1.95]	0.505	0.805
ZNF639	101	4	3.96%	196	2	1.02%	0.25 [0.059-1.05]	0.113	0.511
ZNF646	101	4	3.96%	196	6	3.06%	0.77 [0.26-2.26]	0.685	0.943
ZNF648	101	5	4.95%	196	18	9.18%	1.94 [0.82-4.58]	0.203	0.570
ZNF649	101	1	0.99%	196	4	2.04%	2.08 [0.33-13.25]	0.514	0.805
ZNF652	101	5	4.95%	196	20	10.20%	2.18 [0.93-5.10]	0.130	0.535
ZNF665	101	1	0.99%	196	4	2.04%	2.08 [0.33-13.25]	0.514	0.805
ZNF668	101	4	3.96%	196	6	3.06%	0.77 [0.26-2.26]	0.685	0.943
ZNF669	101	5	4.95%	196	19	9.69%	2.06 [0.88-4.83]	0.163	0.535
ZNF670	101	5	4.95%	196	19	9.69%	2.06 [0.88-4.83]	0.163	0.535
ZNF672	101	6	5.94%	196	19	9.69%	1.70 [0.77-3.78]	0.274	0.643
ZNF678	101	6	5.94%	196	20	10.20%	1.80 [0.81-3.98]	0.224	0.596
ZNF687	101	6	5.94%	196	13	6.63%	1.12 [0.49-2.60]	0.817	1.000
ZNF688	101	4	3.96%	196	5	2.55%	0.63 [0.21-1.95]	0.505	0.805
ZNF689	101	4	3.96%	196	5	2.55%	0.63 [0.21-1.95]	0.505	0.805
ZNF692	101	5	4.95%	196	19	9.69%	2.06 [0.88-4.83]	0.163	0.535
ZNF695	101	5	4.95%	196	19	9.69%	2.06 [0.88-4.83]	0.163	0.535
ZNF696	101	17	16.83%	196	51	26.02%	1.74 [1.04-2.90]	0.076	0.507
ZNF703	101	11	10.89%	196	54	27.55%	3.11 [1.73-5.60]	1.48E-03	0.507
ZNF704	101	17	16.83%	196	49	25.00%	1.65 [0.98-2.76]	0.111	0.511
ZNF706	101	16	15.84%	196	57	29.08%	2.18 [1.30-3.66]	1.33E-02	0.507
ZNF707	101	17	16.83%	196	51	26.02%	1.74 [1.04-2.90]	0.076	0.507
ZNF710	101	1	0.99%	196	8	4.08%	4.26 [0.73-24.65]	0.175	0.535
ZNF720	101	4	3.96%	196	4	2.04%	0.51 [0.16-1.65]	0.342	0.716
ZNF747	101	4	3.96%	196	5	2.55%	0.63 [0.21-1.95]	0.505	0.805
ZNF750	101	2	1.98%	196	14	7.14%	3.81 [1.08-13.43]	0.081	0.507
ZNF75A	101	5	4.95%	196	4	2.04%	0.40 [0.13-1.23]	0.179	0.535
ZNF764	101	4	3.96%	196	5	2.55%	0.63 [0.21-1.95]	0.505	0.805
ZNF768	101	4	3.96%	196	5	2.55%	0.63 [0.21-1.95]	0.505	0.805
ZNF771	101	4	3.96%	196	5	2.55%	0.63 [0.21-1.95]	0.505	0.805
ZNF774	101	2	1.98%	196	6	3.06%	1.56 [0.40-6.08]	0.589	0.869
ZNF784	101	2	1.98%	196	4	2.04%	1.03 [0.24-4.35]	0.972	1.000
ZNF785	101	4	3.96%	196	5	2.55%	0.63 [0.21-1.95]	0.505	0.805
ZNF7	101	17	16.83%	196	49	25.00%	1.65 [0.98-2.76]	0.111	0.511
ZNF830	101	1	0.99%	196	5	2.55%	2.62 [0.43-16.05]	0.383	0.750
ZNF831	101	8	7.92%	196	15	7.65%	0.96 [0.46-2.04]	0.935	1.000

Symbol	CINSARC Low			CINSARC High			CINSARC High vs. Low		
	N tested	N altered	Fq alteration	N tested	N altered	Fq alteration	OR [95%CI]	p-value*	q-value
ZNF843	101	4	3.96%	196	4	2.04%	0.51 [0.16-1.65]	0.342	0.716
ZNFX1	101	7	6.93%	196	7	3.57%	0.50 [0.20-1.23]	0.203	0.570
ZNHIT3	101	2	1.98%	196	6	3.06%	1.56 [0.40-6.08]	0.589	0.869
ZNRF2	101	0	0.00%	196	7	3.57%	3.2e+07 [0e+00-Inf]	0.992	1.000
ZP2	101	5	4.95%	196	2	1.02%	0.20 [0.049-0.80]	0.056	0.507
ZP4	101	5	4.95%	196	19	9.69%	2.06 [0.88-4.83]	0.163	0.535
ZPBP2	101	6	5.94%	196	12	6.12%	1.03 [0.44-2.41]	0.950	1.000
ZSCAN10	101	6	5.94%	196	4	2.04%	0.33 [0.11-0.97]	0.092	0.507
ZSCAN2	101	1	0.99%	196	5	2.55%	2.62 [0.43-16.05]	0.383	0.750
ZSWIM1	101	5	4.95%	196	4	2.04%	0.40 [0.13-1.23]	0.179	0.535
ZSWIM3	101	5	4.95%	196	4	2.04%	0.40 [0.13-1.23]	0.179	0.535

Supplementary Table 6: Comparison of frequency of homozygous deletions among the 3,842 genes altered in at least 5/297 tested samples (TCGA) between the two CINSARC classes in Luminal B breast cancers.

Symbol	CINSARC Low			CINSARC High			CINSARC High vs. Low		
	N tested	N altered	Fq alteration	N tested	N altered	Fq alteration	OR [95%CI]	p-value*	q-value
RBFOX1	101	0	0.00%	196	0	0.00%	1.00 [0e+00-Inf]	1.000	1.000
AANAT	101	0	0.00%	196	0	0.00%	1.00 [0e+00-Inf]	1.000	1.000
AATF	101	0	0.00%	196	0	0.00%	1.00 [0e+00-Inf]	1.000	1.000
AATK	101	0	0.00%	196	0	0.00%	1.00 [0e+00-Inf]	1.000	1.000
ABAT	101	0	0.00%	196	0	0.00%	1.00 [0e+00-Inf]	1.000	1.000
ABCA10	101	0	0.00%	196	0	0.00%	1.00 [0e+00-Inf]	1.000	1.000
ABCA17P	101	0	0.00%	196	0	0.00%	1.00 [0e+00-Inf]	1.000	1.000
ABCA3	101	0	0.00%	196	0	0.00%	1.00 [0e+00-Inf]	1.000	1.000
ABCA5	101	0	0.00%	196	0	0.00%	1.00 [0e+00-Inf]	1.000	1.000
ABCA6	101	0	0.00%	196	0	0.00%	1.00 [0e+00-Inf]	1.000	1.000
ABCA8	101	0	0.00%	196	0	0.00%	1.00 [0e+00-Inf]	1.000	1.000
ABCA9	101	0	0.00%	196	0	0.00%	1.00 [0e+00-Inf]	1.000	1.000
ABCB10	101	0	0.00%	196	0	0.00%	1.00 [0e+00-Inf]	1.000	1.000
ABCB5	101	0	0.00%	196	0	0.00%	1.00 [0e+00-Inf]	1.000	1.000
ABCC12	101	0	0.00%	196	0	0.00%	1.00 [0e+00-Inf]	1.000	1.000
ABCC1	101	0	0.00%	196	0	0.00%	1.00 [0e+00-Inf]	1.000	1.000
ABCC3	101	0	0.00%	196	0	0.00%	1.00 [0e+00-Inf]	1.000	1.000
ABCC5	101	0	0.00%	196	0	0.00%	1.00 [0e+00-Inf]	1.000	1.000
ABCC6P1	101	0	0.00%	196	0	0.00%	1.00 [0e+00-Inf]	1.000	1.000
ABCC6P2	101	0	0.00%	196	0	0.00%	1.00 [0e+00-Inf]	1.000	1.000
ABCC6	101	0	0.00%	196	0	0.00%	1.00 [0e+00-Inf]	1.000	1.000
ABCC8	101	0	0.00%	196	0	0.00%	1.00 [0e+00-Inf]	1.000	1.000
ABCF3	101	0	0.00%	196	0	0.00%	1.00 [0e+00-Inf]	1.000	1.000
ABHD15	101	0	0.00%	196	0	0.00%	1.00 [0e+00-Inf]	1.000	1.000
ABHD2	101	0	0.00%	196	0	0.00%	1.00 [0e+00-Inf]	1.000	1.000
ABI3	101	0	0.00%	196	0	0.00%	1.00 [0e+00-Inf]	1.000	1.000
ABL2	101	0	0.00%	196	0	0.00%	1.00 [0e+00-Inf]	1.000	1.000
ABRA	101	0	0.00%	196	0	0.00%	1.00 [0e+00-Inf]	1.000	1.000
ACACA	101	0	0.00%	196	0	0.00%	1.00 [0e+00-Inf]	1.000	1.000
ACAN	101	0	0.00%	196	0	0.00%	1.00 [0e+00-Inf]	1.000	1.000
ACAP2	101	0	0.00%	196	0	0.00%	1.00 [0e+00-Inf]	1.000	1.000
ACBD3	101	0	0.00%	196	0	0.00%	1.00 [0e+00-Inf]	1.000	1.000
ACBD6	101	0	0.00%	196	0	0.00%	1.00 [0e+00-Inf]	1.000	1.000
ACER3	101	0	0.00%	196	0	0.00%	1.00 [0e+00-Inf]	1.000	1.000
ACE	101	0	0.00%	196	0	0.00%	1.00 [0e+00-Inf]	1.000	1.000
ACIN1	101	0	0.00%	196	0	0.00%	1.00 [0e+00-Inf]	1.000	1.000
ACOT1	101	3	2.97%	196	4	2.04%	0.68 [0.19-2.43]	0.619	1.000
ACOT8	101	0	0.00%	196	0	0.00%	1.00 [0e+00-Inf]	1.000	1.000
ACOX1	101	0	0.00%	196	0	0.00%	1.00 [0e+00-Inf]	1.000	1.000
ACP6	101	0	0.00%	196	0	0.00%	1.00 [0e+00-Inf]	1.000	1.000
ACSF2	101	0	0.00%	196	0	0.00%	1.00 [0e+00-Inf]	1.000	1.000
ACSM1	101	0	0.00%	196	0	0.00%	1.00 [0e+00-Inf]	1.000	1.000
ACSM2A	101	0	0.00%	196	0	0.00%	1.00 [0e+00-Inf]	1.000	1.000
ACSM2B	101	0	0.00%	196	0	0.00%	1.00 [0e+00-Inf]	1.000	1.000
ACSM3	101	0	0.00%	196	0	0.00%	1.00 [0e+00-Inf]	1.000	1.000
ACSM5	101	0	0.00%	196	0	0.00%	1.00 [0e+00-Inf]	1.000	1.000
ACSS2	101	0	0.00%	196	0	0.00%	1.00 [0e+00-Inf]	1.000	1.000
ACSS3	101	0	0.00%	196	0	0.00%	1.00 [0e+00-Inf]	1.000	1.000
ACTA1	101	0	0.00%	196	0	0.00%	1.00 [0e+00-Inf]	1.000	1.000
ACTG1	101	0	0.00%	196	0	0.00%	1.00 [0e+00-Inf]	1.000	1.000
ACTL6A	101	0	0.00%	196	0	0.00%	1.00 [0e+00-Inf]	1.000	1.000
ACTN2	101	0	0.00%	196	0	0.00%	1.00 [0e+00-Inf]	1.000	1.000
ACTN3	101	0	0.00%	196	0	0.00%	1.00 [0e+00-Inf]	1.000	1.000
ACTR5	101	0	0.00%	196	0	0.00%	1.00 [0e+00-Inf]	1.000	1.000
ACY3	101	0	0.00%	196	0	0.00%	1.00 [0e+00-Inf]	1.000	1.000
ADAM15	101	0	0.00%	196	0	0.00%	1.00 [0e+00-Inf]	1.000	1.000
ADAM18	101	0	0.00%	196	0	0.00%	1.00 [0e+00-Inf]	1.000	1.000
ADAM2	101	0	0.00%	196	0	0.00%	1.00 [0e+00-Inf]	1.000	1.000
ADAM30	101	0	0.00%	196	0	0.00%	1.00 [0e+00-Inf]	1.000	1.000
ADAM32	101	0	0.00%	196	0	0.00%	1.00 [0e+00-Inf]	1.000	1.000
ADAM3A	101	11	10.89%	196	11	5.61%	0.49 [0.23-1.01]	0.106	1.000
ADAM5	101	0	0.00%	196	0	0.00%	1.00 [0e+00-Inf]	1.000	1.000
ADAM9	101	0	0.00%	196	0	0.00%	1.00 [0e+00-Inf]	1.000	1.000
ADAMTS4	101	0	0.00%	196	0	0.00%	1.00 [0e+00-Inf]	1.000	1.000
ADAMTSL4	101	0	0.00%	196	0	0.00%	1.00 [0e+00-Inf]	1.000	1.000
ADAP2	101	0	0.00%	196	0	0.00%	1.00 [0e+00-Inf]	1.000	1.000
ADAR	101	0	0.00%	196	0	0.00%	1.00 [0e+00-Inf]	1.000	1.000
ADA	101	0	0.00%	196	0	0.00%	1.00 [0e+00-Inf]	1.000	1.000

Symbol	CINSARC Low			CINSARC High			CINSARC High vs. Low		
	N tested	N altered	Fq alteration	N tested	N altered	Fq alteration	OR [95%CI]	p-value*	q-value
ADCK2	101	0	0.00%	196	0	0.00%	1.00 [0e+00-Inf]	1.000	1.000
ADCK5	101	0	0.00%	196	0	0.00%	1.00 [0e+00-Inf]	1.000	1.000
ADCY10	101	0	0.00%	196	0	0.00%	1.00 [0e+00-Inf]	1.000	1.000
ADCY8	101	0	0.00%	196	0	0.00%	1.00 [0e+00-Inf]	1.000	1.000
ADCY9	101	0	0.00%	196	0	0.00%	1.00 [0e+00-Inf]	1.000	1.000
ADCYAP1R1	101	0	0.00%	196	0	0.00%	1.00 [0e+00-Inf]	1.000	1.000
ADHFE1	101	0	0.00%	196	0	0.00%	1.00 [0e+00-Inf]	1.000	1.000
ADIG	101	0	0.00%	196	0	0.00%	1.00 [0e+00-Inf]	1.000	1.000
ADIPOR1	101	0	0.00%	196	0	0.00%	1.00 [0e+00-Inf]	1.000	1.000
ADK	101	0	0.00%	196	0	0.00%	1.00 [0e+00-Inf]	1.000	1.000
ADNP	101	0	0.00%	196	0	0.00%	1.00 [0e+00-Inf]	1.000	1.000
ADORA1	101	0	0.00%	196	0	0.00%	1.00 [0e+00-Inf]	1.000	1.000
ADRB3	101	0	0.00%	196	0	0.00%	1.00 [0e+00-Inf]	1.000	1.000
ADRM1	101	0	0.00%	196	0	0.00%	1.00 [0e+00-Inf]	1.000	1.000
ADSS	101	0	0.00%	196	0	0.00%	1.00 [0e+00-Inf]	1.000	1.000
AEN	101	0	0.00%	196	0	0.00%	1.00 [0e+00-Inf]	1.000	1.000
AFMID	101	0	0.00%	196	0	0.00%	1.00 [0e+00-Inf]	1.000	1.000
AFM	101	0	0.00%	196	0	0.00%	1.00 [0e+00-Inf]	1.000	1.000
AFP	101	0	0.00%	196	0	0.00%	1.00 [0e+00-Inf]	1.000	1.000
AGAP2	101	0	0.00%	196	0	0.00%	1.00 [0e+00-Inf]	1.000	1.000
AGAP5	101	0	0.00%	196	0	0.00%	1.00 [0e+00-Inf]	1.000	1.000
AGBL1	101	0	0.00%	196	0	0.00%	1.00 [0e+00-Inf]	1.000	1.000
AGT	101	0	0.00%	196	0	0.00%	1.00 [0e+00-Inf]	1.000	1.000
AHCTF1	101	0	0.00%	196	0	0.00%	1.00 [0e+00-Inf]	1.000	1.000
AHSP	101	0	0.00%	196	0	0.00%	1.00 [0e+00-Inf]	1.000	1.000
AIDA	101	0	0.00%	196	0	0.00%	1.00 [0e+00-Inf]	1.000	1.000
AIM2	101	0	0.00%	196	0	0.00%	1.00 [0e+00-Inf]	1.000	1.000
AIP	101	0	0.00%	196	0	0.00%	1.00 [0e+00-Inf]	1.000	1.000
AKAP10	101	0	0.00%	196	0	0.00%	1.00 [0e+00-Inf]	1.000	1.000
AKAP13	101	0	0.00%	196	0	0.00%	1.00 [0e+00-Inf]	1.000	1.000
AKAP1	101	0	0.00%	196	0	0.00%	1.00 [0e+00-Inf]	1.000	1.000
AKAP6	101	0	0.00%	196	0	0.00%	1.00 [0e+00-Inf]	1.000	1.000
AKT3	101	0	0.00%	196	0	0.00%	1.00 [0e+00-Inf]	1.000	1.000
ALB	101	0	0.00%	196	0	0.00%	1.00 [0e+00-Inf]	1.000	1.000
ALDH3A1	101	0	0.00%	196	0	0.00%	1.00 [0e+00-Inf]	1.000	1.000
ALDH3A2	101	0	0.00%	196	0	0.00%	1.00 [0e+00-Inf]	1.000	1.000
ALDH3B1	101	0	0.00%	196	0	0.00%	1.00 [0e+00-Inf]	1.000	1.000
ALDH3B2	101	0	0.00%	196	0	0.00%	1.00 [0e+00-Inf]	1.000	1.000
ALDH9A1	101	0	0.00%	196	0	0.00%	1.00 [0e+00-Inf]	1.000	1.000
ALDOA	101	0	0.00%	196	0	0.00%	1.00 [0e+00-Inf]	1.000	1.000
ALDOC	101	0	0.00%	196	0	0.00%	1.00 [0e+00-Inf]	1.000	1.000
ALG1	101	0	0.00%	196	0	0.00%	1.00 [0e+00-Inf]	1.000	1.000
ALG3	101	0	0.00%	196	0	0.00%	1.00 [0e+00-Inf]	1.000	1.000
ALG8	101	0	0.00%	196	0	0.00%	1.00 [0e+00-Inf]	1.000	1.000
ALPK3	101	0	0.00%	196	0	0.00%	1.00 [0e+00-Inf]	1.000	1.000
CCL18	101	0	0.00%	196	0	0.00%	1.00 [0e+00-Inf]	1.000	1.000
AMDHD2	101	0	0.00%	196	0	0.00%	1.00 [0e+00-Inf]	1.000	1.000
AMELY	101	64	63.37%	196	115	58.67%	0.82 [0.54-1.24]	0.434	1.000
AMZ2P1	101	0	0.00%	196	0	0.00%	1.00 [0e+00-Inf]	1.000	1.000
AMZ2	101	0	0.00%	196	0	0.00%	1.00 [0e+00-Inf]	1.000	1.000
ANAPC11	101	0	0.00%	196	0	0.00%	1.00 [0e+00-Inf]	1.000	1.000
ANGEL2	101	0	0.00%	196	0	0.00%	1.00 [0e+00-Inf]	1.000	1.000
ANGPT1	101	0	0.00%	196	0	0.00%	1.00 [0e+00-Inf]	1.000	1.000
ANGPTL1	101	0	0.00%	196	0	0.00%	1.00 [0e+00-Inf]	1.000	1.000
ANK1	101	0	0.00%	196	0	0.00%	1.00 [0e+00-Inf]	1.000	1.000
ANK3	101	0	0.00%	196	0	0.00%	1.00 [0e+00-Inf]	1.000	1.000
ANKFN1	101	0	0.00%	196	0	0.00%	1.00 [0e+00-Inf]	1.000	1.000
ANKRD13B	101	0	0.00%	196	0	0.00%	1.00 [0e+00-Inf]	1.000	1.000
ANKRD13D	101	0	0.00%	196	0	0.00%	1.00 [0e+00-Inf]	1.000	1.000
ANKRD17	101	0	0.00%	196	0	0.00%	1.00 [0e+00-Inf]	1.000	1.000
ANKRD34A	101	0	0.00%	196	0	0.00%	1.00 [0e+00-Inf]	1.000	1.000
ANKRD35	101	0	0.00%	196	0	0.00%	1.00 [0e+00-Inf]	1.000	1.000
ANKRD36BP1	101	0	0.00%	196	0	0.00%	1.00 [0e+00-Inf]	1.000	1.000
ANKRD40	101	0	0.00%	196	0	0.00%	1.00 [0e+00-Inf]	1.000	1.000
ANKRD45	101	0	0.00%	196	0	0.00%	1.00 [0e+00-Inf]	1.000	1.000
ANKRD46	101	0	0.00%	196	0	0.00%	1.00 [0e+00-Inf]	1.000	1.000
ANKS3	101	0	0.00%	196	0	0.00%	1.00 [0e+00-Inf]	1.000	1.000
ANKS4B	101	0	0.00%	196	0	0.00%	1.00 [0e+00-Inf]	1.000	1.000
ANO1	101	0	0.00%	196	0	0.00%	1.00 [0e+00-Inf]	1.000	1.000
ANO5	101	0	0.00%	196	0	0.00%	1.00 [0e+00-Inf]	1.000	1.000
ANP32E	101	0	0.00%	196	0	0.00%	1.00 [0e+00-Inf]	1.000	1.000

Symbol	CINSARC Low			CINSARC High			CINSARC High vs. Low		
	N tested	N altered	Fq alteration	N tested	N altered	Fq alteration	OR [95%CI]	p-value*	q-value
ANPEP	101	0	0.00%	196	0	0.00%	1.00 [0e+00-Inf]	1.000	1.000
ANTXRL	101	0	0.00%	196	0	0.00%	1.00 [0e+00-Inf]	1.000	1.000
ANXA13	101	0	0.00%	196	0	0.00%	1.00 [0e+00-Inf]	1.000	1.000
ANXA7	101	0	0.00%	196	0	0.00%	1.00 [0e+00-Inf]	1.000	1.000
ANXA9	101	0	0.00%	196	0	0.00%	1.00 [0e+00-Inf]	1.000	1.000
AP2B1	101	0	0.00%	196	0	0.00%	1.00 [0e+00-Inf]	1.000	1.000
AP2M1	101	0	0.00%	196	0	0.00%	1.00 [0e+00-Inf]	1.000	1.000
AP3B2	101	0	0.00%	196	0	0.00%	1.00 [0e+00-Inf]	1.000	1.000
AP3M1	101	0	0.00%	196	0	0.00%	1.00 [0e+00-Inf]	1.000	1.000
AP3M2	101	0	0.00%	196	0	0.00%	1.00 [0e+00-Inf]	1.000	1.000
AP3S2	101	0	0.00%	196	0	0.00%	1.00 [0e+00-Inf]	1.000	1.000
AP4S1	101	0	0.00%	196	0	0.00%	1.00 [0e+00-Inf]	1.000	1.000
APCDD1L	101	0	0.00%	196	0	0.00%	1.00 [0e+00-Inf]	1.000	1.000
APCS	101	0	0.00%	196	0	0.00%	1.00 [0e+00-Inf]	1.000	1.000
APH1A	101	0	0.00%	196	0	0.00%	1.00 [0e+00-Inf]	1.000	1.000
APOA2	101	0	0.00%	196	0	0.00%	1.00 [0e+00-Inf]	1.000	1.000
APOBR	101	0	0.00%	196	0	0.00%	1.00 [0e+00-Inf]	1.000	1.000
APOBEC4	101	0	0.00%	196	0	0.00%	1.00 [0e+00-Inf]	1.000	1.000
APOD	101	0	0.00%	196	0	0.00%	1.00 [0e+00-Inf]	1.000	1.000
APOH	101	0	0.00%	196	0	0.00%	1.00 [0e+00-Inf]	1.000	1.000
APPBP2	101	0	0.00%	196	0	0.00%	1.00 [0e+00-Inf]	1.000	1.000
AQP10	101	0	0.00%	196	0	0.00%	1.00 [0e+00-Inf]	1.000	1.000
AQP11	101	0	0.00%	196	0	0.00%	1.00 [0e+00-Inf]	1.000	1.000
AQP1	101	0	0.00%	196	0	0.00%	1.00 [0e+00-Inf]	1.000	1.000
AQP8	101	0	0.00%	196	0	0.00%	1.00 [0e+00-Inf]	1.000	1.000
ARAP1	101	0	0.00%	196	0	0.00%	1.00 [0e+00-Inf]	1.000	1.000
ARC	101	0	0.00%	196	0	0.00%	1.00 [0e+00-Inf]	1.000	1.000
AREG	101	0	0.00%	196	0	0.00%	1.00 [0e+00-Inf]	1.000	1.000
ARF1	101	0	0.00%	196	0	0.00%	1.00 [0e+00-Inf]	1.000	1.000
ARFGAP1	101	0	0.00%	196	0	0.00%	1.00 [0e+00-Inf]	1.000	1.000
ARFGEF1	101	0	0.00%	196	0	0.00%	1.00 [0e+00-Inf]	1.000	1.000
ARFGEF2	101	0	0.00%	196	0	0.00%	1.00 [0e+00-Inf]	1.000	1.000
ARFRP1	101	0	0.00%	196	0	0.00%	1.00 [0e+00-Inf]	1.000	1.000
ARHGAP17	101	0	0.00%	196	0	0.00%	1.00 [0e+00-Inf]	1.000	1.000
ARHGAP23	101	0	0.00%	196	0	0.00%	1.00 [0e+00-Inf]	1.000	1.000
ARHGAP30	101	0	0.00%	196	0	0.00%	1.00 [0e+00-Inf]	1.000	1.000
ARHGAP39	101	0	0.00%	196	0	0.00%	1.00 [0e+00-Inf]	1.000	1.000
ARHGAP5	101	0	0.00%	196	0	0.00%	1.00 [0e+00-Inf]	1.000	1.000
ARHGDIA	101	0	0.00%	196	0	0.00%	1.00 [0e+00-Inf]	1.000	1.000
ARHGDIG	101	0	0.00%	196	0	0.00%	1.00 [0e+00-Inf]	1.000	1.000
ARHGEF11	101	0	0.00%	196	0	0.00%	1.00 [0e+00-Inf]	1.000	1.000
ARHGEF17	101	0	0.00%	196	0	0.00%	1.00 [0e+00-Inf]	1.000	1.000
ARHGEF2	101	0	0.00%	196	0	0.00%	1.00 [0e+00-Inf]	1.000	1.000
ARID3C	101	0	0.00%	196	0	0.00%	1.00 [0e+00-Inf]	1.000	1.000
ARID4B	101	0	0.00%	196	0	0.00%	1.00 [0e+00-Inf]	1.000	1.000
ARL16	101	0	0.00%	196	0	0.00%	1.00 [0e+00-Inf]	1.000	1.000
ARL17B	101	0	0.00%	196	0	0.00%	1.00 [0e+00-Inf]	1.000	1.000
ARL5C	101	0	0.00%	196	0	0.00%	1.00 [0e+00-Inf]	1.000	1.000
ARL6IP1	101	0	0.00%	196	0	0.00%	1.00 [0e+00-Inf]	1.000	1.000
ARL8A	101	0	0.00%	196	0	0.00%	1.00 [0e+00-Inf]	1.000	1.000
ARMC1	101	0	0.00%	196	0	0.00%	1.00 [0e+00-Inf]	1.000	1.000
ARMC2	101	0	0.00%	196	1	0.51%	3.2e+07 [0e+00-Inf]	0.997	1.000
ARMC5	101	0	0.00%	196	0	0.00%	1.00 [0e+00-Inf]	1.000	1.000
ARMC7	101	0	0.00%	196	0	0.00%	1.00 [0e+00-Inf]	1.000	1.000
ARNT2	101	0	0.00%	196	0	0.00%	1.00 [0e+00-Inf]	1.000	1.000
ARNT	101	0	0.00%	196	0	0.00%	1.00 [0e+00-Inf]	1.000	1.000
ARPC5	101	0	0.00%	196	0	0.00%	1.00 [0e+00-Inf]	1.000	1.000
ARRB1	101	0	0.00%	196	0	0.00%	1.00 [0e+00-Inf]	1.000	1.000
ARRDC4	101	0	0.00%	196	0	0.00%	1.00 [0e+00-Inf]	1.000	1.000
ARSG	101	0	0.00%	196	0	0.00%	1.00 [0e+00-Inf]	1.000	1.000
ART3	101	0	0.00%	196	0	0.00%	1.00 [0e+00-Inf]	1.000	1.000
ARV1	101	0	0.00%	196	0	0.00%	1.00 [0e+00-Inf]	1.000	1.000
ASAP1-IT1	101	0	0.00%	196	0	0.00%	1.00 [0e+00-Inf]	1.000	1.000
ASAP1	101	0	0.00%	196	0	0.00%	1.00 [0e+00-Inf]	1.000	1.000
ASB7	101	0	0.00%	196	0	0.00%	1.00 [0e+00-Inf]	1.000	1.000
ASH1L	101	0	0.00%	196	0	0.00%	1.00 [0e+00-Inf]	1.000	1.000
ASH2L	101	0	0.00%	196	0	0.00%	1.00 [0e+00-Inf]	1.000	1.000
ASPHD1	101	0	0.00%	196	0	0.00%	1.00 [0e+00-Inf]	1.000	1.000
ASPH	101	0	0.00%	196	0	0.00%	1.00 [0e+00-Inf]	1.000	1.000
ASPM	101	0	0.00%	196	0	0.00%	1.00 [0e+00-Inf]	1.000	1.000
ASPSCR1	101	0	0.00%	196	0	0.00%	1.00 [0e+00-Inf]	1.000	1.000

Symbol	CINSARC Low			CINSARC High			CINSARC High vs. Low		
	N tested	N altered	Fq alteration	N tested	N altered	Fq alteration	OR [95%CI]	p-value*	q-value
ASTN1	101	0	0.00%	196	0	0.00%	1.00 [0e+00-Inf]	1.000	1.000
ATAD2	101	0	0.00%	196	0	0.00%	1.00 [0e+00-Inf]	1.000	1.000
ATAD5	101	0	0.00%	196	0	0.00%	1.00 [0e+00-Inf]	1.000	1.000
ATF3	101	0	0.00%	196	0	0.00%	1.00 [0e+00-Inf]	1.000	1.000
ATF6	101	0	0.00%	196	0	0.00%	1.00 [0e+00-Inf]	1.000	1.000
ATF7IP2	101	0	0.00%	196	0	0.00%	1.00 [0e+00-Inf]	1.000	1.000
ATG16L2	101	0	0.00%	196	0	0.00%	1.00 [0e+00-Inf]	1.000	1.000
ATG5	101	0	0.00%	196	0	0.00%	1.00 [0e+00-Inf]	1.000	1.000
ATP11B	101	0	0.00%	196	0	0.00%	1.00 [0e+00-Inf]	1.000	1.000
ATP13A3	101	0	0.00%	196	0	0.00%	1.00 [0e+00-Inf]	1.000	1.000
ATP13A4	101	0	0.00%	196	0	0.00%	1.00 [0e+00-Inf]	1.000	1.000
ATP13A5	101	0	0.00%	196	0	0.00%	1.00 [0e+00-Inf]	1.000	1.000
ATP1A2	101	0	0.00%	196	0	0.00%	1.00 [0e+00-Inf]	1.000	1.000
ATP1A4	101	0	0.00%	196	0	0.00%	1.00 [0e+00-Inf]	1.000	1.000
ATP1B1	101	0	0.00%	196	0	0.00%	1.00 [0e+00-Inf]	1.000	1.000
ATP2A1	101	0	0.00%	196	0	0.00%	1.00 [0e+00-Inf]	1.000	1.000
ATP2B4	101	0	0.00%	196	0	0.00%	1.00 [0e+00-Inf]	1.000	1.000
ATP6V0C	101	0	0.00%	196	0	0.00%	1.00 [0e+00-Inf]	1.000	1.000
ATP6V0D2	101	0	0.00%	196	0	0.00%	1.00 [0e+00-Inf]	1.000	1.000
ATP6V1C1	101	0	0.00%	196	0	0.00%	1.00 [0e+00-Inf]	1.000	1.000
ATP6V1G3	101	0	0.00%	196	0	0.00%	1.00 [0e+00-Inf]	1.000	1.000
ATP6V1H	101	0	0.00%	196	0	0.00%	1.00 [0e+00-Inf]	1.000	1.000
ATP8B2	101	0	0.00%	196	0	0.00%	1.00 [0e+00-Inf]	1.000	1.000
ATP9A	101	0	0.00%	196	0	0.00%	1.00 [0e+00-Inf]	1.000	1.000
ATXN2L	101	0	0.00%	196	0	0.00%	1.00 [0e+00-Inf]	1.000	1.000
ATXN7L3B	101	0	0.00%	196	0	0.00%	1.00 [0e+00-Inf]	1.000	1.000
AURKA	101	0	0.00%	196	0	0.00%	1.00 [0e+00-Inf]	1.000	1.000
AVIL	101	0	0.00%	196	0	0.00%	1.00 [0e+00-Inf]	1.000	1.000
AVL9	101	0	0.00%	196	0	0.00%	1.00 [0e+00-Inf]	1.000	1.000
AVPR1A	101	0	0.00%	196	0	0.00%	1.00 [0e+00-Inf]	1.000	1.000
AVPR1B	101	0	0.00%	196	0	0.00%	1.00 [0e+00-Inf]	1.000	1.000
AXIN1	101	0	0.00%	196	0	0.00%	1.00 [0e+00-Inf]	1.000	1.000
AXIN2	101	0	0.00%	196	0	0.00%	1.00 [0e+00-Inf]	1.000	1.000
AZIN1	101	0	0.00%	196	0	0.00%	1.00 [0e+00-Inf]	1.000	1.000
B3GALNT2	101	0	0.00%	196	0	0.00%	1.00 [0e+00-Inf]	1.000	1.000
B3GALT2	101	0	0.00%	196	0	0.00%	1.00 [0e+00-Inf]	1.000	1.000
B3GNT5	101	0	0.00%	196	0	0.00%	1.00 [0e+00-Inf]	1.000	1.000
B3GNT6	101	0	0.00%	196	0	0.00%	1.00 [0e+00-Inf]	1.000	1.000
B3GNTL1	101	0	0.00%	196	0	0.00%	1.00 [0e+00-Inf]	1.000	1.000
B4GALNT2	101	0	0.00%	196	0	0.00%	1.00 [0e+00-Inf]	1.000	1.000
B4GALT3	101	0	0.00%	196	0	0.00%	1.00 [0e+00-Inf]	1.000	1.000
B4GALT5	101	0	0.00%	196	0	0.00%	1.00 [0e+00-Inf]	1.000	1.000
B9D1	101	0	0.00%	196	0	0.00%	1.00 [0e+00-Inf]	1.000	1.000
BAALC	101	0	0.00%	196	0	0.00%	1.00 [0e+00-Inf]	1.000	1.000
BAG4	101	0	0.00%	196	0	0.00%	1.00 [0e+00-Inf]	1.000	1.000
BAHCC1	101	0	0.00%	196	0	0.00%	1.00 [0e+00-Inf]	1.000	1.000
BAIAP2	101	0	0.00%	196	0	0.00%	1.00 [0e+00-Inf]	1.000	1.000
BAIAP3	101	0	0.00%	196	0	0.00%	1.00 [0e+00-Inf]	1.000	1.000
PRRC2C	101	0	0.00%	196	0	0.00%	1.00 [0e+00-Inf]	1.000	1.000
BATF3	101	0	0.00%	196	0	0.00%	1.00 [0e+00-Inf]	1.000	1.000
BAZ1A	101	0	0.00%	196	0	0.00%	1.00 [0e+00-Inf]	1.000	1.000
BBS10	101	0	0.00%	196	0	0.00%	1.00 [0e+00-Inf]	1.000	1.000
BBS1	101	0	0.00%	196	0	0.00%	1.00 [0e+00-Inf]	1.000	1.000
BBS9	101	0	0.00%	196	0	0.00%	1.00 [0e+00-Inf]	1.000	1.000
BCAN	101	0	0.00%	196	0	0.00%	1.00 [0e+00-Inf]	1.000	1.000
BCAR4	101	0	0.00%	196	0	0.00%	1.00 [0e+00-Inf]	1.000	1.000
BCAS1	101	0	0.00%	196	0	0.00%	1.00 [0e+00-Inf]	1.000	1.000
BCAS3	101	0	0.00%	196	0	0.00%	1.00 [0e+00-Inf]	1.000	1.000
BCAS4	101	0	0.00%	196	0	0.00%	1.00 [0e+00-Inf]	1.000	1.000
BCKDK	101	0	0.00%	196	0	0.00%	1.00 [0e+00-Inf]	1.000	1.000
BCL2	101	0	0.00%	196	0	0.00%	1.00 [0e+00-Inf]	1.000	1.000
BCL7C	101	0	0.00%	196	0	0.00%	1.00 [0e+00-Inf]	1.000	1.000
NBEAP1	101	0	0.00%	196	0	0.00%	1.00 [0e+00-Inf]	1.000	1.000
BCL9	101	0	0.00%	196	0	0.00%	1.00 [0e+00-Inf]	1.000	1.000
BCORP1	101	64	63.37%	196	116	59.18%	0.84 [0.55-1.27]	0.485	1.000
BDH1	101	0	0.00%	196	0	0.00%	1.00 [0e+00-Inf]	1.000	1.000
BEND3	101	0	0.00%	196	0	0.00%	1.00 [0e+00-Inf]	1.000	1.000
BEST3	101	0	0.00%	196	0	0.00%	1.00 [0e+00-Inf]	1.000	1.000
LINC00293	101	0	0.00%	196	0	0.00%	1.00 [0e+00-Inf]	1.000	1.000
BFAR	101	0	0.00%	196	0	0.00%	1.00 [0e+00-Inf]	1.000	1.000
BGLAP	101	0	0.00%	196	0	0.00%	1.00 [0e+00-Inf]	1.000	1.000

Symbol	CINSARC Low			CINSARC High			CINSARC High vs. Low		
	N tested	N altered	Fq alteration	N tested	N altered	Fq alteration	OR [95%CI]	p-value*	q-value
BHLHE22	101	0	0.00%	196	0	0.00%	1.00 [0e+00-Inf]	1.000	1.000
BHLHE23	101	0	0.00%	196	0	0.00%	1.00 [0e+00-Inf]	1.000	1.000
BIRC5	101	0	0.00%	196	0	0.00%	1.00 [0e+00-Inf]	1.000	1.000
BIRC7	101	0	0.00%	196	0	0.00%	1.00 [0e+00-Inf]	1.000	1.000
BLCAP	101	0	0.00%	196	0	0.00%	1.00 [0e+00-Inf]	1.000	1.000
BLMH	101	0	0.00%	196	0	0.00%	1.00 [0e+00-Inf]	1.000	1.000
BLM	101	0	0.00%	196	0	0.00%	1.00 [0e+00-Inf]	1.000	1.000
BLZF1	101	0	0.00%	196	0	0.00%	1.00 [0e+00-Inf]	1.000	1.000
BMP7	101	0	0.00%	196	0	0.00%	1.00 [0e+00-Inf]	1.000	1.000
BMPER	101	0	0.00%	196	0	0.00%	1.00 [0e+00-Inf]	1.000	1.000
BMS1P4	101	0	0.00%	196	0	0.00%	1.00 [0e+00-Inf]	1.000	1.000
BNIPL	101	0	0.00%	196	0	0.00%	1.00 [0e+00-Inf]	1.000	1.000
BOD1	101	0	0.00%	196	0	0.00%	1.00 [0e+00-Inf]	1.000	1.000
BOLA1	101	0	0.00%	196	0	0.00%	1.00 [0e+00-Inf]	1.000	1.000
BOLA2	101	0	0.00%	196	0	0.00%	1.00 [0e+00-Inf]	1.000	1.000
BOP1	101	0	0.00%	196	0	0.00%	1.00 [0e+00-Inf]	1.000	1.000
BPI	101	0	0.00%	196	0	0.00%	1.00 [0e+00-Inf]	1.000	1.000
BPNT1	101	0	0.00%	196	0	0.00%	1.00 [0e+00-Inf]	1.000	1.000
BPTF	101	0	0.00%	196	0	0.00%	1.00 [0e+00-Inf]	1.000	1.000
BPY2	101	65	64.36%	196	116	59.18%	0.80 [0.53-1.22]	0.387	1.000
BRAF	101	0	0.00%	196	0	0.00%	1.00 [0e+00-Inf]	1.000	1.000
BREA2	101	0	0.00%	196	0	0.00%	1.00 [0e+00-Inf]	1.000	1.000
BRF2	101	0	0.00%	196	0	0.00%	1.00 [0e+00-Inf]	1.000	1.000
BRIP1	101	0	0.00%	196	0	0.00%	1.00 [0e+00-Inf]	1.000	1.000
BRMS1L	101	0	0.00%	196	0	0.00%	1.00 [0e+00-Inf]	1.000	1.000
BRMS1	101	0	0.00%	196	0	0.00%	1.00 [0e+00-Inf]	1.000	1.000
MPC2	101	0	0.00%	196	0	0.00%	1.00 [0e+00-Inf]	1.000	1.000
BRSK1	101	0	0.00%	196	0	0.00%	1.00 [0e+00-Inf]	1.000	1.000
SLX4	101	0	0.00%	196	0	0.00%	1.00 [0e+00-Inf]	1.000	1.000
BTBD17	101	0	0.00%	196	0	0.00%	1.00 [0e+00-Inf]	1.000	1.000
BTC	101	0	0.00%	196	0	0.00%	1.00 [0e+00-Inf]	1.000	1.000
BTG1	101	0	0.00%	196	0	0.00%	1.00 [0e+00-Inf]	1.000	1.000
BTG2	101	0	0.00%	196	0	0.00%	1.00 [0e+00-Inf]	1.000	1.000
BTNL3	101	0	0.00%	196	0	0.00%	1.00 [0e+00-Inf]	1.000	1.000
BVES	101	0	0.00%	196	0	0.00%	1.00 [0e+00-Inf]	1.000	1.000
C10orf105	101	0	0.00%	196	0	0.00%	1.00 [0e+00-Inf]	1.000	1.000
ZNF503-AS2	101	0	0.00%	196	0	0.00%	1.00 [0e+00-Inf]	1.000	1.000
C10orf55	101	0	0.00%	196	0	0.00%	1.00 [0e+00-Inf]	1.000	1.000
C11orf24	101	0	0.00%	196	0	0.00%	1.00 [0e+00-Inf]	1.000	1.000
KIAA1549L	101	0	0.00%	196	0	0.00%	1.00 [0e+00-Inf]	1.000	1.000
ANAPC15	101	0	0.00%	196	0	0.00%	1.00 [0e+00-Inf]	1.000	1.000
C11orf58	101	0	0.00%	196	0	0.00%	1.00 [0e+00-Inf]	1.000	1.000
LAMTOR1	101	0	0.00%	196	0	0.00%	1.00 [0e+00-Inf]	1.000	1.000
PPP1R32	101	0	0.00%	196	0	0.00%	1.00 [0e+00-Inf]	1.000	1.000
AAMDC	101	0	0.00%	196	0	0.00%	1.00 [0e+00-Inf]	1.000	1.000
C11orf80	101	0	0.00%	196	0	0.00%	1.00 [0e+00-Inf]	1.000	1.000
C11orf86	101	0	0.00%	196	0	0.00%	1.00 [0e+00-Inf]	1.000	1.000
C12orf29	101	0	0.00%	196	0	0.00%	1.00 [0e+00-Inf]	1.000	1.000
C12orf50	101	0	0.00%	196	0	0.00%	1.00 [0e+00-Inf]	1.000	1.000
C12orf66	101	0	0.00%	196	0	0.00%	1.00 [0e+00-Inf]	1.000	1.000
C12orf74	101	0	0.00%	196	0	0.00%	1.00 [0e+00-Inf]	1.000	1.000
DTD2	101	0	0.00%	196	0	0.00%	1.00 [0e+00-Inf]	1.000	1.000
ARHGAP5-AS1	101	0	0.00%	196	0	0.00%	1.00 [0e+00-Inf]	1.000	1.000
SPTSSA	101	0	0.00%	196	0	0.00%	1.00 [0e+00-Inf]	1.000	1.000
IGBP1P1	101	0	0.00%	196	0	0.00%	1.00 [0e+00-Inf]	1.000	1.000
C15orf32	101	0	0.00%	196	0	0.00%	1.00 [0e+00-Inf]	1.000	1.000
HEXA-AS1	101	0	0.00%	196	0	0.00%	1.00 [0e+00-Inf]	1.000	1.000
TICRR	101	0	0.00%	196	0	0.00%	1.00 [0e+00-Inf]	1.000	1.000
DNM1P46	101	0	0.00%	196	0	0.00%	1.00 [0e+00-Inf]	1.000	1.000
GDPGP1	101	0	0.00%	196	0	0.00%	1.00 [0e+00-Inf]	1.000	1.000
TSR3	101	0	0.00%	196	0	0.00%	1.00 [0e+00-Inf]	1.000	1.000
PAGR1	101	0	0.00%	196	0	0.00%	1.00 [0e+00-Inf]	1.000	1.000
C16orf54	101	0	0.00%	196	0	0.00%	1.00 [0e+00-Inf]	1.000	1.000
C16orf58	101	0	0.00%	196	0	0.00%	1.00 [0e+00-Inf]	1.000	1.000
CDIP1	101	0	0.00%	196	0	0.00%	1.00 [0e+00-Inf]	1.000	1.000
FOPNL	101	0	0.00%	196	0	0.00%	1.00 [0e+00-Inf]	1.000	1.000
METTL22	101	0	0.00%	196	0	0.00%	1.00 [0e+00-Inf]	1.000	1.000
C16orf71	101	0	0.00%	196	0	0.00%	1.00 [0e+00-Inf]	1.000	1.000
C16orf72	101	0	0.00%	196	0	0.00%	1.00 [0e+00-Inf]	1.000	1.000
MEIOB	101	0	0.00%	196	0	0.00%	1.00 [0e+00-Inf]	1.000	1.000
RMI2	101	0	0.00%	196	0	0.00%	1.00 [0e+00-Inf]	1.000	1.000

Symbol	CINSARC Low			CINSARC High			CINSARC High vs. Low		
	N tested	N altered	Fq alteration	N tested	N altered	Fq alteration	OR [95%CI]	p-value*	q-value
BRICD5	101	0	0.00%	196	0	0.00%	1.00 [0e+00-Inf]	1.000	1.000
C16orf82	101	0	0.00%	196	0	0.00%	1.00 [0e+00-Inf]	1.000	1.000
KNOP1	101	0	0.00%	196	0	0.00%	1.00 [0e+00-Inf]	1.000	1.000
C16orf89	101	0	0.00%	196	0	0.00%	1.00 [0e+00-Inf]	1.000	1.000
C16orf90	101	0	0.00%	196	0	0.00%	1.00 [0e+00-Inf]	1.000	1.000
C16orf91	101	0	0.00%	196	0	0.00%	1.00 [0e+00-Inf]	1.000	1.000
C16orf92	101	0	0.00%	196	0	0.00%	1.00 [0e+00-Inf]	1.000	1.000
OGFOD3	101	0	0.00%	196	0	0.00%	1.00 [0e+00-Inf]	1.000	1.000
TEN1	101	0	0.00%	196	0	0.00%	1.00 [0e+00-Inf]	1.000	1.000
LYRM9	101	0	0.00%	196	0	0.00%	1.00 [0e+00-Inf]	1.000	1.000
HID1	101	0	0.00%	196	0	0.00%	1.00 [0e+00-Inf]	1.000	1.000
MIEN1	101	0	0.00%	196	0	0.00%	1.00 [0e+00-Inf]	1.000	1.000
TEFM	101	0	0.00%	196	0	0.00%	1.00 [0e+00-Inf]	1.000	1.000
sept-04	101	0	0.00%	196	0	0.00%	1.00 [0e+00-Inf]	1.000	1.000
C17orf50	101	0	0.00%	196	0	0.00%	1.00 [0e+00-Inf]	1.000	1.000
LINC00469	101	0	0.00%	196	0	0.00%	1.00 [0e+00-Inf]	1.000	1.000
LINC00482	101	0	0.00%	196	0	0.00%	1.00 [0e+00-Inf]	1.000	1.000
EFCAB13	101	0	0.00%	196	0	0.00%	1.00 [0e+00-Inf]	1.000	1.000
C17orf58	101	0	0.00%	196	0	0.00%	1.00 [0e+00-Inf]	1.000	1.000
MILR1	101	0	0.00%	196	0	0.00%	1.00 [0e+00-Inf]	1.000	1.000
FAM222B	101	0	0.00%	196	0	0.00%	1.00 [0e+00-Inf]	1.000	1.000
C17orf64	101	0	0.00%	196	0	0.00%	1.00 [0e+00-Inf]	1.000	1.000
C17orf67	101	0	0.00%	196	0	0.00%	1.00 [0e+00-Inf]	1.000	1.000
SMG8	101	0	0.00%	196	0	0.00%	1.00 [0e+00-Inf]	1.000	1.000
C17orf77	101	0	0.00%	196	0	0.00%	1.00 [0e+00-Inf]	1.000	1.000
C17orf78	101	0	0.00%	196	0	0.00%	1.00 [0e+00-Inf]	1.000	1.000
C17orf80	101	0	0.00%	196	0	0.00%	1.00 [0e+00-Inf]	1.000	1.000
C17orf82	101	0	0.00%	196	0	0.00%	1.00 [0e+00-Inf]	1.000	1.000
OXLD1	101	0	0.00%	196	0	0.00%	1.00 [0e+00-Inf]	1.000	1.000
PRAC2	101	0	0.00%	196	0	0.00%	1.00 [0e+00-Inf]	1.000	1.000
METTL23	101	0	0.00%	196	0	0.00%	1.00 [0e+00-Inf]	1.000	1.000
C17orf98	101	0	0.00%	196	0	0.00%	1.00 [0e+00-Inf]	1.000	1.000
C17orf99	101	0	0.00%	196	0	0.00%	1.00 [0e+00-Inf]	1.000	1.000
LINC00305	101	0	0.00%	196	0	0.00%	1.00 [0e+00-Inf]	1.000	1.000
DYNAP	101	0	0.00%	196	0	0.00%	1.00 [0e+00-Inf]	1.000	1.000
C18orf54	101	0	0.00%	196	0	0.00%	1.00 [0e+00-Inf]	1.000	1.000
C1QTNF1	101	0	0.00%	196	0	0.00%	1.00 [0e+00-Inf]	1.000	1.000
C1QTNF8	101	0	0.00%	196	0	0.00%	1.00 [0e+00-Inf]	1.000	1.000
C1orf100	101	0	0.00%	196	0	0.00%	1.00 [0e+00-Inf]	1.000	1.000
RUSC1-AS1	101	0	0.00%	196	0	0.00%	1.00 [0e+00-Inf]	1.000	1.000
C1orf105	101	0	0.00%	196	0	0.00%	1.00 [0e+00-Inf]	1.000	1.000
C1orf112	101	0	0.00%	196	0	0.00%	1.00 [0e+00-Inf]	1.000	1.000
CCDC181	101	0	0.00%	196	0	0.00%	1.00 [0e+00-Inf]	1.000	1.000
C1orf115	101	0	0.00%	196	0	0.00%	1.00 [0e+00-Inf]	1.000	1.000
C1orf116	101	0	0.00%	196	0	0.00%	1.00 [0e+00-Inf]	1.000	1.000
SPRTN	101	0	0.00%	196	0	0.00%	1.00 [0e+00-Inf]	1.000	1.000
AXDND1	101	0	0.00%	196	0	0.00%	1.00 [0e+00-Inf]	1.000	1.000
MROH9	101	0	0.00%	196	0	0.00%	1.00 [0e+00-Inf]	1.000	1.000
C1orf131	101	0	0.00%	196	0	0.00%	1.00 [0e+00-Inf]	1.000	1.000
SERTAD4-AS1	101	0	0.00%	196	0	0.00%	1.00 [0e+00-Inf]	1.000	1.000
SHCBP1L	101	0	0.00%	196	0	0.00%	1.00 [0e+00-Inf]	1.000	1.000
GCSAML	101	0	0.00%	196	0	0.00%	1.00 [0e+00-Inf]	1.000	1.000
PFN1P2	101	0	0.00%	196	0	0.00%	1.00 [0e+00-Inf]	1.000	1.000
METTL18	101	0	0.00%	196	0	0.00%	1.00 [0e+00-Inf]	1.000	1.000
LINC00303	101	0	0.00%	196	0	0.00%	1.00 [0e+00-Inf]	1.000	1.000
TSACC	101	0	0.00%	196	0	0.00%	1.00 [0e+00-Inf]	1.000	1.000
C1orf189	101	0	0.00%	196	0	0.00%	1.00 [0e+00-Inf]	1.000	1.000
C1orf198	101	0	0.00%	196	0	0.00%	1.00 [0e+00-Inf]	1.000	1.000
C1orf21	101	0	0.00%	196	0	0.00%	1.00 [0e+00-Inf]	1.000	1.000
C1orf220	101	0	0.00%	196	0	0.00%	1.00 [0e+00-Inf]	1.000	1.000
C1orf226	101	0	0.00%	196	0	0.00%	1.00 [0e+00-Inf]	1.000	1.000
C1orf229	101	0	0.00%	196	0	0.00%	1.00 [0e+00-Inf]	1.000	1.000
RIIAD1	101	0	0.00%	196	0	0.00%	1.00 [0e+00-Inf]	1.000	1.000
TRMT1L	101	0	0.00%	196	0	0.00%	1.00 [0e+00-Inf]	1.000	1.000
SWT1	101	0	0.00%	196	0	0.00%	1.00 [0e+00-Inf]	1.000	1.000
COA6	101	0	0.00%	196	0	0.00%	1.00 [0e+00-Inf]	1.000	1.000
C1orf35	101	0	0.00%	196	0	0.00%	1.00 [0e+00-Inf]	1.000	1.000
C1orf43	101	0	0.00%	196	0	0.00%	1.00 [0e+00-Inf]	1.000	1.000
TEX35	101	0	0.00%	196	0	0.00%	1.00 [0e+00-Inf]	1.000	1.000
C1orf53	101	0	0.00%	196	0	0.00%	1.00 [0e+00-Inf]	1.000	1.000
C1orf54	101	0	0.00%	196	0	0.00%	1.00 [0e+00-Inf]	1.000	1.000

Symbol	CINSARC Low			CINSARC High			CINSARC High vs. Low		
	N tested	N altered	Fq alteration	N tested	N altered	Fq alteration	OR [95%CI]	p-value*	q-value
SDE2	101	0	0.00%	196	0	0.00%	1.00 [0e+00-Inf]	1.000	1.000
C1orf56	101	0	0.00%	196	0	0.00%	1.00 [0e+00-Inf]	1.000	1.000
NTPCR	101	0	0.00%	196	0	0.00%	1.00 [0e+00-Inf]	1.000	1.000
BROX	101	0	0.00%	196	0	0.00%	1.00 [0e+00-Inf]	1.000	1.000
C1orf61	101	0	0.00%	196	0	0.00%	1.00 [0e+00-Inf]	1.000	1.000
RRNAD1	101	0	0.00%	196	0	0.00%	1.00 [0e+00-Inf]	1.000	1.000
C1orf68	101	0	0.00%	196	0	0.00%	1.00 [0e+00-Inf]	1.000	1.000
IBA57	101	0	0.00%	196	0	0.00%	1.00 [0e+00-Inf]	1.000	1.000
C1orf74	101	0	0.00%	196	0	0.00%	1.00 [0e+00-Inf]	1.000	1.000
CHTOP	101	0	0.00%	196	0	0.00%	1.00 [0e+00-Inf]	1.000	1.000
LRRC71	101	0	0.00%	196	0	0.00%	1.00 [0e+00-Inf]	1.000	1.000
CCSAP	101	0	0.00%	196	0	0.00%	1.00 [0e+00-Inf]	1.000	1.000
LINC00467	101	0	0.00%	196	0	0.00%	1.00 [0e+00-Inf]	1.000	1.000
SUCO	101	0	0.00%	196	0	0.00%	1.00 [0e+00-Inf]	1.000	1.000
FAM209A	101	0	0.00%	196	0	0.00%	1.00 [0e+00-Inf]	1.000	1.000
FAM209B	101	0	0.00%	196	0	0.00%	1.00 [0e+00-Inf]	1.000	1.000
FAM210B	101	0	0.00%	196	0	0.00%	1.00 [0e+00-Inf]	1.000	1.000
OSER1	101	0	0.00%	196	0	0.00%	1.00 [0e+00-Inf]	1.000	1.000
SOGA1	101	0	0.00%	196	0	0.00%	1.00 [0e+00-Inf]	1.000	1.000
TLDC2	101	0	0.00%	196	0	0.00%	1.00 [0e+00-Inf]	1.000	1.000
GID8	101	0	0.00%	196	0	0.00%	1.00 [0e+00-Inf]	1.000	1.000
OCSTAMP	101	0	0.00%	196	0	0.00%	1.00 [0e+00-Inf]	1.000	1.000
MROH8	101	0	0.00%	196	0	0.00%	1.00 [0e+00-Inf]	1.000	1.000
ABHD16B	101	0	0.00%	196	0	0.00%	1.00 [0e+00-Inf]	1.000	1.000
RBBP8NL	101	0	0.00%	196	0	0.00%	1.00 [0e+00-Inf]	1.000	1.000
CNBD2	101	0	0.00%	196	0	0.00%	1.00 [0e+00-Inf]	1.000	1.000
SPATA25	101	0	0.00%	196	0	0.00%	1.00 [0e+00-Inf]	1.000	1.000
C20orf173	101	0	0.00%	196	0	0.00%	1.00 [0e+00-Inf]	1.000	1.000
FAM217B	101	0	0.00%	196	0	0.00%	1.00 [0e+00-Inf]	1.000	1.000
C20orf197	101	0	0.00%	196	0	0.00%	1.00 [0e+00-Inf]	1.000	1.000
ZFAS1	101	0	0.00%	196	0	0.00%	1.00 [0e+00-Inf]	1.000	1.000
MRGBP	101	0	0.00%	196	0	0.00%	1.00 [0e+00-Inf]	1.000	1.000
AAR2	101	0	0.00%	196	0	0.00%	1.00 [0e+00-Inf]	1.000	1.000
C20orf85	101	0	0.00%	196	0	0.00%	1.00 [0e+00-Inf]	1.000	1.000
C2CD3	101	0	0.00%	196	0	0.00%	1.00 [0e+00-Inf]	1.000	1.000
C2CD4D	101	0	0.00%	196	0	0.00%	1.00 [0e+00-Inf]	1.000	1.000
ERGIC3	101	0	0.00%	196	0	0.00%	1.00 [0e+00-Inf]	1.000	1.000
XXYLT1	101	0	0.00%	196	0	0.00%	1.00 [0e+00-Inf]	1.000	1.000
CEP19	101	0	0.00%	196	0	0.00%	1.00 [0e+00-Inf]	1.000	1.000
MB21D2	101	0	0.00%	196	0	0.00%	1.00 [0e+00-Inf]	1.000	1.000
C3orf70	101	0	0.00%	196	0	0.00%	1.00 [0e+00-Inf]	1.000	1.000
C4BPA	101	0	0.00%	196	0	0.00%	1.00 [0e+00-Inf]	1.000	1.000
C4BPB	101	0	0.00%	196	0	0.00%	1.00 [0e+00-Inf]	1.000	1.000
GFOD1	101	0	0.00%	196	0	0.00%	1.00 [0e+00-Inf]	1.000	1.000
C6orf203	101	0	0.00%	196	0	0.00%	1.00 [0e+00-Inf]	1.000	1.000
CCDC170	101	0	0.00%	196	0	0.00%	1.00 [0e+00-Inf]	1.000	1.000
PPP1R17	101	0	0.00%	196	0	0.00%	1.00 [0e+00-Inf]	1.000	1.000
MALSU1	101	0	0.00%	196	0	0.00%	1.00 [0e+00-Inf]	1.000	1.000
C7orf31	101	0	0.00%	196	0	0.00%	1.00 [0e+00-Inf]	1.000	1.000
MTURN	101	0	0.00%	196	0	0.00%	1.00 [0e+00-Inf]	1.000	1.000
FAM221A	101	0	0.00%	196	0	0.00%	1.00 [0e+00-Inf]	1.000	1.000
C7orf71	101	0	0.00%	196	0	0.00%	1.00 [0e+00-Inf]	1.000	1.000
TMEM249	101	0	0.00%	196	0	0.00%	1.00 [0e+00-Inf]	1.000	1.000
C8orf31	101	0	0.00%	196	0	0.00%	1.00 [0e+00-Inf]	1.000	1.000
C8orf33	101	0	0.00%	196	0	0.00%	1.00 [0e+00-Inf]	1.000	1.000
C8orf34	101	0	0.00%	196	0	0.00%	1.00 [0e+00-Inf]	1.000	1.000
C8orf37	101	0	0.00%	196	0	0.00%	1.00 [0e+00-Inf]	1.000	1.000
NDUFAF6	101	0	0.00%	196	0	0.00%	1.00 [0e+00-Inf]	1.000	1.000
RBM12B-AS1	101	0	0.00%	196	0	0.00%	1.00 [0e+00-Inf]	1.000	1.000
SMIM19	101	0	0.00%	196	0	0.00%	1.00 [0e+00-Inf]	1.000	1.000
TTI2	101	0	0.00%	196	0	0.00%	1.00 [0e+00-Inf]	1.000	1.000
C8orf44	101	0	0.00%	196	0	0.00%	1.00 [0e+00-Inf]	1.000	1.000
MCMDC2	101	0	0.00%	196	0	0.00%	1.00 [0e+00-Inf]	1.000	1.000
RHPN1-AS1	101	0	0.00%	196	0	0.00%	1.00 [0e+00-Inf]	1.000	1.000
THEM6	101	0	0.00%	196	0	0.00%	1.00 [0e+00-Inf]	1.000	1.000
LINC00588	101	0	0.00%	196	0	0.00%	1.00 [0e+00-Inf]	1.000	1.000
MROH6	101	0	0.00%	196	0	0.00%	1.00 [0e+00-Inf]	1.000	1.000
C8orf76	101	0	0.00%	196	0	0.00%	1.00 [0e+00-Inf]	1.000	1.000
ZNF252P-AS1	101	0	0.00%	196	0	0.00%	1.00 [0e+00-Inf]	1.000	1.000
TRIQK	101	0	0.00%	196	0	0.00%	1.00 [0e+00-Inf]	1.000	1.000
SBSPON	101	0	0.00%	196	0	0.00%	1.00 [0e+00-Inf]	1.000	1.000

Symbol	CINSARC Low			CINSARC High			CINSARC High vs. Low		
	N tested	N altered	Fq alteration	N tested	N altered	Fq alteration	OR [95%CI]	p-value*	q-value
AARD	101	0	0.00%	196	0	0.00%	1.00 [0e+00-Inf]	1.000	1.000
C8orf86	101	0	0.00%	196	0	0.00%	1.00 [0e+00-Inf]	1.000	1.000
C9orf131	101	0	0.00%	196	0	0.00%	1.00 [0e+00-Inf]	1.000	1.000
FAM205A	101	0	0.00%	196	0	0.00%	1.00 [0e+00-Inf]	1.000	1.000
RPP25L	101	0	0.00%	196	0	0.00%	1.00 [0e+00-Inf]	1.000	1.000
CA10	101	0	0.00%	196	0	0.00%	1.00 [0e+00-Inf]	1.000	1.000
CA13	101	0	0.00%	196	0	0.00%	1.00 [0e+00-Inf]	1.000	1.000
CA14	101	0	0.00%	196	0	0.00%	1.00 [0e+00-Inf]	1.000	1.000
CA1	101	0	0.00%	196	0	0.00%	1.00 [0e+00-Inf]	1.000	1.000
CA2	101	0	0.00%	196	0	0.00%	1.00 [0e+00-Inf]	1.000	1.000
CA3	101	0	0.00%	196	0	0.00%	1.00 [0e+00-Inf]	1.000	1.000
CA4	101	0	0.00%	196	0	0.00%	1.00 [0e+00-Inf]	1.000	1.000
CA8	101	0	0.00%	196	0	0.00%	1.00 [0e+00-Inf]	1.000	1.000
CABLES2	101	0	0.00%	196	0	0.00%	1.00 [0e+00-Inf]	1.000	1.000
CABP2	101	0	0.00%	196	0	0.00%	1.00 [0e+00-Inf]	1.000	1.000
CABP4	101	0	0.00%	196	0	0.00%	1.00 [0e+00-Inf]	1.000	1.000
CACNA1E	101	0	0.00%	196	0	0.00%	1.00 [0e+00-Inf]	1.000	1.000
CACNA1G	101	0	0.00%	196	0	0.00%	1.00 [0e+00-Inf]	1.000	1.000
CACNA1H	101	0	0.00%	196	0	0.00%	1.00 [0e+00-Inf]	1.000	1.000
CACNA1S	101	0	0.00%	196	0	0.00%	1.00 [0e+00-Inf]	1.000	1.000
CACNB1	101	0	0.00%	196	0	0.00%	1.00 [0e+00-Inf]	1.000	1.000
CACNG1	101	0	0.00%	196	0	0.00%	1.00 [0e+00-Inf]	1.000	1.000
CACNG3	101	0	0.00%	196	0	0.00%	1.00 [0e+00-Inf]	1.000	1.000
CACNG4	101	0	0.00%	196	0	0.00%	1.00 [0e+00-Inf]	1.000	1.000
CACNG5	101	0	0.00%	196	0	0.00%	1.00 [0e+00-Inf]	1.000	1.000
CACYBP	101	0	0.00%	196	0	0.00%	1.00 [0e+00-Inf]	1.000	1.000
CADM3	101	0	0.00%	196	0	0.00%	1.00 [0e+00-Inf]	1.000	1.000
CALB1	101	0	0.00%	196	0	0.00%	1.00 [0e+00-Inf]	1.000	1.000
CALCA	101	0	0.00%	196	0	0.00%	1.00 [0e+00-Inf]	1.000	1.000
CALCB	101	0	0.00%	196	0	0.00%	1.00 [0e+00-Inf]	1.000	1.000
CALCOCO2	101	0	0.00%	196	0	0.00%	1.00 [0e+00-Inf]	1.000	1.000
CAMK1G	101	0	0.00%	196	0	0.00%	1.00 [0e+00-Inf]	1.000	1.000
CAMK2B	101	0	0.00%	196	0	0.00%	1.00 [0e+00-Inf]	1.000	1.000
CAMK2G	101	0	0.00%	196	0	0.00%	1.00 [0e+00-Inf]	1.000	1.000
CAMK2N2	101	0	0.00%	196	0	0.00%	1.00 [0e+00-Inf]	1.000	1.000
CAMSAP2	101	0	0.00%	196	0	0.00%	1.00 [0e+00-Inf]	1.000	1.000
CAND1	101	0	0.00%	196	0	0.00%	1.00 [0e+00-Inf]	1.000	1.000
CANT1	101	0	0.00%	196	0	0.00%	1.00 [0e+00-Inf]	1.000	1.000
CAPN2	101	0	0.00%	196	0	0.00%	1.00 [0e+00-Inf]	1.000	1.000
CAPN5	101	0	0.00%	196	0	0.00%	1.00 [0e+00-Inf]	1.000	1.000
CAPN8	101	0	0.00%	196	0	0.00%	1.00 [0e+00-Inf]	1.000	1.000
CAPN9	101	0	0.00%	196	0	0.00%	1.00 [0e+00-Inf]	1.000	1.000
CAPS2	101	0	0.00%	196	0	0.00%	1.00 [0e+00-Inf]	1.000	1.000
CARD14	101	0	0.00%	196	0	0.00%	1.00 [0e+00-Inf]	1.000	1.000
CARHSP1	101	0	0.00%	196	0	0.00%	1.00 [0e+00-Inf]	1.000	1.000
CARNS1	101	0	0.00%	196	0	0.00%	1.00 [0e+00-Inf]	1.000	1.000
CASC3	101	0	0.00%	196	0	0.00%	1.00 [0e+00-Inf]	1.000	1.000
CASKIN1	101	0	0.00%	196	0	0.00%	1.00 [0e+00-Inf]	1.000	1.000
CASKIN2	101	0	0.00%	196	0	0.00%	1.00 [0e+00-Inf]	1.000	1.000
CASQ1	101	0	0.00%	196	0	0.00%	1.00 [0e+00-Inf]	1.000	1.000
CASS4	101	0	0.00%	196	0	0.00%	1.00 [0e+00-Inf]	1.000	1.000
CATSPER1	101	0	0.00%	196	0	0.00%	1.00 [0e+00-Inf]	1.000	1.000
CBLN4	101	0	0.00%	196	0	0.00%	1.00 [0e+00-Inf]	1.000	1.000
CBX1	101	0	0.00%	196	0	0.00%	1.00 [0e+00-Inf]	1.000	1.000
CBX2	101	0	0.00%	196	0	0.00%	1.00 [0e+00-Inf]	1.000	1.000
CBX3	101	0	0.00%	196	0	0.00%	1.00 [0e+00-Inf]	1.000	1.000
CBX4	101	0	0.00%	196	0	0.00%	1.00 [0e+00-Inf]	1.000	1.000
CBX8	101	0	0.00%	196	0	0.00%	1.00 [0e+00-Inf]	1.000	1.000
CCBE1	101	0	0.00%	196	0	0.00%	1.00 [0e+00-Inf]	1.000	1.000
CCDC106	101	0	0.00%	196	0	0.00%	1.00 [0e+00-Inf]	1.000	1.000
MCU	101	0	0.00%	196	0	0.00%	1.00 [0e+00-Inf]	1.000	1.000
CCDC126	101	0	0.00%	196	0	0.00%	1.00 [0e+00-Inf]	1.000	1.000
CCDC137	101	0	0.00%	196	0	0.00%	1.00 [0e+00-Inf]	1.000	1.000
CCDC144B	101	0	0.00%	196	0	0.00%	1.00 [0e+00-Inf]	1.000	1.000
CCDC154	101	0	0.00%	196	0	0.00%	1.00 [0e+00-Inf]	1.000	1.000
CCDC158	101	0	0.00%	196	0	0.00%	1.00 [0e+00-Inf]	1.000	1.000
CCDC39	101	0	0.00%	196	0	0.00%	1.00 [0e+00-Inf]	1.000	1.000
CCDC40	101	0	0.00%	196	0	0.00%	1.00 [0e+00-Inf]	1.000	1.000
CEP95	101	0	0.00%	196	0	0.00%	1.00 [0e+00-Inf]	1.000	1.000
CEP112	101	0	0.00%	196	0	0.00%	1.00 [0e+00-Inf]	1.000	1.000
CCDC47	101	0	0.00%	196	0	0.00%	1.00 [0e+00-Inf]	1.000	1.000

Symbol	CINSARC Low			CINSARC High			CINSARC High vs. Low		
	N tested	N altered	Fq alteration	N tested	N altered	Fq alteration	OR [95%CI]	p-value*	q-value
CCDC50	101	0	0.00%	196	0	0.00%	1.00 [0e+00-Inf]	1.000	1.000
NSRP1	101	0	0.00%	196	0	0.00%	1.00 [0e+00-Inf]	1.000	1.000
CCDC57	101	0	0.00%	196	0	0.00%	1.00 [0e+00-Inf]	1.000	1.000
CCDC68	101	0	0.00%	196	0	0.00%	1.00 [0e+00-Inf]	1.000	1.000
CCDC78	101	0	0.00%	196	0	0.00%	1.00 [0e+00-Inf]	1.000	1.000
CCDC81	101	0	0.00%	196	0	0.00%	1.00 [0e+00-Inf]	1.000	1.000
CCDC83	101	0	0.00%	196	0	0.00%	1.00 [0e+00-Inf]	1.000	1.000
CCDC87	101	0	0.00%	196	0	0.00%	1.00 [0e+00-Inf]	1.000	1.000
CCDC89	101	0	0.00%	196	0	0.00%	1.00 [0e+00-Inf]	1.000	1.000
CCL14	101	0	0.00%	196	0	0.00%	1.00 [0e+00-Inf]	1.000	1.000
CCL15	101	0	0.00%	196	0	0.00%	1.00 [0e+00-Inf]	1.000	1.000
CCL16	101	0	0.00%	196	0	0.00%	1.00 [0e+00-Inf]	1.000	1.000
CCL19	101	0	0.00%	196	0	0.00%	1.00 [0e+00-Inf]	1.000	1.000
CCL21	101	0	0.00%	196	0	0.00%	1.00 [0e+00-Inf]	1.000	1.000
CCL23	101	0	0.00%	196	0	0.00%	1.00 [0e+00-Inf]	1.000	1.000
CCL27	101	0	0.00%	196	0	0.00%	1.00 [0e+00-Inf]	1.000	1.000
CCL3L1	101	0	0.00%	196	0	0.00%	1.00 [0e+00-Inf]	1.000	1.000
CCL3L3	101	0	0.00%	196	0	0.00%	1.00 [0e+00-Inf]	1.000	1.000
CCL3	101	0	0.00%	196	0	0.00%	1.00 [0e+00-Inf]	1.000	1.000
CCL4L2	101	0	0.00%	196	0	0.00%	1.00 [0e+00-Inf]	1.000	1.000
CCL4	101	0	0.00%	196	0	0.00%	1.00 [0e+00-Inf]	1.000	1.000
CCL5	101	0	0.00%	196	0	0.00%	1.00 [0e+00-Inf]	1.000	1.000
CCND1	101	0	0.00%	196	0	0.00%	1.00 [0e+00-Inf]	1.000	1.000
CCNE2	101	0	0.00%	196	0	0.00%	1.00 [0e+00-Inf]	1.000	1.000
CCNF	101	0	0.00%	196	0	0.00%	1.00 [0e+00-Inf]	1.000	1.000
CCR7	101	0	0.00%	196	0	0.00%	1.00 [0e+00-Inf]	1.000	1.000
CCS	101	0	0.00%	196	0	0.00%	1.00 [0e+00-Inf]	1.000	1.000
CCT2	101	0	0.00%	196	0	0.00%	1.00 [0e+00-Inf]	1.000	1.000
CCT3	101	0	0.00%	196	0	0.00%	1.00 [0e+00-Inf]	1.000	1.000
CCT6B	101	0	0.00%	196	0	0.00%	1.00 [0e+00-Inf]	1.000	1.000
CD160	101	0	0.00%	196	0	0.00%	1.00 [0e+00-Inf]	1.000	1.000
CD19	101	0	0.00%	196	0	0.00%	1.00 [0e+00-Inf]	1.000	1.000
CD1A	101	0	0.00%	196	0	0.00%	1.00 [0e+00-Inf]	1.000	1.000
CD1B	101	0	0.00%	196	0	0.00%	1.00 [0e+00-Inf]	1.000	1.000
CD1C	101	0	0.00%	196	0	0.00%	1.00 [0e+00-Inf]	1.000	1.000
CD1D	101	0	0.00%	196	0	0.00%	1.00 [0e+00-Inf]	1.000	1.000
CD1E	101	0	0.00%	196	0	0.00%	1.00 [0e+00-Inf]	1.000	1.000
CD244	101	0	0.00%	196	0	0.00%	1.00 [0e+00-Inf]	1.000	1.000
CD247	101	0	0.00%	196	0	0.00%	1.00 [0e+00-Inf]	1.000	1.000
CD248	101	0	0.00%	196	0	0.00%	1.00 [0e+00-Inf]	1.000	1.000
CD24	101	64	63.37%	196	116	59.18%	0.84 [0.55-1.27]	0.485	1.000
CD2BP2	101	0	0.00%	196	0	0.00%	1.00 [0e+00-Inf]	1.000	1.000
CD300A	101	0	0.00%	196	0	0.00%	1.00 [0e+00-Inf]	1.000	1.000
CD300C	101	0	0.00%	196	0	0.00%	1.00 [0e+00-Inf]	1.000	1.000
CD300E	101	0	0.00%	196	0	0.00%	1.00 [0e+00-Inf]	1.000	1.000
CD300LB	101	0	0.00%	196	0	0.00%	1.00 [0e+00-Inf]	1.000	1.000
CD300LD	101	0	0.00%	196	0	0.00%	1.00 [0e+00-Inf]	1.000	1.000
CD300LF	101	0	0.00%	196	0	0.00%	1.00 [0e+00-Inf]	1.000	1.000
CD34	101	0	0.00%	196	0	0.00%	1.00 [0e+00-Inf]	1.000	1.000
CD40	101	0	0.00%	196	0	0.00%	1.00 [0e+00-Inf]	1.000	1.000
CD44	101	0	0.00%	196	0	0.00%	1.00 [0e+00-Inf]	1.000	1.000
CD46	101	0	0.00%	196	0	0.00%	1.00 [0e+00-Inf]	1.000	1.000
CD48	101	0	0.00%	196	0	0.00%	1.00 [0e+00-Inf]	1.000	1.000
CD55	101	0	0.00%	196	0	0.00%	1.00 [0e+00-Inf]	1.000	1.000
CD59	101	0	0.00%	196	0	0.00%	1.00 [0e+00-Inf]	1.000	1.000
CD5L	101	0	0.00%	196	0	0.00%	1.00 [0e+00-Inf]	1.000	1.000
CD5	101	0	0.00%	196	0	0.00%	1.00 [0e+00-Inf]	1.000	1.000
CD79B	101	0	0.00%	196	0	0.00%	1.00 [0e+00-Inf]	1.000	1.000
CD7	101	0	0.00%	196	0	0.00%	1.00 [0e+00-Inf]	1.000	1.000
CD84	101	0	0.00%	196	0	0.00%	1.00 [0e+00-Inf]	1.000	1.000
CDC27	101	0	0.00%	196	0	0.00%	1.00 [0e+00-Inf]	1.000	1.000
CDC42BPA	101	0	0.00%	196	0	0.00%	1.00 [0e+00-Inf]	1.000	1.000
CDC42EP4	101	0	0.00%	196	0	0.00%	1.00 [0e+00-Inf]	1.000	1.000
CDC42SE1	101	0	0.00%	196	0	0.00%	1.00 [0e+00-Inf]	1.000	1.000
CDC6	101	0	0.00%	196	0	0.00%	1.00 [0e+00-Inf]	1.000	1.000
CDC73	101	0	0.00%	196	0	0.00%	1.00 [0e+00-Inf]	1.000	1.000
CDCA7L	101	0	0.00%	196	0	0.00%	1.00 [0e+00-Inf]	1.000	1.000
CDH17	101	0	0.00%	196	0	0.00%	1.00 [0e+00-Inf]	1.000	1.000
CDH20	101	0	0.00%	196	0	0.00%	1.00 [0e+00-Inf]	1.000	1.000
CDH22	101	0	0.00%	196	0	0.00%	1.00 [0e+00-Inf]	1.000	1.000
CDH24	101	0	0.00%	196	0	0.00%	1.00 [0e+00-Inf]	1.000	1.000

Symbol	CINSARC Low			CINSARC High			CINSARC High vs. Low		
	N tested	N altered	Fq alteration	N tested	N altered	Fq alteration	OR [95%CI]	p-value*	q-value
CDH26	101	0	0.00%	196	0	0.00%	1.00 [0e+00-Inf]	1.000	1.000
CDH4	101	0	0.00%	196	0	0.00%	1.00 [0e+00-Inf]	1.000	1.000
CDIPT	101	0	0.00%	196	0	0.00%	1.00 [0e+00-Inf]	1.000	1.000
CDK12	101	0	0.00%	196	0	0.00%	1.00 [0e+00-Inf]	1.000	1.000
CDK18	101	0	0.00%	196	0	0.00%	1.00 [0e+00-Inf]	1.000	1.000
CDK2AP2	101	0	0.00%	196	0	0.00%	1.00 [0e+00-Inf]	1.000	1.000
CDK3	101	0	0.00%	196	0	0.00%	1.00 [0e+00-Inf]	1.000	1.000
CDK4	101	0	0.00%	196	0	0.00%	1.00 [0e+00-Inf]	1.000	1.000
CDK5R1	101	0	0.00%	196	0	0.00%	1.00 [0e+00-Inf]	1.000	1.000
CDK5RAP3	101	0	0.00%	196	0	0.00%	1.00 [0e+00-Inf]	1.000	1.000
CDKL2	101	0	0.00%	196	0	0.00%	1.00 [0e+00-Inf]	1.000	1.000
CDR2L	101	0	0.00%	196	0	0.00%	1.00 [0e+00-Inf]	1.000	1.000
CDR2	101	0	0.00%	196	0	0.00%	1.00 [0e+00-Inf]	1.000	1.000
CDY1B	101	65	64.36%	196	117	59.69%	0.82 [0.54-1.25]	0.435	1.000
CDY1	101	65	64.36%	196	117	59.69%	0.82 [0.54-1.25]	0.435	1.000
CDY2B	101	65	64.36%	196	117	59.69%	0.82 [0.54-1.25]	0.435	1.000
CEBPB	101	0	0.00%	196	0	0.00%	1.00 [0e+00-Inf]	1.000	1.000
CEBPD	101	0	0.00%	196	0	0.00%	1.00 [0e+00-Inf]	1.000	1.000
CELF3	101	0	0.00%	196	0	0.00%	1.00 [0e+00-Inf]	1.000	1.000
CELF6	101	0	0.00%	196	0	0.00%	1.00 [0e+00-Inf]	1.000	1.000
CEMP1	101	0	0.00%	196	0	0.00%	1.00 [0e+00-Inf]	1.000	1.000
CENPF	101	0	0.00%	196	0	0.00%	1.00 [0e+00-Inf]	1.000	1.000
CENPL	101	0	0.00%	196	0	0.00%	1.00 [0e+00-Inf]	1.000	1.000
CEP170	101	0	0.00%	196	0	0.00%	1.00 [0e+00-Inf]	1.000	1.000
CEP250	101	0	0.00%	196	0	0.00%	1.00 [0e+00-Inf]	1.000	1.000
CEP290	101	0	0.00%	196	0	0.00%	1.00 [0e+00-Inf]	1.000	1.000
CEP350	101	0	0.00%	196	0	0.00%	1.00 [0e+00-Inf]	1.000	1.000
CFHR1	101	0	0.00%	196	0	0.00%	1.00 [0e+00-Inf]	1.000	1.000
CFHR2	101	0	0.00%	196	0	0.00%	1.00 [0e+00-Inf]	1.000	1.000
CFHR3	101	0	0.00%	196	0	0.00%	1.00 [0e+00-Inf]	1.000	1.000
CFHR4	101	0	0.00%	196	0	0.00%	1.00 [0e+00-Inf]	1.000	1.000
CFHR5	101	0	0.00%	196	0	0.00%	1.00 [0e+00-Inf]	1.000	1.000
CFH	101	0	0.00%	196	0	0.00%	1.00 [0e+00-Inf]	1.000	1.000
CFL2	101	0	0.00%	196	0	0.00%	1.00 [0e+00-Inf]	1.000	1.000
CGN	101	0	0.00%	196	0	0.00%	1.00 [0e+00-Inf]	1.000	1.000
CHAD	101	0	0.00%	196	0	0.00%	1.00 [0e+00-Inf]	1.000	1.000
CHCHD1	101	0	0.00%	196	0	0.00%	1.00 [0e+00-Inf]	1.000	1.000
CHCHD7	101	0	0.00%	196	0	0.00%	1.00 [0e+00-Inf]	1.000	1.000
COA4	101	0	0.00%	196	0	0.00%	1.00 [0e+00-Inf]	1.000	1.000
CHD1L	101	0	0.00%	196	0	0.00%	1.00 [0e+00-Inf]	1.000	1.000
CHD2	101	0	0.00%	196	0	0.00%	1.00 [0e+00-Inf]	1.000	1.000
CHD6	101	0	0.00%	196	0	0.00%	1.00 [0e+00-Inf]	1.000	1.000
CHD7	101	0	0.00%	196	0	0.00%	1.00 [0e+00-Inf]	1.000	1.000
CHI3L1	101	0	0.00%	196	0	0.00%	1.00 [0e+00-Inf]	1.000	1.000
CHIT1	101	0	0.00%	196	0	0.00%	1.00 [0e+00-Inf]	1.000	1.000
CHKA	101	0	0.00%	196	0	0.00%	1.00 [0e+00-Inf]	1.000	1.000
CHML	101	0	0.00%	196	0	0.00%	1.00 [0e+00-Inf]	1.000	1.000
CHMP4C	101	0	0.00%	196	0	0.00%	1.00 [0e+00-Inf]	1.000	1.000
CHMP6	101	0	0.00%	196	0	0.00%	1.00 [0e+00-Inf]	1.000	1.000
CHN2	101	0	0.00%	196	0	0.00%	1.00 [0e+00-Inf]	1.000	1.000
CHORDC1	101	0	0.00%	196	0	0.00%	1.00 [0e+00-Inf]	1.000	1.000
CHP2	101	0	0.00%	196	0	0.00%	1.00 [0e+00-Inf]	1.000	1.000
CHRAC1	101	0	0.00%	196	0	0.00%	1.00 [0e+00-Inf]	1.000	1.000
CHRDL2	101	0	0.00%	196	0	0.00%	1.00 [0e+00-Inf]	1.000	1.000
CHRD	101	0	0.00%	196	0	0.00%	1.00 [0e+00-Inf]	1.000	1.000
CHRM3	101	0	0.00%	196	0	0.00%	1.00 [0e+00-Inf]	1.000	1.000
CHRNA4	101	0	0.00%	196	0	0.00%	1.00 [0e+00-Inf]	1.000	1.000
CHRNA6	101	0	0.00%	196	0	0.00%	1.00 [0e+00-Inf]	1.000	1.000
CHRNB2	101	0	0.00%	196	0	0.00%	1.00 [0e+00-Inf]	1.000	1.000
CHRNB3	101	0	0.00%	196	0	0.00%	1.00 [0e+00-Inf]	1.000	1.000
CHTF18	101	0	0.00%	196	0	0.00%	1.00 [0e+00-Inf]	1.000	1.000
CIB1	101	0	0.00%	196	0	0.00%	1.00 [0e+00-Inf]	1.000	1.000
CIB2	101	0	0.00%	196	0	0.00%	1.00 [0e+00-Inf]	1.000	1.000
CIITA	101	0	0.00%	196	0	0.00%	1.00 [0e+00-Inf]	1.000	1.000
CISD3	101	0	0.00%	196	0	0.00%	1.00 [0e+00-Inf]	1.000	1.000
CKS1B	101	0	0.00%	196	0	0.00%	1.00 [0e+00-Inf]	1.000	1.000
CLCF1	101	0	0.00%	196	0	0.00%	1.00 [0e+00-Inf]	1.000	1.000
CLCN2	101	0	0.00%	196	0	0.00%	1.00 [0e+00-Inf]	1.000	1.000
CLCN7	101	0	0.00%	196	0	0.00%	1.00 [0e+00-Inf]	1.000	1.000
CLDN11	101	0	0.00%	196	0	0.00%	1.00 [0e+00-Inf]	1.000	1.000
CLDN16	101	0	0.00%	196	0	0.00%	1.00 [0e+00-Inf]	1.000	1.000

Symbol	CINSARC Low			CINSARC High			CINSARC High vs. Low		
	N tested	N altered	Fq alteration	N tested	N altered	Fq alteration	OR [95%CI]	p-value*	q-value
CLDN1	101	0	0.00%	196	0	0.00%	1.00 [0e+00-Inf]	1.000	1.000
CLDN6	101	0	0.00%	196	0	0.00%	1.00 [0e+00-Inf]	1.000	1.000
CLDN9	101	0	0.00%	196	0	0.00%	1.00 [0e+00-Inf]	1.000	1.000
CLEC14A	101	0	0.00%	196	0	0.00%	1.00 [0e+00-Inf]	1.000	1.000
CLEC16A	101	0	0.00%	196	0	0.00%	1.00 [0e+00-Inf]	1.000	1.000
CLK2	101	0	0.00%	196	0	0.00%	1.00 [0e+00-Inf]	1.000	1.000
CLLU1OS	101	0	0.00%	196	0	0.00%	1.00 [0e+00-Inf]	1.000	1.000
CLLU1	101	0	0.00%	196	0	0.00%	1.00 [0e+00-Inf]	1.000	1.000
CLN3	101	0	0.00%	196	0	0.00%	1.00 [0e+00-Inf]	1.000	1.000
CLNS1A	101	0	0.00%	196	0	0.00%	1.00 [0e+00-Inf]	1.000	1.000
CLPB	101	0	0.00%	196	0	0.00%	1.00 [0e+00-Inf]	1.000	1.000
CLRN1-AS1	101	0	0.00%	196	0	0.00%	1.00 [0e+00-Inf]	1.000	1.000
CLRN1	101	0	0.00%	196	0	0.00%	1.00 [0e+00-Inf]	1.000	1.000
CLTC	101	0	0.00%	196	0	0.00%	1.00 [0e+00-Inf]	1.000	1.000
CLUAP1	101	0	0.00%	196	0	0.00%	1.00 [0e+00-Inf]	1.000	1.000
CLVS1	101	0	0.00%	196	0	0.00%	1.00 [0e+00-Inf]	1.000	1.000
CNBD1	101	0	0.00%	196	0	0.00%	1.00 [0e+00-Inf]	1.000	1.000
CNGB3	101	0	0.00%	196	0	0.00%	1.00 [0e+00-Inf]	1.000	1.000
CNIH2	101	0	0.00%	196	0	0.00%	1.00 [0e+00-Inf]	1.000	1.000
CNIH3	101	0	0.00%	196	0	0.00%	1.00 [0e+00-Inf]	1.000	1.000
CNIH4	101	0	0.00%	196	0	0.00%	1.00 [0e+00-Inf]	1.000	1.000
CNOT2	101	0	0.00%	196	0	0.00%	1.00 [0e+00-Inf]	1.000	1.000
CNST	101	0	0.00%	196	0	0.00%	1.00 [0e+00-Inf]	1.000	1.000
CNTFR	101	0	0.00%	196	0	0.00%	1.00 [0e+00-Inf]	1.000	1.000
CNTN2	101	0	0.00%	196	0	0.00%	1.00 [0e+00-Inf]	1.000	1.000
COCH	101	0	0.00%	196	0	0.00%	1.00 [0e+00-Inf]	1.000	1.000
COG1	101	0	0.00%	196	0	0.00%	1.00 [0e+00-Inf]	1.000	1.000
COG2	101	0	0.00%	196	0	0.00%	1.00 [0e+00-Inf]	1.000	1.000
COG7	101	0	0.00%	196	0	0.00%	1.00 [0e+00-Inf]	1.000	1.000
COIL	101	0	0.00%	196	0	0.00%	1.00 [0e+00-Inf]	1.000	1.000
COL14A1	101	0	0.00%	196	0	0.00%	1.00 [0e+00-Inf]	1.000	1.000
COL1A1	101	0	0.00%	196	0	0.00%	1.00 [0e+00-Inf]	1.000	1.000
COL20A1	101	0	0.00%	196	0	0.00%	1.00 [0e+00-Inf]	1.000	1.000
COL22A1	101	0	0.00%	196	0	0.00%	1.00 [0e+00-Inf]	1.000	1.000
COL9A3	101	0	0.00%	196	0	0.00%	1.00 [0e+00-Inf]	1.000	1.000
COLEC10	101	0	0.00%	196	0	0.00%	1.00 [0e+00-Inf]	1.000	1.000
COMMD5	101	0	0.00%	196	0	0.00%	1.00 [0e+00-Inf]	1.000	1.000
COMTD1	101	0	0.00%	196	0	0.00%	1.00 [0e+00-Inf]	1.000	1.000
COPA	101	0	0.00%	196	0	0.00%	1.00 [0e+00-Inf]	1.000	1.000
COPS5	101	0	0.00%	196	0	0.00%	1.00 [0e+00-Inf]	1.000	1.000
COPZ2	101	0	0.00%	196	0	0.00%	1.00 [0e+00-Inf]	1.000	1.000
COQ7	101	0	0.00%	196	0	0.00%	1.00 [0e+00-Inf]	1.000	1.000
CORO1A	101	0	0.00%	196	0	0.00%	1.00 [0e+00-Inf]	1.000	1.000
CORO1B	101	0	0.00%	196	0	0.00%	1.00 [0e+00-Inf]	1.000	1.000
CORO6	101	0	0.00%	196	0	0.00%	1.00 [0e+00-Inf]	1.000	1.000
CORO7	101	0	0.00%	196	0	0.00%	1.00 [0e+00-Inf]	1.000	1.000
COX11	101	0	0.00%	196	0	0.00%	1.00 [0e+00-Inf]	1.000	1.000
COX18	101	0	0.00%	196	0	0.00%	1.00 [0e+00-Inf]	1.000	1.000
COX6A2	101	0	0.00%	196	0	0.00%	1.00 [0e+00-Inf]	1.000	1.000
COX6B2	101	0	0.00%	196	0	0.00%	1.00 [0e+00-Inf]	1.000	1.000
COX6C	101	0	0.00%	196	0	0.00%	1.00 [0e+00-Inf]	1.000	1.000
CCP110	101	0	0.00%	196	0	0.00%	1.00 [0e+00-Inf]	1.000	1.000
CPA6	101	0	0.00%	196	0	0.00%	1.00 [0e+00-Inf]	1.000	1.000
CPD	101	0	0.00%	196	0	0.00%	1.00 [0e+00-Inf]	1.000	1.000
CPEB1	101	0	0.00%	196	0	0.00%	1.00 [0e+00-Inf]	1.000	1.000
CPLX4	101	0	0.00%	196	0	0.00%	1.00 [0e+00-Inf]	1.000	1.000
CPM	101	0	0.00%	196	0	0.00%	1.00 [0e+00-Inf]	1.000	1.000
CPN2	101	0	0.00%	196	0	0.00%	1.00 [0e+00-Inf]	1.000	1.000
CPNE1	101	0	0.00%	196	0	0.00%	1.00 [0e+00-Inf]	1.000	1.000
CPNE3	101	0	0.00%	196	0	0.00%	1.00 [0e+00-Inf]	1.000	1.000
CPPED1	101	0	0.00%	196	0	0.00%	1.00 [0e+00-Inf]	1.000	1.000
CPSF1	101	0	0.00%	196	0	0.00%	1.00 [0e+00-Inf]	1.000	1.000
CPSF4L	101	0	0.00%	196	0	0.00%	1.00 [0e+00-Inf]	1.000	1.000
CPSF6	101	0	0.00%	196	0	0.00%	1.00 [0e+00-Inf]	1.000	1.000
CPSF7	101	0	0.00%	196	0	0.00%	1.00 [0e+00-Inf]	1.000	1.000
CPT1A	101	0	0.00%	196	0	0.00%	1.00 [0e+00-Inf]	1.000	1.000
CPVL	101	0	0.00%	196	0	0.00%	1.00 [0e+00-Inf]	1.000	1.000
CP	101	0	0.00%	196	0	0.00%	1.00 [0e+00-Inf]	1.000	1.000
CR1L	101	0	0.00%	196	0	0.00%	1.00 [0e+00-Inf]	1.000	1.000
CR1	101	0	0.00%	196	0	0.00%	1.00 [0e+00-Inf]	1.000	1.000
CR2	101	0	0.00%	196	0	0.00%	1.00 [0e+00-Inf]	1.000	1.000

Symbol	CINSARC Low			CINSARC High			CINSARC High vs. Low		
	N tested	N altered	Fq alteration	N tested	N altered	Fq alteration	OR [95%CI]	p-value*	q-value
CRABP2	101	0	0.00%	196	0	0.00%	1.00 [0e+00-Inf]	1.000	1.000
CRB1	101	0	0.00%	196	0	0.00%	1.00 [0e+00-Inf]	1.000	1.000
CRCT1	101	0	0.00%	196	0	0.00%	1.00 [0e+00-Inf]	1.000	1.000
CREB3L4	101	0	0.00%	196	0	0.00%	1.00 [0e+00-Inf]	1.000	1.000
CREB5	101	0	0.00%	196	0	0.00%	1.00 [0e+00-Inf]	1.000	1.000
CREBBP	101	0	0.00%	196	0	0.00%	1.00 [0e+00-Inf]	1.000	1.000
CREBZF	101	0	0.00%	196	0	0.00%	1.00 [0e+00-Inf]	1.000	1.000
CREG1	101	0	0.00%	196	0	0.00%	1.00 [0e+00-Inf]	1.000	1.000
CRHR2	101	0	0.00%	196	0	0.00%	1.00 [0e+00-Inf]	1.000	1.000
CRH	101	0	0.00%	196	0	0.00%	1.00 [0e+00-Inf]	1.000	1.000
CRISPLD1	101	0	0.00%	196	0	0.00%	1.00 [0e+00-Inf]	1.000	1.000
CRLF3	101	0	0.00%	196	0	0.00%	1.00 [0e+00-Inf]	1.000	1.000
CRNN	101	0	0.00%	196	0	0.00%	1.00 [0e+00-Inf]	1.000	1.000
CRP	101	0	0.00%	196	0	0.00%	1.00 [0e+00-Inf]	1.000	1.000
CRTC2	101	0	0.00%	196	0	0.00%	1.00 [0e+00-Inf]	1.000	1.000
CRTC3	101	0	0.00%	196	0	0.00%	1.00 [0e+00-Inf]	1.000	1.000
CRYBA1	101	0	0.00%	196	0	0.00%	1.00 [0e+00-Inf]	1.000	1.000
CRYM	101	0	0.00%	196	0	0.00%	1.00 [0e+00-Inf]	1.000	1.000
YBX3P1	101	0	0.00%	196	0	0.00%	1.00 [0e+00-Inf]	1.000	1.000
CSE1L	101	0	0.00%	196	0	0.00%	1.00 [0e+00-Inf]	1.000	1.000
CSF3	101	0	0.00%	196	0	0.00%	1.00 [0e+00-Inf]	1.000	1.000
CSH1	101	0	0.00%	196	0	0.00%	1.00 [0e+00-Inf]	1.000	1.000
CSH2	101	0	0.00%	196	0	0.00%	1.00 [0e+00-Inf]	1.000	1.000
CSHL1	101	0	0.00%	196	0	0.00%	1.00 [0e+00-Inf]	1.000	1.000
CSMD1	101	3	2.97%	196	3	1.53%	0.51 [0.13-1.98]	0.412	1.000
CSMD3	101	0	0.00%	196	0	0.00%	1.00 [0e+00-Inf]	1.000	1.000
CSNK1D	101	0	0.00%	196	0	0.00%	1.00 [0e+00-Inf]	1.000	1.000
CSPP1	101	0	0.00%	196	0	0.00%	1.00 [0e+00-Inf]	1.000	1.000
CSRP1	101	0	0.00%	196	0	0.00%	1.00 [0e+00-Inf]	1.000	1.000
CSRP2	101	0	0.00%	196	0	0.00%	1.00 [0e+00-Inf]	1.000	1.000
CSRP3	101	0	0.00%	196	0	0.00%	1.00 [0e+00-Inf]	1.000	1.000
CST6	101	0	0.00%	196	0	0.00%	1.00 [0e+00-Inf]	1.000	1.000
CSTF1	101	0	0.00%	196	0	0.00%	1.00 [0e+00-Inf]	1.000	1.000
CTAGE4	101	0	0.00%	196	0	0.00%	1.00 [0e+00-Inf]	1.000	1.000
CTAGE6	101	0	0.00%	196	0	0.00%	1.00 [0e+00-Inf]	1.000	1.000
CTCFL	101	0	0.00%	196	0	0.00%	1.00 [0e+00-Inf]	1.000	1.000
CTDSP2	101	0	0.00%	196	0	0.00%	1.00 [0e+00-Inf]	1.000	1.000
CTF1	101	0	0.00%	196	0	0.00%	1.00 [0e+00-Inf]	1.000	1.000
CTHRC1	101	0	0.00%	196	0	0.00%	1.00 [0e+00-Inf]	1.000	1.000
CTNNBL1	101	0	0.00%	196	0	0.00%	1.00 [0e+00-Inf]	1.000	1.000
CTSA	101	0	0.00%	196	0	0.00%	1.00 [0e+00-Inf]	1.000	1.000
CTSC	101	0	0.00%	196	0	0.00%	1.00 [0e+00-Inf]	1.000	1.000
CTSE	101	0	0.00%	196	0	0.00%	1.00 [0e+00-Inf]	1.000	1.000
CTSF	101	0	0.00%	196	0	0.00%	1.00 [0e+00-Inf]	1.000	1.000
CTSK	101	0	0.00%	196	0	0.00%	1.00 [0e+00-Inf]	1.000	1.000
CTSS	101	0	0.00%	196	0	0.00%	1.00 [0e+00-Inf]	1.000	1.000
CTSZ	101	0	0.00%	196	0	0.00%	1.00 [0e+00-Inf]	1.000	1.000
CTTN	101	0	0.00%	196	0	0.00%	1.00 [0e+00-Inf]	1.000	1.000
CUEDC1	101	0	0.00%	196	0	0.00%	1.00 [0e+00-Inf]	1.000	1.000
CWC25	101	0	0.00%	196	0	0.00%	1.00 [0e+00-Inf]	1.000	1.000
CXADRP2	101	0	0.00%	196	0	0.00%	1.00 [0e+00-Inf]	1.000	1.000
CXCL10	101	0	0.00%	196	0	0.00%	1.00 [0e+00-Inf]	1.000	1.000
CXCL11	101	0	0.00%	196	0	0.00%	1.00 [0e+00-Inf]	1.000	1.000
CXCL1	101	0	0.00%	196	0	0.00%	1.00 [0e+00-Inf]	1.000	1.000
CXCL2	101	0	0.00%	196	0	0.00%	1.00 [0e+00-Inf]	1.000	1.000
CXCL3	101	0	0.00%	196	0	0.00%	1.00 [0e+00-Inf]	1.000	1.000
CXCL5	101	0	0.00%	196	0	0.00%	1.00 [0e+00-Inf]	1.000	1.000
CXCL6	101	0	0.00%	196	0	0.00%	1.00 [0e+00-Inf]	1.000	1.000
CXCL9	101	0	0.00%	196	0	0.00%	1.00 [0e+00-Inf]	1.000	1.000
CYB561	101	0	0.00%	196	0	0.00%	1.00 [0e+00-Inf]	1.000	1.000
CYB5R1	101	0	0.00%	196	0	0.00%	1.00 [0e+00-Inf]	1.000	1.000
CYB561A3	101	0	0.00%	196	0	0.00%	1.00 [0e+00-Inf]	1.000	1.000
CYC1	101	0	0.00%	196	0	0.00%	1.00 [0e+00-Inf]	1.000	1.000
CYCSP52	101	0	0.00%	196	0	0.00%	1.00 [0e+00-Inf]	1.000	1.000
CYCS	101	0	0.00%	196	0	0.00%	1.00 [0e+00-Inf]	1.000	1.000
CYGB	101	0	0.00%	196	0	0.00%	1.00 [0e+00-Inf]	1.000	1.000
CYHR1	101	0	0.00%	196	0	0.00%	1.00 [0e+00-Inf]	1.000	1.000
CYP11B1	101	0	0.00%	196	0	0.00%	1.00 [0e+00-Inf]	1.000	1.000
CYP11B2	101	0	0.00%	196	0	0.00%	1.00 [0e+00-Inf]	1.000	1.000
CYP24A1	101	0	0.00%	196	0	0.00%	1.00 [0e+00-Inf]	1.000	1.000
CYP27B1	101	0	0.00%	196	0	0.00%	1.00 [0e+00-Inf]	1.000	1.000

Symbol	CINSARC Low			CINSARC High			CINSARC High vs. Low		
	N tested	N altered	Fq alteration	N tested	N altered	Fq alteration	OR [95%CI]	p-value*	q-value
CYP7A1	101	0	0.00%	196	0	0.00%	1.00 [0e+00-Inf]	1.000	1.000
CYP7B1	101	0	0.00%	196	0	0.00%	1.00 [0e+00-Inf]	1.000	1.000
CYTH1	101	0	0.00%	196	0	0.00%	1.00 [0e+00-Inf]	1.000	1.000
DAP3	101	0	0.00%	196	0	0.00%	1.00 [0e+00-Inf]	1.000	1.000
DARS2	101	0	0.00%	196	0	0.00%	1.00 [0e+00-Inf]	1.000	1.000
DAZ1	101	65	64.36%	196	117	59.69%	0.82 [0.54-1.25]	0.435	1.000
DAZ2	101	65	64.36%	196	117	59.69%	0.82 [0.54-1.25]	0.435	1.000
DAZ3	101	65	64.36%	196	117	59.69%	0.82 [0.54-1.25]	0.435	1.000
DAZ4	101	65	64.36%	196	117	59.69%	0.82 [0.54-1.25]	0.435	1.000
DBNDD2	101	0	0.00%	196	0	0.00%	1.00 [0e+00-Inf]	1.000	1.000
DCAF13	101	0	0.00%	196	0	0.00%	1.00 [0e+00-Inf]	1.000	1.000
DCAF4L2	101	0	0.00%	196	0	0.00%	1.00 [0e+00-Inf]	1.000	1.000
DCAF6	101	0	0.00%	196	0	0.00%	1.00 [0e+00-Inf]	1.000	1.000
DCAF7	101	0	0.00%	196	0	0.00%	1.00 [0e+00-Inf]	1.000	1.000
DCAF8	101	0	0.00%	196	0	0.00%	1.00 [0e+00-Inf]	1.000	1.000
ECI1	101	0	0.00%	196	0	0.00%	1.00 [0e+00-Inf]	1.000	1.000
DCN	101	0	0.00%	196	0	0.00%	1.00 [0e+00-Inf]	1.000	1.000
DCST1	101	0	0.00%	196	0	0.00%	1.00 [0e+00-Inf]	1.000	1.000
DCST2	101	0	0.00%	196	0	0.00%	1.00 [0e+00-Inf]	1.000	1.000
DCTN3	101	0	0.00%	196	0	0.00%	1.00 [0e+00-Inf]	1.000	1.000
DCTN5	101	0	0.00%	196	0	0.00%	1.00 [0e+00-Inf]	1.000	1.000
DCTPP1	101	0	0.00%	196	0	0.00%	1.00 [0e+00-Inf]	1.000	1.000
DCUN1D1	101	0	0.00%	196	0	0.00%	1.00 [0e+00-Inf]	1.000	1.000
DCUN1D3	101	0	0.00%	196	0	0.00%	1.00 [0e+00-Inf]	1.000	1.000
DCXR	101	0	0.00%	196	0	0.00%	1.00 [0e+00-Inf]	1.000	1.000
DDB1	101	0	0.00%	196	0	0.00%	1.00 [0e+00-Inf]	1.000	1.000
DDHD2	101	0	0.00%	196	0	0.00%	1.00 [0e+00-Inf]	1.000	1.000
DDR2	101	0	0.00%	196	0	0.00%	1.00 [0e+00-Inf]	1.000	1.000
DDX27	101	0	0.00%	196	0	0.00%	1.00 [0e+00-Inf]	1.000	1.000
DDX3Y	101	64	63.37%	196	115	58.67%	0.82 [0.54-1.24]	0.434	1.000
DDX42	101	0	0.00%	196	0	0.00%	1.00 [0e+00-Inf]	1.000	1.000
DDX52	101	0	0.00%	196	0	0.00%	1.00 [0e+00-Inf]	1.000	1.000
DDX59	101	0	0.00%	196	0	0.00%	1.00 [0e+00-Inf]	1.000	1.000
DDX5	101	0	0.00%	196	0	0.00%	1.00 [0e+00-Inf]	1.000	1.000
DECR1	101	0	0.00%	196	0	0.00%	1.00 [0e+00-Inf]	1.000	1.000
DECR2	101	0	0.00%	196	0	0.00%	1.00 [0e+00-Inf]	1.000	1.000
DEDD	101	0	0.00%	196	0	0.00%	1.00 [0e+00-Inf]	1.000	1.000
DEFB108B	101	0	0.00%	196	0	0.00%	1.00 [0e+00-Inf]	1.000	1.000
DEGS1	101	0	0.00%	196	0	0.00%	1.00 [0e+00-Inf]	1.000	1.000
DENND1B	101	0	0.00%	196	0	0.00%	1.00 [0e+00-Inf]	1.000	1.000
DENND3	101	0	0.00%	196	0	0.00%	1.00 [0e+00-Inf]	1.000	1.000
DENND4B	101	0	0.00%	196	0	0.00%	1.00 [0e+00-Inf]	1.000	1.000
DEPTOR	101	0	0.00%	196	0	0.00%	1.00 [0e+00-Inf]	1.000	1.000
DERL1	101	0	0.00%	196	0	0.00%	1.00 [0e+00-Inf]	1.000	1.000
DET1	101	0	0.00%	196	0	0.00%	1.00 [0e+00-Inf]	1.000	1.000
DEXI	101	0	0.00%	196	0	0.00%	1.00 [0e+00-Inf]	1.000	1.000
DGAT1	101	0	0.00%	196	0	0.00%	1.00 [0e+00-Inf]	1.000	1.000
DGAT2	101	0	0.00%	196	0	0.00%	1.00 [0e+00-Inf]	1.000	1.000
DGKE	101	0	0.00%	196	0	0.00%	1.00 [0e+00-Inf]	1.000	1.000
DHCR7	101	0	0.00%	196	0	0.00%	1.00 [0e+00-Inf]	1.000	1.000
DHRS11	101	0	0.00%	196	0	0.00%	1.00 [0e+00-Inf]	1.000	1.000
DHRS13	101	0	0.00%	196	0	0.00%	1.00 [0e+00-Inf]	1.000	1.000
DHX35	101	0	0.00%	196	0	0.00%	1.00 [0e+00-Inf]	1.000	1.000
DHX40	101	0	0.00%	196	0	0.00%	1.00 [0e+00-Inf]	1.000	1.000
DHX9	101	0	0.00%	196	0	0.00%	1.00 [0e+00-Inf]	1.000	1.000
DIDO1	101	0	0.00%	196	0	0.00%	1.00 [0e+00-Inf]	1.000	1.000
DISC1	101	0	0.00%	196	0	0.00%	1.00 [0e+00-Inf]	1.000	1.000
DISC2	101	0	0.00%	196	0	0.00%	1.00 [0e+00-Inf]	1.000	1.000
DISP1	101	0	0.00%	196	0	0.00%	1.00 [0e+00-Inf]	1.000	1.000
DKFZP586I14	101	0	0.00%	196	0	0.00%	1.00 [0e+00-Inf]	1.000	1.000
NCR3LG1	101	0	0.00%	196	0	0.00%	1.00 [0e+00-Inf]	1.000	1.000
DKK4	101	0	0.00%	196	0	0.00%	1.00 [0e+00-Inf]	1.000	1.000
DLG1	101	0	0.00%	196	0	0.00%	1.00 [0e+00-Inf]	1.000	1.000
DLGAP4	101	0	0.00%	196	0	0.00%	1.00 [0e+00-Inf]	1.000	1.000
DLX3	101	0	0.00%	196	0	0.00%	1.00 [0e+00-Inf]	1.000	1.000
DLX4	101	0	0.00%	196	0	0.00%	1.00 [0e+00-Inf]	1.000	1.000
DNAH11	101	0	0.00%	196	0	0.00%	1.00 [0e+00-Inf]	1.000	1.000
DNAH14	101	0	0.00%	196	0	0.00%	1.00 [0e+00-Inf]	1.000	1.000
DNAH17	101	0	0.00%	196	0	0.00%	1.00 [0e+00-Inf]	1.000	1.000
DNAH3	101	0	0.00%	196	0	0.00%	1.00 [0e+00-Inf]	1.000	1.000
DNAI2	101	0	0.00%	196	0	0.00%	1.00 [0e+00-Inf]	1.000	1.000

Symbol	CINSARC Low			CINSARC High			CINSARC High vs. Low		
	N tested	N altered	Fq alteration	N tested	N altered	Fq alteration	OR [95%CI]	p-value*	q-value
DNAJA3	101	0	0.00%	196	0	0.00%	1.00 [0e+00-Inf]	1.000	1.000
DNAJB13	101	0	0.00%	196	0	0.00%	1.00 [0e+00-Inf]	1.000	1.000
DNAJB5	101	0	0.00%	196	0	0.00%	1.00 [0e+00-Inf]	1.000	1.000
DNAJB6	101	0	0.00%	196	0	0.00%	1.00 [0e+00-Inf]	1.000	1.000
DNAJC19	101	0	0.00%	196	0	0.00%	1.00 [0e+00-Inf]	1.000	1.000
DNAJC5B	101	0	0.00%	196	0	0.00%	1.00 [0e+00-Inf]	1.000	1.000
DNAJC5	101	0	0.00%	196	0	0.00%	1.00 [0e+00-Inf]	1.000	1.000
DNAJC9	101	0	0.00%	196	0	0.00%	1.00 [0e+00-Inf]	1.000	1.000
DNASE1L2	101	0	0.00%	196	0	0.00%	1.00 [0e+00-Inf]	1.000	1.000
DNASE1	101	0	0.00%	196	1	0.51%	3.2e+07 [0e+00-Inf]	0.997	1.000
DNM3	101	0	0.00%	196	0	0.00%	1.00 [0e+00-Inf]	1.000	1.000
DNTTIP1	101	0	0.00%	196	0	0.00%	1.00 [0e+00-Inf]	1.000	1.000
DOC2A	101	0	0.00%	196	0	0.00%	1.00 [0e+00-Inf]	1.000	1.000
DOK5	101	0	0.00%	196	0	0.00%	1.00 [0e+00-Inf]	1.000	1.000
DPH3P1	101	0	0.00%	196	0	0.00%	1.00 [0e+00-Inf]	1.000	1.000
DPM1	101	0	0.00%	196	0	0.00%	1.00 [0e+00-Inf]	1.000	1.000
DPM3	101	0	0.00%	196	0	0.00%	1.00 [0e+00-Inf]	1.000	1.000
DPP3	101	0	0.00%	196	0	0.00%	1.00 [0e+00-Inf]	1.000	1.000
DPRXP4	101	0	0.00%	196	0	0.00%	1.00 [0e+00-Inf]	1.000	1.000
DPT	101	0	0.00%	196	0	0.00%	1.00 [0e+00-Inf]	1.000	1.000
DPY19L1	101	0	0.00%	196	0	0.00%	1.00 [0e+00-Inf]	1.000	1.000
DPY19L2P1	101	0	0.00%	196	0	0.00%	1.00 [0e+00-Inf]	1.000	1.000
DPY19L2	101	0	0.00%	196	0	0.00%	1.00 [0e+00-Inf]	1.000	1.000
DPY19L4	101	0	0.00%	196	0	0.00%	1.00 [0e+00-Inf]	1.000	1.000
DPYS	101	0	0.00%	196	0	0.00%	1.00 [0e+00-Inf]	1.000	1.000
DSCC1	101	0	0.00%	196	0	0.00%	1.00 [0e+00-Inf]	1.000	1.000
DSG1	101	0	0.00%	196	0	0.00%	1.00 [0e+00-Inf]	1.000	1.000
DSG4	101	0	0.00%	196	0	0.00%	1.00 [0e+00-Inf]	1.000	1.000
DSN1	101	0	0.00%	196	0	0.00%	1.00 [0e+00-Inf]	1.000	1.000
DSTYK	101	0	0.00%	196	0	0.00%	1.00 [0e+00-Inf]	1.000	1.000
DTL	101	0	0.00%	196	0	0.00%	1.00 [0e+00-Inf]	1.000	1.000
DUPD1	101	0	0.00%	196	0	0.00%	1.00 [0e+00-Inf]	1.000	1.000
DUS1L	101	0	0.00%	196	0	0.00%	1.00 [0e+00-Inf]	1.000	1.000
DUSP10	101	0	0.00%	196	0	0.00%	1.00 [0e+00-Inf]	1.000	1.000
DUSP12	101	0	0.00%	196	0	0.00%	1.00 [0e+00-Inf]	1.000	1.000
DUSP13	101	0	0.00%	196	0	0.00%	1.00 [0e+00-Inf]	1.000	1.000
DUSP14	101	0	0.00%	196	0	0.00%	1.00 [0e+00-Inf]	1.000	1.000
DUSP23	101	0	0.00%	196	0	0.00%	1.00 [0e+00-Inf]	1.000	1.000
DUSP26	101	0	0.00%	196	0	0.00%	1.00 [0e+00-Inf]	1.000	1.000
DUSP27	101	0	0.00%	196	0	0.00%	1.00 [0e+00-Inf]	1.000	1.000
DUSP5P1	101	0	0.00%	196	0	0.00%	1.00 [0e+00-Inf]	1.000	1.000
DUSP6	101	0	0.00%	196	0	0.00%	1.00 [0e+00-Inf]	1.000	1.000
DVL3	101	0	0.00%	196	0	0.00%	1.00 [0e+00-Inf]	1.000	1.000
DYNLL2	101	0	0.00%	196	0	0.00%	1.00 [0e+00-Inf]	1.000	1.000
DYNLRB1	101	0	0.00%	196	0	0.00%	1.00 [0e+00-Inf]	1.000	1.000
DYRK2	101	0	0.00%	196	0	0.00%	1.00 [0e+00-Inf]	1.000	1.000
DYRK3	101	0	0.00%	196	0	0.00%	1.00 [0e+00-Inf]	1.000	1.000
PPP1R27	101	0	0.00%	196	0	0.00%	1.00 [0e+00-Inf]	1.000	1.000
E2F5	101	0	0.00%	196	0	0.00%	1.00 [0e+00-Inf]	1.000	1.000
E2F7	101	0	0.00%	196	0	0.00%	1.00 [0e+00-Inf]	1.000	1.000
E2F8	101	0	0.00%	196	0	0.00%	1.00 [0e+00-Inf]	1.000	1.000
E4F1	101	0	0.00%	196	0	0.00%	1.00 [0e+00-Inf]	1.000	1.000
EAPP	101	0	0.00%	196	0	0.00%	1.00 [0e+00-Inf]	1.000	1.000
EARS2	101	0	0.00%	196	0	0.00%	1.00 [0e+00-Inf]	1.000	1.000
EBAG9	101	0	0.00%	196	0	0.00%	1.00 [0e+00-Inf]	1.000	1.000
ECD	101	0	0.00%	196	0	0.00%	1.00 [0e+00-Inf]	1.000	1.000
ECE2	101	0	0.00%	196	0	0.00%	1.00 [0e+00-Inf]	1.000	1.000
ECM1	101	0	0.00%	196	0	0.00%	1.00 [0e+00-Inf]	1.000	1.000
EDARADD	101	0	0.00%	196	0	0.00%	1.00 [0e+00-Inf]	1.000	1.000
EDEM2	101	0	0.00%	196	0	0.00%	1.00 [0e+00-Inf]	1.000	1.000
EDEM3	101	0	0.00%	196	0	0.00%	1.00 [0e+00-Inf]	1.000	1.000
EDN3	101	0	0.00%	196	0	0.00%	1.00 [0e+00-Inf]	1.000	1.000
EEA1	101	0	0.00%	196	0	0.00%	1.00 [0e+00-Inf]	1.000	1.000
EED	101	0	0.00%	196	0	0.00%	1.00 [0e+00-Inf]	1.000	1.000
EEF1A2	101	0	0.00%	196	0	0.00%	1.00 [0e+00-Inf]	1.000	1.000
EEF1D	101	0	0.00%	196	0	0.00%	1.00 [0e+00-Inf]	1.000	1.000
EEF2K	101	0	0.00%	196	0	0.00%	1.00 [0e+00-Inf]	1.000	1.000
EFCAB1	101	0	0.00%	196	0	0.00%	1.00 [0e+00-Inf]	1.000	1.000
EFCAB2	101	0	0.00%	196	0	0.00%	1.00 [0e+00-Inf]	1.000	1.000
EFCAB3	101	0	0.00%	196	0	0.00%	1.00 [0e+00-Inf]	1.000	1.000
EFCAB5	101	0	0.00%	196	0	0.00%	1.00 [0e+00-Inf]	1.000	1.000

Symbol	CINSARC Low			CINSARC High			CINSARC High vs. Low		
	N tested	N altered	Fq alteration	N tested	N altered	Fq alteration	OR [95%CI]	p-value*	q-value
EFNA1	101	0	0.00%	196	0	0.00%	1.00 [0e+00-Inf]	1.000	1.000
EFNA3	101	0	0.00%	196	0	0.00%	1.00 [0e+00-Inf]	1.000	1.000
EFNA4	101	0	0.00%	196	0	0.00%	1.00 [0e+00-Inf]	1.000	1.000
EFR3A	101	0	0.00%	196	0	0.00%	1.00 [0e+00-Inf]	1.000	1.000
EGLN1	101	0	0.00%	196	0	0.00%	1.00 [0e+00-Inf]	1.000	1.000
EGLN3	101	0	0.00%	196	0	0.00%	1.00 [0e+00-Inf]	1.000	1.000
EHHADH	101	0	0.00%	196	0	0.00%	1.00 [0e+00-Inf]	1.000	1.000
EIF1AD	101	0	0.00%	196	0	0.00%	1.00 [0e+00-Inf]	1.000	1.000
EIF1AY	101	64	63.37%	196	116	59.18%	0.84 [0.55-1.27]	0.485	1.000
EIF2B5	101	0	0.00%	196	0	0.00%	1.00 [0e+00-Inf]	1.000	1.000
AGO2	101	0	0.00%	196	0	0.00%	1.00 [0e+00-Inf]	1.000	1.000
EIF3CL	101	0	0.00%	196	0	0.00%	1.00 [0e+00-Inf]	1.000	1.000
EIF3C	101	0	0.00%	196	0	0.00%	1.00 [0e+00-Inf]	1.000	1.000
EIF3E	101	0	0.00%	196	0	0.00%	1.00 [0e+00-Inf]	1.000	1.000
EIF3H	101	0	0.00%	196	0	0.00%	1.00 [0e+00-Inf]	1.000	1.000
EIF4A3	101	0	0.00%	196	0	0.00%	1.00 [0e+00-Inf]	1.000	1.000
EIF4EBP1	101	0	0.00%	196	0	0.00%	1.00 [0e+00-Inf]	1.000	1.000
EIF4G1	101	0	0.00%	196	0	0.00%	1.00 [0e+00-Inf]	1.000	1.000
EIF6	101	0	0.00%	196	0	0.00%	1.00 [0e+00-Inf]	1.000	1.000
ELF3	101	0	0.00%	196	0	0.00%	1.00 [0e+00-Inf]	1.000	1.000
ELK4	101	0	0.00%	196	0	0.00%	1.00 [0e+00-Inf]	1.000	1.000
ELMO2	101	0	0.00%	196	0	0.00%	1.00 [0e+00-Inf]	1.000	1.000
EME1	101	0	0.00%	196	0	0.00%	1.00 [0e+00-Inf]	1.000	1.000
EME2	101	0	0.00%	196	0	0.00%	1.00 [0e+00-Inf]	1.000	1.000
EMILIN3	101	0	0.00%	196	0	0.00%	1.00 [0e+00-Inf]	1.000	1.000
EMP2	101	0	0.00%	196	0	0.00%	1.00 [0e+00-Inf]	1.000	1.000
ENAH	101	0	0.00%	196	0	0.00%	1.00 [0e+00-Inf]	1.000	1.000
ENGASE	101	0	0.00%	196	0	0.00%	1.00 [0e+00-Inf]	1.000	1.000
ENHO	101	0	0.00%	196	0	0.00%	1.00 [0e+00-Inf]	1.000	1.000
ENPP2	101	0	0.00%	196	0	0.00%	1.00 [0e+00-Inf]	1.000	1.000
ENPP7	101	0	0.00%	196	0	0.00%	1.00 [0e+00-Inf]	1.000	1.000
ENSA	101	0	0.00%	196	0	0.00%	1.00 [0e+00-Inf]	1.000	1.000
ENY2	101	0	0.00%	196	0	0.00%	1.00 [0e+00-Inf]	1.000	1.000
EPB41L1	101	0	0.00%	196	0	0.00%	1.00 [0e+00-Inf]	1.000	1.000
EPGN	101	0	0.00%	196	0	0.00%	1.00 [0e+00-Inf]	1.000	1.000
EPHB3	101	0	0.00%	196	0	0.00%	1.00 [0e+00-Inf]	1.000	1.000
EPHB6	101	0	0.00%	196	0	0.00%	1.00 [0e+00-Inf]	1.000	1.000
EPHX1	101	0	0.00%	196	0	0.00%	1.00 [0e+00-Inf]	1.000	1.000
EPN1	101	0	0.00%	196	0	0.00%	1.00 [0e+00-Inf]	1.000	1.000
EPN2	101	0	0.00%	196	0	0.00%	1.00 [0e+00-Inf]	1.000	1.000
EPN3	101	0	0.00%	196	0	0.00%	1.00 [0e+00-Inf]	1.000	1.000
EPPK1	101	0	0.00%	196	0	0.00%	1.00 [0e+00-Inf]	1.000	1.000
EPRS	101	0	0.00%	196	0	0.00%	1.00 [0e+00-Inf]	1.000	1.000
EPX	101	0	0.00%	196	0	0.00%	1.00 [0e+00-Inf]	1.000	1.000
ERAL1	101	0	0.00%	196	0	0.00%	1.00 [0e+00-Inf]	1.000	1.000
ERBB2	101	0	0.00%	196	0	0.00%	1.00 [0e+00-Inf]	1.000	1.000
ERCC4	101	0	0.00%	196	0	0.00%	1.00 [0e+00-Inf]	1.000	1.000
EREG	101	0	0.00%	196	0	0.00%	1.00 [0e+00-Inf]	1.000	1.000
ERI2	101	0	0.00%	196	0	0.00%	1.00 [0e+00-Inf]	1.000	1.000
ERLIN2	101	0	0.00%	196	0	0.00%	1.00 [0e+00-Inf]	1.000	1.000
ERN1	101	0	0.00%	196	0	0.00%	1.00 [0e+00-Inf]	1.000	1.000
ERN2	101	0	0.00%	196	0	0.00%	1.00 [0e+00-Inf]	1.000	1.000
ESRP1	101	0	0.00%	196	0	0.00%	1.00 [0e+00-Inf]	1.000	1.000
ESRRG	101	0	0.00%	196	0	0.00%	1.00 [0e+00-Inf]	1.000	1.000
ETNK2	101	0	0.00%	196	0	0.00%	1.00 [0e+00-Inf]	1.000	1.000
ETV3L	101	0	0.00%	196	0	0.00%	1.00 [0e+00-Inf]	1.000	1.000
ETV3	101	0	0.00%	196	0	0.00%	1.00 [0e+00-Inf]	1.000	1.000
EVPL	101	0	0.00%	196	0	0.00%	1.00 [0e+00-Inf]	1.000	1.000
EVX1	101	0	0.00%	196	0	0.00%	1.00 [0e+00-Inf]	1.000	1.000
EXO1	101	0	0.00%	196	0	0.00%	1.00 [0e+00-Inf]	1.000	1.000
EXOC7	101	0	0.00%	196	0	0.00%	1.00 [0e+00-Inf]	1.000	1.000
EXOC8	101	0	0.00%	196	0	0.00%	1.00 [0e+00-Inf]	1.000	1.000
EXOSC4	101	0	0.00%	196	0	0.00%	1.00 [0e+00-Inf]	1.000	1.000
EXT1	101	0	0.00%	196	0	0.00%	1.00 [0e+00-Inf]	1.000	1.000
EYA1	101	0	0.00%	196	0	0.00%	1.00 [0e+00-Inf]	1.000	1.000
EYA2	101	0	0.00%	196	0	0.00%	1.00 [0e+00-Inf]	1.000	1.000
F11R	101	0	0.00%	196	0	0.00%	1.00 [0e+00-Inf]	1.000	1.000
F13B	101	0	0.00%	196	0	0.00%	1.00 [0e+00-Inf]	1.000	1.000
F5	101	0	0.00%	196	0	0.00%	1.00 [0e+00-Inf]	1.000	1.000
FABP12	101	0	0.00%	196	0	0.00%	1.00 [0e+00-Inf]	1.000	1.000
FABP4	101	0	0.00%	196	0	0.00%	1.00 [0e+00-Inf]	1.000	1.000

Symbol	CINSARC Low			CINSARC High			CINSARC High vs. Low		
	N tested	N altered	Fq alteration	N tested	N altered	Fq alteration	OR [95%CI]	p-value*	q-value
FABP5	101	0	0.00%	196	0	0.00%	1.00 [0e+00-Inf]	1.000	1.000
FABP9	101	0	0.00%	196	0	0.00%	1.00 [0e+00-Inf]	1.000	1.000
FADD	101	0	0.00%	196	0	0.00%	1.00 [0e+00-Inf]	1.000	1.000
FADS6	101	0	0.00%	196	0	0.00%	1.00 [0e+00-Inf]	1.000	1.000
FAHD1	101	0	0.00%	196	0	0.00%	1.00 [0e+00-Inf]	1.000	1.000
UBALD1	101	0	0.00%	196	0	0.00%	1.00 [0e+00-Inf]	1.000	1.000
UBALD2	101	0	0.00%	196	0	0.00%	1.00 [0e+00-Inf]	1.000	1.000
FAM104A	101	0	0.00%	196	0	0.00%	1.00 [0e+00-Inf]	1.000	1.000
FAM106A	101	1	0.99%	196	1	0.51%	0.51 [0.05-5.30]	0.638	1.000
ABHD17C	101	0	0.00%	196	0	0.00%	1.00 [0e+00-Inf]	1.000	1.000
FAM110B	101	0	0.00%	196	0	0.00%	1.00 [0e+00-Inf]	1.000	1.000
FAM117A	101	0	0.00%	196	0	0.00%	1.00 [0e+00-Inf]	1.000	1.000
FAM126A	101	0	0.00%	196	0	0.00%	1.00 [0e+00-Inf]	1.000	1.000
FAM129A	101	0	0.00%	196	0	0.00%	1.00 [0e+00-Inf]	1.000	1.000
FAM131A	101	0	0.00%	196	0	0.00%	1.00 [0e+00-Inf]	1.000	1.000
FAM135B	101	0	0.00%	196	0	0.00%	1.00 [0e+00-Inf]	1.000	1.000
FAM149B1	101	0	0.00%	196	0	0.00%	1.00 [0e+00-Inf]	1.000	1.000
FAM163A	101	0	0.00%	196	0	0.00%	1.00 [0e+00-Inf]	1.000	1.000
ZC2HC1A	101	0	0.00%	196	0	0.00%	1.00 [0e+00-Inf]	1.000	1.000
FAM168A	101	0	0.00%	196	0	0.00%	1.00 [0e+00-Inf]	1.000	1.000
FAM169B	101	0	0.00%	196	0	0.00%	1.00 [0e+00-Inf]	1.000	1.000
FAM173A	101	0	0.00%	196	0	0.00%	1.00 [0e+00-Inf]	1.000	1.000
FAM174B	101	0	0.00%	196	0	0.00%	1.00 [0e+00-Inf]	1.000	1.000
FAM177A1	101	0	0.00%	196	0	0.00%	1.00 [0e+00-Inf]	1.000	1.000
FAM177B	101	0	0.00%	196	0	0.00%	1.00 [0e+00-Inf]	1.000	1.000
FAM181B	101	0	0.00%	196	0	0.00%	1.00 [0e+00-Inf]	1.000	1.000
FAM189B	101	0	0.00%	196	0	0.00%	1.00 [0e+00-Inf]	1.000	1.000
TVP23A	101	0	0.00%	196	0	0.00%	1.00 [0e+00-Inf]	1.000	1.000
FAM197Y2	101	64	63.37%	196	116	59.18%	0.84 [0.55-1.27]	0.485	1.000
FAM20A	101	0	0.00%	196	0	0.00%	1.00 [0e+00-Inf]	1.000	1.000
FAM20B	101	0	0.00%	196	0	0.00%	1.00 [0e+00-Inf]	1.000	1.000
COX20	101	0	0.00%	196	0	0.00%	1.00 [0e+00-Inf]	1.000	1.000
FAM41AY1	101	65	64.36%	196	117	59.69%	0.82 [0.54-1.25]	0.435	1.000
FAM43A	101	0	0.00%	196	0	0.00%	1.00 [0e+00-Inf]	1.000	1.000
FAM47E	101	0	0.00%	196	0	0.00%	1.00 [0e+00-Inf]	1.000	1.000
FAM49B	101	0	0.00%	196	0	0.00%	1.00 [0e+00-Inf]	1.000	1.000
FAM57B	101	0	0.00%	196	0	0.00%	1.00 [0e+00-Inf]	1.000	1.000
BRINP2	101	0	0.00%	196	0	0.00%	1.00 [0e+00-Inf]	1.000	1.000
BRINP3	101	0	0.00%	196	0	0.00%	1.00 [0e+00-Inf]	1.000	1.000
FAM66D	101	1	0.99%	196	1	0.51%	0.51 [0.05-5.30]	0.638	1.000
FAM71A	101	0	0.00%	196	0	0.00%	1.00 [0e+00-Inf]	1.000	1.000
FAM71E2	101	0	0.00%	196	0	0.00%	1.00 [0e+00-Inf]	1.000	1.000
FAM72A	101	0	0.00%	196	0	0.00%	1.00 [0e+00-Inf]	1.000	1.000
SPATA31A6	101	0	0.00%	196	0	0.00%	1.00 [0e+00-Inf]	1.000	1.000
FAM78B	101	0	0.00%	196	0	0.00%	1.00 [0e+00-Inf]	1.000	1.000
RMDN1	101	0	0.00%	196	0	0.00%	1.00 [0e+00-Inf]	1.000	1.000
FAM83A	101	0	0.00%	196	0	0.00%	1.00 [0e+00-Inf]	1.000	1.000
FAM83C	101	0	0.00%	196	0	0.00%	1.00 [0e+00-Inf]	1.000	1.000
FAM83D	101	0	0.00%	196	0	0.00%	1.00 [0e+00-Inf]	1.000	1.000
FAM83H	101	0	0.00%	196	0	0.00%	1.00 [0e+00-Inf]	1.000	1.000
FAM86C1	101	0	0.00%	196	0	0.00%	1.00 [0e+00-Inf]	1.000	1.000
FAM89A	101	0	0.00%	196	0	0.00%	1.00 [0e+00-Inf]	1.000	1.000
FAM91A1	101	0	0.00%	196	0	0.00%	1.00 [0e+00-Inf]	1.000	1.000
FANCF	101	0	0.00%	196	0	0.00%	1.00 [0e+00-Inf]	1.000	1.000
FANCI	101	0	0.00%	196	0	0.00%	1.00 [0e+00-Inf]	1.000	1.000
FASLG	101	0	0.00%	196	0	0.00%	1.00 [0e+00-Inf]	1.000	1.000
FASN	101	0	0.00%	196	0	0.00%	1.00 [0e+00-Inf]	1.000	1.000
FBF1	101	0	0.00%	196	0	0.00%	1.00 [0e+00-Inf]	1.000	1.000
FBRS	101	0	0.00%	196	0	0.00%	1.00 [0e+00-Inf]	1.000	1.000
FBXL16	101	0	0.00%	196	0	0.00%	1.00 [0e+00-Inf]	1.000	1.000
FBXL19	101	0	0.00%	196	0	0.00%	1.00 [0e+00-Inf]	1.000	1.000
FBXL20	101	0	0.00%	196	0	0.00%	1.00 [0e+00-Inf]	1.000	1.000
FBXL6	101	0	0.00%	196	0	0.00%	1.00 [0e+00-Inf]	1.000	1.000
FBXO28	101	0	0.00%	196	0	0.00%	1.00 [0e+00-Inf]	1.000	1.000
FBXO32	101	0	0.00%	196	0	0.00%	1.00 [0e+00-Inf]	1.000	1.000
FBXO3	101	0	0.00%	196	0	0.00%	1.00 [0e+00-Inf]	1.000	1.000
FBXO43	101	0	0.00%	196	0	0.00%	1.00 [0e+00-Inf]	1.000	1.000
FBXO45	101	0	0.00%	196	0	0.00%	1.00 [0e+00-Inf]	1.000	1.000
FBXO47	101	0	0.00%	196	0	0.00%	1.00 [0e+00-Inf]	1.000	1.000
FCAMR	101	0	0.00%	196	0	0.00%	1.00 [0e+00-Inf]	1.000	1.000
FCER1A	101	0	0.00%	196	0	0.00%	1.00 [0e+00-Inf]	1.000	1.000

Symbol	CINSARC Low			CINSARC High			CINSARC High vs. Low		
	N tested	N altered	Fq alteration	N tested	N altered	Fq alteration	OR [95%CI]	p-value*	q-value
FCER1G	101	0	0.00%	196	0	0.00%	1.00 [0e+00-Inf]	1.000	1.000
FCGR1A	101	0	0.00%	196	0	0.00%	1.00 [0e+00-Inf]	1.000	1.000
FCGR2A	101	0	0.00%	196	0	0.00%	1.00 [0e+00-Inf]	1.000	1.000
FCGR2B	101	0	0.00%	196	0	0.00%	1.00 [0e+00-Inf]	1.000	1.000
FCGR2C	101	0	0.00%	196	0	0.00%	1.00 [0e+00-Inf]	1.000	1.000
FCGR3A	101	0	0.00%	196	0	0.00%	1.00 [0e+00-Inf]	1.000	1.000
FCGR3B	101	0	0.00%	196	0	0.00%	1.00 [0e+00-Inf]	1.000	1.000
FCHO1	101	0	0.00%	196	0	0.00%	1.00 [0e+00-Inf]	1.000	1.000
FCHSD2	101	0	0.00%	196	0	0.00%	1.00 [0e+00-Inf]	1.000	1.000
FCRL1	101	0	0.00%	196	0	0.00%	1.00 [0e+00-Inf]	1.000	1.000
FCRL2	101	0	0.00%	196	0	0.00%	1.00 [0e+00-Inf]	1.000	1.000
FCRL3	101	0	0.00%	196	0	0.00%	1.00 [0e+00-Inf]	1.000	1.000
FCRL4	101	0	0.00%	196	0	0.00%	1.00 [0e+00-Inf]	1.000	1.000
FCRL5	101	0	0.00%	196	0	0.00%	1.00 [0e+00-Inf]	1.000	1.000
FCRL6	101	0	0.00%	196	0	0.00%	1.00 [0e+00-Inf]	1.000	1.000
FCRLA	101	0	0.00%	196	0	0.00%	1.00 [0e+00-Inf]	1.000	1.000
FCRLB	101	0	0.00%	196	0	0.00%	1.00 [0e+00-Inf]	1.000	1.000
FDPS	101	0	0.00%	196	0	0.00%	1.00 [0e+00-Inf]	1.000	1.000
FDXR	101	0	0.00%	196	0	0.00%	1.00 [0e+00-Inf]	1.000	1.000
FER1L6	101	0	0.00%	196	0	0.00%	1.00 [0e+00-Inf]	1.000	1.000
FES	101	0	0.00%	196	0	0.00%	1.00 [0e+00-Inf]	1.000	1.000
FGD5	101	0	0.00%	196	0	0.00%	1.00 [0e+00-Inf]	1.000	1.000
FGD6	101	0	0.00%	196	0	0.00%	1.00 [0e+00-Inf]	1.000	1.000
FGF12	101	0	0.00%	196	0	0.00%	1.00 [0e+00-Inf]	1.000	1.000
FGF19	101	0	0.00%	196	0	0.00%	1.00 [0e+00-Inf]	1.000	1.000
FGF3	101	0	0.00%	196	0	0.00%	1.00 [0e+00-Inf]	1.000	1.000
FGF4	101	0	0.00%	196	0	0.00%	1.00 [0e+00-Inf]	1.000	1.000
FGFR1	101	0	0.00%	196	0	0.00%	1.00 [0e+00-Inf]	1.000	1.000
FH	101	0	0.00%	196	0	0.00%	1.00 [0e+00-Inf]	1.000	1.000
FITM2	101	0	0.00%	196	0	0.00%	1.00 [0e+00-Inf]	1.000	1.000
FIZ1	101	0	0.00%	196	0	0.00%	1.00 [0e+00-Inf]	1.000	1.000
FKBP14	101	0	0.00%	196	0	0.00%	1.00 [0e+00-Inf]	1.000	1.000
FKBP9	101	0	0.00%	196	0	0.00%	1.00 [0e+00-Inf]	1.000	1.000
FLAD1	101	0	0.00%	196	0	0.00%	1.00 [0e+00-Inf]	1.000	1.000
FLG2	101	0	0.00%	196	0	0.00%	1.00 [0e+00-Inf]	1.000	1.000
FLG	101	0	0.00%	196	0	0.00%	1.00 [0e+00-Inf]	1.000	1.000
FLJ16779	101	0	0.00%	196	0	0.00%	1.00 [0e+00-Inf]	1.000	1.000
FLJ42393	101	0	0.00%	196	0	0.00%	1.00 [0e+00-Inf]	1.000	1.000
FLJ42627	101	0	0.00%	196	0	0.00%	1.00 [0e+00-Inf]	1.000	1.000
FLOT2	101	0	0.00%	196	0	0.00%	1.00 [0e+00-Inf]	1.000	1.000
FLT1	101	0	0.00%	196	0	0.00%	1.00 [0e+00-Inf]	1.000	1.000
FLVCR1	101	0	0.00%	196	0	0.00%	1.00 [0e+00-Inf]	1.000	1.000
FLYWCH1	101	0	0.00%	196	0	0.00%	1.00 [0e+00-Inf]	1.000	1.000
FLYWCH2	101	0	0.00%	196	0	0.00%	1.00 [0e+00-Inf]	1.000	1.000
FMN2	101	0	0.00%	196	0	0.00%	1.00 [0e+00-Inf]	1.000	1.000
FMO1	101	0	0.00%	196	0	0.00%	1.00 [0e+00-Inf]	1.000	1.000
FMO2	101	0	0.00%	196	0	0.00%	1.00 [0e+00-Inf]	1.000	1.000
FMO3	101	0	0.00%	196	0	0.00%	1.00 [0e+00-Inf]	1.000	1.000
FMO4	101	0	0.00%	196	0	0.00%	1.00 [0e+00-Inf]	1.000	1.000
FMO5	101	0	0.00%	196	0	0.00%	1.00 [0e+00-Inf]	1.000	1.000
FMO6P	101	0	0.00%	196	0	0.00%	1.00 [0e+00-Inf]	1.000	1.000
FMO9P	101	0	0.00%	196	0	0.00%	1.00 [0e+00-Inf]	1.000	1.000
FMOD	101	0	0.00%	196	0	0.00%	1.00 [0e+00-Inf]	1.000	1.000
FN3KRP	101	0	0.00%	196	0	0.00%	1.00 [0e+00-Inf]	1.000	1.000
FN3K	101	0	0.00%	196	0	0.00%	1.00 [0e+00-Inf]	1.000	1.000
FNDC8	101	0	0.00%	196	0	0.00%	1.00 [0e+00-Inf]	1.000	1.000
FNTA	101	0	0.00%	196	0	0.00%	1.00 [0e+00-Inf]	1.000	1.000
FOLR1	101	0	0.00%	196	0	0.00%	1.00 [0e+00-Inf]	1.000	1.000
FOLR2	101	0	0.00%	196	0	0.00%	1.00 [0e+00-Inf]	1.000	1.000
FOLR3	101	0	0.00%	196	0	0.00%	1.00 [0e+00-Inf]	1.000	1.000
FOXA1	101	0	0.00%	196	0	0.00%	1.00 [0e+00-Inf]	1.000	1.000
FOXH1	101	0	0.00%	196	0	0.00%	1.00 [0e+00-Inf]	1.000	1.000
FOXJ1	101	0	0.00%	196	0	0.00%	1.00 [0e+00-Inf]	1.000	1.000
FOXK2	101	0	0.00%	196	0	0.00%	1.00 [0e+00-Inf]	1.000	1.000
FOXN1	101	0	0.00%	196	0	0.00%	1.00 [0e+00-Inf]	1.000	1.000
FPR1	101	0	0.00%	196	0	0.00%	1.00 [0e+00-Inf]	1.000	1.000
FPR2	101	0	0.00%	196	0	0.00%	1.00 [0e+00-Inf]	1.000	1.000
FPR3	101	0	0.00%	196	0	0.00%	1.00 [0e+00-Inf]	1.000	1.000
FRS2	101	0	0.00%	196	0	0.00%	1.00 [0e+00-Inf]	1.000	1.000
FSCN2	101	0	0.00%	196	0	0.00%	1.00 [0e+00-Inf]	1.000	1.000
FSD2	101	0	0.00%	196	0	0.00%	1.00 [0e+00-Inf]	1.000	1.000

Symbol	CINSARC Low			CINSARC High			CINSARC High vs. Low		
	N tested	N altered	Fq alteration	N tested	N altered	Fq alteration	OR [95%CI]	p-value*	q-value
FTSJ3	101	0	0.00%	196	0	0.00%	1.00 [0e+00-Inf]	1.000	1.000
FURIN	101	0	0.00%	196	0	0.00%	1.00 [0e+00-Inf]	1.000	1.000
FUS	101	0	0.00%	196	0	0.00%	1.00 [0e+00-Inf]	1.000	1.000
FUT10	101	0	0.00%	196	0	0.00%	1.00 [0e+00-Inf]	1.000	1.000
FUT11	101	0	0.00%	196	0	0.00%	1.00 [0e+00-Inf]	1.000	1.000
FUT3	101	0	0.00%	196	0	0.00%	1.00 [0e+00-Inf]	1.000	1.000
FXR1	101	0	0.00%	196	0	0.00%	1.00 [0e+00-Inf]	1.000	1.000
FYTTD1	101	0	0.00%	196	0	0.00%	1.00 [0e+00-Inf]	1.000	1.000
FZD4	101	0	0.00%	196	0	0.00%	1.00 [0e+00-Inf]	1.000	1.000
FZD6	101	0	0.00%	196	0	0.00%	1.00 [0e+00-Inf]	1.000	1.000
G0S2	101	0	0.00%	196	0	0.00%	1.00 [0e+00-Inf]	1.000	1.000
G2E3	101	0	0.00%	196	0	0.00%	1.00 [0e+00-Inf]	1.000	1.000
G3BP2	101	0	0.00%	196	0	0.00%	1.00 [0e+00-Inf]	1.000	1.000
GAA	101	0	0.00%	196	0	0.00%	1.00 [0e+00-Inf]	1.000	1.000
GAB2	101	0	0.00%	196	0	0.00%	1.00 [0e+00-Inf]	1.000	1.000
GABARAPL3	101	0	0.00%	196	0	0.00%	1.00 [0e+00-Inf]	1.000	1.000
GABPB2	101	0	0.00%	196	0	0.00%	1.00 [0e+00-Inf]	1.000	1.000
GAL3ST3	101	0	0.00%	196	0	0.00%	1.00 [0e+00-Inf]	1.000	1.000
GALK1	101	0	0.00%	196	0	0.00%	1.00 [0e+00-Inf]	1.000	1.000
GALNT2	101	0	0.00%	196	0	0.00%	1.00 [0e+00-Inf]	1.000	1.000
GALR2	101	0	0.00%	196	0	0.00%	1.00 [0e+00-Inf]	1.000	1.000
GALT	101	0	0.00%	196	0	0.00%	1.00 [0e+00-Inf]	1.000	1.000
GAL	101	0	0.00%	196	0	0.00%	1.00 [0e+00-Inf]	1.000	1.000
GARS	101	0	0.00%	196	0	0.00%	1.00 [0e+00-Inf]	1.000	1.000
GAS2L2	101	0	0.00%	196	0	0.00%	1.00 [0e+00-Inf]	1.000	1.000
GAS2	101	0	0.00%	196	0	0.00%	1.00 [0e+00-Inf]	1.000	1.000
GAS5	101	0	0.00%	196	0	0.00%	1.00 [0e+00-Inf]	1.000	1.000
GATA5	101	0	0.00%	196	0	0.00%	1.00 [0e+00-Inf]	1.000	1.000
GATAD2B	101	0	0.00%	196	0	0.00%	1.00 [0e+00-Inf]	1.000	1.000
GBAP1	101	0	0.00%	196	0	0.00%	1.00 [0e+00-Inf]	1.000	1.000
GBA	101	0	0.00%	196	0	0.00%	1.00 [0e+00-Inf]	1.000	1.000
GCGR	101	0	0.00%	196	0	0.00%	1.00 [0e+00-Inf]	1.000	1.000
GCK	101	0	0.00%	196	0	0.00%	1.00 [0e+00-Inf]	1.000	1.000
GCNT7	101	0	0.00%	196	0	0.00%	1.00 [0e+00-Inf]	1.000	1.000
GDAP1L1	101	0	0.00%	196	0	0.00%	1.00 [0e+00-Inf]	1.000	1.000
GDAP1	101	0	0.00%	196	0	0.00%	1.00 [0e+00-Inf]	1.000	1.000
GDE1	101	0	0.00%	196	0	0.00%	1.00 [0e+00-Inf]	1.000	1.000
GDF5	101	0	0.00%	196	0	0.00%	1.00 [0e+00-Inf]	1.000	1.000
GDF6	101	0	0.00%	196	0	0.00%	1.00 [0e+00-Inf]	1.000	1.000
GDPD1	101	0	0.00%	196	0	0.00%	1.00 [0e+00-Inf]	1.000	1.000
GDPD3	101	0	0.00%	196	0	0.00%	1.00 [0e+00-Inf]	1.000	1.000
GDPD4	101	0	0.00%	196	0	0.00%	1.00 [0e+00-Inf]	1.000	1.000
GDPD5	101	0	0.00%	196	0	0.00%	1.00 [0e+00-Inf]	1.000	1.000
GEM	101	0	0.00%	196	0	0.00%	1.00 [0e+00-Inf]	1.000	1.000
GFER	101	0	0.00%	196	0	0.00%	1.00 [0e+00-Inf]	1.000	1.000
GGA2	101	0	0.00%	196	0	0.00%	1.00 [0e+00-Inf]	1.000	1.000
GGA3	101	0	0.00%	196	0	0.00%	1.00 [0e+00-Inf]	1.000	1.000
GGCT	101	0	0.00%	196	0	0.00%	1.00 [0e+00-Inf]	1.000	1.000
GGH	101	0	0.00%	196	0	0.00%	1.00 [0e+00-Inf]	1.000	1.000
GGNBP2	101	0	0.00%	196	0	0.00%	1.00 [0e+00-Inf]	1.000	1.000
GGPS1	101	0	0.00%	196	0	0.00%	1.00 [0e+00-Inf]	1.000	1.000
GGT7	101	0	0.00%	196	0	0.00%	1.00 [0e+00-Inf]	1.000	1.000
GH1	101	0	0.00%	196	0	0.00%	1.00 [0e+00-Inf]	1.000	1.000
GH2	101	0	0.00%	196	0	0.00%	1.00 [0e+00-Inf]	1.000	1.000
GHRHR	101	0	0.00%	196	0	0.00%	1.00 [0e+00-Inf]	1.000	1.000
GHRH	101	0	0.00%	196	0	0.00%	1.00 [0e+00-Inf]	1.000	1.000
GINS4	101	0	0.00%	196	0	0.00%	1.00 [0e+00-Inf]	1.000	1.000
GIP	101	0	0.00%	196	0	0.00%	1.00 [0e+00-Inf]	1.000	1.000
GIT1	101	0	0.00%	196	0	0.00%	1.00 [0e+00-Inf]	1.000	1.000
SLX1B	101	0	0.00%	196	0	0.00%	1.00 [0e+00-Inf]	1.000	1.000
GJA5	101	0	0.00%	196	0	0.00%	1.00 [0e+00-Inf]	1.000	1.000
GJA8	101	0	0.00%	196	0	0.00%	1.00 [0e+00-Inf]	1.000	1.000
GJC2	101	0	0.00%	196	0	0.00%	1.00 [0e+00-Inf]	1.000	1.000
GJD3	101	0	0.00%	196	0	0.00%	1.00 [0e+00-Inf]	1.000	1.000
GLI4	101	0	0.00%	196	0	0.00%	1.00 [0e+00-Inf]	1.000	1.000
GLIPR1L1	101	0	0.00%	196	0	0.00%	1.00 [0e+00-Inf]	1.000	1.000
GLIPR1L2	101	0	0.00%	196	0	0.00%	1.00 [0e+00-Inf]	1.000	1.000
GLIPR1	101	0	0.00%	196	0	0.00%	1.00 [0e+00-Inf]	1.000	1.000
GLIS2	101	0	0.00%	196	0	0.00%	1.00 [0e+00-Inf]	1.000	1.000
GLRX2	101	0	0.00%	196	0	0.00%	1.00 [0e+00-Inf]	1.000	1.000
COLGALT2	101	0	0.00%	196	0	0.00%	1.00 [0e+00-Inf]	1.000	1.000

Symbol	CINSARC Low			CINSARC High			CINSARC High vs. Low		
	N tested	N altered	Fq alteration	N tested	N altered	Fq alteration	OR [95%CI]	p-value*	q-value
GLUL	101	0	0.00%	196	0	0.00%	1.00 [0e+00-Inf]	1.000	1.000
GLYR1	101	0	0.00%	196	0	0.00%	1.00 [0e+00-Inf]	1.000	1.000
GMEB2	101	0	0.00%	196	0	0.00%	1.00 [0e+00-Inf]	1.000	1.000
GML	101	0	0.00%	196	0	0.00%	1.00 [0e+00-Inf]	1.000	1.000
GNA13	101	0	0.00%	196	0	0.00%	1.00 [0e+00-Inf]	1.000	1.000
GNAS-AS1	101	0	0.00%	196	0	0.00%	1.00 [0e+00-Inf]	1.000	1.000
GNAS	101	0	0.00%	196	0	0.00%	1.00 [0e+00-Inf]	1.000	1.000
GNB4	101	0	0.00%	196	0	0.00%	1.00 [0e+00-Inf]	1.000	1.000
GNG13	101	0	0.00%	196	0	0.00%	1.00 [0e+00-Inf]	1.000	1.000
GNG4	101	0	0.00%	196	0	0.00%	1.00 [0e+00-Inf]	1.000	1.000
GNGT2	101	0	0.00%	196	0	0.00%	1.00 [0e+00-Inf]	1.000	1.000
GNPAT	101	0	0.00%	196	0	0.00%	1.00 [0e+00-Inf]	1.000	1.000
GNPTG	101	0	0.00%	196	0	0.00%	1.00 [0e+00-Inf]	1.000	1.000
GNRHR2	101	0	0.00%	196	0	0.00%	1.00 [0e+00-Inf]	1.000	1.000
GNS	101	0	0.00%	196	0	0.00%	1.00 [0e+00-Inf]	1.000	1.000
GOLGA2P3Y	101	64	63.37%	196	116	59.18%	0.84 [0.55-1.27]	0.485	1.000
GOLGA6L5P	101	0	0.00%	196	0	0.00%	1.00 [0e+00-Inf]	1.000	1.000
GOLGA6L6	101	0	0.00%	196	0	0.00%	1.00 [0e+00-Inf]	1.000	1.000
GOLGA7	101	0	0.00%	196	0	0.00%	1.00 [0e+00-Inf]	1.000	1.000
GOLGA8CP	101	0	0.00%	196	0	0.00%	1.00 [0e+00-Inf]	1.000	1.000
GOLPH3L	101	0	0.00%	196	0	0.00%	1.00 [0e+00-Inf]	1.000	1.000
GOLT1A	101	0	0.00%	196	0	0.00%	1.00 [0e+00-Inf]	1.000	1.000
GON4L	101	0	0.00%	196	0	0.00%	1.00 [0e+00-Inf]	1.000	1.000
GORAB	101	0	0.00%	196	0	0.00%	1.00 [0e+00-Inf]	1.000	1.000
GOSR1	101	0	0.00%	196	0	0.00%	1.00 [0e+00-Inf]	1.000	1.000
GOSR2	101	0	0.00%	196	0	0.00%	1.00 [0e+00-Inf]	1.000	1.000
GOT1L1	101	0	0.00%	196	0	0.00%	1.00 [0e+00-Inf]	1.000	1.000
GP2	101	0	0.00%	196	0	0.00%	1.00 [0e+00-Inf]	1.000	1.000
GP5	101	0	0.00%	196	0	0.00%	1.00 [0e+00-Inf]	1.000	1.000
GPA33	101	0	0.00%	196	0	0.00%	1.00 [0e+00-Inf]	1.000	1.000
GPAA1	101	0	0.00%	196	0	0.00%	1.00 [0e+00-Inf]	1.000	1.000
GPATCH2	101	0	0.00%	196	0	0.00%	1.00 [0e+00-Inf]	1.000	1.000
GPATCH4	101	0	0.00%	196	0	0.00%	1.00 [0e+00-Inf]	1.000	1.000
GPIHBP1	101	0	0.00%	196	0	0.00%	1.00 [0e+00-Inf]	1.000	1.000
GPNMB	101	0	0.00%	196	0	0.00%	1.00 [0e+00-Inf]	1.000	1.000
GPR137B	101	0	0.00%	196	0	0.00%	1.00 [0e+00-Inf]	1.000	1.000
GPR139	101	0	0.00%	196	0	0.00%	1.00 [0e+00-Inf]	1.000	1.000
GPR142	101	0	0.00%	196	0	0.00%	1.00 [0e+00-Inf]	1.000	1.000
GPR152	101	0	0.00%	196	0	0.00%	1.00 [0e+00-Inf]	1.000	1.000
GPR161	101	0	0.00%	196	0	0.00%	1.00 [0e+00-Inf]	1.000	1.000
GPR171	101	0	0.00%	196	0	0.00%	1.00 [0e+00-Inf]	1.000	1.000
SLC52A2	101	0	0.00%	196	0	0.00%	1.00 [0e+00-Inf]	1.000	1.000
GPR179	101	0	0.00%	196	0	0.00%	1.00 [0e+00-Inf]	1.000	1.000
GPR20	101	0	0.00%	196	0	0.00%	1.00 [0e+00-Inf]	1.000	1.000
GPR25	101	0	0.00%	196	0	0.00%	1.00 [0e+00-Inf]	1.000	1.000
GPR37L1	101	0	0.00%	196	0	0.00%	1.00 [0e+00-Inf]	1.000	1.000
GPR52	101	0	0.00%	196	0	0.00%	1.00 [0e+00-Inf]	1.000	1.000
GPR87	101	0	0.00%	196	0	0.00%	1.00 [0e+00-Inf]	1.000	1.000
GPR89A	101	0	0.00%	196	0	0.00%	1.00 [0e+00-Inf]	1.000	1.000
GPR89B	101	0	0.00%	196	0	0.00%	1.00 [0e+00-Inf]	1.000	1.000
GPRC5B	101	0	0.00%	196	0	0.00%	1.00 [0e+00-Inf]	1.000	1.000
GPRC5C	101	0	0.00%	196	0	0.00%	1.00 [0e+00-Inf]	1.000	1.000
GPS1	101	0	0.00%	196	0	0.00%	1.00 [0e+00-Inf]	1.000	1.000
GPT2	101	0	0.00%	196	0	0.00%	1.00 [0e+00-Inf]	1.000	1.000
GPT	101	0	0.00%	196	0	0.00%	1.00 [0e+00-Inf]	1.000	1.000
GRAPL	101	0	0.00%	196	1	0.51%	3.2e+07 [0e+00-Inf]	0.997	1.000
GRB2	101	0	0.00%	196	0	0.00%	1.00 [0e+00-Inf]	1.000	1.000
GRB7	101	0	0.00%	196	0	0.00%	1.00 [0e+00-Inf]	1.000	1.000
GREM2	101	0	0.00%	196	0	0.00%	1.00 [0e+00-Inf]	1.000	1.000
GRHL2	101	0	0.00%	196	0	0.00%	1.00 [0e+00-Inf]	1.000	1.000
GRIN2A	101	0	0.00%	196	0	0.00%	1.00 [0e+00-Inf]	1.000	1.000
GRIN2C	101	0	0.00%	196	0	0.00%	1.00 [0e+00-Inf]	1.000	1.000
GRINA	101	0	0.00%	196	0	0.00%	1.00 [0e+00-Inf]	1.000	1.000
GRIP1	101	0	0.00%	196	0	0.00%	1.00 [0e+00-Inf]	1.000	1.000
GRM5	101	0	0.00%	196	0	0.00%	1.00 [0e+00-Inf]	1.000	1.000
GRP	101	0	0.00%	196	0	0.00%	1.00 [0e+00-Inf]	1.000	1.000
GSDMA	101	0	0.00%	196	0	0.00%	1.00 [0e+00-Inf]	1.000	1.000
GSDMB	101	0	0.00%	196	0	0.00%	1.00 [0e+00-Inf]	1.000	1.000
GSDMC	101	1	0.99%	196	0	0.00%	1.6e-08 [0e+00-Inf]	0.996	1.000
GSDMD	101	0	0.00%	196	0	0.00%	1.00 [0e+00-Inf]	1.000	1.000
GSG1L	101	0	0.00%	196	0	0.00%	1.00 [0e+00-Inf]	1.000	1.000

Symbol	CINSARC Low			CINSARC High			CINSARC High vs. Low		
	N tested	N altered	Fq alteration	N tested	N altered	Fq alteration	OR [95%CI]	p-value*	q-value
GSPT1	101	0	0.00%	196	0	0.00%	1.00 [0e+00-Inf]	1.000	1.000
GSR	101	0	0.00%	196	0	0.00%	1.00 [0e+00-Inf]	1.000	1.000
GSS	101	0	0.00%	196	0	0.00%	1.00 [0e+00-Inf]	1.000	1.000
GSTM1	101	1	0.99%	196	0	0.00%	1.6e-08 [0e+00-Inf]	0.996	1.000
GSTM2	101	0	0.00%	196	0	0.00%	1.00 [0e+00-Inf]	1.000	1.000
GSTP1	101	0	0.00%	196	0	0.00%	1.00 [0e+00-Inf]	1.000	1.000
GSTT1	101	4	3.96%	196	4	2.04%	0.51 [0.16-1.65]	0.342	1.000
GTF2E2	101	0	0.00%	196	0	0.00%	1.00 [0e+00-Inf]	1.000	1.000
GTF2H2	101	0	0.00%	196	0	0.00%	1.00 [0e+00-Inf]	1.000	1.000
GTF3C1	101	0	0.00%	196	0	0.00%	1.00 [0e+00-Inf]	1.000	1.000
MTG2	101	0	0.00%	196	0	0.00%	1.00 [0e+00-Inf]	1.000	1.000
GTSF1L	101	0	0.00%	196	0	0.00%	1.00 [0e+00-Inf]	1.000	1.000
GUCY2EP	101	0	0.00%	196	0	0.00%	1.00 [0e+00-Inf]	1.000	1.000
GUK1	101	0	0.00%	196	0	0.00%	1.00 [0e+00-Inf]	1.000	1.000
H3F3A	101	0	0.00%	196	0	0.00%	1.00 [0e+00-Inf]	1.000	1.000
H3F3B	101	0	0.00%	196	0	0.00%	1.00 [0e+00-Inf]	1.000	1.000
HACE1	101	0	0.00%	196	0	0.00%	1.00 [0e+00-Inf]	1.000	1.000
HAGHL	101	0	0.00%	196	0	0.00%	1.00 [0e+00-Inf]	1.000	1.000
HAGH	101	0	0.00%	196	0	0.00%	1.00 [0e+00-Inf]	1.000	1.000
HAPLN2	101	0	0.00%	196	0	0.00%	1.00 [0e+00-Inf]	1.000	1.000
HAPLN3	101	0	0.00%	196	0	0.00%	1.00 [0e+00-Inf]	1.000	1.000
HAR1A	101	0	0.00%	196	0	0.00%	1.00 [0e+00-Inf]	1.000	1.000
HAR1B	101	0	0.00%	196	0	0.00%	1.00 [0e+00-Inf]	1.000	1.000
HAS1	101	0	0.00%	196	0	0.00%	1.00 [0e+00-Inf]	1.000	1.000
HAS2-AS1	101	0	0.00%	196	0	0.00%	1.00 [0e+00-Inf]	1.000	1.000
HAS2	101	0	0.00%	196	0	0.00%	1.00 [0e+00-Inf]	1.000	1.000
HAX1	101	0	0.00%	196	0	0.00%	1.00 [0e+00-Inf]	1.000	1.000
HBA1	101	0	0.00%	196	0	0.00%	1.00 [0e+00-Inf]	1.000	1.000
HBA2	101	0	0.00%	196	0	0.00%	1.00 [0e+00-Inf]	1.000	1.000
HBM	101	0	0.00%	196	0	0.00%	1.00 [0e+00-Inf]	1.000	1.000
HBQ1	101	0	0.00%	196	0	0.00%	1.00 [0e+00-Inf]	1.000	1.000
HBZ	101	0	0.00%	196	0	0.00%	1.00 [0e+00-Inf]	1.000	1.000
HCFC1R1	101	0	0.00%	196	0	0.00%	1.00 [0e+00-Inf]	1.000	1.000
HCN3	101	0	0.00%	196	0	0.00%	1.00 [0e+00-Inf]	1.000	1.000
HDDC3	101	0	0.00%	196	0	0.00%	1.00 [0e+00-Inf]	1.000	1.000
HDGF	101	0	0.00%	196	0	0.00%	1.00 [0e+00-Inf]	1.000	1.000
HEATR1	101	0	0.00%	196	0	0.00%	1.00 [0e+00-Inf]	1.000	1.000
HEATR4	101	0	0.00%	196	0	0.00%	1.00 [0e+00-Inf]	1.000	1.000
HEATR5A	101	0	0.00%	196	0	0.00%	1.00 [0e+00-Inf]	1.000	1.000
HEATR6	101	0	0.00%	196	0	0.00%	1.00 [0e+00-Inf]	1.000	1.000
MROH1	101	0	0.00%	196	0	0.00%	1.00 [0e+00-Inf]	1.000	1.000
HECTD1	101	0	0.00%	196	0	0.00%	1.00 [0e+00-Inf]	1.000	1.000
HELB	101	0	0.00%	196	0	0.00%	1.00 [0e+00-Inf]	1.000	1.000
HELZ	101	0	0.00%	196	0	0.00%	1.00 [0e+00-Inf]	1.000	1.000
HERC2P4	101	0	0.00%	196	0	0.00%	1.00 [0e+00-Inf]	1.000	1.000
HES1	101	0	0.00%	196	0	0.00%	1.00 [0e+00-Inf]	1.000	1.000
HEXA	101	0	0.00%	196	0	0.00%	1.00 [0e+00-Inf]	1.000	1.000
HEY1	101	0	0.00%	196	0	0.00%	1.00 [0e+00-Inf]	1.000	1.000
HGSNAT	101	0	0.00%	196	0	0.00%	1.00 [0e+00-Inf]	1.000	1.000
HGS	101	0	0.00%	196	0	0.00%	1.00 [0e+00-Inf]	1.000	1.000
HHAT	101	0	0.00%	196	0	0.00%	1.00 [0e+00-Inf]	1.000	1.000
HHIPL2	101	0	0.00%	196	0	0.00%	1.00 [0e+00-Inf]	1.000	1.000
HHLA1	101	0	0.00%	196	0	0.00%	1.00 [0e+00-Inf]	1.000	1.000
HIBADH	101	0	0.00%	196	0	0.00%	1.00 [0e+00-Inf]	1.000	1.000
HILS1	101	0	0.00%	196	0	0.00%	1.00 [0e+00-Inf]	1.000	1.000
HIRIP3	101	0	0.00%	196	0	0.00%	1.00 [0e+00-Inf]	1.000	1.000
HIST2H2AA3	101	0	0.00%	196	0	0.00%	1.00 [0e+00-Inf]	1.000	1.000
HIST2H2AB	101	0	0.00%	196	0	0.00%	1.00 [0e+00-Inf]	1.000	1.000
HIST2H2AC	101	0	0.00%	196	0	0.00%	1.00 [0e+00-Inf]	1.000	1.000
HIST2H2BE	101	0	0.00%	196	0	0.00%	1.00 [0e+00-Inf]	1.000	1.000
HIST2H2BF	101	0	0.00%	196	0	0.00%	1.00 [0e+00-Inf]	1.000	1.000
HIST2H3C	101	0	0.00%	196	0	0.00%	1.00 [0e+00-Inf]	1.000	1.000
HIST2H3D	101	0	0.00%	196	0	0.00%	1.00 [0e+00-Inf]	1.000	1.000
HIST2H4A	101	0	0.00%	196	0	0.00%	1.00 [0e+00-Inf]	1.000	1.000
HIST3H2A	101	0	0.00%	196	0	0.00%	1.00 [0e+00-Inf]	1.000	1.000
HIST3H2BB	101	0	0.00%	196	0	0.00%	1.00 [0e+00-Inf]	1.000	1.000
HIST3H3	101	0	0.00%	196	0	0.00%	1.00 [0e+00-Inf]	1.000	1.000
HLA-DRB1	101	0	0.00%	196	0	0.00%	1.00 [0e+00-Inf]	1.000	1.000
HLA-DRB5	101	0	0.00%	196	0	0.00%	1.00 [0e+00-Inf]	1.000	1.000
HLA-DRB6	101	0	0.00%	196	0	0.00%	1.00 [0e+00-Inf]	1.000	1.000
HLF	101	0	0.00%	196	0	0.00%	1.00 [0e+00-Inf]	1.000	1.000

Symbol	CINSARC Low			CINSARC High			CINSARC High vs. Low		
	N tested	N altered	Fq alteration	N tested	N altered	Fq alteration	OR [95%CI]	p-value*	q-value
HLX	101	0	0.00%	196	0	0.00%	1.00 [0e+00-Inf]	1.000	1.000
HMCN1	101	0	0.00%	196	0	0.00%	1.00 [0e+00-Inf]	1.000	1.000
HMGA2	101	0	0.00%	196	1	0.51%	3.2e+07 [0e+00-Inf]	0.997	1.000
HMGB1	101	0	0.00%	196	0	0.00%	1.00 [0e+00-Inf]	1.000	1.000
HMGB3P1	101	0	0.00%	196	0	0.00%	1.00 [0e+00-Inf]	1.000	1.000
HMOX2	101	0	0.00%	196	0	0.00%	1.00 [0e+00-Inf]	1.000	1.000
HMSD	101	0	0.00%	196	0	0.00%	1.00 [0e+00-Inf]	1.000	1.000
HNF1B	101	0	0.00%	196	0	0.00%	1.00 [0e+00-Inf]	1.000	1.000
HNF4A	101	0	0.00%	196	0	0.00%	1.00 [0e+00-Inf]	1.000	1.000
HNF4G	101	0	0.00%	196	0	0.00%	1.00 [0e+00-Inf]	1.000	1.000
HNRNPA2B1	101	0	0.00%	196	0	0.00%	1.00 [0e+00-Inf]	1.000	1.000
HNRNPU	101	0	0.00%	196	0	0.00%	1.00 [0e+00-Inf]	1.000	1.000
HOMER2	101	0	0.00%	196	0	0.00%	1.00 [0e+00-Inf]	1.000	1.000
HOOK3	101	0	0.00%	196	0	0.00%	1.00 [0e+00-Inf]	1.000	1.000
HORMAD1	101	0	0.00%	196	0	0.00%	1.00 [0e+00-Inf]	1.000	1.000
HOXA10	101	0	0.00%	196	0	0.00%	1.00 [0e+00-Inf]	1.000	1.000
HOXA11-AS	101	0	0.00%	196	0	0.00%	1.00 [0e+00-Inf]	1.000	1.000
HOXA11	101	0	0.00%	196	0	0.00%	1.00 [0e+00-Inf]	1.000	1.000
HOXA13	101	0	0.00%	196	0	0.00%	1.00 [0e+00-Inf]	1.000	1.000
HOXA1	101	0	0.00%	196	0	0.00%	1.00 [0e+00-Inf]	1.000	1.000
HOXA2	101	0	0.00%	196	0	0.00%	1.00 [0e+00-Inf]	1.000	1.000
HOXA3	101	0	0.00%	196	0	0.00%	1.00 [0e+00-Inf]	1.000	1.000
HOXA4	101	0	0.00%	196	0	0.00%	1.00 [0e+00-Inf]	1.000	1.000
HOXA5	101	0	0.00%	196	0	0.00%	1.00 [0e+00-Inf]	1.000	1.000
HOXA6	101	0	0.00%	196	0	0.00%	1.00 [0e+00-Inf]	1.000	1.000
HOXA7	101	0	0.00%	196	0	0.00%	1.00 [0e+00-Inf]	1.000	1.000
HOXA9	101	0	0.00%	196	0	0.00%	1.00 [0e+00-Inf]	1.000	1.000
HOXB13	101	0	0.00%	196	0	0.00%	1.00 [0e+00-Inf]	1.000	1.000
HOXB1	101	0	0.00%	196	0	0.00%	1.00 [0e+00-Inf]	1.000	1.000
HOXB2	101	0	0.00%	196	0	0.00%	1.00 [0e+00-Inf]	1.000	1.000
HOXB3	101	0	0.00%	196	0	0.00%	1.00 [0e+00-Inf]	1.000	1.000
HOXB4	101	0	0.00%	196	0	0.00%	1.00 [0e+00-Inf]	1.000	1.000
HOXB5	101	0	0.00%	196	0	0.00%	1.00 [0e+00-Inf]	1.000	1.000
HOXB6	101	0	0.00%	196	0	0.00%	1.00 [0e+00-Inf]	1.000	1.000
HOXB7	101	0	0.00%	196	0	0.00%	1.00 [0e+00-Inf]	1.000	1.000
HOXB8	101	0	0.00%	196	0	0.00%	1.00 [0e+00-Inf]	1.000	1.000
HOXB9	101	0	0.00%	196	0	0.00%	1.00 [0e+00-Inf]	1.000	1.000
HPS3	101	0	0.00%	196	0	0.00%	1.00 [0e+00-Inf]	1.000	1.000
HPYR1	101	0	0.00%	196	0	0.00%	1.00 [0e+00-Inf]	1.000	1.000
HRH3	101	0	0.00%	196	0	0.00%	1.00 [0e+00-Inf]	1.000	1.000
RBFOX3	101	0	0.00%	196	0	0.00%	1.00 [0e+00-Inf]	1.000	1.000
HRNR	101	0	0.00%	196	0	0.00%	1.00 [0e+00-Inf]	1.000	1.000
HS3ST2	101	0	0.00%	196	0	0.00%	1.00 [0e+00-Inf]	1.000	1.000
HS3ST4	101	0	0.00%	196	0	0.00%	1.00 [0e+00-Inf]	1.000	1.000
HS3ST6	101	0	0.00%	196	0	0.00%	1.00 [0e+00-Inf]	1.000	1.000
HSD11B1	101	0	0.00%	196	0	0.00%	1.00 [0e+00-Inf]	1.000	1.000
HSD17B7	101	0	0.00%	196	0	0.00%	1.00 [0e+00-Inf]	1.000	1.000
HSD3B7	101	0	0.00%	196	0	0.00%	1.00 [0e+00-Inf]	1.000	1.000
HSF1	101	0	0.00%	196	0	0.00%	1.00 [0e+00-Inf]	1.000	1.000
HSF5	101	0	0.00%	196	0	0.00%	1.00 [0e+00-Inf]	1.000	1.000
HSFY2	101	65	64.36%	196	117	59.69%	0.82 [0.54-1.25]	0.435	1.000
HSPA6	101	0	0.00%	196	0	0.00%	1.00 [0e+00-Inf]	1.000	1.000
HSPA7	101	0	0.00%	196	0	0.00%	1.00 [0e+00-Inf]	1.000	1.000
HTR3C	101	0	0.00%	196	0	0.00%	1.00 [0e+00-Inf]	1.000	1.000
HTR3D	101	0	0.00%	196	0	0.00%	1.00 [0e+00-Inf]	1.000	1.000
HTR3E	101	0	0.00%	196	0	0.00%	1.00 [0e+00-Inf]	1.000	1.000
HTRA4	101	0	0.00%	196	0	0.00%	1.00 [0e+00-Inf]	1.000	1.000
IARS2	101	0	0.00%	196	0	0.00%	1.00 [0e+00-Inf]	1.000	1.000
ICAM2	101	0	0.00%	196	0	0.00%	1.00 [0e+00-Inf]	1.000	1.000
IDH2	101	0	0.00%	196	0	0.00%	1.00 [0e+00-Inf]	1.000	1.000
IDO1	101	0	0.00%	196	0	0.00%	1.00 [0e+00-Inf]	1.000	1.000
IDO2	101	0	0.00%	196	0	0.00%	1.00 [0e+00-Inf]	1.000	1.000
IER5	101	0	0.00%	196	0	0.00%	1.00 [0e+00-Inf]	1.000	1.000
IFI16	101	0	0.00%	196	0	0.00%	1.00 [0e+00-Inf]	1.000	1.000
IFNG	101	0	0.00%	196	0	0.00%	1.00 [0e+00-Inf]	1.000	1.000
IFT140	101	0	0.00%	196	0	0.00%	1.00 [0e+00-Inf]	1.000	1.000
IFT20	101	0	0.00%	196	0	0.00%	1.00 [0e+00-Inf]	1.000	1.000
IFT52	101	0	0.00%	196	0	0.00%	1.00 [0e+00-Inf]	1.000	1.000
IGF1R	101	0	0.00%	196	0	0.00%	1.00 [0e+00-Inf]	1.000	1.000
IGF2BP1	101	0	0.00%	196	0	0.00%	1.00 [0e+00-Inf]	1.000	1.000
IGF2BP2	101	0	0.00%	196	0	0.00%	1.00 [0e+00-Inf]	1.000	1.000

Symbol	CINSARC Low			CINSARC High			CINSARC High vs. Low		
	N tested	N altered	Fq alteration	N tested	N altered	Fq alteration	OR [95%CI]	p-value*	q-value
IGF2BP3	101	0	0.00%	196	0	0.00%	1.00 [0e+00-Inf]	1.000	1.000
IGFALS	101	0	0.00%	196	0	0.00%	1.00 [0e+00-Inf]	1.000	1.000
IGFBP4	101	0	0.00%	196	0	0.00%	1.00 [0e+00-Inf]	1.000	1.000
IGFN1	101	0	0.00%	196	0	0.00%	1.00 [0e+00-Inf]	1.000	1.000
IGHMBP2	101	0	0.00%	196	0	0.00%	1.00 [0e+00-Inf]	1.000	1.000
IGSF22	101	0	0.00%	196	0	0.00%	1.00 [0e+00-Inf]	1.000	1.000
IGSF6	101	0	0.00%	196	0	0.00%	1.00 [0e+00-Inf]	1.000	1.000
IGSF8	101	0	0.00%	196	0	0.00%	1.00 [0e+00-Inf]	1.000	1.000
IGSF9	101	0	0.00%	196	0	0.00%	1.00 [0e+00-Inf]	1.000	1.000
IKBKB	101	0	0.00%	196	0	0.00%	1.00 [0e+00-Inf]	1.000	1.000
IKBKE	101	0	0.00%	196	0	0.00%	1.00 [0e+00-Inf]	1.000	1.000
IKZF3	101	0	0.00%	196	0	0.00%	1.00 [0e+00-Inf]	1.000	1.000
IL10	101	0	0.00%	196	0	0.00%	1.00 [0e+00-Inf]	1.000	1.000
IL11RA	101	0	0.00%	196	0	0.00%	1.00 [0e+00-Inf]	1.000	1.000
IL11	101	0	0.00%	196	0	0.00%	1.00 [0e+00-Inf]	1.000	1.000
IL18BP	101	0	0.00%	196	0	0.00%	1.00 [0e+00-Inf]	1.000	1.000
IL19	101	0	0.00%	196	0	0.00%	1.00 [0e+00-Inf]	1.000	1.000
IL1RAP	101	0	0.00%	196	0	0.00%	1.00 [0e+00-Inf]	1.000	1.000
IL20	101	0	0.00%	196	0	0.00%	1.00 [0e+00-Inf]	1.000	1.000
IL21R	101	0	0.00%	196	0	0.00%	1.00 [0e+00-Inf]	1.000	1.000
IL22	101	0	0.00%	196	0	0.00%	1.00 [0e+00-Inf]	1.000	1.000
IL24	101	0	0.00%	196	0	0.00%	1.00 [0e+00-Inf]	1.000	1.000
IL26	101	0	0.00%	196	0	0.00%	1.00 [0e+00-Inf]	1.000	1.000
IL27	101	0	0.00%	196	0	0.00%	1.00 [0e+00-Inf]	1.000	1.000
IL32	101	0	0.00%	196	0	0.00%	1.00 [0e+00-Inf]	1.000	1.000
IL4R	101	0	0.00%	196	0	0.00%	1.00 [0e+00-Inf]	1.000	1.000
IL6R	101	0	0.00%	196	0	0.00%	1.00 [0e+00-Inf]	1.000	1.000
IL6	101	0	0.00%	196	0	0.00%	1.00 [0e+00-Inf]	1.000	1.000
IL7	101	0	0.00%	196	0	0.00%	1.00 [0e+00-Inf]	1.000	1.000
ILDR2	101	0	0.00%	196	0	0.00%	1.00 [0e+00-Inf]	1.000	1.000
ILF2	101	0	0.00%	196	0	0.00%	1.00 [0e+00-Inf]	1.000	1.000
IMPA1	101	0	0.00%	196	0	0.00%	1.00 [0e+00-Inf]	1.000	1.000
IMPAD1	101	0	0.00%	196	0	0.00%	1.00 [0e+00-Inf]	1.000	1.000
INMT	101	0	0.00%	196	0	0.00%	1.00 [0e+00-Inf]	1.000	1.000
INO80E	101	0	0.00%	196	0	0.00%	1.00 [0e+00-Inf]	1.000	1.000
INPPL1	101	0	0.00%	196	0	0.00%	1.00 [0e+00-Inf]	1.000	1.000
INSC	101	0	0.00%	196	0	0.00%	1.00 [0e+00-Inf]	1.000	1.000
INSM2	101	0	0.00%	196	0	0.00%	1.00 [0e+00-Inf]	1.000	1.000
INSRR	101	0	0.00%	196	0	0.00%	1.00 [0e+00-Inf]	1.000	1.000
INTS2	101	0	0.00%	196	0	0.00%	1.00 [0e+00-Inf]	1.000	1.000
INTS3	101	0	0.00%	196	0	0.00%	1.00 [0e+00-Inf]	1.000	1.000
INTS4	101	0	0.00%	196	0	0.00%	1.00 [0e+00-Inf]	1.000	1.000
INTS7	101	0	0.00%	196	0	0.00%	1.00 [0e+00-Inf]	1.000	1.000
INTS8	101	0	0.00%	196	0	0.00%	1.00 [0e+00-Inf]	1.000	1.000
IPO9	101	0	0.00%	196	0	0.00%	1.00 [0e+00-Inf]	1.000	1.000
IQCG	101	0	0.00%	196	0	0.00%	1.00 [0e+00-Inf]	1.000	1.000
IQCK	101	0	0.00%	196	0	0.00%	1.00 [0e+00-Inf]	1.000	1.000
IQGAP1	101	0	0.00%	196	0	0.00%	1.00 [0e+00-Inf]	1.000	1.000
IQGAP3	101	0	0.00%	196	0	0.00%	1.00 [0e+00-Inf]	1.000	1.000
IRAK3	101	0	0.00%	196	0	0.00%	1.00 [0e+00-Inf]	1.000	1.000
IRF2BP2	101	0	0.00%	196	0	0.00%	1.00 [0e+00-Inf]	1.000	1.000
IRF6	101	0	0.00%	196	0	0.00%	1.00 [0e+00-Inf]	1.000	1.000
IRS2	101	0	0.00%	196	0	0.00%	1.00 [0e+00-Inf]	1.000	1.000
ISG20L2	101	0	0.00%	196	0	0.00%	1.00 [0e+00-Inf]	1.000	1.000
ISG20	101	0	0.00%	196	0	0.00%	1.00 [0e+00-Inf]	1.000	1.000
ISOC2	101	0	0.00%	196	0	0.00%	1.00 [0e+00-Inf]	1.000	1.000
ITCH	101	0	0.00%	196	0	0.00%	1.00 [0e+00-Inf]	1.000	1.000
ITGA10	101	0	0.00%	196	0	0.00%	1.00 [0e+00-Inf]	1.000	1.000
ITGA3	101	0	0.00%	196	0	0.00%	1.00 [0e+00-Inf]	1.000	1.000
ITGAD	101	0	0.00%	196	0	0.00%	1.00 [0e+00-Inf]	1.000	1.000
ITGAL	101	0	0.00%	196	0	0.00%	1.00 [0e+00-Inf]	1.000	1.000
ITGAM	101	0	0.00%	196	0	0.00%	1.00 [0e+00-Inf]	1.000	1.000
ITGAX	101	0	0.00%	196	0	0.00%	1.00 [0e+00-Inf]	1.000	1.000
ITGB3	101	0	0.00%	196	0	0.00%	1.00 [0e+00-Inf]	1.000	1.000
ITGB4	101	0	0.00%	196	0	0.00%	1.00 [0e+00-Inf]	1.000	1.000
ITLN1	101	0	0.00%	196	0	0.00%	1.00 [0e+00-Inf]	1.000	1.000
ITLN2	101	0	0.00%	196	0	0.00%	1.00 [0e+00-Inf]	1.000	1.000
ITPKB	101	0	0.00%	196	0	0.00%	1.00 [0e+00-Inf]	1.000	1.000
ITPRIPL2	101	0	0.00%	196	0	0.00%	1.00 [0e+00-Inf]	1.000	1.000
IVL	101	0	0.00%	196	0	0.00%	1.00 [0e+00-Inf]	1.000	1.000
IVNS1ABP	101	0	0.00%	196	0	0.00%	1.00 [0e+00-Inf]	1.000	1.000

Symbol	CINSARC Low			CINSARC High			CINSARC High vs. Low		
	N tested	N altered	Fq alteration	N tested	N altered	Fq alteration	OR [95%CI]	p-value*	q-value
JAZF1	101	0	0.00%	196	0	0.00%	1.00 [0e+00-Inf]	1.000	1.000
JMJD4	101	0	0.00%	196	0	0.00%	1.00 [0e+00-Inf]	1.000	1.000
KDM8	101	0	0.00%	196	0	0.00%	1.00 [0e+00-Inf]	1.000	1.000
JMJD6	101	0	0.00%	196	0	0.00%	1.00 [0e+00-Inf]	1.000	1.000
JMJD8	101	0	0.00%	196	0	0.00%	1.00 [0e+00-Inf]	1.000	1.000
JPH1	101	0	0.00%	196	0	0.00%	1.00 [0e+00-Inf]	1.000	1.000
JPH2	101	0	0.00%	196	0	0.00%	1.00 [0e+00-Inf]	1.000	1.000
JRK	101	0	0.00%	196	0	0.00%	1.00 [0e+00-Inf]	1.000	1.000
JTB	101	0	0.00%	196	0	0.00%	1.00 [0e+00-Inf]	1.000	1.000
KATNAL1	101	0	0.00%	196	0	0.00%	1.00 [0e+00-Inf]	1.000	1.000
KBTBD2	101	0	0.00%	196	0	0.00%	1.00 [0e+00-Inf]	1.000	1.000
KCNB1	101	0	0.00%	196	0	0.00%	1.00 [0e+00-Inf]	1.000	1.000
KCNB2	101	0	0.00%	196	0	0.00%	1.00 [0e+00-Inf]	1.000	1.000
KCNC1	101	0	0.00%	196	0	0.00%	1.00 [0e+00-Inf]	1.000	1.000
KCNC2	101	0	0.00%	196	0	0.00%	1.00 [0e+00-Inf]	1.000	1.000
KCNE3	101	0	0.00%	196	0	0.00%	1.00 [0e+00-Inf]	1.000	1.000
KCNG1	101	0	0.00%	196	0	0.00%	1.00 [0e+00-Inf]	1.000	1.000
KCNH1	101	0	0.00%	196	0	0.00%	1.00 [0e+00-Inf]	1.000	1.000
KCNH6	101	0	0.00%	196	0	0.00%	1.00 [0e+00-Inf]	1.000	1.000
KCNJ10	101	0	0.00%	196	0	0.00%	1.00 [0e+00-Inf]	1.000	1.000
KCNJ11	101	0	0.00%	196	0	0.00%	1.00 [0e+00-Inf]	1.000	1.000
KCNJ16	101	0	0.00%	196	0	0.00%	1.00 [0e+00-Inf]	1.000	1.000
KCNJ2	101	0	0.00%	196	0	0.00%	1.00 [0e+00-Inf]	1.000	1.000
KCNJ9	101	0	0.00%	196	0	0.00%	1.00 [0e+00-Inf]	1.000	1.000
KCNK15	101	0	0.00%	196	0	0.00%	1.00 [0e+00-Inf]	1.000	1.000
KCNK1	101	0	0.00%	196	0	0.00%	1.00 [0e+00-Inf]	1.000	1.000
KCNK2	101	0	0.00%	196	0	0.00%	1.00 [0e+00-Inf]	1.000	1.000
KCNK9	101	0	0.00%	196	0	0.00%	1.00 [0e+00-Inf]	1.000	1.000
KCNMB2	101	0	0.00%	196	0	0.00%	1.00 [0e+00-Inf]	1.000	1.000
KCNMB3	101	0	0.00%	196	0	0.00%	1.00 [0e+00-Inf]	1.000	1.000
KCNMB4	101	0	0.00%	196	0	0.00%	1.00 [0e+00-Inf]	1.000	1.000
KCNN3	101	0	0.00%	196	0	0.00%	1.00 [0e+00-Inf]	1.000	1.000
KCNQ2	101	0	0.00%	196	0	0.00%	1.00 [0e+00-Inf]	1.000	1.000
KCNQ3	101	0	0.00%	196	0	0.00%	1.00 [0e+00-Inf]	1.000	1.000
KCNS1	101	0	0.00%	196	0	0.00%	1.00 [0e+00-Inf]	1.000	1.000
KCNS2	101	0	0.00%	196	0	0.00%	1.00 [0e+00-Inf]	1.000	1.000
KCNT2	101	0	0.00%	196	0	0.00%	1.00 [0e+00-Inf]	1.000	1.000
KCNU1	101	0	0.00%	196	0	0.00%	1.00 [0e+00-Inf]	1.000	1.000
KCNV1	101	0	0.00%	196	0	0.00%	1.00 [0e+00-Inf]	1.000	1.000
KCTD13	101	0	0.00%	196	0	0.00%	1.00 [0e+00-Inf]	1.000	1.000
KCTD14	101	0	0.00%	196	0	0.00%	1.00 [0e+00-Inf]	1.000	1.000
KCTD21	101	0	0.00%	196	0	0.00%	1.00 [0e+00-Inf]	1.000	1.000
KCTD2	101	0	0.00%	196	0	0.00%	1.00 [0e+00-Inf]	1.000	1.000
KCTD3	101	0	0.00%	196	0	0.00%	1.00 [0e+00-Inf]	1.000	1.000
KCTD5	101	0	0.00%	196	0	0.00%	1.00 [0e+00-Inf]	1.000	1.000
KDM2A	101	0	0.00%	196	0	0.00%	1.00 [0e+00-Inf]	1.000	1.000
KDM5B	101	0	0.00%	196	0	0.00%	1.00 [0e+00-Inf]	1.000	1.000
KDM5D	101	64	63.37%	196	116	59.18%	0.84 [0.55-1.27]	0.485	1.000
KDSR	101	0	0.00%	196	0	0.00%	1.00 [0e+00-Inf]	1.000	1.000
KEL	101	0	0.00%	196	0	0.00%	1.00 [0e+00-Inf]	1.000	1.000
KERA	101	1	0.99%	196	0	0.00%	1.6e-08 [0e+00-Inf]	0.996	1.000
KHDRBS3	101	0	0.00%	196	0	0.00%	1.00 [0e+00-Inf]	1.000	1.000
KIAA0040	101	0	0.00%	196	0	0.00%	1.00 [0e+00-Inf]	1.000	1.000
KIAA0087	101	0	0.00%	196	0	0.00%	1.00 [0e+00-Inf]	1.000	1.000
KIAA0100	101	0	0.00%	196	0	0.00%	1.00 [0e+00-Inf]	1.000	1.000
SPIDR	101	0	0.00%	196	0	0.00%	1.00 [0e+00-Inf]	1.000	1.000
KIAA0391	101	0	0.00%	196	0	0.00%	1.00 [0e+00-Inf]	1.000	1.000
TTI1	101	0	0.00%	196	0	0.00%	1.00 [0e+00-Inf]	1.000	1.000
KIAA0556	101	0	0.00%	196	0	0.00%	1.00 [0e+00-Inf]	1.000	1.000
CLUHP3	101	0	0.00%	196	0	0.00%	1.00 [0e+00-Inf]	1.000	1.000
ZSWIM8	101	0	0.00%	196	0	0.00%	1.00 [0e+00-Inf]	1.000	1.000
MAP10	101	0	0.00%	196	0	0.00%	1.00 [0e+00-Inf]	1.000	1.000
KIAA1614	101	0	0.00%	196	0	0.00%	1.00 [0e+00-Inf]	1.000	1.000
KIAA1755	101	0	0.00%	196	0	0.00%	1.00 [0e+00-Inf]	1.000	1.000
KIF14	101	0	0.00%	196	0	0.00%	1.00 [0e+00-Inf]	1.000	1.000
KIF19	101	0	0.00%	196	0	0.00%	1.00 [0e+00-Inf]	1.000	1.000
KIF21B	101	0	0.00%	196	0	0.00%	1.00 [0e+00-Inf]	1.000	1.000
KIF22	101	0	0.00%	196	0	0.00%	1.00 [0e+00-Inf]	1.000	1.000
KIF26B	101	0	0.00%	196	0	0.00%	1.00 [0e+00-Inf]	1.000	1.000
KIF2B	101	0	0.00%	196	0	0.00%	1.00 [0e+00-Inf]	1.000	1.000
KIF7	101	0	0.00%	196	0	0.00%	1.00 [0e+00-Inf]	1.000	1.000

Symbol	CINSARC Low			CINSARC High			CINSARC High vs. Low		
	N tested	N altered	Fq alteration	N tested	N altered	Fq alteration	OR [95%CI]	p-value*	q-value
KIFAP3	101	0	0.00%	196	0	0.00%	1.00 [0e+00-Inf]	1.000	1.000
KIFC2	101	0	0.00%	196	0	0.00%	1.00 [0e+00-Inf]	1.000	1.000
KIR2DL1	101	0	0.00%	196	0	0.00%	1.00 [0e+00-Inf]	1.000	1.000
KIR2DL3	101	0	0.00%	196	0	0.00%	1.00 [0e+00-Inf]	1.000	1.000
KIR2DL4	101	0	0.00%	196	0	0.00%	1.00 [0e+00-Inf]	1.000	1.000
KIR3DL1	101	0	0.00%	196	0	0.00%	1.00 [0e+00-Inf]	1.000	1.000
KIR3DL3	101	0	0.00%	196	0	0.00%	1.00 [0e+00-Inf]	1.000	1.000
KISS1	101	0	0.00%	196	0	0.00%	1.00 [0e+00-Inf]	1.000	1.000
KITLG	101	0	0.00%	196	0	0.00%	1.00 [0e+00-Inf]	1.000	1.000
KLC2	101	0	0.00%	196	0	0.00%	1.00 [0e+00-Inf]	1.000	1.000
KLF10	101	0	0.00%	196	0	0.00%	1.00 [0e+00-Inf]	1.000	1.000
KLHDC8A	101	0	0.00%	196	0	0.00%	1.00 [0e+00-Inf]	1.000	1.000
KLHDC9	101	0	0.00%	196	0	0.00%	1.00 [0e+00-Inf]	1.000	1.000
KLHL12	101	0	0.00%	196	0	0.00%	1.00 [0e+00-Inf]	1.000	1.000
KLHL20	101	0	0.00%	196	0	0.00%	1.00 [0e+00-Inf]	1.000	1.000
KLHL24	101	0	0.00%	196	0	0.00%	1.00 [0e+00-Inf]	1.000	1.000
KLHL25	101	0	0.00%	196	0	0.00%	1.00 [0e+00-Inf]	1.000	1.000
KLHL35	101	0	0.00%	196	0	0.00%	1.00 [0e+00-Inf]	1.000	1.000
KLHL38	101	0	0.00%	196	0	0.00%	1.00 [0e+00-Inf]	1.000	1.000
KLHL6	101	0	0.00%	196	0	0.00%	1.00 [0e+00-Inf]	1.000	1.000
KLHL7	101	0	0.00%	196	0	0.00%	1.00 [0e+00-Inf]	1.000	1.000
KMO	101	0	0.00%	196	0	0.00%	1.00 [0e+00-Inf]	1.000	1.000
KPNA2	101	0	0.00%	196	0	0.00%	1.00 [0e+00-Inf]	1.000	1.000
KPNB1	101	0	0.00%	196	0	0.00%	1.00 [0e+00-Inf]	1.000	1.000
KPRP	101	0	0.00%	196	0	0.00%	1.00 [0e+00-Inf]	1.000	1.000
KREMEN2	101	0	0.00%	196	0	0.00%	1.00 [0e+00-Inf]	1.000	1.000
KRR1	101	0	0.00%	196	0	0.00%	1.00 [0e+00-Inf]	1.000	1.000
KRT10	101	0	0.00%	196	0	0.00%	1.00 [0e+00-Inf]	1.000	1.000
KRT12	101	0	0.00%	196	0	0.00%	1.00 [0e+00-Inf]	1.000	1.000
KRT222	101	0	0.00%	196	0	0.00%	1.00 [0e+00-Inf]	1.000	1.000
KRT23	101	0	0.00%	196	0	0.00%	1.00 [0e+00-Inf]	1.000	1.000
KRT24	101	0	0.00%	196	0	0.00%	1.00 [0e+00-Inf]	1.000	1.000
KRT25	101	0	0.00%	196	0	0.00%	1.00 [0e+00-Inf]	1.000	1.000
KRT26	101	0	0.00%	196	0	0.00%	1.00 [0e+00-Inf]	1.000	1.000
KRT27	101	0	0.00%	196	0	0.00%	1.00 [0e+00-Inf]	1.000	1.000
KRT28	101	0	0.00%	196	0	0.00%	1.00 [0e+00-Inf]	1.000	1.000
KRT34	101	0	0.00%	196	0	0.00%	1.00 [0e+00-Inf]	1.000	1.000
KRTAP5-10	101	0	0.00%	196	0	0.00%	1.00 [0e+00-Inf]	1.000	1.000
KRTAP5-11	101	0	0.00%	196	0	0.00%	1.00 [0e+00-Inf]	1.000	1.000
KRTAP5-7	101	0	0.00%	196	0	0.00%	1.00 [0e+00-Inf]	1.000	1.000
KRTAP5-8	101	0	0.00%	196	0	0.00%	1.00 [0e+00-Inf]	1.000	1.000
KRTAP5-9	101	0	0.00%	196	0	0.00%	1.00 [0e+00-Inf]	1.000	1.000
KRTCAP2	101	0	0.00%	196	0	0.00%	1.00 [0e+00-Inf]	1.000	1.000
KSR1	101	0	0.00%	196	0	0.00%	1.00 [0e+00-Inf]	1.000	1.000
L3MBTL1	101	0	0.00%	196	0	0.00%	1.00 [0e+00-Inf]	1.000	1.000
LACTB2	101	0	0.00%	196	0	0.00%	1.00 [0e+00-Inf]	1.000	1.000
LAD1	101	0	0.00%	196	0	0.00%	1.00 [0e+00-Inf]	1.000	1.000
LAMA5	101	0	0.00%	196	0	0.00%	1.00 [0e+00-Inf]	1.000	1.000
LAMB3	101	0	0.00%	196	0	0.00%	1.00 [0e+00-Inf]	1.000	1.000
LAMC1	101	0	0.00%	196	0	0.00%	1.00 [0e+00-Inf]	1.000	1.000
LAMC2	101	0	0.00%	196	0	0.00%	1.00 [0e+00-Inf]	1.000	1.000
LAMP3	101	0	0.00%	196	0	0.00%	1.00 [0e+00-Inf]	1.000	1.000
LAPTM4B	101	0	0.00%	196	0	0.00%	1.00 [0e+00-Inf]	1.000	1.000
LASP1	101	0	0.00%	196	0	0.00%	1.00 [0e+00-Inf]	1.000	1.000
CERS2	101	0	0.00%	196	0	0.00%	1.00 [0e+00-Inf]	1.000	1.000
LAT	101	0	0.00%	196	0	0.00%	1.00 [0e+00-Inf]	1.000	1.000
LAX1	101	0	0.00%	196	0	0.00%	1.00 [0e+00-Inf]	1.000	1.000
LBP	101	0	0.00%	196	0	0.00%	1.00 [0e+00-Inf]	1.000	1.000
LBR	101	0	0.00%	196	0	0.00%	1.00 [0e+00-Inf]	1.000	1.000
LCE1A	101	0	0.00%	196	0	0.00%	1.00 [0e+00-Inf]	1.000	1.000
LCE1B	101	0	0.00%	196	0	0.00%	1.00 [0e+00-Inf]	1.000	1.000
LCE1C	101	0	0.00%	196	0	0.00%	1.00 [0e+00-Inf]	1.000	1.000
LCE1D	101	0	0.00%	196	0	0.00%	1.00 [0e+00-Inf]	1.000	1.000
LCE1E	101	0	0.00%	196	0	0.00%	1.00 [0e+00-Inf]	1.000	1.000
LCE1F	101	0	0.00%	196	0	0.00%	1.00 [0e+00-Inf]	1.000	1.000
LCE2A	101	0	0.00%	196	0	0.00%	1.00 [0e+00-Inf]	1.000	1.000
LCE2B	101	0	0.00%	196	0	0.00%	1.00 [0e+00-Inf]	1.000	1.000
LCE2C	101	0	0.00%	196	0	0.00%	1.00 [0e+00-Inf]	1.000	1.000
LCE2D	101	0	0.00%	196	0	0.00%	1.00 [0e+00-Inf]	1.000	1.000
LCE3A	101	0	0.00%	196	0	0.00%	1.00 [0e+00-Inf]	1.000	1.000
LCE3B	101	3	2.97%	196	7	3.57%	1.21 [0.38-3.83]	0.786	1.000

Symbol	CINSARC Low			CINSARC High			CINSARC High vs. Low		
	N tested	N altered	Fq alteration	N tested	N altered	Fq alteration	OR [95%CI]	p-value*	q-value
LCE3C	101	3	2.97%	196	8	4.08%	1.39 [0.45-4.31]	0.632	1.000
LCE3D	101	0	0.00%	196	0	0.00%	1.00 [0e+00-Inf]	1.000	1.000
LCE3E	101	0	0.00%	196	0	0.00%	1.00 [0e+00-Inf]	1.000	1.000
LCE4A	101	0	0.00%	196	0	0.00%	1.00 [0e+00-Inf]	1.000	1.000
LCE5A	101	0	0.00%	196	0	0.00%	1.00 [0e+00-Inf]	1.000	1.000
LCE6A	101	0	0.00%	196	0	0.00%	1.00 [0e+00-Inf]	1.000	1.000
LCMT1	101	0	0.00%	196	0	0.00%	1.00 [0e+00-Inf]	1.000	1.000
LDHAL6A	101	0	0.00%	196	0	0.00%	1.00 [0e+00-Inf]	1.000	1.000
LDHA	101	0	0.00%	196	0	0.00%	1.00 [0e+00-Inf]	1.000	1.000
LDHC	101	0	0.00%	196	0	0.00%	1.00 [0e+00-Inf]	1.000	1.000
LEFTY1	101	0	0.00%	196	0	0.00%	1.00 [0e+00-Inf]	1.000	1.000
LEFTY2	101	0	0.00%	196	0	0.00%	1.00 [0e+00-Inf]	1.000	1.000
LELP1	101	0	0.00%	196	0	0.00%	1.00 [0e+00-Inf]	1.000	1.000
LEMD1	101	0	0.00%	196	0	0.00%	1.00 [0e+00-Inf]	1.000	1.000
LEMD3	101	0	0.00%	196	0	0.00%	1.00 [0e+00-Inf]	1.000	1.000
LENEP	101	0	0.00%	196	0	0.00%	1.00 [0e+00-Inf]	1.000	1.000
LETM2	101	0	0.00%	196	0	0.00%	1.00 [0e+00-Inf]	1.000	1.000
LGALS3BP	101	0	0.00%	196	0	0.00%	1.00 [0e+00-Inf]	1.000	1.000
LGALS8	101	0	0.00%	196	0	0.00%	1.00 [0e+00-Inf]	1.000	1.000
LGALS9C	101	1	0.99%	196	1	0.51%	0.51 [0.05-5.30]	0.638	1.000
LGALS9	101	0	0.00%	196	0	0.00%	1.00 [0e+00-Inf]	1.000	1.000
LGR5	101	0	0.00%	196	0	0.00%	1.00 [0e+00-Inf]	1.000	1.000
LGR6	101	0	0.00%	196	0	0.00%	1.00 [0e+00-Inf]	1.000	1.000
LHX1	101	0	0.00%	196	0	0.00%	1.00 [0e+00-Inf]	1.000	1.000
LHX4	101	0	0.00%	196	0	0.00%	1.00 [0e+00-Inf]	1.000	1.000
LHX9	101	0	0.00%	196	0	0.00%	1.00 [0e+00-Inf]	1.000	1.000
LIG3	101	0	0.00%	196	0	0.00%	1.00 [0e+00-Inf]	1.000	1.000
LIMD2	101	0	0.00%	196	0	0.00%	1.00 [0e+00-Inf]	1.000	1.000
LIME1	101	0	0.00%	196	0	0.00%	1.00 [0e+00-Inf]	1.000	1.000
LIN28B	101	0	0.00%	196	0	0.00%	1.00 [0e+00-Inf]	1.000	1.000
LIN7A	101	0	0.00%	196	0	0.00%	1.00 [0e+00-Inf]	1.000	1.000
LIN9	101	0	0.00%	196	0	0.00%	1.00 [0e+00-Inf]	1.000	1.000
LINGO4	101	0	0.00%	196	0	0.00%	1.00 [0e+00-Inf]	1.000	1.000
LIPH	101	0	0.00%	196	0	0.00%	1.00 [0e+00-Inf]	1.000	1.000
LIPT2	101	0	0.00%	196	0	0.00%	1.00 [0e+00-Inf]	1.000	1.000
LITAF	101	0	0.00%	196	0	0.00%	1.00 [0e+00-Inf]	1.000	1.000
LIX1L	101	0	0.00%	196	0	0.00%	1.00 [0e+00-Inf]	1.000	1.000
LLGL2	101	0	0.00%	196	0	0.00%	1.00 [0e+00-Inf]	1.000	1.000
LLPH	101	0	0.00%	196	0	0.00%	1.00 [0e+00-Inf]	1.000	1.000
LMAN1	101	0	0.00%	196	0	0.00%	1.00 [0e+00-Inf]	1.000	1.000
LMF1	101	0	0.00%	196	0	0.00%	1.00 [0e+00-Inf]	1.000	1.000
LMLN	101	0	0.00%	196	0	0.00%	1.00 [0e+00-Inf]	1.000	1.000
LMNA	101	0	0.00%	196	8	0.00%	1.00 [0e+00-Inf]	1.000	1.000
LMOD1	101	0	0.00%	196	0	0.00%	1.00 [0e+00-Inf]	1.000	1.000
LMX1A	101	0	0.00%	196	0	0.00%	1.00 [0e+00-Inf]	1.000	1.000
LOC10012678	101	0	0.00%	196	0	0.00%	1.00 [0e+00-Inf]	1.000	1.000
LOC10013098	101	0	0.00%	196	0	0.00%	1.00 [0e+00-Inf]	1.000	1.000
LOC10013149	101	0	0.00%	196	0	0.00%	1.00 [0e+00-Inf]	1.000	1.000
LOC10013436	101	0	0.00%	196	0	0.00%	1.00 [0e+00-Inf]	1.000	1.000
LOC10019098	101	1	0.99%	196	0	0.00%	1.6e-08 [0e+00-Inf]	0.996	1.000
LOC148696	101	0	0.00%	196	0	0.00%	1.00 [0e+00-Inf]	1.000	1.000
LOC154761	101	0	0.00%	196	0	0.00%	1.00 [0e+00-Inf]	1.000	1.000
LOC220729	101	0	0.00%	196	0	0.00%	1.00 [0e+00-Inf]	1.000	1.000
LOC388242	101	0	0.00%	196	0	0.00%	1.00 [0e+00-Inf]	1.000	1.000
LOC391322	101	4	3.96%	196	4	2.04%	0.51 [0.16-1.65]	0.342	1.000
LOC401463	101	0	0.00%	196	0	0.00%	1.00 [0e+00-Inf]	1.000	1.000
LOC441204	101	0	0.00%	196	0	0.00%	1.00 [0e+00-Inf]	1.000	1.000
LOC606724	101	0	0.00%	196	0	0.00%	1.00 [0e+00-Inf]	1.000	1.000
LOC646214	101	0	0.00%	196	0	0.00%	1.00 [0e+00-Inf]	1.000	1.000
LOC646762	101	0	0.00%	196	0	0.00%	1.00 [0e+00-Inf]	1.000	1.000
LOC652276	101	0	0.00%	196	0	0.00%	1.00 [0e+00-Inf]	1.000	1.000
LOC653653	101	0	0.00%	196	0	0.00%	1.00 [0e+00-Inf]	1.000	1.000
LOC728024	101	0	0.00%	196	0	0.00%	1.00 [0e+00-Inf]	1.000	1.000
LOC728989	101	0	0.00%	196	0	0.00%	1.00 [0e+00-Inf]	1.000	1.000
LOR	101	0	0.00%	196	0	0.00%	1.00 [0e+00-Inf]	1.000	1.000
LPGAT1	101	0	0.00%	196	0	0.00%	1.00 [0e+00-Inf]	1.000	1.000
LPIN3	101	0	0.00%	196	0	0.00%	1.00 [0e+00-Inf]	1.000	1.000
LPO	101	0	0.00%	196	0	0.00%	1.00 [0e+00-Inf]	1.000	1.000
LPP	101	0	0.00%	196	8	0.00%	1.00 [0e+00-Inf]	1.000	1.000
LRCH3	101	0	0.00%	196	0	0.00%	1.00 [0e+00-Inf]	1.000	1.000
LRFN4	101	0	0.00%	196	0	0.00%	1.00 [0e+00-Inf]	1.000	1.000

Symbol	CINSARC Low			CINSARC High			CINSARC High vs. Low		
	N tested	N altered	Fq alteration	N tested	N altered	Fq alteration	OR [95%CI]	p-value*	q-value
LRP12	101	0	0.00%	196	0	0.00%	1.00 [0e+00-Inf]	1.000	1.000
LRP5	101	0	0.00%	196	0	0.00%	1.00 [0e+00-Inf]	1.000	1.000
LRRC10B	101	0	0.00%	196	0	0.00%	1.00 [0e+00-Inf]	1.000	1.000
LRRC10	101	0	0.00%	196	0	0.00%	1.00 [0e+00-Inf]	1.000	1.000
LRRC14	101	0	0.00%	196	0	0.00%	1.00 [0e+00-Inf]	1.000	1.000
LRRC15	101	0	0.00%	196	0	0.00%	1.00 [0e+00-Inf]	1.000	1.000
LRRC24	101	0	0.00%	196	0	0.00%	1.00 [0e+00-Inf]	1.000	1.000
LRRC28	101	0	0.00%	196	0	0.00%	1.00 [0e+00-Inf]	1.000	1.000
LRRC32	101	0	0.00%	196	0	0.00%	1.00 [0e+00-Inf]	1.000	1.000
NRROS	101	0	0.00%	196	0	0.00%	1.00 [0e+00-Inf]	1.000	1.000
LRRC37A2	101	0	0.00%	196	0	0.00%	1.00 [0e+00-Inf]	1.000	1.000
LRRC37A3	101	0	0.00%	196	0	0.00%	1.00 [0e+00-Inf]	1.000	1.000
LRRC37A	101	0	0.00%	196	0	0.00%	1.00 [0e+00-Inf]	1.000	1.000
LRRC45	101	0	0.00%	196	0	0.00%	1.00 [0e+00-Inf]	1.000	1.000
LRRC46	101	0	0.00%	196	0	0.00%	1.00 [0e+00-Inf]	1.000	1.000
LRRC52	101	0	0.00%	196	0	0.00%	1.00 [0e+00-Inf]	1.000	1.000
LRRC59	101	0	0.00%	196	0	0.00%	1.00 [0e+00-Inf]	1.000	1.000
PPP1R42	101	0	0.00%	196	0	0.00%	1.00 [0e+00-Inf]	1.000	1.000
LRRC69	101	0	0.00%	196	0	0.00%	1.00 [0e+00-Inf]	1.000	1.000
LRRC6	101	0	0.00%	196	0	0.00%	1.00 [0e+00-Inf]	1.000	1.000
LRRCC1	101	0	0.00%	196	0	0.00%	1.00 [0e+00-Inf]	1.000	1.000
LRRN2	101	0	0.00%	196	0	0.00%	1.00 [0e+00-Inf]	1.000	1.000
LRTOMT	101	0	0.00%	196	0	0.00%	1.00 [0e+00-Inf]	1.000	1.000
LSG1	101	0	0.00%	196	0	0.00%	1.00 [0e+00-Inf]	1.000	1.000
LSM14B	101	0	0.00%	196	0	0.00%	1.00 [0e+00-Inf]	1.000	1.000
LSM1	101	0	0.00%	196	0	0.00%	1.00 [0e+00-Inf]	1.000	1.000
LSM5	101	0	0.00%	196	0	0.00%	1.00 [0e+00-Inf]	1.000	1.000
LUC7L3	101	0	0.00%	196	0	0.00%	1.00 [0e+00-Inf]	1.000	1.000
LUC7L	101	0	0.00%	196	0	0.00%	1.00 [0e+00-Inf]	1.000	1.000
LUM	101	0	0.00%	196	0	0.00%	1.00 [0e+00-Inf]	1.000	1.000
LY6D	101	0	0.00%	196	0	0.00%	1.00 [0e+00-Inf]	1.000	1.000
LY6E	101	0	0.00%	196	0	0.00%	1.00 [0e+00-Inf]	1.000	1.000
LY6H	101	0	0.00%	196	0	0.00%	1.00 [0e+00-Inf]	1.000	1.000
LY6K	101	0	0.00%	196	0	0.00%	1.00 [0e+00-Inf]	1.000	1.000
LY96	101	0	0.00%	196	0	0.00%	1.00 [0e+00-Inf]	1.000	1.000
LY9	101	0	0.00%	196	0	0.00%	1.00 [0e+00-Inf]	1.000	1.000
LYNX1	101	0	0.00%	196	0	0.00%	1.00 [0e+00-Inf]	1.000	1.000
LYN	101	0	0.00%	196	0	0.00%	1.00 [0e+00-Inf]	1.000	1.000
LYPD2	101	0	0.00%	196	0	0.00%	1.00 [0e+00-Inf]	1.000	1.000
LYPLA1	101	0	0.00%	196	0	0.00%	1.00 [0e+00-Inf]	1.000	1.000
LYPLAL1	101	0	0.00%	196	0	0.00%	1.00 [0e+00-Inf]	1.000	1.000
LYRM1	101	0	0.00%	196	0	0.00%	1.00 [0e+00-Inf]	1.000	1.000
LYSMD1	101	0	0.00%	196	0	0.00%	1.00 [0e+00-Inf]	1.000	1.000
LYSMD4	101	0	0.00%	196	0	0.00%	1.00 [0e+00-Inf]	1.000	1.000
LYST	101	0	0.00%	196	0	0.00%	1.00 [0e+00-Inf]	1.000	1.000
LYZL6	101	0	0.00%	196	0	0.00%	1.00 [0e+00-Inf]	1.000	1.000
LYZ	101	0	0.00%	196	0	0.00%	1.00 [0e+00-Inf]	1.000	1.000
MAEL	101	0	0.00%	196	0	0.00%	1.00 [0e+00-Inf]	1.000	1.000
MAF1	101	0	0.00%	196	0	0.00%	1.00 [0e+00-Inf]	1.000	1.000
MAFA	101	0	0.00%	196	0	0.00%	1.00 [0e+00-Inf]	1.000	1.000
MAFB	101	0	0.00%	196	0	0.00%	1.00 [0e+00-Inf]	1.000	1.000
MAFG	101	0	0.00%	196	0	0.00%	1.00 [0e+00-Inf]	1.000	1.000
MAGEF1	101	0	0.00%	196	0	0.00%	1.00 [0e+00-Inf]	1.000	1.000
MAK16	101	0	0.00%	196	0	0.00%	1.00 [0e+00-Inf]	1.000	1.000
MAL2	101	0	0.00%	196	0	0.00%	1.00 [0e+00-Inf]	1.000	1.000
MALT1	101	0	0.00%	196	0	0.00%	1.00 [0e+00-Inf]	1.000	1.000
MAN2A2	101	0	0.00%	196	0	0.00%	1.00 [0e+00-Inf]	1.000	1.000
MANBAL	101	0	0.00%	196	0	0.00%	1.00 [0e+00-Inf]	1.000	1.000
MAP1LC3A	101	0	0.00%	196	0	0.00%	1.00 [0e+00-Inf]	1.000	1.000
MAP1LC3C	101	0	0.00%	196	0	0.00%	1.00 [0e+00-Inf]	1.000	1.000
MAP2K4	101	1	0.99%	196	4	2.04%	2.08 [0.33-13.25]	0.514	1.000
MAP2K6	101	0	0.00%	196	0	0.00%	1.00 [0e+00-Inf]	1.000	1.000
MAP3K13	101	0	0.00%	196	0	0.00%	1.00 [0e+00-Inf]	1.000	1.000
MAP3K3	101	0	0.00%	196	0	0.00%	1.00 [0e+00-Inf]	1.000	1.000
MAP6D1	101	0	0.00%	196	0	0.00%	1.00 [0e+00-Inf]	1.000	1.000
MAP6	101	0	0.00%	196	0	0.00%	1.00 [0e+00-Inf]	1.000	1.000
MAPK15	101	0	0.00%	196	0	0.00%	1.00 [0e+00-Inf]	1.000	1.000
MAPK3	101	0	0.00%	196	0	0.00%	1.00 [0e+00-Inf]	1.000	1.000
MAPK7	101	0	0.00%	196	0	0.00%	1.00 [0e+00-Inf]	1.000	1.000
MAPK8IP3	101	0	0.00%	196	0	0.00%	1.00 [0e+00-Inf]	1.000	1.000
MAPKAPK2	101	0	0.00%	196	0	0.00%	1.00 [0e+00-Inf]	1.000	1.000

Symbol	CINSARC Low			CINSARC High			CINSARC High vs. Low		
	N tested	N altered	Fq alteration	N tested	N altered	Fq alteration	OR [95%CI]	p-value*	q-value
MARCH10	101	0	0.00%	196	0	0.00%	1.00 [0e+00-Inf]	1.000	1.000
MARCH9	101	0	0.00%	196	0	0.00%	1.00 [0e+00-Inf]	1.000	1.000
MARK1	101	0	0.00%	196	0	0.00%	1.00 [0e+00-Inf]	1.000	1.000
MATN2	101	0	0.00%	196	0	0.00%	1.00 [0e+00-Inf]	1.000	1.000
MATN4	101	0	0.00%	196	0	0.00%	1.00 [0e+00-Inf]	1.000	1.000
MAZ	101	0	0.00%	196	0	0.00%	1.00 [0e+00-Inf]	1.000	1.000
MBIP	101	0	0.00%	196	0	0.00%	1.00 [0e+00-Inf]	1.000	1.000
MBTD1	101	0	0.00%	196	0	0.00%	1.00 [0e+00-Inf]	1.000	1.000
MC3R	101	0	0.00%	196	0	0.00%	1.00 [0e+00-Inf]	1.000	1.000
MCCC1	101	0	0.00%	196	0	0.00%	1.00 [0e+00-Inf]	1.000	1.000
MCF2L2	101	0	0.00%	196	0	0.00%	1.00 [0e+00-Inf]	1.000	1.000
MCL1	101	0	0.00%	196	0	0.00%	1.00 [0e+00-Inf]	1.000	1.000
MCM4	101	0	0.00%	196	0	0.00%	1.00 [0e+00-Inf]	1.000	1.000
MCTP2	101	0	0.00%	196	0	0.00%	1.00 [0e+00-Inf]	1.000	1.000
MDM1	101	0	0.00%	196	0	0.00%	1.00 [0e+00-Inf]	1.000	1.000
MDM2	101	0	0.00%	196	0	0.00%	1.00 [0e+00-Inf]	1.000	1.000
MDM4	101	0	0.00%	196	0	0.00%	1.00 [0e+00-Inf]	1.000	1.000
MED13	101	0	0.00%	196	0	0.00%	1.00 [0e+00-Inf]	1.000	1.000
MED1	101	0	0.00%	196	0	0.00%	1.00 [0e+00-Inf]	1.000	1.000
MED24	101	0	0.00%	196	0	0.00%	1.00 [0e+00-Inf]	1.000	1.000
MED30	101	0	0.00%	196	0	0.00%	1.00 [0e+00-Inf]	1.000	1.000
MEF2A	101	0	0.00%	196	0	0.00%	1.00 [0e+00-Inf]	1.000	1.000
MEF2D	101	0	0.00%	196	0	0.00%	1.00 [0e+00-Inf]	1.000	1.000
MEFV	101	0	0.00%	196	0	0.00%	1.00 [0e+00-Inf]	1.000	1.000
MESP1	101	0	0.00%	196	0	0.00%	1.00 [0e+00-Inf]	1.000	1.000
MESP2	101	0	0.00%	196	0	0.00%	1.00 [0e+00-Inf]	1.000	1.000
METRNL	101	0	0.00%	196	0	0.00%	1.00 [0e+00-Inf]	1.000	1.000
METRN	101	0	0.00%	196	0	0.00%	1.00 [0e+00-Inf]	1.000	1.000
METTL11B	101	0	0.00%	196	0	0.00%	1.00 [0e+00-Inf]	1.000	1.000
METTL1	101	0	0.00%	196	0	0.00%	1.00 [0e+00-Inf]	1.000	1.000
METTL2A	101	0	0.00%	196	0	0.00%	1.00 [0e+00-Inf]	1.000	1.000
METTL9	101	0	0.00%	196	0	0.00%	1.00 [0e+00-Inf]	1.000	1.000
MEX3A	101	0	0.00%	196	0	0.00%	1.00 [0e+00-Inf]	1.000	1.000
MFAP4	101	0	0.00%	196	0	0.00%	1.00 [0e+00-Inf]	1.000	1.000
MFGE8	101	0	0.00%	196	0	0.00%	1.00 [0e+00-Inf]	1.000	1.000
MFN1	101	0	0.00%	196	0	0.00%	1.00 [0e+00-Inf]	1.000	1.000
MFSD11	101	0	0.00%	196	0	0.00%	1.00 [0e+00-Inf]	1.000	1.000
MFSD3	101	0	0.00%	196	0	0.00%	1.00 [0e+00-Inf]	1.000	1.000
MGAT5B	101	0	0.00%	196	0	0.00%	1.00 [0e+00-Inf]	1.000	1.000
MGC16275	101	0	0.00%	196	0	0.00%	1.00 [0e+00-Inf]	1.000	1.000
MGC2889	101	0	0.00%	196	0	0.00%	1.00 [0e+00-Inf]	1.000	1.000
MGRN1	101	0	0.00%	196	0	0.00%	1.00 [0e+00-Inf]	1.000	1.000
MGST3	101	0	0.00%	196	0	0.00%	1.00 [0e+00-Inf]	1.000	1.000
MIA3	101	0	0.00%	196	0	0.00%	1.00 [0e+00-Inf]	1.000	1.000
MIF4GD	101	0	0.00%	196	0	0.00%	1.00 [0e+00-Inf]	1.000	1.000
MIPOL1	101	0	0.00%	196	0	0.00%	1.00 [0e+00-Inf]	1.000	1.000
MIXL1	101	0	0.00%	196	0	0.00%	1.00 [0e+00-Inf]	1.000	1.000
MKRN1	101	0	0.00%	196	0	0.00%	1.00 [0e+00-Inf]	1.000	1.000
MKS1	101	0	0.00%	196	0	0.00%	1.00 [0e+00-Inf]	1.000	1.000
MLLT11	101	0	0.00%	196	0	0.00%	1.00 [0e+00-Inf]	1.000	1.000
MLLT6	101	0	0.00%	196	0	0.00%	1.00 [0e+00-Inf]	1.000	1.000
MLST8	101	0	0.00%	196	0	0.00%	1.00 [0e+00-Inf]	1.000	1.000
MMD	101	0	0.00%	196	0	0.00%	1.00 [0e+00-Inf]	1.000	1.000
MMP16	101	0	0.00%	196	0	0.00%	1.00 [0e+00-Inf]	1.000	1.000
MMP24	101	0	0.00%	196	0	0.00%	1.00 [0e+00-Inf]	1.000	1.000
MMP25	101	0	0.00%	196	0	0.00%	1.00 [0e+00-Inf]	1.000	1.000
MMP28	101	0	0.00%	196	0	0.00%	1.00 [0e+00-Inf]	1.000	1.000
MMP9	101	0	0.00%	196	0	0.00%	1.00 [0e+00-Inf]	1.000	1.000
MNDA	101	0	0.00%	196	0	0.00%	1.00 [0e+00-Inf]	1.000	1.000
MOCS3	101	0	0.00%	196	0	0.00%	1.00 [0e+00-Inf]	1.000	1.000
MOGAT2	101	0	0.00%	196	0	0.00%	1.00 [0e+00-Inf]	1.000	1.000
MON2	101	0	0.00%	196	0	0.00%	1.00 [0e+00-Inf]	1.000	1.000
MARC1	101	0	0.00%	196	0	0.00%	1.00 [0e+00-Inf]	1.000	1.000
MARC2	101	0	0.00%	196	0	0.00%	1.00 [0e+00-Inf]	1.000	1.000
MOS	101	0	0.00%	196	0	0.00%	1.00 [0e+00-Inf]	1.000	1.000
MPG	101	0	0.00%	196	0	0.00%	1.00 [0e+00-Inf]	1.000	1.000
MPO	101	0	0.00%	196	0	0.00%	1.00 [0e+00-Inf]	1.000	1.000
MPP6	101	0	0.00%	196	0	0.00%	1.00 [0e+00-Inf]	1.000	1.000
MPV17L	101	0	0.00%	196	0	0.00%	1.00 [0e+00-Inf]	1.000	1.000
MPZL1	101	0	0.00%	196	0	0.00%	1.00 [0e+00-Inf]	1.000	1.000
MPZ	101	0	0.00%	196	0	0.00%	1.00 [0e+00-Inf]	1.000	1.000

Symbol	CINSARC Low			CINSARC High			CINSARC High vs. Low		
	N tested	N altered	Fq alteration	N tested	N altered	Fq alteration	OR [95%CI]	p-value*	q-value
MR1	101	0	0.00%	196	0	0.00%	1.00 [0e+00-Inf]	1.000	1.000
MRC2	101	0	0.00%	196	0	0.00%	1.00 [0e+00-Inf]	1.000	1.000
MRGPRD	101	0	0.00%	196	0	0.00%	1.00 [0e+00-Inf]	1.000	1.000
MRGPRF	101	0	0.00%	196	0	0.00%	1.00 [0e+00-Inf]	1.000	1.000
MRGPRX1	101	1	0.99%	196	1	0.51%	0.51 [0.05-5.30]	0.638	1.000
MRM1	101	0	0.00%	196	0	0.00%	1.00 [0e+00-Inf]	1.000	1.000
MRPL10	101	0	0.00%	196	0	0.00%	1.00 [0e+00-Inf]	1.000	1.000
MRPL11	101	0	0.00%	196	0	0.00%	1.00 [0e+00-Inf]	1.000	1.000
MRPL12	101	0	0.00%	196	0	0.00%	1.00 [0e+00-Inf]	1.000	1.000
MRPL13	101	0	0.00%	196	0	0.00%	1.00 [0e+00-Inf]	1.000	1.000
MRPL15	101	0	0.00%	196	0	0.00%	1.00 [0e+00-Inf]	1.000	1.000
MRPL21	101	0	0.00%	196	0	0.00%	1.00 [0e+00-Inf]	1.000	1.000
MRPL24	101	0	0.00%	196	0	0.00%	1.00 [0e+00-Inf]	1.000	1.000
MRPL27	101	0	0.00%	196	0	0.00%	1.00 [0e+00-Inf]	1.000	1.000
MRPL28	101	0	0.00%	196	0	0.00%	1.00 [0e+00-Inf]	1.000	1.000
MRPL38	101	0	0.00%	196	0	0.00%	1.00 [0e+00-Inf]	1.000	1.000
MRPL45	101	0	0.00%	196	0	0.00%	1.00 [0e+00-Inf]	1.000	1.000
MRPL46	101	0	0.00%	196	0	0.00%	1.00 [0e+00-Inf]	1.000	1.000
MRPL47	101	0	0.00%	196	0	0.00%	1.00 [0e+00-Inf]	1.000	1.000
MRPL48	101	0	0.00%	196	0	0.00%	1.00 [0e+00-Inf]	1.000	1.000
MRPL55	101	0	0.00%	196	0	0.00%	1.00 [0e+00-Inf]	1.000	1.000
MRPL9	101	0	0.00%	196	0	0.00%	1.00 [0e+00-Inf]	1.000	1.000
MRPS11	101	0	0.00%	196	0	0.00%	1.00 [0e+00-Inf]	1.000	1.000
MRPS14	101	0	0.00%	196	0	0.00%	1.00 [0e+00-Inf]	1.000	1.000
MRPS16	101	0	0.00%	196	0	0.00%	1.00 [0e+00-Inf]	1.000	1.000
MRPS21	101	0	0.00%	196	0	0.00%	1.00 [0e+00-Inf]	1.000	1.000
MRPS23	101	0	0.00%	196	0	0.00%	1.00 [0e+00-Inf]	1.000	1.000
MRPS28	101	0	0.00%	196	0	0.00%	1.00 [0e+00-Inf]	1.000	1.000
MRPS34	101	0	0.00%	196	0	0.00%	1.00 [0e+00-Inf]	1.000	1.000
MRPS7	101	0	0.00%	196	0	0.00%	1.00 [0e+00-Inf]	1.000	1.000
MRS2P2	101	0	0.00%	196	0	0.00%	1.00 [0e+00-Inf]	1.000	1.000
MSC	101	0	0.00%	196	0	0.00%	1.00 [0e+00-Inf]	1.000	1.000
MSI2	101	0	0.00%	196	0	0.00%	1.00 [0e+00-Inf]	1.000	1.000
MSL1	101	0	0.00%	196	0	0.00%	1.00 [0e+00-Inf]	1.000	1.000
MSLN	101	0	0.00%	196	0	0.00%	1.00 [0e+00-Inf]	1.000	1.000
MSRB3	101	0	0.00%	196	0	0.00%	1.00 [0e+00-Inf]	1.000	1.000
MSTO1	101	0	0.00%	196	0	0.00%	1.00 [0e+00-Inf]	1.000	1.000
MSTO2P	101	0	0.00%	196	0	0.00%	1.00 [0e+00-Inf]	1.000	1.000
MSX2P1	101	0	0.00%	196	0	0.00%	1.00 [0e+00-Inf]	1.000	1.000
MTBP	101	0	0.00%	196	0	0.00%	1.00 [0e+00-Inf]	1.000	1.000
MTDH	101	0	0.00%	196	0	0.00%	1.00 [0e+00-Inf]	1.000	1.000
MTFR1	101	0	0.00%	196	0	0.00%	1.00 [0e+00-Inf]	1.000	1.000
MTHFD2L	101	0	0.00%	196	0	0.00%	1.00 [0e+00-Inf]	1.000	1.000
MTMR11	101	0	0.00%	196	0	0.00%	1.00 [0e+00-Inf]	1.000	1.000
MTMR4	101	0	0.00%	196	0	0.00%	1.00 [0e+00-Inf]	1.000	1.000
MTR	101	0	0.00%	196	0	0.00%	1.00 [0e+00-Inf]	1.000	1.000
MTSS1	101	0	0.00%	196	0	0.00%	1.00 [0e+00-Inf]	1.000	1.000
MTVR2	101	0	0.00%	196	0	0.00%	1.00 [0e+00-Inf]	1.000	1.000
MTX1	101	0	0.00%	196	0	0.00%	1.00 [0e+00-Inf]	1.000	1.000
MUC1	101	0	0.00%	196	0	0.00%	1.00 [0e+00-Inf]	1.000	1.000
MUC20	101	0	0.00%	196	0	0.00%	1.00 [0e+00-Inf]	1.000	1.000
MUC4	101	0	0.00%	196	0	0.00%	1.00 [0e+00-Inf]	1.000	1.000
MVP	101	0	0.00%	196	0	0.00%	1.00 [0e+00-Inf]	1.000	1.000
MXRA7	101	0	0.00%	196	0	0.00%	1.00 [0e+00-Inf]	1.000	1.000
MYADML2	101	0	0.00%	196	0	0.00%	1.00 [0e+00-Inf]	1.000	1.000
MYBL1	101	0	0.00%	196	0	0.00%	1.00 [0e+00-Inf]	1.000	1.000
MYBL2	101	0	0.00%	196	0	0.00%	1.00 [0e+00-Inf]	1.000	1.000
MYBPH	101	0	0.00%	196	0	0.00%	1.00 [0e+00-Inf]	1.000	1.000
MYCBPAP	101	0	0.00%	196	0	0.00%	1.00 [0e+00-Inf]	1.000	1.000
MYC	101	0	0.00%	196	0	0.00%	1.00 [0e+00-Inf]	1.000	1.000
MYEOV	101	0	0.00%	196	0	0.00%	1.00 [0e+00-Inf]	1.000	1.000
MYF5	101	0	0.00%	196	0	0.00%	1.00 [0e+00-Inf]	1.000	1.000
MYF6	101	0	0.00%	196	0	0.00%	1.00 [0e+00-Inf]	1.000	1.000
MYH11	101	0	0.00%	196	0	0.00%	1.00 [0e+00-Inf]	1.000	1.000
MYH7B	101	0	0.00%	196	0	0.00%	1.00 [0e+00-Inf]	1.000	1.000
MYL4	101	0	0.00%	196	0	0.00%	1.00 [0e+00-Inf]	1.000	1.000
MYL9	101	0	0.00%	196	0	0.00%	1.00 [0e+00-Inf]	1.000	1.000
MYLPF	101	0	0.00%	196	0	0.00%	1.00 [0e+00-Inf]	1.000	1.000
MYO15B	101	0	0.00%	196	0	0.00%	1.00 [0e+00-Inf]	1.000	1.000
MYO16	101	0	0.00%	196	0	0.00%	1.00 [0e+00-Inf]	1.000	1.000
MYO18A	101	0	0.00%	196	0	0.00%	1.00 [0e+00-Inf]	1.000	1.000

Symbol	CINSARC Low			CINSARC High			CINSARC High vs. Low		
	N tested	N altered	Fq alteration	N tested	N altered	Fq alteration	OR [95%CI]	p-value*	q-value
MYO19	101	0	0.00%	196	0	0.00%	1.00 [0e+00-Inf]	1.000	1.000
MYO1D	101	0	0.00%	196	0	0.00%	1.00 [0e+00-Inf]	1.000	1.000
MYO7A	101	0	0.00%	196	0	0.00%	1.00 [0e+00-Inf]	1.000	1.000
MYOC	101	0	0.00%	196	0	0.00%	1.00 [0e+00-Inf]	1.000	1.000
MYOD1	101	0	0.00%	196	0	0.00%	1.00 [0e+00-Inf]	1.000	1.000
MYOG	101	0	0.00%	196	0	0.00%	1.00 [0e+00-Inf]	1.000	1.000
MYOZ1	101	0	0.00%	196	0	0.00%	1.00 [0e+00-Inf]	1.000	1.000
KAT8	101	0	0.00%	196	0	0.00%	1.00 [0e+00-Inf]	1.000	1.000
KAT7	101	0	0.00%	196	0	0.00%	1.00 [0e+00-Inf]	1.000	1.000
KAT6A	101	0	0.00%	196	0	0.00%	1.00 [0e+00-Inf]	1.000	1.000
KAT6B	101	0	0.00%	196	0	0.00%	1.00 [0e+00-Inf]	1.000	1.000
MYT1	101	0	0.00%	196	0	0.00%	1.00 [0e+00-Inf]	1.000	1.000
NAAA	101	0	0.00%	196	0	0.00%	1.00 [0e+00-Inf]	1.000	1.000
NAALAD2	101	0	0.00%	196	0	0.00%	1.00 [0e+00-Inf]	1.000	1.000
NACA2	101	0	0.00%	196	0	0.00%	1.00 [0e+00-Inf]	1.000	1.000
NADSYN1	101	0	0.00%	196	0	0.00%	1.00 [0e+00-Inf]	1.000	1.000
NAGPA	101	0	0.00%	196	0	0.00%	1.00 [0e+00-Inf]	1.000	1.000
NAIP	101	0	0.00%	196	0	0.00%	1.00 [0e+00-Inf]	1.000	1.000
NAP1L1	101	0	0.00%	196	0	0.00%	1.00 [0e+00-Inf]	1.000	1.000
NARF	101	0	0.00%	196	0	0.00%	1.00 [0e+00-Inf]	1.000	1.000
NARS2	101	0	0.00%	196	0	0.00%	1.00 [0e+00-Inf]	1.000	1.000
NAT14	101	0	0.00%	196	0	0.00%	1.00 [0e+00-Inf]	1.000	1.000
NAA60	101	0	0.00%	196	0	0.00%	1.00 [0e+00-Inf]	1.000	1.000
NAT9	101	0	0.00%	196	0	0.00%	1.00 [0e+00-Inf]	1.000	1.000
NAV1	101	0	0.00%	196	0	0.00%	1.00 [0e+00-Inf]	1.000	1.000
NAV3	101	0	0.00%	196	0	0.00%	1.00 [0e+00-Inf]	1.000	1.000
NBN	101	0	0.00%	196	0	0.00%	1.00 [0e+00-Inf]	1.000	1.000
NBPF10	101	0	0.00%	196	0	0.00%	1.00 [0e+00-Inf]	1.000	1.000
NBPF14	101	0	0.00%	196	0	0.00%	1.00 [0e+00-Inf]	1.000	1.000
NBPF15	101	0	0.00%	196	0	0.00%	1.00 [0e+00-Inf]	1.000	1.000
NBPF7	101	0	0.00%	196	0	0.00%	1.00 [0e+00-Inf]	1.000	1.000
NBPF9	101	0	0.00%	196	0	0.00%	1.00 [0e+00-Inf]	1.000	1.000
NCALD	101	0	0.00%	196	0	0.00%	1.00 [0e+00-Inf]	1.000	1.000
NCBP2	101	0	0.00%	196	0	0.00%	1.00 [0e+00-Inf]	1.000	1.000
NCF2	101	0	0.00%	196	0	0.00%	1.00 [0e+00-Inf]	1.000	1.000
NCOA2	101	0	0.00%	196	0	0.00%	1.00 [0e+00-Inf]	1.000	1.000
NCOA3	101	0	0.00%	196	0	0.00%	1.00 [0e+00-Inf]	1.000	1.000
NCOA5	101	0	0.00%	196	0	0.00%	1.00 [0e+00-Inf]	1.000	1.000
NCOA6	101	0	0.00%	196	0	0.00%	1.00 [0e+00-Inf]	1.000	1.000
LINC00029	101	0	0.00%	196	0	0.00%	1.00 [0e+00-Inf]	1.000	1.000
LINC00051	101	0	0.00%	196	0	0.00%	1.00 [0e+00-Inf]	1.000	1.000
LINC00052	101	0	0.00%	196	0	0.00%	1.00 [0e+00-Inf]	1.000	1.000
FBXL19-AS1	101	0	0.00%	196	0	0.00%	1.00 [0e+00-Inf]	1.000	1.000
LINC00685	101	0	0.00%	196	0	0.00%	1.00 [0e+00-Inf]	1.000	1.000
CRYM-AS1	101	0	0.00%	196	0	0.00%	1.00 [0e+00-Inf]	1.000	1.000
TTTY14	101	64	63.37%	196	116	59.18%	0.84 [0.55-1.27]	0.485	1.000
FAM224B	101	65	64.36%	196	117	59.69%	0.82 [0.54-1.25]	0.435	1.000
LINC00235	101	0	0.00%	196	0	0.00%	1.00 [0e+00-Inf]	1.000	1.000
NCSTN	101	0	0.00%	196	0	0.00%	1.00 [0e+00-Inf]	1.000	1.000
NDE1	101	0	0.00%	196	0	0.00%	1.00 [0e+00-Inf]	1.000	1.000
NDRG1	101	0	0.00%	196	0	0.00%	1.00 [0e+00-Inf]	1.000	1.000
NDRG3	101	0	0.00%	196	0	0.00%	1.00 [0e+00-Inf]	1.000	1.000
NDST2	101	0	0.00%	196	0	0.00%	1.00 [0e+00-Inf]	1.000	1.000
NDUFAB1	101	0	0.00%	196	0	0.00%	1.00 [0e+00-Inf]	1.000	1.000
NDUFB10	101	0	0.00%	196	0	0.00%	1.00 [0e+00-Inf]	1.000	1.000
NDUFB2	101	0	0.00%	196	0	0.00%	1.00 [0e+00-Inf]	1.000	1.000
NDUFB5	101	0	0.00%	196	0	0.00%	1.00 [0e+00-Inf]	1.000	1.000
NDUFB9	101	0	0.00%	196	0	0.00%	1.00 [0e+00-Inf]	1.000	1.000
NDUFC2	101	0	0.00%	196	0	0.00%	1.00 [0e+00-Inf]	1.000	1.000
NDUFS2	101	0	0.00%	196	0	0.00%	1.00 [0e+00-Inf]	1.000	1.000
NDUFS8	101	0	0.00%	196	0	0.00%	1.00 [0e+00-Inf]	1.000	1.000
NDUFV1	101	0	0.00%	196	0	0.00%	1.00 [0e+00-Inf]	1.000	1.000
NECAB1	101	0	0.00%	196	0	0.00%	1.00 [0e+00-Inf]	1.000	1.000
NEK2	101	0	0.00%	196	0	0.00%	1.00 [0e+00-Inf]	1.000	1.000
NEK7	101	0	0.00%	196	0	0.00%	1.00 [0e+00-Inf]	1.000	1.000
NEK8	101	0	0.00%	196	0	0.00%	1.00 [0e+00-Inf]	1.000	1.000
NENF	101	0	0.00%	196	0	0.00%	1.00 [0e+00-Inf]	1.000	1.000
NES	101	0	0.00%	196	0	0.00%	1.00 [0e+00-Inf]	1.000	1.000
NEU3	101	0	0.00%	196	0	0.00%	1.00 [0e+00-Inf]	1.000	1.000
NEURL2	101	0	0.00%	196	0	0.00%	1.00 [0e+00-Inf]	1.000	1.000
NEUROD2	101	0	0.00%	196	0	0.00%	1.00 [0e+00-Inf]	1.000	1.000

Symbol	CINSARC Low			CINSARC High			CINSARC High vs. Low		
	N tested	N altered	Fq alteration	N tested	N altered	Fq alteration	OR [95%CI]	p-value*	q-value
NEUROD6	101	0	0.00%	196	0	0.00%	1.00 [0e+00-Inf]	1.000	1.000
NF1	101	0	0.00%	196	0	0.00%	1.00 [0e+00-Inf]	1.000	1.000
NFASC	101	0	0.00%	196	0	0.00%	1.00 [0e+00-Inf]	1.000	1.000
NFATC2IP	101	0	0.00%	196	0	0.00%	1.00 [0e+00-Inf]	1.000	1.000
NFATC2	101	0	0.00%	196	0	0.00%	1.00 [0e+00-Inf]	1.000	1.000
NFE2L1	101	0	0.00%	196	0	0.00%	1.00 [0e+00-Inf]	1.000	1.000
NFE2L3	101	0	0.00%	196	0	0.00%	1.00 [0e+00-Inf]	1.000	1.000
NFKBIA	101	0	0.00%	196	0	0.00%	1.00 [0e+00-Inf]	1.000	1.000
TONSL	101	1	0.99%	196	0	0.00%	1.6e-08 [0e+00-Inf]	0.996	1.000
NFS1	101	0	0.00%	196	0	0.00%	1.00 [0e+00-Inf]	1.000	1.000
NGFR	101	0	0.00%	196	0	0.00%	1.00 [0e+00-Inf]	1.000	1.000
NGRN	101	0	0.00%	196	0	0.00%	1.00 [0e+00-Inf]	1.000	1.000
NHLH1	101	0	0.00%	196	0	0.00%	1.00 [0e+00-Inf]	1.000	1.000
NHLRC4	101	0	0.00%	196	0	0.00%	1.00 [0e+00-Inf]	1.000	1.000
NID1	101	0	0.00%	196	0	0.00%	1.00 [0e+00-Inf]	1.000	1.000
NIPAL2	101	0	0.00%	196	0	0.00%	1.00 [0e+00-Inf]	1.000	1.000
NIT1	101	0	0.00%	196	0	0.00%	1.00 [0e+00-Inf]	1.000	1.000
NKAIN3	101	0	0.00%	196	0	0.00%	1.00 [0e+00-Inf]	1.000	1.000
NKAIN4	101	0	0.00%	196	0	0.00%	1.00 [0e+00-Inf]	1.000	1.000
NKX2-1	101	0	0.00%	196	0	0.00%	1.00 [0e+00-Inf]	1.000	1.000
NKX2-5	101	0	0.00%	196	0	0.00%	1.00 [0e+00-Inf]	1.000	1.000
NKX2-8	101	0	0.00%	196	0	0.00%	1.00 [0e+00-Inf]	1.000	1.000
NKX6-3	101	0	0.00%	196	0	0.00%	1.00 [0e+00-Inf]	1.000	1.000
NLE1	101	0	0.00%	196	0	0.00%	1.00 [0e+00-Inf]	1.000	1.000
NLGN1	101	0	0.00%	196	0	0.00%	1.00 [0e+00-Inf]	1.000	1.000
NLGN4Y	101	64	63.37%	196	115	58.67%	0.82 [0.54-1.24]	0.434	1.000
NLK	101	0	0.00%	196	0	0.00%	1.00 [0e+00-Inf]	1.000	1.000
NLRC3	101	0	0.00%	196	0	0.00%	1.00 [0e+00-Inf]	1.000	1.000
NLRP11	101	0	0.00%	196	0	0.00%	1.00 [0e+00-Inf]	1.000	1.000
NLRP13	101	0	0.00%	196	0	0.00%	1.00 [0e+00-Inf]	1.000	1.000
NLRP3	101	0	0.00%	196	0	0.00%	1.00 [0e+00-Inf]	1.000	1.000
NLRP4	101	0	0.00%	196	0	0.00%	1.00 [0e+00-Inf]	1.000	1.000
NLRP8	101	0	0.00%	196	0	0.00%	1.00 [0e+00-Inf]	1.000	1.000
NLRP9	101	0	0.00%	196	0	0.00%	1.00 [0e+00-Inf]	1.000	1.000
NMB	101	0	0.00%	196	0	0.00%	1.00 [0e+00-Inf]	1.000	1.000
NME1-NME2	101	0	0.00%	196	0	0.00%	1.00 [0e+00-Inf]	1.000	1.000
NME1	101	0	0.00%	196	0	0.00%	1.00 [0e+00-Inf]	1.000	1.000
NME2	101	0	0.00%	196	0	0.00%	1.00 [0e+00-Inf]	1.000	1.000
NME3	101	0	0.00%	196	0	0.00%	1.00 [0e+00-Inf]	1.000	1.000
NME4	101	0	0.00%	196	0	0.00%	1.00 [0e+00-Inf]	1.000	1.000
NME7	101	0	0.00%	196	0	0.00%	1.00 [0e+00-Inf]	1.000	1.000
NMNAT2	101	0	0.00%	196	0	0.00%	1.00 [0e+00-Inf]	1.000	1.000
NMRAL1	101	0	0.00%	196	0	0.00%	1.00 [0e+00-Inf]	1.000	1.000
NNAT	101	0	0.00%	196	0	0.00%	1.00 [0e+00-Inf]	1.000	1.000
NOD1	101	0	0.00%	196	0	0.00%	1.00 [0e+00-Inf]	1.000	1.000
NOG	101	0	0.00%	196	0	0.00%	1.00 [0e+00-Inf]	1.000	1.000
NOL11	101	0	0.00%	196	0	0.00%	1.00 [0e+00-Inf]	1.000	1.000
NOM1	101	0	0.00%	196	0	0.00%	1.00 [0e+00-Inf]	1.000	1.000
NOMO1	101	0	0.00%	196	0	0.00%	1.00 [0e+00-Inf]	1.000	1.000
NOMO2	101	0	0.00%	196	0	0.00%	1.00 [0e+00-Inf]	1.000	1.000
NOMO3	101	0	0.00%	196	0	0.00%	1.00 [0e+00-Inf]	1.000	1.000
NOS1AP	101	0	0.00%	196	0	0.00%	1.00 [0e+00-Inf]	1.000	1.000
NOS2	101	0	0.00%	196	0	0.00%	1.00 [0e+00-Inf]	1.000	1.000
NOTUM	101	0	0.00%	196	0	0.00%	1.00 [0e+00-Inf]	1.000	1.000
NOVA1	101	0	0.00%	196	0	0.00%	1.00 [0e+00-Inf]	1.000	1.000
NOXO1	101	0	0.00%	196	0	0.00%	1.00 [0e+00-Inf]	1.000	1.000
NPAS3	101	0	0.00%	196	0	0.00%	1.00 [0e+00-Inf]	1.000	1.000
NPAS4	101	0	0.00%	196	0	0.00%	1.00 [0e+00-Inf]	1.000	1.000
NPBWR1	101	0	0.00%	196	0	0.00%	1.00 [0e+00-Inf]	1.000	1.000
NPBWR2	101	0	0.00%	196	0	0.00%	1.00 [0e+00-Inf]	1.000	1.000
NPB	101	0	0.00%	196	0	0.00%	1.00 [0e+00-Inf]	1.000	1.000
NPEPL1	101	0	0.00%	196	0	0.00%	1.00 [0e+00-Inf]	1.000	1.000
NPEPPS	101	0	0.00%	196	0	0.00%	1.00 [0e+00-Inf]	1.000	1.000
NPHS2	101	0	0.00%	196	0	0.00%	1.00 [0e+00-Inf]	1.000	1.000
NPIPB3	101	1	0.99%	196	0	0.00%	1.6e-08 [0e+00-Inf]	0.996	1.000
NPIPA1	101	0	0.00%	196	0	0.00%	1.00 [0e+00-Inf]	1.000	1.000
NPLOC4	101	0	0.00%	196	0	0.00%	1.00 [0e+00-Inf]	1.000	1.000
NPL	101	0	0.00%	196	0	0.00%	1.00 [0e+00-Inf]	1.000	1.000
NPR1	101	0	0.00%	196	0	0.00%	1.00 [0e+00-Inf]	1.000	1.000
NPRL3	101	0	0.00%	196	0	0.00%	1.00 [0e+00-Inf]	1.000	1.000
NPSR1	101	0	0.00%	196	0	0.00%	1.00 [0e+00-Inf]	1.000	1.000

Symbol	CINSARC Low			CINSARC High			CINSARC High vs. Low		
	N tested	N altered	Fq alteration	N tested	N altered	Fq alteration	OR [95%CI]	p-value*	q-value
NPTX1	101	0	0.00%	196	0	0.00%	1.00 [0e+00-Inf]	1.000	1.000
NPVF	101	0	0.00%	196	0	0.00%	1.00 [0e+00-Inf]	1.000	1.000
NPW	101	0	0.00%	196	0	0.00%	1.00 [0e+00-Inf]	1.000	1.000
NPY	101	0	0.00%	196	0	0.00%	1.00 [0e+00-Inf]	1.000	1.000
NR1D1	101	0	0.00%	196	0	0.00%	1.00 [0e+00-Inf]	1.000	1.000
NR1I3	101	0	0.00%	196	0	0.00%	1.00 [0e+00-Inf]	1.000	1.000
NR2E1	101	0	0.00%	196	0	0.00%	1.00 [0e+00-Inf]	1.000	1.000
NR2F2	101	0	0.00%	196	0	0.00%	1.00 [0e+00-Inf]	1.000	1.000
NR5A2	101	0	0.00%	196	0	0.00%	1.00 [0e+00-Inf]	1.000	1.000
NRBP2	101	0	0.00%	196	0	0.00%	1.00 [0e+00-Inf]	1.000	1.000
NRG1	101	0	0.00%	196	0	0.00%	1.00 [0e+00-Inf]	1.000	1.000
NSL1	101	0	0.00%	196	0	0.00%	1.00 [0e+00-Inf]	1.000	1.000
NSMAF	101	0	0.00%	196	0	0.00%	1.00 [0e+00-Inf]	1.000	1.000
NSMCE1	101	0	0.00%	196	0	0.00%	1.00 [0e+00-Inf]	1.000	1.000
NSMCE2	101	0	0.00%	196	0	0.00%	1.00 [0e+00-Inf]	1.000	1.000
NT5C3A	101	0	0.00%	196	0	0.00%	1.00 [0e+00-Inf]	1.000	1.000
NT5C	101	0	0.00%	196	0	0.00%	1.00 [0e+00-Inf]	1.000	1.000
NTAN1	101	0	0.00%	196	0	0.00%	1.00 [0e+00-Inf]	1.000	1.000
NTHL1	101	0	0.00%	196	0	0.00%	1.00 [0e+00-Inf]	1.000	1.000
NTN3	101	0	0.00%	196	0	0.00%	1.00 [0e+00-Inf]	1.000	1.000
NTRK1	101	0	0.00%	196	0	0.00%	1.00 [0e+00-Inf]	1.000	1.000
NTRK3	101	0	0.00%	196	0	0.00%	1.00 [0e+00-Inf]	1.000	1.000
NTSR1	101	0	0.00%	196	0	0.00%	1.00 [0e+00-Inf]	1.000	1.000
NUAK2	101	0	0.00%	196	0	0.00%	1.00 [0e+00-Inf]	1.000	1.000
NUBP1	101	0	0.00%	196	0	0.00%	1.00 [0e+00-Inf]	1.000	1.000
NUBP2	101	0	0.00%	196	0	0.00%	1.00 [0e+00-Inf]	1.000	1.000
NUBPL	101	0	0.00%	196	0	0.00%	1.00 [0e+00-Inf]	1.000	1.000
NUCB2	101	0	0.00%	196	0	0.00%	1.00 [0e+00-Inf]	1.000	1.000
NUCKS1	101	0	0.00%	196	0	0.00%	1.00 [0e+00-Inf]	1.000	1.000
NUDCD1	101	0	0.00%	196	0	0.00%	1.00 [0e+00-Inf]	1.000	1.000
NUDCD3	101	0	0.00%	196	0	0.00%	1.00 [0e+00-Inf]	1.000	1.000
NUDT13	101	0	0.00%	196	0	0.00%	1.00 [0e+00-Inf]	1.000	1.000
NUDT16L1	101	0	0.00%	196	0	0.00%	1.00 [0e+00-Inf]	1.000	1.000
NUDT17	101	0	0.00%	196	0	0.00%	1.00 [0e+00-Inf]	1.000	1.000
NUDT4	101	0	0.00%	196	0	0.00%	1.00 [0e+00-Inf]	1.000	1.000
NUDT8	101	0	0.00%	196	0	0.00%	1.00 [0e+00-Inf]	1.000	1.000
NUF2	101	0	0.00%	196	0	0.00%	1.00 [0e+00-Inf]	1.000	1.000
NUFIP2	101	0	0.00%	196	0	0.00%	1.00 [0e+00-Inf]	1.000	1.000
NUMA1	101	0	0.00%	196	0	0.00%	1.00 [0e+00-Inf]	1.000	1.000
NUP107	101	0	0.00%	196	0	0.00%	1.00 [0e+00-Inf]	1.000	1.000
NUP133	101	0	0.00%	196	0	0.00%	1.00 [0e+00-Inf]	1.000	1.000
NUP210L	101	0	0.00%	196	0	0.00%	1.00 [0e+00-Inf]	1.000	1.000
NUP54	101	0	0.00%	196	0	0.00%	1.00 [0e+00-Inf]	1.000	1.000
NUP85	101	0	0.00%	196	0	0.00%	1.00 [0e+00-Inf]	1.000	1.000
NUPL2	101	0	0.00%	196	0	0.00%	1.00 [0e+00-Inf]	1.000	1.000
NUPR1	101	0	0.00%	196	0	0.00%	1.00 [0e+00-Inf]	1.000	1.000
NVL	101	0	0.00%	196	0	0.00%	1.00 [0e+00-Inf]	1.000	1.000
NXPH3	101	0	0.00%	196	0	0.00%	1.00 [0e+00-Inf]	1.000	1.000
OAZ3	101	0	0.00%	196	0	0.00%	1.00 [0e+00-Inf]	1.000	1.000
OBSCN	101	0	0.00%	196	0	0.00%	1.00 [0e+00-Inf]	1.000	1.000
OC90	101	0	0.00%	196	0	0.00%	1.00 [0e+00-Inf]	1.000	1.000
OCLM	101	0	0.00%	196	0	0.00%	1.00 [0e+00-Inf]	1.000	1.000
ODF1	101	0	0.00%	196	0	0.00%	1.00 [0e+00-Inf]	1.000	1.000
TENM4	101	0	0.00%	196	0	0.00%	1.00 [0e+00-Inf]	1.000	1.000
OGFR	101	0	0.00%	196	0	0.00%	1.00 [0e+00-Inf]	1.000	1.000
OLFML2B	101	0	0.00%	196	0	0.00%	1.00 [0e+00-Inf]	1.000	1.000
OMP	101	0	0.00%	196	0	0.00%	1.00 [0e+00-Inf]	1.000	1.000
OPA1	101	0	0.00%	196	0	0.00%	1.00 [0e+00-Inf]	1.000	1.000
OPLAH	101	0	0.00%	196	0	0.00%	1.00 [0e+00-Inf]	1.000	1.000
OPN3	101	0	0.00%	196	0	0.00%	1.00 [0e+00-Inf]	1.000	1.000
OPRK1	101	0	0.00%	196	0	0.00%	1.00 [0e+00-Inf]	1.000	1.000
OPRL1	101	0	0.00%	196	0	0.00%	1.00 [0e+00-Inf]	1.000	1.000
OPTC	101	0	0.00%	196	0	0.00%	1.00 [0e+00-Inf]	1.000	1.000
OR10J1	101	0	0.00%	196	0	0.00%	1.00 [0e+00-Inf]	1.000	1.000
OR10J3	101	0	0.00%	196	0	0.00%	1.00 [0e+00-Inf]	1.000	1.000
OR10J5	101	0	0.00%	196	0	0.00%	1.00 [0e+00-Inf]	1.000	1.000
OR10K1	101	0	0.00%	196	0	0.00%	1.00 [0e+00-Inf]	1.000	1.000
OR10K2	101	0	0.00%	196	0	0.00%	1.00 [0e+00-Inf]	1.000	1.000
OR10R2	101	0	0.00%	196	0	0.00%	1.00 [0e+00-Inf]	1.000	1.000
OR10T2	101	0	0.00%	196	0	0.00%	1.00 [0e+00-Inf]	1.000	1.000
OR10X1	101	0	0.00%	196	0	0.00%	1.00 [0e+00-Inf]	1.000	1.000

Symbol	CINSARC Low			CINSARC High			CINSARC High vs. Low		
	N tested	N altered	Fq alteration	N tested	N altered	Fq alteration	OR [95%CI]	p-value*	q-value
OR10Z1	101	0	0.00%	196	0	0.00%	1.00 [0e+00-Inf]	1.000	1.000
OR11L1	101	0	0.00%	196	0	0.00%	1.00 [0e+00-Inf]	1.000	1.000
OR13G1	101	0	0.00%	196	0	0.00%	1.00 [0e+00-Inf]	1.000	1.000
OR14A16	101	0	0.00%	196	0	0.00%	1.00 [0e+00-Inf]	1.000	1.000
OR14C36	101	0	0.00%	196	0	0.00%	1.00 [0e+00-Inf]	1.000	1.000
OR14I1	101	0	0.00%	196	0	0.00%	1.00 [0e+00-Inf]	1.000	1.000
OR1C1	101	0	0.00%	196	0	0.00%	1.00 [0e+00-Inf]	1.000	1.000
OR1F1	101	0	0.00%	196	0	0.00%	1.00 [0e+00-Inf]	1.000	1.000
OR1F2P	101	0	0.00%	196	0	0.00%	1.00 [0e+00-Inf]	1.000	1.000
OR2A12	101	0	0.00%	196	0	0.00%	1.00 [0e+00-Inf]	1.000	1.000
OR2A14	101	0	0.00%	196	0	0.00%	1.00 [0e+00-Inf]	1.000	1.000
OR2A1	101	0	0.00%	196	0	0.00%	1.00 [0e+00-Inf]	1.000	1.000
OR2A25	101	0	0.00%	196	0	0.00%	1.00 [0e+00-Inf]	1.000	1.000
OR2A2	101	0	0.00%	196	0	0.00%	1.00 [0e+00-Inf]	1.000	1.000
OR2A5	101	0	0.00%	196	0	0.00%	1.00 [0e+00-Inf]	1.000	1.000
OR2A7	101	0	0.00%	196	0	0.00%	1.00 [0e+00-Inf]	1.000	1.000
OR2A9P	101	0	0.00%	196	0	0.00%	1.00 [0e+00-Inf]	1.000	1.000
OR2AK2	101	0	0.00%	196	0	0.00%	1.00 [0e+00-Inf]	1.000	1.000
OR2AT4	101	0	0.00%	196	0	0.00%	1.00 [0e+00-Inf]	1.000	1.000
OR2B11	101	0	0.00%	196	0	0.00%	1.00 [0e+00-Inf]	1.000	1.000
OR2C1	101	0	0.00%	196	0	0.00%	1.00 [0e+00-Inf]	1.000	1.000
OR2C3	101	0	0.00%	196	0	0.00%	1.00 [0e+00-Inf]	1.000	1.000
OR2F1	101	0	0.00%	196	0	0.00%	1.00 [0e+00-Inf]	1.000	1.000
OR2F2	101	0	0.00%	196	0	0.00%	1.00 [0e+00-Inf]	1.000	1.000
OR2G2	101	0	0.00%	196	0	0.00%	1.00 [0e+00-Inf]	1.000	1.000
OR2G3	101	0	0.00%	196	0	0.00%	1.00 [0e+00-Inf]	1.000	1.000
OR2G6	101	0	0.00%	196	0	0.00%	1.00 [0e+00-Inf]	1.000	1.000
OR2L13	101	0	0.00%	196	0	0.00%	1.00 [0e+00-Inf]	1.000	1.000
OR2L1P	101	0	0.00%	196	0	0.00%	1.00 [0e+00-Inf]	1.000	1.000
OR2L2	101	0	0.00%	196	0	0.00%	1.00 [0e+00-Inf]	1.000	1.000
OR2L3	101	0	0.00%	196	0	0.00%	1.00 [0e+00-Inf]	1.000	1.000
OR2L8	101	0	0.00%	196	0	0.00%	1.00 [0e+00-Inf]	1.000	1.000
OR2M1P	101	0	0.00%	196	0	0.00%	1.00 [0e+00-Inf]	1.000	1.000
OR2M2	101	0	0.00%	196	0	0.00%	1.00 [0e+00-Inf]	1.000	1.000
OR2M3	101	0	0.00%	196	0	0.00%	1.00 [0e+00-Inf]	1.000	1.000
OR2M4	101	0	0.00%	196	0	0.00%	1.00 [0e+00-Inf]	1.000	1.000
OR2M5	101	0	0.00%	196	0	0.00%	1.00 [0e+00-Inf]	1.000	1.000
OR2M7	101	0	0.00%	196	0	0.00%	1.00 [0e+00-Inf]	1.000	1.000
OR2T10	101	3	2.97%	196	2	1.02%	0.34 [0.074-1.53]	0.237	1.000
OR2T11	101	3	2.97%	196	2	1.02%	0.34 [0.074-1.53]	0.237	1.000
OR2T12	101	0	0.00%	196	0	0.00%	1.00 [0e+00-Inf]	1.000	1.000
OR2T1	101	0	0.00%	196	0	0.00%	1.00 [0e+00-Inf]	1.000	1.000
OR2T27	101	0	0.00%	196	0	0.00%	1.00 [0e+00-Inf]	1.000	1.000
OR2T29	101	0	0.00%	196	0	0.00%	1.00 [0e+00-Inf]	1.000	1.000
OR2T2	101	0	0.00%	196	0	0.00%	1.00 [0e+00-Inf]	1.000	1.000
OR2T33	101	0	0.00%	196	0	0.00%	1.00 [0e+00-Inf]	1.000	1.000
OR2T34	101	1	0.99%	196	0	0.00%	1.6e-08 [0e+00-Inf]	0.996	1.000
OR2T35	101	0	0.00%	196	0	0.00%	1.00 [0e+00-Inf]	1.000	1.000
OR2T3	101	0	0.00%	196	0	0.00%	1.00 [0e+00-Inf]	1.000	1.000
OR2T4	101	0	0.00%	196	0	0.00%	1.00 [0e+00-Inf]	1.000	1.000
OR2T5	101	0	0.00%	196	0	0.00%	1.00 [0e+00-Inf]	1.000	1.000
OR2T6	101	0	0.00%	196	0	0.00%	1.00 [0e+00-Inf]	1.000	1.000
OR2T8	101	0	0.00%	196	0	0.00%	1.00 [0e+00-Inf]	1.000	1.000
OR2W3	101	0	0.00%	196	0	0.00%	1.00 [0e+00-Inf]	1.000	1.000
OR2W5	101	0	0.00%	196	0	0.00%	1.00 [0e+00-Inf]	1.000	1.000
OR4C6	101	1	0.99%	196	4	2.04%	2.08 [0.33-13.25]	0.514	1.000
OR4D1	101	0	0.00%	196	0	0.00%	1.00 [0e+00-Inf]	1.000	1.000
OR4D2	101	0	0.00%	196	0	0.00%	1.00 [0e+00-Inf]	1.000	1.000
OR4F5	101	0	0.00%	196	0	0.00%	1.00 [0e+00-Inf]	1.000	1.000
OR4F6	101	0	0.00%	196	0	0.00%	1.00 [0e+00-Inf]	1.000	1.000
OR4K1	101	0	0.00%	196	0	0.00%	1.00 [0e+00-Inf]	1.000	1.000
OR4K2	101	1	0.99%	196	0	0.00%	1.6e-08 [0e+00-Inf]	0.996	1.000
OR4K5	101	0	0.00%	196	0	0.00%	1.00 [0e+00-Inf]	1.000	1.000
OR4M1	101	0	0.00%	196	0	0.00%	1.00 [0e+00-Inf]	1.000	1.000
OR4M2	101	0	0.00%	196	0	0.00%	1.00 [0e+00-Inf]	1.000	1.000
OR4N2	101	0	0.00%	196	0	0.00%	1.00 [0e+00-Inf]	1.000	1.000
OR4N3P	101	0	0.00%	196	0	0.00%	1.00 [0e+00-Inf]	1.000	1.000
OR4N4	101	0	0.00%	196	0	0.00%	1.00 [0e+00-Inf]	1.000	1.000
OR4P4	101	1	0.99%	196	4	2.04%	2.08 [0.33-13.25]	0.514	1.000
OR4Q3	101	0	0.00%	196	0	0.00%	1.00 [0e+00-Inf]	1.000	1.000
OR4S2	101	1	0.99%	196	4	2.04%	2.08 [0.33-13.25]	0.514	1.000

Symbol	CINSARC Low			CINSARC High			CINSARC High vs. Low		
	N tested	N altered	Fq alteration	N tested	N altered	Fq alteration	OR [95%CI]	p-value*	q-value
OR52N5	101	3	2.97%	196	7	3.57%	1.21 [0.38-3.83]	0.786	1.000
OR6B1	101	0	0.00%	196	0	0.00%	1.00 [0e+00-Inf]	1.000	1.000
OR6F1	101	0	0.00%	196	0	0.00%	1.00 [0e+00-Inf]	1.000	1.000
OR6K2	101	0	0.00%	196	0	0.00%	1.00 [0e+00-Inf]	1.000	1.000
OR6K3	101	0	0.00%	196	0	0.00%	1.00 [0e+00-Inf]	1.000	1.000
OR6K6	101	0	0.00%	196	0	0.00%	1.00 [0e+00-Inf]	1.000	1.000
OR6N1	101	0	0.00%	196	0	0.00%	1.00 [0e+00-Inf]	1.000	1.000
OR6N2	101	0	0.00%	196	0	0.00%	1.00 [0e+00-Inf]	1.000	1.000
OR6P1	101	0	0.00%	196	0	0.00%	1.00 [0e+00-Inf]	1.000	1.000
OR6V1	101	0	0.00%	196	0	0.00%	1.00 [0e+00-Inf]	1.000	1.000
OR6W1P	101	0	0.00%	196	0	0.00%	1.00 [0e+00-Inf]	1.000	1.000
OR6Y1	101	0	0.00%	196	0	0.00%	1.00 [0e+00-Inf]	1.000	1.000
OR9A2	101	0	0.00%	196	0	0.00%	1.00 [0e+00-Inf]	1.000	1.000
ORAI3	101	0	0.00%	196	0	0.00%	1.00 [0e+00-Inf]	1.000	1.000
ORMDL3	101	0	0.00%	196	0	0.00%	1.00 [0e+00-Inf]	1.000	1.000
OSBPL2	101	0	0.00%	196	0	0.00%	1.00 [0e+00-Inf]	1.000	1.000
OSBPL3	101	0	0.00%	196	0	0.00%	1.00 [0e+00-Inf]	1.000	1.000
OSBPL7	101	0	0.00%	196	0	0.00%	1.00 [0e+00-Inf]	1.000	1.000
OSBPL8	101	0	0.00%	196	0	0.00%	1.00 [0e+00-Inf]	1.000	1.000
OSGIN2	101	0	0.00%	196	0	0.00%	1.00 [0e+00-Inf]	1.000	1.000
OSR2	101	0	0.00%	196	0	0.00%	1.00 [0e+00-Inf]	1.000	1.000
OSTM1	101	0	0.00%	196	0	0.00%	1.00 [0e+00-Inf]	1.000	1.000
OSTN	101	0	0.00%	196	0	0.00%	1.00 [0e+00-Inf]	1.000	1.000
SLC51A	101	0	0.00%	196	0	0.00%	1.00 [0e+00-Inf]	1.000	1.000
OTOA	101	0	0.00%	196	0	0.00%	1.00 [0e+00-Inf]	1.000	1.000
OTOP2	101	0	0.00%	196	0	0.00%	1.00 [0e+00-Inf]	1.000	1.000
OTOP3	101	0	0.00%	196	0	0.00%	1.00 [0e+00-Inf]	1.000	1.000
OTUD6B	101	0	0.00%	196	0	0.00%	1.00 [0e+00-Inf]	1.000	1.000
OTUD7B	101	0	0.00%	196	0	0.00%	1.00 [0e+00-Inf]	1.000	1.000
OXR1	101	0	0.00%	196	0	0.00%	1.00 [0e+00-Inf]	1.000	1.000
P2RY12	101	0	0.00%	196	0	0.00%	1.00 [0e+00-Inf]	1.000	1.000
P2RY13	101	0	0.00%	196	0	0.00%	1.00 [0e+00-Inf]	1.000	1.000
P2RY14	101	0	0.00%	196	0	0.00%	1.00 [0e+00-Inf]	1.000	1.000
P2RY2	101	0	0.00%	196	0	0.00%	1.00 [0e+00-Inf]	1.000	1.000
P2RY6	101	0	0.00%	196	0	0.00%	1.00 [0e+00-Inf]	1.000	1.000
P4HA1	101	0	0.00%	196	0	0.00%	1.00 [0e+00-Inf]	1.000	1.000
P4HA3	101	0	0.00%	196	0	0.00%	1.00 [0e+00-Inf]	1.000	1.000
P4HB	101	0	0.00%	196	0	0.00%	1.00 [0e+00-Inf]	1.000	1.000
POTEM	101	0	0.00%	196	0	0.00%	1.00 [0e+00-Inf]	1.000	1.000
PAAF1	101	0	0.00%	196	0	0.00%	1.00 [0e+00-Inf]	1.000	1.000
PABPC1L	101	0	0.00%	196	0	0.00%	1.00 [0e+00-Inf]	1.000	1.000
PABPC1	101	0	0.00%	196	0	0.00%	1.00 [0e+00-Inf]	1.000	1.000
PACS1	101	0	0.00%	196	7	0.00%	1.00 [0e+00-Inf]	1.000	1.000
PAG1	101	0	0.00%	196	0	0.00%	1.00 [0e+00-Inf]	1.000	1.000
PAK1	101	0	0.00%	196	0	0.00%	1.00 [0e+00-Inf]	1.000	1.000
PAK2	101	0	0.00%	196	0	0.00%	1.00 [0e+00-Inf]	1.000	1.000
PALB2	101	0	0.00%	196	0	0.00%	1.00 [0e+00-Inf]	1.000	1.000
PAPPA2	101	0	0.00%	196	0	0.00%	1.00 [0e+00-Inf]	1.000	1.000
PAQR4	101	0	0.00%	196	0	0.00%	1.00 [0e+00-Inf]	1.000	1.000
PAQR6	101	0	0.00%	196	0	0.00%	1.00 [0e+00-Inf]	1.000	1.000
PAWR	101	0	0.00%	196	0	0.00%	1.00 [0e+00-Inf]	1.000	1.000
PARD6B	101	0	0.00%	196	0	0.00%	1.00 [0e+00-Inf]	1.000	1.000
PARL	101	0	0.00%	196	0	0.00%	1.00 [0e+00-Inf]	1.000	1.000
PARM1	101	0	0.00%	196	0	0.00%	1.00 [0e+00-Inf]	1.000	1.000
PARN	101	0	0.00%	196	0	0.00%	1.00 [0e+00-Inf]	1.000	1.000
PARP10	101	0	0.00%	196	0	0.00%	1.00 [0e+00-Inf]	1.000	1.000
PARP1	101	0	0.00%	196	0	0.00%	1.00 [0e+00-Inf]	1.000	1.000
PARP6	101	0	0.00%	196	0	0.00%	1.00 [0e+00-Inf]	1.000	1.000
PAX9	101	0	0.00%	196	0	0.00%	1.00 [0e+00-Inf]	1.000	1.000
PBX1	101	0	0.00%	196	0	0.00%	1.00 [0e+00-Inf]	1.000	1.000
PBXIP1	101	0	0.00%	196	0	0.00%	1.00 [0e+00-Inf]	1.000	1.000
PCDH11Y	101	58	57.43%	196	102	52.04%	0.80 [0.54-1.21]	0.378	1.000
PCF11	101	0	0.00%	196	0	0.00%	1.00 [0e+00-Inf]	1.000	1.000
PCGF2	101	0	0.00%	196	0	0.00%	1.00 [0e+00-Inf]	1.000	1.000
PCIF1	101	0	0.00%	196	0	0.00%	1.00 [0e+00-Inf]	1.000	1.000
PCK1	101	0	0.00%	196	0	0.00%	1.00 [0e+00-Inf]	1.000	1.000
PCMTD1	101	0	0.00%	196	0	0.00%	1.00 [0e+00-Inf]	1.000	1.000
PCMTD2	101	0	0.00%	196	0	0.00%	1.00 [0e+00-Inf]	1.000	1.000
PCP4L1	101	0	0.00%	196	7	0.00%	1.00 [0e+00-Inf]	1.000	1.000
PCSK6	101	0	0.00%	196	0	0.00%	1.00 [0e+00-Inf]	1.000	1.000
PCTP	101	0	0.00%	196	0	0.00%	1.00 [0e+00-Inf]	1.000	1.000

Symbol	CINSARC Low			CINSARC High			CINSARC High vs. Low		
	N tested	N altered	Fq alteration	N tested	N altered	Fq alteration	OR [95%CI]	p-value*	q-value
PCYT1A	101	0	0.00%	196	0	0.00%	1.00 [0e+00-Inf]	1.000	1.000
PCYT2	101	0	0.00%	196	0	0.00%	1.00 [0e+00-Inf]	1.000	1.000
PC	101	0	0.00%	196	0	0.00%	1.00 [0e+00-Inf]	1.000	1.000
PDC	101	0	0.00%	196	0	0.00%	1.00 [0e+00-Inf]	1.000	1.000
PDE1C	101	0	0.00%	196	0	0.00%	1.00 [0e+00-Inf]	1.000	1.000
PDE2A	101	0	0.00%	196	0	0.00%	1.00 [0e+00-Inf]	1.000	1.000
PDE4DIP	101	0	0.00%	196	0	0.00%	1.00 [0e+00-Inf]	1.000	1.000
PDE6G	101	0	0.00%	196	0	0.00%	1.00 [0e+00-Inf]	1.000	1.000
PDE7A	101	0	0.00%	196	0	0.00%	1.00 [0e+00-Inf]	1.000	1.000
PDE8A	101	0	0.00%	196	0	0.00%	1.00 [0e+00-Inf]	1.000	1.000
PDIA2	101	0	0.00%	196	0	0.00%	1.00 [0e+00-Inf]	1.000	1.000
PDIA3P1	101	0	0.00%	196	0	0.00%	1.00 [0e+00-Inf]	1.000	1.000
PDILT	101	0	0.00%	196	0	0.00%	1.00 [0e+00-Inf]	1.000	1.000
PDK2	101	0	0.00%	196	0	0.00%	1.00 [0e+00-Inf]	1.000	1.000
PDP1	101	0	0.00%	196	0	0.00%	1.00 [0e+00-Inf]	1.000	1.000
PDPK1	101	0	0.00%	196	0	0.00%	1.00 [0e+00-Inf]	1.000	1.000
PDSS2	101	0	0.00%	196	0	0.00%	1.00 [0e+00-Inf]	1.000	1.000
PDXDC1	101	0	0.00%	196	0	0.00%	1.00 [0e+00-Inf]	1.000	1.000
PDZD9	101	0	0.00%	196	0	0.00%	1.00 [0e+00-Inf]	1.000	1.000
PDZK1P1	101	0	0.00%	196	0	0.00%	1.00 [0e+00-Inf]	1.000	1.000
PDZK1	101	0	0.00%	196	0	0.00%	1.00 [0e+00-Inf]	1.000	1.000
PEA15	101	0	0.00%	196	0	0.00%	1.00 [0e+00-Inf]	1.000	1.000
PEAR1	101	0	0.00%	196	0	0.00%	1.00 [0e+00-Inf]	1.000	1.000
PECAM1	101	0	0.00%	196	0	0.00%	1.00 [0e+00-Inf]	1.000	1.000
PELI3	101	0	0.00%	196	0	0.00%	1.00 [0e+00-Inf]	1.000	1.000
PENK	101	0	0.00%	196	0	0.00%	1.00 [0e+00-Inf]	1.000	1.000
PEX11A	101	0	0.00%	196	0	0.00%	1.00 [0e+00-Inf]	1.000	1.000
PEX11B	101	0	0.00%	196	0	0.00%	1.00 [0e+00-Inf]	1.000	1.000
PEX19	101	0	0.00%	196	0	0.00%	1.00 [0e+00-Inf]	1.000	1.000
PEX2	101	0	0.00%	196	0	0.00%	1.00 [0e+00-Inf]	1.000	1.000
PEX5L	101	0	0.00%	196	0	0.00%	1.00 [0e+00-Inf]	1.000	1.000
PF4V1	101	0	0.00%	196	0	0.00%	1.00 [0e+00-Inf]	1.000	1.000
PF4	101	0	0.00%	196	0	0.00%	1.00 [0e+00-Inf]	1.000	1.000
PFDN2	101	0	0.00%	196	0	0.00%	1.00 [0e+00-Inf]	1.000	1.000
PFDN4	101	0	0.00%	196	0	0.00%	1.00 [0e+00-Inf]	1.000	1.000
PFKFB2	101	0	0.00%	196	0	0.00%	1.00 [0e+00-Inf]	1.000	1.000
PGA3	101	0	0.00%	196	0	0.00%	1.00 [0e+00-Inf]	1.000	1.000
PGA4	101	0	0.00%	196	0	0.00%	1.00 [0e+00-Inf]	1.000	1.000
PGA5	101	0	0.00%	196	0	0.00%	1.00 [0e+00-Inf]	1.000	1.000
PGAP3	101	0	0.00%	196	0	0.00%	1.00 [0e+00-Inf]	1.000	1.000
PGBD2	101	0	0.00%	196	0	0.00%	1.00 [0e+00-Inf]	1.000	1.000
PGBD5	101	0	0.00%	196	0	0.00%	1.00 [0e+00-Inf]	1.000	1.000
CPQ	101	0	0.00%	196	0	0.00%	1.00 [0e+00-Inf]	1.000	1.000
PGLYRP3	101	0	0.00%	196	0	0.00%	1.00 [0e+00-Inf]	1.000	1.000
PGLYRP4	101	0	0.00%	196	0	0.00%	1.00 [0e+00-Inf]	1.000	1.000
PGM2L1	101	0	0.00%	196	0	0.00%	1.00 [0e+00-Inf]	1.000	1.000
PGPEP1L	101	0	0.00%	196	0	0.00%	1.00 [0e+00-Inf]	1.000	1.000
PGP	101	0	0.00%	196	0	0.00%	1.00 [0e+00-Inf]	1.000	1.000
PGS1	101	0	0.00%	196	0	0.00%	1.00 [0e+00-Inf]	1.000	1.000
PHACTR1	101	0	0.00%	196	0	0.00%	1.00 [0e+00-Inf]	1.000	1.000
PHACTR3	101	0	0.00%	196	0	0.00%	1.00 [0e+00-Inf]	1.000	1.000
PHB	101	0	0.00%	196	0	0.00%	1.00 [0e+00-Inf]	1.000	1.000
PHF12	101	0	0.00%	196	0	0.00%	1.00 [0e+00-Inf]	1.000	1.000
PHF20L1	101	0	0.00%	196	0	0.00%	1.00 [0e+00-Inf]	1.000	1.000
PHF20	101	0	0.00%	196	0	0.00%	1.00 [0e+00-Inf]	1.000	1.000
PHKB	101	0	0.00%	196	0	0.00%	1.00 [0e+00-Inf]	1.000	1.000
PHKG2	101	0	0.00%	196	0	0.00%	1.00 [0e+00-Inf]	1.000	1.000
PHLDA1	101	0	0.00%	196	0	0.00%	1.00 [0e+00-Inf]	1.000	1.000
PHLDA3	101	0	0.00%	196	0	0.00%	1.00 [0e+00-Inf]	1.000	1.000
PHOSPHO1	101	0	0.00%	196	0	0.00%	1.00 [0e+00-Inf]	1.000	1.000
PHOX2A	101	0	0.00%	196	0	0.00%	1.00 [0e+00-Inf]	1.000	1.000
PI15	101	0	0.00%	196	0	0.00%	1.00 [0e+00-Inf]	1.000	1.000
PI3	101	0	0.00%	196	0	0.00%	1.00 [0e+00-Inf]	1.000	1.000
PI4KB	101	0	0.00%	196	0	0.00%	1.00 [0e+00-Inf]	1.000	1.000
PIAS3	101	0	0.00%	196	0	0.00%	1.00 [0e+00-Inf]	1.000	1.000
PIGC	101	0	0.00%	196	0	0.00%	1.00 [0e+00-Inf]	1.000	1.000
PIGM	101	0	0.00%	196	0	0.00%	1.00 [0e+00-Inf]	1.000	1.000
PIGQ	101	0	0.00%	196	0	0.00%	1.00 [0e+00-Inf]	1.000	1.000
PIGR	101	0	0.00%	196	0	0.00%	1.00 [0e+00-Inf]	1.000	1.000
PIGS	101	0	0.00%	196	0	0.00%	1.00 [0e+00-Inf]	1.000	1.000
PIGT	101	0	0.00%	196	0	0.00%	1.00 [0e+00-Inf]	1.000	1.000

Symbol	CINSARC Low			CINSARC High			CINSARC High vs. Low		
	N tested	N altered	Fq alteration	N tested	N altered	Fq alteration	OR [95%CI]	p-value*	q-value
PIGU	101	0	0.00%	196	0	0.00%	1.00 [0e+00-Inf]	1.000	1.000
PIGW	101	0	0.00%	196	0	0.00%	1.00 [0e+00-Inf]	1.000	1.000
PIGX	101	0	0.00%	196	0	0.00%	1.00 [0e+00-Inf]	1.000	1.000
PIGZ	101	0	0.00%	196	0	0.00%	1.00 [0e+00-Inf]	1.000	1.000
PIK3C2B	101	0	0.00%	196	0	0.00%	1.00 [0e+00-Inf]	1.000	1.000
PIK3CA	101	0	0.00%	196	0	0.00%	1.00 [0e+00-Inf]	1.000	1.000
PIP4K2B	101	0	0.00%	196	0	0.00%	1.00 [0e+00-Inf]	1.000	1.000
PIP5K1A	101	0	0.00%	196	0	0.00%	1.00 [0e+00-Inf]	1.000	1.000
PIPOX	101	0	0.00%	196	0	0.00%	1.00 [0e+00-Inf]	1.000	1.000
PIP	101	0	0.00%	196	0	0.00%	1.00 [0e+00-Inf]	1.000	1.000
PITPNC1	101	0	0.00%	196	0	0.00%	1.00 [0e+00-Inf]	1.000	1.000
PITPNM1	101	0	0.00%	196	0	0.00%	1.00 [0e+00-Inf]	1.000	1.000
PKD1	101	0	0.00%	196	0	0.00%	1.00 [0e+00-Inf]	1.000	1.000
PKHD1L1	101	0	0.00%	196	0	0.00%	1.00 [0e+00-Inf]	1.000	1.000
PKIA	101	0	0.00%	196	0	0.00%	1.00 [0e+00-Inf]	1.000	1.000
PKIG	101	0	0.00%	196	0	0.00%	1.00 [0e+00-Inf]	1.000	1.000
PKLR	101	0	0.00%	196	0	0.00%	1.00 [0e+00-Inf]	1.000	1.000
PKMYT1	101	0	0.00%	196	0	0.00%	1.00 [0e+00-Inf]	1.000	1.000
PKP1	101	0	0.00%	196	0	0.00%	1.00 [0e+00-Inf]	1.000	1.000
PLA2G10	101	0	0.00%	196	0	0.00%	1.00 [0e+00-Inf]	1.000	1.000
PLA2G4A	101	0	0.00%	196	0	0.00%	1.00 [0e+00-Inf]	1.000	1.000
PLAG1	101	0	0.00%	196	0	0.00%	1.00 [0e+00-Inf]	1.000	1.000
PLAT	101	0	0.00%	196	0	0.00%	1.00 [0e+00-Inf]	1.000	1.000
PLAU	101	0	0.00%	196	0	0.00%	1.00 [0e+00-Inf]	1.000	1.000
PLCG1	101	0	0.00%	196	0	0.00%	1.00 [0e+00-Inf]	1.000	1.000
PLCXD1	101	0	0.00%	196	0	0.00%	1.00 [0e+00-Inf]	1.000	1.000
PLD5	101	0	0.00%	196	0	0.00%	1.00 [0e+00-Inf]	1.000	1.000
PLEC	101	0	0.00%	196	0	0.00%	1.00 [0e+00-Inf]	1.000	1.000
PLEKHA2	101	0	0.00%	196	0	0.00%	1.00 [0e+00-Inf]	1.000	1.000
PLEKHA6	101	0	0.00%	196	0	0.00%	1.00 [0e+00-Inf]	1.000	1.000
PLEKHA7	101	0	0.00%	196	0	0.00%	1.00 [0e+00-Inf]	1.000	1.000
PLEKHA8	101	0	0.00%	196	0	0.00%	1.00 [0e+00-Inf]	1.000	1.000
PLEKHB1	101	0	0.00%	196	0	0.00%	1.00 [0e+00-Inf]	1.000	1.000
PLEKHF2	101	0	0.00%	196	0	0.00%	1.00 [0e+00-Inf]	1.000	1.000
PLEKHG7	101	0	0.00%	196	0	0.00%	1.00 [0e+00-Inf]	1.000	1.000
PLEKHO1	101	0	0.00%	196	0	0.00%	1.00 [0e+00-Inf]	1.000	1.000
PLIN1	101	0	0.00%	196	0	0.00%	1.00 [0e+00-Inf]	1.000	1.000
PLK1	101	0	0.00%	196	0	0.00%	1.00 [0e+00-Inf]	1.000	1.000
PLTP	101	0	0.00%	196	0	0.00%	1.00 [0e+00-Inf]	1.000	1.000
PLXDC1	101	0	0.00%	196	0	0.00%	1.00 [0e+00-Inf]	1.000	1.000
PLXNA2	101	0	0.00%	196	0	0.00%	1.00 [0e+00-Inf]	1.000	1.000
PM20D1	101	0	0.00%	196	0	0.00%	1.00 [0e+00-Inf]	1.000	1.000
PMAIP1	101	0	0.00%	196	0	0.00%	1.00 [0e+00-Inf]	1.000	1.000
PMEPA1	101	0	0.00%	196	0	0.00%	1.00 [0e+00-Inf]	1.000	1.000
PMF1	101	0	0.00%	196	0	0.00%	1.00 [0e+00-Inf]	1.000	1.000
PMM2	101	0	0.00%	196	0	0.00%	1.00 [0e+00-Inf]	1.000	1.000
PMP2	101	0	0.00%	196	0	0.00%	1.00 [0e+00-Inf]	1.000	1.000
PMVK	101	0	0.00%	196	0	0.00%	1.00 [0e+00-Inf]	1.000	1.000
PNMT	101	0	0.00%	196	0	0.00%	1.00 [0e+00-Inf]	1.000	1.000
PNPO	101	0	0.00%	196	0	0.00%	1.00 [0e+00-Inf]	1.000	1.000
POC1B	101	0	0.00%	196	0	0.00%	1.00 [0e+00-Inf]	1.000	1.000
POGK	101	0	0.00%	196	0	0.00%	1.00 [0e+00-Inf]	1.000	1.000
POGZ	101	0	0.00%	196	0	0.00%	1.00 [0e+00-Inf]	1.000	1.000
POLB	101	0	0.00%	196	0	0.00%	1.00 [0e+00-Inf]	1.000	1.000
POLD3	101	0	0.00%	196	0	0.00%	1.00 [0e+00-Inf]	1.000	1.000
POLD4	101	0	0.00%	196	0	0.00%	1.00 [0e+00-Inf]	1.000	1.000
POLDIP2	101	0	0.00%	196	0	0.00%	1.00 [0e+00-Inf]	1.000	1.000
POLG2	101	0	0.00%	196	0	0.00%	1.00 [0e+00-Inf]	1.000	1.000
POLG	101	0	0.00%	196	0	0.00%	1.00 [0e+00-Inf]	1.000	1.000
POLI	101	0	0.00%	196	0	0.00%	1.00 [0e+00-Inf]	1.000	1.000
POLR2H	101	0	0.00%	196	0	0.00%	1.00 [0e+00-Inf]	1.000	1.000
POLR2K	101	0	0.00%	196	0	0.00%	1.00 [0e+00-Inf]	1.000	1.000
POLR3C	101	0	0.00%	196	0	0.00%	1.00 [0e+00-Inf]	1.000	1.000
POLR3E	101	0	0.00%	196	0	0.00%	1.00 [0e+00-Inf]	1.000	1.000
POLR3GL	101	0	0.00%	196	0	0.00%	1.00 [0e+00-Inf]	1.000	1.000
POLR3K	101	0	0.00%	196	0	0.00%	1.00 [0e+00-Inf]	1.000	1.000
POM121L8P	101	0	0.00%	196	1	0.51%	3.2e+07 [0e+00-Inf]	0.997	1.000
POMP	101	0	0.00%	196	0	0.00%	1.00 [0e+00-Inf]	1.000	1.000
POP1	101	0	0.00%	196	0	0.00%	1.00 [0e+00-Inf]	1.000	1.000
POPDC3	101	0	0.00%	196	0	0.00%	1.00 [0e+00-Inf]	1.000	1.000
POTEA	101	0	0.00%	196	0	0.00%	1.00 [0e+00-Inf]	1.000	1.000

Symbol	CINSARC Low			CINSARC High			CINSARC High vs. Low		
	N tested	N altered	Fq alteration	N tested	N altered	Fq alteration	OR [95%CI]	p-value*	q-value
POTEB	101	0	0.00%	196	0	0.00%	1.00 [0e+00-Inf]	1.000	1.000
POTEG	101	0	0.00%	196	0	0.00%	1.00 [0e+00-Inf]	1.000	1.000
POU2F1	101	0	0.00%	196	0	0.00%	1.00 [0e+00-Inf]	1.000	1.000
POU5F1B	101	0	0.00%	196	0	0.00%	1.00 [0e+00-Inf]	1.000	1.000
PPBPP2	101	0	0.00%	196	0	0.00%	1.00 [0e+00-Inf]	1.000	1.000
PPBP	101	0	0.00%	196	0	0.00%	1.00 [0e+00-Inf]	1.000	1.000
PPDPF	101	0	0.00%	196	0	0.00%	1.00 [0e+00-Inf]	1.000	1.000
PPEF2	101	0	0.00%	196	0	0.00%	1.00 [0e+00-Inf]	1.000	1.000
PPFIA1	101	0	0.00%	196	0	0.00%	1.00 [0e+00-Inf]	1.000	1.000
PPFIA2	101	0	0.00%	196	0	0.00%	1.00 [0e+00-Inf]	1.000	1.000
PPFIA4	101	0	0.00%	196	0	0.00%	1.00 [0e+00-Inf]	1.000	1.000
PPIAL4D	101	0	0.00%	196	0	0.00%	1.00 [0e+00-Inf]	1.000	1.000
PPIAL4E	101	0	0.00%	196	0	0.00%	1.00 [0e+00-Inf]	1.000	1.000
PPL	101	0	0.00%	196	0	0.00%	1.00 [0e+00-Inf]	1.000	1.000
PPM1D	101	0	0.00%	196	0	0.00%	1.00 [0e+00-Inf]	1.000	1.000
PPM1E	101	0	0.00%	196	0	0.00%	1.00 [0e+00-Inf]	1.000	1.000
PPM1H	101	0	0.00%	196	0	0.00%	1.00 [0e+00-Inf]	1.000	1.000
PPME1	101	0	0.00%	196	0	0.00%	1.00 [0e+00-Inf]	1.000	1.000
PPOX	101	0	0.00%	196	0	0.00%	1.00 [0e+00-Inf]	1.000	1.000
PPP1CA	101	0	0.00%	196	0	0.00%	1.00 [0e+00-Inf]	1.000	1.000
PPP1R12A	101	0	0.00%	196	0	0.00%	1.00 [0e+00-Inf]	1.000	1.000
PPP1R12B	101	0	0.00%	196	0	0.00%	1.00 [0e+00-Inf]	1.000	1.000
PPP1R15B	101	0	0.00%	196	0	0.00%	1.00 [0e+00-Inf]	1.000	1.000
PPP1R16A	101	0	0.00%	196	0	0.00%	1.00 [0e+00-Inf]	1.000	1.000
PPP1R16B	101	0	0.00%	196	0	0.00%	1.00 [0e+00-Inf]	1.000	1.000
PPP1R1B	101	0	0.00%	196	0	0.00%	1.00 [0e+00-Inf]	1.000	1.000
PPP1R2	101	0	0.00%	196	0	0.00%	1.00 [0e+00-Inf]	1.000	1.000
PPP1R3D	101	0	0.00%	196	0	0.00%	1.00 [0e+00-Inf]	1.000	1.000
PPP1R9B	101	0	0.00%	196	0	0.00%	1.00 [0e+00-Inf]	1.000	1.000
PPP2CB	101	0	0.00%	196	0	0.00%	1.00 [0e+00-Inf]	1.000	1.000
PPP2R3B	101	0	0.00%	196	0	0.00%	1.00 [0e+00-Inf]	1.000	1.000
PPP2R3C	101	0	0.00%	196	0	0.00%	1.00 [0e+00-Inf]	1.000	1.000
PPP2R5A	101	0	0.00%	196	0	0.00%	1.00 [0e+00-Inf]	1.000	1.000
PPP3CB	101	0	0.00%	196	0	0.00%	1.00 [0e+00-Inf]	1.000	1.000
PPP4C	101	0	0.00%	196	0	0.00%	1.00 [0e+00-Inf]	1.000	1.000
PPP4R1L	101	0	0.00%	196	0	0.00%	1.00 [0e+00-Inf]	1.000	1.000
DESI2	101	0	0.00%	196	0	0.00%	1.00 [0e+00-Inf]	1.000	1.000
PRAC1	101	0	0.00%	196	0	0.00%	1.00 [0e+00-Inf]	1.000	1.000
PRCC	101	0	0.00%	196	0	0.00%	1.00 [0e+00-Inf]	1.000	1.000
PRCD	101	0	0.00%	196	0	0.00%	1.00 [0e+00-Inf]	1.000	1.000
PRCP	101	0	0.00%	196	0	0.00%	1.00 [0e+00-Inf]	1.000	1.000
PRDM14	101	0	0.00%	196	0	0.00%	1.00 [0e+00-Inf]	1.000	1.000
PRDM1	101	0	0.00%	196	0	0.00%	1.00 [0e+00-Inf]	1.000	1.000
PRDX6	101	0	0.00%	196	0	0.00%	1.00 [0e+00-Inf]	1.000	1.000
PRELP	101	0	0.00%	196	0	0.00%	1.00 [0e+00-Inf]	1.000	1.000
PREP	101	0	0.00%	196	0	0.00%	1.00 [0e+00-Inf]	1.000	1.000
PREX1	101	0	0.00%	196	0	0.00%	1.00 [0e+00-Inf]	1.000	1.000
PREX2	101	0	0.00%	196	0	0.00%	1.00 [0e+00-Inf]	1.000	1.000
PRG4	101	0	0.00%	196	0	0.00%	1.00 [0e+00-Inf]	1.000	1.000
HELZ2	101	0	0.00%	196	0	0.00%	1.00 [0e+00-Inf]	1.000	1.000
PRKAB2	101	0	0.00%	196	0	0.00%	1.00 [0e+00-Inf]	1.000	1.000
PRKAR1A	101	0	0.00%	196	0	0.00%	1.00 [0e+00-Inf]	1.000	1.000
PRKCA	101	0	0.00%	196	0	0.00%	1.00 [0e+00-Inf]	1.000	1.000
PRKCB	101	0	0.00%	196	0	0.00%	1.00 [0e+00-Inf]	1.000	1.000
PRKCI	101	0	0.00%	196	0	0.00%	1.00 [0e+00-Inf]	1.000	1.000
PRKD1	101	0	0.00%	196	0	0.00%	1.00 [0e+00-Inf]	1.000	1.000
PRKDC	101	0	0.00%	196	0	0.00%	1.00 [0e+00-Inf]	1.000	1.000
PRKY	101	64	63.37%	196	116	59.18%	0.84 [0.55-1.27]	0.485	1.000
PRM1	101	0	0.00%	196	0	0.00%	1.00 [0e+00-Inf]	1.000	1.000
PRM2	101	0	0.00%	196	0	0.00%	1.00 [0e+00-Inf]	1.000	1.000
PRM3	101	0	0.00%	196	0	0.00%	1.00 [0e+00-Inf]	1.000	1.000
PROCA1	101	0	0.00%	196	0	0.00%	1.00 [0e+00-Inf]	1.000	1.000
PROCR	101	0	0.00%	196	0	0.00%	1.00 [0e+00-Inf]	1.000	1.000
PROX1	101	0	0.00%	196	0	0.00%	1.00 [0e+00-Inf]	1.000	1.000
PRPF3	101	0	0.00%	196	0	0.00%	1.00 [0e+00-Inf]	1.000	1.000
PRPF6	101	0	0.00%	196	0	0.00%	1.00 [0e+00-Inf]	1.000	1.000
PRPSAP1	101	0	0.00%	196	0	0.00%	1.00 [0e+00-Inf]	1.000	1.000
PRR11	101	0	0.00%	196	0	0.00%	1.00 [0e+00-Inf]	1.000	1.000
PRR14	101	0	0.00%	196	0	0.00%	1.00 [0e+00-Inf]	1.000	1.000
PRR15L	101	0	0.00%	196	0	0.00%	1.00 [0e+00-Inf]	1.000	1.000
PRR15	101	0	0.00%	196	0	0.00%	1.00 [0e+00-Inf]	1.000	1.000

Symbol	CINSARC Low			CINSARC High			CINSARC High vs. Low		
	N tested	N altered	Fq alteration	N tested	N altered	Fq alteration	OR [95%CI]	p-value*	q-value
PRR25	101	0	0.00%	196	0	0.00%	1.00 [0e+00-Inf]	1.000	1.000
PRRT2	101	0	0.00%	196	0	0.00%	1.00 [0e+00-Inf]	1.000	1.000
PRRX1	101	0	0.00%	196	0	0.00%	1.00 [0e+00-Inf]	1.000	1.000
PRSS1	101	0	0.00%	196	0	0.00%	1.00 [0e+00-Inf]	1.000	1.000
PRSS21	101	0	0.00%	196	0	0.00%	1.00 [0e+00-Inf]	1.000	1.000
PRSS22	101	0	0.00%	196	0	0.00%	1.00 [0e+00-Inf]	1.000	1.000
PRSS23	101	0	0.00%	196	0	0.00%	1.00 [0e+00-Inf]	1.000	1.000
PRSS27	101	0	0.00%	196	0	0.00%	1.00 [0e+00-Inf]	1.000	1.000
PRSS30P	101	0	0.00%	196	0	0.00%	1.00 [0e+00-Inf]	1.000	1.000
PRSS33	101	0	0.00%	196	0	0.00%	1.00 [0e+00-Inf]	1.000	1.000
PRSS36	101	0	0.00%	196	0	0.00%	1.00 [0e+00-Inf]	1.000	1.000
PRSS38	101	0	0.00%	196	0	0.00%	1.00 [0e+00-Inf]	1.000	1.000
PRSS41	101	0	0.00%	196	0	0.00%	1.00 [0e+00-Inf]	1.000	1.000
PRSS53	101	0	0.00%	196	0	0.00%	1.00 [0e+00-Inf]	1.000	1.000
PRSS8	101	0	0.00%	196	0	0.00%	1.00 [0e+00-Inf]	1.000	1.000
PRY2	101	65	64.36%	196	117	59.69%	0.82 [0.54-1.25]	0.435	1.000
PSAP	101	0	0.00%	196	0	0.00%	1.00 [0e+00-Inf]	1.000	1.000
PSCA	101	0	0.00%	196	0	0.00%	1.00 [0e+00-Inf]	1.000	1.000
PSEN2	101	0	0.00%	196	0	0.00%	1.00 [0e+00-Inf]	1.000	1.000
PSKH2	101	0	0.00%	196	0	0.00%	1.00 [0e+00-Inf]	1.000	1.000
PSMA6	101	0	0.00%	196	0	0.00%	1.00 [0e+00-Inf]	1.000	1.000
PSMA7	101	0	0.00%	196	0	0.00%	1.00 [0e+00-Inf]	1.000	1.000
PSMB11	101	0	0.00%	196	0	0.00%	1.00 [0e+00-Inf]	1.000	1.000
PSMB3	101	0	0.00%	196	0	0.00%	1.00 [0e+00-Inf]	1.000	1.000
PSMB4	101	0	0.00%	196	0	0.00%	1.00 [0e+00-Inf]	1.000	1.000
PSMB5	101	0	0.00%	196	0	0.00%	1.00 [0e+00-Inf]	1.000	1.000
PSMC5	101	0	0.00%	196	0	0.00%	1.00 [0e+00-Inf]	1.000	1.000
PSMD11	101	0	0.00%	196	0	0.00%	1.00 [0e+00-Inf]	1.000	1.000
PSMD12	101	0	0.00%	196	0	0.00%	1.00 [0e+00-Inf]	1.000	1.000
PSMD2	101	0	0.00%	196	0	0.00%	1.00 [0e+00-Inf]	1.000	1.000
PSMD3	101	0	0.00%	196	0	0.00%	1.00 [0e+00-Inf]	1.000	1.000
PSMD4	101	0	0.00%	196	0	0.00%	1.00 [0e+00-Inf]	1.000	1.000
PTDSS1	101	0	0.00%	196	0	0.00%	1.00 [0e+00-Inf]	1.000	1.000
PTGIS	101	0	0.00%	196	0	0.00%	1.00 [0e+00-Inf]	1.000	1.000
PTGS2	101	0	0.00%	196	0	0.00%	1.00 [0e+00-Inf]	1.000	1.000
PTK2	101	0	0.00%	196	0	0.00%	1.00 [0e+00-Inf]	1.000	1.000
PTK6	101	0	0.00%	196	0	0.00%	1.00 [0e+00-Inf]	1.000	1.000
PTP4A3	101	0	0.00%	196	0	0.00%	1.00 [0e+00-Inf]	1.000	1.000
PTPN14	101	0	0.00%	196	0	0.00%	1.00 [0e+00-Inf]	1.000	1.000
PTPN1	101	0	0.00%	196	0	0.00%	1.00 [0e+00-Inf]	1.000	1.000
PTPN5	101	0	0.00%	196	0	0.00%	1.00 [0e+00-Inf]	1.000	1.000
PTPN7	101	0	0.00%	196	0	0.00%	1.00 [0e+00-Inf]	1.000	1.000
PTPRB	101	0	0.00%	196	0	0.00%	1.00 [0e+00-Inf]	1.000	1.000
PTPRCAP	101	0	0.00%	196	0	0.00%	1.00 [0e+00-Inf]	1.000	1.000
PTPRC	101	0	0.00%	196	0	0.00%	1.00 [0e+00-Inf]	1.000	1.000
PTPRR	101	0	0.00%	196	0	0.00%	1.00 [0e+00-Inf]	1.000	1.000
PTPRT	101	0	0.00%	196	0	0.00%	1.00 [0e+00-Inf]	1.000	1.000
PTPRVP	101	0	0.00%	196	0	0.00%	1.00 [0e+00-Inf]	1.000	1.000
PTRH2	101	0	0.00%	196	0	0.00%	1.00 [0e+00-Inf]	1.000	1.000
PTTG3P	101	0	0.00%	196	0	0.00%	1.00 [0e+00-Inf]	1.000	1.000
PTX4	101	0	0.00%	196	0	0.00%	1.00 [0e+00-Inf]	1.000	1.000
PUF60	101	0	0.00%	196	0	0.00%	1.00 [0e+00-Inf]	1.000	1.000
PURG	101	0	0.00%	196	0	0.00%	1.00 [0e+00-Inf]	1.000	1.000
PVT1	101	0	0.00%	196	0	0.00%	1.00 [0e+00-Inf]	1.000	1.000
PXDNL	101	0	0.00%	196	0	0.00%	1.00 [0e+00-Inf]	1.000	1.000
PYCARD	101	0	0.00%	196	0	0.00%	1.00 [0e+00-Inf]	1.000	1.000
PYCR1	101	0	0.00%	196	0	0.00%	1.00 [0e+00-Inf]	1.000	1.000
PYCR2	101	0	0.00%	196	0	0.00%	1.00 [0e+00-Inf]	1.000	1.000
PYDC1	101	0	0.00%	196	0	0.00%	1.00 [0e+00-Inf]	1.000	1.000
PYDC2	101	0	0.00%	196	0	0.00%	1.00 [0e+00-Inf]	1.000	1.000
PYGO2	101	0	0.00%	196	0	0.00%	1.00 [0e+00-Inf]	1.000	1.000
PYHIN1	101	0	0.00%	196	0	0.00%	1.00 [0e+00-Inf]	1.000	1.000
PYY2	101	0	0.00%	196	0	0.00%	1.00 [0e+00-Inf]	1.000	1.000
QPRT	101	0	0.00%	196	0	0.00%	1.00 [0e+00-Inf]	1.000	1.000
QRICH2	101	0	0.00%	196	0	0.00%	1.00 [0e+00-Inf]	1.000	1.000
QRSL1	101	0	0.00%	196	0	0.00%	1.00 [0e+00-Inf]	1.000	1.000
QSER1	101	0	0.00%	196	0	0.00%	1.00 [0e+00-Inf]	1.000	1.000
QSOX1	101	0	0.00%	196	0	0.00%	1.00 [0e+00-Inf]	1.000	1.000
R3HDML	101	0	0.00%	196	0	0.00%	1.00 [0e+00-Inf]	1.000	1.000
RAB11FIP1	101	0	0.00%	196	0	0.00%	1.00 [0e+00-Inf]	1.000	1.000
RAB11FIP3	101	0	0.00%	196	0	0.00%	1.00 [0e+00-Inf]	1.000	1.000

Symbol	CINSARC Low			CINSARC High			CINSARC High vs. Low		
	N tested	N altered	Fq alteration	N tested	N altered	Fq alteration	OR [95%CI]	p-value*	q-value
RAB13	101	0	0.00%	196	0	0.00%	1.00 [0e+00-Inf]	1.000	1.000
RAB19	101	0	0.00%	196	0	0.00%	1.00 [0e+00-Inf]	1.000	1.000
RAB1B	101	0	0.00%	196	0	0.00%	1.00 [0e+00-Inf]	1.000	1.000
RAB21	101	0	0.00%	196	0	0.00%	1.00 [0e+00-Inf]	1.000	1.000
RAB22A	101	0	0.00%	196	0	0.00%	1.00 [0e+00-Inf]	1.000	1.000
RAB25	101	0	0.00%	196	0	0.00%	1.00 [0e+00-Inf]	1.000	1.000
RAB26	101	0	0.00%	196	0	0.00%	1.00 [0e+00-Inf]	1.000	1.000
RAB27B	101	0	0.00%	196	0	0.00%	1.00 [0e+00-Inf]	1.000	1.000
RAB2A	101	0	0.00%	196	0	0.00%	1.00 [0e+00-Inf]	1.000	1.000
RAB30	101	0	0.00%	196	0	0.00%	1.00 [0e+00-Inf]	1.000	1.000
RAB34	101	0	0.00%	196	0	0.00%	1.00 [0e+00-Inf]	1.000	1.000
RAB37	101	0	0.00%	196	0	0.00%	1.00 [0e+00-Inf]	1.000	1.000
RAB38	101	0	0.00%	196	0	0.00%	1.00 [0e+00-Inf]	1.000	1.000
RAB3GAP2	101	0	0.00%	196	0	0.00%	1.00 [0e+00-Inf]	1.000	1.000
RAB3IP	101	0	0.00%	196	0	0.00%	1.00 [0e+00-Inf]	1.000	1.000
RAB40B	101	0	0.00%	196	0	0.00%	1.00 [0e+00-Inf]	1.000	1.000
RAB40C	101	0	0.00%	196	0	0.00%	1.00 [0e+00-Inf]	1.000	1.000
RAB4A	101	0	0.00%	196	0	0.00%	1.00 [0e+00-Inf]	1.000	1.000
RAB6A	101	0	0.00%	196	0	0.00%	1.00 [0e+00-Inf]	1.000	1.000
RABEP2	101	0	0.00%	196	0	0.00%	1.00 [0e+00-Inf]	1.000	1.000
RABGAP1L	101	0	0.00%	196	0	0.00%	1.00 [0e+00-Inf]	1.000	1.000
RABIF	101	0	0.00%	196	0	0.00%	1.00 [0e+00-Inf]	1.000	1.000
RAC3	101	0	0.00%	196	0	0.00%	1.00 [0e+00-Inf]	1.000	1.000
RAD21	101	0	0.00%	196	0	0.00%	1.00 [0e+00-Inf]	1.000	1.000
RAD51C	101	0	0.00%	196	0	0.00%	1.00 [0e+00-Inf]	1.000	1.000
RAD51D	101	0	0.00%	196	0	0.00%	1.00 [0e+00-Inf]	1.000	1.000
RAD54B	101	0	0.00%	196	0	0.00%	1.00 [0e+00-Inf]	1.000	1.000
RAD9A	101	0	0.00%	196	0	0.00%	1.00 [0e+00-Inf]	1.000	1.000
RAE1	101	0	0.00%	196	0	0.00%	1.00 [0e+00-Inf]	1.000	1.000
SLC50A1	101	0	0.00%	196	0	0.00%	1.00 [0e+00-Inf]	1.000	1.000
RALA	101	0	0.00%	196	0	0.00%	1.00 [0e+00-Inf]	1.000	1.000
RALGAPA1	101	0	0.00%	196	0	0.00%	1.00 [0e+00-Inf]	1.000	1.000
RALGAPB	101	0	0.00%	196	0	0.00%	1.00 [0e+00-Inf]	1.000	1.000
RALGPS2	101	0	0.00%	196	0	0.00%	1.00 [0e+00-Inf]	1.000	1.000
RALYL	101	0	0.00%	196	0	0.00%	1.00 [0e+00-Inf]	1.000	1.000
RAP1B	101	0	0.00%	196	0	0.00%	1.00 [0e+00-Inf]	1.000	1.000
RAPGEF5	101	0	0.00%	196	0	0.00%	1.00 [0e+00-Inf]	1.000	1.000
RAPGEFL1	101	0	0.00%	196	0	0.00%	1.00 [0e+00-Inf]	1.000	1.000
RARA	101	0	0.00%	196	0	0.00%	1.00 [0e+00-Inf]	1.000	1.000
RASAL2	101	0	0.00%	196	0	0.00%	1.00 [0e+00-Inf]	1.000	1.000
RASL10B	101	0	0.00%	196	0	0.00%	1.00 [0e+00-Inf]	1.000	1.000
RASSF3	101	0	0.00%	196	0	0.00%	1.00 [0e+00-Inf]	1.000	1.000
RASSF5	101	0	0.00%	196	0	0.00%	1.00 [0e+00-Inf]	1.000	1.000
RASSF6	101	0	0.00%	196	0	0.00%	1.00 [0e+00-Inf]	1.000	1.000
RAX	101	0	0.00%	196	0	0.00%	1.00 [0e+00-Inf]	1.000	1.000
RB1CC1	101	0	0.00%	196	0	0.00%	1.00 [0e+00-Inf]	1.000	1.000
RBBP5	101	0	0.00%	196	0	0.00%	1.00 [0e+00-Inf]	1.000	1.000
RBBP6	101	0	0.00%	196	0	0.00%	1.00 [0e+00-Inf]	1.000	1.000
RBL1	101	0	0.00%	196	0	0.00%	1.00 [0e+00-Inf]	1.000	1.000
RBM12B	101	0	0.00%	196	0	0.00%	1.00 [0e+00-Inf]	1.000	1.000
RBM12	101	0	0.00%	196	0	0.00%	1.00 [0e+00-Inf]	1.000	1.000
RBM14	101	0	0.00%	196	0	0.00%	1.00 [0e+00-Inf]	1.000	1.000
RBM34	101	0	0.00%	196	0	0.00%	1.00 [0e+00-Inf]	1.000	1.000
RBM38	101	0	0.00%	196	0	0.00%	1.00 [0e+00-Inf]	1.000	1.000
RBM39	101	0	0.00%	196	0	0.00%	1.00 [0e+00-Inf]	1.000	1.000
RBM4B	101	0	0.00%	196	0	0.00%	1.00 [0e+00-Inf]	1.000	1.000
RBM4	101	0	0.00%	196	0	0.00%	1.00 [0e+00-Inf]	1.000	1.000
RBM8A	101	0	0.00%	196	0	0.00%	1.00 [0e+00-Inf]	1.000	1.000
RBMY1A1	101	52	51.49%	196	98	50.00%	0.94 [0.63-1.41]	0.808	1.000
RBMY1A3P	101	64	63.37%	196	116	59.18%	0.84 [0.55-1.27]	0.485	1.000
RBMY1B	101	52	51.49%	196	98	50.00%	0.94 [0.63-1.41]	0.808	1.000
RBMY1E	101	52	51.49%	196	98	50.00%	0.94 [0.63-1.41]	0.808	1.000
RBMY1F	101	65	64.36%	196	117	59.69%	0.82 [0.54-1.25]	0.435	1.000
RBMY1J	101	65	64.36%	196	117	59.69%	0.82 [0.54-1.25]	0.435	1.000
RBMY2EP	101	64	63.37%	196	116	59.18%	0.84 [0.55-1.27]	0.485	1.000
RBMY2FP	101	65	64.36%	196	117	59.69%	0.82 [0.54-1.25]	0.435	1.000
RBMY3AP	101	64	63.37%	196	115	58.67%	0.82 [0.54-1.24]	0.434	1.000
RBPJL	101	0	0.00%	196	0	0.00%	1.00 [0e+00-Inf]	1.000	1.000
RC3H1	101	0	0.00%	196	0	0.00%	1.00 [0e+00-Inf]	1.000	1.000
RCE1	101	0	0.00%	196	0	0.00%	1.00 [0e+00-Inf]	1.000	1.000
RCHY1	101	0	0.00%	196	0	0.00%	1.00 [0e+00-Inf]	1.000	1.000

Symbol	CINSARC Low			CINSARC High			CINSARC High vs. Low		
	N tested	N altered	Fq alteration	N tested	N altered	Fq alteration	OR [95%CI]	p-value*	q-value
RCOR3	101	0	0.00%	196	0	0.00%	1.00 [0e+00-Inf]	1.000	1.000
RCSD1	101	0	0.00%	196	0	0.00%	1.00 [0e+00-Inf]	1.000	1.000
RD3	101	0	0.00%	196	0	0.00%	1.00 [0e+00-Inf]	1.000	1.000
RDH10	101	0	0.00%	196	0	0.00%	1.00 [0e+00-Inf]	1.000	1.000
RDM1	101	0	0.00%	196	0	0.00%	1.00 [0e+00-Inf]	1.000	1.000
RECQL4	101	0	0.00%	196	0	0.00%	1.00 [0e+00-Inf]	1.000	1.000
RECQL5	101	0	0.00%	196	0	0.00%	1.00 [0e+00-Inf]	1.000	1.000
RELT	101	0	0.00%	196	0	0.00%	1.00 [0e+00-Inf]	1.000	1.000
REN	101	0	0.00%	196	0	0.00%	1.00 [0e+00-Inf]	1.000	1.000
RFFL	101	0	0.00%	196	0	0.00%	1.00 [0e+00-Inf]	1.000	1.000
RFNG	101	0	0.00%	196	0	0.00%	1.00 [0e+00-Inf]	1.000	1.000
RFPL4A	101	0	0.00%	196	0	0.00%	1.00 [0e+00-Inf]	1.000	1.000
RFX5	101	0	0.00%	196	0	0.00%	1.00 [0e+00-Inf]	1.000	1.000
RGL1	101	0	0.00%	196	0	0.00%	1.00 [0e+00-Inf]	1.000	1.000
RGMA	101	0	0.00%	196	0	0.00%	1.00 [0e+00-Inf]	1.000	1.000
RGS11	101	0	0.00%	196	0	0.00%	1.00 [0e+00-Inf]	1.000	1.000
RGS13	101	0	0.00%	196	0	0.00%	1.00 [0e+00-Inf]	1.000	1.000
RGS16	101	0	0.00%	196	0	0.00%	1.00 [0e+00-Inf]	1.000	1.000
RGS18	101	0	0.00%	196	0	0.00%	1.00 [0e+00-Inf]	1.000	1.000
RGS19	101	0	0.00%	196	0	0.00%	1.00 [0e+00-Inf]	1.000	1.000
RGS1	101	0	0.00%	196	0	0.00%	1.00 [0e+00-Inf]	1.000	1.000
RGS20	101	0	0.00%	196	0	0.00%	1.00 [0e+00-Inf]	1.000	1.000
RGS21	101	0	0.00%	196	0	0.00%	1.00 [0e+00-Inf]	1.000	1.000
RGS22	101	0	0.00%	196	0	0.00%	1.00 [0e+00-Inf]	1.000	1.000
RGS2	101	0	0.00%	196	0	0.00%	1.00 [0e+00-Inf]	1.000	1.000
RGS4	101	0	0.00%	196	0	0.00%	1.00 [0e+00-Inf]	1.000	1.000
RGS5	101	0	0.00%	196	0	0.00%	1.00 [0e+00-Inf]	1.000	1.000
RGS7	101	0	0.00%	196	0	0.00%	1.00 [0e+00-Inf]	1.000	1.000
RGS8	101	0	0.00%	196	0	0.00%	1.00 [0e+00-Inf]	1.000	1.000
RGS9	101	0	0.00%	196	0	0.00%	1.00 [0e+00-Inf]	1.000	1.000
RGSL1	101	0	0.00%	196	0	0.00%	1.00 [0e+00-Inf]	1.000	1.000
RHBDF1	101	0	0.00%	196	0	0.00%	1.00 [0e+00-Inf]	1.000	1.000
RHBDF2	101	0	0.00%	196	0	0.00%	1.00 [0e+00-Inf]	1.000	1.000
RHBDL1	101	0	0.00%	196	0	0.00%	1.00 [0e+00-Inf]	1.000	1.000
RHBG	101	0	0.00%	196	0	0.00%	1.00 [0e+00-Inf]	1.000	1.000
RHCG	101	0	0.00%	196	0	0.00%	1.00 [0e+00-Inf]	1.000	1.000
RHOD	101	0	0.00%	196	0	0.00%	1.00 [0e+00-Inf]	1.000	1.000
RHOT2	101	0	0.00%	196	0	0.00%	1.00 [0e+00-Inf]	1.000	1.000
RHOU	101	0	0.00%	196	0	0.00%	1.00 [0e+00-Inf]	1.000	1.000
RHPN1	101	0	0.00%	196	0	0.00%	1.00 [0e+00-Inf]	1.000	1.000
RIMS2	101	0	0.00%	196	0	0.00%	1.00 [0e+00-Inf]	1.000	1.000
RIMS4	101	0	0.00%	196	0	0.00%	1.00 [0e+00-Inf]	1.000	1.000
RIN1	101	0	0.00%	196	0	0.00%	1.00 [0e+00-Inf]	1.000	1.000
RIPK2	101	0	0.00%	196	0	0.00%	1.00 [0e+00-Inf]	1.000	1.000
RIT1	101	0	0.00%	196	0	0.00%	1.00 [0e+00-Inf]	1.000	1.000
RLBP1	101	0	0.00%	196	0	0.00%	1.00 [0e+00-Inf]	1.000	1.000
RNASEL	101	0	0.00%	196	0	0.00%	1.00 [0e+00-Inf]	1.000	1.000
RNF112	101	0	0.00%	196	0	0.00%	1.00 [0e+00-Inf]	1.000	1.000
RNF114	101	0	0.00%	196	0	0.00%	1.00 [0e+00-Inf]	1.000	1.000
RNF115	101	0	0.00%	196	0	0.00%	1.00 [0e+00-Inf]	1.000	1.000
RNF121	101	0	0.00%	196	0	0.00%	1.00 [0e+00-Inf]	1.000	1.000
RNF122	101	0	0.00%	196	0	0.00%	1.00 [0e+00-Inf]	1.000	1.000
RNF126P1	101	0	0.00%	196	0	0.00%	1.00 [0e+00-Inf]	1.000	1.000
RNF135	101	0	0.00%	196	0	0.00%	1.00 [0e+00-Inf]	1.000	1.000
RNF139	101	0	0.00%	196	0	0.00%	1.00 [0e+00-Inf]	1.000	1.000
RNF151	101	0	0.00%	196	0	0.00%	1.00 [0e+00-Inf]	1.000	1.000
RNF157	101	0	0.00%	196	0	0.00%	1.00 [0e+00-Inf]	1.000	1.000
RNF168	101	0	0.00%	196	0	0.00%	1.00 [0e+00-Inf]	1.000	1.000
RNF169	101	0	0.00%	196	0	0.00%	1.00 [0e+00-Inf]	1.000	1.000
RNF170	101	0	0.00%	196	0	0.00%	1.00 [0e+00-Inf]	1.000	1.000
RNF187	101	0	0.00%	196	0	0.00%	1.00 [0e+00-Inf]	1.000	1.000
RNF19A	101	0	0.00%	196	0	0.00%	1.00 [0e+00-Inf]	1.000	1.000
RNF213	101	0	0.00%	196	0	0.00%	1.00 [0e+00-Inf]	1.000	1.000
RNF2	101	0	0.00%	196	0	0.00%	1.00 [0e+00-Inf]	1.000	1.000
RNF40	101	0	0.00%	196	0	0.00%	1.00 [0e+00-Inf]	1.000	1.000
RNF43	101	0	0.00%	196	0	0.00%	1.00 [0e+00-Inf]	1.000	1.000
RNFT1	101	0	0.00%	196	0	0.00%	1.00 [0e+00-Inf]	1.000	1.000
RNPEP	101	0	0.00%	196	0	0.00%	1.00 [0e+00-Inf]	1.000	1.000
RNPS1	101	0	0.00%	196	0	0.00%	1.00 [0e+00-Inf]	1.000	1.000
LAMTOR2	101	0	0.00%	196	0	0.00%	1.00 [0e+00-Inf]	1.000	1.000
ROGDI	101	0	0.00%	196	0	0.00%	1.00 [0e+00-Inf]	1.000	1.000

Symbol	CINSARC Low			CINSARC High			CINSARC High vs. Low		
	N tested	N altered	Fq alteration	N tested	N altered	Fq alteration	OR [95%CI]	p-value*	q-value
ROMO1	101	0	0.00%	196	0	0.00%	1.00 [0e+00-Inf]	1.000	1.000
RORC	101	0	0.00%	196	0	0.00%	1.00 [0e+00-Inf]	1.000	1.000
RP1L1	101	1	0.99%	196	0	0.00%	1.6e-08 [0e+00-Inf]	0.996	1.000
RP1	101	0	0.00%	196	0	0.00%	1.00 [0e+00-Inf]	1.000	1.000
RP9P	101	0	0.00%	196	0	0.00%	1.00 [0e+00-Inf]	1.000	1.000
RP9	101	0	0.00%	196	0	0.00%	1.00 [0e+00-Inf]	1.000	1.000
RPL19	101	0	0.00%	196	0	0.00%	1.00 [0e+00-Inf]	1.000	1.000
RPL23A	101	0	0.00%	196	0	0.00%	1.00 [0e+00-Inf]	1.000	1.000
RPL23P8	101	0	0.00%	196	0	0.00%	1.00 [0e+00-Inf]	1.000	1.000
RPL23	101	0	0.00%	196	0	0.00%	1.00 [0e+00-Inf]	1.000	1.000
RPL28	101	0	0.00%	196	0	0.00%	1.00 [0e+00-Inf]	1.000	1.000
RPL30	101	0	0.00%	196	0	0.00%	1.00 [0e+00-Inf]	1.000	1.000
RPL31P11	101	0	0.00%	196	0	0.00%	1.00 [0e+00-Inf]	1.000	1.000
RPL35A	101	0	0.00%	196	0	0.00%	1.00 [0e+00-Inf]	1.000	1.000
RPL38	101	0	0.00%	196	0	0.00%	1.00 [0e+00-Inf]	1.000	1.000
RPL3L	101	0	0.00%	196	0	0.00%	1.00 [0e+00-Inf]	1.000	1.000
RPL7	101	0	0.00%	196	0	0.00%	1.00 [0e+00-Inf]	1.000	1.000
RPL8	101	0	0.00%	196	0	0.00%	1.00 [0e+00-Inf]	1.000	1.000
RPLP0P2	101	0	0.00%	196	0	0.00%	1.00 [0e+00-Inf]	1.000	1.000
RPN2	101	0	0.00%	196	0	0.00%	1.00 [0e+00-Inf]	1.000	1.000
RPRD1B	101	0	0.00%	196	0	0.00%	1.00 [0e+00-Inf]	1.000	1.000
RPRD2	101	0	0.00%	196	0	0.00%	1.00 [0e+00-Inf]	1.000	1.000
RPRML	101	0	0.00%	196	0	0.00%	1.00 [0e+00-Inf]	1.000	1.000
RPS10P7	101	0	0.00%	196	0	0.00%	1.00 [0e+00-Inf]	1.000	1.000
RPS15A	101	0	0.00%	196	0	0.00%	1.00 [0e+00-Inf]	1.000	1.000
RPS17	101	0	0.00%	196	0	0.00%	1.00 [0e+00-Inf]	1.000	1.000
RPS20	101	0	0.00%	196	0	0.00%	1.00 [0e+00-Inf]	1.000	1.000
RPS21	101	0	0.00%	196	0	0.00%	1.00 [0e+00-Inf]	1.000	1.000
RPS27	101	0	0.00%	196	0	0.00%	1.00 [0e+00-Inf]	1.000	1.000
RPS2P32	101	0	0.00%	196	0	0.00%	1.00 [0e+00-Inf]	1.000	1.000
RPS2	101	0	0.00%	196	0	0.00%	1.00 [0e+00-Inf]	1.000	1.000
RPS3	101	0	0.00%	196	0	0.00%	1.00 [0e+00-Inf]	1.000	1.000
RPS4Y1	101	62	61.39%	196	108	55.10%	0.77 [0.51-1.16]	0.300	1.000
RPS4Y2	101	64	63.37%	196	116	59.18%	0.84 [0.55-1.27]	0.485	1.000
RPS6KB1	101	0	0.00%	196	0	0.00%	1.00 [0e+00-Inf]	1.000	1.000
RPS6KB2	101	0	0.00%	196	0	0.00%	1.00 [0e+00-Inf]	1.000	1.000
RPS6KC1	101	0	0.00%	196	0	0.00%	1.00 [0e+00-Inf]	1.000	1.000
RPSAP52	101	0	0.00%	196	1	0.51%	3.2e+07 [0e+00-Inf]	0.997	1.000
RPTN	101	0	0.00%	196	0	0.00%	1.00 [0e+00-Inf]	1.000	1.000
RPTOR	101	0	0.00%	196	0	0.00%	1.00 [0e+00-Inf]	1.000	1.000
RPUSD1	101	0	0.00%	196	0	0.00%	1.00 [0e+00-Inf]	1.000	1.000
RRM2B	101	0	0.00%	196	0	0.00%	1.00 [0e+00-Inf]	1.000	1.000
RRN3P1	101	0	0.00%	196	0	0.00%	1.00 [0e+00-Inf]	1.000	1.000
RRN3P2	101	0	0.00%	196	0	0.00%	1.00 [0e+00-Inf]	1.000	1.000
RRN3P3	101	0	0.00%	196	0	0.00%	1.00 [0e+00-Inf]	1.000	1.000
RRN3	101	0	0.00%	196	0	0.00%	1.00 [0e+00-Inf]	1.000	1.000
RRP15	101	0	0.00%	196	0	0.00%	1.00 [0e+00-Inf]	1.000	1.000
RRS1	101	0	0.00%	196	0	0.00%	1.00 [0e+00-Inf]	1.000	1.000
RSAD1	101	0	0.00%	196	0	0.00%	1.00 [0e+00-Inf]	1.000	1.000
RSF1	101	0	0.00%	196	0	0.00%	1.00 [0e+00-Inf]	1.000	1.000
RSL1D1	101	0	0.00%	196	0	0.00%	1.00 [0e+00-Inf]	1.000	1.000
RSPO2	101	0	0.00%	196	0	0.00%	1.00 [0e+00-Inf]	1.000	1.000
RTEL1	101	0	0.00%	196	0	0.00%	1.00 [0e+00-Inf]	1.000	1.000
RTN4IP1	101	0	0.00%	196	0	0.00%	1.00 [0e+00-Inf]	1.000	1.000
SNX29	101	0	0.00%	196	0	0.00%	1.00 [0e+00-Inf]	1.000	1.000
SNX29P2	101	0	0.00%	196	0	0.00%	1.00 [0e+00-Inf]	1.000	1.000
RUNX1T1	101	0	0.00%	196	0	0.00%	1.00 [0e+00-Inf]	1.000	1.000
RUSC1	101	0	0.00%	196	0	0.00%	1.00 [0e+00-Inf]	1.000	1.000
RXFP4	101	0	0.00%	196	0	0.00%	1.00 [0e+00-Inf]	1.000	1.000
RXRG	101	0	0.00%	196	0	0.00%	1.00 [0e+00-Inf]	1.000	1.000
RYR2	101	0	0.00%	196	0	0.00%	1.00 [0e+00-Inf]	1.000	1.000
S100A10	101	0	0.00%	196	0	0.00%	1.00 [0e+00-Inf]	1.000	1.000
S100A11	101	0	0.00%	196	0	0.00%	1.00 [0e+00-Inf]	1.000	1.000
S100A12	101	0	0.00%	196	0	0.00%	1.00 [0e+00-Inf]	1.000	1.000
S100A13	101	0	0.00%	196	0	0.00%	1.00 [0e+00-Inf]	1.000	1.000
S100A14	101	0	0.00%	196	0	0.00%	1.00 [0e+00-Inf]	1.000	1.000
S100A16	101	0	0.00%	196	0	0.00%	1.00 [0e+00-Inf]	1.000	1.000
S100A1	101	0	0.00%	196	0	0.00%	1.00 [0e+00-Inf]	1.000	1.000
S100A2	101	0	0.00%	196	0	0.00%	1.00 [0e+00-Inf]	1.000	1.000
S100A3	101	0	0.00%	196	0	0.00%	1.00 [0e+00-Inf]	1.000	1.000
S100A4	101	0	0.00%	196	0	0.00%	1.00 [0e+00-Inf]	1.000	1.000

Symbol	CINSARC Low			CINSARC High			CINSARC High vs. Low		
	N tested	N altered	Fq alteration	N tested	N altered	Fq alteration	OR [95%CI]	p-value*	q-value
S100A5	101	0	0.00%	196	0	0.00%	1.00 [0e+00-Inf]	1.000	1.000
S100A6	101	0	0.00%	196	0	0.00%	1.00 [0e+00-Inf]	1.000	1.000
S100A7A	101	0	0.00%	196	0	0.00%	1.00 [0e+00-Inf]	1.000	1.000
S100A7L2	101	0	0.00%	196	0	0.00%	1.00 [0e+00-Inf]	1.000	1.000
S100A7	101	0	0.00%	196	0	0.00%	1.00 [0e+00-Inf]	1.000	1.000
S100A8	101	0	0.00%	196	0	0.00%	1.00 [0e+00-Inf]	1.000	1.000
S100A9	101	0	0.00%	196	0	0.00%	1.00 [0e+00-Inf]	1.000	1.000
SALL4	101	0	0.00%	196	0	0.00%	1.00 [0e+00-Inf]	1.000	1.000
SAMD10	101	0	0.00%	196	0	0.00%	1.00 [0e+00-Inf]	1.000	1.000
SAMD12	101	0	0.00%	196	0	0.00%	1.00 [0e+00-Inf]	1.000	1.000
SAMD14	101	0	0.00%	196	0	0.00%	1.00 [0e+00-Inf]	1.000	1.000
SAMD8	101	0	0.00%	196	0	0.00%	1.00 [0e+00-Inf]	1.000	1.000
SAMHD1	101	0	0.00%	196	0	0.00%	1.00 [0e+00-Inf]	1.000	1.000
SAP30BP	101	0	0.00%	196	0	0.00%	1.00 [0e+00-Inf]	1.000	1.000
PPP6R3	101	0	0.00%	196	0	0.00%	1.00 [0e+00-Inf]	1.000	1.000
SARM1	101	0	0.00%	196	0	0.00%	1.00 [0e+00-Inf]	1.000	1.000
SBF1P1	101	0	0.00%	196	0	0.00%	1.00 [0e+00-Inf]	1.000	1.000
SBK1	101	0	0.00%	196	0	0.00%	1.00 [0e+00-Inf]	1.000	1.000
SBK2	101	0	0.00%	196	0	0.00%	1.00 [0e+00-Inf]	1.000	1.000
SCAMP3	101	0	0.00%	196	0	0.00%	1.00 [0e+00-Inf]	1.000	1.000
SCAND1	101	0	0.00%	196	0	0.00%	1.00 [0e+00-Inf]	1.000	1.000
SCAND2P	101	0	0.00%	196	0	0.00%	1.00 [0e+00-Inf]	1.000	1.000
SCARB2	101	0	0.00%	196	0	0.00%	1.00 [0e+00-Inf]	1.000	1.000
SCARNA15	101	0	0.00%	196	0	0.00%	1.00 [0e+00-Inf]	1.000	1.000
SCARNA16	101	0	0.00%	196	0	0.00%	1.00 [0e+00-Inf]	1.000	1.000
SCARNA20	101	0	0.00%	196	0	0.00%	1.00 [0e+00-Inf]	1.000	1.000
SCARNA3	101	0	0.00%	196	0	0.00%	1.00 [0e+00-Inf]	1.000	1.000
SCARNA4	101	0	0.00%	196	0	0.00%	1.00 [0e+00-Inf]	1.000	1.000
SCCPDH	101	0	0.00%	196	0	0.00%	1.00 [0e+00-Inf]	1.000	1.000
SCFD1	101	0	0.00%	196	0	0.00%	1.00 [0e+00-Inf]	1.000	1.000
SCML4	101	0	0.00%	196	0	0.00%	1.00 [0e+00-Inf]	1.000	1.000
SCN4A	101	0	0.00%	196	0	0.00%	1.00 [0e+00-Inf]	1.000	1.000
SCNM1	101	0	0.00%	196	0	0.00%	1.00 [0e+00-Inf]	1.000	1.000
SCNN1B	101	0	0.00%	196	0	0.00%	1.00 [0e+00-Inf]	1.000	1.000
SCNN1G	101	0	0.00%	196	0	0.00%	1.00 [0e+00-Inf]	1.000	1.000
SCPEP1	101	0	0.00%	196	0	0.00%	1.00 [0e+00-Inf]	1.000	1.000
SCRIB	101	0	0.00%	196	0	0.00%	1.00 [0e+00-Inf]	1.000	1.000
SCRN1	101	0	0.00%	196	0	0.00%	1.00 [0e+00-Inf]	1.000	1.000
SCRN2	101	0	0.00%	196	0	0.00%	1.00 [0e+00-Inf]	1.000	1.000
SCRT1	101	0	0.00%	196	0	0.00%	1.00 [0e+00-Inf]	1.000	1.000
SCYL3	101	0	0.00%	196	0	0.00%	1.00 [0e+00-Inf]	1.000	1.000
SDAD1	101	0	0.00%	196	0	0.00%	1.00 [0e+00-Inf]	1.000	1.000
SDC2	101	0	0.00%	196	0	0.00%	1.00 [0e+00-Inf]	1.000	1.000
SDC4	101	0	0.00%	196	0	0.00%	1.00 [0e+00-Inf]	1.000	1.000
SDCBP	101	0	0.00%	196	0	0.00%	1.00 [0e+00-Inf]	1.000	1.000
SDCCAG8	101	0	0.00%	196	0	0.00%	1.00 [0e+00-Inf]	1.000	1.000
SDF2	101	0	0.00%	196	0	0.00%	1.00 [0e+00-Inf]	1.000	1.000
SDHAF2	101	0	0.00%	196	0	0.00%	1.00 [0e+00-Inf]	1.000	1.000
SDHAP1	101	0	0.00%	196	0	0.00%	1.00 [0e+00-Inf]	1.000	1.000
SDHAP2	101	0	0.00%	196	0	0.00%	1.00 [0e+00-Inf]	1.000	1.000
SDHC	101	0	0.00%	196	0	0.00%	1.00 [0e+00-Inf]	1.000	1.000
SDK2	101	0	0.00%	196	0	0.00%	1.00 [0e+00-Inf]	1.000	1.000
SDR16C5	101	0	0.00%	196	0	0.00%	1.00 [0e+00-Inf]	1.000	1.000
SEBOX	101	0	0.00%	196	0	0.00%	1.00 [0e+00-Inf]	1.000	1.000
SEC11A	101	0	0.00%	196	0	0.00%	1.00 [0e+00-Inf]	1.000	1.000
SEC11C	101	0	0.00%	196	0	0.00%	1.00 [0e+00-Inf]	1.000	1.000
SEC14L1	101	0	0.00%	196	0	0.00%	1.00 [0e+00-Inf]	1.000	1.000
SEC14L5	101	0	0.00%	196	0	0.00%	1.00 [0e+00-Inf]	1.000	1.000
SEC16B	101	0	0.00%	196	0	0.00%	1.00 [0e+00-Inf]	1.000	1.000
SEC22B	101	0	0.00%	196	0	0.00%	1.00 [0e+00-Inf]	1.000	1.000
SEC24C	101	0	0.00%	196	0	0.00%	1.00 [0e+00-Inf]	1.000	1.000
SEC63	101	0	0.00%	196	0	0.00%	1.00 [0e+00-Inf]	1.000	1.000
SECTM1	101	0	0.00%	196	0	0.00%	1.00 [0e+00-Inf]	1.000	1.000
SELENBP1	101	0	0.00%	196	0	0.00%	1.00 [0e+00-Inf]	1.000	1.000
SELE	101	0	0.00%	196	0	0.00%	1.00 [0e+00-Inf]	1.000	1.000
SELL	101	0	0.00%	196	0	0.00%	1.00 [0e+00-Inf]	1.000	1.000
SELP	101	0	0.00%	196	0	0.00%	1.00 [0e+00-Inf]	1.000	1.000
SEMA4A	101	0	0.00%	196	0	0.00%	1.00 [0e+00-Inf]	1.000	1.000
SEMA4B	101	0	0.00%	196	0	0.00%	1.00 [0e+00-Inf]	1.000	1.000
SEMA6C	101	0	0.00%	196	0	0.00%	1.00 [0e+00-Inf]	1.000	1.000
SEMG1	101	0	0.00%	196	0	0.00%	1.00 [0e+00-Inf]	1.000	1.000

Symbol	CINSARC Low			CINSARC High			CINSARC High vs. Low		
	N tested	N altered	Fq alteration	N tested	N altered	Fq alteration	OR [95%CI]	p-value*	q-value
SEMG2	101	0	0.00%	196	0	0.00%	1.00 [0e+00-Inf]	1.000	1.000
SENP2	101	0	0.00%	196	0	0.00%	1.00 [0e+00-Inf]	1.000	1.000
SENP5	101	0	0.00%	196	0	0.00%	1.00 [0e+00-Inf]	1.000	1.000
SEPHS2	101	0	0.00%	196	0	0.00%	1.00 [0e+00-Inf]	1.000	1.000
sept-12	101	0	0.00%	196	0	0.00%	1.00 [0e+00-Inf]	1.000	1.000
sept-01	101	0	0.00%	196	0	0.00%	1.00 [0e+00-Inf]	1.000	1.000
sept-09	101	0	0.00%	196	0	0.00%	1.00 [0e+00-Inf]	1.000	1.000
MSRB1	101	0	0.00%	196	0	0.00%	1.00 [0e+00-Inf]	1.000	1.000
SERINC3	101	0	0.00%	196	0	0.00%	1.00 [0e+00-Inf]	1.000	1.000
SERPINB10	101	0	0.00%	196	0	0.00%	1.00 [0e+00-Inf]	1.000	1.000
SERPINB11	101	0	0.00%	196	0	0.00%	1.00 [0e+00-Inf]	1.000	1.000
SERPINB12	101	0	0.00%	196	0	0.00%	1.00 [0e+00-Inf]	1.000	1.000
SERPINB13	101	0	0.00%	196	0	0.00%	1.00 [0e+00-Inf]	1.000	1.000
SERPINB2	101	0	0.00%	196	0	0.00%	1.00 [0e+00-Inf]	1.000	1.000
SERPINB3	101	0	0.00%	196	0	0.00%	1.00 [0e+00-Inf]	1.000	1.000
SERPINB4	101	0	0.00%	196	0	0.00%	1.00 [0e+00-Inf]	1.000	1.000
SERPINB5	101	0	0.00%	196	0	0.00%	1.00 [0e+00-Inf]	1.000	1.000
SERPINB7	101	0	0.00%	196	0	0.00%	1.00 [0e+00-Inf]	1.000	1.000
SERPINB8	101	0	0.00%	196	0	0.00%	1.00 [0e+00-Inf]	1.000	1.000
SERPINC1	101	0	0.00%	196	0	0.00%	1.00 [0e+00-Inf]	1.000	1.000
SERPINH1	101	0	0.00%	196	0	0.00%	1.00 [0e+00-Inf]	1.000	1.000
SERTAD4	101	0	0.00%	196	0	0.00%	1.00 [0e+00-Inf]	1.000	1.000
SESN1	101	0	0.00%	196	1	0.51%	3.2e+07 [0e+00-Inf]	0.997	1.000
SETD1A	101	0	0.00%	196	0	0.00%	1.00 [0e+00-Inf]	1.000	1.000
SETDB1	101	0	0.00%	196	0	0.00%	1.00 [0e+00-Inf]	1.000	1.000
SEZ6L2	101	0	0.00%	196	0	0.00%	1.00 [0e+00-Inf]	1.000	1.000
SEZ6	101	0	0.00%	196	0	0.00%	1.00 [0e+00-Inf]	1.000	1.000
SF3B2	101	0	0.00%	196	0	0.00%	1.00 [0e+00-Inf]	1.000	1.000
SF3B4	101	0	0.00%	196	0	0.00%	1.00 [0e+00-Inf]	1.000	1.000
SFRP1	101	0	0.00%	196	0	0.00%	1.00 [0e+00-Inf]	1.000	1.000
SRSF1	101	0	0.00%	196	0	0.00%	1.00 [0e+00-Inf]	1.000	1.000
SRSF2	101	0	0.00%	196	0	0.00%	1.00 [0e+00-Inf]	1.000	1.000
SRSF6	101	0	0.00%	196	0	0.00%	1.00 [0e+00-Inf]	1.000	1.000
SFT2D2	101	0	0.00%	196	0	0.00%	1.00 [0e+00-Inf]	1.000	1.000
SFTA3	101	0	0.00%	196	0	0.00%	1.00 [0e+00-Inf]	1.000	1.000
SFTPA1	101	0	0.00%	196	0	0.00%	1.00 [0e+00-Inf]	1.000	1.000
SGCA	101	0	0.00%	196	0	0.00%	1.00 [0e+00-Inf]	1.000	1.000
POMK	101	0	0.00%	196	0	0.00%	1.00 [0e+00-Inf]	1.000	1.000
SGK2	101	0	0.00%	196	0	0.00%	1.00 [0e+00-Inf]	1.000	1.000
SGK3	101	0	0.00%	196	0	0.00%	1.00 [0e+00-Inf]	1.000	1.000
SGSH	101	0	0.00%	196	0	0.00%	1.00 [0e+00-Inf]	1.000	1.000
SH2B1	101	0	0.00%	196	0	0.00%	1.00 [0e+00-Inf]	1.000	1.000
SH2D1B	101	0	0.00%	196	0	0.00%	1.00 [0e+00-Inf]	1.000	1.000
SH2D2A	101	0	0.00%	196	0	0.00%	1.00 [0e+00-Inf]	1.000	1.000
SH2D7	101	0	0.00%	196	0	0.00%	1.00 [0e+00-Inf]	1.000	1.000
SH3BP5L	101	0	0.00%	196	0	0.00%	1.00 [0e+00-Inf]	1.000	1.000
SHANK2	101	0	0.00%	196	0	0.00%	1.00 [0e+00-Inf]	1.000	1.000
SHARPIN	101	0	0.00%	196	0	0.00%	1.00 [0e+00-Inf]	1.000	1.000
SHC1	101	0	0.00%	196	0	0.00%	1.00 [0e+00-Inf]	1.000	1.000
SHE	101	0	0.00%	196	0	0.00%	1.00 [0e+00-Inf]	1.000	1.000
SHISA4	101	0	0.00%	196	0	0.00%	1.00 [0e+00-Inf]	1.000	1.000
SHISA7	101	0	0.00%	196	0	0.00%	1.00 [0e+00-Inf]	1.000	1.000
SHISA9	101	0	0.00%	196	0	0.00%	1.00 [0e+00-Inf]	1.000	1.000
SIAH2	101	0	0.00%	196	0	0.00%	1.00 [0e+00-Inf]	1.000	1.000
SIGLEC14	101	4	3.96%	196	8	4.08%	1.03 [0.37-2.88]	0.960	1.000
SIGLEC9	101	0	0.00%	196	0	0.00%	1.00 [0e+00-Inf]	1.000	1.000
SIGMAR1	101	0	0.00%	196	0	0.00%	1.00 [0e+00-Inf]	1.000	1.000
SIPA1L2	101	0	0.00%	196	0	0.00%	1.00 [0e+00-Inf]	1.000	1.000
SIRPB1	101	0	0.00%	196	0	0.00%	1.00 [0e+00-Inf]	1.000	1.000
SIRT7	101	0	0.00%	196	0	0.00%	1.00 [0e+00-Inf]	1.000	1.000
SKA2	101	0	0.00%	196	0	0.00%	1.00 [0e+00-Inf]	1.000	1.000
SKAP1	101	0	0.00%	196	0	0.00%	1.00 [0e+00-Inf]	1.000	1.000
SKAP2	101	0	0.00%	196	0	0.00%	1.00 [0e+00-Inf]	1.000	1.000
SKIL	101	0	0.00%	196	0	0.00%	1.00 [0e+00-Inf]	1.000	1.000
SLA2	101	0	0.00%	196	0	0.00%	1.00 [0e+00-Inf]	1.000	1.000
SLAMF1	101	0	0.00%	196	0	0.00%	1.00 [0e+00-Inf]	1.000	1.000
SLAMF6	101	0	0.00%	196	0	0.00%	1.00 [0e+00-Inf]	1.000	1.000
SLAMF7	101	0	0.00%	196	0	0.00%	1.00 [0e+00-Inf]	1.000	1.000
SLAMF8	101	0	0.00%	196	0	0.00%	1.00 [0e+00-Inf]	1.000	1.000
SLAMF9	101	0	0.00%	196	0	0.00%	1.00 [0e+00-Inf]	1.000	1.000
SLA	101	0	0.00%	196	0	0.00%	1.00 [0e+00-Inf]	1.000	1.000

Symbol	CINSARC Low			CINSARC High			CINSARC High vs. Low		
	N tested	N altered	Fq alteration	N tested	N altered	Fq alteration	OR [95%CI]	p-value*	q-value
SLC10A5	101	0	0.00%	196	0	0.00%	1.00 [0e+00-Inf]	1.000	1.000
SLC12A5	101	0	0.00%	196	0	0.00%	1.00 [0e+00-Inf]	1.000	1.000
SLC13A2	101	0	0.00%	196	0	0.00%	1.00 [0e+00-Inf]	1.000	1.000
SLC13A3	101	0	0.00%	196	0	0.00%	1.00 [0e+00-Inf]	1.000	1.000
SLC16A3	101	0	0.00%	196	0	0.00%	1.00 [0e+00-Inf]	1.000	1.000
SLC16A5	101	0	0.00%	196	0	0.00%	1.00 [0e+00-Inf]	1.000	1.000
SLC16A6	101	0	0.00%	196	0	0.00%	1.00 [0e+00-Inf]	1.000	1.000
SLC17A6	101	0	0.00%	196	0	0.00%	1.00 [0e+00-Inf]	1.000	1.000
SLC17A9	101	0	0.00%	196	0	0.00%	1.00 [0e+00-Inf]	1.000	1.000
SLC19A2	101	0	0.00%	196	0	0.00%	1.00 [0e+00-Inf]	1.000	1.000
SLC1A2	101	0	0.00%	196	0	0.00%	1.00 [0e+00-Inf]	1.000	1.000
SLC20A2	101	0	0.00%	196	0	0.00%	1.00 [0e+00-Inf]	1.000	1.000
SLC25A10	101	0	0.00%	196	0	0.00%	1.00 [0e+00-Inf]	1.000	1.000
SLC25A19	101	0	0.00%	196	0	0.00%	1.00 [0e+00-Inf]	1.000	1.000
SLC25A21	101	0	0.00%	196	0	0.00%	1.00 [0e+00-Inf]	1.000	1.000
SLC25A32	101	0	0.00%	196	0	0.00%	1.00 [0e+00-Inf]	1.000	1.000
SLC25A44	101	0	0.00%	196	0	0.00%	1.00 [0e+00-Inf]	1.000	1.000
SLC26A11	101	0	0.00%	196	0	0.00%	1.00 [0e+00-Inf]	1.000	1.000
SLC26A7	101	0	0.00%	196	0	0.00%	1.00 [0e+00-Inf]	1.000	1.000
SLC26A9	101	0	0.00%	196	0	0.00%	1.00 [0e+00-Inf]	1.000	1.000
SLC27A3	101	0	0.00%	196	0	0.00%	1.00 [0e+00-Inf]	1.000	1.000
SLC28A1	101	0	0.00%	196	0	0.00%	1.00 [0e+00-Inf]	1.000	1.000
SLC29A2	101	0	0.00%	196	0	0.00%	1.00 [0e+00-Inf]	1.000	1.000
SLC2A10	101	0	0.00%	196	0	0.00%	1.00 [0e+00-Inf]	1.000	1.000
SLC2A4RG	101	0	0.00%	196	0	0.00%	1.00 [0e+00-Inf]	1.000	1.000
SLC30A10	101	0	0.00%	196	0	0.00%	1.00 [0e+00-Inf]	1.000	1.000
SLC30A1	101	0	0.00%	196	0	0.00%	1.00 [0e+00-Inf]	1.000	1.000
SLC30A8	101	0	0.00%	196	0	0.00%	1.00 [0e+00-Inf]	1.000	1.000
SLC32A1	101	0	0.00%	196	0	0.00%	1.00 [0e+00-Inf]	1.000	1.000
SLC35B1	101	0	0.00%	196	0	0.00%	1.00 [0e+00-Inf]	1.000	1.000
SLC35C2	101	0	0.00%	196	0	0.00%	1.00 [0e+00-Inf]	1.000	1.000
SLC35E3	101	0	0.00%	196	0	0.00%	1.00 [0e+00-Inf]	1.000	1.000
SLC35F3	101	0	0.00%	196	0	0.00%	1.00 [0e+00-Inf]	1.000	1.000
SLC37A3	101	0	0.00%	196	0	0.00%	1.00 [0e+00-Inf]	1.000	1.000
SLC38A10	101	0	0.00%	196	0	0.00%	1.00 [0e+00-Inf]	1.000	1.000
SLC39A11	101	0	0.00%	196	0	0.00%	1.00 [0e+00-Inf]	1.000	1.000
SLC39A1	101	0	0.00%	196	0	0.00%	1.00 [0e+00-Inf]	1.000	1.000
SLC39A4	101	1	0.99%	196	0	0.00%	1.6e-08 [0e+00-Inf]	0.996	1.000
SLC41A1	101	0	0.00%	196	0	0.00%	1.00 [0e+00-Inf]	1.000	1.000
SLC45A3	101	0	0.00%	196	0	0.00%	1.00 [0e+00-Inf]	1.000	1.000
SLC45A4	101	0	0.00%	196	0	0.00%	1.00 [0e+00-Inf]	1.000	1.000
SLC46A1	101	0	0.00%	196	0	0.00%	1.00 [0e+00-Inf]	1.000	1.000
SLC46A3	101	0	0.00%	196	0	0.00%	1.00 [0e+00-Inf]	1.000	1.000
SLC47A1	101	0	0.00%	196	0	0.00%	1.00 [0e+00-Inf]	1.000	1.000
SLC47A2	101	0	0.00%	196	0	0.00%	1.00 [0e+00-Inf]	1.000	1.000
SLC5A11	101	0	0.00%	196	0	0.00%	1.00 [0e+00-Inf]	1.000	1.000
SLC5A2	101	0	0.00%	196	0	0.00%	1.00 [0e+00-Inf]	1.000	1.000
SLC6A10P	101	0	0.00%	196	0	0.00%	1.00 [0e+00-Inf]	1.000	1.000
SLC6A4	101	0	0.00%	196	0	0.00%	1.00 [0e+00-Inf]	1.000	1.000
SLC7A13	101	0	0.00%	196	0	0.00%	1.00 [0e+00-Inf]	1.000	1.000
SLC7A5P1	101	0	0.00%	196	0	0.00%	1.00 [0e+00-Inf]	1.000	1.000
SLC7A5P2	101	1	0.99%	196	0	0.00%	1.6e-08 [0e+00-Inf]	0.996	1.000
SLC9C2	101	0	0.00%	196	0	0.00%	1.00 [0e+00-Inf]	1.000	1.000
SLC9A3R1	101	0	0.00%	196	0	0.00%	1.00 [0e+00-Inf]	1.000	1.000
SLC9A3R2	101	0	0.00%	196	0	0.00%	1.00 [0e+00-Inf]	1.000	1.000
SLC9A8	101	0	0.00%	196	0	0.00%	1.00 [0e+00-Inf]	1.000	1.000
SLCO2B1	101	0	0.00%	196	0	0.00%	1.00 [0e+00-Inf]	1.000	1.000
SLCO3A1	101	0	0.00%	196	0	0.00%	1.00 [0e+00-Inf]	1.000	1.000
SLCO4A1	101	0	0.00%	196	0	0.00%	1.00 [0e+00-Inf]	1.000	1.000
SLCO5A1	101	0	0.00%	196	0	0.00%	1.00 [0e+00-Inf]	1.000	1.000
SLFN11	101	0	0.00%	196	0	0.00%	1.00 [0e+00-Inf]	1.000	1.000
SLFN5	101	0	0.00%	196	0	0.00%	1.00 [0e+00-Inf]	1.000	1.000
SLPI	101	0	0.00%	196	0	0.00%	1.00 [0e+00-Inf]	1.000	1.000
SLURP1	101	0	0.00%	196	0	0.00%	1.00 [0e+00-Inf]	1.000	1.000
SMARCD2	101	0	0.00%	196	0	0.00%	1.00 [0e+00-Inf]	1.000	1.000
SMARCE1	101	0	0.00%	196	0	0.00%	1.00 [0e+00-Inf]	1.000	1.000
SMCP	101	0	0.00%	196	0	0.00%	1.00 [0e+00-Inf]	1.000	1.000
SMG1	101	0	0.00%	196	0	0.00%	1.00 [0e+00-Inf]	1.000	1.000
SMG5	101	0	0.00%	196	0	0.00%	1.00 [0e+00-Inf]	1.000	1.000
SMG7	101	0	0.00%	196	0	0.00%	1.00 [0e+00-Inf]	1.000	1.000
SMURF2	101	0	0.00%	196	0	0.00%	1.00 [0e+00-Inf]	1.000	1.000

Symbol	CINSARC Low			CINSARC High			CINSARC High vs. Low		
	N tested	N altered	Fq alteration	N tested	N altered	Fq alteration	OR [95%CI]	p-value*	q-value
SMYD2	101	0	0.00%	196	0	0.00%	1.00 [0e+00-Inf]	1.000	1.000
SMYD3	101	0	0.00%	196	0	0.00%	1.00 [0e+00-Inf]	1.000	1.000
SNAI1	101	0	0.00%	196	0	0.00%	1.00 [0e+00-Inf]	1.000	1.000
SNAI2	101	0	0.00%	196	0	0.00%	1.00 [0e+00-Inf]	1.000	1.000
SNAP47	101	0	0.00%	196	0	0.00%	1.00 [0e+00-Inf]	1.000	1.000
SNAPIN	101	0	0.00%	196	0	0.00%	1.00 [0e+00-Inf]	1.000	1.000
SNAR-I	101	0	0.00%	196	0	0.00%	1.00 [0e+00-Inf]	1.000	1.000
SNF8	101	0	0.00%	196	0	0.00%	1.00 [0e+00-Inf]	1.000	1.000
SNHG11	101	0	0.00%	196	0	0.00%	1.00 [0e+00-Inf]	1.000	1.000
SNHG6	101	0	0.00%	196	0	0.00%	1.00 [0e+00-Inf]	1.000	1.000
SNHG9	101	0	0.00%	196	0	0.00%	1.00 [0e+00-Inf]	1.000	1.000
SNN	101	0	0.00%	196	0	0.00%	1.00 [0e+00-Inf]	1.000	1.000
SNORA10	101	0	0.00%	196	0	0.00%	1.00 [0e+00-Inf]	1.000	1.000
SNORA14B	101	0	0.00%	196	0	0.00%	1.00 [0e+00-Inf]	1.000	1.000
SNORA16B	101	0	0.00%	196	0	0.00%	1.00 [0e+00-Inf]	1.000	1.000
SNORA21	101	0	0.00%	196	0	0.00%	1.00 [0e+00-Inf]	1.000	1.000
SNORA30	101	0	0.00%	196	0	0.00%	1.00 [0e+00-Inf]	1.000	1.000
SNORA36B	101	0	0.00%	196	0	0.00%	1.00 [0e+00-Inf]	1.000	1.000
SNORA37	101	0	0.00%	196	0	0.00%	1.00 [0e+00-Inf]	1.000	1.000
SNORA38B	101	0	0.00%	196	0	0.00%	1.00 [0e+00-Inf]	1.000	1.000
SNORA64	101	0	0.00%	196	0	0.00%	1.00 [0e+00-Inf]	1.000	1.000
SNORA71A	101	0	0.00%	196	0	0.00%	1.00 [0e+00-Inf]	1.000	1.000
SNORA71B	101	0	0.00%	196	0	0.00%	1.00 [0e+00-Inf]	1.000	1.000
SNORA71C	101	0	0.00%	196	0	0.00%	1.00 [0e+00-Inf]	1.000	1.000
SNORA71D	101	0	0.00%	196	0	0.00%	1.00 [0e+00-Inf]	1.000	1.000
SNORA72	101	0	0.00%	196	0	0.00%	1.00 [0e+00-Inf]	1.000	1.000
SNORA77	101	0	0.00%	196	0	0.00%	1.00 [0e+00-Inf]	1.000	1.000
SNORA78	101	0	0.00%	196	0	0.00%	1.00 [0e+00-Inf]	1.000	1.000
SNORD104	101	0	0.00%	196	0	0.00%	1.00 [0e+00-Inf]	1.000	1.000
SNORD124	101	0	0.00%	196	0	0.00%	1.00 [0e+00-Inf]	1.000	1.000
SNORD12B	101	0	0.00%	196	0	0.00%	1.00 [0e+00-Inf]	1.000	1.000
SNORD12C	101	0	0.00%	196	0	0.00%	1.00 [0e+00-Inf]	1.000	1.000
SNORD12	101	0	0.00%	196	0	0.00%	1.00 [0e+00-Inf]	1.000	1.000
SNORD15A	101	0	0.00%	196	0	0.00%	1.00 [0e+00-Inf]	1.000	1.000
SNORD15B	101	0	0.00%	196	0	0.00%	1.00 [0e+00-Inf]	1.000	1.000
SNORD1A	101	0	0.00%	196	0	0.00%	1.00 [0e+00-Inf]	1.000	1.000
SNORD1B	101	0	0.00%	196	0	0.00%	1.00 [0e+00-Inf]	1.000	1.000
SNORD1C	101	0	0.00%	196	0	0.00%	1.00 [0e+00-Inf]	1.000	1.000
SNORD42A	101	0	0.00%	196	0	0.00%	1.00 [0e+00-Inf]	1.000	1.000
SNORD42B	101	0	0.00%	196	0	0.00%	1.00 [0e+00-Inf]	1.000	1.000
SNORD44	101	0	0.00%	196	0	0.00%	1.00 [0e+00-Inf]	1.000	1.000
SNORD47	101	0	0.00%	196	0	0.00%	1.00 [0e+00-Inf]	1.000	1.000
SNORD4A	101	0	0.00%	196	0	0.00%	1.00 [0e+00-Inf]	1.000	1.000
SNORD4B	101	0	0.00%	196	0	0.00%	1.00 [0e+00-Inf]	1.000	1.000
SNORD54	101	0	0.00%	196	0	0.00%	1.00 [0e+00-Inf]	1.000	1.000
SNORD60	101	0	0.00%	196	0	0.00%	1.00 [0e+00-Inf]	1.000	1.000
SNORD66	101	0	0.00%	196	0	0.00%	1.00 [0e+00-Inf]	1.000	1.000
SNORD74	101	0	0.00%	196	0	0.00%	1.00 [0e+00-Inf]	1.000	1.000
SNORD75	101	0	0.00%	196	0	0.00%	1.00 [0e+00-Inf]	1.000	1.000
SNORD76	101	0	0.00%	196	0	0.00%	1.00 [0e+00-Inf]	1.000	1.000
SNORD77	101	0	0.00%	196	0	0.00%	1.00 [0e+00-Inf]	1.000	1.000
SNORD78	101	0	0.00%	196	0	0.00%	1.00 [0e+00-Inf]	1.000	1.000
SNORD79	101	0	0.00%	196	0	0.00%	1.00 [0e+00-Inf]	1.000	1.000
SNORD80	101	0	0.00%	196	0	0.00%	1.00 [0e+00-Inf]	1.000	1.000
SNORD81	101	0	0.00%	196	0	0.00%	1.00 [0e+00-Inf]	1.000	1.000
SNORD87	101	0	0.00%	196	0	0.00%	1.00 [0e+00-Inf]	1.000	1.000
SNORD93	101	0	0.00%	196	0	0.00%	1.00 [0e+00-Inf]	1.000	1.000
SNRNP25	101	0	0.00%	196	0	0.00%	1.00 [0e+00-Inf]	1.000	1.000
SNRPA1	101	0	0.00%	196	0	0.00%	1.00 [0e+00-Inf]	1.000	1.000
SNRPE	101	0	0.00%	196	0	0.00%	1.00 [0e+00-Inf]	1.000	1.000
SNTB1	101	0	0.00%	196	0	0.00%	1.00 [0e+00-Inf]	1.000	1.000
SNTG1	101	0	0.00%	196	0	0.00%	1.00 [0e+00-Inf]	1.000	1.000
SNX10	101	0	0.00%	196	0	0.00%	1.00 [0e+00-Inf]	1.000	1.000
SNX11	101	0	0.00%	196	0	0.00%	1.00 [0e+00-Inf]	1.000	1.000
SNX16	101	0	0.00%	196	0	0.00%	1.00 [0e+00-Inf]	1.000	1.000
SNX21	101	0	0.00%	196	0	0.00%	1.00 [0e+00-Inf]	1.000	1.000
SNX27	101	0	0.00%	196	0	0.00%	1.00 [0e+00-Inf]	1.000	1.000
SNX31	101	0	0.00%	196	0	0.00%	1.00 [0e+00-Inf]	1.000	1.000
SNX3	101	0	0.00%	196	0	0.00%	1.00 [0e+00-Inf]	1.000	1.000
SNX6	101	0	0.00%	196	0	0.00%	1.00 [0e+00-Inf]	1.000	1.000
SOAT1	101	0	0.00%	196	0	0.00%	1.00 [0e+00-Inf]	1.000	1.000

Symbol	CINSARC Low			CINSARC High			CINSARC High vs. Low		
	N tested	N altered	Fq alteration	N tested	N altered	Fq alteration	OR [95%CI]	p-value*	q-value
SOBP	101	0	0.00%	196	0	0.00%	1.00 [0e+00-Inf]	1.000	1.000
SOCS1	101	0	0.00%	196	0	0.00%	1.00 [0e+00-Inf]	1.000	1.000
SOCS3	101	0	0.00%	196	0	0.00%	1.00 [0e+00-Inf]	1.000	1.000
SOCS7	101	0	0.00%	196	0	0.00%	1.00 [0e+00-Inf]	1.000	1.000
CAPN15	101	0	0.00%	196	0	0.00%	1.00 [0e+00-Inf]	1.000	1.000
SOX13	101	0	0.00%	196	0	0.00%	1.00 [0e+00-Inf]	1.000	1.000
SOX17	101	0	0.00%	196	0	0.00%	1.00 [0e+00-Inf]	1.000	1.000
SOX18	101	0	0.00%	196	0	0.00%	1.00 [0e+00-Inf]	1.000	1.000
SOX2-OT	101	0	0.00%	196	0	0.00%	1.00 [0e+00-Inf]	1.000	1.000
SOX2	101	0	0.00%	196	0	0.00%	1.00 [0e+00-Inf]	1.000	1.000
SOX6	101	0	0.00%	196	0	0.00%	1.00 [0e+00-Inf]	1.000	1.000
SOX8	101	0	0.00%	196	0	0.00%	1.00 [0e+00-Inf]	1.000	1.000
SOX9	101	0	0.00%	196	0	0.00%	1.00 [0e+00-Inf]	1.000	1.000
SP2	101	0	0.00%	196	0	0.00%	1.00 [0e+00-Inf]	1.000	1.000
SP4	101	0	0.00%	196	0	0.00%	1.00 [0e+00-Inf]	1.000	1.000
SP6	101	0	0.00%	196	0	0.00%	1.00 [0e+00-Inf]	1.000	1.000
SP8	101	0	0.00%	196	0	0.00%	1.00 [0e+00-Inf]	1.000	1.000
SPACA3	101	0	0.00%	196	0	0.00%	1.00 [0e+00-Inf]	1.000	1.000
SPAG1	101	0	0.00%	196	0	0.00%	1.00 [0e+00-Inf]	1.000	1.000
SPAG4	101	0	0.00%	196	0	0.00%	1.00 [0e+00-Inf]	1.000	1.000
SPAG5	101	0	0.00%	196	0	0.00%	1.00 [0e+00-Inf]	1.000	1.000
SPAG9	101	0	0.00%	196	0	0.00%	1.00 [0e+00-Inf]	1.000	1.000
SPATA17	101	0	0.00%	196	0	0.00%	1.00 [0e+00-Inf]	1.000	1.000
SPATA20	101	0	0.00%	196	0	0.00%	1.00 [0e+00-Inf]	1.000	1.000
SPATA2	101	0	0.00%	196	0	0.00%	1.00 [0e+00-Inf]	1.000	1.000
SPATA8	101	0	0.00%	196	0	0.00%	1.00 [0e+00-Inf]	1.000	1.000
SPATC1	101	0	0.00%	196	0	0.00%	1.00 [0e+00-Inf]	1.000	1.000
SPCS2	101	0	0.00%	196	0	0.00%	1.00 [0e+00-Inf]	1.000	1.000
SPHAR	101	0	0.00%	196	0	0.00%	1.00 [0e+00-Inf]	1.000	1.000
SPHK1	101	0	0.00%	196	0	0.00%	1.00 [0e+00-Inf]	1.000	1.000
EPPIN	101	0	0.00%	196	0	0.00%	1.00 [0e+00-Inf]	1.000	1.000
SPINT3	101	0	0.00%	196	0	0.00%	1.00 [0e+00-Inf]	1.000	1.000
SPINT4	101	0	0.00%	196	0	0.00%	1.00 [0e+00-Inf]	1.000	1.000
SPNS1	101	0	0.00%	196	0	0.00%	1.00 [0e+00-Inf]	1.000	1.000
SPN	101	0	0.00%	196	0	0.00%	1.00 [0e+00-Inf]	1.000	1.000
SPO11	101	0	0.00%	196	0	0.00%	1.00 [0e+00-Inf]	1.000	1.000
SPOP	101	0	0.00%	196	0	0.00%	1.00 [0e+00-Inf]	1.000	1.000
SPRR1A	101	0	0.00%	196	0	0.00%	1.00 [0e+00-Inf]	1.000	1.000
SPRR1B	101	0	0.00%	196	0	0.00%	1.00 [0e+00-Inf]	1.000	1.000
SPRR2A	101	0	0.00%	196	0	0.00%	1.00 [0e+00-Inf]	1.000	1.000
SPRR2B	101	0	0.00%	196	0	0.00%	1.00 [0e+00-Inf]	1.000	1.000
SPRR2C	101	0	0.00%	196	0	0.00%	1.00 [0e+00-Inf]	1.000	1.000
SPRR2D	101	0	0.00%	196	0	0.00%	1.00 [0e+00-Inf]	1.000	1.000
SPRR2E	101	0	0.00%	196	0	0.00%	1.00 [0e+00-Inf]	1.000	1.000
SPRR2F	101	0	0.00%	196	0	0.00%	1.00 [0e+00-Inf]	1.000	1.000
SPRR2G	101	0	0.00%	196	0	0.00%	1.00 [0e+00-Inf]	1.000	1.000
SPRR3	101	0	0.00%	196	0	0.00%	1.00 [0e+00-Inf]	1.000	1.000
SPRR4	101	0	0.00%	196	0	0.00%	1.00 [0e+00-Inf]	1.000	1.000
SPSB3	101	0	0.00%	196	0	0.00%	1.00 [0e+00-Inf]	1.000	1.000
SPTA1	101	0	0.00%	196	0	0.00%	1.00 [0e+00-Inf]	1.000	1.000
SPTBN2	101	0	0.00%	196	0	0.00%	1.00 [0e+00-Inf]	1.000	1.000
SQLE	101	0	0.00%	196	0	0.00%	1.00 [0e+00-Inf]	1.000	1.000
SRCAP	101	0	0.00%	196	0	0.00%	1.00 [0e+00-Inf]	1.000	1.000
SRCIN1	101	0	0.00%	196	0	0.00%	1.00 [0e+00-Inf]	1.000	1.000
SRC	101	0	0.00%	196	0	0.00%	1.00 [0e+00-Inf]	1.000	1.000
SRGAP1	101	0	0.00%	196	0	0.00%	1.00 [0e+00-Inf]	1.000	1.000
SRGAP2	101	0	0.00%	196	0	0.00%	1.00 [0e+00-Inf]	1.000	1.000
SRL	101	0	0.00%	196	0	0.00%	1.00 [0e+00-Inf]	1.000	1.000
SRMS	101	0	0.00%	196	0	0.00%	1.00 [0e+00-Inf]	1.000	1.000
SRP54	101	0	0.00%	196	0	0.00%	1.00 [0e+00-Inf]	1.000	1.000
SRP68	101	0	0.00%	196	0	0.00%	1.00 [0e+00-Inf]	1.000	1.000
SRP9	101	0	0.00%	196	0	0.00%	1.00 [0e+00-Inf]	1.000	1.000
SRRM2	101	0	0.00%	196	0	0.00%	1.00 [0e+00-Inf]	1.000	1.000
SRY	101	62	61.39%	196	108	55.10%	0.77 [0.51-1.16]	0.300	1.000
SS18L1	101	0	0.00%	196	0	0.00%	1.00 [0e+00-Inf]	1.000	1.000
SSC5D	101	0	0.00%	196	0	0.00%	1.00 [0e+00-Inf]	1.000	1.000
SSH2	101	0	0.00%	196	0	0.00%	1.00 [0e+00-Inf]	1.000	1.000
SSH3	101	0	0.00%	196	0	0.00%	1.00 [0e+00-Inf]	1.000	1.000
SSR2	101	0	0.00%	196	0	0.00%	1.00 [0e+00-Inf]	1.000	1.000
SSTR1	101	0	0.00%	196	0	0.00%	1.00 [0e+00-Inf]	1.000	1.000
SSTR2	101	0	0.00%	196	0	0.00%	1.00 [0e+00-Inf]	1.000	1.000

Symbol	CINSARC Low			CINSARC High			CINSARC High vs. Low		
	N tested	N altered	Fq alteration	N tested	N altered	Fq alteration	OR [95%CI]	p-value*	q-value
SSTR5	101	0	0.00%	196	0	0.00%	1.00 [0e+00-Inf]	1.000	1.000
ST18	101	0	0.00%	196	0	0.00%	1.00 [0e+00-Inf]	1.000	1.000
ST3GAL1	101	0	0.00%	196	0	0.00%	1.00 [0e+00-Inf]	1.000	1.000
ST6GALNAC1	101	0	0.00%	196	0	0.00%	1.00 [0e+00-Inf]	1.000	1.000
ST6GALNAC2	101	0	0.00%	196	0	0.00%	1.00 [0e+00-Inf]	1.000	1.000
ST8SIA2	101	0	0.00%	196	0	0.00%	1.00 [0e+00-Inf]	1.000	1.000
STAC2	101	0	0.00%	196	0	0.00%	1.00 [0e+00-Inf]	1.000	1.000
STARD10	101	0	0.00%	196	0	0.00%	1.00 [0e+00-Inf]	1.000	1.000
STARD3	101	0	0.00%	196	0	0.00%	1.00 [0e+00-Inf]	1.000	1.000
STARD6	101	0	0.00%	196	0	0.00%	1.00 [0e+00-Inf]	1.000	1.000
STAR	101	0	0.00%	196	0	0.00%	1.00 [0e+00-Inf]	1.000	1.000
STAU1	101	0	0.00%	196	0	0.00%	1.00 [0e+00-Inf]	1.000	1.000
STAU2	101	0	0.00%	196	0	0.00%	1.00 [0e+00-Inf]	1.000	1.000
STBD1	101	0	0.00%	196	0	0.00%	1.00 [0e+00-Inf]	1.000	1.000
STC2	101	0	0.00%	196	0	0.00%	1.00 [0e+00-Inf]	1.000	1.000
STK31	101	0	0.00%	196	0	0.00%	1.00 [0e+00-Inf]	1.000	1.000
STK3	101	0	0.00%	196	0	0.00%	1.00 [0e+00-Inf]	1.000	1.000
STK4	101	0	0.00%	196	0	0.00%	1.00 [0e+00-Inf]	1.000	1.000
STMN2	101	0	0.00%	196	0	0.00%	1.00 [0e+00-Inf]	1.000	1.000
STMN3	101	0	0.00%	196	0	0.00%	1.00 [0e+00-Inf]	1.000	1.000
STRADA	101	0	0.00%	196	0	0.00%	1.00 [0e+00-Inf]	1.000	1.000
STRN3	101	0	0.00%	196	0	0.00%	1.00 [0e+00-Inf]	1.000	1.000
STUB1	101	0	0.00%	196	0	0.00%	1.00 [0e+00-Inf]	1.000	1.000
STX16	101	0	0.00%	196	0	0.00%	1.00 [0e+00-Inf]	1.000	1.000
STX1B	101	0	0.00%	196	0	0.00%	1.00 [0e+00-Inf]	1.000	1.000
STX4	101	0	0.00%	196	0	0.00%	1.00 [0e+00-Inf]	1.000	1.000
STX6	101	0	0.00%	196	0	0.00%	1.00 [0e+00-Inf]	1.000	1.000
STXBP4	101	0	0.00%	196	0	0.00%	1.00 [0e+00-Inf]	1.000	1.000
SULF1	101	0	0.00%	196	0	0.00%	1.00 [0e+00-Inf]	1.000	1.000
SULF2	101	0	0.00%	196	0	0.00%	1.00 [0e+00-Inf]	1.000	1.000
SULT1A1	101	0	0.00%	196	0	0.00%	1.00 [0e+00-Inf]	1.000	1.000
SULT1A2	101	0	0.00%	196	0	0.00%	1.00 [0e+00-Inf]	1.000	1.000
SULT1A3	101	0	0.00%	196	0	0.00%	1.00 [0e+00-Inf]	1.000	1.000
SUMO1P1	101	0	0.00%	196	0	0.00%	1.00 [0e+00-Inf]	1.000	1.000
SUMO1P3	101	0	0.00%	196	0	0.00%	1.00 [0e+00-Inf]	1.000	1.000
SUMO2	101	0	0.00%	196	0	0.00%	1.00 [0e+00-Inf]	1.000	1.000
SUPT4H1	101	0	0.00%	196	0	0.00%	1.00 [0e+00-Inf]	1.000	1.000
SUPT6H	101	0	0.00%	196	0	0.00%	1.00 [0e+00-Inf]	1.000	1.000
SUSD4	101	0	0.00%	196	0	0.00%	1.00 [0e+00-Inf]	1.000	1.000
SV2A	101	0	0.00%	196	0	0.00%	1.00 [0e+00-Inf]	1.000	1.000
SV2B	101	0	0.00%	196	0	0.00%	1.00 [0e+00-Inf]	1.000	1.000
SVIP	101	0	0.00%	196	0	0.00%	1.00 [0e+00-Inf]	1.000	1.000
SYBU	101	0	0.00%	196	0	0.00%	1.00 [0e+00-Inf]	1.000	1.000
SYCP2	101	0	0.00%	196	0	0.00%	1.00 [0e+00-Inf]	1.000	1.000
SYNGR2	101	0	0.00%	196	0	0.00%	1.00 [0e+00-Inf]	1.000	1.000
SYNGR3	101	0	0.00%	196	0	0.00%	1.00 [0e+00-Inf]	1.000	1.000
SYNM	101	0	0.00%	196	0	0.00%	1.00 [0e+00-Inf]	1.000	1.000
SYNPO2L	101	0	0.00%	196	0	0.00%	1.00 [0e+00-Inf]	1.000	1.000
SYNRG	101	0	0.00%	196	0	0.00%	1.00 [0e+00-Inf]	1.000	1.000
SYS1-DBNDD2	101	0	0.00%	196	0	0.00%	1.00 [0e+00-Inf]	1.000	1.000
SYS1	101	0	0.00%	196	0	0.00%	1.00 [0e+00-Inf]	1.000	1.000
SYT11	101	0	0.00%	196	0	0.00%	1.00 [0e+00-Inf]	1.000	1.000
SYT12	101	0	0.00%	196	0	0.00%	1.00 [0e+00-Inf]	1.000	1.000
SYT14	101	0	0.00%	196	0	0.00%	1.00 [0e+00-Inf]	1.000	1.000
SYT17	101	0	0.00%	196	0	0.00%	1.00 [0e+00-Inf]	1.000	1.000
SYT2	101	0	0.00%	196	0	0.00%	1.00 [0e+00-Inf]	1.000	1.000
SYT7	101	0	0.00%	196	0	0.00%	1.00 [0e+00-Inf]	1.000	1.000
SYTL2	101	0	0.00%	196	0	0.00%	1.00 [0e+00-Inf]	1.000	1.000
TAC4	101	0	0.00%	196	0	0.00%	1.00 [0e+00-Inf]	1.000	1.000
TACC1	101	0	0.00%	196	0	0.00%	1.00 [0e+00-Inf]	1.000	1.000
TACO1	101	0	0.00%	196	0	0.00%	1.00 [0e+00-Inf]	1.000	1.000
TADA1	101	0	0.00%	196	0	0.00%	1.00 [0e+00-Inf]	1.000	1.000
TADA2A	101	0	0.00%	196	0	0.00%	1.00 [0e+00-Inf]	1.000	1.000
TAF15	101	0	0.00%	196	0	0.00%	1.00 [0e+00-Inf]	1.000	1.000
TAF1A	101	0	0.00%	196	0	0.00%	1.00 [0e+00-Inf]	1.000	1.000
TAF2	101	0	0.00%	196	0	0.00%	1.00 [0e+00-Inf]	1.000	1.000
TAF4	101	0	0.00%	196	0	0.00%	1.00 [0e+00-Inf]	1.000	1.000
TAF5L	101	0	0.00%	196	0	0.00%	1.00 [0e+00-Inf]	1.000	1.000
TAGLN2	101	0	0.00%	196	0	0.00%	1.00 [0e+00-Inf]	1.000	1.000
TANC2	101	0	0.00%	196	0	0.00%	1.00 [0e+00-Inf]	1.000	1.000
TAOK1	101	0	0.00%	196	0	0.00%	1.00 [0e+00-Inf]	1.000	1.000

Symbol	CINSARC Low			CINSARC High			CINSARC High vs. Low		
	N tested	N altered	Fq alteration	N tested	N altered	Fq alteration	OR [95%CI]	p-value*	q-value
TAOK2	101	0	0.00%	196	0	0.00%	1.00 [0e+00-Inf]	1.000	1.000
TARBP1	101	0	0.00%	196	0	0.00%	1.00 [0e+00-Inf]	1.000	1.000
TARP	101	0	0.00%	196	0	0.00%	1.00 [0e+00-Inf]	1.000	1.000
TARS2	101	0	0.00%	196	0	0.00%	1.00 [0e+00-Inf]	1.000	1.000
TAS2R39	101	0	0.00%	196	0	0.00%	1.00 [0e+00-Inf]	1.000	1.000
TAS2R40	101	0	0.00%	196	0	0.00%	1.00 [0e+00-Inf]	1.000	1.000
TAS2R43	101	0	0.00%	196	0	0.00%	1.00 [0e+00-Inf]	1.000	1.000
TATDN1	101	0	0.00%	196	0	0.00%	1.00 [0e+00-Inf]	1.000	1.000
TATDN3	101	0	0.00%	196	0	0.00%	1.00 [0e+00-Inf]	1.000	1.000
TAX1BP1	101	0	0.00%	196	0	0.00%	1.00 [0e+00-Inf]	1.000	1.000
TBC1D10B	101	0	0.00%	196	0	0.00%	1.00 [0e+00-Inf]	1.000	1.000
TBC1D10C	101	0	0.00%	196	0	0.00%	1.00 [0e+00-Inf]	1.000	1.000
TBC1D15	101	0	0.00%	196	0	0.00%	1.00 [0e+00-Inf]	1.000	1.000
TBC1D16	101	0	0.00%	196	0	0.00%	1.00 [0e+00-Inf]	1.000	1.000
TBC1D24	101	0	0.00%	196	0	0.00%	1.00 [0e+00-Inf]	1.000	1.000
TBC1D3B	101	0	0.00%	196	0	0.00%	1.00 [0e+00-Inf]	1.000	1.000
TBC1D3C	101	0	0.00%	196	0	0.00%	1.00 [0e+00-Inf]	1.000	1.000
TBC1D3G	101	0	0.00%	196	0	0.00%	1.00 [0e+00-Inf]	1.000	1.000
TBC1D3H	101	0	0.00%	196	0	0.00%	1.00 [0e+00-Inf]	1.000	1.000
TBC1D3P2	101	0	0.00%	196	0	0.00%	1.00 [0e+00-Inf]	1.000	1.000
TBC1D3	101	0	0.00%	196	0	0.00%	1.00 [0e+00-Inf]	1.000	1.000
TBC1D7	101	0	0.00%	196	0	0.00%	1.00 [0e+00-Inf]	1.000	1.000
TBCD	101	0	0.00%	196	0	0.00%	1.00 [0e+00-Inf]	1.000	1.000
TBCE	101	0	0.00%	196	0	0.00%	1.00 [0e+00-Inf]	1.000	1.000
TBK1	101	0	0.00%	196	0	0.00%	1.00 [0e+00-Inf]	1.000	1.000
TBKBP1	101	0	0.00%	196	0	0.00%	1.00 [0e+00-Inf]	1.000	1.000
TBL1XR1	101	0	0.00%	196	0	0.00%	1.00 [0e+00-Inf]	1.000	1.000
TBL1Y	101	64	63.37%	196	115	58.67%	0.82 [0.54-1.24]	0.434	1.000
TBL3	101	0	0.00%	196	0	0.00%	1.00 [0e+00-Inf]	1.000	1.000
TBX10	101	0	0.00%	196	0	0.00%	1.00 [0e+00-Inf]	1.000	1.000
TBX19	101	0	0.00%	196	0	0.00%	1.00 [0e+00-Inf]	1.000	1.000
TBX21	101	0	0.00%	196	0	0.00%	1.00 [0e+00-Inf]	1.000	1.000
TBX2	101	0	0.00%	196	0	0.00%	1.00 [0e+00-Inf]	1.000	1.000
TBX4	101	0	0.00%	196	0	0.00%	1.00 [0e+00-Inf]	1.000	1.000
TBX6	101	0	0.00%	196	0	0.00%	1.00 [0e+00-Inf]	1.000	1.000
TCAM1P	101	0	0.00%	196	0	0.00%	1.00 [0e+00-Inf]	1.000	1.000
TCAP	101	0	0.00%	196	0	0.00%	1.00 [0e+00-Inf]	1.000	1.000
TCEA1	101	0	0.00%	196	0	0.00%	1.00 [0e+00-Inf]	1.000	1.000
TCEA2	101	0	0.00%	196	0	0.00%	1.00 [0e+00-Inf]	1.000	1.000
TCFL5	101	0	0.00%	196	0	0.00%	1.00 [0e+00-Inf]	1.000	1.000
TCHHL1	101	0	0.00%	196	0	0.00%	1.00 [0e+00-Inf]	1.000	1.000
TCHH	101	0	0.00%	196	0	0.00%	1.00 [0e+00-Inf]	1.000	1.000
TCIRG1	101	0	0.00%	196	0	0.00%	1.00 [0e+00-Inf]	1.000	1.000
TCTEX1D2	101	0	0.00%	196	0	0.00%	1.00 [0e+00-Inf]	1.000	1.000
TDRD10	101	0	0.00%	196	0	0.00%	1.00 [0e+00-Inf]	1.000	1.000
TDRD5	101	0	0.00%	196	0	0.00%	1.00 [0e+00-Inf]	1.000	1.000
TDRKH	101	0	0.00%	196	0	0.00%	1.00 [0e+00-Inf]	1.000	1.000
TEDDM1	101	0	0.00%	196	0	0.00%	1.00 [0e+00-Inf]	1.000	1.000
TEKT5	101	0	0.00%	196	0	0.00%	1.00 [0e+00-Inf]	1.000	1.000
TELO2	101	0	0.00%	196	0	0.00%	1.00 [0e+00-Inf]	1.000	1.000
TERF1	101	0	0.00%	196	0	0.00%	1.00 [0e+00-Inf]	1.000	1.000
TEX14	101	0	0.00%	196	0	0.00%	1.00 [0e+00-Inf]	1.000	1.000
TEX15	101	0	0.00%	196	0	0.00%	1.00 [0e+00-Inf]	1.000	1.000
TEX19	101	0	0.00%	196	0	0.00%	1.00 [0e+00-Inf]	1.000	1.000
TEX2	101	0	0.00%	196	0	0.00%	1.00 [0e+00-Inf]	1.000	1.000
TFAP2C	101	0	0.00%	196	0	0.00%	1.00 [0e+00-Inf]	1.000	1.000
TFAP4	101	0	0.00%	196	0	0.00%	1.00 [0e+00-Inf]	1.000	1.000
TFB2M	101	0	0.00%	196	0	0.00%	1.00 [0e+00-Inf]	1.000	1.000
TFRC	101	0	0.00%	196	0	0.00%	1.00 [0e+00-Inf]	1.000	1.000
TGFB1I1	101	0	0.00%	196	0	0.00%	1.00 [0e+00-Inf]	1.000	1.000
TGFB2	101	0	0.00%	196	0	0.00%	1.00 [0e+00-Inf]	1.000	1.000
TGIF2LY	101	58	57.43%	196	102	52.04%	0.80 [0.54-1.21]	0.378	1.000
TGIF2	101	0	0.00%	196	0	0.00%	1.00 [0e+00-Inf]	1.000	1.000
TGM2	101	0	0.00%	196	0	0.00%	1.00 [0e+00-Inf]	1.000	1.000
TGS1	101	0	0.00%	196	0	0.00%	1.00 [0e+00-Inf]	1.000	1.000
TG	101	0	0.00%	196	0	0.00%	1.00 [0e+00-Inf]	1.000	1.000
NELFCD	101	0	0.00%	196	0	0.00%	1.00 [0e+00-Inf]	1.000	1.000
THAP1	101	0	0.00%	196	0	0.00%	1.00 [0e+00-Inf]	1.000	1.000
THAP2	101	0	0.00%	196	0	0.00%	1.00 [0e+00-Inf]	1.000	1.000
THAP6	101	0	0.00%	196	0	0.00%	1.00 [0e+00-Inf]	1.000	1.000
THBS3	101	0	0.00%	196	0	0.00%	1.00 [0e+00-Inf]	1.000	1.000

Symbol	CINSARC Low			CINSARC High			CINSARC High vs. Low		
	N tested	N altered	Fq alteration	N tested	N altered	Fq alteration	OR [95%CI]	p-value*	q-value
THEM4	101	0	0.00%	196	0	0.00%	1.00 [0e+00-Inf]	1.000	1.000
THEM5	101	0	0.00%	196	0	0.00%	1.00 [0e+00-Inf]	1.000	1.000
THOC3	101	0	0.00%	196	0	0.00%	1.00 [0e+00-Inf]	1.000	1.000
ALYREF	101	0	0.00%	196	0	0.00%	1.00 [0e+00-Inf]	1.000	1.000
THOC6	101	0	0.00%	196	0	0.00%	1.00 [0e+00-Inf]	1.000	1.000
THPO	101	0	0.00%	196	0	0.00%	1.00 [0e+00-Inf]	1.000	1.000
THRA	101	0	0.00%	196	0	0.00%	1.00 [0e+00-Inf]	1.000	1.000
THRSP	101	0	0.00%	196	0	0.00%	1.00 [0e+00-Inf]	1.000	1.000
THUMPD1	101	0	0.00%	196	0	0.00%	1.00 [0e+00-Inf]	1.000	1.000
TIAF1	101	0	0.00%	196	0	0.00%	1.00 [0e+00-Inf]	1.000	1.000
TIGD5	101	0	0.00%	196	0	0.00%	1.00 [0e+00-Inf]	1.000	1.000
TIGD7	101	0	0.00%	196	0	0.00%	1.00 [0e+00-Inf]	1.000	1.000
PAM16	101	0	0.00%	196	0	0.00%	1.00 [0e+00-Inf]	1.000	1.000
TIMM17A	101	0	0.00%	196	0	0.00%	1.00 [0e+00-Inf]	1.000	1.000
TIMP2	101	0	0.00%	196	0	0.00%	1.00 [0e+00-Inf]	1.000	1.000
TIPRL	101	0	0.00%	196	0	0.00%	1.00 [0e+00-Inf]	1.000	1.000
TK1	101	0	0.00%	196	0	0.00%	1.00 [0e+00-Inf]	1.000	1.000
TLCD1	101	0	0.00%	196	0	0.00%	1.00 [0e+00-Inf]	1.000	1.000
TLK2	101	0	0.00%	196	0	0.00%	1.00 [0e+00-Inf]	1.000	1.000
TLR5	101	0	0.00%	196	0	0.00%	1.00 [0e+00-Inf]	1.000	1.000
TM2D2	101	0	0.00%	196	0	0.00%	1.00 [0e+00-Inf]	1.000	1.000
TM4SF19	101	0	0.00%	196	0	0.00%	1.00 [0e+00-Inf]	1.000	1.000
DCSTAMP	101	0	0.00%	196	0	0.00%	1.00 [0e+00-Inf]	1.000	1.000
TMBIM4	101	0	0.00%	196	0	0.00%	1.00 [0e+00-Inf]	1.000	1.000
TMC3	101	0	0.00%	196	0	0.00%	1.00 [0e+00-Inf]	1.000	1.000
TMC5	101	0	0.00%	196	0	0.00%	1.00 [0e+00-Inf]	1.000	1.000
TMC6	101	0	0.00%	196	0	0.00%	1.00 [0e+00-Inf]	1.000	1.000
TMC7	101	0	0.00%	196	0	0.00%	1.00 [0e+00-Inf]	1.000	1.000
TMC8	101	0	0.00%	196	0	0.00%	1.00 [0e+00-Inf]	1.000	1.000
TMCC2	101	0	0.00%	196	0	0.00%	1.00 [0e+00-Inf]	1.000	1.000
TMCO1	101	0	0.00%	196	0	0.00%	1.00 [0e+00-Inf]	1.000	1.000
TMED10P1	101	0	0.00%	196	0	0.00%	1.00 [0e+00-Inf]	1.000	1.000
TMEM100	101	0	0.00%	196	0	0.00%	1.00 [0e+00-Inf]	1.000	1.000
TMEM104	101	0	0.00%	196	0	0.00%	1.00 [0e+00-Inf]	1.000	1.000
TMEM105	101	0	0.00%	196	0	0.00%	1.00 [0e+00-Inf]	1.000	1.000
TMEM114	101	0	0.00%	196	0	0.00%	1.00 [0e+00-Inf]	1.000	1.000
TMEM126A	101	0	0.00%	196	0	0.00%	1.00 [0e+00-Inf]	1.000	1.000
TMEM126B	101	0	0.00%	196	0	0.00%	1.00 [0e+00-Inf]	1.000	1.000
TMEM134	101	0	0.00%	196	0	0.00%	1.00 [0e+00-Inf]	1.000	1.000
TMEM135	101	0	0.00%	196	0	0.00%	1.00 [0e+00-Inf]	1.000	1.000
TMEM138	101	0	0.00%	196	0	0.00%	1.00 [0e+00-Inf]	1.000	1.000
TMEM150B	101	0	0.00%	196	0	0.00%	1.00 [0e+00-Inf]	1.000	1.000
TMEM151A	101	0	0.00%	196	0	0.00%	1.00 [0e+00-Inf]	1.000	1.000
TMEM159	101	0	0.00%	196	0	0.00%	1.00 [0e+00-Inf]	1.000	1.000
TMEM183A	101	0	0.00%	196	0	0.00%	1.00 [0e+00-Inf]	1.000	1.000
TMEM186	101	0	0.00%	196	0	0.00%	1.00 [0e+00-Inf]	1.000	1.000
TMEM189-UB	101	0	0.00%	196	0	0.00%	1.00 [0e+00-Inf]	1.000	1.000
TMEM189	101	0	0.00%	196	0	0.00%	1.00 [0e+00-Inf]	1.000	1.000
TMEM190	101	0	0.00%	196	0	0.00%	1.00 [0e+00-Inf]	1.000	1.000
TMEM199	101	0	0.00%	196	0	0.00%	1.00 [0e+00-Inf]	1.000	1.000
TMEM19	101	0	0.00%	196	0	0.00%	1.00 [0e+00-Inf]	1.000	1.000
TMEM202	101	0	0.00%	196	0	0.00%	1.00 [0e+00-Inf]	1.000	1.000
TMEM204	101	0	0.00%	196	0	0.00%	1.00 [0e+00-Inf]	1.000	1.000
TMEM206	101	0	0.00%	196	0	0.00%	1.00 [0e+00-Inf]	1.000	1.000
TMEM207	101	0	0.00%	196	0	0.00%	1.00 [0e+00-Inf]	1.000	1.000
TMEM216	101	0	0.00%	196	0	0.00%	1.00 [0e+00-Inf]	1.000	1.000
TMEM219	101	0	0.00%	196	0	0.00%	1.00 [0e+00-Inf]	1.000	1.000
TMEM41A	101	0	0.00%	196	0	0.00%	1.00 [0e+00-Inf]	1.000	1.000
TMEM44	101	0	0.00%	196	0	0.00%	1.00 [0e+00-Inf]	1.000	1.000
VMP1	101	0	0.00%	196	0	0.00%	1.00 [0e+00-Inf]	1.000	1.000
TMEM63A	101	0	0.00%	196	0	0.00%	1.00 [0e+00-Inf]	1.000	1.000
TMEM64	101	0	0.00%	196	0	0.00%	1.00 [0e+00-Inf]	1.000	1.000
TMEM65	101	0	0.00%	196	0	0.00%	1.00 [0e+00-Inf]	1.000	1.000
TMEM67	101	0	0.00%	196	0	0.00%	1.00 [0e+00-Inf]	1.000	1.000
TMEM68	101	0	0.00%	196	0	0.00%	1.00 [0e+00-Inf]	1.000	1.000
TMEM70	101	0	0.00%	196	0	0.00%	1.00 [0e+00-Inf]	1.000	1.000
TMEM71	101	0	0.00%	196	0	0.00%	1.00 [0e+00-Inf]	1.000	1.000
TMEM74	101	0	0.00%	196	0	0.00%	1.00 [0e+00-Inf]	1.000	1.000
TMEM79	101	0	0.00%	196	0	0.00%	1.00 [0e+00-Inf]	1.000	1.000
TMEM81	101	0	0.00%	196	0	0.00%	1.00 [0e+00-Inf]	1.000	1.000
TMEM86A	101	0	0.00%	196	0	0.00%	1.00 [0e+00-Inf]	1.000	1.000

Symbol	CINSARC Low			CINSARC High			CINSARC High vs. Low		
	N tested	N altered	Fq alteration	N tested	N altered	Fq alteration	OR [95%CI]	p-value*	q-value
TMEM8A	101	0	0.00%	196	0	0.00%	1.00 [0e+00-Inf]	1.000	1.000
TMEM92	101	0	0.00%	196	0	0.00%	1.00 [0e+00-Inf]	1.000	1.000
TMEM97	101	0	0.00%	196	0	0.00%	1.00 [0e+00-Inf]	1.000	1.000
TMEM98	101	0	0.00%	196	0	0.00%	1.00 [0e+00-Inf]	1.000	1.000
TMEM99	101	0	0.00%	196	0	0.00%	1.00 [0e+00-Inf]	1.000	1.000
TMEM9	101	0	0.00%	196	0	0.00%	1.00 [0e+00-Inf]	1.000	1.000
TMIGD1	101	0	0.00%	196	0	0.00%	1.00 [0e+00-Inf]	1.000	1.000
TMOD4	101	0	0.00%	196	0	0.00%	1.00 [0e+00-Inf]	1.000	1.000
TMSB4Y	101	64	63.37%	196	116	59.18%	0.84 [0.55-1.27]	0.485	1.000
TMTC3	101	0	0.00%	196	0	0.00%	1.00 [0e+00-Inf]	1.000	1.000
TNFAIP1	101	0	0.00%	196	0	0.00%	1.00 [0e+00-Inf]	1.000	1.000
TNFAIP8L2	101	0	0.00%	196	0	0.00%	1.00 [0e+00-Inf]	1.000	1.000
TNFRSF11A	101	0	0.00%	196	0	0.00%	1.00 [0e+00-Inf]	1.000	1.000
TNFRSF11B	101	0	0.00%	196	0	0.00%	1.00 [0e+00-Inf]	1.000	1.000
TNFRSF12A	101	0	0.00%	196	0	0.00%	1.00 [0e+00-Inf]	1.000	1.000
TNFRSF17	101	0	0.00%	196	0	0.00%	1.00 [0e+00-Inf]	1.000	1.000
TNFRSF6B	101	0	0.00%	196	0	0.00%	1.00 [0e+00-Inf]	1.000	1.000
TNFSF18	101	0	0.00%	196	0	0.00%	1.00 [0e+00-Inf]	1.000	1.000
TNFSF4	101	0	0.00%	196	0	0.00%	1.00 [0e+00-Inf]	1.000	1.000
TNK2	101	0	0.00%	196	0	0.00%	1.00 [0e+00-Inf]	1.000	1.000
TNNC2	101	0	0.00%	196	0	0.00%	1.00 [0e+00-Inf]	1.000	1.000
TNNI1	101	0	0.00%	196	0	0.00%	1.00 [0e+00-Inf]	1.000	1.000
TNNT2	101	0	0.00%	196	0	0.00%	1.00 [0e+00-Inf]	1.000	1.000
TNN	101	0	0.00%	196	0	0.00%	1.00 [0e+00-Inf]	1.000	1.000
TNP2	101	0	0.00%	196	0	0.00%	1.00 [0e+00-Inf]	1.000	1.000
TNRC6A	101	0	0.00%	196	0	0.00%	1.00 [0e+00-Inf]	1.000	1.000
TNRC6C	101	0	0.00%	196	0	0.00%	1.00 [0e+00-Inf]	1.000	1.000
TNR	101	0	0.00%	196	0	0.00%	1.00 [0e+00-Inf]	1.000	1.000
TNS4	101	0	0.00%	196	0	0.00%	1.00 [0e+00-Inf]	1.000	1.000
TOB1	101	0	0.00%	196	0	0.00%	1.00 [0e+00-Inf]	1.000	1.000
TOM1L1	101	0	0.00%	196	0	0.00%	1.00 [0e+00-Inf]	1.000	1.000
TOMM20	101	0	0.00%	196	0	0.00%	1.00 [0e+00-Inf]	1.000	1.000
TOMM34	101	0	0.00%	196	0	0.00%	1.00 [0e+00-Inf]	1.000	1.000
TOMM40L	101	0	0.00%	196	0	0.00%	1.00 [0e+00-Inf]	1.000	1.000
TOMM7	101	0	0.00%	196	0	0.00%	1.00 [0e+00-Inf]	1.000	1.000
TOP1MT	101	0	0.00%	196	0	0.00%	1.00 [0e+00-Inf]	1.000	1.000
TOP1P1	101	0	0.00%	196	0	0.00%	1.00 [0e+00-Inf]	1.000	1.000
TOP1	101	0	0.00%	196	0	0.00%	1.00 [0e+00-Inf]	1.000	1.000
TOP2A	101	0	0.00%	196	0	0.00%	1.00 [0e+00-Inf]	1.000	1.000
TOR1AIP1	101	0	0.00%	196	0	0.00%	1.00 [0e+00-Inf]	1.000	1.000
TOR1AIP2	101	0	0.00%	196	0	0.00%	1.00 [0e+00-Inf]	1.000	1.000
TOR3A	101	0	0.00%	196	0	0.00%	1.00 [0e+00-Inf]	1.000	1.000
TOX2	101	0	0.00%	196	0	0.00%	1.00 [0e+00-Inf]	1.000	1.000
TOX	101	0	0.00%	196	0	0.00%	1.00 [0e+00-Inf]	1.000	1.000
TP53BP2	101	0	0.00%	196	0	0.00%	1.00 [0e+00-Inf]	1.000	1.000
TP53I13	101	0	0.00%	196	0	0.00%	1.00 [0e+00-Inf]	1.000	1.000
TP53INP1	101	0	0.00%	196	0	0.00%	1.00 [0e+00-Inf]	1.000	1.000
TP53INP2	101	0	0.00%	196	0	0.00%	1.00 [0e+00-Inf]	1.000	1.000
TP53RK	101	0	0.00%	196	0	0.00%	1.00 [0e+00-Inf]	1.000	1.000
TP53TG3B	101	0	0.00%	196	0	0.00%	1.00 [0e+00-Inf]	1.000	1.000
TP53TG5	101	0	0.00%	196	0	0.00%	1.00 [0e+00-Inf]	1.000	1.000
TP63	101	0	0.00%	196	0	0.00%	1.00 [0e+00-Inf]	1.000	1.000
TPCN2	101	0	0.00%	196	0	0.00%	1.00 [0e+00-Inf]	1.000	1.000
TPD52L2	101	0	0.00%	196	0	0.00%	1.00 [0e+00-Inf]	1.000	1.000
TPD52	101	0	0.00%	196	0	0.00%	1.00 [0e+00-Inf]	1.000	1.000
TPH1	101	0	0.00%	196	0	0.00%	1.00 [0e+00-Inf]	1.000	1.000
TPH2	101	0	0.00%	196	0	0.00%	1.00 [0e+00-Inf]	1.000	1.000
TPM3	101	0	0.00%	196	0	0.00%	1.00 [0e+00-Inf]	1.000	1.000
TPRG1	101	0	0.00%	196	0	0.00%	1.00 [0e+00-Inf]	1.000	1.000
TPR	101	0	0.00%	196	0	0.00%	1.00 [0e+00-Inf]	1.000	1.000
TPSAB1	101	0	0.00%	196	0	0.00%	1.00 [0e+00-Inf]	1.000	1.000
TPSB2	101	0	0.00%	196	0	0.00%	1.00 [0e+00-Inf]	1.000	1.000
TPSD1	101	0	0.00%	196	0	0.00%	1.00 [0e+00-Inf]	1.000	1.000
TPSG1	101	0	0.00%	196	0	0.00%	1.00 [0e+00-Inf]	1.000	1.000
TRA2A	101	0	0.00%	196	0	0.00%	1.00 [0e+00-Inf]	1.000	1.000
TRAF3IP3	101	0	0.00%	196	0	0.00%	1.00 [0e+00-Inf]	1.000	1.000
TRAF4	101	0	0.00%	196	0	0.00%	1.00 [0e+00-Inf]	1.000	1.000
TRAF5	101	0	0.00%	196	0	0.00%	1.00 [0e+00-Inf]	1.000	1.000
TRAF7	101	0	0.00%	196	0	0.00%	1.00 [0e+00-Inf]	1.000	1.000
TRAM1	101	0	0.00%	196	0	0.00%	1.00 [0e+00-Inf]	1.000	1.000
TRAP1	101	0	0.00%	196	0	0.00%	1.00 [0e+00-Inf]	1.000	1.000

Symbol	CINSARC Low			CINSARC High			CINSARC High vs. Low		
	N tested	N altered	Fq alteration	N tested	N altered	Fq alteration	OR [95%CI]	p-value*	q-value
TRAPPC9	101	0	0.00%	196	0	0.00%	1.00 [0e+00-Inf]	1.000	1.000
TRHDE	101	0	0.00%	196	0	0.00%	1.00 [0e+00-Inf]	1.000	1.000
TRHR	101	0	0.00%	196	0	0.00%	1.00 [0e+00-Inf]	1.000	1.000
TRIB1	101	0	0.00%	196	0	0.00%	1.00 [0e+00-Inf]	1.000	1.000
TRIL	101	0	0.00%	196	0	0.00%	1.00 [0e+00-Inf]	1.000	1.000
TRIM11	101	0	0.00%	196	0	0.00%	1.00 [0e+00-Inf]	1.000	1.000
TRIM17	101	0	0.00%	196	0	0.00%	1.00 [0e+00-Inf]	1.000	1.000
TRIM25	101	0	0.00%	196	0	0.00%	1.00 [0e+00-Inf]	1.000	1.000
TRIM37	101	0	0.00%	196	0	0.00%	1.00 [0e+00-Inf]	1.000	1.000
TRIM46	101	0	0.00%	196	0	0.00%	1.00 [0e+00-Inf]	1.000	1.000
TRIM47	101	0	0.00%	196	0	0.00%	1.00 [0e+00-Inf]	1.000	1.000
TRIM55	101	0	0.00%	196	0	0.00%	1.00 [0e+00-Inf]	1.000	1.000
TRIM58	101	0	0.00%	196	0	0.00%	1.00 [0e+00-Inf]	1.000	1.000
TRIM65	101	0	0.00%	196	0	0.00%	1.00 [0e+00-Inf]	1.000	1.000
TRIM67	101	0	0.00%	196	0	0.00%	1.00 [0e+00-Inf]	1.000	1.000
TRIM72	101	0	0.00%	196	0	0.00%	1.00 [0e+00-Inf]	1.000	1.000
TRMT12	101	0	0.00%	196	0	0.00%	1.00 [0e+00-Inf]	1.000	1.000
TRPA1	101	0	0.00%	196	0	0.00%	1.00 [0e+00-Inf]	1.000	1.000
TRPC4AP	101	0	0.00%	196	0	0.00%	1.00 [0e+00-Inf]	1.000	1.000
TRPS1	101	0	0.00%	196	0	0.00%	1.00 [0e+00-Inf]	1.000	1.000
TRPV5	101	0	0.00%	196	0	0.00%	1.00 [0e+00-Inf]	1.000	1.000
TRPV6	101	0	0.00%	196	0	0.00%	1.00 [0e+00-Inf]	1.000	1.000
PRSS3P2	101	9	8.91%	196	6	3.06%	0.32 [0.13-0.79]	3.70E-02	1.000
TSC2	101	0	0.00%	196	0	0.00%	1.00 [0e+00-Inf]	1.000	1.000
TSEN15	101	0	0.00%	196	0	0.00%	1.00 [0e+00-Inf]	1.000	1.000
TSEN54	101	0	0.00%	196	0	0.00%	1.00 [0e+00-Inf]	1.000	1.000
TSFM	101	0	0.00%	196	0	0.00%	1.00 [0e+00-Inf]	1.000	1.000
TSG101	101	0	0.00%	196	0	0.00%	1.00 [0e+00-Inf]	1.000	1.000
TSHZ2	101	0	0.00%	196	0	0.00%	1.00 [0e+00-Inf]	1.000	1.000
TSKU	101	0	0.00%	196	0	0.00%	1.00 [0e+00-Inf]	1.000	1.000
TSNARE1	101	0	0.00%	196	0	0.00%	1.00 [0e+00-Inf]	1.000	1.000
TSNAX-DISC1	101	0	0.00%	196	0	0.00%	1.00 [0e+00-Inf]	1.000	1.000
TSNAX	101	0	0.00%	196	0	0.00%	1.00 [0e+00-Inf]	1.000	1.000
TSPAN10	101	0	0.00%	196	0	0.00%	1.00 [0e+00-Inf]	1.000	1.000
TSPAN31	101	0	0.00%	196	0	0.00%	1.00 [0e+00-Inf]	1.000	1.000
TSPAN8	101	0	0.00%	196	0	0.00%	1.00 [0e+00-Inf]	1.000	1.000
TSPY1	101	64	63.37%	196	116	59.18%	0.84 [0.55-1.27]	0.485	1.000
TSPY2	101	63	62.38%	196	111	56.63%	0.79 [0.52-1.19]	0.342	1.000
TSPY3	101	64	63.37%	196	116	59.18%	0.84 [0.55-1.27]	0.485	1.000
TSPY4	101	64	63.37%	196	116	59.18%	0.84 [0.55-1.27]	0.485	1.000
TSPYL5	101	0	0.00%	196	0	0.00%	1.00 [0e+00-Inf]	1.000	1.000
TSTA3	101	0	0.00%	196	0	0.00%	1.00 [0e+00-Inf]	1.000	1.000
TSTD1	101	0	0.00%	196	0	0.00%	1.00 [0e+00-Inf]	1.000	1.000
TTC13	101	0	0.00%	196	0	0.00%	1.00 [0e+00-Inf]	1.000	1.000
TTC14	101	0	0.00%	196	0	0.00%	1.00 [0e+00-Inf]	1.000	1.000
TTC23	101	0	0.00%	196	0	0.00%	1.00 [0e+00-Inf]	1.000	1.000
TTC24	101	0	0.00%	196	0	0.00%	1.00 [0e+00-Inf]	1.000	1.000
EMC2	101	0	0.00%	196	0	0.00%	1.00 [0e+00-Inf]	1.000	1.000
TTLL6	101	0	0.00%	196	0	0.00%	1.00 [0e+00-Inf]	1.000	1.000
TTPAL	101	0	0.00%	196	0	0.00%	1.00 [0e+00-Inf]	1.000	1.000
TTPA	101	0	0.00%	196	0	0.00%	1.00 [0e+00-Inf]	1.000	1.000
TTTY10	101	64	63.37%	196	116	59.18%	0.84 [0.55-1.27]	0.485	1.000
TTTY11	101	64	63.37%	196	116	59.18%	0.84 [0.55-1.27]	0.485	1.000
TTTY12	101	64	63.37%	196	116	59.18%	0.84 [0.55-1.27]	0.485	1.000
TTTY13	101	65	64.36%	196	117	59.69%	0.82 [0.54-1.25]	0.435	1.000
TTTY15	101	64	63.37%	196	115	58.67%	0.82 [0.54-1.24]	0.434	1.000
TTTY16	101	64	63.37%	196	116	59.18%	0.84 [0.55-1.27]	0.485	1.000
TTTY17A	101	64	63.37%	196	112	57.14%	0.77 [0.51-1.17]	0.302	1.000
TTTY17B	101	64	63.37%	196	112	57.14%	0.77 [0.51-1.17]	0.302	1.000
TTTY18	101	64	63.37%	196	116	59.18%	0.84 [0.55-1.27]	0.485	1.000
TTTY19	101	64	63.37%	196	116	59.18%	0.84 [0.55-1.27]	0.485	1.000
TTTY1B	101	64	63.37%	196	116	59.18%	0.84 [0.55-1.27]	0.485	1.000
TTTY20	101	64	63.37%	196	116	59.18%	0.84 [0.55-1.27]	0.485	1.000
TTTY21	101	64	63.37%	196	116	59.18%	0.84 [0.55-1.27]	0.485	1.000
TTTY22	101	64	63.37%	196	116	59.18%	0.84 [0.55-1.27]	0.485	1.000
TTTY23	101	63	62.38%	196	114	58.16%	0.84 [0.55-1.27]	0.484	1.000
TTTY2	101	64	63.37%	196	116	59.18%	0.84 [0.55-1.27]	0.485	1.000
TTTY3B	101	66	65.35%	196	117	59.69%	0.79 [0.52-1.19]	0.343	1.000
TTTY4C	101	65	64.36%	196	117	59.69%	0.82 [0.54-1.25]	0.435	1.000
TTTY5	101	65	64.36%	196	117	59.69%	0.82 [0.54-1.25]	0.435	1.000
TTTY6B	101	65	64.36%	196	117	59.69%	0.82 [0.54-1.25]	0.435	1.000

Symbol	CINSARC Low			CINSARC High			CINSARC High vs. Low		
	N tested	N altered	Fq alteration	N tested	N altered	Fq alteration	OR [95%CI]	p-value*	q-value
TTTY6	101	65	64.36%	196	117	59.69%	0.82 [0.54-1.25]	0.435	1.000
TTTY7	101	64	63.37%	196	116	59.18%	0.84 [0.55-1.27]	0.485	1.000
TTTY8	101	64	63.37%	196	116	59.18%	0.84 [0.55-1.27]	0.485	1.000
TTTY9B	101	65	64.36%	196	117	59.69%	0.82 [0.54-1.25]	0.435	1.000
TTYH2	101	0	0.00%	196	0	0.00%	1.00 [0e+00-Inf]	1.000	1.000
TUBB1	101	0	0.00%	196	0	0.00%	1.00 [0e+00-Inf]	1.000	1.000
TUBD1	101	0	0.00%	196	0	0.00%	1.00 [0e+00-Inf]	1.000	1.000
TUFM	101	0	0.00%	196	0	0.00%	1.00 [0e+00-Inf]	1.000	1.000
TUFT1	101	0	0.00%	196	0	0.00%	1.00 [0e+00-Inf]	1.000	1.000
TXNDC11	101	0	0.00%	196	0	0.00%	1.00 [0e+00-Inf]	1.000	1.000
TXNIP	101	0	0.00%	196	0	0.00%	1.00 [0e+00-Inf]	1.000	1.000
U2AF2	101	0	0.00%	196	0	0.00%	1.00 [0e+00-Inf]	1.000	1.000
UAP1	101	0	0.00%	196	0	0.00%	1.00 [0e+00-Inf]	1.000	1.000
UBAP2L	101	0	0.00%	196	0	0.00%	1.00 [0e+00-Inf]	1.000	1.000
UBE2C	101	0	0.00%	196	0	0.00%	1.00 [0e+00-Inf]	1.000	1.000
UBE2I	101	0	0.00%	196	0	0.00%	1.00 [0e+00-Inf]	1.000	1.000
UBE2MP1	101	1	0.99%	196	0	0.00%	1.6e-08 [0e+00-Inf]	0.996	1.000
UBE2N	101	0	0.00%	196	0	0.00%	1.00 [0e+00-Inf]	1.000	1.000
UBE2O	101	0	0.00%	196	0	0.00%	1.00 [0e+00-Inf]	1.000	1.000
UBE2Q1	101	0	0.00%	196	0	0.00%	1.00 [0e+00-Inf]	1.000	1.000
UBE2Q2P1	101	0	0.00%	196	0	0.00%	1.00 [0e+00-Inf]	1.000	1.000
UBE2S	101	0	0.00%	196	0	0.00%	1.00 [0e+00-Inf]	1.000	1.000
UBE2T	101	0	0.00%	196	0	0.00%	1.00 [0e+00-Inf]	1.000	1.000
UBE2V1	101	0	0.00%	196	0	0.00%	1.00 [0e+00-Inf]	1.000	1.000
UBE2V2	101	0	0.00%	196	0	0.00%	1.00 [0e+00-Inf]	1.000	1.000
UBE2W	101	0	0.00%	196	0	0.00%	1.00 [0e+00-Inf]	1.000	1.000
UBE2Z	101	0	0.00%	196	0	0.00%	1.00 [0e+00-Inf]	1.000	1.000
UBFD1	101	0	0.00%	196	0	0.00%	1.00 [0e+00-Inf]	1.000	1.000
UBL3	101	0	0.00%	196	0	0.00%	1.00 [0e+00-Inf]	1.000	1.000
UBN1	101	0	0.00%	196	0	0.00%	1.00 [0e+00-Inf]	1.000	1.000
UBQLN4	101	0	0.00%	196	0	0.00%	1.00 [0e+00-Inf]	1.000	1.000
UBR5	101	0	0.00%	196	0	0.00%	1.00 [0e+00-Inf]	1.000	1.000
UBTFL1	101	0	0.00%	196	0	0.00%	1.00 [0e+00-Inf]	1.000	1.000
UBXN2B	101	0	0.00%	196	0	0.00%	1.00 [0e+00-Inf]	1.000	1.000
UBXN7	101	0	0.00%	196	0	0.00%	1.00 [0e+00-Inf]	1.000	1.000
UBXN8	101	0	0.00%	196	0	0.00%	1.00 [0e+00-Inf]	1.000	1.000
UCHL5	101	0	0.00%	196	0	0.00%	1.00 [0e+00-Inf]	1.000	1.000
UCK2	101	0	0.00%	196	0	0.00%	1.00 [0e+00-Inf]	1.000	1.000
UCKL1-AS1	101	0	0.00%	196	0	0.00%	1.00 [0e+00-Inf]	1.000	1.000
UCKL1	101	0	0.00%	196	0	0.00%	1.00 [0e+00-Inf]	1.000	1.000
UCP2	101	0	0.00%	196	0	0.00%	1.00 [0e+00-Inf]	1.000	1.000
UCP3	101	0	0.00%	196	0	0.00%	1.00 [0e+00-Inf]	1.000	1.000
UFC1	101	0	0.00%	196	0	0.00%	1.00 [0e+00-Inf]	1.000	1.000
UHMK1	101	0	0.00%	196	0	0.00%	1.00 [0e+00-Inf]	1.000	1.000
ULK2	101	0	0.00%	196	0	0.00%	1.00 [0e+00-Inf]	1.000	1.000
UMOD	101	0	0.00%	196	0	0.00%	1.00 [0e+00-Inf]	1.000	1.000
UNC119	101	0	0.00%	196	0	0.00%	1.00 [0e+00-Inf]	1.000	1.000
UNC13D	101	0	0.00%	196	0	0.00%	1.00 [0e+00-Inf]	1.000	1.000
UNC45A	101	0	0.00%	196	0	0.00%	1.00 [0e+00-Inf]	1.000	1.000
UNC45B	101	0	0.00%	196	0	0.00%	1.00 [0e+00-Inf]	1.000	1.000
UNC5D	101	0	0.00%	196	0	0.00%	1.00 [0e+00-Inf]	1.000	1.000
UNC93B1	101	0	0.00%	196	0	0.00%	1.00 [0e+00-Inf]	1.000	1.000
UNKL	101	0	0.00%	196	0	0.00%	1.00 [0e+00-Inf]	1.000	1.000
UNK	101	0	0.00%	196	0	0.00%	1.00 [0e+00-Inf]	1.000	1.000
UQCC1	101	0	0.00%	196	0	0.00%	1.00 [0e+00-Inf]	1.000	1.000
UQCRB	101	0	0.00%	196	0	0.00%	1.00 [0e+00-Inf]	1.000	1.000
UQCRC2	101	0	0.00%	196	0	0.00%	1.00 [0e+00-Inf]	1.000	1.000
URB2	101	0	0.00%	196	0	0.00%	1.00 [0e+00-Inf]	1.000	1.000
USF1	101	0	0.00%	196	0	0.00%	1.00 [0e+00-Inf]	1.000	1.000
USH1C	101	0	0.00%	196	0	0.00%	1.00 [0e+00-Inf]	1.000	1.000
USH1G	101	0	0.00%	196	0	0.00%	1.00 [0e+00-Inf]	1.000	1.000
USH2A	101	0	0.00%	196	1	0.51%	3.2e+07 [0e+00-Inf]	0.997	1.000
USO1	101	0	0.00%	196	0	0.00%	1.00 [0e+00-Inf]	1.000	1.000
USP13	101	0	0.00%	196	0	0.00%	1.00 [0e+00-Inf]	1.000	1.000
USP17L2	101	1	0.99%	196	1	0.51%	0.51 [0.05-5.30]	0.638	1.000
USP21	101	0	0.00%	196	0	0.00%	1.00 [0e+00-Inf]	1.000	1.000
USP31	101	0	0.00%	196	0	0.00%	1.00 [0e+00-Inf]	1.000	1.000
USP32	101	0	0.00%	196	0	0.00%	1.00 [0e+00-Inf]	1.000	1.000
USP35	101	0	0.00%	196	0	0.00%	1.00 [0e+00-Inf]	1.000	1.000
USP36	101	0	0.00%	196	0	0.00%	1.00 [0e+00-Inf]	1.000	1.000
USP54	101	0	0.00%	196	0	0.00%	1.00 [0e+00-Inf]	1.000	1.000

Symbol	CINSARC Low			CINSARC High			CINSARC High vs. Low		
	N tested	N altered	Fq alteration	N tested	N altered	Fq alteration	OR [95%CI]	p-value*	q-value
USP7	101	0	0.00%	196	0	0.00%	1.00 [0e+00-Inf]	1.000	1.000
USP9Y	101	64	63.37%	196	115	58.67%	0.82 [0.54-1.24]	0.434	1.000
USPL1	101	0	0.00%	196	0	0.00%	1.00 [0e+00-Inf]	1.000	1.000
UTP18	101	0	0.00%	196	0	0.00%	1.00 [0e+00-Inf]	1.000	1.000
UTP23	101	0	0.00%	196	0	0.00%	1.00 [0e+00-Inf]	1.000	1.000
UTS2B	101	0	0.00%	196	0	0.00%	1.00 [0e+00-Inf]	1.000	1.000
UTS2R	101	0	0.00%	196	0	0.00%	1.00 [0e+00-Inf]	1.000	1.000
UTY	101	64	63.37%	196	115	58.67%	0.82 [0.54-1.24]	0.434	1.000
UVRAG	101	0	0.00%	196	0	0.00%	1.00 [0e+00-Inf]	1.000	1.000
VAMP4	101	0	0.00%	196	0	0.00%	1.00 [0e+00-Inf]	1.000	1.000
VANGL2	101	0	0.00%	196	0	0.00%	1.00 [0e+00-Inf]	1.000	1.000
VAPB	101	0	0.00%	196	0	0.00%	1.00 [0e+00-Inf]	1.000	1.000
VASH2	101	0	0.00%	196	0	0.00%	1.00 [0e+00-Inf]	1.000	1.000
VASN	101	0	0.00%	196	0	0.00%	1.00 [0e+00-Inf]	1.000	1.000
VCL	101	0	0.00%	196	0	0.00%	1.00 [0e+00-Inf]	1.000	1.000
VCPIP1	101	0	0.00%	196	0	0.00%	1.00 [0e+00-Inf]	1.000	1.000
VCP	101	0	0.00%	196	0	0.00%	1.00 [0e+00-Inf]	1.000	1.000
VCY	101	64	63.37%	196	116	59.18%	0.84 [0.55-1.27]	0.485	1.000
VDAC2	101	0	0.00%	196	0	0.00%	1.00 [0e+00-Inf]	1.000	1.000
VDAC3	101	0	0.00%	196	0	0.00%	1.00 [0e+00-Inf]	1.000	1.000
VEZF1	101	0	0.00%	196	0	0.00%	1.00 [0e+00-Inf]	1.000	1.000
VHLL	101	0	0.00%	196	0	0.00%	1.00 [0e+00-Inf]	1.000	1.000
VKORC1	101	0	0.00%	196	0	0.00%	1.00 [0e+00-Inf]	1.000	1.000
VN1R5	101	0	0.00%	196	0	0.00%	1.00 [0e+00-Inf]	1.000	1.000
VPS13B	101	0	0.00%	196	0	0.00%	1.00 [0e+00-Inf]	1.000	1.000
VPS28	101	1	0.99%	196	0	0.00%	1.6e-08 [0e+00-Inf]	0.996	1.000
VPS37C	101	0	0.00%	196	0	0.00%	1.00 [0e+00-Inf]	1.000	1.000
VPS41	101	0	0.00%	196	0	0.00%	1.00 [0e+00-Inf]	1.000	1.000
VPS45	101	0	0.00%	196	0	0.00%	1.00 [0e+00-Inf]	1.000	1.000
VPS4B	101	0	0.00%	196	0	0.00%	1.00 [0e+00-Inf]	1.000	1.000
VPS72	101	0	0.00%	196	0	0.00%	1.00 [0e+00-Inf]	1.000	1.000
VPS8	101	0	0.00%	196	0	0.00%	1.00 [0e+00-Inf]	1.000	1.000
VSIG8	101	0	0.00%	196	0	0.00%	1.00 [0e+00-Inf]	1.000	1.000
VSTM2L	101	0	0.00%	196	0	0.00%	1.00 [0e+00-Inf]	1.000	1.000
VTN	101	0	0.00%	196	0	0.00%	1.00 [0e+00-Inf]	1.000	1.000
VWA3A	101	0	0.00%	196	0	0.00%	1.00 [0e+00-Inf]	1.000	1.000
VWA5B2	101	0	0.00%	196	0	0.00%	1.00 [0e+00-Inf]	1.000	1.000
VWCE	101	0	0.00%	196	0	0.00%	1.00 [0e+00-Inf]	1.000	1.000
WBP2	101	0	0.00%	196	0	0.00%	1.00 [0e+00-Inf]	1.000	1.000
WDR24	101	0	0.00%	196	0	0.00%	1.00 [0e+00-Inf]	1.000	1.000
WDR26	101	0	0.00%	196	0	0.00%	1.00 [0e+00-Inf]	1.000	1.000
WDR45B	101	0	0.00%	196	0	0.00%	1.00 [0e+00-Inf]	1.000	1.000
WDR53	101	0	0.00%	196	0	0.00%	1.00 [0e+00-Inf]	1.000	1.000
WDR64	101	0	0.00%	196	0	0.00%	1.00 [0e+00-Inf]	1.000	1.000
TBC1D31	101	0	0.00%	196	0	0.00%	1.00 [0e+00-Inf]	1.000	1.000
WDR73	101	0	0.00%	196	0	0.00%	1.00 [0e+00-Inf]	1.000	1.000
WDR90	101	0	0.00%	196	0	0.00%	1.00 [0e+00-Inf]	1.000	1.000
WDR93	101	0	0.00%	196	0	0.00%	1.00 [0e+00-Inf]	1.000	1.000
WDYHV1	101	0	0.00%	196	0	0.00%	1.00 [0e+00-Inf]	1.000	1.000
WFDC10A	101	0	0.00%	196	0	0.00%	1.00 [0e+00-Inf]	1.000	1.000
WFDC10B	101	0	0.00%	196	0	0.00%	1.00 [0e+00-Inf]	1.000	1.000
WFDC11	101	0	0.00%	196	0	0.00%	1.00 [0e+00-Inf]	1.000	1.000
WFDC12	101	0	0.00%	196	0	0.00%	1.00 [0e+00-Inf]	1.000	1.000
WFDC13	101	0	0.00%	196	0	0.00%	1.00 [0e+00-Inf]	1.000	1.000
WFDC2	101	0	0.00%	196	0	0.00%	1.00 [0e+00-Inf]	1.000	1.000
WFDC3	101	0	0.00%	196	0	0.00%	1.00 [0e+00-Inf]	1.000	1.000
WFDC5	101	0	0.00%	196	0	0.00%	1.00 [0e+00-Inf]	1.000	1.000
WFDC6	101	0	0.00%	196	0	0.00%	1.00 [0e+00-Inf]	1.000	1.000
WFDC8	101	0	0.00%	196	0	0.00%	1.00 [0e+00-Inf]	1.000	1.000
WFDC9	101	0	0.00%	196	0	0.00%	1.00 [0e+00-Inf]	1.000	1.000
WFIKKN1	101	0	0.00%	196	0	0.00%	1.00 [0e+00-Inf]	1.000	1.000
WFIKKN2	101	0	0.00%	196	0	0.00%	1.00 [0e+00-Inf]	1.000	1.000
WHAMM	101	0	0.00%	196	0	0.00%	1.00 [0e+00-Inf]	1.000	1.000
WIF1	101	0	0.00%	196	0	0.00%	1.00 [0e+00-Inf]	1.000	1.000
WIPF2	101	0	0.00%	196	0	0.00%	1.00 [0e+00-Inf]	1.000	1.000
WIPF3	101	0	0.00%	196	0	0.00%	1.00 [0e+00-Inf]	1.000	1.000
WIPI1	101	0	0.00%	196	0	0.00%	1.00 [0e+00-Inf]	1.000	1.000
WNT11	101	0	0.00%	196	0	0.00%	1.00 [0e+00-Inf]	1.000	1.000
WNT3A	101	0	0.00%	196	0	0.00%	1.00 [0e+00-Inf]	1.000	1.000
WNT3	101	0	0.00%	196	0	0.00%	1.00 [0e+00-Inf]	1.000	1.000
WNT9A	101	0	0.00%	196	0	0.00%	1.00 [0e+00-Inf]	1.000	1.000

Symbol	CINSARC Low			CINSARC High			CINSARC High vs. Low		
	N tested	N altered	Fq alteration	N tested	N altered	Fq alteration	OR [95%CI]	p-value*	q-value
WNT9B	101	0	0.00%	196	0	0.00%	1.00 [0e+00-Inf]	1.000	1.000
WRN	101	0	0.00%	196	0	0.00%	1.00 [0e+00-Inf]	1.000	1.000
WSB1	101	0	0.00%	196	0	0.00%	1.00 [0e+00-Inf]	1.000	1.000
WWP1	101	0	0.00%	196	0	0.00%	1.00 [0e+00-Inf]	1.000	1.000
XCL1	101	0	0.00%	196	0	0.00%	1.00 [0e+00-Inf]	1.000	1.000
XCL2	101	0	0.00%	196	0	0.00%	1.00 [0e+00-Inf]	1.000	1.000
XKR4	101	0	0.00%	196	0	0.00%	1.00 [0e+00-Inf]	1.000	1.000
XKR9	101	0	0.00%	196	0	0.00%	1.00 [0e+00-Inf]	1.000	1.000
XKRY2	101	65	64.36%	196	117	59.69%	0.82 [0.54-1.25]	0.435	1.000
XKRY	101	65	64.36%	196	117	59.69%	0.82 [0.54-1.25]	0.435	1.000
XPO6	101	0	0.00%	196	0	0.00%	1.00 [0e+00-Inf]	1.000	1.000
XPOT	101	0	0.00%	196	0	0.00%	1.00 [0e+00-Inf]	1.000	1.000
XPR1	101	0	0.00%	196	0	0.00%	1.00 [0e+00-Inf]	1.000	1.000
XRRA1	101	0	0.00%	196	0	0.00%	1.00 [0e+00-Inf]	1.000	1.000
XYLT1	101	0	0.00%	196	0	0.00%	1.00 [0e+00-Inf]	1.000	1.000
XYLT2	101	0	0.00%	196	0	0.00%	1.00 [0e+00-Inf]	1.000	1.000
YEATS2	101	0	0.00%	196	0	0.00%	1.00 [0e+00-Inf]	1.000	1.000
YEATS4	101	0	0.00%	196	0	0.00%	1.00 [0e+00-Inf]	1.000	1.000
YIF1A	101	0	0.00%	196	0	0.00%	1.00 [0e+00-Inf]	1.000	1.000
YKT6	101	0	0.00%	196	0	0.00%	1.00 [0e+00-Inf]	1.000	1.000
YOD1	101	0	0.00%	196	0	0.00%	1.00 [0e+00-Inf]	1.000	1.000
YPEL2	101	0	0.00%	196	0	0.00%	1.00 [0e+00-Inf]	1.000	1.000
YPEL3	101	0	0.00%	196	0	0.00%	1.00 [0e+00-Inf]	1.000	1.000
YTHDF1	101	0	0.00%	196	0	0.00%	1.00 [0e+00-Inf]	1.000	1.000
YTHDF3	101	0	0.00%	196	0	0.00%	1.00 [0e+00-Inf]	1.000	1.000
YWHAB	101	0	0.00%	196	0	0.00%	1.00 [0e+00-Inf]	1.000	1.000
YWHAZ	101	0	0.00%	196	0	0.00%	1.00 [0e+00-Inf]	1.000	1.000
YY1AP1	101	0	0.00%	196	0	0.00%	1.00 [0e+00-Inf]	1.000	1.000
ZACN	101	0	0.00%	196	0	0.00%	1.00 [0e+00-Inf]	1.000	1.000
ZBP1	101	0	0.00%	196	0	0.00%	1.00 [0e+00-Inf]	1.000	1.000
ZBTB10	101	0	0.00%	196	0	0.00%	1.00 [0e+00-Inf]	1.000	1.000
ZBTB37	101	0	0.00%	196	0	0.00%	1.00 [0e+00-Inf]	1.000	1.000
ZBTB41	101	0	0.00%	196	0	0.00%	1.00 [0e+00-Inf]	1.000	1.000
ZBTB46	101	0	0.00%	196	0	0.00%	1.00 [0e+00-Inf]	1.000	1.000
ZBTB7B	101	0	0.00%	196	0	0.00%	1.00 [0e+00-Inf]	1.000	1.000
ZC3H11A	101	0	0.00%	196	0	0.00%	1.00 [0e+00-Inf]	1.000	1.000
ZC3H3	101	0	0.00%	196	0	0.00%	1.00 [0e+00-Inf]	1.000	1.000
ZC3H7A	101	0	0.00%	196	0	0.00%	1.00 [0e+00-Inf]	1.000	1.000
ZCCHC2	101	0	0.00%	196	0	0.00%	1.00 [0e+00-Inf]	1.000	1.000
ZDHHC17	101	0	0.00%	196	0	0.00%	1.00 [0e+00-Inf]	1.000	1.000
ZDHHC19	101	0	0.00%	196	0	0.00%	1.00 [0e+00-Inf]	1.000	1.000
ZDHHC24	101	0	0.00%	196	0	0.00%	1.00 [0e+00-Inf]	1.000	1.000
ZFAND1	101	0	0.00%	196	0	0.00%	1.00 [0e+00-Inf]	1.000	1.000
ZFAT-AS1	101	0	0.00%	196	0	0.00%	1.00 [0e+00-Inf]	1.000	1.000
ZFAT	101	0	0.00%	196	0	0.00%	1.00 [0e+00-Inf]	1.000	1.000
ZFC3H1	101	0	0.00%	196	0	0.00%	1.00 [0e+00-Inf]	1.000	1.000
ZFHX4	101	0	0.00%	196	0	0.00%	1.00 [0e+00-Inf]	1.000	1.000
ZFP41	101	0	0.00%	196	0	0.00%	1.00 [0e+00-Inf]	1.000	1.000
ZFP64	101	0	0.00%	196	0	0.00%	1.00 [0e+00-Inf]	1.000	1.000
ZFPM2	101	0	0.00%	196	0	0.00%	1.00 [0e+00-Inf]	1.000	1.000
ZFY	101	60	59.41%	196	106	54.08%	0.80 [0.54-1.21]	0.382	1.000
ZG16B	101	0	0.00%	196	0	0.00%	1.00 [0e+00-Inf]	1.000	1.000
ZG16	101	0	0.00%	196	0	0.00%	1.00 [0e+00-Inf]	1.000	1.000
ZGPAT	101	0	0.00%	196	0	0.00%	1.00 [0e+00-Inf]	1.000	1.000
ZHX1	101	0	0.00%	196	0	0.00%	1.00 [0e+00-Inf]	1.000	1.000
ZHX2	101	0	0.00%	196	0	0.00%	1.00 [0e+00-Inf]	1.000	1.000
ZHX3	101	0	0.00%	196	0	0.00%	1.00 [0e+00-Inf]	1.000	1.000
ZKSCAN2	101	0	0.00%	196	0	0.00%	1.00 [0e+00-Inf]	1.000	1.000
ZMAT3	101	0	0.00%	196	0	0.00%	1.00 [0e+00-Inf]	1.000	1.000
ZMAT4	101	0	0.00%	196	0	0.00%	1.00 [0e+00-Inf]	1.000	1.000
MSS51	101	0	0.00%	196	0	0.00%	1.00 [0e+00-Inf]	1.000	1.000
ZMYND8	101	0	0.00%	196	0	0.00%	1.00 [0e+00-Inf]	1.000	1.000
ZNF124	101	0	0.00%	196	0	0.00%	1.00 [0e+00-Inf]	1.000	1.000
ZNF160	101	0	0.00%	196	0	0.00%	1.00 [0e+00-Inf]	1.000	1.000
ZNF16	101	0	0.00%	196	0	0.00%	1.00 [0e+00-Inf]	1.000	1.000
ZNF174	101	0	0.00%	196	0	0.00%	1.00 [0e+00-Inf]	1.000	1.000
ZNF200	101	0	0.00%	196	0	0.00%	1.00 [0e+00-Inf]	1.000	1.000
ZNF205	101	0	0.00%	196	0	0.00%	1.00 [0e+00-Inf]	1.000	1.000
ZNF213	101	0	0.00%	196	0	0.00%	1.00 [0e+00-Inf]	1.000	1.000
ZNF217	101	0	0.00%	196	0	0.00%	1.00 [0e+00-Inf]	1.000	1.000
ZBTB18	101	0	0.00%	196	0	0.00%	1.00 [0e+00-Inf]	1.000	1.000

Symbol	CINSARC Low			CINSARC High			CINSARC High vs. Low		
	N tested	N altered	Fq alteration	N tested	N altered	Fq alteration	OR [95%CI]	p-value*	q-value
ZNF250	101	0	0.00%	196	0	0.00%	1.00 [0e+00-Inf]	1.000	1.000
ZNF251	101	0	0.00%	196	0	0.00%	1.00 [0e+00-Inf]	1.000	1.000
ZNF252P	101	0	0.00%	196	0	0.00%	1.00 [0e+00-Inf]	1.000	1.000
ZNF263	101	0	0.00%	196	0	0.00%	1.00 [0e+00-Inf]	1.000	1.000
ZNF267	101	0	0.00%	196	0	0.00%	1.00 [0e+00-Inf]	1.000	1.000
ZNF281	101	0	0.00%	196	0	0.00%	1.00 [0e+00-Inf]	1.000	1.000
ZNF311	101	0	0.00%	196	0	0.00%	1.00 [0e+00-Inf]	1.000	1.000
ZNF334	101	0	0.00%	196	0	0.00%	1.00 [0e+00-Inf]	1.000	1.000
ZNF335	101	0	0.00%	196	0	0.00%	1.00 [0e+00-Inf]	1.000	1.000
ZNF347	101	0	0.00%	196	0	0.00%	1.00 [0e+00-Inf]	1.000	1.000
ZNF34	101	0	0.00%	196	0	0.00%	1.00 [0e+00-Inf]	1.000	1.000
ZNF350	101	0	0.00%	196	0	0.00%	1.00 [0e+00-Inf]	1.000	1.000
ZNF415	101	0	0.00%	196	0	0.00%	1.00 [0e+00-Inf]	1.000	1.000
ZSCAN32	101	0	0.00%	196	0	0.00%	1.00 [0e+00-Inf]	1.000	1.000
ZNF468	101	0	0.00%	196	0	0.00%	1.00 [0e+00-Inf]	1.000	1.000
ZNF48	101	0	0.00%	196	0	0.00%	1.00 [0e+00-Inf]	1.000	1.000
ZNF496	101	0	0.00%	196	0	0.00%	1.00 [0e+00-Inf]	1.000	1.000
ZNF500	101	0	0.00%	196	0	0.00%	1.00 [0e+00-Inf]	1.000	1.000
ZNF503	101	0	0.00%	196	0	0.00%	1.00 [0e+00-Inf]	1.000	1.000
ZNF512B	101	0	0.00%	196	0	0.00%	1.00 [0e+00-Inf]	1.000	1.000
ZNF517	101	0	0.00%	196	0	0.00%	1.00 [0e+00-Inf]	1.000	1.000
ZNF524	101	0	0.00%	196	0	0.00%	1.00 [0e+00-Inf]	1.000	1.000
ZNF532	101	0	0.00%	196	0	0.00%	1.00 [0e+00-Inf]	1.000	1.000
ZNF572	101	0	0.00%	196	0	0.00%	1.00 [0e+00-Inf]	1.000	1.000
ZNF579	101	0	0.00%	196	0	0.00%	1.00 [0e+00-Inf]	1.000	1.000
ZNF580	101	0	0.00%	196	0	0.00%	1.00 [0e+00-Inf]	1.000	1.000
ZNF581	101	0	0.00%	196	0	0.00%	1.00 [0e+00-Inf]	1.000	1.000
ZNF592	101	0	0.00%	196	0	0.00%	1.00 [0e+00-Inf]	1.000	1.000
ZNF597	101	0	0.00%	196	0	0.00%	1.00 [0e+00-Inf]	1.000	1.000
ZNF598	101	0	0.00%	196	0	0.00%	1.00 [0e+00-Inf]	1.000	1.000
ZNF613	101	0	0.00%	196	0	0.00%	1.00 [0e+00-Inf]	1.000	1.000
ZNF614	101	0	0.00%	196	0	0.00%	1.00 [0e+00-Inf]	1.000	1.000
ZNF615	101	0	0.00%	196	0	0.00%	1.00 [0e+00-Inf]	1.000	1.000
ZNF623	101	0	0.00%	196	0	0.00%	1.00 [0e+00-Inf]	1.000	1.000
ZNF628	101	0	0.00%	196	0	0.00%	1.00 [0e+00-Inf]	1.000	1.000
ZNF629	101	0	0.00%	196	0	0.00%	1.00 [0e+00-Inf]	1.000	1.000
ZNF639	101	0	0.00%	196	0	0.00%	1.00 [0e+00-Inf]	1.000	1.000
ZNF646	101	0	0.00%	196	0	0.00%	1.00 [0e+00-Inf]	1.000	1.000
ZNF648	101	0	0.00%	196	0	0.00%	1.00 [0e+00-Inf]	1.000	1.000
ZNF649	101	0	0.00%	196	0	0.00%	1.00 [0e+00-Inf]	1.000	1.000
ZNF652	101	0	0.00%	196	0	0.00%	1.00 [0e+00-Inf]	1.000	1.000
ZNF665	101	0	0.00%	196	0	0.00%	1.00 [0e+00-Inf]	1.000	1.000
ZNF668	101	0	0.00%	196	0	0.00%	1.00 [0e+00-Inf]	1.000	1.000
ZNF669	101	0	0.00%	196	0	0.00%	1.00 [0e+00-Inf]	1.000	1.000
ZNF670	101	0	0.00%	196	0	0.00%	1.00 [0e+00-Inf]	1.000	1.000
ZNF672	101	0	0.00%	196	0	0.00%	1.00 [0e+00-Inf]	1.000	1.000
ZNF678	101	0	0.00%	196	0	0.00%	1.00 [0e+00-Inf]	1.000	1.000
ZNF687	101	0	0.00%	196	0	0.00%	1.00 [0e+00-Inf]	1.000	1.000
ZNF688	101	0	0.00%	196	0	0.00%	1.00 [0e+00-Inf]	1.000	1.000
ZNF689	101	0	0.00%	196	0	0.00%	1.00 [0e+00-Inf]	1.000	1.000
ZNF692	101	0	0.00%	196	0	0.00%	1.00 [0e+00-Inf]	1.000	1.000
ZNF695	101	0	0.00%	196	0	0.00%	1.00 [0e+00-Inf]	1.000	1.000
ZNF696	101	0	0.00%	196	0	0.00%	1.00 [0e+00-Inf]	1.000	1.000
ZNF703	101	0	0.00%	196	0	0.00%	1.00 [0e+00-Inf]	1.000	1.000
ZNF704	101	0	0.00%	196	0	0.00%	1.00 [0e+00-Inf]	1.000	1.000
ZNF706	101	0	0.00%	196	0	0.00%	1.00 [0e+00-Inf]	1.000	1.000
ZNF707	101	0	0.00%	196	0	0.00%	1.00 [0e+00-Inf]	1.000	1.000
ZNF710	101	0	0.00%	196	0	0.00%	1.00 [0e+00-Inf]	1.000	1.000
ZNF720	101	0	0.00%	196	0	0.00%	1.00 [0e+00-Inf]	1.000	1.000
ZNF747	101	0	0.00%	196	0	0.00%	1.00 [0e+00-Inf]	1.000	1.000
ZNF750	101	0	0.00%	196	0	0.00%	1.00 [0e+00-Inf]	1.000	1.000
ZNF75A	101	0	0.00%	196	0	0.00%	1.00 [0e+00-Inf]	1.000	1.000
ZNF764	101	0	0.00%	196	0	0.00%	1.00 [0e+00-Inf]	1.000	1.000
ZNF768	101	0	0.00%	196	0	0.00%	1.00 [0e+00-Inf]	1.000	1.000
ZNF771	101	0	0.00%	196	0	0.00%	1.00 [0e+00-Inf]	1.000	1.000
ZNF774	101	0	0.00%	196	0	0.00%	1.00 [0e+00-Inf]	1.000	1.000
ZNF784	101	0	0.00%	196	0	0.00%	1.00 [0e+00-Inf]	1.000	1.000
ZNF785	101	0	0.00%	196	0	0.00%	1.00 [0e+00-Inf]	1.000	1.000
ZNF7	101	0	0.00%	196	0	0.00%	1.00 [0e+00-Inf]	1.000	1.000
ZNF830	101	0	0.00%	196	0	0.00%	1.00 [0e+00-Inf]	1.000	1.000
ZNF831	101	0	0.00%	196	0	0.00%	1.00 [0e+00-Inf]	1.000	1.000

Symbol	CINSARC Low			CINSARC High			CINSARC High vs. Low		
	N tested	N altered	Fq alteration	N tested	N altered	Fq alteration	OR [95%CI]	p-value*	q-value
ZNF843	101	0	0.00%	196	0	0.00%	1.00 [0e+00-Inf]	1.000	1.000
ZNFX1	101	0	0.00%	196	0	0.00%	1.00 [0e+00-Inf]	1.000	1.000
ZNHIT3	101	0	0.00%	196	0	0.00%	1.00 [0e+00-Inf]	1.000	1.000
ZNRF2	101	0	0.00%	196	0	0.00%	1.00 [0e+00-Inf]	1.000	1.000
ZP2	101	0	0.00%	196	0	0.00%	1.00 [0e+00-Inf]	1.000	1.000
ZP4	101	0	0.00%	196	0	0.00%	1.00 [0e+00-Inf]	1.000	1.000
ZPBP2	101	0	0.00%	196	0	0.00%	1.00 [0e+00-Inf]	1.000	1.000
ZSCAN10	101	0	0.00%	196	0	0.00%	1.00 [0e+00-Inf]	1.000	1.000
ZSCAN2	101	0	0.00%	196	0	0.00%	1.00 [0e+00-Inf]	1.000	1.000
ZSWIM1	101	0	0.00%	196	0	0.00%	1.00 [0e+00-Inf]	1.000	1.000
ZSWIM3	101	0	0.00%	196	0	0.00%	1.00 [0e+00-Inf]	1.000	1.000

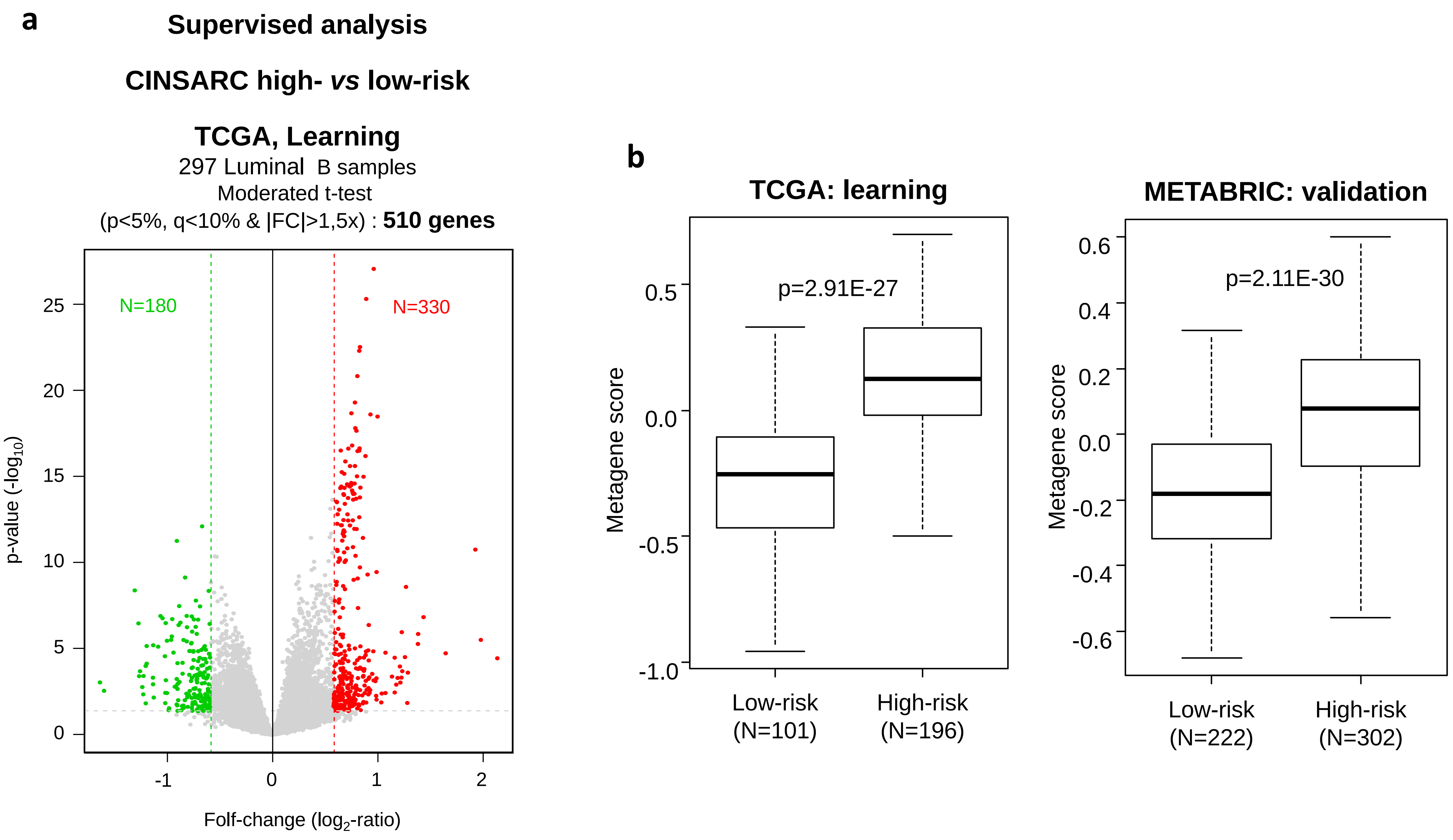

Supplementary Figure 1: Identification and validation of 510 genes differentially expressed between the two CINSARC classes in Luminal B breast cancers.

a/ Identification of the signature in the TCGA data set (N=297). Volcano-plot showing the 510 genes differentially expressed between the two CINSARC classes. b/ Box plot of the 510-gene metagene in the learning set (TCGA) and the validation set (METABRIC). The p-value is for the Student t-test.

Supplementary Table 7: List of 510 genes differentially expressed between the two CINSARC classes in Luminal B breast cancers.

Symbol	description	Cytoband	Entrez Gene ID	Low-risk (mean, log2)	High-risk (mean, log2)	High vs.Low (log2-ratio)	t	p-value	q-value	Associated to
CENPA	centromere protein A	2p23.3	1058	6.31	7.27	0.97	12.13	8.31E-28	8.89E-24	up CINSARC High vs. Low
BUB1	BUB1 mitotic checkpoint se	2q13	699	8.41	9.31	0.90	11.64	4.63E-26	2.48E-22	up CINSARC High vs. Low
NCAPH	non-SMC condensin I com	2q11.2	23397	7.86	8.70	0.84	10.83	2.88E-23	1.03E-19	up CINSARC High vs. Low
HJURP	Holliday junction recognitio	2q37.1	55355	7.66	8.49	0.83	10.76	4.80E-23	1.28E-19	up CINSARC High vs. Low
CCNA2	cyclin A2	4q27	890	8.46	9.27	0.81	10.33	1.42E-21	3.03E-18	up CINSARC High vs. Low
CCNB2	cyclin B2	15q22.2	9133	8.35	9.14	0.79	9.86	4.83E-20	8.61E-17	up CINSARC High vs. Low
CEP55	centrosomal protein 55	10q23.33	55165	8.28	9.04	0.76	9.67	2.05E-19	3.13E-16	up CINSARC High vs. Low
BIRC5	baculoviral IAP repeat con	17q25.3	332	8.12	9.06	0.94	9.65	2.40E-19	3.21E-16	up CINSARC High vs. Low
KIF14	kinesin family member 14	1q32.1	9928	7.21	8.22	1.00	9.61	3.17E-19	3.76E-16	up CINSARC High vs. Low
FOXM1	forkhead box M1	12p13.33	2305	9.45	10.24	0.79	9.40	1.49E-18	1.59E-15	up CINSARC High vs. Low
DEPDC1B	DEP domain containing 1B	5q12.1	55789	6.56	7.36	0.80	9.35	2.13E-18	2.07E-15	up CINSARC High vs. Low
KPNA2	karyopherin subunit alpha	17q24.2	3838	11.20	11.96	0.76	9.08	1.53E-17	1.36E-14	up CINSARC High vs. Low
TTK	TTK protein kinase	6q14.1	7272	7.50	8.34	0.83	9.03	2.23E-17	1.77E-14	up CINSARC High vs. Low
NCAPG	non-SMC condensin I com	4p15.31	64151	8.52	9.25	0.73	9.02	2.31E-17	1.77E-14	up CINSARC High vs. Low
KIF23	kinesin family member 23	15q23	9493	8.77	9.42	0.65	8.99	3.00E-17	2.03E-14	up CINSARC High vs. Low
CKAP2L	cytoskeleton associated pr	2q14.1	150468	7.10	7.93	0.83	8.98	3.04E-17	2.03E-14	up CINSARC High vs. Low
CENPE	centromere protein E	4q24	1062	8.29	9.10	0.81	8.98	3.22E-17	2.03E-14	up CINSARC High vs. Low
TROAP	trophinin associated protei	12q13.12	10024	7.52	8.41	0.89	8.88	6.23E-17	3.70E-14	up CINSARC High vs. Low
BUB1B	BUB1 mitotic checkpoint se	15q15.1	701	8.39	9.09	0.70	8.78	1.28E-16	7.23E-14	up CINSARC High vs. Low
AURKA	aurora kinase A	20q13.2	6790	8.54	9.33	0.79	8.69	2.38E-16	1.21E-13	up CINSARC High vs. Low
KIF2C	kinesin family member 2C	1p34.1	11004	8.46	9.21	0.74	8.69	2.38E-16	1.21E-13	up CINSARC High vs. Low
PRC1	protein regulator of cytokin	15q26.1	9055	9.95	10.61	0.66	8.58	5.38E-16	2.62E-13	up CINSARC High vs. Low
PARPBP	PARP1 binding protein	12q23.2	55010	6.93	7.62	0.69	8.55	6.71E-16	3.12E-13	up CINSARC High vs. Low
SGO1	shugoshin 1	3p24.3	151648	6.11	6.92	0.81	8.50	9.42E-16	4.20E-13	up CINSARC High vs. Low
TICRR	TOPBP1 interacting check	15q26.1	90381	6.74	7.61	0.87	8.49	9.94E-16	4.26E-13	up CINSARC High vs. Low
NUF2	NUF2 component of NDC8	1q23.3	83540	7.89	8.64	0.75	8.37	2.28E-15	9.38E-13	up CINSARC High vs. Low
DLGAP5	DLG associated protein 5	14q22.3	9787	7.89	8.68	0.79	8.36	2.49E-15	9.85E-13	up CINSARC High vs. Low
ARHGAP11A	Rho GTPase activating pro	15q13.3	9824	8.49	9.20	0.71	8.34	2.74E-15	1.05E-12	up CINSARC High vs. Low
SPAG5	sperm associated antigen	17q11.2	10615	9.31	10.03	0.72	8.32	3.14E-15	1.12E-12	up CINSARC High vs. Low
CENPF	centromere protein F	1q41	1063	10.73	11.49	0.75	8.32	3.15E-15	1.12E-12	up CINSARC High vs. Low
BLM	BLM RecQ like helicase	15q26.1	641	7.11	7.85	0.74	8.30	3.69E-15	1.24E-12	up CINSARC High vs. Low
KIF20A	kinesin family member 20A	5q31.2	10112	8.82	9.48	0.66	8.30	3.71E-15	1.24E-12	up CINSARC High vs. Low
MCM10	minichromosome maintena	10p13	55388	7.27	8.11	0.84	8.28	4.26E-15	1.38E-12	up CINSARC High vs. Low
MAD2L1	mitotic arrest deficient 2 lik	4q27	4085	8.44	9.13	0.69	8.27	4.46E-15	1.39E-12	up CINSARC High vs. Low
KIF4A	kinesin family member 4A	Xq13.1	24137	8.81	9.46	0.65	8.27	4.54E-15	1.39E-12	up CINSARC High vs. Low
TRIP13	thyroid hormone receptor i	5p15.33	9319	8.09	8.85	0.76	8.22	6.22E-15	1.85E-12	up CINSARC High vs. Low
CDCA3	cell division cycle associat	12p13.31	83461	7.37	8.14	0.77	8.18	8.17E-15	2.36E-12	up CINSARC High vs. Low
MTFR2	mitochondrial fission regula	6q23.3	113115	5.61	6.40	0.78	8.16	9.79E-15	2.72E-12	up CINSARC High vs. Low
SKA1	spindle and kinetochore as	18q21.1	220134	6.85	7.62	0.77	8.15	1.01E-14	2.72E-12	up CINSARC High vs. Low
GTSE1	G2 and S-phase expresse	22q13.31	51512	7.37	8.05	0.68	8.15	1.02E-14	2.72E-12	up CINSARC High vs. Low
NDC80	NDC80 kinetochore compl	18p11.32	10403	7.75	8.43	0.68	8.13	1.14E-14	2.98E-12	up CINSARC High vs. Low
UBE2C	ubiquitin conjugating enzyr	20q13.12	11065	8.89	9.72	0.84	8.08	1.59E-14	4.05E-12	up CINSARC High vs. Low
SKA3	spindle and kinetochore as	13q12.11	221150	7.13	7.85	0.72	8.07	1.72E-14	4.27E-12	up CINSARC High vs. Low
ASPM	abnormal spindle microtub	1q31.3	259266	8.81	9.61	0.80	8.06	1.90E-14	4.62E-12	up CINSARC High vs. Low
RRM2	ribonucleotide reductase re	2p25.1	6241	9.58	10.35	0.77	8.04	2.14E-14	5.08E-12	up CINSARC High vs. Low
TPX2	TPX2 microtubule nucleati	20q11.21	22974	10.26	10.87	0.61	8.00	2.79E-14	6.36E-12	up CINSARC High vs. Low
CDCA8	cell division cycle associat	1p34.3	55143	8.30	8.92	0.62	7.99	3.02E-14	6.73E-12	up CINSARC High vs. Low
CDK1	cyclin dependent kinase 1	10q21.2	983	9.53	10.22	0.69	7.96	3.76E-14	8.03E-12	up CINSARC High vs. Low
ECT2	epithelial cell transforming	3q26.31	1894	9.89	10.53	0.64	7.84	8.24E-14	1.69E-11	up CINSARC High vs. Low
KIF11	kinesin family member 11	10q23.33	3832	9.40	10.02	0.62	7.74	1.52E-13	3.02E-11	up CINSARC High vs. Low
POLQ	DNA polymerase theta	3q13.33	10721	6.91	7.63	0.72	7.74	1.52E-13	3.02E-11	up CINSARC High vs. Low
FAM72B	family with sequence simila	1p11.2	653820	6.47	7.30	0.83	7.68	2.25E-13	4.37E-11	up CINSARC High vs. Low
NEK2	NIMA related kinase 2	1q32.3	4751	8.78	9.46	0.68	7.63	3.28E-13	6.26E-11	up CINSARC High vs. Low
KIF18B	kinesin family member 18B	17q21.31	146909	7.37	8.14	0.77	7.62	3.41E-13	6.41E-11	up CINSARC High vs. Low
DIAPH3	diaphanous related formin	13q21.2	81624	7.04	7.77	0.72	7.62	3.50E-13	6.46E-11	up CINSARC High vs. Low
RAD51AP1	RAD51 associated protein	12p13.32	10635	7.48	8.10	0.62	7.55	5.43E-13	9.84E-11	up CINSARC High vs. Low
CDKN3	cyclin dependent kinase in	14q22.2	1033	7.14	7.79	0.66	7.52	6.54E-13	1.15E-10	up CINSARC High vs. Low
PLK1	polo like kinase 1	16p12.2	5347	8.74	9.40	0.66	7.52	6.56E-13	1.15E-10	up CINSARC High vs. Low
DSCC1	DNA replication and sister	8q24.12	79075	7.22	7.97	0.74	7.51	6.78E-13	1.17E-10	up CINSARC High vs. Low
AURKB	aurora kinase B	17p13.1	9212	7.05	7.84	0.78	7.44	1.06E-12	1.78E-10	up CINSARC High vs. Low
TOP2A	DNA topoisomerase II alph	17q21.2	7153	11.32	12.12	0.80	7.44	1.09E-12	1.80E-10	up CINSARC High vs. Low
MELK	maternal embryonic leucin	9p13.2	9833	8.03	8.71	0.68	7.41	1.30E-12	2.10E-10	up CINSARC High vs. Low
PTTG1	PTTG1 regulator of sister c	5q33.3	9232	8.30	8.98	0.68	7.40	1.37E-12	2.19E-10	up CINSARC High vs. Low
MKI67	marker of proliferation Ki-6	10q26.2	4288	10.61	11.30	0.69	7.38	1.56E-12	2.46E-10	up CINSARC High vs. Low
ESPL1	extra spindle pole bodies li	12q13.13	9700	8.30	8.98	0.68	7.37	1.65E-12	2.56E-10	up CINSARC High vs. Low
ANLN	anillin actin binding protein	7p14.2	54443	9.17	9.84	0.67	7.34	2.10E-12	3.16E-10	up CINSARC High vs. Low
SHCBP1	SHC binding and spindle a	16q11.2	79801	7.43	8.12	0.69	7.29	2.86E-12	4.25E-10	up CINSARC High vs. Low
FAM72A	family with sequence simila	1q32.1	729533	4.65	5.51	0.86	7.25	3.59E-12	5.12E-10	up CINSARC High vs. Low
PIMREG	PICALM interacting mitotic	17p13.2	54478	6.39	7.06	0.67	7.19	5.15E-12	7.25E-10	up CINSARC High vs. Low
FAM83D	family with sequence simila	20q11.23	81610	7.92	8.69	0.77	7.05	1.22E-11	1.67E-09	up CINSARC High vs. Low
CIP2A	cell proliferation regulating	3q13.13	57650	7.28	8.00	0.72	7.03	1.43E-11	1.94E-09	up CINSARC High vs. Low
INAVA	innate immunity activator	1q32.1	55765	5.13	7.06	1.93	7.00	1.71E-11	2.29E-09	up CINSARC High vs. Low
IQGAP3	IQ motif containing GTPas	1q22	128239	9.13	9.75	0.62	7.00	1.75E-11	2.31E-09	up CINSARC High vs. Low
UBE2T	ubiquitin conjugating enzyr	1q32.1	29089	8.56	9.18	0.62	6.97	2.08E-11	2.71E-09	up CINSARC High vs. Low
CDC20	cell division cycle 20	1p34.2	991	8.68	9.36	0.69	6.94	2.49E-11	3.21E-09	up CINSARC High vs. Low
FAM72D	family with sequence simila	1q21.1	728833	5.55	6.35	0.79	6.86	3.90E-11	4.91E-09	up CINSARC High vs. Low
HASPIN	histone H3 associated prot	17p13.2	83903	5.45	6.09	0.64	6.81	5.42E-11	6.52E-09	up CINSARC High vs. Low
NEIL3	nei like DNA glycosylase 3	4q34.3	55247	5.77	6.42	0.65	6.78	6.62E-11	7.87E-09	up CINSARC High vs. Low
RTKN2	rhotekin 2	10q21.2	219790	6.95	7.66	0.70	6.76	7.18E-11	8.44E-09	up CINSARC High vs. Low
KIF18A	kinesin family member 18A	11p14.1	81930	7.07	7.70	0.63	6.73	8.86E-11	1.01E-08	up CINSARC High vs. Low
DEPDC1	DEP domain containing 1	1p31.3	55635	7.48	8.17	0.69	6.73	8.95E-11	1.01E-08	up CINSARC High vs. Low
PIF1	PIF1 5'-to-3' DNA helicase	15q22.31	80119	4.56	5.39	0.84	6.60	1.85E-10	2.06E-08	up CINSARC High vs. Low
PRR11	proline rich 11	17q22	55771	6.05	7.04	0.99	6.50	3.46E-10	3.74E-08	up CINSARC High vs. Low
MYBL2	MYB proto-oncogene like 2	20q13.12	4605	9.16	10.07	0.91	6.44	4.83E-10	5.16E-08	up CINSARC High vs. Low
E2F8	E2F transcription factor 8	11p15.1	79733	6.47	7.29	0.81	6.35	8.17E-10	8.40E-08	up CINSARC High vs. Low
SQLE	squalene epoxidase	8q24.13	6713	10.52	11.29	0.78	6.32	9.77E-10	9.95E-08	up CINSARC High vs. Low
EXO1	exonuclease 1	1q43	9156	7.57	8.18	0.62	6.27	1.29E-09	1.29E-07	up CINSARC High vs. Low
ORC1	origin recognition complex	1p32.3	4998	6.35	6.96	0.61	6.19	1.95E-09	1.88E-07	up CINSARC High vs. Low
CCNE2	cyclin E2	8q22.1	9134	7.90	8.58	0.68	6.17	2.24E-09	2.06E-07	up CINSARC High vs. Low
LAD1	ladinin 1	1q32.1	3898	7.98	9.25	1.27	6.15	2.53E-09	2.29E-07	up CINSARC High vs. Low
CENPI	centromere protein I	Xq22.1	2491	5.75	6.45	0.69	6.09	3.43E-09	2.98E-07	up CINSARC High vs. Low
ORC6	origin recognition complex	16q11.2	23594	6.62	7.26	0.64	5.85	1.33E-08	9.91E-07	up CINSARC High vs. Low
RAD54B	RAD54 homolog B	8q22.1	25788	7.33	7.93	0.60	5.81	1.64E-08	1.19E-06	up CINSARC High vs. Low
DNMT3B	DNA methyltransferase 3 b	20q11.21	1789	6.54	7.18	0.64	5.76	2.07E-08	1.46E-06	up CINSARC High vs. Low
CCNE1	cyclin E1	19q12	898	5.70	6.38	0.67	5.63	4.20E-08	2.74E-06	up CINSARC High vs. Low
MEX3A	mex-3 RNA binding family	1q22	92312	8.59	9.41	0.82	5.63	4.25E-08	2.76E-06	up CINSARC High vs. Low
CDC6	cell division cycle 6	17q21.2	990	8.65	9.25	0.60	5.53	6.87E-08	4.22E-06	up CINSARC High vs. Low
CTNND2	catenin delta 2	5p15.2	1501	6.84	8.28	1.44	5.39	1.44E-07	7.93E-06	up CINSARC High vs. Low
MMD	monocyte to macrophage c	17q22	23531	7.90	8.55	0.65	5.38	1.48E-07	8.11E-06	up CINSARC High vs. Low
KLF5	Kruppel like factor 5	13q22.1	688	6.93	7.85	0.92	5.18	4.14E-07	1.98E-05	up CINSARC High vs. Low
C18orf54	chromosome 18 open read	18q21.2	162681	6.52	7.15	0.63	5.07	6.94E-07	3.16E-05	up CINSARC High vs. Low
NMU	neuromedin U	4q12	10874	1.34	2.57	1.23	4.98	1.08E-06	4.55E-05	up CINSARC High vs. Low
E2F7	E2F transcription factor 7	12q21.2	144455	6.43	7.03	0.60	4.96	1.19E-06	4.97E-05	up CINSARC High vs. Low

Symbol	description	Cytoband	Entrez Gene ID	Low-risk (mean, log2)	High-risk (mean, log2)	High vs.Low (log2-ratio)	t	p-value	q-value	Associated to
GPX2	glutathione peroxidase 2	14q23.3	2877	2.79	4.18	1.39	4.93	1.37E-06	5.59E-05	up CINSARC High vs. Low
SINHCAF	SIN3-HDAC complex asso	12p11.21	58516	9.35	10.02	0.68	4.91	1.48E-06	5.85E-05	up CINSARC High vs. Low
CCDC150	coiled-coil domain containi	2q33.1	284992	4.00	4.66	0.65	4.91	1.48E-06	5.85E-05	up CINSARC High vs. Low
GAS2L3	growth arrest specific 2 like	12q23.1	283431	5.42	6.09	0.67	4.83	2.17E-06	8.19E-05	up CINSARC High vs. Low
MSMB	microseminoprotein beta	10q11.22	4477	4.28	6.27	1.99	4.76	3.03E-06	1.10E-04	up CINSARC High vs. Low
GFRA3	GDNF family receptor alph	5q31.2	2676	1.09	1.71	0.61	4.70	4.05E-06	1.38E-04	up CINSARC High vs. Low
PKP1	plakophilin 1	1q32.1	5317	4.88	6.27	1.39	4.64	5.21E-06	1.67E-04	up CINSARC High vs. Low
KNL1	kinetochore scaffold 1	15q15.1	57082	7.29	7.94	0.65	4.62	5.81E-06	1.83E-04	up CINSARC High vs. Low
RPRM	reprimo, TP53 dependent (	2q23.3	56475	0.88	1.61	0.73	4.59	6.42E-06	1.97E-04	up CINSARC High vs. Low
TAF4B	TATA-box binding protein a	18q11.2	6875	6.18	6.83	0.66	4.57	7.04E-06	2.12E-04	up CINSARC High vs. Low
AIF1L	allograft inflammatory facto	9q34.12-q34.13	83543	8.49	9.33	0.84	4.57	7.16E-06	2.15E-04	up CINSARC High vs. Low
GGH	gamma-glutamyl hydrolase	8q12.3	8836	8.17	8.96	0.79	4.51	9.45E-06	2.70E-04	up CINSARC High vs. Low
LRP8	LDL receptor related prote	1p32.3	7804	6.57	7.17	0.60	4.48	1.05E-05	2.92E-04	up CINSARC High vs. Low
ZC2HC1A	zinc finger C2HC-type con	8q21.13	51101	7.22	7.95	0.74	4.48	1.08E-05	2.98E-04	up CINSARC High vs. Low
MND1	meiotic nuclear divisions 1	4q31.3	84057	5.55	6.16	0.61	4.47	1.09E-05	2.99E-04	up CINSARC High vs. Low
UGT8	UDP glycosyltransferase 8	4q26	7368	2.13	3.04	0.92	4.45	1.23E-05	3.27E-04	up CINSARC High vs. Low
MYBL1	MYB proto-oncogene like 1	8q13.1	4603	7.85	8.54	0.69	4.42	1.39E-05	3.56E-04	up CINSARC High vs. Low
TNNI1	troponin I1, slow skeletal ty	1q32.1	7135	3.33	4.29	0.96	4.42	1.40E-05	3.58E-04	up CINSARC High vs. Low
CBX2	chromobox 2	17q25.3	84733	7.13	8.02	0.89	4.41	1.42E-05	3.61E-04	up CINSARC High vs. Low
CAMP	cathelicidin antimicrobial pe	3p21.31	820	2.71	3.79	1.08	4.38	1.68E-05	4.11E-04	up CINSARC High vs. Low
FAM3B	family with sequence simila	21q22.3	54097	4.54	6.19	1.65	4.36	1.82E-05	4.36E-04	up CINSARC High vs. Low
BRIP1	BRCA1 interacting protein	17q23.2	83990	6.94	7.54	0.59	4.35	1.85E-05	4.40E-04	up CINSARC High vs. Low
NFIB	nuclear factor I B	9p23-p22.3	4781	9.05	9.71	0.66	4.34	1.93E-05	4.53E-04	up CINSARC High vs. Low
PFN2	profilin 2	3q25.1	5217	10.33	10.93	0.60	4.33	2.03E-05	4.68E-04	up CINSARC High vs. Low
CNIH2	cornichon family AMPA rec	11q13.2	254263	5.59	6.23	0.64	4.32	2.15E-05	4.89E-04	up CINSARC High vs. Low
CTSV	cathepsin V	9q22.33	1515	5.69	6.32	0.63	4.31	2.25E-05	5.04E-04	up CINSARC High vs. Low
ZNF711	zinc finger protein 711	Xq21.1	7552	5.46	6.35	0.89	4.30	2.31E-05	5.16E-04	up CINSARC High vs. Low
GABBR2	gamma-aminobutyric acid t	9q22.33	9568	0.42	1.12	0.70	4.29	2.45E-05	5.43E-04	up CINSARC High vs. Low
SDK2	sidekick cell adhesion mole	17q25.1	54549	5.03	6.30	1.26	4.24	3.04E-05	6.46E-04	up CINSARC High vs. Low
RNF43	ring finger protein 43	17q22	54894	8.55	9.42	0.87	4.22	3.25E-05	6.80E-04	up CINSARC High vs. Low
VWDE	von Willebrand factor D an	7p21.3	221806	2.34	3.51	1.17	4.22	3.28E-05	6.82E-04	up CINSARC High vs. Low
CHRNA5	cholinergic receptor nicotin	15q25.1	1138	4.64	5.47	0.83	4.21	3.32E-05	6.89E-04	up CINSARC High vs. Low
PRAME	preferentially expressed ar	22q11.22	23532	3.19	5.33	2.14	4.20	3.54E-05	7.24E-04	up CINSARC High vs. Low
LAPTM4B	lysosomal protein transmer	8q22.1	55353	11.46	12.14	0.68	4.19	3.71E-05	7.53E-04	up CINSARC High vs. Low
FAM171A2	family with sequence simila	17q21.31	284069	4.86	5.54	0.67	4.18	3.83E-05	7.73E-04	up CINSARC High vs. Low
ACPP	acid phosphatase, prostate	3q22.1	55	4.19	4.88	0.69	4.17	4.03E-05	8.01E-04	up CINSARC High vs. Low
MYCN	MYCN proto-oncogene, bH	2p24.3	4613	3.38	4.19	0.81	4.14	4.57E-05	8.84E-04	up CINSARC High vs. Low
CHST9	carbohydrate sulfotransfera	18q11.2	83539	2.52	3.44	0.92	4.13	4.76E-05	9.10E-04	up CINSARC High vs. Low
DSC2	desmocollin 2	18q12.1	1824	8.68	9.41	0.73	4.10	5.23E-05	9.84E-04	up CINSARC High vs. Low
LOC148709	actin gamma 1 pseudogen	1q32.1	148709	3.38	4.03	0.65	4.10	5.43E-05	1.02E-03	up CINSARC High vs. Low
SLC7A5	solute carrier family 7 mem	16q24.2	8140	9.87	10.52	0.65	4.05	6.64E-05	1.19E-03	up CINSARC High vs. Low
PKHD1L1	PKHD1 like 1	8q23.1-q23.2	93035	1.30	1.97	0.67	4.04	6.70E-05	1.20E-03	up CINSARC High vs. Low
IKZF2	IKAROS family zinc finger 2	2q34	22807	7.06	7.84	0.79	4.03	7.11E-05	1.26E-03	up CINSARC High vs. Low
ADTRP	androgen dependent TFPI	6p24.1	84830	2.87	3.48	0.61	4.00	7.95E-05	1.36E-03	up CINSARC High vs. Low
CIB2	calcium and integrin bindin	15q25.1	10518	4.98	5.58	0.60	3.95	9.92E-05	1.61E-03	up CINSARC High vs. Low
FAM83A	family with sequence simila	8q24.13	84985	3.64	4.85	1.22	3.93	1.06E-04	1.69E-03	up CINSARC High vs. Low
STXBP5L	syntaxin binding protein 5 l	3q13.33	9515	1.00	1.84	0.84	3.89	1.22E-04	1.88E-03	up CINSARC High vs. Low
FSD1	fibronectin type III and SPF	19p13.3	79187	1.13	1.81	0.68	3.88	1.30E-04	1.98E-03	up CINSARC High vs. Low
CNGA2	cyclic nucleotide gated cha	Xq28	1260	0.48	1.28	0.80	3.87	1.36E-04	2.04E-03	up CINSARC High vs. Low
PARM1	prostate androgen-regulate	4q13.3	25849	8.29	8.94	0.65	3.86	1.40E-04	2.08E-03	up CINSARC High vs. Low
STYK1	serine/threonine/tyrosine k	12p13.2	55359	5.24	6.09	0.85	3.85	1.47E-04	2.17E-03	up CINSARC High vs. Low
SCN8A	sodium voltage-gated char	12q13.13	6334	4.10	4.92	0.82	3.84	1.49E-04	2.18E-03	up CINSARC High vs. Low
TEX19	testis expressed 19	17q25.3	400629	2.22	3.10	0.88	3.84	1.50E-04	2.19E-03	up CINSARC High vs. Low
GPC2	glypican 2	7q22.1	221914	3.62	4.28	0.66	3.80	1.78E-04	2.53E-03	up CINSARC High vs. Low
EDARADD	EDAR associated death do	1q42.3-q43	128178	7.59	8.47	0.87	3.78	1.91E-04	2.67E-03	up CINSARC High vs. Low
AREG	amphiregulin	4q13.3	374	6.34	7.58	1.24	3.77	1.99E-04	2.75E-03	up CINSARC High vs. Low
SCML2	Scm polycomb group prote	Xp22.13	10389	5.22	5.92	0.70	3.76	2.05E-04	2.79E-03	up CINSARC High vs. Low
MAGEA4	MAGE family member A4	Xq28	4103	0.15	0.74	0.59	3.72	2.38E-04	3.12E-03	up CINSARC High vs. Low
ONECUT2	one cut homeobox 2	18q21.31	9480	3.06	4.35	1.29	3.71	2.44E-04	3.15E-03	up CINSARC High vs. Low
CYP2J2	cytochrome P450 family 2	1p32.1	1573	6.66	7.39	0.73	3.69	2.62E-04	3.33E-03	up CINSARC High vs. Low
SLC4A8	solute carrier family 4 mem	12q13.13	9498	8.33	9.06	0.74	3.69	2.64E-04	3.34E-03	up CINSARC High vs. Low
KLHL13	kelch like family member 13	Xq24	90293	5.18	5.86	0.68	3.69	2.68E-04	3.39E-03	up CINSARC High vs. Low
RAB39B	RAB39B, member RAS on	Xq28	116442	4.92	5.87	0.95	3.67	2.87E-04	3.53E-03	up CINSARC High vs. Low
SLC15A2	solute carrier family 15 me	3q13.33	6565	4.63	5.33	0.70	3.60	3.69E-04	4.20E-03	up CINSARC High vs. Low
HNF4G	hepatocyte nuclear factor 4	8q21.13	3174	4.20	5.07	0.87	3.60	3.71E-04	4.21E-03	up CINSARC High vs. Low
GAREM1	GRB2 associated regulator	18q12.1	64762	5.83	6.47	0.65	3.59	3.89E-04	4.34E-03	up CINSARC High vs. Low
DMRTA1	DMRT like family A1	9p21.3	63951	1.60	2.36	0.76	3.59	3.91E-04	4.35E-03	up CINSARC High vs. Low
MMP1	matrix metallopeptidase 1	11q22.2	4312	5.86	7.00	1.14	3.57	4.10E-04	4.50E-03	up CINSARC High vs. Low
DLL3	delta like canonical Notch l	19q13.2	10683	1.27	2.13	0.87	3.57	4.19E-04	4.56E-03	up CINSARC High vs. Low
TNNT2	troponin T2, cardiac type	1q32.1	7139	0.88	1.52	0.64	3.56	4.25E-04	4.61E-03	up CINSARC High vs. Low
FGF13	fibroblast growth factor 13	Xq26.3-q27.1	2258	6.49	7.24	0.76	3.55	4.47E-04	4.76E-03	up CINSARC High vs. Low
LRRIQ1	leucine rich repeats and IQ	12q21.31	84125	3.02	3.94	0.93	3.55	4.52E-04	4.79E-03	up CINSARC High vs. Low
HSPA4L	heat shock protein family A	4q28.1	22824	6.46	7.13	0.67	3.55	4.53E-04	4.79E-03	up CINSARC High vs. Low
MAGEA1	MAGE family member A1	Xq28	4100	1.22	2.45	1.23	3.54	4.72E-04	4.95E-03	up CINSARC High vs. Low
RHBG	Rh family B glycoprotein (g	1q22	57127	3.17	3.93	0.75	3.53	4.79E-04	4.99E-03	up CINSARC High vs. Low
CARMIL3	capping protein regulator a	14q11.2	90668	3.52	4.24	0.73	3.53	4.79E-04	4.99E-03	up CINSARC High vs. Low
TTPA	alpha tocopherol transfer p	8q12.3	7274	1.51	2.22	0.71	3.52	4.91E-04	5.08E-03	up CINSARC High vs. Low
TSPAN8	tetraspanin 8	12q21.1	7103	1.46	2.66	1.19	3.52	4.94E-04	5.09E-03	up CINSARC High vs. Low
EPHA7	EPH receptor A7	6q16.1	2045	2.89	3.71	0.82	3.52	5.01E-04	5.14E-03	up CINSARC High vs. Low
SAMD11	sterile alpha motif domain c	1p36.33	148398	4.87	5.86	0.99	3.51	5.16E-04	5.28E-03	up CINSARC High vs. Low
SEPT3	septin 3	22q13.2	55964	7.44	8.09	0.65	3.49	5.61E-04	5.60E-03	up CINSARC High vs. Low
SLITRK5	SLIT and NTRK like family	13q31.2	26050	2.46	3.38	0.92	3.49	5.63E-04	5.61E-03	up CINSARC High vs. Low
CRYBG2	crystallin beta-gamma dom	1p36.11	55057	3.29	4.00	0.71	3.48	5.67E-04	5.62E-03	up CINSARC High vs. Low
WASF3	WASP family member 3	13q12.13	10810	6.18	6.77	0.59	3.47	5.99E-04	5.85E-03	up CINSARC High vs. Low
RFX6	regulatory factor X6	6q22.1	222546	0.64	1.31	0.67	3.46	6.09E-04	5.93E-03	up CINSARC High vs. Low
SLCO4C1	solute carrier organic anior	5q21.1	353189	2.70	3.36	0.66	3.44	6.61E-04	6.31E-03	up CINSARC High vs. Low
TMEM40	transmembrane protein 40	3p25.2	55287	2.40	3.36	0.97	3.43	6.82E-04	6.47E-03	up CINSARC High vs. Low
CCL18	C-C motif chemokine ligan	17q12	6362	4.26	5.15	0.89	3.41	7.29E-04	6.81E-03	up CINSARC High vs. Low
PIK3C2G	phosphatidylinositol-4-phos	12p12.3	5288	2.24	2.99	0.76	3.41	7.37E-04	6.86E-03	up CINSARC High vs. Low
IL19	interleukin 19	1q32.1	29949	1.59	2.57	0.98	3.40	7.59E-04	6.99E-03	up CINSARC High vs. Low
ZYG11A	zyg-11 family member A, c	1p32.3	440590	5.79	6.46	0.67	3.40	7.77E-04	7.11E-03	up CINSARC High vs. Low
DLX4	distal-less homeobox 4	17q21.33	1748	3.60	4.26	0.66	3.36	8.75E-04	7.78E-03	up CINSARC High vs. Low
PSCA	prostate stem cell antigen	8q24.3	8000	4.25	5.47	1.22	3.35	9.10E-04	8.00E-03	up CINSARC High vs. Low
TSPAN5	tetraspanin 5	4q23	10098	6.01	6.68	0.67	3.30	1.08E-03	9.10E-03	up CINSARC High vs. Low
TENM2	teneurin transmembrane p	5q34	57451	4.43	5.18	0.75	3.29	1.12E-03	9.28E-03	up CINSARC High vs. Low
ULBP1	UL16 binding protein 1	6q25.1	80329	3.07	3.75	0.68	3.29	1.12E-03	9.28E-03	up CINSARC High vs. Low
HS6ST3	heparan sulfate 6-O-sulfotr	13q32.1	266722	6.10	7.29	1.18	3.27	1.20E-03	9.72E-03	up CINSARC High vs. Low
GABRA3	gamma-aminobutyric acid t	Xq28	2556	0.50	1.23	0.72	3.24	1.32E-03	1.04E-02	up CINSARC High vs. Low
ADGRG6	adhesion G protein-couple	6q24.2	57211	6.21	7.01	0.80	3.24	1.34E-03	1.05E-02	up CINSARC High vs. Low
EYA2	EYA transcriptional coactiv	20q13.12	2139	6.99	7.79	0.80	3.24	1.35E-03	1.06E-02	up CINSARC High vs. Low
ALG1L	ALG1 chitobiosyldiphospho	3q21.2	200810	3.72	4.60	0.88	3.23	1.36E-03	1.06E-02	up CINSARC High vs. Low
FIGN	fidgetin, microtubule severi	2q24.3	55137	3.49	4.13	0.64	3.23	1.38E-03	1.07E-02	up CINSARC High vs. Low
A2ML1	alpha-2-macroglobulin like	12p13.31	144568	1.43	2.15	0.72	3.20	1.51E-03	1.14E-02	up CINSARC High vs. Low
PLCB4	phospholipase C beta 4	20p12.3-p12.2	5332	6.67	7.44	0.77	3.15	1.80E-03	1.29E-02	up CINSARC High vs. Low
PDE3B	phosphodiesterase 3B	11p15.2	5140	5.47	6.10	0.62	3.15	1.83E-03	1.31E-02	up CINSARC High vs. Low

Symbol	description	Cytoband	Entrez Gene ID	Low-risk (mean, log2)	High-risk (mean, log2)	High vs.Low (log2-ratio)	t	p-value	q-value	Associated to
MUC13	mucin 13, cell surface asso	3q21.2	56667	0.95	1.67	0.72	3.14	1.84E-03	1.32E-02	up CINSARC High vs. Low
IYD	iodotyrosine deiodinase	6q25.1	389434	3.41	4.32	0.92	3.13	1.90E-03	1.34E-02	up CINSARC High vs. Low
EFS	embryonal Fyn-associated	14q11.2	10278	8.13	8.79	0.66	3.13	1.95E-03	1.36E-02	up CINSARC High vs. Low
GAGE12D	G antigen 12D	Xp11.23	100132399	0.11	0.84	0.72	3.13	1.95E-03	1.36E-02	up CINSARC High vs. Low
MMP3	matrix metallopeptidase 3	11q22.2	4314	5.65	6.45	0.80	3.11	2.06E-03	1.42E-02	up CINSARC High vs. Low
DLX3	distal-less homeobox 3	17q21.33	1747	4.79	5.60	0.81	3.11	2.07E-03	1.42E-02	up CINSARC High vs. Low
EFNB3	ephrin B3	17p13.1	1949	5.54	6.17	0.63	3.08	2.25E-03	1.50E-02	up CINSARC High vs. Low
SFTPA2	surfactant protein A2	10q22.3	729238	3.11	3.92	0.80	3.06	2.41E-03	1.57E-02	up CINSARC High vs. Low
MSI1	musashi RNA binding prote	12q24.31	4440	4.92	5.82	0.90	3.06	2.43E-03	1.58E-02	up CINSARC High vs. Low
ARX	aristaless related homeobo	Xp21.3	170302	1.87	2.80	0.93	3.05	2.49E-03	1.60E-02	up CINSARC High vs. Low
L1CAM	L1 cell adhesion molecule	Xq28	3897	3.85	4.68	0.84	3.05	2.50E-03	1.61E-02	up CINSARC High vs. Low
NAP1L2	nucleosome assembly prot	Xq13.2	4674	4.09	4.76	0.67	3.04	2.56E-03	1.62E-02	up CINSARC High vs. Low
PADI3	peptidyl arginine deiminase	1p36.13	51702	1.78	2.53	0.75	3.00	2.96E-03	1.79E-02	up CINSARC High vs. Low
LHX1	LIM homeobox 1	17q12	3975	0.58	1.21	0.63	2.98	3.11E-03	1.84E-02	up CINSARC High vs. Low
TMSB15A	thymosin beta 15a	Xq22.1	11013	3.49	4.09	0.60	2.97	3.27E-03	1.90E-02	up CINSARC High vs. Low
SLC9A2	solute carrier family 9 mem	2q12.1	6549	4.04	5.20	1.17	2.95	3.43E-03	1.97E-02	up CINSARC High vs. Low
ST6GALNAC1	ST6 N-acetylgalactosamini	17q25.1	55808	2.67	3.33	0.66	2.94	3.55E-03	2.02E-02	up CINSARC High vs. Low
SLAIN1	SLAIN motif family membe	13q22.3	122060	3.75	4.46	0.71	2.94	3.58E-03	2.03E-02	up CINSARC High vs. Low
GPR158	G protein-coupled receptor	10p12.1	57512	3.25	4.05	0.79	2.94	3.58E-03	2.03E-02	up CINSARC High vs. Low
ADCY5	adenylate cyclase 5	3q21.1	111	5.22	6.15	0.93	2.93	3.60E-03	2.03E-02	up CINSARC High vs. Low
NXPH1	neurexophilin 1	7p21.3	30010	1.94	3.02	1.08	2.93	3.70E-03	2.08E-02	up CINSARC High vs. Low
ATP1A3	ATPase Na+/K+ transportin	19q13.2	478	4.42	5.30	0.88	2.92	3.75E-03	2.10E-02	up CINSARC High vs. Low
TMPRSS4	transmembrane serine pro	11q23.3	56649	4.30	5.34	1.04	2.90	3.97E-03	2.17E-02	up CINSARC High vs. Low
SLC27A6	solute carrier family 27 me	5q23.3	28965	2.56	3.24	0.68	2.89	4.14E-03	2.23E-02	up CINSARC High vs. Low
S100A8	S100 calcium binding prote	1q21.3	6279	4.64	5.36	0.72	2.89	4.16E-03	2.24E-02	up CINSARC High vs. Low
GAGE4	G antigen 4	Xp11.4-p11.2	2576	0.15	0.82	0.66	2.89	4.16E-03	2.24E-02	up CINSARC High vs. Low
SLC6A15	solute carrier family 6 mem	12q21.31	55117	0.80	1.39	0.59	2.88	4.31E-03	2.28E-02	up CINSARC High vs. Low
PLCH1	phospholipase C eta 1	3q25.31	23007	5.00	5.92	0.92	2.86	4.47E-03	2.34E-02	up CINSARC High vs. Low
OGDHL	oxoglutarate dehydrogena	10q11.23	55753	1.51	2.23	0.71	2.86	4.49E-03	2.34E-02	up CINSARC High vs. Low
VSTM2L	V-set and transmembrane	20q11.23	128434	3.88	4.66	0.78	2.85	4.68E-03	2.41E-02	up CINSARC High vs. Low
GALNT3	polypeptide N-acetylgalact	2q24.3	2591	7.96	8.69	0.73	2.85	4.69E-03	2.42E-02	up CINSARC High vs. Low
SERPINB5	serpin family B member 5	18q21.33	5268	4.28	5.11	0.82	2.84	4.89E-03	2.48E-02	up CINSARC High vs. Low
TTLL7	tubulin tyrosine ligase like	1p31.1	79739	5.34	5.98	0.64	2.83	4.93E-03	2.49E-02	up CINSARC High vs. Low
FAM178B	family with sequence simila	2q11.2	51252	3.42	4.15	0.73	2.82	5.10E-03	2.55E-02	up CINSARC High vs. Low
ZBED9	zinc finger BED-type conta	6p22.1	114821	3.29	4.14	0.86	2.82	5.12E-03	2.55E-02	up CINSARC High vs. Low
IL6	interleukin 6	7p15.3	3569	2.94	3.53	0.59	2.82	5.16E-03	2.56E-02	up CINSARC High vs. Low
PCDH11X	protocadherin 11 X-linked	Xq21.31	27328	0.90	1.49	0.59	2.82	5.18E-03	2.57E-02	up CINSARC High vs. Low
SPRR3	small proline rich protein 3	1q21.3	6707	0.56	1.21	0.65	2.81	5.31E-03	2.61E-02	up CINSARC High vs. Low
ALDH3B2	aldehyde dehydrogenase 3	11q13.2	222	9.67	10.39	0.72	2.81	5.36E-03	2.63E-02	up CINSARC High vs. Low
CBLN1	cerebellin 1 precursor	16q12.1	869	2.31	2.98	0.67	2.80	5.37E-03	2.63E-02	up CINSARC High vs. Low
POF1B	POF1B actin binding prote	Xq21.1	79983	4.51	5.50	0.99	2.80	5.40E-03	2.64E-02	up CINSARC High vs. Low
FERMT1	fermitin family member 1	20p12.3	55612	2.81	3.43	0.62	2.77	5.88E-03	2.81E-02	up CINSARC High vs. Low
FCRL3	Fc receptor like 3	1q23.1	115352	2.39	2.99	0.60	2.75	6.37E-03	2.97E-02	up CINSARC High vs. Low
AOX1	aldehyde oxidase 1	2q33.1	316	6.08	6.67	0.59	2.74	6.44E-03	3.00E-02	up CINSARC High vs. Low
NMNAT2	nicotinamide nucleotide ad	1q25.3	23057	4.46	5.13	0.67	2.74	6.48E-03	3.01E-02	up CINSARC High vs. Low
KIAA0319	KIAA0319	6p22.3	9856	3.63	4.36	0.74	2.73	6.63E-03	3.05E-02	up CINSARC High vs. Low
RSPO4	R-spondin 4	20p13	343637	1.89	2.49	0.59	2.73	6.70E-03	3.07E-02	up CINSARC High vs. Low
FRAS1	Fraser extracellular matrix	4q21.21	80144	5.64	6.28	0.63	2.73	6.70E-03	3.07E-02	up CINSARC High vs. Low
TMEM108	transmembrane protein 10	3q22.1	66000	4.34	4.94	0.60	2.73	6.71E-03	3.07E-02	up CINSARC High vs. Low
ACTN2	actinin alpha 2	1q43	88	1.90	2.68	0.78	2.70	7.23E-03	3.25E-02	up CINSARC High vs. Low
ELFN2	extracellular leucine rich re	22q13.1	114794	2.88	3.64	0.76	2.70	7.30E-03	3.27E-02	up CINSARC High vs. Low
AOC1	amine oxidase copper con	7q36.1	26	3.06	3.80	0.74	2.70	7.42E-03	3.31E-02	up CINSARC High vs. Low
GJB3	gap junction protein beta 3	1p34.3	2707	2.29	2.88	0.59	2.68	7.71E-03	3.40E-02	up CINSARC High vs. Low
CYP4F3	cytochrome P450 family 4	19p13.12	4051	2.02	2.68	0.66	2.68	7.72E-03	3.40E-02	up CINSARC High vs. Low
GAGE2D	G antigen 2D	Xp11.23	729408	0.19	0.81	0.63	2.68	7.75E-03	3.41E-02	up CINSARC High vs. Low
FDCSP	follicular dendritic cell secr	4q13.3|4q13	260436	1.65	2.41	0.76	2.66	8.22E-03	3.54E-02	up CINSARC High vs. Low
EREG	epiregulin	4q13.3	2069	1.53	2.17	0.64	2.66	8.35E-03	3.58E-02	up CINSARC High vs. Low
PLAC4	placenta enriched 4	21q22.2	191585	1.86	2.53	0.66	2.65	8.37E-03	3.58E-02	up CINSARC High vs. Low
PALM3	paralemmin 3	19p13.12	342979	4.22	4.83	0.61	2.65	8.57E-03	3.64E-02	up CINSARC High vs. Low
DSC3	desmocollin 3	18q12.1	1825	4.19	4.93	0.75	2.63	8.89E-03	3.73E-02	up CINSARC High vs. Low
HOXC10	homeobox C10	12q13.13	3226	7.48	8.47	0.99	2.63	8.96E-03	3.75E-02	up CINSARC High vs. Low
FOLR1	folate receptor 1	11q13.4	2348	2.63	3.43	0.79	2.63	8.99E-03	3.76E-02	up CINSARC High vs. Low
MGAT5B	alpha-1,6-mannosylglycopr	17q25.2	146664	3.27	3.99	0.72	2.60	9.78E-03	3.99E-02	up CINSARC High vs. Low
COCH	cochlin	14q12	1690	4.07	4.86	0.79	2.60	9.83E-03	4.00E-02	up CINSARC High vs. Low
SLC5A12	solute carrier family 5 mem	11p14.2	159963	1.05	1.70	0.64	2.59	1.00E-02	4.05E-02	up CINSARC High vs. Low
BAMBI	BMP and activin membran	10p12.1	25805	8.85	9.53	0.69	2.58	1.05E-02	4.18E-02	up CINSARC High vs. Low
RIMS1	regulating synaptic membra	6q13	22999	4.09	4.85	0.76	2.57	1.06E-02	4.20E-02	up CINSARC High vs. Low
SLC6A11	solute carrier family 6 mem	3p25.3	6538	1.53	2.24	0.71	2.57	1.08E-02	4.25E-02	up CINSARC High vs. Low
BEND7	BEN domain containing 7	10p13	222389	5.99	6.58	0.59	2.57	1.08E-02	4.26E-02	up CINSARC High vs. Low
SIX1	SIX homeobox 1	14q23.1	6495	7.71	8.46	0.75	2.53	1.18E-02	4.50E-02	up CINSARC High vs. Low
AQP9	aquaporin 9	15q21.3	366	4.53	5.14	0.61	2.52	1.21E-02	4.59E-02	up CINSARC High vs. Low
ATP6V0A4	ATPase H+ transporting V0	7q34	50617	3.42	4.29	0.87	2.52	1.22E-02	4.59E-02	up CINSARC High vs. Low
GABRP	gamma-aminobutyric acid t	5q35.1	2568	4.76	5.55	0.79	2.52	1.23E-02	4.63E-02	up CINSARC High vs. Low
LY6K	lymphocyte antigen 6 famil	8q24.3	54742	4.41	5.13	0.72	2.50	1.28E-02	4.73E-02	up CINSARC High vs. Low
TRPA1	transient receptor potential	8q21.11	8989	3.94	4.98	1.04	2.50	1.31E-02	4.79E-02	up CINSARC High vs. Low
JCHAIN	joining chain of multimeric	4q13.3	3512	8.28	9.05	0.77	2.50	1.31E-02	4.80E-02	up CINSARC High vs. Low
PTPRZ1	protein tyrosine phosphata	7q31.32	5803	1.70	2.31	0.61	2.49	1.31E-02	4.81E-02	up CINSARC High vs. Low
MAGEA6	MAGE family member A6	Xq28	4105	0.96	1.85	0.90	2.49	1.33E-02	4.84E-02	up CINSARC High vs. Low
PRSS1	serine protease 1	7q34	5644	1.02	1.76	0.74	2.49	1.34E-02	4.86E-02	up CINSARC High vs. Low
ZNF492	zinc finger protein 492	19p12	57615	2.40	3.00	0.59	2.49	1.34E-02	4.86E-02	up CINSARC High vs. Low
TSPYL5	TSPY like 5	8q22.1	85453	8.32	8.92	0.60	2.47	1.40E-02	0.050	up CINSARC High vs. Low
VSTM2A	V-set and transmembrane	7p11.2	222008	3.46	4.74	1.29	2.47	1.41E-02	0.050	up CINSARC High vs. Low
PKIA	cAMP-dependent protein k	8q21.13	5569	4.37	4.97	0.60	2.45	1.49E-02	0.052	up CINSARC High vs. Low
SLC6A14	solute carrier family 6 mem	Xq23	11254	1.98	2.57	0.59	2.45	1.49E-02	0.052	up CINSARC High vs. Low
MAGEA3	MAGE family member A3	Xq28	4102	0.93	1.79	0.86	2.45	1.50E-02	0.053	up CINSARC High vs. Low
CDK5R2	cyclin dependent kinase 5	2q35	8941	0.90	1.51	0.61	2.43	1.59E-02	0.055	up CINSARC High vs. Low
PAX2	paired box 2	10q24.31	5076	3.49	4.22	0.74	2.42	1.63E-02	0.055	up CINSARC High vs. Low
TRIM36	tripartite motif containing 3	5q22.3	55521	6.91	7.53	0.62	2.41	1.63E-02	0.056	up CINSARC High vs. Low
SPTSSB	serine palmitoyltransferase	3q26.1	165679	7.03	7.82	0.79	2.41	1.67E-02	0.057	up CINSARC High vs. Low
CACNG6	calcium voltage-gated chan	19q13.42	59285	1.64	2.47	0.82	2.40	1.68E-02	0.057	up CINSARC High vs. Low
MKRN3	makorin ring finger protein	15q11.2	7681	2.02	2.71	0.69	2.39	1.73E-02	0.058	up CINSARC High vs. Low
GLYATL3	glycine-N-acyltransferase l	6p12.3	389396	0.52	1.11	0.59	2.38	1.79E-02	0.059	up CINSARC High vs. Low
CELF3	CUGBP Elav-like family me	1q21.3	11189	1.79	2.44	0.66	2.38	1.79E-02	0.059	up CINSARC High vs. Low
DQX1	DEAQ-box RNA dependen	2p13.1	165545	4.08	4.72	0.63	2.37	1.83E-02	0.060	up CINSARC High vs. Low
LRAT	lecithin retinol acyltransfera	4q32.1	9227	3.08	3.76	0.68	2.36	1.91E-02	0.061	up CINSARC High vs. Low
DPYSL5	dihydropyrimidinase like 5	2p23.3	56896	1.03	1.66	0.63	2.35	1.93E-02	0.062	up CINSARC High vs. Low
ASIC2	acid sensing ion channel s	17q11.2-q12	40	2.14	2.88	0.74	2.35	1.94E-02	0.062	up CINSARC High vs. Low
RASAL1	RAS protein activator like 1	12q24.13	8437	2.83	3.43	0.60	2.34	1.97E-02	0.063	up CINSARC High vs. Low
KCNK3	potassium two pore domai	2p23.3	3777	3.94	4.69	0.75	2.34	2.00E-02	0.063	up CINSARC High vs. Low
BRINP3	BMP/retinoic acid inducible	1q31.1	339479	0.97	1.80	0.83	2.33	2.04E-02	0.064	up CINSARC High vs. Low
PCSK2	proprotein convertase subt	20p12.1	5126	1.18	1.95	0.77	2.32	2.08E-02	0.065	up CINSARC High vs. Low
SERPINA5	serpin family A member 5	14q32.13	5104	9.36	10.13	0.76	2.31	2.14E-02	0.066	up CINSARC High vs. Low
IDO1	indoleamine 2,3-dioxygena	8p11.21	3620	5.28	5.87	0.59	2.31	2.16E-02	0.066	up CINSARC High vs. Low
BRSK2	BR serine/threonine kinase	11p15.5	9024	3.01	3.69	0.69	2.29	2.27E-02	0.068	up CINSARC High vs. Low
ADAMDEC1	ADAM like decysin 1	8p21.2	27299	5.54	6.14	0.59	2.27	2.39E-02	0.071	up CINSARC High vs. Low

Symbol	description	Cytoband	Entrez Gene ID	Low-risk (mean, log2)	High-risk (mean, log2)	High vs.Low (log2-ratio)	t	p-value	q-value	Associated to
TMEM150C	transmembrane protein 15	4q21.22	441027	8.09	8.83	0.74	2.27	2.42E-02	0.071	up CINSARC High vs. Low
B3GNT3	UDP-GlcNAc:betaGal beta	19p13.11	10331	3.46	4.06	0.60	2.26	2.48E-02	0.072	up CINSARC High vs. Low
DSG3	desmoglein 3	18q12.1	1830	3.18	3.86	0.68	2.25	2.51E-02	0.073	up CINSARC High vs. Low
CXCL9	C-X-C motif chemokine liga	4q21.1	4283	8.58	9.20	0.62	2.24	2.60E-02	0.074	up CINSARC High vs. Low
HOXC11	homeobox C11	12q13.13	3227	4.81	5.62	0.81	2.19	2.90E-02	0.080	up CINSARC High vs. Low
CCL21	C-C motif chemokine ligan	9p13.3	6366	4.47	5.12	0.65	2.19	2.92E-02	0.080	up CINSARC High vs. Low
MAGEA12	MAGE family member A12	Xq28	4111	0.69	1.30	0.61	2.16	3.13E-02	0.083	up CINSARC High vs. Low
BPIFB1	BPI fold containing family E	20q11.21	92747	6.73	7.42	0.69	2.16	3.14E-02	0.084	up CINSARC High vs. Low
SLC4A10	solute carrier family 4 mem	2q24.2	57282	2.28	3.01	0.72	2.15	3.20E-02	0.085	up CINSARC High vs. Low
LEP	leptin	7q32.1	3952	2.67	3.31	0.64	2.15	3.26E-02	0.086	up CINSARC High vs. Low
ADGRV1	adhesion G protein-couple	5q14.3	84059	7.31	8.00	0.69	2.13	3.43E-02	0.088	up CINSARC High vs. Low
ZIC2	Zic family member 2	13q32.3	7546	3.15	3.87	0.72	2.12	3.48E-02	0.089	up CINSARC High vs. Low
PLA2G2D	phospholipase A2 group III	1p36.12	26279	3.45	4.06	0.61	2.11	3.60E-02	0.091	up CINSARC High vs. Low
MUCL1	mucin like 1	12q13.2	118430	5.19	6.03	0.84	2.10	3.66E-02	0.092	up CINSARC High vs. Low
KRT6B	keratin 6B	12q13.13	3854	3.97	4.70	0.73	2.08	3.79E-02	0.094	up CINSARC High vs. Low
ALOX15	arachidonate 15-lipoxygen	17p13.2	246	3.70	4.32	0.62	2.06	4.05E-02	0.098	up CINSARC High vs. Low
OLFM4	olfactomedin 4	13q14.3	10562	2.74	3.47	0.73	2.05	4.12E-02	0.099	up CINSARC High vs. Low
GRPR	gastrin releasing peptide re	Xp22.2	2925	7.32	6.62	-0.70	-2.04	4.20E-02	0.100	down CINSARC High vs. Low
SLC7A2	solute carrier family 7 mem	8p22	6542	11.97	11.32	-0.65	-2.06	4.04E-02	0.097	down CINSARC High vs. Low
CST9	cystatin 9	20p11.21	128822	5.59	4.69	-0.90	-2.06	4.02E-02	0.097	down CINSARC High vs. Low
INSYN2A	inhibitory synaptic factor 2A	10q26.2	642938	6.62	5.87	-0.75	-2.07	3.95E-02	0.096	down CINSARC High vs. Low
RGS22	regulator of G protein signa	8q22.2	26166	6.53	5.92	-0.60	-2.07	3.91E-02	0.095	down CINSARC High vs. Low
FOXI1	forkhead box I1	5q35.1	2299	5.47	4.60	-0.86	-2.09	3.73E-02	0.093	down CINSARC High vs. Low
ANKRD30A	ankyrin repeat domain 30A	10p11.21	91074	8.34	7.36	-0.98	-2.09	3.71E-02	0.092	down CINSARC High vs. Low
TNNI3	troponin I3, cardiac type	19q13.42	7137	3.14	2.48	-0.66	-2.10	3.63E-02	0.091	down CINSARC High vs. Low
CITED1	Cbp/p300 interacting trans	Xq13.1	4435	4.72	4.10	-0.61	-2.13	3.38E-02	0.087	down CINSARC High vs. Low
PDZK1IP1	PDZK1 interacting protein	1p33	10158	7.23	6.52	-0.71	-2.14	3.32E-02	0.086	down CINSARC High vs. Low
KRT15	keratin 15	17q21.2	3866	8.56	7.89	-0.67	-2.17	3.05E-02	0.082	down CINSARC High vs. Low
SYT13	synaptotagmin 13	11p11.2	57586	9.36	8.38	-0.98	-2.20	2.85E-02	0.079	down CINSARC High vs. Low
WFDC2	WAP four-disulfide core do	20q13.12	10406	9.53	8.69	-0.84	-2.22	2.72E-02	0.076	down CINSARC High vs. Low
TMEM145	transmembrane protein 14	19q13.2	284339	5.73	5.13	-0.60	-2.23	2.67E-02	0.076	down CINSARC High vs. Low
ORM1	orosomucoid 1	9q32	5004	3.43	2.67	-0.76	-2.23	2.64E-02	0.075	down CINSARC High vs. Low
AGTR1	angiotensin II receptor type	3q24	185	7.59	6.75	-0.84	-2.23	2.63E-02	0.075	down CINSARC High vs. Low
MUC1	mucin 1, cell surface assoc	1q22	4582	12.88	12.29	-0.59	-2.26	2.43E-02	0.071	down CINSARC High vs. Low
IGSF21	immunoglobin superfamily	1p36.13	84966	6.59	5.97	-0.63	-2.27	2.39E-02	0.071	down CINSARC High vs. Low
CCNO	cyclin O	5q11.2	10309	6.65	5.85	-0.80	-2.28	2.35E-02	0.070	down CINSARC High vs. Low
ORM2	orosomucoid 2	9q32	5005	2.41	1.76	-0.65	-2.28	2.32E-02	0.069	down CINSARC High vs. Low
SP5	Sp5 transcription factor	2q31.1	389058	3.73	3.10	-0.63	-2.30	2.23E-02	0.067	down CINSARC High vs. Low
DLX2	distal-less homeobox 2	2q31.1	1746	3.32	2.63	-0.70	-2.30	2.21E-02	0.067	down CINSARC High vs. Low
SEZ6L	seizure related 6 homolog	22q12.1	23544	4.29	3.44	-0.85	-2.32	2.13E-02	0.066	down CINSARC High vs. Low
COL4A6	collagen type IV alpha 6 ch	Xq22.3	1288	5.23	4.56	-0.66	-2.32	2.11E-02	0.065	down CINSARC High vs. Low
CCDC196	coiled-coil domain containi	14q23.3	440184	2.47	1.85	-0.62	-2.33	2.07E-02	0.065	down CINSARC High vs. Low
KCNE4	potassium voltage-gated c	2q36.1	23704	9.70	9.05	-0.65	-2.34	1.98E-02	0.063	down CINSARC High vs. Low
NDP	norrin cystine knot growth f	Xp11.3	4693	4.74	3.90	-0.84	-2.34	1.97E-02	0.063	down CINSARC High vs. Low
SLC7A4	solute carrier family 7 mem	22q11.21	6545	4.24	3.34	-0.90	-2.35	1.93E-02	0.062	down CINSARC High vs. Low
DOK7	docking protein 7	4p16.3	285489	7.27	6.65	-0.61	-2.36	1.87E-02	0.061	down CINSARC High vs. Low
ATP1A4	ATPase Na+/K+ transporti	1q23.2	480	4.27	3.68	-0.60	-2.38	1.78E-02	0.059	down CINSARC High vs. Low
PHGR1	proline, histidine and glycin	15q15.1	644844	3.44	2.68	-0.75	-2.40	1.71E-02	0.058	down CINSARC High vs. Low
CYP2A7	cytochrome P450 family 2	19q13.2	1549	3.07	2.16	-0.90	-2.40	1.71E-02	0.058	down CINSARC High vs. Low
MYO3B	myosin IIIB	2q31.1	140469	3.21	2.49	-0.72	-2.44	1.54E-02	0.054	down CINSARC High vs. Low
GRIA2	glutamate ionotropic recep	4q32.1	2891	5.40	4.21	-1.20	-2.45	1.47E-02	0.052	down CINSARC High vs. Low
CARTPT	CART prepropeptide	5q13.2	9607	2.21	1.20	-1.01	-2.46	1.44E-02	0.051	down CINSARC High vs. Low
RHBDL3	rhomboid like 3	17q11.2	162494	4.57	3.93	-0.64	-2.48	1.37E-02	4.94E-02	down CINSARC High vs. Low
TPSG1	tryptase gamma 1	16p13.3	25823	4.84	4.09	-0.75	-2.50	1.31E-02	4.80E-02	down CINSARC High vs. Low
C19orf33	chromosome 19 open read	19q13.2	64073	8.17	7.44	-0.73	-2.52	1.22E-02	4.60E-02	down CINSARC High vs. Low
MPPED1	metallophosphoesterase d	22q13.2	758	2.15	1.49	-0.66	-2.55	1.12E-02	4.37E-02	down CINSARC High vs. Low
CFAP221	cilia and flagella associated	2q14.2	200373	3.14	2.46	-0.67	-2.56	1.11E-02	4.33E-02	down CINSARC High vs. Low
CHRD	chordin	3q27.1	8646	8.51	7.92	-0.59	-2.57	1.06E-02	4.21E-02	down CINSARC High vs. Low
TUBA3D	tubulin alpha 3d	2q21.1	113457	5.81	4.98	-0.83	-2.60	9.72E-03	3.97E-02	down CINSARC High vs. Low
FGF10	fibroblast growth factor 10	5p12	2255	3.66	2.77	-0.89	-2.60	9.72E-03	3.97E-02	down CINSARC High vs. Low
ZNF385D	zinc finger protein 385D	3p24.3	79750	4.61	3.98	-0.63	-2.62	9.13E-03	3.80E-02	down CINSARC High vs. Low
GJA1	gap junction protein alpha	6q22.31	2697	12.20	11.58	-0.62	-2.68	7.79E-03	3.42E-02	down CINSARC High vs. Low
CDHR4	cadherin related family me	3p21.31	389118	2.12	1.47	-0.65	-2.68	7.69E-03	3.39E-02	down CINSARC High vs. Low
PPP1R36	protein phosphatase 1 regu	14q23.3	145376	4.44	3.84	-0.59	-2.69	7.59E-03	3.36E-02	down CINSARC High vs. Low
PHYHD1	phytanoyl-CoA dioxygenas	9q34.11	254295	7.70	7.00	-0.70	-2.70	7.35E-03	3.28E-02	down CINSARC High vs. Low
KLHL1	kelch like family member 1	13q21.33	57626	1.40	0.64	-0.76	-2.71	7.14E-03	3.22E-02	down CINSARC High vs. Low
CLIC6	chloride intracellular chann	21q22.12	54102	8.94	7.82	-1.12	-2.73	6.78E-03	3.10E-02	down CINSARC High vs. Low
TTC22	tetratricopeptide repeat do	1p32.3	55001	4.45	3.72	-0.73	-2.74	6.58E-03	3.04E-02	down CINSARC High vs. Low
CFB	complement factor B	6p21.33	629	12.82	12.02	-0.80	-2.74	6.52E-03	3.02E-02	down CINSARC High vs. Low
DMKN	dermokine	19q13.12	93099	7.49	6.80	-0.68	-2.75	6.31E-03	2.96E-02	down CINSARC High vs. Low
SEC14L5	SEC14 like lipid binding 5	16p13.3	9717	3.41	2.79	-0.62	-2.76	6.14E-03	2.90E-02	down CINSARC High vs. Low
NAT1	N-acetyltransferase 1	8p22	9	11.23	10.52	-0.71	-2.80	5.46E-03	2.65E-02	down CINSARC High vs. Low
STC2	stanniocalcin 2	5q35.2	8614	12.84	12.05	-0.79	-2.80	5.41E-03	2.64E-02	down CINSARC High vs. Low
C5orf38	chromosome 5 open readi	5p15.33	153571	6.08	5.41	-0.67	-2.80	5.41E-03	2.64E-02	down CINSARC High vs. Low
SKIDA1	SKI/DACH domain contain	10p12.31	387640	4.25	3.64	-0.61	-2.81	5.32E-03	2.62E-02	down CINSARC High vs. Low
SYT12	synaptotagmin 12	11q13.2	91683	8.95	8.32	-0.63	-2.82	5.16E-03	2.56E-02	down CINSARC High vs. Low
CDSN	corneodesmosin	6p21.33	1041	6.01	5.21	-0.80	-2.82	5.15E-03	2.56E-02	down CINSARC High vs. Low
CHI3L2	chitinase 3 like 2	1p13.2	1117	6.57	5.78	-0.79	-2.84	4.80E-03	2.45E-02	down CINSARC High vs. Low
GALNT5	polypeptide N-acetylgalact	2q24.1	11227	6.98	6.29	-0.69	-2.85	4.73E-03	2.43E-02	down CINSARC High vs. Low
CST5	cystatin D	20p11.21	1473	4.15	2.93	-1.22	-2.87	4.41E-03	2.32E-02	down CINSARC High vs. Low
CCDC65	coiled-coil domain containi	12q13.12	85478	4.25	3.62	-0.63	-2.88	4.28E-03	2.27E-02	down CINSARC High vs. Low
ENTPD8	ectonucleoside triphosphat	9q34.3	377841	5.90	5.22	-0.68	-2.88	4.27E-03	2.27E-02	down CINSARC High vs. Low
RANBP3L	RAN binding protein 3 like	5p13.2	202151	5.28	4.54	-0.74	-2.88	4.26E-03	2.27E-02	down CINSARC High vs. Low
P2RY11	purinergic receptor P2Y11	19p13.2	5032	6.26	5.45	-0.81	-2.90	4.04E-03	2.19E-02	down CINSARC High vs. Low
CHST8	carbohydrate sulfotransfera	19q13.11	64377	6.87	5.87	-1.00	-2.93	3.70E-03	2.08E-02	down CINSARC High vs. Low
PGR	progesterone receptor	11q22.1	5241	10.63	9.62	-1.01	-2.93	3.62E-03	2.05E-02	down CINSARC High vs. Low
ACSM1	acyl-CoA synthetase mediu	16p12.3	116285	5.00	4.41	-0.59	-2.98	3.16E-03	1.86E-02	down CINSARC High vs. Low
CA14	carbonic anhydrase 14	1q21.2	23632	4.40	3.80	-0.60	-2.99	3.01E-03	1.80E-02	down CINSARC High vs. Low
SPTBN4	spectrin beta, non-erythroc	19q13.2	57731	6.37	5.76	-0.61	-3.01	2.83E-03	1.73E-02	down CINSARC High vs. Low
RAB38	RAB38, member RAS onco	11q14.2	23682	7.07	6.29	-0.78	-3.01	2.79E-03	1.72E-02	down CINSARC High vs. Low
SERPINA6	serpin family A member 6	14q32.13	866	6.19	4.60	-1.59	-3.02	2.76E-03	1.70E-02	down CINSARC High vs. Low
SEC14L2	SEC14 like lipid binding 2	22q12.2	23541	10.14	9.45	-0.69	-3.04	2.54E-03	1.62E-02	down CINSARC High vs. Low
TDRD1	tudor domain containing 1	10q25.3	56165	2.84	1.94	-0.90	-3.10	2.14E-03	1.45E-02	down CINSARC High vs. Low
CLDN1	claudin 1	3q28	9076	7.64	7.04	-0.61	-3.10	2.13E-03	1.44E-02	down CINSARC High vs. Low
FBXO2	F-box protein 2	1p36.22	26232	7.70	6.97	-0.73	-3.10	2.12E-03	1.44E-02	down CINSARC High vs. Low
LACRT	lacritin	12q13.2	90070	1.10	0.36	-0.74	-3.10	2.11E-03	1.44E-02	down CINSARC High vs. Low
MPV17L	MPV17 mitochondrial inner	16p13.11	255027	7.87	7.15	-0.73	-3.13	1.92E-03	1.35E-02	down CINSARC High vs. Low
MYBPC1	myosin binding protein C, s	12q23.2	4604	3.84	2.61	-1.23	-3.17	1.67E-03	1.22E-02	down CINSARC High vs. Low
TMPRSS6	transmembrane serine pro	22q12.3	164656	7.10	6.17	-0.92	-3.18	1.60E-03	1.19E-02	down CINSARC High vs. Low
NEK10	NIMA related kinase 10	3p24.1	152110	6.88	5.96	-0.91	-3.21	1.46E-03	1.11E-02	down CINSARC High vs. Low
ZNF385B	zinc finger protein 385B	2q31.2-q31.3	151126	5.91	4.78	-1.13	-3.28	1.15E-03	9.46E-03	down CINSARC High vs. Low
REEP6	receptor accessory protein	19p13.3	92840	10.11	9.51	-0.60	-3.28	1.14E-03	9.40E-03	down CINSARC High vs. Low
S100A1	S100 calcium binding prote	1q21.3	6271	7.98	7.09	-0.89	-3.30	1.07E-03	9.05E-03	down CINSARC High vs. Low
GCM1	glial cells missing transcrip	6p12.1	8521	2.73	2.07	-0.66	-3.31	1.03E-03	8.82E-03	down CINSARC High vs. Low
FGD3	FYVE, RhoGEF and PH do	9q22.31	89846	9.87	9.23	-0.64	-3.34	9.55E-04	8.31E-03	down CINSARC High vs. Low

Symbol	description	Cytoband	Entrez Gene ID	Low-risk (mean, log2)	High-risk (mean, log2)	High vs.Low (log2-ratio)	t	p-value	q-value	Associated to
NXNL2	nucleoredoxin like 2	9q22.1	158046	4.63	3.92	-0.71	-3.35	8.98E-04	7.91E-03	down CINSARC High vs. Low
PIP	prolactin induced protein	7q34	5304	9.20	7.56	-1.63	-3.35	8.97E-04	7.91E-03	down CINSARC High vs. Low
ADCY1	adenylate cyclase 1	7p12.3	107	10.17	9.29	-0.88	-3.37	8.44E-04	7.55E-03	down CINSARC High vs. Low
GSTM2	glutathione S-transferase r	1p13.3	2946	9.37	8.60	-0.77	-3.41	7.44E-04	6.89E-03	down CINSARC High vs. Low
GAD1	glutamate decarboxylase 1	2q31.1	2571	3.32	2.31	-1.01	-3.44	6.68E-04	6.37E-03	down CINSARC High vs. Low
SEMA3B	semaphorin 3B	3p21.31	7869	9.17	8.45	-0.72	-3.45	6.43E-04	6.17E-03	down CINSARC High vs. Low
KCND3	potassium voltage-gated c	1p13.2	3752	5.85	4.94	-0.90	-3.49	5.65E-04	5.61E-03	down CINSARC High vs. Low
LINC00173	long intergenic non-protein	12q24.22	100287569	4.74	4.03	-0.71	-3.49	5.51E-04	5.54E-03	down CINSARC High vs. Low
JHY	junctional cadherin comple	11q24.1	79864	7.04	6.42	-0.62	-3.50	5.34E-04	5.41E-03	down CINSARC High vs. Low
RAMP3	receptor activity modifying	7p13	10268	8.85	8.09	-0.76	-3.53	4.85E-04	5.04E-03	down CINSARC High vs. Low
KRT23	keratin 23	17q21.2	25984	7.68	6.55	-1.13	-3.53	4.76E-04	4.98E-03	down CINSARC High vs. Low
PSORS1C1	psoriasis susceptibility 1 ca	6p21.33	170679	3.28	2.67	-0.61	-3.54	4.65E-04	4.90E-03	down CINSARC High vs. Low
TEKT3	tektin 3	17p12	64518	3.69	3.04	-0.65	-3.54	4.64E-04	4.89E-03	down CINSARC High vs. Low
PTPRT	protein tyrosine phosphata	20q12-q13.11	11122	9.55	8.29	-1.26	-3.59	3.88E-04	4.34E-03	down CINSARC High vs. Low
PPP4R4	protein phosphatase 4 regu	14q32.12-q32.13	57718	4.58	3.37	-1.22	-3.59	3.80E-04	4.27E-03	down CINSARC High vs. Low
CFAP57	cilia and flagella associated	1p34.2	149465	4.09	3.44	-0.65	-3.60	3.72E-04	4.22E-03	down CINSARC High vs. Low
EGOT	eosinophil granule ontoger	3p26.1	100126791	4.28	3.55	-0.73	-3.61	3.59E-04	4.13E-03	down CINSARC High vs. Low
FBXL16	F-box and leucine rich repe	16p13.3	146330	9.65	8.94	-0.71	-3.61	3.58E-04	4.12E-03	down CINSARC High vs. Low
CLU	clusterin	8p21.1	1191	13.35	12.74	-0.61	-3.62	3.45E-04	4.03E-03	down CINSARC High vs. Low
ADGRB2	adhesion G protein-couple	1p35.2	576	9.38	8.69	-0.69	-3.62	3.42E-04	4.00E-03	down CINSARC High vs. Low
LINC00160	long intergenic non-protein	21q22.12	54064	3.26	2.59	-0.67	-3.64	3.24E-04	3.87E-03	down CINSARC High vs. Low
PGGHG	protein-glucosylgalactosylh	11p15.5	80162	9.96	9.17	-0.78	-3.64	3.24E-04	3.87E-03	down CINSARC High vs. Low
CCDC153	coiled-coil domain containi	11q23.3	283152	4.48	3.77	-0.71	-3.64	3.17E-04	3.82E-03	down CINSARC High vs. Low
NTN4	netrin 4	12q22	59277	10.61	9.76	-0.85	-3.68	2.80E-04	3.47E-03	down CINSARC High vs. Low
ABAT	4-aminobutyrate aminotran	16p13.2	18	10.66	9.99	-0.67	-3.68	2.74E-04	3.41E-03	down CINSARC High vs. Low
PCP2	Purkinje cell protein 2	19p13.2	126006	5.73	5.04	-0.68	-3.72	2.40E-04	3.13E-03	down CINSARC High vs. Low
ABLIM3	actin binding LIM protein fa	5q32	22885	9.47	8.84	-0.64	-3.73	2.26E-04	3.00E-03	down CINSARC High vs. Low
GRIA1	glutamate ionotropic recep	5q33.2	2890	3.11	1.85	-1.25	-3.76	2.01E-04	2.76E-03	down CINSARC High vs. Low
LOC100128239	uncharacterized LOC1001	11q25	100128239	3.45	2.74	-0.70	-3.85	1.44E-04	2.14E-03	down CINSARC High vs. Low
TMEM63C	transmembrane protein 63	14q24.3	57156	9.14	8.52	-0.61	-3.85	1.44E-04	2.13E-03	down CINSARC High vs. Low
TSPAN10	tetraspanin 10	17q25.3	83882	5.23	4.47	-0.76	-3.87	1.31E-04	1.99E-03	down CINSARC High vs. Low
ARHGEF37	Rho guanine nucleotide ex	5q32	389337	9.25	8.66	-0.59	-3.89	1.24E-04	1.91E-03	down CINSARC High vs. Low
RAI2	retinoic acid induced 2	Xp22.13	10742	8.76	8.01	-0.75	-3.90	1.20E-04	1.85E-03	down CINSARC High vs. Low
TPRG1	tumor protein p63 regulate	3q28	285386	9.29	8.09	-1.20	-3.95	9.91E-05	1.61E-03	down CINSARC High vs. Low
HPN	hepsin	19q13.11	3249	9.95	9.28	-0.67	-3.96	9.56E-05	1.57E-03	down CINSARC High vs. Low
CFAP70	cilia and flagella associated	10q22.2	118491	6.74	6.08	-0.66	-3.97	8.97E-05	1.50E-03	down CINSARC High vs. Low
ACMSD	aminocarboxymuconate se	2q21.3	130013	2.93	1.74	-1.19	-4.02	7.27E-05	1.28E-03	down CINSARC High vs. Low
IGFALS	insulin like growth factor bi	16p13.3	3483	5.90	5.01	-0.90	-4.04	6.81E-05	1.21E-03	down CINSARC High vs. Low
GNMT	glycine N-methyltransferas	6p21.1	27232	4.91	4.07	-0.85	-4.05	6.50E-05	1.18E-03	down CINSARC High vs. Low
ACADS	acyl-CoA dehydrogenase s	12q24.31	35	8.30	7.68	-0.62	-4.06	6.16E-05	1.13E-03	down CINSARC High vs. Low
EPHX2	epoxide hydrolase 2	8p21.2-p21.1	2053	9.11	8.41	-0.70	-4.11	5.20E-05	9.82E-04	down CINSARC High vs. Low
DCDC1	doublecortin domain conta	11p13	341019	3.39	2.65	-0.74	-4.14	4.44E-05	8.68E-04	down CINSARC High vs. Low
IZUMO4	IZUMO family member 4	19p13.3	113177	4.65	4.00	-0.65	-4.18	3.91E-05	7.85E-04	down CINSARC High vs. Low
C17orf107	chromosome 17 open reac	17p13.2	100130311	6.27	5.64	-0.63	-4.18	3.85E-05	7.74E-04	down CINSARC High vs. Low
NOSTRIN	nitric oxide synthase traffic	2q24.3	115677	8.83	8.14	-0.69	-4.21	3.35E-05	6.91E-04	down CINSARC High vs. Low
ADAMTS13	ADAM metallopeptidase wi	9q34.2	11093	6.41	5.82	-0.59	-4.22	3.20E-05	6.73E-04	down CINSARC High vs. Low
ZBTB16	zinc finger and BTB domai	11q23.2	7704	4.30	3.29	-1.02	-4.26	2.69E-05	5.86E-04	down CINSARC High vs. Low
EFCAB6	EF-hand calcium binding d	22q13.2-q13.31	64800	5.68	5.08	-0.60	-4.34	1.95E-05	4.55E-04	down CINSARC High vs. Low
FCGBP	Fc fragment of IgG binding	19q13.2	8857	8.70	7.76	-0.94	-4.38	1.66E-05	4.07E-04	down CINSARC High vs. Low
KCNQ3	potassium voltage-gated c	8q24.22	3786	4.18	3.43	-0.75	-4.40	1.48E-05	3.75E-04	down CINSARC High vs. Low
RSPH1	radial spoke head compon	21q22.3	89765	7.45	6.75	-0.70	-4.42	1.38E-05	3.54E-04	down CINSARC High vs. Low
DEGS2	delta 4-desaturase, sphing	14q32.2	123099	10.61	9.83	-0.78	-4.43	1.35E-05	3.51E-04	down CINSARC High vs. Low
FSCN2	fascin actin-bundling protei	17q25.3	25794	4.55	3.80	-0.74	-4.43	1.34E-05	3.49E-04	down CINSARC High vs. Low
PLD4	phospholipase D family me	14q32.33	122618	6.30	5.53	-0.78	-4.43	1.34E-05	3.49E-04	down CINSARC High vs. Low
ZMYND10	zinc finger MYND-type con	3p21.31	51364	8.38	7.71	-0.67	-4.46	1.16E-05	3.11E-04	down CINSARC High vs. Low
CHRNA2	cholinergic receptor nicotin	8p21.2	1135	1.22	0.59	-0.63	-4.47	1.14E-05	3.07E-04	down CINSARC High vs. Low
WDR93	WD repeat domain 93	15q26.1	56964	5.13	4.48	-0.65	-4.51	9.27E-06	2.65E-04	down CINSARC High vs. Low
CACNA1F	calcium voltage-gated chai	Xp11.23	778	4.29	3.21	-1.08	-4.56	7.51E-06	2.24E-04	down CINSARC High vs. Low
MDGA1	MAM domain containing gl	6p21.2	266727	7.17	6.53	-0.64	-4.58	6.95E-06	2.09E-04	down CINSARC High vs. Low
SCUBE2	signal peptide, CUB domai	11p15.4	57758	12.63	11.44	-1.19	-4.58	6.89E-06	2.09E-04	down CINSARC High vs. Low
SERPINA1	serpin family A member 1	14q32.13	5265	11.24	10.11	-1.13	-4.60	6.21E-06	1.93E-04	down CINSARC High vs. Low
MAGI2	membrane associated gua	7q21.11	9863	8.11	7.34	-0.77	-4.65	4.91E-06	1.60E-04	down CINSARC High vs. Low
ANKRD24	ankyrin repeat domain 24	19p13.3	170961	5.04	4.28	-0.76	-4.67	4.53E-06	1.51E-04	down CINSARC High vs. Low
RAB30	RAB30, member RAS onc	11q14.1	27314	8.74	7.93	-0.81	-4.72	3.68E-06	1.27E-04	down CINSARC High vs. Low
TENT5B	terminal nucleotidyltransfer	1p36.11	115572	7.97	6.97	-1.00	-4.73	3.40E-06	1.20E-04	down CINSARC High vs. Low
SPEF1	sperm flagellar 1	20p13	25876	5.74	4.90	-0.84	-4.76	3.05E-06	1.10E-04	down CINSARC High vs. Low
AZGP1	alpha-2-glycoprotein 1, zin	7q22.1	563	14.26	13.30	-0.96	-4.76	3.02E-06	1.10E-04	down CINSARC High vs. Low
BCAN	brevican	1q23.1	63827	3.77	2.82	-0.95	-4.86	1.89E-06	7.27E-05	down CINSARC High vs. Low
TSNAXIP1	translin associated factor X	16q22.1	55815	5.03	4.32	-0.71	-4.93	1.35E-06	5.55E-05	down CINSARC High vs. Low
PRICKLE4	prickle planar cell polarity p	6p21.1	29964	6.36	5.60	-0.76	-5.00	1.00E-06	4.25E-05	down CINSARC High vs. Low
TNNC1	troponin C1, slow skeletal a	3p21.1	7134	2.75	1.94	-0.81	-5.12	5.53E-07	2.57E-05	down CINSARC High vs. Low
C2orf73	chromosome 2 open readi	2p16.2	129852	2.25	1.52	-0.72	-5.13	5.30E-07	2.48E-05	down CINSARC High vs. Low
CFAP43	cilia and flagella associated	10q25.1	80217	6.17	5.29	-0.89	-5.18	4.17E-07	1.98E-05	down CINSARC High vs. Low
NUDT18	nudix hydrolase 18	8p21.3	79873	7.66	7.07	-0.59	-5.21	3.59E-07	1.76E-05	down CINSARC High vs. Low
PEBP4	phosphatidylethanolamine	8p21.3	157310	2.83	1.56	-1.27	-5.22	3.33E-07	1.64E-05	down CINSARC High vs. Low
RHBDL1	rhomboid like 1	16p13.3	9028	6.40	5.40	-1.01	-5.23	3.18E-07	1.58E-05	down CINSARC High vs. Low
DUSP27	dual specificity phosphatas	1q24.1	92235	2.87	2.00	-0.87	-5.24	3.00E-07	1.50E-05	down CINSARC High vs. Low
CCDC189	coiled-coil domain containi	16p11.2	90835	6.75	6.05	-0.70	-5.32	2.02E-07	1.07E-05	down CINSARC High vs. Low
KIF13B	kinesin family member 13B	8p12	23303	10.79	10.05	-0.74	-5.33	1.96E-07	1.04E-05	down CINSARC High vs. Low
CX3CR1	C-X3-C motif chemokine re	3p22.2	1524	7.15	6.21	-0.95	-5.34	1.87E-07	1.01E-05	down CINSARC High vs. Low
DNAAF1	dynein axonemal assembly	16q24.1	123872	5.56	4.52	-1.04	-5.36	1.67E-07	9.06E-06	down CINSARC High vs. Low
CATSPERG	cation channel sperm assc	19q13.2	57828	5.15	4.39	-0.76	-5.41	1.30E-07	7.30E-06	down CINSARC High vs. Low
NHLRC4	NHL repeat containing 4	16p13.3	283948	6.37	5.31	-1.06	-5.42	1.24E-07	7.00E-06	down CINSARC High vs. Low
CASC1	cancer susceptibility 1	12p12.1	55259	5.95	5.14	-0.81	-5.42	1.23E-07	6.96E-06	down CINSARC High vs. Low
RIBC1	RIB43A domain with coiled	Xp11.22	158787	6.54	5.86	-0.68	-5.67	3.44E-08	2.28E-06	down CINSARC High vs. Low
DRC3	dynein regulatory complex	17p11.2	83450	6.63	5.75	-0.88	-5.68	3.28E-08	2.20E-06	down CINSARC High vs. Low
C3orf18	chromosome 3 open readi	3p21.31	51161	7.78	7.06	-0.72	-5.81	1.57E-08	1.15E-06	down CINSARC High vs. Low
TTC12	tetratricopeptide repeat do	11q23.2	54970	8.40	7.80	-0.60	-6.05	4.31E-09	3.66E-07	down CINSARC High vs. Low
TTC36	tetratricopeptide repeat do	11q23.3	143941	6.17	4.87	-1.30	-6.06	4.04E-09	3.46E-07	down CINSARC High vs. Low
MORN1	MORN repeat containing 1	1p36.32	79906	6.71	5.88	-0.82	-6.37	7.18E-10	7.46E-08	down CINSARC High vs. Low
C16orf71	chromosome 16 open reac	16p13.3	146562	5.96	5.05	-0.90	-7.19	5.37E-12	7.46E-10	down CINSARC High vs. Low
TMEM25	transmembrane protein 25	11q23.3	84866	10.38	9.71	-0.66	-7.50	7.61E-13	1.29E-10	down CINSARC High vs. Low

Supplementary Table 8: Ontologies of the 510 genes differentially expressed between the two CINSARC classes in Luminal B breast cancers.

Terms ID	Terms	up CINSARC High vs. Low				down CINSARC High vs. Low			
		N	Gene	p-value	q-value	N	Gene	p-value	q-value
GO:00002	mitotic cell cycle	42	*CENPA,BUB1,NCAF*	3.43E-21	2.02E-18				
GO:00070	mitotic nuclear division	32	*BUB1,CCNA2,CCNE*	3.84E-20	1.13E-17				
GO:00070	chromosome segregation	14	*BUB1,HJURP,BIRC5*	9.02E-13	1.77E-10				
GO:00000	mitotic sister chromatid segregation	8	*SPAG5,MAD2L1,ND*	1.81E-10	2.66E-08				
GO:00070	mitotic spindle assembly checkpoint	8	*BUB1,TTK,BUB1B,C*	6.20E-09	7.29E-07				
GO:00002	mitotic cytokinesis	6	*CEP55,KIF23,KIF20*	2.07E-07	1.69E-05				
GO:00070	microtubule depolymerization	4	*KIF14,KIF2C,KIF18E*	2.16E-07	1.69E-05				
GO:00009	cytokinesis	8	*BIRC5,KIF23,PRC1,*	2.30E-07	1.69E-05				
GO:00070	microtubule-based movement	9	*KIF14,KIF23,CENPE*	3.07E-07	2.01E-05				
GO:00072	small GTPase mediated signal transdu	29	*CENPA,BUB1,BIRC*	5.78E-07	3.40E-05				
GO:00000	G2/M transition of mitotic cell cycle	12	*CCNA2,CCNB2,BIR*	1.49E-06	7.98E-05				
GO:00000	regulation of transcription involved in G	5	*RRM2,CDK1,ORC1,*	1.88E-06	9.23E-05				
GO:00070	mitotic metaphase plate congression	6	*CEP55,KIF14,CENP*	3.03E-06	1.37E-04				
GO:00070	spindle organization	4	*TTK,SPAG5,ASPM,I*	4.71E-06	1.98E-04				
GO:00000	regulation of cyclin-dependent protein	6	*CCNA2,BLM,CDKN3*	5.33E-06	2.01E-04				
GO:00062	DNA replication initiation	5	*MCM10,ORC1,CCNI*	5.47E-06	2.01E-04				
GO:00070	mitotic spindle organization	5	*TTK,AURKA,NDC80*	1.08E-05	3.73E-04				
GO:00903	mitotic spindle assembly	5	*BIRC5,TPX2,KIF11,*	2.37E-05	7.33E-04				
GO:00103	regulation of G2/M transition of mitotic	3	*KIF14,CENPF,PKIA*	3.06E-05	8.99E-04				
GO:00062	DNA replication	10	*TICRR,BLM,MCM10*	3.78E-05	1.06E-03				
GO:00310	endocrine pancreas development	5	*ONECUT2,HNF4G,F*	4.68E-05	1.25E-03				
GO:00074	peripheral nervous system developme	4	*GFRA3,UGT8,SCN8*	5.84E-05	1.49E-03				
GO:00070	mitotic G2 DNA damage checkpoint	3	*CCNA2,BLM,CDK1*	6.44E-05	1.58E-03				
GO:00000	G1/S transition of mitotic cell cycle	10	*MCM10,RRM2,CDK*	9.74E-05	2.29E-03				
GO:00068	ion transport	10	*CHRNA5,SLC7A5,S*	1.23E-04	2.78E-03				
GO:00311	mRNA 3-end processing	220	*CENPA,BUB1,NCAF*	1.63E-04	3.56E-03				
GO:00070	mitotic cell cycle checkpoint	4	*BUB1,TTK,BUB1B,M*	1.79E-04	3.76E-03				
GO:00198	antigen processing and presentation o	7	*KIF23,CENPE,KIF2*	2.16E-04	4.37E-03				
GO:00901	positive regulation of branching involve	3	*LHX1,SIX1,PAX2*	3.21E-04	6.28E-03				
GO:00082	cell proliferation	15	*BUB1,BUB1B,KIF2C*	5.42E-04	1.03E-02				
GO:00016	urogenital system development	2	*LHX1,PAX2*	6.28E-04	1.12E-02				
GO:00158	neutral amino acid transport	2	*SLC7A5,SLC6A15*	6.28E-04	1.12E-02				
GO:00082	positive regulation of cell proliferation	17	*BIRC5,KIF14,FOXM*	6.68E-04	1.12E-02				
GO:00063	double-strand break repair	8	*KPNA2,BLM,TRIP13*	7.55E-04	1.23E-02				
GO:00025	chronic inflammatory response	2	*CAMP,S100A8*	8.53E-04	1.29E-02				
GO:00063	mitotic recombination	2	*TOP2A,RAD54B*	8.53E-04	1.29E-02				
GO:00310	response to caffeine	2	*DNMT3B,IL6*	8.53E-04	1.29E-02				
GO:00063	nucleosome assembly	6	*CENPA,HJURP,CEN*	9.70E-04	1.43E-02				
GO:00073	nervous system development	12	*GFRA3,SLC7A5,SC*	1.06E-03	1.43E-02				
GO:00017	organ induction	2	*SIX1,HOXC11*	1.12E-03	1.43E-02				
GO:00070	mitotic chromosome condensation	2	*NCAPH,NCAPG*	1.12E-03	1.43E-02				
GO:00164	somatic hypermutation of immunoglob	2	*POLQ,EXO1*	1.12E-03	1.43E-02				
GO:00218	layer formation in cerebral cortex	2	*LRP8,CDK5R2*	1.12E-03	1.43E-02				
GO:00218	cell proliferation in forebrain	2	*KIF14,ARX*	1.12E-03	1.43E-02				
GO:19035	regulation of nucleic acid-templated tra	3	*TRIP13,SAMD11,AC*	1.30E-03	1.62E-02				
GO:00100	glial cell differentiation	2	*NFIB,PAX2*	1.44E-03	1.73E-02				
GO:00165	protein autoprocessing	2	*CTSV,PCSK2*	1.44E-03	1.73E-02				
GO:00714	cellular response to ionizing radiation	3	*BLM,ECT2,RAD51A*	1.49E-03	1.75E-02				
GO:00018	response to yeast	2	*CAMP,IL6*	1.81E-03	2.00E-02				
GO:00022	innate immune response in mucosa	2	*CAMP,BPIFB1*	1.81E-03	2.00E-02				
GO:00105	regulation of double-strand break repa	2	*RAD51AP1,FIGN*	1.81E-03	2.00E-02				
GO:00068	neurotransmitter transport	3	*SLC6A15,SLC6A11,*	1.94E-03	2.07E-02				
GO:00071	cell communication	3	*CCL18,FRAS1,GJB*	1.94E-03	2.07E-02				
GO:00066	leukotriene metabolic process	2	*CYP4F3,ALOX15*	2.23E-03	2.27E-02				
GO:19043	positive regulation of telomere capping	2	*NEK2,AURKB*	2.23E-03	2.27E-02				
GO:00062	DNA repair	14	*FOXM1,KPNA2,PAR*	2.24E-03	2.27E-02				
GO:00068	amino acid transport	3	*SLC7A5,SLC6A15,S*	2.48E-03	2.35E-02				
GO:00100	response to organic substance	3	*SQLE,AQP9,TRPA1*	2.48E-03	2.35E-02				
GO:20012	positive regulation of intrinsic apoptoti	3	*BUB1,IL19,S100A8*	2.48E-03	2.35E-02				
GO:00068	regulation of pH	2	*SLC9A2,ATP6V0A4*	2.71E-03	2.53E-02				
GO:00009	cell separation after cytokinesis	2	*CEP55,KIF20A*	3.24E-03	2.85E-02				
GO:00015	oocyte maturation	2	*TRIP13,EREG*	3.24E-03	2.85E-02				
GO:00020	morphogenesis of an epithelium	2	*SERPINB5,FRAS1*	3.24E-03	2.85E-02				
GO:19038	positive regulation of dendrite extensio	2	*RIMS1,RASAL1*	3.24E-03	2.85E-02				
GO:00026	positive regulation of leukocyte chemo	2	*IL6,CXCL9*	3.84E-03	3.27E-02				
GO:00099	anterior/posterior axis specification	2	*AURKA,LHX1*	3.84E-03	3.27E-02				
GO:00062	DNA-dependent DNA replication	2	*POLQ,ORC6*	4.50E-03	3.78E-02				
GO:00020	sprouting angiogenesis	2	*E2F8,E2F7*	5.23E-03	4.21E-02				
GO:00901	cochlea development	2	*PAX2,KCNK3*	5.23E-03	4.21E-02				
GO:00025	monocyte chemotaxis	3	*CCL18,IL6,CCL21*	5.62E-03	4.47E-02				
GO:00001	establishment of mitotic spindle orienta	2	*CENPA,NDC80*	6.02E-03	4.59E-02				
GO:00072	gamma-aminobutyric acid signaling pa	2	*GABBR2,GABRA3*	6.02E-03	4.59E-02				
GO:00901	cochlea morphogenesis	2	*SIX1,PAX2*	6.02E-03	4.59E-02				
GO:00713	cellular response to interleukin-1	4	*CAMP,CCL18,IL6,C*	6.23E-03	4.69E-02				
GO:00070	mitotic nuclear envelope disassembly	3	*CCNB2,CDK1,PLK1*	6.70E-03	4.87E-02				
GO:00308	positive regulation of actin filament pol	3	*PFN2,CCL21,ALOX*	6.70E-03	4.87E-02				
GO:00075	sex differentiation	2	*CENPI,DMRTA1*	6.87E-03	4.87E-02				
GO:00108	retina layer formation	2	*SDK2,LHX1*	6.87E-03	4.87E-02				
GO:00715	cellular response to dexamethasone s	2	*DNMT3B,IL6*	6.87E-03	4.87E-02				
GO:00063	DNA recombination	4	*BLM,PIF1,EXO1,MN*	7.07E-03	4.95E-02				
GO:00016	branching involved in ureteric bud mor	3	*LHX1,SIX1,PAX2*	7.28E-03	0.050				
GO:00007	double-strand break repair via homolo	6	*BLM,POLQ,RAD51A*	7.77E-03	0.052				
GO:00018	positive regulation of cytokine producti	2	*EREG,LEP*	7.80E-03	0.052				
GO:00713	cellular response to epidermal growth	2	*GAREM,PAX2*	7.80E-03	0.052				
GO:00015	microtubule bundle formation	2	*KIF20A,PLK1*	8.80E-03	0.057				

Terms ID	Terms	up CINSARC High vs. Low N	Gene	p-value	q-value	down CINSARC High vs. Low N	Gene	p-value	q-value
GO:00017	neuron migration	5	*ASPM,GFRA3,FGF1*	9.79E-03	0.061				
GO:00109	negative regulation of phosphatase ac	3	*CASC5,PCDH11X,E*	9.95E-03	0.061				
GO:00002	microtubule cytoskeleton organization	4	*BIRC5,PRC1,CDK1,*	1.07E-02	0.061				
GO:00071	heterophilic cell-cell adhesion via plası	3	*TENM2,L1CAM,CBL*	1.07E-02	0.061				
GO:00986	anion transmembrane transport	3	*SLC4A8,SLCO4C1,S*	1.07E-02	0.061				
GO:00062	DNA metabolic process	2	*KPNA2,MKI67*	1.10E-02	0.061				
GO:00073	pattern specification process	2	*LHX1,SIX1*	1.10E-02	0.061				
GO:00099	proximal/distal pattern formation	2	*HOXC10,HOXC11*	1.10E-02	0.061				
GO:00709	protein K11-linked ubiquitination	2	*UBE2C,UBE2T*	1.22E-02	0.061				
GO:00015	retinoid metabolic process	3	*LRP8,GPC2,LRAT*	1.32E-02	0.061				
GO:00197	antibacterial humoral response	2	*CAMP,IGJ*	1.35E-02	0.062				
GO:00072	spermatogenesis	11	*TRIP13,ASPM,DIAP*	1.37E-02	0.062				
GO:00301	cell differentiation	13	*FOXM1,CENPF,CB*	1.47E-02	0.063				
GO:00033	amino acid transmembrane transport	2	*SLC7A5,SLC6A14*	1.49E-02	0.063				
GO:00071	reciprocal meiotic recombination	2	*TRIP13,RAD54B*	1.49E-02	0.063				
GO:00215	cerebellum development	2	*LHX1,CDK5R2*	1.49E-02	0.063				
GO:00718	DNA biosynthetic process	2	*CENPF,POLQ*	1.49E-02	0.063				
GO:00096	response to toxic substance	4	*DNMT3B,SCN8A,TT*	1.53E-02	0.063				
GO:00076	phototransduction, visible light	4	*LRP8,CNGA2,GPC2*	1.69E-02	0.066				
GO:00193	arachidonic acid metabolic process	3	*CYP2J2,CYP4F3,AL*	1.70E-02	0.066				
GO:00080	regulation of heart contraction	2	*CYP2J2,TNNT2*	1.78E-02	0.066				
GO:00159	ATP hydrolysis coupled proton transpo	2	*ATP1A3,ATP6V0A4*	1.78E-02	0.066				
GO:00300	insulin secretion	2	*FAM3B,LEP*	1.94E-02	0.067				
GO:00071	homophilic cell adhesion via plasma m	6	*SDK2,DSC2,L1CAM*	2.01E-02	0.067				
GO:00311	organ regeneration	3	*CCNA2,MKI67,CCN*	2.03E-02	0.067				
GO:00071	negative regulation of cell adhesion	2	*PDE3B,ADAMDEC1*	2.28E-02	0.069				
GO:00713	cellular response to tumor necrosis fac	4	*CAMP,CCL18,IL6,C*	2.41E-02	0.071				
GO:00068	water transport	2	*ADCY5,AQP9*	2.46E-02	0.071				
GO:00906	activation of GTPase activity	2	*ECT2,CCL21*	2.46E-02	0.071				
GO:00076	sensory perception of sound	5	*SCN8A,GJB3,COCH*	2.55E-02	0.071				
GO:00075	excretion	2	*AQP9,ATP6V0A4*	2.65E-02	0.072				
GO:00094	response to cold	2	*IL6,TRPA1*	2.65E-02	0.072				
GO:00100	response to zinc ion	2	*GGH,S100A8*	2.65E-02	0.072				
GO:00157	bicarbonate transport	2	*SLC4A8,SLC4A10*	2.65E-02	0.072				
GO:00063	transcription from RNA polymerase II p	15	*FOXM1,TRIP13,PTT*	2.89E-02	0.074				
GO:00016	ureteric bud development	2	*LHX1,SIX1*	3.05E-02	0.077				
GO:00903	phagosome maturation	2	*CAMP,ATP6V0A4*	3.05E-02	0.077				
GO:00075	blood coagulation	13	*KIF23,CENPE,KIF2*	3.25E-02	0.080				
GO:00713	cellular response to retinoic acid	3	*PAX2,BRINP3,LEP*	3.40E-02	0.083				
GO:00022	hematopoietic progenitor cell differenti	3	*TOP2A,ANLN,PTPR*	3.89E-02	0.091				
GO:00064	protein phosphorylation	14	*BUB1,BIRC5,TTK,B*	3.91E-02	0.091				
GO:00303	embryonic limb morphogenesis	2	*FRAS1,HOXC10*	3.94E-02	0.091				
GO:00073	regulation of mitotic cell cycle	3	*BIRC5,PLK1,RPRM*	4.06E-02	0.091				
GO:00001	negative regulation of transcription fro	18	*FOXM1,PLK1,AURK*	4.08E-02	0.091				
GO:00068	sodium ion transport	3	*SCN8A,ATP1A3,SL*	4.42E-02	0.098				
GO:00140	response to organic cyclic compound	2	*MKI67,TRPA1*	4.69E-02	0.101				
GO:00160	lipid catabolic process	3	*PLCB4,PLCH1,PLA*	4.80E-02	0.102				
GO:00018	placenta development	5	*E2F8,E2F7,DLX3,G*	1.97E-05	6.42E-04	2	*CITED1,NDP*	4.45E-03	4.21E-02
GO:00162	O-glycan processing	5	*MUC13,ST6GALNA*	6.51E-04	1.12E-02	2	*MUC1,GALNT5*	2.38E-02	0.067
GO:00725	reactive oxygen species metabolic pro	3	*IL19,AOX1,PAX2*	2.48E-03	2.35E-02	2	*MPV17L,EPHX2*	4.45E-03	4.21E-02
GO:00072	cell-cell signaling	8	*AREG,FGF13,CCL1*	2.09E-02	0.067	5	*NDP,CARTPT,GJA1*	3.11E-02	0.080
GO:00217	hippocampus development	3	*KIF14,FGF13,CDK5*	1.15E-02	0.061	2	*DLX2,BCAN*	1.45E-02	0.053
GO:00064	protein O-linked glycosylation	4	*MUC13,GALNT3,B3*	1.32E-02	0.061	3	*MUC1,GALNT5,AD*	9.03E-03	4.21E-02
GO:00074	axonogenesis	4	*SLITRK5,PTPRZ1,P*	1.77E-02	0.066	3	*SPTBN4,ADCY1,CA*	1.16E-02	4.86E-02
GO:00065	cellular amino acid metabolic process	2	*SLC7A5,SLC6A14*	2.11E-02	0.067	2	*SLC7A2,SLC7A4*	4.86E-03	4.21E-02
GO:00076	adult walking behavior	2	*SCN8A,EFNB3*	2.11E-02	0.067	2	*KLHL1,SPTBN4*	4.86E-03	4.21E-02
GO:00900	establishment of protein localization to	2	*TSPAN5,ACTN2*	1.94E-02	0.067	2	*SPTBN4,TSPAN10*	4.45E-03	4.21E-02
GO:00300	muscle filament sliding	3	*TNNI1,TNNT2,ACT*	4.67E-03	3.87E-02	3	*TNNI3,MYBPC1,TN*	6.45E-04	4.21E-02
GO:00072	synaptic transmission	12	*GABBR2,CHRNA5,S*	2.65E-02	0.072	10	*GRIA2,CARTPT,P2*	2.95E-03	4.21E-02
GO:00085	epidermis development					2	*KRT15,CDSN*	4.98E-02	0.099
GO:00025	platelet degranulation					2	*CLU,SERPINA1*	4.53E-02	0.094
GO:00074	brain development					4	*CITED1,DLX2,C11o*	4.50E-02	0.094
GO:00065	proteolysis					8	*WFDC2,TPSG1,CF*	4.30E-02	0.092
GO:00071	cell adhesion					8	*COL4A6,CDSN,MY*	3.95E-02	0.088
GO:00986	cation transmembrane transport					2	*TMEM63C,CHRNA2*	3.43E-02	0.083
GO:00074	central nervous system development					3	*CHST8,ZBTB16,BC*	2.91E-02	0.076
GO:00072	neurotransmitter secretion					2	*GAD1,ABAT*	2.71E-02	0.073
GO:00718	potassium ion transmembrane transpo					3	*KCNE4,HPN,KCNQ*	2.29E-02	0.066
GO:00015	vasculogenesis					2	*TNNI3,CITED1*	2.28E-02	0.066
GO:00165	negative regulation of angiogenesis					2	*BAI2,CX3CR1*	1.89E-02	0.060
GO:00160	synaptic vesicle exocytosis					2	*SYT13,SYT12*	1.62E-02	0.056
GO:00072	phospholipase C-activating G-protein c					2	*AGTR1,P2RY11*	1.54E-02	0.053
GO:00303	sperm motility					2	*ATP1A4,TEKT3*	1.54E-02	0.053
GO:00067	glutathione metabolic process					2	*CLIC6,GSTM2*	1.38E-02	0.051
GO:00069	vesicle fusion					2	*SYT13,SYT12*	1.38E-02	0.051
GO:00303	negative regulation of cell migration					3	*CHRD,MAGI2,CX3C*	1.31E-02	0.050
GO:00300	actin cytoskeleton organization					4	*KLHL1,FGD3,ABLI*	1.22E-02	4.95E-02
GO:00071	adenylate cyclase-activating G-protein					2	*P2RY11,ADCY1*	1.09E-02	4.70E-02
GO:00068	potassium ion transport					3	*ATP1A4,KCND3,KC*	1.03E-02	4.57E-02
GO:00305	negative regulation of BMP signaling p					2	*CHRD,TMPRSS6*	8.94E-03	4.21E-02
GO:00095	fertilization					2	*ATP1A4,SPTBN4*	8.35E-03	4.21E-02
GO:00068	xenobiotic metabolic process					5	*CYP2A7,NAT1,ACS*	8.27E-03	4.21E-02
GO:00076	visual perception					5	*NDP,MYO3B,NXNL*	8.27E-03	4.21E-02
GO:00097	embryo development					2	*FOXI1,RAI2*	6.72E-03	4.21E-02
GO:20012	negative regulation of extrinsic apopto					2	*FGF10,CX3CR1*	5.29E-03	4.21E-02
GO:00171	regulation of calcium ion-dependent ex					2	*SYT13,SYT12*	4.86E-03	4.21E-02
GO:00164	protein processing					3	*RHBDL3,ADAMTS1*	4.50E-03	4.21E-02

Terms ID	Terms	up CINSARC High vs. Low				down CINSARC High vs. Low			
		N	Gene	p-value	q-value	N	Gene	p-value	q-value
GO:00016[illegible]	metanephros development					2	*CITED1,FGF10*	3.70E-03	4.21E-02
GO:00018[illegible]	retina homeostasis					2	*PIP,AZGP1*	3.70E-03	4.21E-02
GO:00068[illegible]	transport					9	*SLC7A2,SLC25A48,*	3.70E-03	4.21E-02
GO:00068[illegible]	cellular calcium ion homeostasis					4	*TNNI3,STC2,EPHX2*	3.06E-03	4.21E-02
GO:00076[illegible]	long-term memory					2	*ADCY1,GRIA1*	2.73E-03	4.21E-02
GO:00069[illegible]	complement activation					2	*CFB,CLU*	2.44E-03	4.21E-02
GO:00723[illegible]	intrinsic apoptotic signaling pathway by					2	*ZNF385D,ZNF385B*	2.44E-03	4.21E-02
GO:00109[illegible]	negative regulation of endopeptidase a					5	*CST9,WFDC2,CST5*	1.57E-03	4.21E-02
GO:00015[illegible]	detection of chemical stimulus involved					2	*PIP,AZGP1*	1.49E-03	4.21E-02
GO:00033[illegible]	cilium movement					2	*CFAP221,DNAAF1*	1.49E-03	4.21E-02
GO:00098[illegible]	embryonic pattern specification					2	*FGF10,ZBTB16*	1.49E-03	4.21E-02
GO:00197[illegible]	calcium-mediated signaling					3	*AGTR1,P2RY11,LA*	1.13E-03	4.21E-02
GO:00193[illegible]	epoxygenase P450 pathway					2	*CYP2A7,EPHX2*	9.61E-04	4.21E-02
GO:00069[illegible]	acute-phase response					3	*ORM1,ORM2,SERP*	2.91E-04	3.23E-02
GO:00216[illegible]	cerebellar Purkinje cell layer developm					2	*SEZ6L,KLHL1*	2.30E-04	3.23E-02
GO:00018[illegible]	mesonephros development					2	*FGF10,ZBTB16*	1.74E-04	3.23E-02

Supplementary Table 9: Comparison of expression levels of 226 proteins/phosphoproteins between the two CINSARC classes in Luminal B TCGA breast cancers.

Gene#Prot	CINSARC, High- vs. Low-risk			
	N	Odds ratio [95%CI]	p-value	q-value
CCNB1#Cyclin_B1	240	1.66 [1.41-1.96]	6.53E-07	1.46E-04
FASN#FASN	240	1.41 [1.09-1.83]	2.85E-02	0.227
MSH6#MSH6	240	1.32 [1.19-1.47]	1.38E-05	1.03E-03
FOXM1#FoxM1	240	1.31 [1.20-1.44]	2.31E-06	2.58E-04
RPS6KB1#p70S6K	240	1.28 [1.11-1.47]	3.86E-03	0.086
EIF4EBP1#4E-BP1	240	1.26 [1.08-1.46]	1.22E-02	0.143
SYK#Syk	240	1.26 [1.10-1.44]	4.65E-03	0.093
ACACA#ACC1	240	1.24 [1.03-1.49]	0.0548	0.313
SLC1A5#SLC1A5	218	1.24 [1.07-1.43]	1.52E-02	0.161
ENY2#ENY2	218	1.23 [1.10-1.37]	2.46E-03	0.063
TSC1#TSC1	240	1.23 [1.08-1.40]	9.29E-03	0.117
ACACA ACACB#ACC_pS79	240	1.21 [1.02-1.44]	0.0717	0.385
GAPDH#GAPDH	240	1.21 [0.99-1.49]	0.12	0.532
TFRC#TFRC	240	1.21 [1.04-1.40]	3.88E-02	0.277
YWHAZ#14-3-3_zeta	240	1.20 [1.09-1.33]	2.21E-03	0.063
ASNS#ASNS	240	1.20 [1.08-1.33]	5.48E-03	0.093
CASP7#Caspase-7_cleavedD198	240	1.20 [1.03-1.39]	4.68E-02	0.291
RBM15#RBM15	240	1.20 [1.03-1.40]	4.80E-02	0.291
NKX2-1#TTF1	22	1.20 [0.76-1.91]	0.514	0.811
TUBA1B#Acetyl-a-Tubulin-Lys40	240	1.19 [0.96-1.46]	0.178	0.584
KAT2A#GCN5L2	218	1.19 [1.07-1.32]	6.22E-03	0.093
SCD#SCD	240	1.18 [0.94-1.49]	0.234	0.643
MYH11#MYH11	240	1.17 [0.83-1.65]	0.455	0.799
EIF4G1#eIF4G	240	1.17 [1.03-1.33]	4.82E-02	0.291
IGFBP2#IGFBP2	240	1.16 [0.94-1.43]	0.252	0.643
XRCC5#Ku80	240	1.15 [1.06-1.25]	6.65E-03	0.093
NFKB1#NF-kB-p65_pS536	240	1.15 [1.00-1.32]	0.104	0.494
STAT5A#STAT5-alpha	240	1.15 [0.99-1.33]	0.123	0.532
ERBB2#HER2	240	1.14 [0.97-1.34]	0.197	0.605
INPP4B#INPP4B	240	1.14 [0.96-1.36]	0.206	0.605
EEF2#eEF2	240	1.14 [1.05-1.24]	9.44E-03	0.117
CCNE2#Cyclin_E2	240	1.13 [1.06-1.20]	1.10E-03	0.061
EIF4EBP1#4E-BP1_pT70	240	1.12 [1.05-1.20]	6.61E-03	0.093
PRKAA1#AMPK_pT172	240	1.12 [0.98-1.26]	0.151	0.570
CCNE1#Cyclin_E1	240	1.12 [1.02-1.22]	4.17E-02	0.277
GSK3A GSK3B#GSK3-alpha-beta	240	1.12 [1.03-1.22]	2.67E-02	0.221
MSH2#MSH2	240	1.12 [1.04-1.21]	1.39E-02	0.155
RPS6#S6	240	1.12 [0.99-1.26]	0.124	0.532
RPS6#S6_pS240_S244	240	1.12 [0.96-1.30]	0.213	0.617
EIF4EBP1#4E-BP1_pS65	240	1.11 [1.03-1.19]	1.67E-02	0.163
ADAR#ADAR1	240	1.11 [1.01-1.22]	0.0791	0.401
PARP1#PARP1	109	1.11 [0.86-1.44]	0.491	0.808
PXN#Paxillin	240	1.11 [1.03-1.19]	1.87E-02	0.174
AKT1 AKT2 AKT3#Akt	240	1.10 [0.98-1.23]	0.169	0.580
ANXA1#Annexin-1	240	1.10 [0.97-1.25]	0.2	0.605
CDK1#CDK1	240	1.09 [1.03-1.16]	1.68E-02	0.163
LCK#Lck	240	1.09 [0.99-1.21]	0.15	0.570
PTEN#PTEN	240	1.09 [0.99-1.20]	0.128	0.539
TSC2#Tuberin	240	1.09 [0.99-1.21]	0.154	0.570
CDKN1B#p27_pT198	240	1.09 [1.03-1.14]	6.07E-03	0.093
SQSTM1#p62-LCK-ligand	240	1.09 [0.89-1.32]	0.483	0.808
PRKCB#PKC-pan_BetaII_pS660	240	1.08 [0.98-1.18]	0.204	0.605
EIF4E#eIF4E	240	1.08 [1.02-1.14]	2.25E-02	0.193

Gene#Prot	CINSARC, High- vs. Low-risk			
	N	Odds ratio [95%CI]	p-value	q-value
EIF4EBP1#4E-BP1_pT37_T46	240	1.07 [0.91-1.26]	0.5	0.808
TP53BP1#53BP1	240	1.07 [0.94-1.21]	0.406	0.774
RAF1#C-Raf	240	1.07 [1.01-1.13]	4.15E-02	0.277
EPPK1#EPPK1	240	1.07 [0.81-1.41]	0.698	0.899
RB1#Rb_pS807_S811	240	1.07 [0.93-1.24]	0.422	0.774
DIABLO#Smac	240	1.07 [0.97-1.19]	0.263	0.643
SMAD3#Smad3	240	1.07 [1.01-1.13]	0.0535	0.313
SRC#Src	240	1.07 [1.00-1.15]	0.103	0.494
MTOR#mTOR	240	1.07 [0.96-1.19]	0.279	0.662
BRD4#BRD4	240	1.06 [0.94-1.19]	0.418	0.774
DVL3#Dvl3	240	1.06 [1.00-1.13]	0.12	0.532
JAK2#Jak2	240	1.06 [0.97-1.14]	0.266	0.643
PCNA#PCNA	240	1.06 [0.99-1.14]	0.173	0.584
RICTOR#Rictor	240	1.06 [0.96-1.16]	0.312	0.718
KIT#c-Kit	240	1.06 [0.90-1.25]	0.564	0.835
CASP3#Caspase-3	240	1.05 [0.97-1.14]	0.333	0.728
CCND1#Cyclin_D1	240	1.05 [0.95-1.17]	0.427	0.774
ERBB2#HER2_pY1248	240	1.05 [0.94-1.19]	0.47	0.808
PRDX1#PRDX1	240	1.05 [0.98-1.12]	0.284	0.667
RAD51#Rad51	240	1.05 [0.99-1.12]	0.202	0.605
RPTOR#Raptor	240	1.05 [0.98-1.13]	0.263	0.643
BAP1#Bap1-c-4	240	1.04 [0.98-1.11]	0.266	0.643
CDK1#CDK1_pY15	240	1.04 [0.95-1.12]	0.485	0.808
ETS1#ETS-1	240	1.04 [0.95-1.14]	0.442	0.788
FOXO3#FOXO3a	240	1.04 [0.97-1.11]	0.341	0.728
G6PD#G6PD	240	1.04 [0.95-1.13]	0.497	0.808
RPS6#S6_pS235_S236	240	1.04 [0.88-1.22]	0.702	0.899
TP53#p53	240	1.04 [0.94-1.15]	0.505	0.808
PRKAA1#AMPK_alpha	240	1.03 [0.95-1.11]	0.577	0.835
MYH9#Myosin-IIa	218	1.03 [0.95-1.11]	0.597	0.835
MYH9#Myosin-IIa_pS1943	240	1.03 [0.93-1.15]	0.626	0.857
PIK3CA #PI3K-p110-alpha	240	1.03 [0.98-1.08]	0.396	0.768
STMN1#Stathmin	240	1.03 [0.96-1.10]	0.499	0.808
YBX1#YB-1	240	1.03 [0.95-1.12]	0.551	0.826
MYC#c-Myc	240	1.03 [0.94-1.13]	0.599	0.835
BRCA2#BRCA2	240	1.02 [0.97-1.07]	0.486	0.808
ITGA2#CD49b	240	1.02 [0.94-1.10]	0.724	0.902
COG3#COG3	240	1.02 [0.92-1.14]	0.755	0.926
CHEK2#Chk2	240	1.02 [0.92-1.14]	0.718	0.901
CLDN7#Claudin-7	240	1.02 [0.85-1.22]	0.869	0.952
GUSP4#DUSP4	240	1.02 [0.85-1.22]	0.882	0.955
GAB2#GAB2	240	1.02 [0.88-1.19]	0.814	0.931
ERBB3#HER3	240	1.02 [0.92-1.13]	0.784	0.930
SERPINE1#PAI-1	240	1.02 [0.88-1.19]	0.824	0.933
RICTOR#Rictor_pT1135	240	1.02 [0.96-1.09]	0.516	0.811
STAT3#STAT3_pY705	240	1.02 [0.94-1.11]	0.661	0.883
SRC#Src_pY527	240	1.02 [0.89-1.17]	0.781	0.930
TIGAR#TIGAR	240	1.02 [0.97-1.07]	0.57	0.835
MAPK14#p38_MAPK	240	1.02 [0.96-1.09]	0.549	0.826
MAPK14#p38_pT180_Y182	240	1.02 [0.90-1.16]	0.809	0.930
ARID1A#ARID1A	240	1.01 [0.94-1.08]	0.855	0.945
AXL#Axl	218	1.01 [0.94-1.09]	0.808	0.930
BRAF#B-Raf	240	1.01 [0.88-1.16]	0.927	0.980
PECAM1#CD31	240	1.01 [0.93-1.09]	0.912	0.973
MAPK1#ERK2	240	1.01 [0.93-1.10]	0.804	0.930
CDH3#P-Cadherin	240	1.01 [0.95-1.08]	0.738	0.915
PEA15#PEA15_pS116	240	1.01 [0.91-1.11]	0.917	0.974

Gene#Prot	CINSARC, High- vs. Low-risk			
	N	Odds ratio [95%CI]	p-value	q-value
SRC#Src_pY416	240	1.01 [0.92-1.11]	0.846	0.945
TGM2#Transglutaminase	240	1.01 [0.94-1.08]	0.795	0.930
MET#c-Met_pY1235	240	1.01 [0.95-1.07]	0.856	0.945
CDKN1B#p27	240	1.01 [0.92-1.10]	0.902	0.967
CDKN1B#p27_pT157	240	1.01 [0.97-1.06]	0.578	0.835
AR#AR	240	1.00 [0.85-1.18]	0.998	0.985
BAD#Bad_pS112	240	1.00 [0.93-1.07]	0.977	0.985
BAK1#Bak	240	1.00 [0.93-1.08]	0.993	0.985
BECN1#Beclin	240	1.00 [0.94-1.06]	0.95	0.985
BCL2L11#Bim	240	1.00 [0.88-1.14]	0.991	0.985
DPP4#CD26	240	1.00 [0.94-1.06]	0.964	0.985
CHEK2#Chk2_pT68	240	1.00 [0.91-1.10]	0.97	0.985
FOXO3#FOXO3a_pS318_S321	240	1.00 [0.96-1.05]	0.855	0.945
MAP2K1#MEK1	240	1.00 [0.92-1.09]	0.968	0.985
NF2#NF2	240	1.00 [0.93-1.07]	0.958	0.985
PRKCA #PKC-alpha_pS657	240	1.00 [0.93-1.08]	0.974	0.985
PRKCD#PKC-delta_pS664	240	1.00 [0.96-1.04]	0.98	0.985
SMAD1#Smad1	240	1.00 [0.95-1.06]	0.98	0.985
XBP1#XBP1	240	1.00 [0.93-1.07]	0.998	0.985
MTOR#mTOR_pS2448	240	1.00 [0.92-1.08]	0.963	0.985
ARAF#A-Raf_pS299	240	0.99 [0.93-1.05]	0.777	0.930
MS4A1#CD20	240	0.99 [0.94-1.05]	0.874	0.952
CASP8#Caspase-8	240	0.99 [0.94-1.05]	0.822	0.933
CHEK1#Chk1_pS296	240	0.99 [0.93-1.06]	0.875	0.952
CHEK1#Chk1_pS345	240	0.99 [0.94-1.05]	0.793	0.930
EGFR#EGFR	240	0.99 [0.94-1.05]	0.76	0.926
NRAS#N-Ras	240	0.99 [0.93-1.04]	0.674	0.895
NDRG1#NDRG1_pT346	240	0.99 [0.84-1.17]	0.96	0.985
SRSF1#SF2	240	0.99 [0.95-1.04]	0.757	0.926
SNAI1#Snail	240	0.99 [0.93-1.04]	0.69	0.899
TSC2#Tuberin_pT1462	240	0.99 [0.91-1.08]	0.898	0.967
XRCC1#XRCC1	240	0.99 [0.94-1.04]	0.679	0.896
BIRC2 #cIAP	240	0.99 [0.94-1.05]	0.837	0.943
ATM#ATM	240	0.98 [0.87-1.11]	0.803	0.930
BAX#Bax	240	0.98 [0.91-1.05]	0.616	0.848
EGFR#EGFR_pY1068	240	0.98 [0.88-1.08]	0.709	0.899
EGFR#EGFR_pY1173	240	0.98 [0.92-1.05]	0.661	0.883
ERCC1#ERCC1	240	0.98 [0.94-1.03]	0.585	0.835
STK11#LKB1	240	0.98 [0.94-1.02]	0.466	0.808
ERRFI1#MIG-6	240	0.98 [0.93-1.03]	0.445	0.788
PARP1#PARP_cleaved	240	0.98 [0.93-1.03]	0.525	0.819
SHC1#Shc_pY317	240	0.98 [0.90-1.05]	0.595	0.835
WWTR1#TAZ	240	0.98 [0.92-1.04]	0.599	0.835
EEF2K#eEF2K	240	0.98 [0.88-1.09]	0.771	0.930
CDKN2A#p16_INK4a	240	0.98 [0.85-1.12]	0.774	0.930
RPS6KB1#p70S6K_pT389	240	0.98 [0.90-1.06]	0.66	0.883
YWHAB#14-3-3_beta	240	0.97 [0.92-1.02]	0.26	0.643
YWHAE#14-3-3_epsilon	240	0.97 [0.93-1.01]	0.259	0.643
BRAF#B-Raf_pS445	240	0.97 [0.87-1.09]	0.691	0.899
BCL2L1#Bcl-xL	240	0.97 [0.92-1.03]	0.42	0.774
BID#Bid	240	0.97 [0.91-1.04]	0.504	0.808
RAF1#C-Raf_pS338	240	0.97 [0.92-1.02]	0.392	0.767
CHEK1#Chk1	240	0.97 [0.91-1.02]	0.319	0.726
GATA6#GATA6	218	0.97 [0.90-1.06]	0.592	0.835
GSK3A GSK3B#GSK3_pS9	240	0.97 [0.83-1.13]	0.719	0.901
NRG1#Heregulin	240	0.97 [0.92-1.03]	0.439	0.788
MRE11A#Mre11	240	0.97 [0.91-1.03]	0.424	0.774

Gene#Prot	CINSARC, High- vs. Low-risk			
	N	Odds ratio [95%CI]	p-value	q-value
NOTCH1#Notch1	240	0.97 [0.93-1.02]	0.322	0.726
PDK1#PDK1_pS241	240	0.97 [0.90-1.05]	0.547	0.826
PEA15#PEA15	240	0.97 [0.91-1.03]	0.345	0.728
AKT1S1#PRAS40_pT246	240	0.97 [0.91-1.02]	0.334	0.728
RB1#Rb	240	0.97 [0.91-1.04]	0.484	0.808
SMAD4#Smad4	240	0.97 [0.93-1.02]	0.349	0.728
ARAF#A-Raf	240	0.96 [0.90-1.03]	0.375	0.749
ANXA7#Annexin_VII	240	0.96 [0.91-1.01]	0.219	0.626
ERBB3#HER3_pY1289	240	0.96 [0.91-1.02]	0.235	0.643
IRF1#IRF-1	240	0.96 [0.91-1.01]	0.167	0.580
COPS5#JAB1	240	0.96 [0.91-1.02]	0.237	0.643
CDH2#N-Cadherin	240	0.96 [0.89-1.04]	0.414	0.774
PIK3R1 PIK3R2#PI3K-p85	240	0.96 [0.89-1.03]	0.341	0.728
PRKCA #PKC-alpha	240	0.96 [0.90-1.02]	0.262	0.643
RAB25#Rab25	240	0.96 [0.87-1.06]	0.507	0.808
SETD2#SETD2	240	0.96 [0.91-1.02]	0.261	0.643
PTPN11#SHP-2_pY542	240	0.96 [0.89-1.04]	0.388	0.766
YAP1#YAP_pS127	240	0.96 [0.85-1.08]	0.552	0.826
ABL1#c-Abl	240	0.96 [0.91-1.01]	0.151	0.570
RPS6KA1#p90RSK_pT359_S363	240	0.96 [0.91-1.03]	0.334	0.728
ACVRL1#ACVRL1	240	0.95 [0.89-1.01]	0.14	0.570
ESR1#ER-alpha_pS118	240	0.95 [0.84-1.08]	0.533	0.826
GSK3A GSK3B#GSK3-alpha-beta_pS21_S9	240	0.95 [0.82-1.10]	0.572	0.835
MAPK9#JNK2	240	0.95 [0.87-1.03]	0.267	0.643
MAP2K1#MEK1_pS217_S221	240	0.95 [0.88-1.03]	0.295	0.685
YAP1#YAP	240	0.95 [0.88-1.03]	0.346	0.728
YBX1#YB-1_pS102	240	0.95 [0.88-1.01]	0.176	0.584
MET#c-Met	240	0.95 [0.90-1.01]	0.166	0.580
BCL2A1#Bcl2A1	240	0.94 [0.86-1.02]	0.202	0.605
CAV1#Caveolin-1	240	0.94 [0.74-1.20]	0.694	0.899
PARK7#DJ-1	240	0.94 [0.87-1.02]	0.193	0.605
CDH1#E-Cadherin	240	0.94 [0.72-1.23]	0.709	0.899
GATA3#GATA3	240	0.94 [0.78-1.14]	0.607	0.841
IGFR1#IGF1R_pY1135_Y1136	240	0.94 [0.89-1.00]	0.0823	0.408
IRS1#IRS1	240	0.94 [0.87-1.03]	0.268	0.643
RPS6KA1#p90RSK	240	0.94 [0.87-1.01]	0.181	0.585
DIRAS3#DIRAS3	240	0.93 [0.86-0.99]	0.0777	0.401
ESR1#ER-alpha	240	0.93 [0.69-1.23]	0.657	0.883
PDK1#PDK1	240	0.93 [0.88-0.98]	2.03E-02	0.181
JUN#c-Jun_pS73	240	0.93 [0.88-0.98]	3.14E-02	0.242
CDKN1A#p21	240	0.93 [0.86-1.01]	0.149	0.570
BCL2#Bcl-2	240	0.92 [0.77-1.09]	0.416	0.774
RAB11A RAB11B#Rab11	240	0.91 [0.82-1.02]	0.159	0.572
CTNNB1#beta-Catenin	240	0.91 [0.77-1.07]	0.356	0.735
PREX1#PREX1	240	0.90 [0.73-1.10]	0.373	0.749
HSPA1A#HSP70	240	0.89 [0.71-1.11]	0.376	0.749
AKT1 AKT2 AKT3#Akt_pT308	240	0.88 [0.77-1.02]	0.15	0.570
FN1#Fibronectin	240	0.88 [0.73-1.05]	0.242	0.643
RAD50#Rad50	240	0.88 [0.83-0.94]	1.71E-03	0.063
CASP9#Caspase-9	15	0.87 [0.60-1.25]	0.537	0.826
COL6A1#Collagen_VI	240	0.87 [0.68-1.12]	0.359	0.735
ERCC5#ERCC5	240	0.87 [0.81-0.94]	2.17E-03	0.063
MAPK8#JNK_pT183_pY185	240	0.86 [0.79-0.93]	2.56E-03	0.063
KDR#VEGFR2	240	0.86 [0.75-0.99]	0.0725	0.385
PDCD4#PDCD4	240	0.85 [0.70-1.03]	0.156	0.570
AKT1 AKT2 AKT3#Akt_pS473	240	0.83 [0.68-1.01]	0.116	0.532
MAPK1 MAPK3#MAPK_pT202_Y204	240	0.79 [0.66-0.95]	3.46E-02	0.257

Gene#Prot	CINSARC, High- vs. Low-risk			
	N	Odds ratio [95%CI]	p-value	q-value
CTNNA1#alpha-Catenin	22	0.74 [0.60-0.93]	4.22E-02	0.277
PGR#PR	240	0.66 [0.45-0.96]	0.0675	0.376

Supplementary Table 10: List of breast cancer data sets included in the study

Reference	Source of data	N° of samples	Technological platform	N° of probe sets	N° of samples	N° of non-redundant non-metastatic, non-inflammatory, primary, invasive breast cancers	N° of non-redundant non-metastatic, non-inflammatory, primary, invasive breast cancers treated with primary surgery and with clinico-pathological annotations including survival
van de Vijver et al., NEJM 2002	http://microarray-pubs.stanford.edu/wound_NKI/	295	Agilent Hu25K	25K	254	254	254
van't Veer et al., Nature 2002	http://www.rii.com/publications/2002/vantveer.html	117	Agilent Hu25K	25K	117	117	97
Expression Project for Oncology (expO), 2005	https://expo.intgen.org/geo GEO: GSE2109	348	Affymetrix U133 Plus 2.0	54K	348	342	
Farmer P et al., Oncogene 2005	GEO: GSE1561	49	Affymetrix U133A	22K	49	49	
Minn AJ et al., Nature 2005	GEO: GSE2603	99	Affymetrix U133A	22K	99	99	82
Wang Y et al., Lancet 2005	GEO: GSE2034	286	Affymetrix U133A	22K	286	286	286
Hess KR et al., J Clin Oncol 2006	MDA133	133	Affymetrix U133A	22K	131	131	
Ivshina et al., Cancer Res 2006	GEO: GSE4922, GSE1456	448	Affymetrix U133 A+B	2x22K	448	448	406
Sotiriou C et al., J Natl Cancer Inst 2006	GEO: GSE2990	189	Affymetrix U133A	22K	80	80	80
Bonnefoi et al., Lancet Oncol 2007	GEO: GSE6861, GSE4779	161	Affymetrix X3P	61K	125	125	
Desmedt C etal., Clin Cancer Res 2007	GEO: GSE7390	198	Affymetrix U133A	22K	154	154	154
Klein A et al., Int J Cancer 2007	GEO: GSE6596	26	Affymetrix U133A	22K	24	20	
Marty et al., Breast Cancer Res 2008	GEO: GSE13787	23	Affymetrix U133 Plus 2.0	54K	23	23	
Merritt WM et al., N Engl J Med 2008	Array Express: E-MTAB-158	130	Affymetrix U133AAofAv2	23K	130	125	119
Schmidt M etal., Cancer Res 2008	GEO: GSE11121	200	Affymetrix U133A	22K	200	200	200
Yu K et al., PLoS Genet 2008	GEO: GSE5364	196	Affymetrix U133A	22K	183	183	
Bos et al., Nature 2009	GEO: GSE12276	204	AffymetrixU133 Plus 2.0	54K	204	204	196
Hoeflich et al., Clin Cancer Res 2009	GEO: GSE12763	30	Affymetrix U133 Plus 2.0	54K	30	30	
Zhang Y et al., Breast Cancer Res Treat 2009	GEO: GSE12093	136	Affymetrix U133A	22K	136	136	136
Miller WR et al., Breast Cancer Res 2010	GEO: GSE5462	116	Affymetrix U133A	22K	116	116	
Barry et al., J Clin Oncol 2010	GEO: GSE23593	50	Affymetrix U133 Plus 2.0	54K	50	50	
Korde et al., Breast Cancer Res Treat 2010	GEO: GSE18728	61	Affymetrix U133 Plus 2.0	54K	61	61	
Prat A et al., Breast Cancer Res 2010	GEO: GSE18229	337	Agilent Hu25K	25K	264	264	235
Silver et al., J Clin Oncol 2010	GEO: GSE18864	84	Affymetrix U133 Plus 2.0	54K	84	84	
Tabchy A et al., Clin Cancer Res 2010	GEO: GSE20271	178	Affymetrix U133A	22K	178	178	
Jonsson et al., Breast Cancer Res 2010	GEO: GSE22133	359	Swegene H_v2.1.1 55K	55K	346	346	339
Chen et al., Breast Cancer Res Treat 2010	GEO: GSE10780	185	Affymetrix U133 Plus 2.0	54K	42	42	
Popovici V et al., Breast Cancer Res 2010	GEO: GSE20194	278	Affymetrix U133A	22K	91	91	
Iwamoto T et al., J Natl Cancer Inst 2011	GEO: GSE22093, GSE22597	247	Affymetrix U133A	22K	100	100	
Desmedt et al., J Clin Oncol 2011	GEO: GSE16446	120	Affymetrix U133 Plus 2.0	54K	120	120	
Guedj et al., Oncogene 2011	Array Express: E-MTAB-365	537	Affymetrix U133 Plus 2.0	54K	452	452	157
Hatzis C et al., JAMA 2011	GEO: GSE25066	508	Affymetrix U133A	22K	504	504	
Sabatier R et al., PLoS One 2011	GEO: GSE31448	353	Affymetrix U133 Plus 2.0	54K	286	280	280
TCGA, Nature 2012	TCGA Data Portal - BRCA -	1215	Illumina, RNAseq V2	20K	1092	1071	1070
Ellis et al., Nature 2012	GEO: GSE29442, GSE35186	201	Agilent-014850 4x44K	44K	201	201	
Curtis et al., Nature 2012	https://www.cbioportal.org/study/summary?id=brca_metabric	2136	Illumina HT 12	49K	1974	1964	1944
	TOTAL	10233			8982	8930	6035
