# Supplementary material for: The CINSARC signature predicts the clinical outcome in patients with Luminal B breast cancer
Source: NPJ Breast Cancer. 2021 May 5;7:48. doi: 10.1038/s41523-021-00256-2 (PMC8099860; doi:10.1038/s41523-021-00256-2)
Supplement: Supplementary file 2 — Reporting Summary [file 41523_2021_256_MOESM2_ESM.pdf]

nature research

Corresponding author(s): Pr François BERTUCCI

Last updated by author(s): Apr 1, 2021

Reporting Summary

Nature Research wishes to improve the reproducibility of the work that we publish. This form provides structure for consistency and transparency in reporting. For further information on Nature Research policies, see our Editorial Policies and the Editorial Policy Checklist.

Statistics

For all statistical analyses, confirm that the following items are present in the figure legend, table legend, main text, or Methods section.

n/a	Confirmed	
☐	☒	The exact sample size (n) for each experimental group/condition, given as a discrete number and unit of measurement
☐	☒	A statement on whether measurements were taken from distinct samples or whether the same sample was measured repeatedly
☐	☒	The statistical test(s) used AND whether they are one- or two-sided<br>*Only common tests should be described solely by name; describe more complex techniques in the Methods section.*
☐	☒	A description of all covariates tested
☐	☒	A description of any assumptions or corrections, such as tests of normality and adjustment for multiple comparisons
☐	☒	A full description of the statistical parameters including central tendency (e.g. means) or other basic estimates (e.g. regression coefficient) AND variation (e.g. standard deviation) or associated estimates of uncertainty (e.g. confidence intervals)
☐	☒	For null hypothesis testing, the test statistic (e.g. F, t, r) with confidence intervals, effect sizes, degrees of freedom and P value noted<br>*Give P values as exact values whenever suitable.*
☐	☒	For Bayesian analysis, information on the choice of priors and Markov chain Monte Carlo settings
☒	☐	For hierarchical and complex designs, identification of the appropriate level for tests and full reporting of outcomes
☒	☐	Estimates of effect sizes (e.g. Cohen's d, Pearson's r), indicating how they were calculated

Our web collection on statistics for biologists contains articles on many of the points above.

Software and code

Policy information about availability of computer code

Data collection	All data collection methods and software used to analyze the data are outlined in the manuscript.
Data analysis	Normalization of public data sets were done by Robust Multi-Array (RMA) with the oligo R package (version 1.46.0) for Affymetrix data and by quantile normalization with the limma R package (version 3.38.3) for other microarray platforms. Supervised analysis was done using a moderated t-test with empirical Bayes statistic included in the limma R package (version 3.38.3). For correction of the multiple-testing hypothesis, False Discovery Rate (FDR) was assessed using qvalue R package (version 2.14.1) (Storey et al., Annals of Statistics, 2003). Several multigene signatures were applied to each dataset separately: CINSARC (Chibon et al., Nat. Med. 2010), PAM50 (Parker et al., J Clin Oncol 2009) and 107-gene predictive signatures (Bertucci et al., Ann. Oncol. 2013) who were based on nearest-centroid classification using genes, data and distance method described in each respective study. Also were applied Rbsig (Maloni et al., Oncotarget 2016), E2F regulon (Turner et al., J. Clin Oncol. 2019), ICR (Roelands et al., J. Immunother. Cancer 2020), TIS (Ayers et al., J. Clin Invest. 2017), TLS (Coppola et al., Am. J. Pathol. 2011),Palmer's immune modules (B-cells, T-cells, and CD8 T-cells) (Palmer et al., BMC Genomics 20106) and the Rooney' cytolytic activity score (Rooney et al., Cell 2015) signatures who were based on a Z-score metagene using gene list described in each respective study. Statistics analysis was done with the stats R package (version 3.5.2) and the survival R package (version 3.1-12) for survival analysis.

For manuscripts utilizing custom algorithms or software that are central to the research but not yet described in published literature, software must be made available to editors and reviewers. We strongly encourage code deposition in a community repository (e.g. GitHub). See the Nature Research guidelines for submitting code & software for further information.

Data

Policy information about availability of data

All manuscripts must include a data availability statement. This statement should provide the following information, where applicable:

- Accession codes, unique identifiers, or web links for publicly available datasets
- A list of figures that have associated raw data
- A description of any restrictions on data availability

The data generated and analysed during this study are described in the following data record: https://doi.org/10.6084/m9.figshare.14350871. All data sets of primary breast cancer were downloaded from the Gene Expression Omnibus (GEO, https://www.ncbi.nlm.nih.gov/geo/), ArrayExpress (https://www.ebi.ac.uk/arrayexpress/), Genomic Data Commons (GDC, https://portal.gdc.cancer.gov/) and cBioPortal (https://www.cbioportal.org/) databases. All accession IDs are provided in Supplementary Table 10 (Table S10 revised.xlsx), which is included with the data record. The data underlying the figures and tables are contained in the files 'Goncalves_supporting_data.xlsx' and 'Table S8.xlsx', which are included with the data record. A detailed list of the data underlying each figure and table is also available in the file 'Goncalves_2021_underlying_data_list.xlsx', which is included with the data record.

Field-specific reporting

Please select the one below that is the best fit for your research. If you are not sure, read the appropriate sections before making your selection.

☒ Life sciences ☐ Behavioural & social sciences ☐ Ecological, evolutionary & environmental sciences

For a reference copy of the document with all sections, see nature.com/documents/nr-reporting-summary-flat.pdf

Life sciences study design

All studies must disclose on these points even when the disclosure is negative.

Sample size	Sample size was determined by availability of gene expression and clinicopathological data at the time of analyses (July 2019). Our series contained 8982 non-redundant invasive breast cancer samples.
Data exclusions	No data was excluded from the analysis.
Replication	Not relevant for our study
Randomization	Not relevant for our study
Blinding	Not relevant for our study

Reporting for specific materials, systems and methods

We require information from authors about some types of materials, experimental systems and methods used in many studies. Here, indicate whether each material, system or method listed is relevant to your study. If you are not sure if a list item applies to your research, read the appropriate section before selecting a response.

Materials & experimental systems

n/a	Involved in the study	
☒	☐	Antibodies
☒	☐	Eukaryotic cell lines
☒	☐	Palaeontology and archaeology
☒	☐	Animals and other organisms
☐	☒	Human research participants
☒	☐	Clinical data
☒	☐	Dual use research of concern

Methods

n/a	Involved in the study	
☒	☐	ChIP-seq
☒	☐	Flow cytometry
☒	☐	MRI-based neuroimaging

Human research participants

Policy information about studies involving human research participants

Population characteristics	Our study is based upon public data from published studies on breast cancer in which population characteristics are detailled and could be found using accession codes provide in the present supplementary table 10. All cases were invasive breast carcinomas profiled using DNA microarrays or RNA-sequencing with expression and clinicopathological data available. All samples are pre-treatment samples (operative specimen or diagnostic biopsy before neo-adjuvant chemotherapy). The detailed characteristics of patients and tumors analysed in the present study are available in a supplementary file.

Recruitment	Our study is based upon publicly available transcriptomic data of invasive primary breast cancer enrolled in 36 retrospective studies published over a 10-year period between 2002 and 2012. The data collection was done in our laboratory in real time after each publication.
Ethics oversight	Our in silico study is based upon public data from published studies in which the informed patients' consent to participate and the ethics and institutional review board were already obtained by authors. The study was approved by our institutional review board (Comité d’Orientation Stratégique, COS).

Note that full information on the approval of the study protocol must also be provided in the manuscript.
